# Identification of *SLC15A3* as a shared prognostic biomarker for AML and COVID-19 via integrative bioinformatics, machine learning and Mendelian randomization

**DOI:** 10.3389/fonc.2026.1911550

**Published:** 2026-10-14

**Authors:** Miaomiao Liu, Junhui Zhu, Huan Li

**Affiliations:** 1 College of Basic Medicine, Beihua University, Jilin, China; 2 College of Public Health, Beihua University, Jilin, China

**Keywords:** AML, COVID-19, ML, MR, SLC15A3

## Abstract

Acute myeloid leukemia (AML) is a lethal hematological malignancy with poor overall prognosis.COVID-19 co-infection further complicates clinical management and worsens survival outcomes of AML patients, highlighting the need to explore the molecular interplay between the two diseases. In this study, we initially screened 109 common differentially expressed genes shared by AML and COVID-19 from public transcriptomic datasets, which were closely involved in tumor progression and viral pathogenesis. We further characterized their biological functions, constructed protein interaction and TF-miRNA regulatory networks, and established an AML prognostic model to evaluate the tumor immune microenvironment. Combining survival analysis, clinical correlation, immune infiltration assessment and m^6^A modification analysis, *SLC15A3* was preliminarily screened as a candidate prognostic gene for AML. Subsequent weighted gene co-expression network analysis (WGCNA), machine learning (ML) and Mendelian randomization (MR) analyses further identified *SLC15A3* as a promising prognostic biomarker for AML. Finally, RT-qPCR and Western blotting assays verified its aberrant expression in AML cells. Consistent with bioinformatic prediction,*SLC15A3* serves as a shared candidate biomarker for AML and COVID-19,although its mechanistic role linking AML and SARS-CoV-2-related inflammation requires further functional validation.

## Introduction

Acute myeloid leukemia (AML) is an aggressive hematological malignancy driven by aberrant proliferation and blocked differentiation of bone-marrow myeloid precursors, associated with poor overall survival (1). Patients with AML have compromised immune function, rendering them highly vulnerable to severe infection (2). During the COVID-19 pandemic, AML patients experienced substantially worse clinical outcomes upon SARS-CoV-2 infection; importantly, patients with hematological malignancies including AML were excluded from pivotal COVID-19 clinical trials (3). This creates an unmet clinical need to understand molecular crosstalk between AML and COVID-19, which may inform risk stratification and therapeutic strategies for co-infected patients (4).

Established AML prognostic markers include *NPM1*, *FLT3-ITD*, and *CEBPA*, which guide current risk stratification in clinical practice (5). Nevertheless, how COVID-19-related molecular signals intersect with AML progression and patient prognosis remains poorly defined (6). Gene expression is tightly shaped by multiple regulatory layers, including transcription factors (TFs), microRNAs (miRNAs),and N^6^-methyladenosine (m^6^A) RNA modification (7–9). These regulators coordinate malignant transformation and host immune-inflammatory responses, and may mediate molecular crosstalk between AML and SARS-CoV-2 infection (10, 11). Transcription factors bind to gene promoters to activate or repress transcription, governing cell fate in normal and malignant hematopoiesis (12); miRNAs are short non-coding RNAs that trigger mRNA degradation or translational repression to remodel tumor microenvironment and antiviral immunity (13, 14); and m^6^A represents a dynamic, reversible RNA modification governing mRNA stability, splicing and translation in both cancer and viral infection (15–17). Collectively, these regulatory systems shape the tumor microenvironment (TME),anti-tumor immunity, and inflammatory host responses to viral infection (18).

Machine-learning and Mendelian randomization (MR) offer robust analytical tools for mining multi-omics public datasets (19). Conventional transcriptomic analyses identify gene-expression correlations, whereas machine-learning prioritizes high-performance biomarker candidates from high-dimensional data.MR leverages genetic variants as instrumental variables to infer putative causal relationships, helping distinguish true biological associations from non-causal correlations observed in observational transcriptome data (20). Such multi-modal analytical workflows have been widely applied to uncover molecular signatures of cancer-virus interaction, supporting their suitability for our research question (21).

In the present study, we aimed to identify shared prognostic biomarkers for AML in the context of COVID-19 comorbidity. We integrated multiple public transcriptomic datasets to screen shared differentially expressed genes between AML and COVID-19.We combined network biology, tumor-microenvironment characterization, m^6^A modification profiling, machine-learning diagnostic modelling, two-sample MR and cell-line experimental validation to nominate and verify candidate prognostic genes. Our work identifies *SLC15A3* as a promising protective prognostic biomarker for AML, which is computationally predicted to represent a shared molecular link with COVID-19-related inflammation, awaiting further functional validation.

## Materials and methods

### Dataset download and preprocessing

COVID-19-related mRNA datasets were retrieved from GEO (GSE180226) and GSEA databases.GSE180226 contained 10 healthy and 26 COVID-19 blood samples; the GSEA COVID-19 dataset included 32 infected and 16 normal samples. AML transcriptomic data were obtained from TCGA (151 AML patients) and GTEx (70 normal bone-marrow controls).DEGs were screened using the limma package with thresholds P < 0.05 and |log_2_FC| ≥ 1,followed by hierarchical clustering with ggplot2. Additional AML datasets (GSE2191, GSE9476, GSE96535, GSE33223, GSE57194) from GEO were merged and batch-corrected; data quality was assessed by box plots and PCA(Principal Component Analysis). Note: The AML group corresponds to transcriptomic samples from patients diagnosed with acute myeloid leukemia; the CON (control) group comprises normal bone-marrow samples obtained from GTEx and GEO public datasets.

### PPI network construction and functional enrichment analysis

To further investigate the function of DEmRNAs in the network at the protein level, the Search Tool for the Retrieval of Interacting Genes (STRING) database was used to construct PPI network (22, 23). DEmRNAs were mapped to STRING, and the median confidence score was 0.8,the protein interactions were evaluated. Cytoscape version 3.7.1 was used to construct the PPI network, and the plug-in “cytoHubba” was used to analyze the sub-network (24). The biological processes in Gene Ontology (GO) and the Kyoto Encyclopedia of Genes and Genomes (KEGG) pathways (P <0.05) were used for the biological functional analysis of DEmRNAs in the network (23). The significant enrichment results were visualized using the ggplot2 R package. For supplementary verification of protein interaction relationships, the GeneMANIA database was applied for additional PPI and signaling pathway analysis.

### TF-gene and TF-miRNA regulatory network analysis

#### Transcription factor-gene interactions

NetworkAnalyst 3.0 was utilized to predict the regulatory interactions between candidate genes and transcription factors (TFs) based on public ChIP-seq datasets (25).The TF-gene regulatory network was constructed and visualized in Cytoscape 3.7.1.To further explore the post-transcriptional regulatory mechanism, a TF-miRNA coregulatory network was established using literature-curated interaction data from the RegNetwork database (26).All regulatory networks were visualized via Cytoscape 3.7.1.

#### Drug-ingredient-target network construction

Genes involved in drug-disease crossover were screened. A number of targets corresponding to COVID-19 active ingredients were imported into Cytoscape3.7.1, and visualization of the network was achieved by creating a network diagram of drug-target disease interactions.

#### Prognostic and immune infiltration analysis

Tumor microenvironment indices, including immune, stromal, and ESTIMATE scores, were calculated for TCGA-AML samples, and intergroup differences were compared using one-way ANOVA. Optimal cutoff values for each score were determined by X-tile software to stratify patients into high-and low-subgroups, and Kaplan-Meier survival analysis was performed to assess the prognostic discrepancies between stratified cohorts (27, 28). Univariate Cox regression analysis was subsequently utilized to screen prognosis-related common differentially expressed genes, with *SLC15A3* finally identified as a core prognostic biomarker for AML (29). The differential expression pattern and prognostic predictive efficacy of *SLC15A3* were further validated in AML populations. Furthermore, partial Spearman correlation analysis was conducted to elucidate the potential association between *SLC15A3* expression and tumor immune infiltration abundance, and scatter plots were generated for intuitive visualization of statistical correlations. All statistical analyses were two-sided, and *P* < 0.05 was regarded as the threshold of statistical significance.

#### TCGA data preprocessing and m^6^A modification analysis

The gene expression data of 200 AML patients were downloaded from the TCGA database (http://cancergenome.nih.gov/). The gene expression levels were presented as log_2_-transformed (FPKM + 1) values. The merged matrix included 200 AML patients in the TCGA and 6 AML patients with 6 healthy donors in the GEO(GSE96535).Somatic mutation information was obtained from the TCGA database, and datasets were obtained for copy number variant (CNV)assays. Unsupervised clustering of 23 m^6^A regulators was performed based on differential gene expression. The results were assayed using R 3.7.2 and R Bioconductor software.

#### Gene set variation analysis (GSVA) and functional annotation

For an in-depth analysis of the biological processes that associate with the different m^6^A modification patterns, the “GSVA” R package for GSVA enrichment analysis was utilized (30).The gene set “c2.cp.kegg.v6.2. symbols” was downloaded from the MSigDB database and used to run GSVA analysis. For functional annotation of genes, we utilized cluster analysis and an FDR with P<0.05.

#### Estimation of the immune cells infiltrating the TME

A gene set of various human immune cell subtypes was obtained from Charoentong et al. Single-sample Gene Set Enrichment Analysis (ssGSEA) was used to calculate the separate enrichment scores for each sample and gene pair. The analysis was used to determine the relative abundance of each cell type that infiltrated the TME for each sample (30).

#### WGCNA co-expression analysis

Differential expression analysis was conducted using a combination of bioinformatics tools, including the limma, dplyr, pheatmap, and ggplot2 packages. This analysis allowed us to generate heatmaps and volcano plots, providing visual representations of gene expression differences between groups. We performed Weighted Gene Co-expression Network Analysis (WGCNA) using the WGCNA package to produce the gene dendrogram and identify clusters of highly correlated genes. To pinpoint genes of particular interest, we then used the Venn Diagram package to find the intersection between the differentially expressed genes and the genes identified in the WGCNA modules.

#### Machine learning model construction and signature gene screening

The merged AML datasets were divided into a training cohort (GSE2191, GSE9476, GSE96535) and an independent validation cohort (GSE33223, GSE57194). Multiple machine learning algorithms and their combinations, including plsRglm, Lasso, Stepglm,E lastic net, Ridge and glmBoost, were compared to construct diagnostic models. Machine learning diagnostic models were established using the randomForestSRC, ComplexHeatmap and pROC packages. Repeated 5-fold cross-validation (3 repetitions) and within-fold grid search for hyperparameter optimization were performed on the training set to reduce overfitting and prevent data leakage. A fixed random seed (123) was set for all data splitting and modeling procedures to ensure reproducibility. Model classification performance was visualized by heatmaps and evaluated by receiver operating characteristic (ROC) curves, with AUC > 0.7 defined as effective diagnostic capability. The plsRglm algorithm was applied to screen core signature genes, which were further validated via volcano plots, box plots, correlation analysis and ROC curves.

#### Analysis of immune cell infiltration

To investigate the potential involvement of the immune system in our study, we conducted an immune cell infiltration analysis on our dataset, using histograms, heatmaps, and box plots. We also generated scatter plots to explore the associations between our identified model genes and the immune cell infiltration patterns. Such analyses identify potential connections between gene expression and immune cell presence, providing insights into the interplay between genetic factors and immune responses in the disease context.

#### Mendelian randomization analysis

To further investigate effects of candidate genes on AML, we employed a MR approach using publicly available data. We obtained outcome data for AML from the FinnGen database (https://www.finngen.fi/en/access_results) and protein exposure data from the pQTL database (https://www.decode.com/summarydata/).For data analysis, we utilized a suite of genetic and genomic analyses tool, such as VariantAnnotation, Gwasglue and TwoSampleMR packages, then visualized the results using scatter plot, forest plot, funnel plot, leaveoneout plot.

#### Verification of key genes by RT-qPCR, WB

The cells used in this study include normal human bone marrow stromal cell line HS-5(human immortalized bone marrow stromal cell line, normal control),and the AML cell lines THP-1 and HL-60. The cells were cultured in RPMI 1640 (containing 10% fetal bovine serum,100μg/mL streptomycin and 100 u/mL penicillin) in an incubator at 37 °C and 5% CO2.We conducted the test of RT-qPCR and WB assay according to the instructions of the reagents.

## Results

### Identification of shared differentially-expressed genes between AML and COVID-19

To identify shared molecular signatures underlying AML and COVID-19 comorbidity, we analyzed three public transcriptomic datasets (GSE180226,GSEA COVID-19 dataset, and integrated TCGA-GTEx AML dataset).The complete analytical workflow is summarized in **Figure 1**.

**Figure 1:**
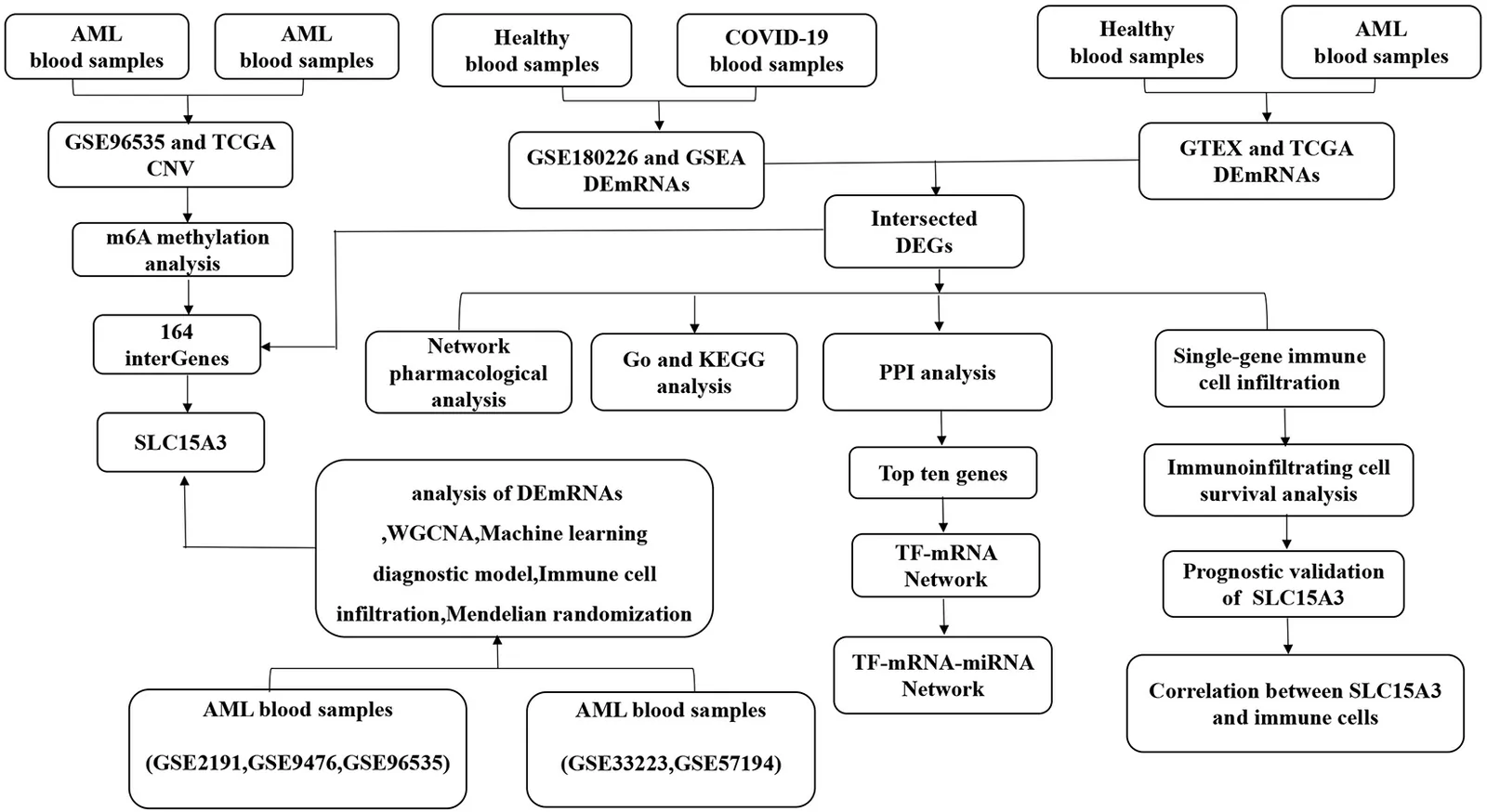
Flowchart of this study.

We detected 4927 differentially-expressed genes (DEGs;2335 up-regulated,2592 down-regulated) in GSE180226,4012 DEGs (1017 up-regulated,2995 down-regulated) in the GSEA COVID-19 cohort, and 1622 DEGs (850 up-regulated, 772 down-regulated) in the TCGA-GTEx AML cohort. Heatmaps (**Figures 2A–C**) provide an overview of global transcriptomic patterns across datasets, while corresponding volcano plots (**Figures 2D–F**) illustrate statistically significant differentially expressed genes identified by limma analysis. The full lists of DEGs are provided in **Supplementary Tables S1**–**S3**.

**Figure 2:**
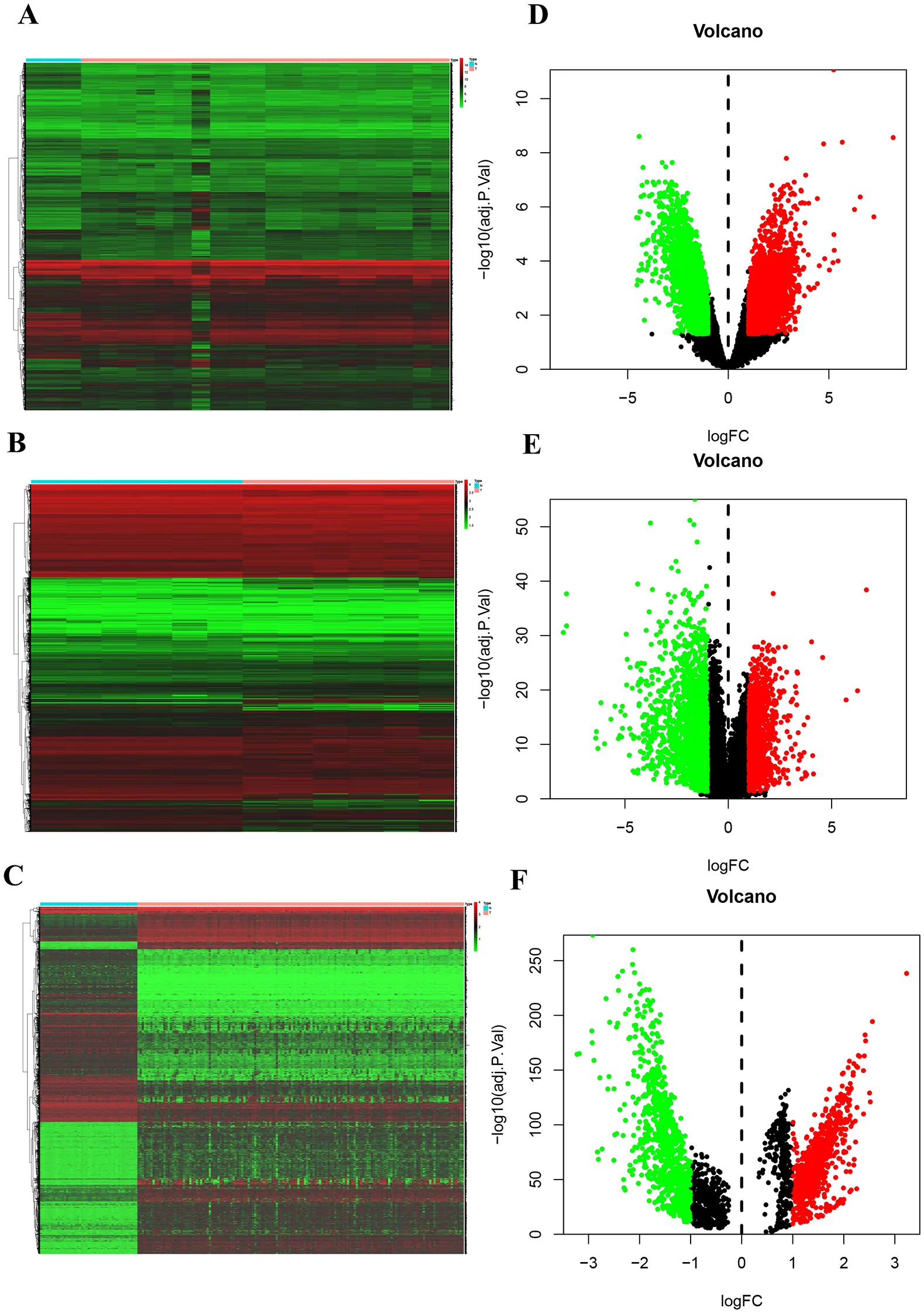
Differential mRNA expression profiles of COVID-19 and AML datasets. Hierarchical-clustering heatmaps and corresponding volcano plots illustrate transcriptomic alterations across three public cohorts:GSE180226 (COVID-19),GSEA COVID-19 dataset, and integrated TCGA-GTEx AML dataset. The GSE180226 cohort included 26 COVID-19 patients and 10 healthy controls; the GSEA COVID-19 dataset contained 32 infected and 16 normal samples; the merged TCGA-GTEx AML dataset comprised 151 AML cases and 70 normal bone-marrow controls. Differential expression analysis was implemented using the R limma package. Empirical Bayesian moderated t-tests were used to compute P values, and the Benjamini–Hochberg approach was applied for FDR correction. Genes satisfying thresholds of |log_2_FC| ≥ 1 and P < 0.05 were defined as DEGs. **(A–C)** Row-wise z-score normalized hierarchical clustering heatmaps showing global expression patterns of DEGs for GSE180226, GSEA COVID-19 dataset, and TCGA-GTEx AML dataset, respectively. **(D–F)** Corresponding volcano plots for each dataset. Red dots denote significantly up-regulated mRNAs, green dots represent significantly down-regulated mRNAs, and black dots indicate genes without statistically significant expression changes. AML indicates acute myeloid leukemia patient samples; CON indicates normal bone-marrow control samples. Heatmaps display row-wise z-score-normalized relative gene expression and serve to provide an overview of global transcriptomic patterns. Conclusions on group-level differential expression are drawn from limma-based statistical testing and volcano plots, not visual evaluation of heatmap color gradients.

### Functional and network characterization of shared DEGs

To explore the biological functions of genes jointly perturbed in AML and COVID-19, we constructed a protein-protein interaction (PPI) network using the shared DEG set. The PPI network contained 109 nodes and 83 protein-protein interaction edges (**Figure 3A**). Using the cytoHubba plugin, we extracted a sub-network and prioritized ten highly-connected hub genes: *HMOX1, MYC, FOS, SNCA, BCL2L, RUNX2, SMC3, CD68, EEF2, MCM2* (**Figure 3B**). The Venn diagram in **Figure 3C** visualizes the overlap yielding these 109 shared DEGs (provided in **Table 1**).

**Figure 3:**
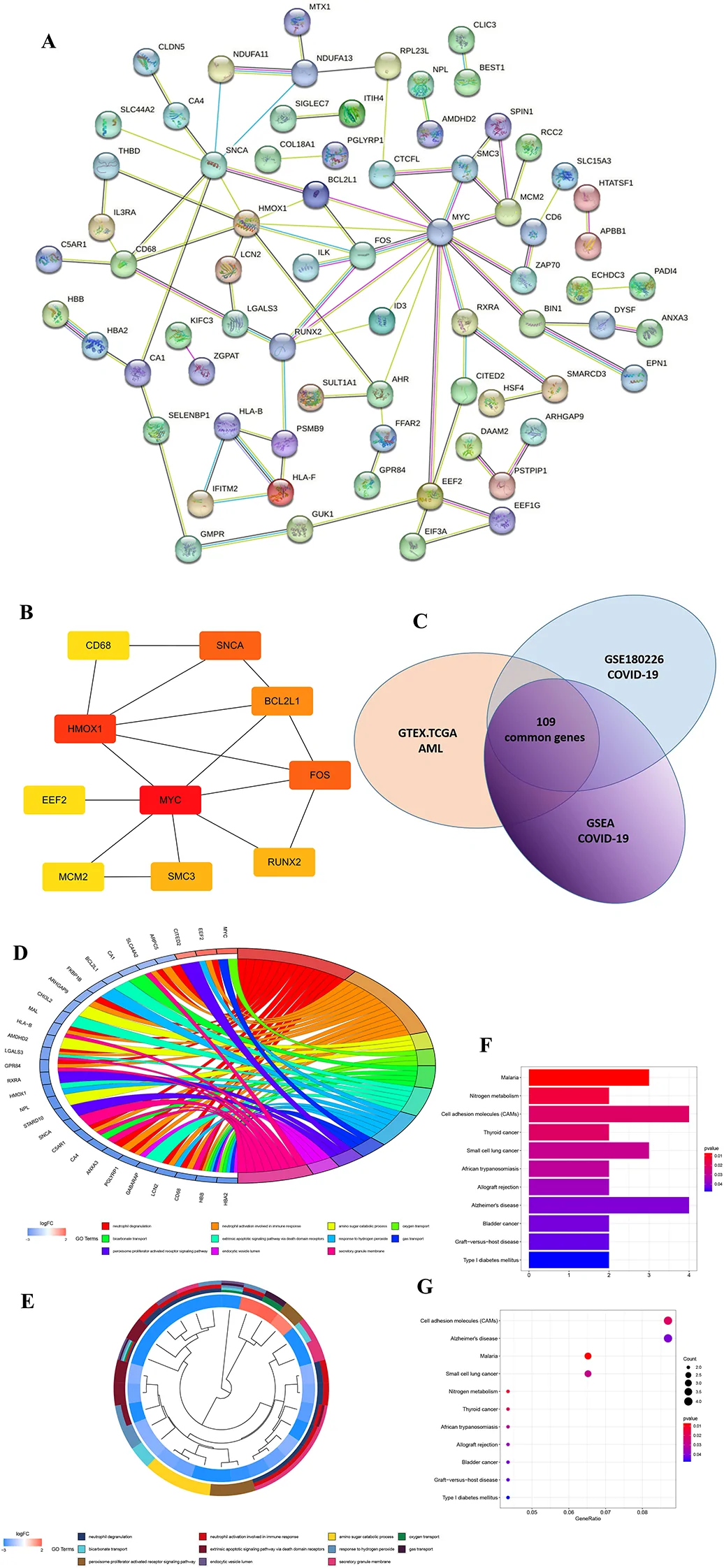
Screening and functional enrichment of shared differentially expressed mRNAs (DEGs) between COVID-19 and AML. Shared DEGs among GSE180226, GSEA-COVID-19 and TCGA-GTEx AML datasets were identified using the R limma package, with cut-offs of |log_2_FC| ≥ 1 and P < 0.05.Empirical Bayesian moderated t-tests were used to calculate P values, and the Benjamini-Hochberg method was applied for FDR correction. **(A)** Protein-protein interaction (PPI) network constructed from shared DEGs. **(B)** Sub-network displaying core hub genes extracted from the full PPI network. **(C)** Venn diagram showing 109 common DEGs across COVID-19 and AML transcriptomic cohorts. **(D, E)** Circos plots presenting Gene Ontology (GO) enrichment results, including biological process (BP), cellular component (CC), and molecular function (MF) categories. **(F, G)** KEGG pathway enrichment analyses for the shared DEGs. GO and KEGG enrichment analyses were implemented via the clusterProfiler R package. Enrichment terms with P < 0.05 and FDR < 0.05 were regarded as statistically significant.

**Table 1:** 109 Conmon genes of COVID-19 and AML.

109 Conmon genes of COVID-19 and AML
ECHDC3 ANXA3 CRIP1 CA4 SDR39U1 IFITM2 PSTPIP1 GYPC SP110 H19 HLA-F SLC6A8 GABARAP TPP1 MYC SLC15A3 BIN1 HLA-B SERF2 MAL ZAP70 NDUFA11 RCC2 RPS19BP1 SH3BP5 ARPC5 AMDHD2 COL18A1 METRNL SMC3 PGLYRP1 RXRA DAPK2 DYSF CHI3L2 SELENBP1 HSF4 CCDC107 CLDN5 APBB1 IL3RA GUK1 GMPR PDE4DIP KIFC3 NDUFA13 SYPL1 SIGLEC7 RUNX2 CD68 GPR84 SPOCD1 NPL CA1 DTX2 ZGPAT BEST1 ILK AK1 HIST1H2BC SMARCD3 ZBP1 PADI4 PRELID1 CLIC3 ARHGAP9 FFAR2 YPEL3 MRPL23 SLC44A2 GCHFR SNCA FOS ID3 AHR TMEM134 LGALS3 TMEM176B THBD HBA2 FKBP1B HMOX1 ITIH4 EPN1 LIN37 MCM2 WASH7P EIF3A DAAM2 PRR5 MTX1 HBB EEF2 HS1BP3 SULT1A1 SP140 LCN2 C5AR1 STARD10 TNNC1 HTATSF1 EEF1G PSMB9 CD6 SPIN1 ZBED4 CITED2 BCL2L1 ST6GALNAC2

GO and KEGG enrichment analyses were performed on these 109 shared genes to interpret affected biological pathways. Neutrophil-related immune responses, including neutrophil degranulation and neutrophil activation involved in immune response, represented the most prominent GO biological-process terms. Top enriched KEGG pathways included malaria, nitrogen metabolism, cell adhesion molecules (CAMs),thyroid cancer, small-cell lung cancer, and allograft rejection (**Figures 3D–G**; **Table 2**). Collectively, these enrichment results highlight that immune-and inflammatory-related pathways are major shared molecular features of AML and COVID-19.

**Table 2:** GO biological process terms and KEGG enriched pathways for conmon genes of COVID-19 and AML.

Term	Genes	P-value
GO biological process		
neutrophil degranulation	ANXA3/HLA-B/ARPC5/PGLYRP1/CD68/GPR84	3.09E-06
neutrophil activation involved in immune response	ANXA3/HLA-B/ARPC5/PGLYRP1/CD68/GPR84/ARHGAP9	3.31E-06
amino sugar catabolic process	AMDHD2/CHI3L2/NPL	4.60E-05
oxygen transport	MYC/HBA2/HBB	7.26E-05
bicarbonate transport	CA4/CA1/HBA2/HBB	9.46E-05
extrinsic apoptotic signaling pathway via death domain receptors	GABARAP/MAL/LGALS3/HMOX1/BCL2L1	0.000138987
response to hydrogen peroxide	HBA2/FKBP1B/HMOX1/HBB/EEF2/LCN2	0.000166212
gas transport	MYC/HBA2/HBB	0.000178257
peroxisome proliferator activated receptor signaling pathway	RXRA/STARD10/CITED2	0.000207126
endocytic vesicle lumen	PGLYRP1/HBA2/HBB	0.000160363
secretory granule membrane	CA4/HLA-B/CD68/GPR84/SLC44A2/SNCA/LGALS3/C5AR1	0.000245524
KEGG pathway		
Malaria	GYPC/HBA2/HBB	0.007132069
Nitrogen metabolism	CA4/CA1	0.013584895
Cell adhesion molecules (CAMs)	HLA-F/HLA-B/CLDN5/CD6	0.02093503
Thyroid cancer	MYC/RXRA	0.021164725
Small cell lung cancer	MYC/RXRA/BCL2L1	0.02815305
African trypanosomiasis	HBA2/HBB	0.030115866
Allograft rejection	HLA-F/HLA-B	0.036777377
Alzheimer's disease	NDUFA11/APBB1/NDUFA13/SNCA	0.041086936
Bladder cancer	MYC/DAPK2	0.042110556
Graft-versus-host disease	HLA-F/HLA-B	0.043949404
Type I diabetes mellitus	HLA-F/HLA-B	0.047715422

### Regulatory-network and compound-target analysis of shared DEGs

To uncover transcriptional and post-transcriptional regulatory mechanisms governing the shared DEGs, we constructed transcription-factor-gene and TF-miRNA co-regulatory networks.

The TF-gene regulatory network comprised 121 nodes and 111 edges (**Figure 4A**). Among hub shared genes,*HMOX1* was regulated by 17 transcription factors and *MYC* by 13 transcription factors (**Table 3**). The integrated TF-miRNA co-regulatory network contained 248 nodes and 238 edges, showing that 111 TFs and 127 miRNAs interact with the identified hub genes (**Figure 4B**; **Table 4**). These results demonstrate complex multi-layer transcriptional and post-transcriptional control acting on genes common to AML and COVID-19.

**Figure 4:**
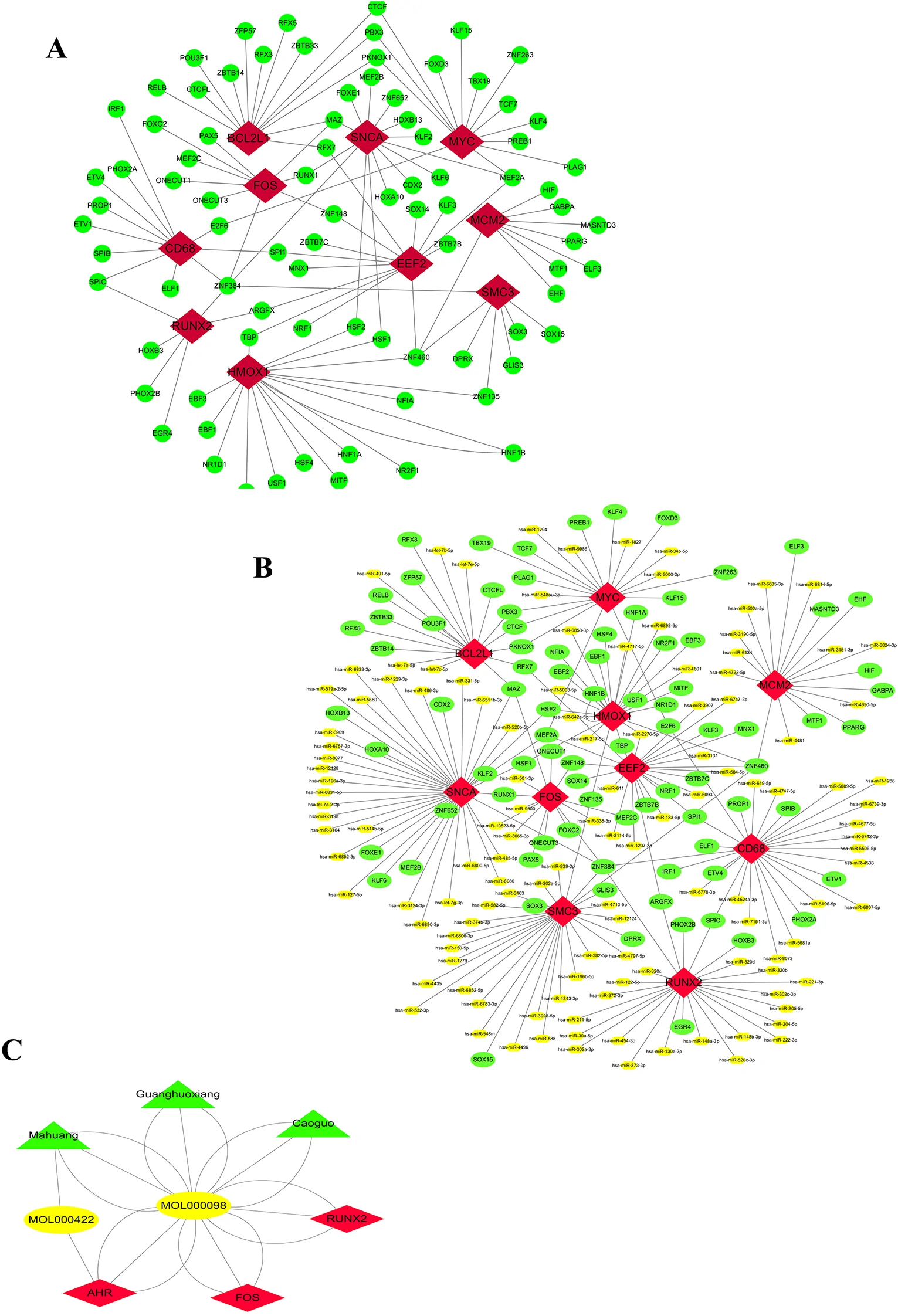
Transcription-factor (TF),miRNA and pharmacological network analyses of shared differentially expressed genes (DEGs) between COVID-19 and AML. Shared DEGs were obtained from GSE180226,GSEA-COVID-19 and TCGA-GTEx AML datasets using the R limma package. Empirical Bayesian moderated t-tests were applied for differential expression calculation, and the Benjamini-Hochberg procedure was used for FDR correction. Genes satisfying |log_2_FC| ≥ 1 and P < 0.05 were identified as significant DEGs. **(A)** TF-mRNA regulatory network illustrating interactions between hub shared DEGs and their predicted transcription factors. **(B)** Integrated TF-mRNA-miRNA co-regulatory network displaying transcriptional and post-transcriptional regulatory relationships of core hub genes. **(C)** Herb-target pharmacological network showing potential binding associations between hub DEGs and candidate herbal components. Molecular interaction predictions were retrieved from standard bioinformatics databases. Only statistically significant interaction pairs were retained for network construction.

**Table 3:** TF–gene interaction.

Identified genes	Transcription factors
HMOX1	ZNF460,HNF1B,TBP,ZNF135,HSF1,EBF1,EBF3,EBF2,NR2F1,MITF,USF1,NR1D1,HNF1A,NFIA HSF2,HSF4
MYC	PBX3,PKNOX1,FOXD3,PREB1,CTCF,KLF4,KLF15,TBX19,E2F6,MEF2A,ZNF263,PLAG1,TCF7
FOS	ZNF384,MEF2C,RUNX1,PAX5,ONECUT1,ONECUT3,FOXC2,MAZ,ZNF148
SNCA	MAZ,ZNF384,FOXE1,HSF1,HSF2,HOXB13,HOXA10,MEF2A,MEF2B,ZNF652,KLF2,KLF6,CDX2,RUNX1
BCL2L1	RFX5,RFX3,RFX7,ZFP57,ZBTB33,ZBTB14,CTCF,CTCFL,RELB,MAZ,PBX3,PKNOX1,POU3F1
RUNX2	ARGFX,HOXB3,PHOX2B,EGR4,ZNF384,SPIC
SMC3	ZNF460,DPRX,ZNF384,ZNF135,GLIS3,SOX15,SOX3
CD68	E2F6,ZNF384,SPIC,PHOX2A,PROP1,ELF1,ETV1,ETV4,IRF1,SPIB,SPI1
EEF2	TBP,NRF1,ZBTB7B,ZBTB7C,RFX7,SOX14,MEF2A,ARGFX,MNX1,SPI1,ZNF148,KLF3,ZNF460
MCM2	ZNF460,MASNTD3,HIF,ELF3,GABPA,EHF,MTF1,PPARG

**Table 4:** TF–miRNA coregulatory interaction.

Identified genes	Transcription factors or miRNA
HMOX1	ZNF460, HNF1B, TBP, ZNF135, HSF1,EBF1,EBF3,EBF2,NR2F1,MITF,USF1,NR1D1,HNF1A,HNF1B,NFIA HSF2,HSF4,hsa-miR-217-5p,hsa-miR-642a-5p,hsa-miR-2276-5p,hsa-miR-4717-5p,hsa-miR-4801, hsa-miR-5003-5p,hsa-miR-6858-3p,hsa-miR-6892-3p
MYC	PBX3,PKNOX1,FOXD3,PREB1,CTCF,KLF4,KLF15,TBX19,E2F6,MEF2A,ZNF263,PLAG1,TCF7,hsa-miR-34b-5p, hsa-miR-5000-3p,hsa-miR-1294,hsa-miR-1827,hsa-miR-548au-3p,hsa-miR-9986
FOS	ZNF384, MEF2C,RUNX1, PAX5,ONECUT1,ONECUT3,FOXC2,MAZ,ZNF148,hsa-miR-338-3p,hsa-miR-501-3p, hsa-miR-3065-3p,hsa-miR-9500,hsa-miR-10523-5p
SNCA	MAZ,ZNF384,FOXE1,HSF1,HSF2,HOXB13,HOXA10,MEF2A,MEF2B,ZNF652,KLF2,KLF6,CDX2,RUNX1,hsa-miR-520b-5p, hsa-miR-519a-2-5p,hsa-miR-8077,hsa-let-7a-2-3p,hsa-miR-196a-3p,hsa-let-7g-3p,hsa-miR-127-5p, hsa-miR-331-5p,hsa-miR-3124-3p,hsa-miR-3164,hsa-miR-6800-5p,hsa-miR-6890-3p,hsa-miR-12128, hsa-miR-485-5p,hsa-miR-3198,hsa-miR-6831-5p,hsa-miR-6833-3p,hsa-miR-486-3p,hsa-miR-514b-5p, hsa-miR-5680,hsa-miR-1229-3p,hsa-miR-6080,hsa-miR-6511b-3p,hsa-miR-6757-3p,hsa-miR-6852-3p, hsa-miR-3909
BCL2L1	RFX5,RFX3,RFX7,ZFP57,ZBTB33,ZBTB14,CTCF,CTCFL,RELB,MAZ,PBX3,PKNOX1,POU3F1,hsa-let-7a-5p, hsa-let-7e-5p,hsa-let-7c-5p,hsa-let-7b-5p,hsa-miR-491-5p
RUNX2	ARGFX,HOXB3,PHOX2B,EGR4,ZNF384,SPIC,hsa-miR-320b,hsa-miR-205-5p,hsa-miR-130a-3p,hsa-miR-148b-3p hsa-miR-320d,hsa-miR-211-5p,hsa-miR-454-3p,hsa-miR-320c,hsa-miR-122-5p,hsa-miR-520c-3p, hsa-miR-372-3p,hsa-miR-373-3p,hsa-miR-302a-3p,hsa-miR-302c-3p,hsa-miR-30a-5p,hsa-miR-148a-3p, hsa-miR-204-5p,hsa-miR-221-3p,hsa-miR-222-3p
SMC3	ZNF460,DPRX,ZNF384,ZNF135,GLIS3,SOX15,SOX3,hsa-miR-939-3p,hsa-miR-6852-5p,hsa-miR-6806-3p, hsa-miR-6783-3p,hsa-miR-588,hsa-miR-582-5p,hsa-miR-548m,hsa-miR-532-3p,hsa-miR-4797-5p, hsa-miR-4713-5p,hsa-miR-4496,hsa-miR-4435,hsa-miR-3928-5p,hsa-miR-382-5p,hsa-miR-374b-3p, hsa-miR-3163,hsa-miR-302a-5p,hsa-miR-196b-5p,hsa-miR-150-5p,hsa-miR-1343-3p,hsa-miR-1279, hsa-miR-12124
CD68	E2F6,ZNF384,SPIC,PHOX2A,PROP1,ELF1,ETV1,ETV4,IRF1,SPIB,SPI1,hsa-miR-8073,hsa-miR-7151-3p, hsa-miR-6807-5p,hsa-miR-6778-3p,hsa-miR-6742-3p,hsa-miR-6739-3p,hsa-miR-6506-5p,hsa-miR-619-5p, hsa-miR-5681a,hsa-miR-5196-5p,hsa-miR-5089-5p,hsa-miR-4747-5p,hsa-miR-4677-5p,hsa-miR-4533, hsa-miR-4524a-3p,hsa-miR-1286
EEF2	TBP,NRF1,ZBTB7B,ZBTB7C,RFX7,SOX14,MEF2A,ARGFX,MNX1,SPI1,ZNF148,KLF3,ZNF460,hsa-miR-584-5p, hsa-miR-5093,hsa-miR-3907,hsa-miR-3131,hsa-miR-2276-5p,hsa-miR-2114-5p,hsa-miR-183-5p, hsa-miR-1207-3p
MCM2	ZNF460,MASNTD3,HIF,ELF3,GABPA,EHF,MTF1,PPARG,hsa-miR-6835-3p,hsa-miR-6824-3p,hsa-miR-6814-5p, hsa-miR-6134,hsa-miR-500a-5p,hsa-miR-4722-5p,hsa-miR-4690-5p,hsa-miR-4481,hsa-miR-3190-5p, hsa-miR-3151-3p

We further built a compound-target network to explore potential therapeutic candidates (**Figure 4C**). Genes *FOS,AHR*,and *RUNX2* emerged as key drug-disease-gene nodes and showed strong connections to the natural-product compound MOL000098.

### Tumor-microenvironment scores associate with AML patient survival

To evaluate whether the tumor microenvironment correlates with clinical outcomes in AML, we calculated immune, stromal, and ESTIMATE scores for TCGA-AML patients and stratified patients into high-and low-score subgroups for survival comparison.

Kaplan-Meier survival analyses revealed that higher ESTIMATE scores (**Figure 5A**, P = 0.011) and higher immune scores (**Figure 5B**, P = 0.021) were significantly associated with poorer overall survival. In contrast, stromal score was not predictive of overall survival (**Figure 5C**, P = 0.690).These findings indicate that immune-related features within the AML microenvironment carry prognostic relevance.

**Figure 5:**
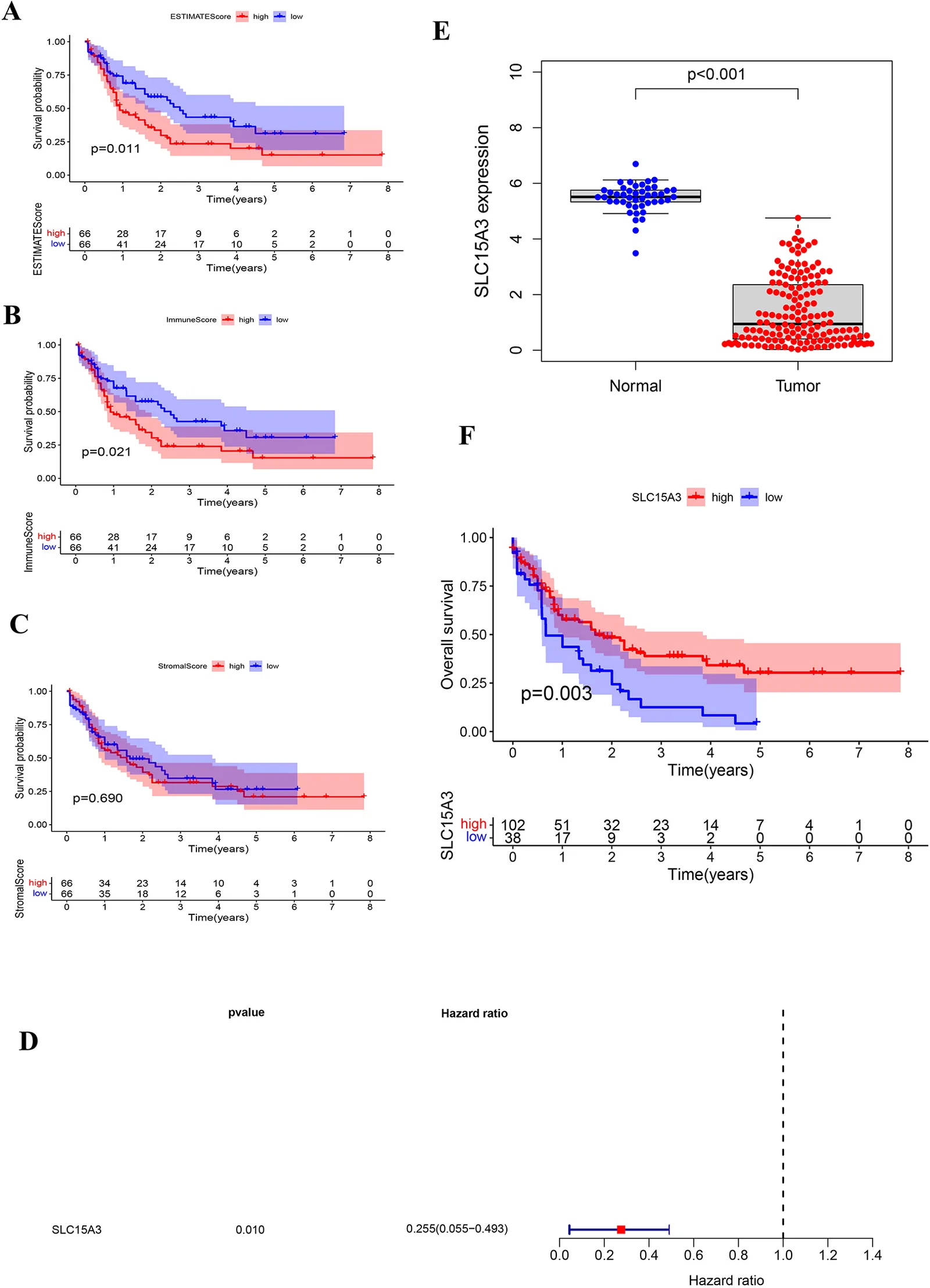
Prognostic and immunological features of *SLC15A3* in AML. All analyses were conducted on the integrated TCGA-GTEx AML dataset using R software. Tumor microenvironment indices including ESTIMATE score, immune score and stromal score were computed with the ESTIMATE algorithm. Kaplan-Meier survival analysis coupled with the log-rank test was applied for survival comparison between stratified subgroups. Univariate Cox regression was performed to evaluate the prognostic significance of *SLC15A3*. Two-tailed statistical tests were adopted, and P < 0.05 was defined as the significance threshold. **(A–C)** Kaplan-Meier overall-survival curves for AML patients stratified by ESTIMATE score, immune score, and stromal score, respectively. **(D)** Forest plot of univariate Cox regression showing the hazard ratio and P-value for *SLC15A3*. **(E)** Expression distribution of *SLC15A3* in AML tumor samples versus normal control samples. **(F)** Kaplan-Meier survival analysis comparing overall survival between *SLC15A3*-high and *SLC15A3*-low AML subgroups.

### Identification of *SLC15A3* as a candidate prognostic gene in AML

We next performed univariate Cox regression on shared DEGs to screen genes associated with AML patient survival.*SLC15A3* was identified as a favorable prognostic factor (HR = 0.255;95 %CI = 0.055–0.493;P = 0.010; **Figure 5D**). Since the entire confidence interval lay below HR = 1,higher *SLC15A3* expression corresponded to reduced mortality risk. Consistent with this prognostic pattern,*SLC15A3* was significantly down-regulated in AML tumor tissue compared with normal bone-marrow samples (**Figure 5E**), and patients with higher *SLC15A3* expression exhibited longer overall survival (**Figure 5F**, P = 0.003).

Given the prognostic value of *SLC15A3*,we analyzed its correlation with tumor-infiltrating immune-cell fractions.*SLC15A3* expression showed significant positive correlations with resting dendritic cells,M1 macrophages,M2 macrophages, monocytes, neutrophils, and resting CD4 memory T-cells. Conversely, significant negative correlations were observed for naive B-cells, eosinophils, resting and activated mast cells, activated NK-cells, and activated CD4 memory T-cells (**Figures 6A–L**). These data link *SLC15A3* expression to diverse immune-cell infiltration patterns in AML.

**Figure 6:**
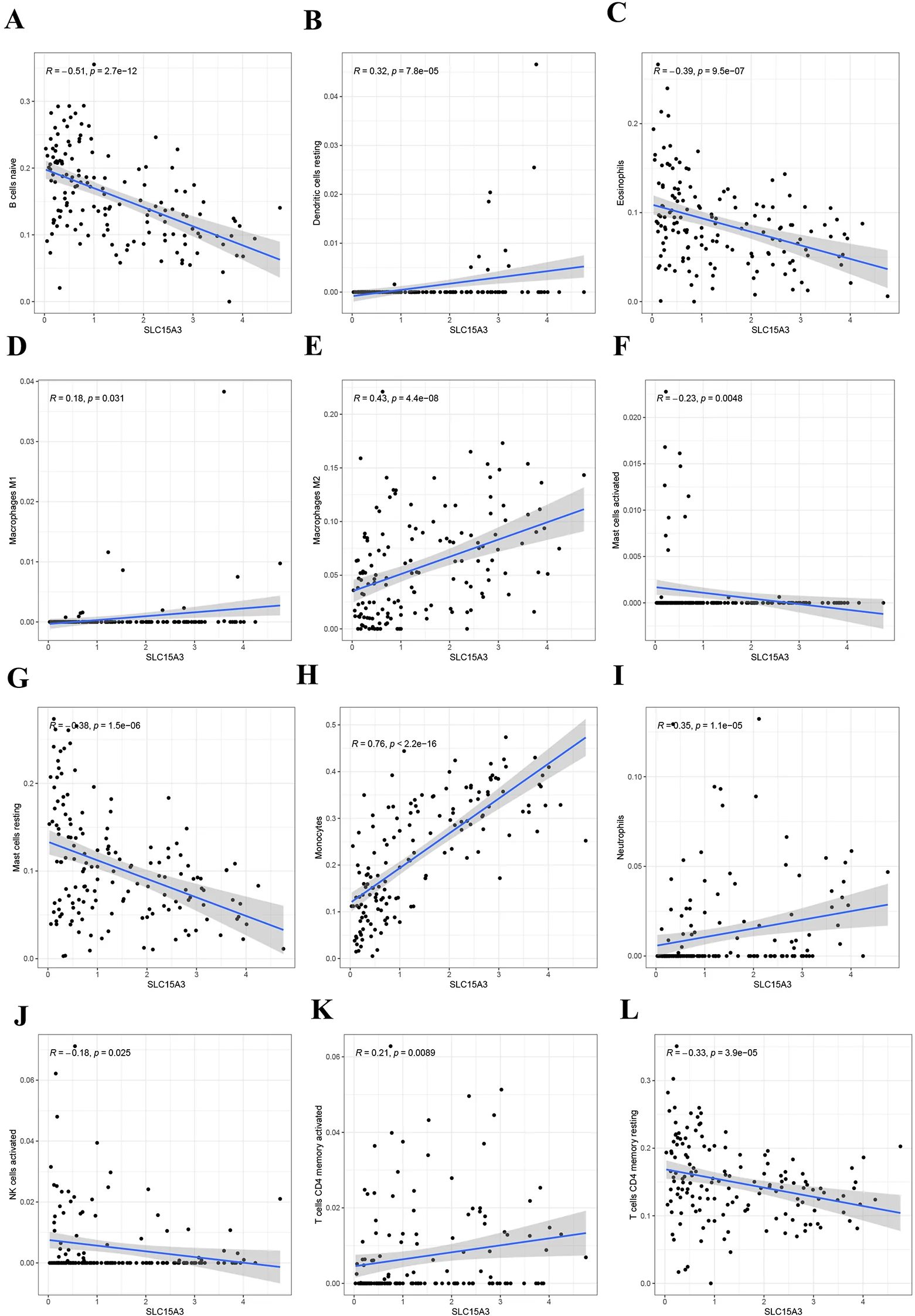
Correlation between *SLC15A3* expression and tumor-infiltrating immune-cell subsets in AML. All analyses were carried out on the integrated TCGA-GTEx AML dataset in R. The CIBERSORT algorithm was utilized to estimate the relative infiltration abundance of 22 immune-cell subtypes within AML samples. Spearman’s correlation test was performed to evaluate associations between *SLC15A3* expression and immune-cell infiltration. Two-tailed tests were applied, with P < 0.05 set as the threshold for statistical significance. **(A–L)** Scatter plots with fitted regression lines illustrating Spearman correlations between *SLC15A3* expression and infiltration levels of distinct immune-cell populations: naive B cells **(A)**, resting dendritic cells **(B)**, eosinophils **(C)**, M1 macrophages **(D)**, M2 macrophages **(E)**, activated mast cells **(F)**, resting mast cells **(G)**, monocytes **(H)**, neutrophils **(I)**, activated NK cells **(J)**, activated CD4 memory T cells **(K)**, and resting CD4 memory T cells **(L)**. The correlation coefficient (R) and corresponding P-value are labelled in each panel.

### Association between *SLC15A3* and m^6^A RNA-modification patterns

Because m^6^A epitranscriptomic modification shapes both cancer and inflammatory immune responses, we classified AML samples into three m^6^A-based molecular clusters (A,B,C) according to the expression of 23 m^6^A regulatory genes (**Figure 7B**). We extracted 164 intersecting genes whose expression differed across the three clusters (**Figure 7A**; **Table 5**).

**Figure 7:**
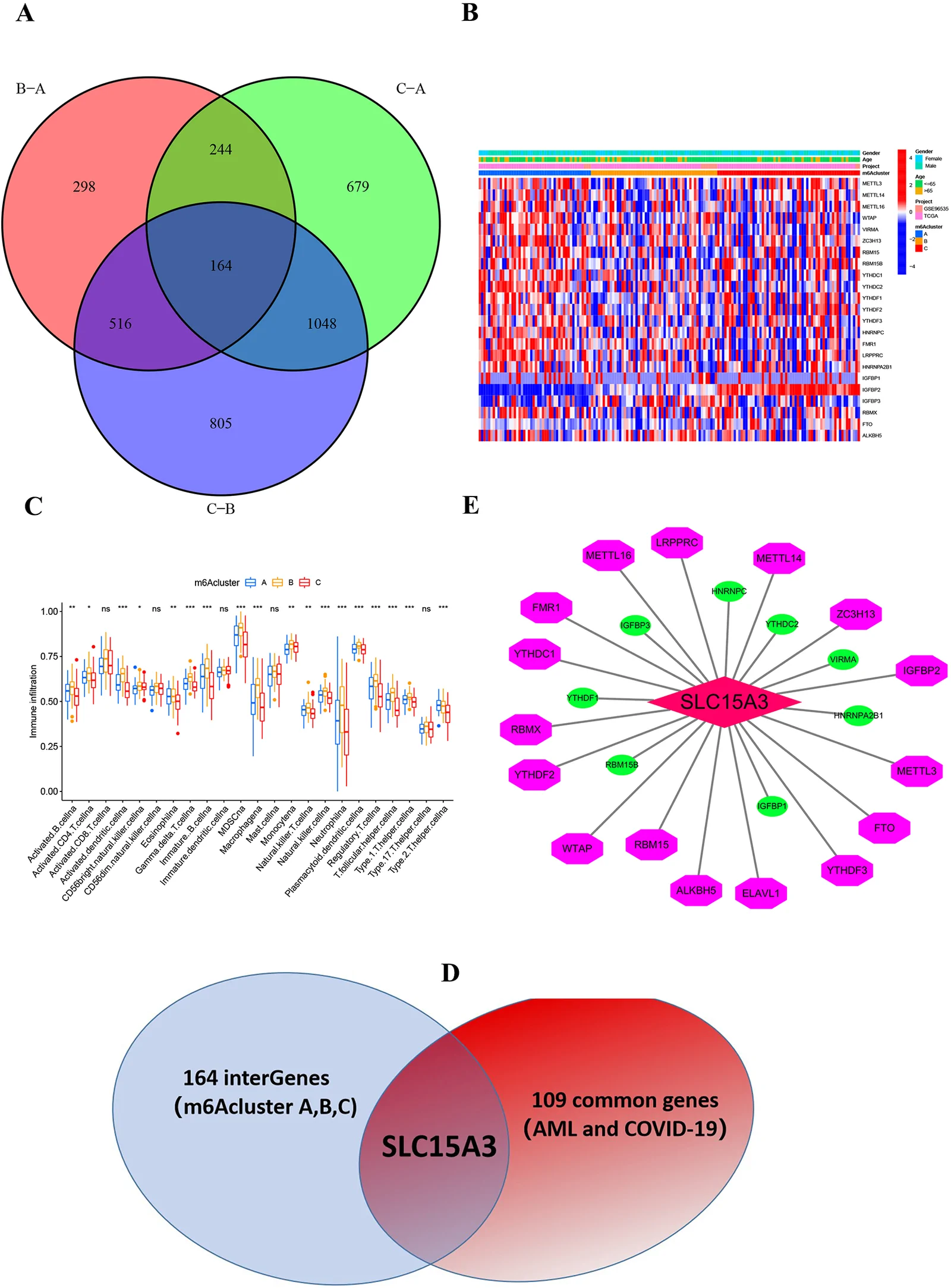
m^6^A-modification-based clustering and identification of core biomarker *SLC15A3* in AML. All analyses were implemented on the integrated TCGA-GTEx AML dataset using R. Consensus clustering was performed to stratify AML samples into three subtypes **(A–C)** based on the expression profiles of m^6^A regulatory genes. Immune-cell infiltration abundance was estimated by the CIBERSORT algorithm, and inter-group differences were assessed using ANOVA. Spearman’s correlation analysis was used to explore relationships between gene expression and m^6^A regulator levels. Two-tailed statistical tests were applied, with P < 0.05 defined as the significance threshold. **(A)** Venn diagram showing intersecting InterGenes derived from three m^6^A modification clusters **(A–C)**. **(B)** Heatmap illustrating differential expression patterns of 23 m^6^A regulatory genes across the three m^6^A clusters; red denotes high expression and blue denotes low expression. **(C)** Box-plot comparison of tumor-infiltrating immune-cell abundances among the three m^6^A modification subtypes. **(D)** Venn diagram identifying *SLC15A3* as the sole overlapping gene between 164 m^6^A-related InterGenes and 109 shared COVID-19-AML DEGs. **(E)** Network visualization of Spearman correlations between *SLC15A3* expression and the 23 m^6^A regulatory genes. * P < 0.05; ** P < 0.01; *** P < 0.001; ns = not significant (P ≥ 0.05).

**Table 5:** 164 InterGenes in cluster A, B and C.

164 InterGenes in cluster A, B and C
IGFBP2 CASC15 RN7SKP240 CSTA IGHV3-33 GNAI1 CFD AZU1 CLU NPR3 LYZ PROM1 PLPPR3 PRTN3 AC002454.1 CD4 HPGDS AL133371.2 RNASE6 ABCC3 TBC1D9 ELANE AP001257.1 MYCL LHFPL6 CDC42EP1 LGALS2 CCR2 TRPC2 MEF2C ARHGEF10L SAMHD1 CXCR2P1 AC116366.2 AC098613.1 OASL PTGDS CYP1B1 VDR TLDC2 HTR1F CLEC4A SIGLEC1 LIN7A FAM198B CCL5 TLR5 TLR7 KCNK5 SLC15A3 IL1RN CYP27A1 CTSG MPEG1 PTAFR MLLT3 IGSF6 PF4 SCARA3 CD36 MIR4319 PHGDH NAALADL1 AL161785.1 ST14 SCIMP PTGFRN MARCKS DOK2 FCN1 SIGLEC7 LY96 KLHL13 FAM201A SIRPD CCR5 BLNK SLC37A2 MS4A6A NCF1C ANO7 RNU6ATAC6P CD300C AC125603.2 JAML NCF1B RBM47 CLEC9A TSPAN7 C19orf38 ADA2 ANXA5 TLR8 SMIM24 LILRB4 CD34 CCR1 AC090673.1 NCF1 CD300LB MGST1 LINC01374 CX3CR1 RAB27B IRF8 GIMAP4 CEBPE VNN3 SIRPB1 FGL2 ADGRE3 CST7 MTMR11 NRIP1 DSC2 TYMP GBP4 MMP2 ADAMTS10 AC245128.3 FGR MS4A3 PPBP AC125603.1 PLD4 LILRB1 NCF2 ATP10A C1QA SLC7A7 LY86 MARCO HK3 LRP1 SERPINB2 ASGR2 MS4A4A CHST15 MYOF S100A9 LRRC25 AC244502.1 FCAR KCTD12 NLRP12 IRX3 RAB31 PLBD1 MEFV LAMB2 KRT18 C1QC S100A8 LAMP5 MAFB CYBB COL4A5 LILRA5 VCAN DEFB1 SNORD10 SNORA63 CD14 S100A12

GSVA pathway analysis revealed distinct pathway enrichment for each cluster. Cluster-A samples were enriched in RNA degradation, spliceosome and chronic myeloid-leukemia-related signatures. Cluster-B samples were enriched in basal-cell carcinoma, ECM-receptor interaction, cytokine-cytokine-receptor interaction, complement-coagulation cascades and systemic lupus erythematosus-associated pathways. Cluster-C samples were characterized by enrichment of the renin-angiotensin-system-related pathway (**Supplementary Figures S1**–**S3**). Immune-cell infiltration abundances also differed significantly across the three m^6^A clusters (**Figure 7C**), supporting that m^6^A modification is linked to immune microenvironment heterogeneity in AML.

We intersected these 164 m^6^A-associated genes with the 109 shared DEGs of AML-COVID-19.Only one gene overlapped:*SLC15A3* (**Figure 7D**). Further correlation analysis demonstrated that *SLC15A3* expression was correlated with multiple m^6^A regulatory factors (**Figure 7E**). This finding indicates that *SLC15A3* is associated with both AML and COVID-19 and correlates with the expression of multiple m^6^A regulators.

### WGCNA-derived identification of AML-related candidate genes

To investigate genes associated with AML, we performed differential analysis on the integrated and batch-corrected data from datasets GSE2191,GSE9476 and GSE96535 (**Supplementary Figure S4**). The global transcriptomic landscape is overviewed in the heatmap (**Figure 8A**), and corresponding volcano plot (**Figure 8B**) presents statistically significant DEGs identified by limma analysis. We then conducted gene co-expression analysis using WGCNA to identify modules and genes related to AML.WGCNA co-expression analysis identified the MEgreen module as most strongly correlated with AML status (r = −0.83, P = 5e-35),from which 297 module-member genes were extracted. Intersecting DEGs and MEgreen-module genes produced 295 “InterGenes”, representing AML-relevant co-expressed differentially-expressed candidates (**Figures 8C, D**). GO-KEGG enrichment of these 295 InterGenes highlighted sodium-ion transmembrane transport, muscle contraction, circulatory-system vascular processes, gastric-cancer-associated pathways and hormone-signaling pathways (**Figures 8E–H**).

**Figure 8:**
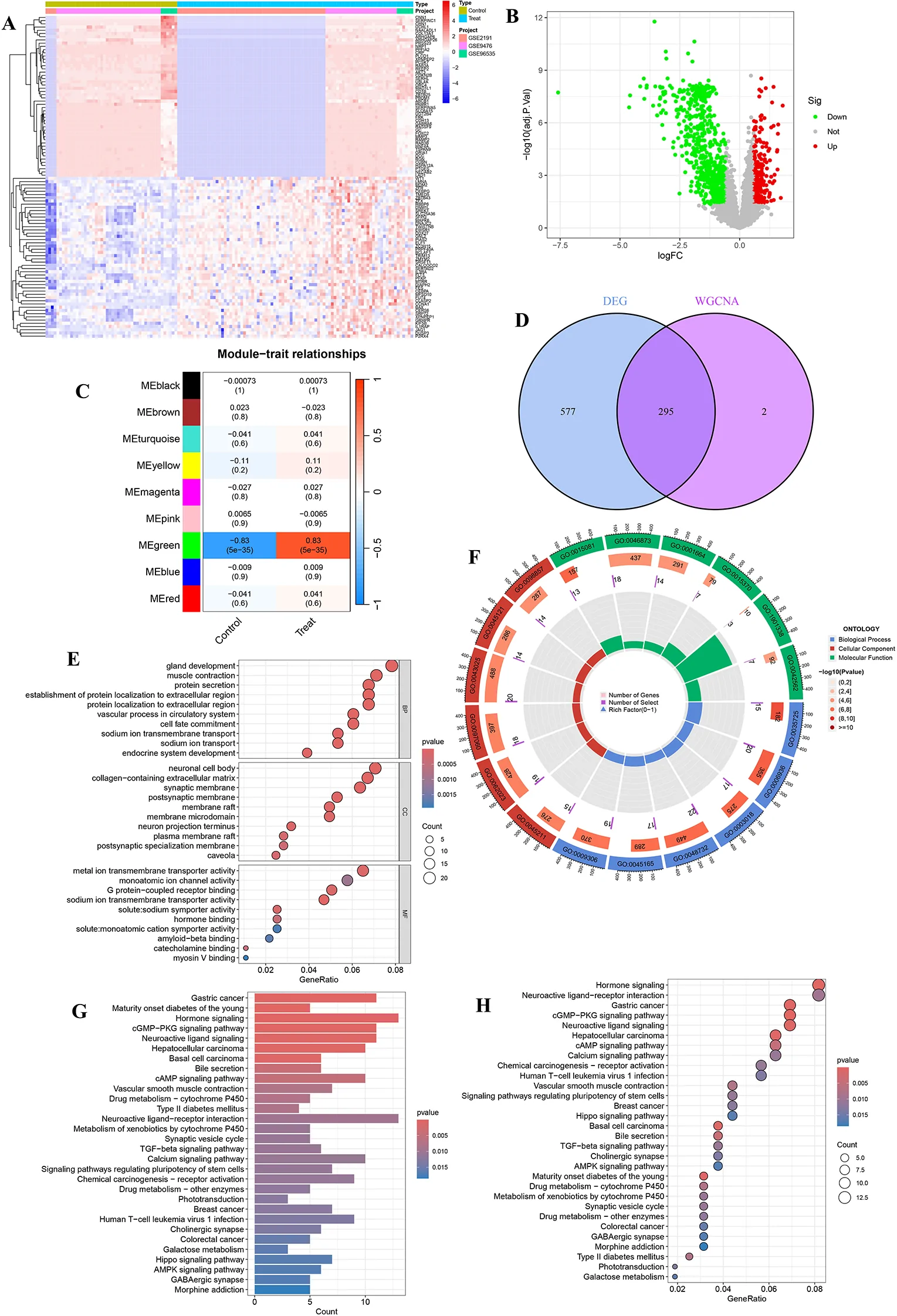
Differential expression analysis, weighted gene co-expression network analysis (WGCNA), and functional enrichment based on integrated AML transcriptomic datasets. Three AML transcriptomic datasets (GSE2191, GSE9476, GSE96535) were integrated for downstream analyses in Differentially expressed genes (DEGs) were screened using the limma package with thresholds of |log_2_FC| ≥ 1 and P < 0.05,following empirical Bayesian moderated t-tests and Benjamini-Hochberg FDR correction. WGCNA was applied to construct co-expression networks and detect AML-associated gene modules. The intersection of DEGs and WGCNA core-module genes yielded 295 InterGenes, which were subjected to GO and KEGG enrichment analyses via the clusterProfiler package. Two-tailed statistical tests were applied, and P < 0.05 was defined as the significance threshold. **(A)** Hierarchical-clustering heatmap of global transcriptomic profiles across merged datasets. Gene expression was normalized by row-wise z-score. **(B)** Volcano plot visualizing DEG signatures in the integrated AML cohort. **(C)** WGCNA module-trait correlation heatmap showing correlation coefficients and corresponding P-values between co-expression modules and AML disease status. **(D)** Venn diagram demonstrating the overlap between DEGs and WGCNA core-module genes, generating 295 InterGenes. **(E, F)** Dot-plot and Circos plot illustrating top enriched Gene Ontology (GO) terms for the 295 InterGenes. **(G, H)** Bar plot and dot-plot displaying significantly enriched Kyoto Encyclopedia of Genes and Genomes (KEGG) pathways for the 295 InterGenes. AML indicates acute myeloid leukemia patient samples; CON indicates normal bone-marrow control samples. This heatmap presents row-wise z-score-normalized relative expression. Given the large number of gene rows included, the plot exhibits a dense visual layout. Biological interpretations rely on downstream statistical analyses and functional enrichment results, rather than direct visual interpretation of heatmap colors.

### Machine-learning screening and immune-microenvironment analysis of candidate genes

To construct AML diagnostic machine-learning models, we used the integrated transcriptomic dataset, with GSE33223 and GSE57194 serving as external validation cohorts. Among tested algorithms, the plsRglm model yielded the highest AUC and optimal performance (**Figure 9A**). ROC analyses for training and validation datasets achieved AUC values above 0.7,indicating reliable capacity to distinguish AML patients from control subjects (**Figures 9B–D**).

**Figure 9:**
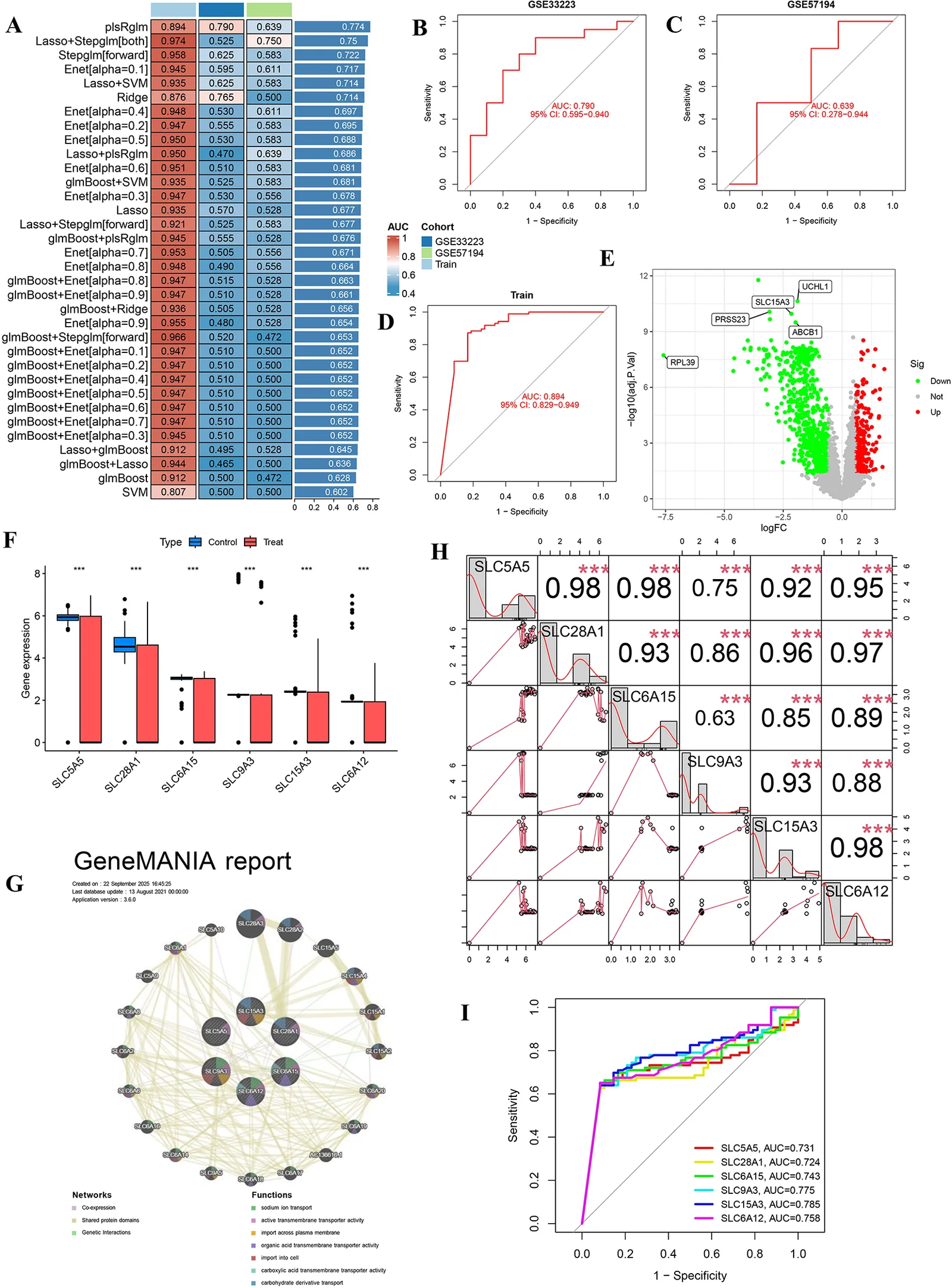
Machine-learning screening and validation of AML diagnostic models and candidate genes. Two independent AML transcriptomic datasets (GSE33223,GSE57194) were used for machine-learning model construction and external validation in R. Multiple algorithms were compared to select the optimal diagnostic model, whose performance was assessed by receiver-operating-characteristic (ROC) curves and area-under-curve (AUC) values. Candidate model-gene differential expression was analyzed using the limma package with Benjamini-Hochberg FDR correction (|log_2_FC| ≥ 1, P < 0.05). Protein-protein interaction and pairwise gene-correlation analyses were conducted to characterize inter-relationships among six core model genes. Two-tailed tests were adopted, and P < 0.05 represented the significance cutoff. **(A)** Performance summary table comparing prediction metrics across multiple machine-learning algorithms. **(B)** ROC curve showing model diagnostic performance in the GSE33223 validation cohort. **(C)** ROC curve showing model diagnostic performance in the GSE57194 validation cohort. **(D)** ROC curve illustrating model performance within the training cohort. **(E)** Volcano plot depicting differential-expression signatures for key model-related genes. **(F)** Box plots displaying expression levels of six core model genes between control and AML groups. **(G)** Gene-interaction network of six core model genes generated via GeneMANIA. **(H)** Pairwise expression-correlation matrix for the six core model genes. **(I)** Individual-gene ROC curves evaluating the diagnostic capability of each of the six core genes. *** P < 0.001.

Combining model feature importance and differential-expression outputs, six solute-carrier-family genes (*SLC5A5,SLC28A1,SLC6A15, SLC9A3, SLC15A3, SLC6A12*) with strong predictive ability were selected (**Figures 9E, F**). Each gene obtained an AUC > 0.7 (**Figure 9I**). Correlation analysis revealed notable co-expression relationships among these six candidates, and the PPI-derived heatmap displayed their functional interactions (**Figures 9G, H**).

We next characterized immune-cell-infiltration differences between AML and control groups (**Figures 10A, B**). Control samples were enriched for naive B cells, naive CD4^+^ T cells and neutrophils, whereas AML samples showed higher signals of CD8^+^ T cells, monocytes and resting mast cells. Pair-wise correlation analysis uncovered complex crosstalk among infiltrating immune subsets (**Figure 10C**). For instance,CD8^+^ T-cell abundance was negatively correlated with monocyte levels (r = −0.55);resting mast cells also correlated negatively with monocytes (r = −0.41). Positive correlations existed between CD8^+^ T cells and naive B cells (r = 0.39),as well as CD8^+^ T cells and resting mast cells (r = 0.39).Such cellular interactions may contribute to AML immunopathogenesis.

**Figure 10:**
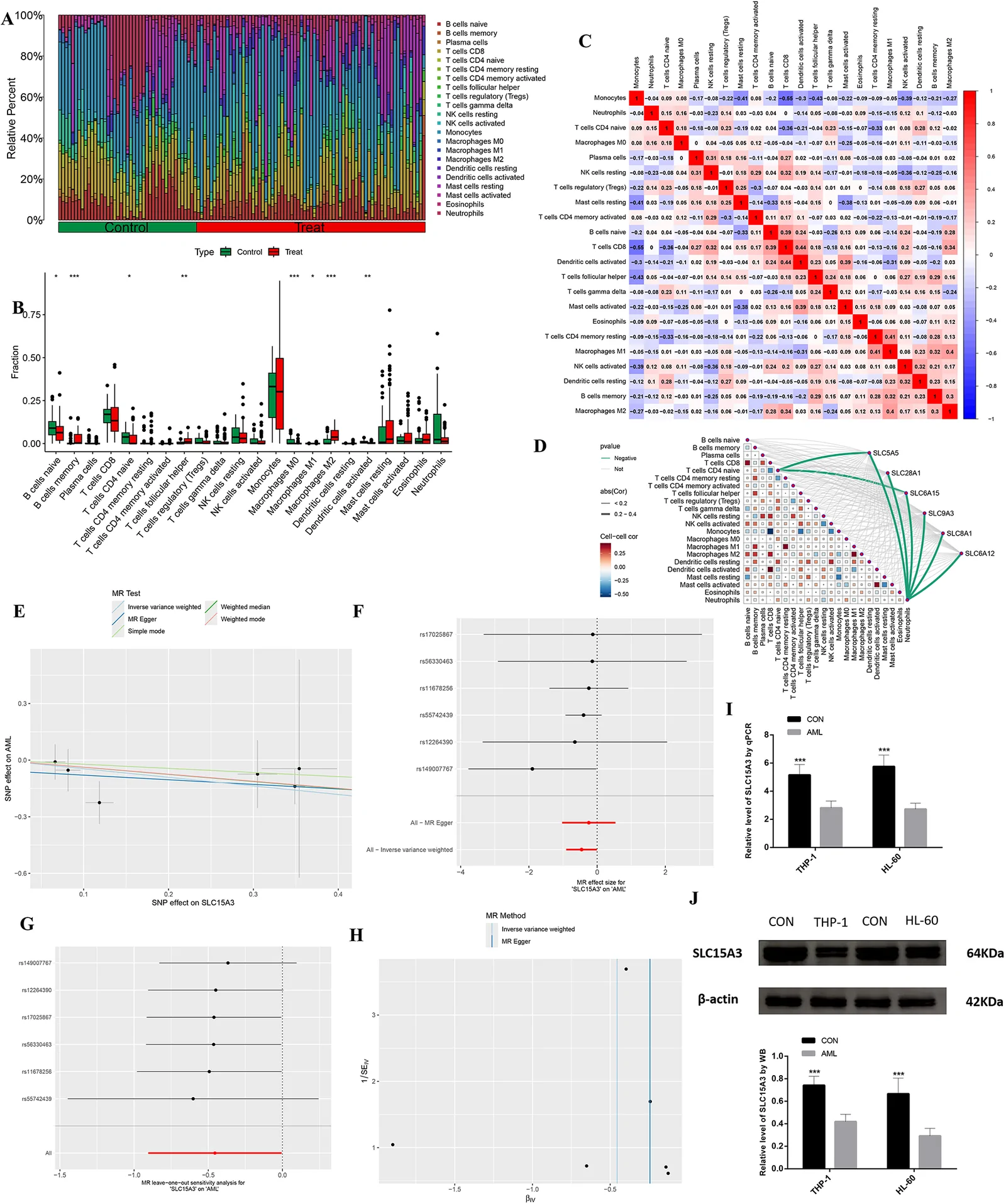
Immune-cell landscape characterization, Mendelian randomization causal inference, and experimental validation of prognostic biomarker SLC15A3 in AML. Bioinformatic analyses were performed on public AML datasets using R.CIBERSORT was applied to quantify relative fractions of 22 tumor-infiltrating immune-cell subsets. Spearman correlation was used to evaluate associations between immune-cell populations and core model genes. Mendelian randomization (MR) was utilized to assess the causal effect of *SLC15A3* on AML risk; leave-one-out sensitivity analysis and funnel plots were conducted to verify result stability and detect potential bias. RT-qPCR and western blotting were performed in HS-5 (normal bone-marrow stromal control),THP-1 and HL-60 AML cell lines to validate *SLC15A3* mRNA and protein abundance. Two-sided tests were applied, and P < 0.05 was defined as the significance threshold. **(A)** Stacked bar chart illustrating relative proportions of 22 immune-cell subsets across control and AML samples. **(B)** Box-plot showing the distribution of immune-cell infiltration fractions between control and AML groups. **(C)** Correlation-matrix heatmap presenting pairwise correlations among distinct immune-cell subtypes. **(D)** Correlation network depicting inter-relationships between six core model genes and tumor-infiltrating immune cells. **(E)** MR scatter plot showing the causal estimate of *SLC15A3* on AML. **(F)** Forest plot summarizing MR effect-size estimates for *SLC15A3*. **(G)** Leave-one-out sensitivity analysis assessing the robustness of MR outcomes for *SLC15A3*. **(H)** Funnel plot for the detection of potential bias in MR analysis. **(I)** RT-qPCR quantification of relative *SLC15A3* mRNA levels in HS-5,THP-1 and HL-60 cell lines. **(J)** Western-blot detection of *SLC15A3* protein expression in HS-5,THP-1 and HL-60 cell lines. * P < 0.05; ** P < 0.01; *** P < 0.001.

We further assessed correlations between the six SLC-family candidates and immune-cell infiltration (**Figure 10D**). All six genes exhibited significant negative correlations with neutrophil infiltration. Detailed gene-immune scatter plots are provided in **Supplementary Figure S5**. Based on multi-omics screening outputs, *SLC15A3* was prioritized for subsequent causal inference and wet-lab validation.

### Mendelian randomization and cellular validation of *SLC15A3*

Mendelian randomization (MR) was performed to evaluate the causal relationship between genetically-predicted *SLC15A3* expression and AML risk.MR analyses supported a protective effect specific to *SLC15A3*.The scatter plot summarizing five MR methods showed that higher genetically-predicted *SLC15A3* expression corresponded to lower AML risk (**Figure 10E**). Forest plots for individual SNP instruments consistently showed negative effect estimates, further confirming that *SLC15A3* acts as a protective factor for AML (**Figure 10F**). Leave-one-out sensitivity analysis confirmed result stability, as removal of any single SNP did not change overall MR outcomes (**Figure 10G**). Funnel-plot analysis showed no obvious heterogeneity across genetic instruments (**Figure 10H**). Collectively, MR evidence suggests that elevated *SLC15A3* expression may reduce AML risk.

We further conducted cellular experiments to validate *SLC15A3* expression patterns’-qPCR and Western blot results demonstrated significant down-regulation of *SLC15A3* in THP-1 and HL-60 AML cell lines compared with normal HS-5 bone-marrow stromal cells (P < 0.01;**Figures 10I, J**). These experimental findings are consistent with our bioinformatic predictions and provide cellular-level evidence supporting the biological role of *SLC15A3* in AML.

## Discussion

COVID-19 caused by SARS-CoV-2 constitutes a major global public-health threat. Cancer patients suffer from disease-intrinsic immune disturbance and therapy-related immunosuppression, rendering them more vulnerable to SARS-CoV-2 infection and associated mortality compared with the general population (31–33). Acute myeloid leukemia (AML) is a highly heterogeneous hematologic malignancy driven by clonal expansion and impaired differentiation of immature myeloid cells. The COVID-19 pandemic has further complicated clinical management for AML patients (6, 34). In this study, we tried to find the correlation between AML and COVID-19,identify important prognostic genes and Chinese medicine components, predict the TF and miRNA of prognostic genes, and identify important genes involved in cellular immune infiltration, providing new ideas for clinical diagnosis and treatment.

We identified 109 shared differentially expressed genes (DEGs) between AML and COVID-19.Functional enrichment highlighted neutrophil-dominated immune-inflammatory responses as core shared biological features, consistent with the well-recognized roles of aberrant neutrophil activation and degranulation in both leukemic immune evasion and the hyperinflammatory phase of COVID-19 (35). PPI network analysis prioritized ten hub genes. Among them, *HMOX1*, regulated by 17 transcription factors, encodes heme oxygenase-1 (*HO-1*)*.HO-1*,as an Nrf2-dependent stress-responsive enzyme, mediates heme catabolism to generate biliverdin and carbon monoxide, thereby conferring anti-oxidative and anti-inflammatory cytoprotection; this pathway has dual implications in tumor progression and virus-associated tissue injury, and may represent a therapeutic node relevant to both AML and COVID-19 (36–38). The high-connectivity gene *SNCA*, well established in Parkinson’s disease pathogenesis, can promote neuro-inflammation through aberrant alpha-synuclein aggregation, which may contribute to the neurological sequelae increasingly reported after SARS-CoV-2 infection (39, 40). It can also form plaques due to abnormal aggregation of itself or the brain environment, affecting the normal function of neurons and brain tissue, this may be related to the sequelae of COVID-19 (41, 42). Network-pharmacology analysis identified *FOS* and *RUNX2* as primary targets of the bioactive compound MOL000098,illustrating how traditional Chinese medicine may exert multi-target regulatory effects at the interface of these diseases (43–45).

Integrative analyses of the tumor microenvironment, immune-cell infiltration and m^6^A modification nominated *SLC15A3* as a promising prognostic marker for AML (46), linking epitranscriptomic regulation to the disease-shared immune landscape. Though we characterized tumor-infiltrating immune-cell composition linked to *SLC15A3*,immune-cell fractions were not multivariate Cox covariates. Complete metadata for bone-marrow blast percentage and flow-based myeloid-differentiation immunophenotype are unavailable across public cohorts, precluding confounder adjustment.Thus,*SLC15A3*’s prognostic signal may be biased by blast burden or differentiation status. Future primary-sample studies with full clinical-flow data are needed for validation.

Mechanistically, *SLC15A3* encodes a lysosomal proton-coupled histidine/peptide transporter (PHT2) implicated in peptide translocation and the regulation of innate immune signaling and MHC-I-related antigen presentation (47). Its down-regulation in AML may perturb lysosomal antigen-processing and inflammatory signaling, potentially shaping both tumor-intrinsic behavior and antiviral host responses. Our observation that *SLC15A3* correlates with multiple immune-cell populations and with m^6^A regulatory factors further supports its relevance as an immune-and epitranscriptome-linked prognostic gene, although the precise downstream mechanisms require experimental dissection. We note our survival analyses were limited to univariate Cox regression. Our integrated public cohorts lack uniformly complete metadata for established AML risk factors (age, cytogenetic risk, *NPM1,FLT3-ITD,CEBPA,TP53*,therapy), precluding fully-adjusted multivariate Cox regression analyses to test the independent prognostic value of SLC15A3 (48). Its observed survival correlation may be confounded by these clinical-molecular variables. Future well-annotated primary AML cohorts are required for multivariate Cox regression to validate whether SLC15A3 retains statistically independent prognostic significance.

Machine Learning (ML) has emerged as a powerful tool in biomedical research, leveraging high-performance computing to elucidate disease patterns and mechanisms through the analysis of large-scale datasets (49). Combining differential-expression results and WGCNA across GSE2191, GSE9476 and GSE96535,295 InterGenes were obtained. The plsRglm model screened six SLC-family candidate genes, all negatively correlated with neutrophil infiltration, reinforcing the link between the solute-carrier gene panel and innate immune features.MR indicated a potential protective effect of genetically predicted *SLC15A3* against AML, providing orthogonal genetic evidence supporting its prognostic relevance. We note limitations in our two-sample MR analysis-statistics for genetic instruments were not reported, so weak-instrument bias cannot be fully excluded. Formal horizontal-pleiotropy tests were absent, and colocalization analysis was not conducted. Thus, our MR provides suggestive rather than definitive causal evidence requiring further validation.

It should be noted that our study lacks direct experimental evidence for *SLC15A3*-mediated SARS-CoV-2-related inflammatory-AML crosstalk. RT-qPCR and WB only confirmed its down-regulation in unstimulated AML cell lines.MR cannot verify inflammation-relevant causality, and bulk signals may reflect bone-marrow immune composition. Thus, *SLC15A3* is a bioinformatically-predicted prognostic candidate requiring further functional validation.

## Conclusion

Patients with AML complicated by COVID-19 infection face severe immune disturbance and difficult clinical management, greatly complicating routine AML treatment. Focusing on the prognostic characteristics of AML, this study explored common molecular features underlying AML and COVID-19 and identified *SLC15A3* as a potential m^6^A-related immune gene associated with tumor immune microenvironment changes and patient survival. Notably, our conclusions are primarily derived from data mining and genetic causal analysis, with only limited preliminary validation.Therefore,*SLC15A3* should be regarded as a preliminary candidate prognostic marker for AML. This work provides novel theoretical clues for further functional verification and clinical prognostic research of AML.

## Data Availability

The datasets presented in this study can be found in online repositories. The names of the repository/repositories and accession number(s) can be found below: https://www.ncbi.nlm.nih.gov/, GSE180226; GSE96535; GSE2191; GSE9476; GSE33223; GSE57194.

## Glossary

AML: Acute myeloid leukemia
BP: Biological Process
CC: Cellular Component
SARS-CoV-2: severe acute respiratory syndrome Coronavirus
DAVID: Database for Annotation,Visualization and Integrated Discovery
GSEA: Gene Set Enrichment Analysis
COVID-19: Coronavirus Disease 2019
FC: Fold change
GO: Gene Ontology
KEGG: Kyoto Encyclopedia of Genesand Genomes
PPI: Protein–protein interaction
LncRNA: Long non-coding RNA
MF: Molecular Function
EMT: Epithelial-Mesenchymal Transition
miRNA: Micro RNA
mRNA: Messenger RNA
ncRNA: Non-coding RNA
ceRNAs: competing endogenous RNAs
PBS: Phosphate buffer saline
PPI: Integration of protein-protein interaction
TF: Transcription factors
TME: tumor microenvironment
GEO: Gene Expression Omnibus
AUC: Area Under the Curve
ROC: Receiver Operating Characteristic
WGCNA: Weighted Gene Co-expression Network Analysis
MR: Mendelian randomization
ML: Machine learning
m^6^A: N^6^-methyladenosine
C-DEGs: Common differentially expressed genes
ESTIMATE: Estimation of Stromal and Immune cells in Malignant Tumor tissues using Expression data
ssGSEA: Single-sample Gene Set Enrichment Analysis
CNV: copy number variant
GTEx: Genotype-Tissue Expression
PCA: Principal Component Analysis
RT-qPCR: Quantitative real-time polymerase chain reaction
WB: Western blotting

## References

[1] Dinardo CD Erba HP Freeman SD Wei AH . Acute myeloid leukaemia. Lancet. (2023) 401:2073–86. doi: 10.1016/s0140-6736(23)00108-3 37068505

[2] Chandra DJ Alber B Saultz JN . The immune resistance signature of acute myeloid leukemia and current immunotherapy strategies. Cancers (Basel). (2024) 16. doi: 10.3390/cancers16152615 39123343 PMC11311077

[3] Pagano L Salmanton-Garćıa J Marchesi F Busca A Corradini P Hoenigl M . COVID-19 infection in adult patients with hematological malignancies: a European Hematology Association Survey (EPICOVIDEHA). J Hematol Oncol. (2021) 14:168. doi: 10.1186/s13045-021-01177-0 34649563 PMC8515781

[4] Garćıa-Súarez J De la Cruz J Cedillo Á Llamas P Duarte R Jiménez-Yuste V . Impact of hematologic malignancy and type of cancer therapy on COVID-19 severity and mortality: lessons from a large population-based registry study. J Hematol Oncol. (2020) 13:133. doi: 10.1186/s13045-020-00970-7 PMC7542567 33032660

[5] Döhner H Dinardo CD Appelbaum FR Craddock C Dombret H Ebert BL . Genetic risk classification for adults with AML receiving less-intensive therapies: the 2024 ELN recommendations. Blood. (2024) 144:2169–73. doi: 10.1182/blood.2024025409 39133932

[6] Shi M Chen L Wei Y Chen R Guo R Luo F . Systematic analysis of prognostic and immunologic characteristics associated with coronavirus disease 2019 regulators in acute myeloid leukemia. Front Genet. (2022) 13:959109. doi: 10.3389/fgene.2022.959109 36147489 PMC9485716

[7] Cai X Wang H Han Y Huang H Qian P . The essential roles of small non-coding RNAs and RNA modifications in normal and malignant hematopoiesis. Front Mol Biosci. (2023) 10:1176416. doi: 10.3389/fmolb.2023.1176416 37065445 PMC10102602

[8] Vago L Gojo I . Immune escape and immunotherapy of acute myeloid leukemia. J Clin Invest. (2020) 130:1552–64. doi: 10.1172/jci129204 32235097 PMC7108895

[9] Thoms JAI Truong P Subramanian S Knezevic K Harvey G Huang Y . Disruption of a GATA2-TAL1-ERG regulatory circuit promotes erythroid transition in healthy and leukemic stem cells. Blood. (2021) 138:1441–55. doi: 10.1182/blood.2020009707 34075404

[10] Menon A Abd-Aziz N Khalid K Poh CL Naidu R . miRNA: A promising therapeutic target in cancer. Int J Mol Sci. (2022) 23. doi: 10.3390/ijms231911502 36232799 PMC9569513

[11] Pieri M Vayianos P Nicolaidou V Felekkis K Papaneophytou C . Alterations in circulating miRNA levels after infection with SARS-CoV-2 could contribute to the development of cardiovascular diseases: What we know so far. Int J Mol Sci. (2023) 24. doi: 10.3390/ijms24032380 36768701 PMC9917196

[12] Eagle K Jiang Y Shi X Li M Obholzer NP Hu T . An oncogenic enhancer encodes selective selenium dependency in AML. Cell Stem Cell. (2022) 29:386–99.e7. doi: 10.1016/j.stem.2022.01.003 35108519 PMC8903199

[13] Anticoli S Dorrucci M Iessi E Meschi S Bordi L Amendola A . Profile of selected microRNAs as markers of sex-specific anti-S/RBD response to COVID-19 mRNA vaccine in health care workers. Int J Mol Sci. (2025) 26. doi: 10.3390/ijms26157636 40806764 PMC12346932

[14] Zhao B Wang W Zhao Y Li Y Zhang Y Yu J . Regulation of antiviral immune response by N (6)-methyladenosine of mRNA. Front Microbiol. (2021) 12:789605. doi: 10.3389/fmicb.2021.789605 34975810 PMC8716812

[15] Zhuang H Yu B Tao D Zhang X Chen X Wang H . The role of m^6^A methylation in therapy resistance in cancer. Mol Cancer. (2023) 22:91. doi: 10.1186/s12943-023-01782-2 37264402 PMC10233906

[16] Zhang X Ma Y Yu J Su R Wang X . Internal m 6 A and m 7 G RNA modifications in hematopoietic system and acute myeloid leukemia. Chin Med J (Engl). (2024) 137:1033–43. doi: 10.1097/cm9.0000000000003073 38545694 PMC11062654

[17] Horner SM Reaves JV . Recent insights into N(6)-methyladenosine during viral infection. Curr Opin Genet Dev. (2024) 87:102213. doi: 10.1016/j.gde.2024.102213 38901100 PMC11317213

[18] Hao X Fan C Yan L Li Y Wang Y Liu J . Metabolic reprogramming and immune regulation in acute myeloid leukemia. Front Immunol. (2025) 16:1655005. doi: 10.3389/fimmu.2025.1655005 41451233 PMC12727915

[19] Alhamrani SQ Ball GR El-Sherif AA Oyelere AK Ghevaert C Alzahrani SH . Machine learning for multi-omics characterization of blood cancers: A systematic review. Cells. (2025) 14. doi: 10.3390/cells14171385 40940796 PMC12427946

[20] Lovegrove CE Howles SA Furniss D Zheng J Burgess S Davey Smith G . Causal inference in health and disease: a review of the principles and applications of Mendelian randomization. J Bone Miner Res. (2024) 39:1539–52. doi: 10.1093/jbmr/zjae136 39167758 PMC11523132

[21] Eleftheriou M Russell J Tzelepis K . Epitranscriptomic advances in normal and malignant hematopoiesis. Leukemia. (2025) 39:2848–57. doi: 10.1038/s41375-025-02765-6 40973767 PMC12634440

[22] Peng DF Feng L Li HQ . Identification of novel circRNA-based ceRNA network involved in the pathogenesis of gastric cancer. Int J Genomics. (2022) 2022. doi: 10.1155/2022/5281846 35685832 PMC9174013

[23] Liu M Yu B Tian Y Liu Y Zhang Y Wang X . Regulatory function and mechanism research for m6A modification WTAP via SUCLG2-AS1- miR-17-5p-JAK1 axis in AML. BMC Cancer. (2024) 24:98. doi: 10.1186/s12885-023-11687-4 38233760 PMC10795285

[24] Choudhary S Anand R Pradhan D Gupta S Jha NK Jha SK . Transcriptomic landscaping of core genes and pathways of mild and severe psoriasis vulgaris. Int J Mol Med. (2021) 47:219–31. doi: 10.3892/ijmm.2020.4771 PMC7723513 33416099

[25] Lv Y Zhang T Cai J Liu Y Chen Y Yang Y . Bioinformatics and systems biology approach to identify the pathogenetic link of Long COVID and Myalgic Encephalomyelitis/Chronic Fatigue Syndrome. Front Immunol. (2022) 13:952987. doi: 10.3389/fimmu.2022.952987 36189286 PMC9524193

[26] Zhang JP He JF . “ Identifying functional TF-miRNA-mRNA regulatory modules with Latent Dirichlet Allocation”, in: Proceedings of the 8th International Conference on Bioinformatics and Biomedical Engineering (ICBBE), Suzhou, PEOPLES R CHINA, F Sep 20-22, 2014. Suzhou: IEEE (2014).

[27] Wang H Wu XS Chen YM . Stromal-immune score-based gene signature: A prognosis stratification tool in gastric cancer. Front Oncol. (2019) 9. doi: 10.3389/fonc.2019.01212 31781506 PMC6861210

[28] Shao R Wang H Liu W Zhang Y Ding K Li Y . Establishment of a prognostic ferroptosis-related gene profile in acute myeloid leukaemia. J Cell Mol Med. (2021) 25:10950–60. doi: 10.1111/jcmm.17013 34741393 PMC8642683

[29] Cheng F Li Q Wang J Liu Y Zhang H Li Y . HCK is a potential prognostic biomarker that correlates with immune cell infiltration in acute myeloid leukemia. Dis Markers. (2022) 2022:3199589. doi: 10.1155/2022/3199589 35280440 PMC8916870

[30] Shen S Yan J Zhang Y Chen M Li Z Chen H . N^6^-methyladenosine (m^6^A)-mediated messenger RNA signatures and the tumor immune microenvironment can predict the prognosis of hepatocellular carcinoma. Ann Transl Med. (2021) 9:59. doi: 10.21037/atm-20-7396 33553352 PMC7859781

[31] De Mello MT Silva A Guerreiro RD Barros L Paiva T Monteiro L . Sleep and COVID-19: considerations about immunity, pathophysiology, and treatment. Sleep Sci. (2020) 13:199–209. doi: 10.1016/j.slsci.2020.07.002 PMC7755266 33381288

[32] Bayrak M Cadirci K . Successful pulsed methylprednisolone and convalescent plasma treatment in a case of a renal transplant recipient with COVID-19 positive pneumonia: a case report. Pan Afr Med J. (2021) 38. doi: 10.11604/pamj.2021.38.273.28577 PMC8179983 34122700

[33] Kim NH Kim KJ Choi J Lee JH Park JH Kim JH . Metabolically unhealthy individuals, either with obesity or not, have a higher risk of critical coronavirus disease 2019 outcomes than metabolically healthy individuals without obesity. Metab Clin Exp. (2022) 128. doi: 10.1016/j.metabol.2021.154894 34600905 PMC8482539

[34] Marchesi F Salmanton-Garcia J Emarah Z Busca A Corradini P Hoenigl M . COVID-19 in adult acute myeloid leukemia patients: a long-term follow-up study from the European Hematology Association survey (EPICOVIDEHA). Haematologica. (2023) 108:22–33. doi: 10.3324/haematol.2022.280847 PMC9827164 35545919

[35] Nieman DC Groen AJ Pugachev A Lucas SJ Shek KL Peake J . Proteomics-based detection of immune dysfunction in an elite adventure athlete trekking across the Antarctica. Proteomes. (2020) 8. doi: 10.3390/proteomes8010004 32138228 PMC7151708

[36] True AL Olive M Boehm M Sato K Wigley WC Chen B . Heme oxygenase-1 deficiency accelerates formation of arterial thrombosis through oxidative damage to the endothelium, which is rescued by inhaled carbon monoxide. Circ Res. (2007) 101:893–901. doi: 10.1161/circresaha.107.158998 17885218

[37] Seldon MP Silva G Pejanovic N Larsen R Soares MP Anrather J . Heme oxygenase-1 inhibits the expression of adhesion molecules associated with endothelial cell activation via inhibition of NF-kappa B RelA phosphorylation at serine 276. J Immunol. (2007) 179:7840–51. doi: 10.4049/jimmunol.179.11.7840 18025230

[38] Wang ZP Liu RF Wang AR . Comparison of HMOX1 expression and enzyme activity in blue-shelled chickens and brown-shelled chickens. Genet Mol Biol. (2013) 36:282–86. doi: 10.1590/s1415-47572013000200020 23885212 PMC3715296

[39] Wider C Ross OA Nishioka K Gilman S Monsell SE Koeppe RA . An evaluation of the impact of MAPT, SNCA and APOE on the burden of Alzheimer's and Lewy body pathology. J Neurol Neurosurg Psychiatry. (2012) 83:424–29. doi: 10.1136/jnnp.2011.261426 PMC3623699 22291217

[40] Bachhuber T Katzmarski N Mccarter JF Felsenstein K Lorenz-Depiereux B Ebert A . Inhibition of amyloid-beta plaque formation by alpha-synuclein. Nat Med. (2015) 21:802–7. doi: 10.1038/nm.3885 26099047

[41] Nuovo GJ Suster D Sawant D Popek E Kearns J Magro CM . The amplification of CNS damage in Alzheimer's disease due to SARS-CoV2 infection*. Ann Diagn Pathol. (2022) 61. doi: 10.1016/j.anndiagpath.2022.152057 36334414 PMC9616485

[42] Crunfli F Carregari VC Veras FP Silva L Silva M Nascimento J . Morphological, cellular, and molecular basis of brain infection in COVID-19 patients. PNAS. (2022) 119. doi: 10.1073/pnas.2200960119 35951647 PMC9436354

[43] Shimokihara S Maruta M Hidaka Y Yoshinaga T Kondo I Yoshimura K . Relationship of decrease in frequency of socialization to daily life, social life, and physical function in community-dwelling adults aged 60 and over after the COVID-19 pandemic. Int J Environ Res Public Health. (2021) 18. doi: 10.3390/ijerph18052573 33806599 PMC7967503

[44] Premkumar T Sajitha Lulu S . Molecular crosstalk between COVID-19 and Alzheimer's disease using microarray and RNA-seq datasets: A system biology approach. Front Med. (2023) 10:1151046. doi: 10.3389/fmed.2023.1151046 37359008 PMC10286240

[45] Cao SN Hou GJ Meng Y Li Y Zhang Y Wang Y . Network pharmacology and molecular docking-based investigation of potential targets of Astragalus membranaceus and Angelica sinensis compound acting on spinal cord injury. Dis Markers. (2022) 2022. doi: 10.1155/2022/2141882 36157206 PMC9499798

[46] Yu YH Xin F Dong L Li Y Zhang Y Liu X . Weighted gene coexpression network analysis identifies critical genes in different subtypes of acute myeloid leukaemia. Biotechnol Biotechnol Equip. (2020) 34:925–36. doi: 10.1080/13102818.2020.181176737339054

[47] Dong M Li P Luo J Liu Y Zhang Y Zhao Y . Oligopeptide/Histidine transporter PHT1 and PHT2 - function, regulation, and pathophysiological implications specifically in immunoregulation. Pharm Res. (2023) 40:2585–96. doi: 10.1007/s11095-023-03589-8 37610621

[48] Döhner H Wei AH Appelbaum FR Craddock C DiNardo CD Dombret H . Diagnosis and management of AML in adults: 2022 recommendations from an international expert panel on behalf of the ELN. Blood. (2022) 140:1345–77. doi: 10.1182/blood.2022016867 35797463

[49] Li Y Cui X Yang X Wang Y Zhang L Liu H . Artificial intelligence in predicting pathogenic microorganisms' antimicrobial resistance: challenges, progress, and prospects. Front Cell Infect Microbiol. (2024) 14:1482186. doi: 10.3389/fcimb.2024.1482186 39554812 PMC11564165

