# Supplementary material for: Identification of SLC15A3 as a shared prognostic biomarker for AML and COVID-19 via integrative bioinformatics, machine learning and Mendelian randomization
Source: Front Oncol. 2026 Oct 14;16:1911550. doi: 10.3389/fonc.2026.1911550 (PMC13713349; doi:10.3389/fonc.2026.1911550)
Supplement: Supplementary file 1 [file Table1.doc]

**Supplementary Table S1 The differentially expressed mRNAs of GSE180226**

| id | logFC | AveExpr | t | P.Value | adj.P.Val | B |
| --- | --- | --- | --- | --- | --- | --- |
| VPS18 | 5.238201108 | 15.16571303 | 17.453608 | 6.72E-16 | 8.64E-12 | 25.54410157 |
| CAV1 | -4.426634379 | 9.020916683 | -13.3156219 | 3.89E-13 | 2.50E-09 | 19.81345108 |
| LRRC2 | 8.198291003 | 13.98644495 | 13.02543265 | 6.42E-13 | 2.75E-09 | 19.34767987 |
| CD86 | 5.668334144 | 13.82897236 | 12.63972009 | 1.27E-12 | 4.08E-09 | 18.71383852 |
| LOC100287803 | 4.739712367 | 9.660517204 | 12.43483492 | 1.83E-12 | 4.71E-09 | 18.37014265 |
| LOC100129292 | 2.889470198 | 10.50089823 | 11.66976143 | 7.51E-12 | 1.61E-08 | 17.04220955 |
| BTF3P11 | -3.280423422 | 8.78321832 | -11.34449456 | 1.40E-11 | 2.30E-08 | 16.45567006 |
| YY1 | -2.783838675 | 7.400374319 | -11.3326278 | 1.43E-11 | 2.30E-08 | 16.43401673 |
| KLF5 | -3.108607121 | 6.503802661 | -11.08080975 | 2.33E-11 | 3.33E-08 | 15.97024059 |
| ATP5A1 | -4.236551703 | 7.606977611 | -11.00143108 | 2.72E-11 | 3.50E-08 | 15.82233951 |
| UBE2C | 3.849698596 | 9.590297581 | 10.62461752 | 5.75E-11 | 6.72E-08 | 15.10890454 |
| INPP5A | -2.814574989 | 6.767576638 | -10.22102509 | 1.31E-10 | 1.21E-07 | 14.3236218 |
| COX4I1 | -3.732561453 | 9.178657451 | -10.21278073 | 1.33E-10 | 1.21E-07 | 14.30734911 |
| E2F2 | 3.277761781 | 8.647378568 | 10.20563898 | 1.35E-10 | 1.21E-07 | 14.29324523 |
| C2orf28 | -4.042027279 | 5.749760712 | -10.17229863 | 1.44E-10 | 1.21E-07 | 14.22731023 |
| GIMAP8 | -3.130211529 | 7.695627149 | -10.12466832 | 1.59E-10 | 1.21E-07 | 14.13284961 |
| LIMCH1 | -3.456883963 | 9.217678714 | -10.1221224 | 1.60E-10 | 1.21E-07 | 14.12779174 |
| PDZD2 | -2.906460832 | 5.743820297 | -9.998301166 | 2.07E-10 | 1.48E-07 | 13.88071986 |
| ARHGAP11A | 2.955977767 | 7.107921143 | 9.945078818 | 2.32E-10 | 1.52E-07 | 13.7738676 |
| EIF4G2 | -3.663662484 | 9.380892772 | -9.915814663 | 2.46E-10 | 1.52E-07 | 13.71494742 |
| RAC1 | -2.78800589 | 8.807433047 | -9.899950188 | 2.55E-10 | 1.52E-07 | 13.68295621 |
| LOC100653218 | 2.946618697 | 8.910391535 | 9.877248401 | 2.67E-10 | 1.52E-07 | 13.63711635 |
| ATP5B | -2.720625969 | 8.342572308 | -9.868160412 | 2.72E-10 | 1.52E-07 | 13.61874557 |
| LOC100652821 | 2.163439064 | 9.677069493 | 9.796088376 | 3.17E-10 | 1.60E-07 | 13.47264829 |
| FLII | -2.597759898 | 6.715441033 | -9.795110778 | 3.18E-10 | 1.60E-07 | 13.47066161 |
| C22orf43 | -4.28375075 | 6.531660754 | -9.786253773 | 3.24E-10 | 1.60E-07 | 13.45265625 |
| MAP3K10 | 2.757741962 | 10.24082091 | 9.72842754 | 3.66E-10 | 1.74E-07 | 13.33483155 |
| USO1 | -2.934750444 | 7.217171565 | -9.653319182 | 4.29E-10 | 1.97E-07 | 13.18109288 |
| MEG3 | 3.079530107 | 9.145757209 | 9.621644442 | 4.59E-10 | 2.04E-07 | 13.11602032 |
| RPL19P12 | -2.968135876 | 10.2874062 | -9.582947425 | 4.99E-10 | 2.14E-07 | 13.03632943 |
| CENPT | 2.627182781 | 7.708975595 | 9.54923986 | 5.36E-10 | 2.22E-07 | 12.96674151 |
| HIST1H3B | 3.612047818 | 9.285612345 | 9.493446114 | 6.04E-10 | 2.42E-07 | 12.85120495 |
| BCAM | 2.614820982 | 14.43965398 | 9.464100286 | 6.44E-10 | 2.42E-07 | 12.79025957 |
| LMBRD1 | -2.831453307 | 8.696932559 | -9.458202481 | 6.52E-10 | 2.42E-07 | 12.77799631 |
| AAMP | -2.974392717 | 5.005523113 | -9.452932283 | 6.59E-10 | 2.42E-07 | 12.76703386 |
| COL27A1 | 2.580646497 | 10.61937121 | 9.441345181 | 6.76E-10 | 2.42E-07 | 12.74291789 |
| DHRS7 | -2.794337973 | 6.702809658 | -9.417880028 | 7.11E-10 | 2.47E-07 | 12.69402215 |
| RNU12 | 3.633054661 | 11.05173378 | 9.379313043 | 7.73E-10 | 2.62E-07 | 12.61348824 |
| PEX2 | -3.160364839 | 5.729131944 | -9.307434633 | 9.04E-10 | 2.98E-07 | 12.46283149 |
| TROAP | 3.20427875 | 9.219788942 | 9.273966919 | 9.72E-10 | 3.10E-07 | 12.39243284 |
| ETF1 | -2.829205786 | 6.613484344 | -9.258146134 | 1.01E-09 | 3.10E-07 | 12.35909868 |
| GABARAPL2 | -2.772889256 | 9.34927298 | -9.255941895 | 1.01E-09 | 3.10E-07 | 12.35445155 |
| LOC100652828 | -2.567171921 | 7.312332872 | -9.231860347 | 1.07E-09 | 3.14E-07 | 12.30363616 |
| RPL23AP82 | -3.35849503 | 11.90637252 | -9.223886904 | 1.08E-09 | 3.14E-07 | 12.2867929 |
| NCOA1 | -2.769138766 | 6.150932183 | -9.211545906 | 1.11E-09 | 3.14E-07 | 12.26070568 |
| C1orf43 | -2.030034927 | 8.046794778 | -9.207018315 | 1.12E-09 | 3.14E-07 | 12.25112952 |
| DRD4 | 2.756090722 | 14.40582447 | 9.197661271 | 1.15E-09 | 3.14E-07 | 12.23132948 |
| C14orf119 | -3.422507975 | 7.097473644 | -9.174089251 | 1.21E-09 | 3.24E-07 | 12.18139444 |
| ATP1A1 | -2.592646274 | 9.988268939 | -9.12034413 | 1.36E-09 | 3.48E-07 | 12.06724437 |
| PKMYT1 | 3.294603511 | 8.634185197 | 9.109511448 | 1.39E-09 | 3.48E-07 | 12.04418679 |
| EMILIN1 | 2.292015899 | 10.08452932 | 9.096960936 | 1.43E-09 | 3.48E-07 | 12.01745182 |
| FAM96B | -2.762982026 | 7.831566482 | -9.089907066 | 1.45E-09 | 3.48E-07 | 12.00241588 |
| PRPF6 | -2.542450784 | 6.817083743 | -9.087110885 | 1.46E-09 | 3.48E-07 | 11.9964536 |
| PIF1 | 2.680985351 | 7.4996728 | 9.075003805 | 1.50E-09 | 3.48E-07 | 11.9706249 |
| PRDX5 | -3.79109579 | 10.51043151 | -9.073665069 | 1.51E-09 | 3.48E-07 | 11.96776762 |
| HIST1H4J | 3.102746973 | 10.82843434 | 9.072097632 | 1.51E-09 | 3.48E-07 | 11.96442189 |
| PARD6B | -4.193946451 | 4.943466099 | -9.042265736 | 1.62E-09 | 3.65E-07 | 11.90067816 |
| C8orf40 | -3.923300238 | 4.94182109 | -8.99186054 | 1.81E-09 | 4.01E-07 | 11.79268526 |
| DHX30 | -3.003742345 | 5.451666652 | -8.963854921 | 1.92E-09 | 4.18E-07 | 11.73252645 |
| CCT7 | -3.00568184 | 9.311174152 | -8.956056012 | 1.96E-09 | 4.18E-07 | 11.71575366 |
| SPRY2 | -2.951968308 | 7.053177075 | -8.939532083 | 2.03E-09 | 4.18E-07 | 11.68018763 |
| MRFAP1 | -2.258652281 | 8.463859036 | -8.929265121 | 2.08E-09 | 4.18E-07 | 11.65806939 |
| KIAA1826 | -2.200021068 | 5.037824316 | -8.920445688 | 2.12E-09 | 4.18E-07 | 11.63905755 |
| RAB11A | -3.192097328 | 7.786786183 | -8.91895038 | 2.12E-09 | 4.18E-07 | 11.63583305 |
| ANXA3 | -4.327794402 | 7.818276091 | -8.918034518 | 2.13E-09 | 4.18E-07 | 11.63385792 |
| PRMT5 | -3.204917365 | 5.113308083 | -8.909456343 | 2.17E-09 | 4.18E-07 | 11.61535252 |
| NUSAP1 | 2.619733073 | 7.730540064 | 8.903522551 | 2.20E-09 | 4.18E-07 | 11.6025456 |
| C19orf56 | -2.211977647 | 8.18201455 | -8.901654635 | 2.21E-09 | 4.18E-07 | 11.59851303 |
| TMEFF2 | 6.558441407 | 11.69280195 | 8.880514986 | 2.31E-09 | 4.31E-07 | 11.5528407 |
| RPS5 | -3.417218964 | 8.301983077 | -8.860688822 | 2.42E-09 | 4.45E-07 | 11.50994806 |
| LOC100506027 | 4.427995507 | 9.92653317 | 8.808279085 | 2.72E-09 | 4.93E-07 | 11.39629217 |
| TATDN1 | -2.510544245 | 5.620492487 | -8.778142869 | 2.91E-09 | 5.20E-07 | 11.33076074 |
| ORC6 | 2.3562949 | 7.823603888 | 8.762084263 | 3.02E-09 | 5.32E-07 | 11.29578812 |
| ZNF713 | 2.517473323 | 11.14529259 | 8.75554097 | 3.06E-09 | 5.32E-07 | 11.28152748 |
| RPL4 | -2.85241467 | 11.84161118 | -8.72069663 | 3.31E-09 | 5.68E-07 | 11.20548356 |
| PHACTR4 | -2.111385718 | 6.282429601 | -8.711814651 | 3.38E-09 | 5.72E-07 | 11.18607185 |
| PRDX1 | -2.809706439 | 9.837507394 | -8.704567198 | 3.43E-09 | 5.74E-07 | 11.17022406 |
| FLJ11710 | 2.981902216 | 9.586072015 | 8.688500396 | 3.56E-09 | 5.87E-07 | 11.13506447 |
| JTB | -2.283114929 | 9.450590805 | -8.671093029 | 3.70E-09 | 6.02E-07 | 11.09692959 |
| LETMD1 | -2.539147131 | 7.536482857 | -8.666541049 | 3.74E-09 | 6.02E-07 | 11.08695028 |
| SLCO2A1 | -3.590615785 | 8.991288317 | -8.644577724 | 3.93E-09 | 6.25E-07 | 11.03875839 |
| EFR3A | -2.203528772 | 8.66893572 | -8.600272691 | 4.35E-09 | 6.82E-07 | 10.9413345 |
| GPD1L | -3.386929839 | 7.944595073 | -8.57659464 | 4.59E-09 | 6.98E-07 | 10.88915296 |
| EXPH5 | -2.376524318 | 7.490629513 | -8.57535911 | 4.60E-09 | 6.98E-07 | 10.8864279 |
| JAK3 | 3.67644877 | 10.87457547 | 8.574489728 | 4.61E-09 | 6.98E-07 | 10.88451029 |
| IRX5 | 3.999885937 | 14.00435698 | 8.539831405 | 4.99E-09 | 7.34E-07 | 10.80797564 |
| THY1 | 3.792815764 | 11.17852228 | 8.53727106 | 5.02E-09 | 7.34E-07 | 10.80231492 |
| SMARCA4 | 3.596034121 | 13.5049852 | 8.53200157 | 5.08E-09 | 7.34E-07 | 10.79066156 |
| CD9 | -2.416695623 | 8.247852833 | -8.531796654 | 5.08E-09 | 7.34E-07 | 10.79020831 |
| ZNF564 | -2.58024962 | 4.150532936 | -8.490796025 | 5.58E-09 | 7.97E-07 | 10.69939966 |
| C22orf32 | -2.788788805 | 8.022375274 | -8.466943411 | 5.89E-09 | 8.33E-07 | 10.64646025 |
| CA2 | -3.269307409 | 7.912208526 | -8.449336167 | 6.13E-09 | 8.57E-07 | 10.60733 |
| SDHB | -2.420482549 | 7.805309169 | -8.430048814 | 6.41E-09 | 8.86E-07 | 10.56441515 |
| BGN | 2.341782238 | 8.641259469 | 8.417303892 | 6.60E-09 | 9.03E-07 | 10.53602828 |
| NDUFB11 | -2.57772353 | 9.463924332 | -8.409673389 | 6.72E-09 | 9.09E-07 | 10.51902172 |
| EXOC3L2 | 2.623915759 | 14.45464392 | 8.404433747 | 6.80E-09 | 9.11E-07 | 10.50733901 |
| C15orf42 | 3.314519283 | 7.497890503 | 8.394669679 | 6.95E-09 | 9.22E-07 | 10.48555787 |
| PPA1 | -3.435159096 | 8.972641595 | -8.365394432 | 7.44E-09 | 9.76E-07 | 10.4201709 |
| POLR1D | -2.558039261 | 6.990744604 | -8.345117322 | 7.79E-09 | 1.00E-06 | 10.37480999 |
| EFNB2 | -3.021209071 | 6.902027008 | -8.338684056 | 7.91E-09 | 1.00E-06 | 10.36040623 |
| HIST1H1B | 3.24896951 | 7.29735713 | 8.338512588 | 7.91E-09 | 1.00E-06 | 10.36002225 |
| SURF1 | -3.247954205 | 6.081882945 | -8.336117683 | 7.95E-09 | 1.00E-06 | 10.35465861 |
| TSPAN1 | -2.932143157 | 7.028784256 | -8.250749667 | 9.69E-09 | 1.21E-06 | 10.16293613 |
| LOC100652824 | 3.42197817 | 10.4657742 | 8.238575551 | 9.96E-09 | 1.22E-06 | 10.13551082 |
| RELT | 2.316812315 | 9.98999145 | 8.237293705 | 9.99E-09 | 1.22E-06 | 10.13262191 |
| PPP6R1 | 6.274017655 | 12.15430229 | 8.223627526 | 1.03E-08 | 1.25E-06 | 10.10180784 |
| HLA-DRB1 | -2.183478653 | 11.24582086 | -8.20973808 | 1.07E-08 | 1.28E-06 | 10.07046325 |
| LOC729313 | -2.418841467 | 10.36137363 | -8.203521425 | 1.08E-08 | 1.29E-06 | 10.05642515 |
| ZC3H15 | -2.30785727 | 7.992405929 | -8.197308652 | 1.10E-08 | 1.29E-06 | 10.04239035 |
| ACIN1 | 2.59598305 | 9.743714988 | 8.186257862 | 1.12E-08 | 1.31E-06 | 10.01741287 |
| CLDN7 | -2.792593916 | 6.819167542 | -8.183026199 | 1.13E-08 | 1.31E-06 | 10.01010526 |
| TANC2 | -2.290030524 | 5.876576652 | -8.169569324 | 1.17E-08 | 1.34E-06 | 9.97965996 |
| RPSAP52 | -2.855769456 | 6.682269395 | -8.162669783 | 1.19E-08 | 1.34E-06 | 9.964040275 |
| TMEM209 | -2.144900329 | 6.590856254 | -8.158611029 | 1.20E-08 | 1.34E-06 | 9.954848629 |
| FAM190B | -2.730643953 | 6.857244671 | -8.157757922 | 1.20E-08 | 1.34E-06 | 9.952916347 |
| C9orf5 | -2.761367416 | 9.149425342 | -8.120292999 | 1.31E-08 | 1.45E-06 | 9.867957166 |
| PKN2 | 2.391259248 | 14.49970834 | 8.095781888 | 1.39E-08 | 1.50E-06 | 9.812266197 |
| TSTD1 | -4.370284851 | 6.837771874 | -8.094576656 | 1.39E-08 | 1.50E-06 | 9.80952564 |
| SYMPK | 2.007142977 | 9.126126685 | 8.090125686 | 1.41E-08 | 1.50E-06 | 9.799402885 |
| ARPC1A | -2.15936927 | 7.925301461 | -8.089738781 | 1.41E-08 | 1.50E-06 | 9.798522824 |
| FHIT | 2.768963028 | 13.70431102 | 8.088223145 | 1.41E-08 | 1.50E-06 | 9.795075123 |
| DRG1 | -2.435135605 | 6.805881712 | -8.086230219 | 1.42E-08 | 1.50E-06 | 9.790541211 |
| ATOH8 | -4.389972967 | 7.184244166 | -8.079448243 | 1.44E-08 | 1.50E-06 | 9.77510801 |
| HIST1H4K | 2.76286049 | 9.55545838 | 8.079236504 | 1.44E-08 | 1.50E-06 | 9.774626067 |
| SH3BGRL | -2.099305655 | 8.806201658 | -8.058804889 | 1.51E-08 | 1.56E-06 | 9.728091746 |
| CPA3 | -2.825741304 | 7.304235406 | -8.049737719 | 1.55E-08 | 1.58E-06 | 9.707421873 |
| SPIN1 | -2.26723639 | 7.485757698 | -8.026934738 | 1.63E-08 | 1.65E-06 | 9.655388256 |
| SERPINH1 | 3.116641148 | 10.92047556 | 8.002076016 | 1.73E-08 | 1.72E-06 | 9.59858054 |
| HIST1H2AJ | 2.50502545 | 8.850000239 | 8.001960863 | 1.73E-08 | 1.72E-06 | 9.598317189 |
| MYLIP | -3.274429563 | 6.541105656 | -7.980083383 | 1.82E-08 | 1.80E-06 | 9.54825026 |
| FXYD3 | -4.000618973 | 5.977603181 | -7.969285879 | 1.87E-08 | 1.83E-06 | 9.523515314 |
| TXN2 | -2.498041409 | 6.918415215 | -7.95583678 | 1.93E-08 | 1.88E-06 | 9.492683282 |
| TMEM116 | -2.818999565 | 5.178347943 | -7.942374448 | 1.99E-08 | 1.92E-06 | 9.461795591 |
| LOC93622 | -3.17635268 | 6.269153816 | -7.932176596 | 2.04E-08 | 1.96E-06 | 9.438381004 |
| ARL6IP6 | -2.310857021 | 6.350502795 | -7.92900601 | 2.05E-08 | 1.96E-06 | 9.43109828 |
| TIMP3 | -3.943965313 | 10.8986815 | -7.922646725 | 2.08E-08 | 1.97E-06 | 9.416487 |
| MRPL18 | -2.329552226 | 9.061925532 | -7.919405944 | 2.10E-08 | 1.97E-06 | 9.409038718 |
| HMX2 | 2.611774149 | 7.968468968 | 7.912550717 | 2.14E-08 | 1.99E-06 | 9.39327853 |
| PTTG2 | 2.165328836 | 8.2928687 | 7.908653426 | 2.15E-08 | 1.99E-06 | 9.384315725 |
| AGAP2 | 2.799487414 | 9.769752886 | 7.903152374 | 2.18E-08 | 1.99E-06 | 9.371661067 |
| LSM10 | -2.676103383 | 5.145672697 | -7.903056085 | 2.18E-08 | 1.99E-06 | 9.371439525 |
| RPS16 | -3.356892521 | 12.24894532 | -7.897433951 | 2.21E-08 | 2.00E-06 | 9.358501896 |
| GNAI1 | -3.150456965 | 4.894572415 | -7.893243757 | 2.23E-08 | 2.01E-06 | 9.348856577 |
| NSA2 | -2.129306289 | 7.659419946 | -7.890252606 | 2.25E-08 | 2.01E-06 | 9.341969816 |
| RPL18 | -3.418631051 | 10.24590659 | -7.871056405 | 2.36E-08 | 2.09E-06 | 9.297743262 |
| CACNA1C | 2.265288301 | 9.31473272 | 7.861323988 | 2.41E-08 | 2.12E-06 | 9.275300959 |
| ANP32A-IT1 | 2.819159271 | 10.5773657 | 7.831832805 | 2.58E-08 | 2.26E-06 | 9.207215958 |
| SCAI | -3.26654452 | 5.702202513 | -7.824539505 | 2.63E-08 | 2.28E-06 | 9.190359633 |
| ATG7 | -2.274205266 | 5.198566662 | -7.821313136 | 2.65E-08 | 2.29E-06 | 9.182900475 |
| RPL15 | -3.40545396 | 9.394330687 | -7.812222033 | 2.71E-08 | 2.32E-06 | 9.161874677 |
| CABP7 | 7.238616032 | 11.78942441 | 7.805947399 | 2.75E-08 | 2.34E-06 | 9.147356103 |
| STX1A | 2.114052312 | 7.77834939 | 7.799938488 | 2.79E-08 | 2.36E-06 | 9.133447268 |
| APOBEC3D | 2.260999878 | 7.217739427 | 7.791067275 | 2.85E-08 | 2.39E-06 | 9.112903932 |
| POLE4 | -2.575704425 | 7.353967237 | -7.782166227 | 2.91E-08 | 2.43E-06 | 9.092280575 |
| SSTR1 | -4.551678268 | 5.084560552 | -7.76780021 | 3.01E-08 | 2.49E-06 | 9.058972043 |
| CTNNBIP1 | -3.06811267 | 10.12755513 | -7.766908506 | 3.01E-08 | 2.49E-06 | 9.056903633 |
| PDIK1L | -2.178848988 | 5.194667305 | -7.760147595 | 3.06E-08 | 2.51E-06 | 9.041217342 |
| C2orf40 | -4.473278817 | 5.643525344 | -7.748578921 | 3.15E-08 | 2.56E-06 | 9.014361739 |
| CSNK1G2 | 1.731082529 | 11.11509984 | 7.742622219 | 3.19E-08 | 2.56E-06 | 9.000526618 |
| MYO5B | -2.289843208 | 7.501310328 | -7.741828177 | 3.20E-08 | 2.56E-06 | 8.998681995 |
| SAMD12 | -2.697902277 | 4.578954402 | -7.740866124 | 3.21E-08 | 2.56E-06 | 8.996446955 |
| ADAMTS7 | 2.78916021 | 10.54548285 | 7.718490695 | 3.38E-08 | 2.68E-06 | 8.944428461 |
| CDKAL1 | 3.113748757 | 9.732734814 | 7.716679552 | 3.40E-08 | 2.68E-06 | 8.940214898 |
| POLR2B | -2.003978438 | 7.051936722 | -7.714170357 | 3.42E-08 | 2.68E-06 | 8.934376596 |
| PGM1 | -3.145839891 | 8.047890833 | -7.708847703 | 3.46E-08 | 2.70E-06 | 8.92198918 |
| SPAG7 | -2.914553848 | 5.177647611 | -7.695724356 | 3.57E-08 | 2.77E-06 | 8.891430605 |
| SH2B3 | -2.700214424 | 5.790922693 | -7.687816245 | 3.64E-08 | 2.80E-06 | 8.873004649 |
| SPG20 | -2.410373405 | 5.976197763 | -7.685516299 | 3.66E-08 | 2.80E-06 | 8.86764415 |
| STX10 | 2.001751229 | 9.326800416 | 7.664722629 | 3.85E-08 | 2.93E-06 | 8.819147346 |
| PHB | -1.84752408 | 7.2323 | -7.643146151 | 4.05E-08 | 3.02E-06 | 8.76876234 |
| COPB1 | -2.375709742 | 6.107713366 | -7.641177426 | 4.07E-08 | 3.02E-06 | 8.764161844 |
| HAGH | -3.225153253 | 5.960551309 | -7.639366013 | 4.09E-08 | 3.02E-06 | 8.759928487 |
| CCNI | -3.103789618 | 10.93232692 | -7.63739056 | 4.11E-08 | 3.02E-06 | 8.755311254 |
| SBDS | -2.361371412 | 7.295502508 | -7.636550499 | 4.12E-08 | 3.02E-06 | 8.753347614 |
| SLC25A11 | -2.452157135 | 4.764299335 | -7.63506338 | 4.13E-08 | 3.02E-06 | 8.749871241 |
| INE1 | 2.320196754 | 9.018485603 | 7.634689285 | 4.13E-08 | 3.02E-06 | 8.748996688 |
| TACC3 | 2.193697362 | 7.127077318 | 7.623686306 | 4.24E-08 | 3.07E-06 | 8.723265594 |
| NBAS | -2.305094302 | 4.148227642 | -7.623093509 | 4.25E-08 | 3.07E-06 | 8.721878836 |
| STUB1 | -3.062215405 | 8.628343456 | -7.614247825 | 4.34E-08 | 3.12E-06 | 8.701180034 |
| PMM1 | -2.513687958 | 6.422046454 | -7.612402494 | 4.36E-08 | 3.12E-06 | 8.696860639 |
| ATP5E | 2.327976636 | 14.07398358 | 7.609187845 | 4.39E-08 | 3.12E-06 | 8.689334952 |
| ELOF1 | -2.419170451 | 5.003521381 | -7.605824058 | 4.43E-08 | 3.13E-06 | 8.681458622 |
| PSMA2 | -2.395095552 | 8.717454731 | -7.594089644 | 4.56E-08 | 3.18E-06 | 8.653970374 |
| CAPNS1 | -2.215040379 | 9.035764084 | -7.591573829 | 4.58E-08 | 3.18E-06 | 8.648074562 |
| ITGA7 | 2.894866868 | 11.85003428 | 7.589881086 | 4.60E-08 | 3.18E-06 | 8.644107135 |
| MICALL2 | 2.916670718 | 10.66602306 | 7.588767071 | 4.61E-08 | 3.18E-06 | 8.641495911 |
| GABARAP | -3.020279723 | 9.270247574 | -7.588280898 | 4.62E-08 | 3.18E-06 | 8.64035628 |
| MYO6 | -2.440384781 | 7.346670449 | -7.560114956 | 4.94E-08 | 3.38E-06 | 8.574278253 |
| NSMCE1 | -2.3395238 | 6.450166974 | -7.552770222 | 5.03E-08 | 3.39E-06 | 8.557029669 |
| FOXF1 | -2.189084091 | 8.855893811 | -7.552533344 | 5.03E-08 | 3.39E-06 | 8.556473256 |
| RBM42 | -2.141278051 | 7.449782312 | -7.55232609 | 5.04E-08 | 3.39E-06 | 8.555986422 |
| MRPL38 | 2.289680159 | 10.05782347 | 7.534317336 | 5.26E-08 | 3.52E-06 | 8.513662229 |
| RPL29 | -2.344899887 | 10.69201612 | -7.52983038 | 5.32E-08 | 3.54E-06 | 8.503110171 |
| DBT | -2.21725894 | 5.951996615 | -7.526496173 | 5.36E-08 | 3.54E-06 | 8.495267297 |
| EPRS | -2.87640892 | 7.597764564 | -7.524813243 | 5.38E-08 | 3.54E-06 | 8.491308061 |
| TPST2 | -2.453476697 | 8.979984104 | -7.524081152 | 5.39E-08 | 3.54E-06 | 8.489585637 |
| UFC1 | -2.670871041 | 6.584737394 | -7.516098219 | 5.50E-08 | 3.58E-06 | 8.470799135 |
| NAV1 | 2.860501081 | 9.808227651 | 7.515180468 | 5.51E-08 | 3.58E-06 | 8.468638812 |
| PLP2 | -2.242758885 | 7.564536823 | -7.512241699 | 5.55E-08 | 3.59E-06 | 8.461720386 |
| NTN4 | -3.098014911 | 7.733298078 | -7.508514025 | 5.60E-08 | 3.60E-06 | 8.452943054 |
| NDUFA11 | -3.190056067 | 9.063442939 | -7.500035767 | 5.71E-08 | 3.66E-06 | 8.432972852 |
| TNFSF10 | -3.007606153 | 8.230890833 | -7.489741772 | 5.86E-08 | 3.73E-06 | 8.408712772 |
| RNF122 | 2.69017205 | 10.89461434 | 7.477765835 | 6.03E-08 | 3.82E-06 | 8.380470936 |
| TMPRSS2 | -2.871066793 | 6.345930791 | -7.470378036 | 6.14E-08 | 3.87E-06 | 8.363039331 |
| BIRC5 | 3.810576025 | 9.539488738 | 7.460746915 | 6.28E-08 | 3.91E-06 | 8.340303608 |
| NDUFA12 | -2.461283528 | 6.8151614 | -7.458057714 | 6.32E-08 | 3.91E-06 | 8.333953126 |
| CMPK1 | -3.731594861 | 5.004358656 | -7.456714857 | 6.34E-08 | 3.91E-06 | 8.330781639 |
| TMC4 | -2.48117505 | 7.215612551 | -7.455224077 | 6.37E-08 | 3.91E-06 | 8.327260517 |
| EIF2AK4 | -2.722861781 | 6.292445283 | -7.455098023 | 6.37E-08 | 3.91E-06 | 8.32696277 |
| FLNB | -2.523440287 | 6.640500634 | -7.454623156 | 6.38E-08 | 3.91E-06 | 8.325841096 |
| ITGB1BP1 | -3.247830285 | 6.011672638 | -7.446216743 | 6.51E-08 | 3.97E-06 | 8.305979469 |
| FLI1 | -2.617689992 | 8.276323848 | -7.440875416 | 6.59E-08 | 4.00E-06 | 8.293354747 |
| PORCN | 2.056172752 | 10.3376896 | 7.435181719 | 6.68E-08 | 4.04E-06 | 8.279892973 |
| BRI3BP | 1.869019459 | 8.145880419 | 7.432177975 | 6.73E-08 | 4.05E-06 | 8.272789392 |
| PTPRM | -2.204906667 | 7.328071319 | -7.420525606 | 6.93E-08 | 4.14E-06 | 8.245221234 |
| CDT1 | 3.350462867 | 7.957730656 | 7.400111846 | 7.28E-08 | 4.33E-06 | 8.196881109 |
| HTATSF1 | -2.790495589 | 8.480984253 | -7.398090549 | 7.31E-08 | 4.33E-06 | 8.192091634 |
| STK25 | -2.141005903 | 6.154114399 | -7.381971422 | 7.60E-08 | 4.49E-06 | 8.153877881 |
| CLU | -2.575576724 | 8.03198591 | -7.353498646 | 8.15E-08 | 4.79E-06 | 8.08629325 |
| SLC38A7 | 2.349359137 | 9.5720616 | 7.350693228 | 8.20E-08 | 4.80E-06 | 8.079628355 |
| PEF1 | -1.998896564 | 6.795715094 | -7.343785176 | 8.34E-08 | 4.86E-06 | 8.063212321 |
| UBE2O | 1.766294773 | 9.812509323 | 7.339106331 | 8.44E-08 | 4.89E-06 | 8.052090118 |
| ZMYM6NB | -2.523617296 | 6.157728197 | -7.321908592 | 8.80E-08 | 5.08E-06 | 8.011184203 |
| SHISA4 | 1.679306817 | 10.41737107 | 7.319619835 | 8.85E-08 | 5.08E-06 | 8.005737319 |
| POU5F1 | 2.178354312 | 8.328984309 | 7.304601797 | 9.18E-08 | 5.25E-06 | 7.969979698 |
| FBXL5 | -2.476010773 | 7.916749736 | -7.299411754 | 9.30E-08 | 5.29E-06 | 7.95761545 |
| TMEM147 | -2.926145224 | 7.974443543 | -7.294221456 | 9.41E-08 | 5.33E-06 | 7.945247069 |
| GPC4 | -2.043467927 | 7.070888851 | -7.286228099 | 9.60E-08 | 5.39E-06 | 7.926192165 |
| RAB9A | -2.545498022 | 7.563583528 | -7.284726295 | 9.63E-08 | 5.39E-06 | 7.922611171 |
| NDUFAB1 | -2.159969729 | 9.330385245 | -7.284553935 | 9.64E-08 | 5.39E-06 | 7.922200167 |
| PRRX1 | 2.611377065 | 7.145810089 | 7.270603848 | 9.97E-08 | 5.54E-06 | 7.888922335 |
| RNF6 | -2.551018231 | 6.373409353 | -7.269085679 | 1.00E-07 | 5.54E-06 | 7.885299222 |
| MAL2 | -3.389280521 | 8.263412245 | -7.267203471 | 1.01E-07 | 5.54E-06 | 7.880806914 |
| PPPDE1 | -1.945033632 | 7.280300576 | -7.266440763 | 1.01E-07 | 5.54E-06 | 7.878986409 |
| NDRG1 | -2.986559568 | 8.991575519 | -7.252272297 | 1.04E-07 | 5.71E-06 | 7.845154015 |
| SFT2D3 | -2.574955861 | 5.199564055 | -7.248784005 | 1.05E-07 | 5.73E-06 | 7.836820442 |
| SNRK | -2.406496262 | 5.859097596 | -7.236246344 | 1.08E-07 | 5.89E-06 | 7.80685477 |
| PDCD1 | 2.594272371 | 8.358078856 | 7.234510939 | 1.09E-07 | 5.89E-06 | 7.802705453 |
| GRHPR | -2.164385002 | 6.500922193 | -7.217044726 | 1.14E-07 | 6.12E-06 | 7.760922419 |
| RPL36AP40 | -2.431058422 | 7.023476483 | -7.201723743 | 1.18E-07 | 6.30E-06 | 7.724238804 |
| RPL10L | -2.224701663 | 8.775172195 | -7.20159706 | 1.18E-07 | 6.30E-06 | 7.723935355 |
| NCAPG | 3.330354957 | 9.031841527 | 7.192001593 | 1.21E-07 | 6.42E-06 | 7.700944998 |
| KAT2B | -3.162942722 | 5.525046408 | -7.190125233 | 1.21E-07 | 6.43E-06 | 7.696447931 |
| KLF10 | -2.974255679 | 5.603487307 | -7.18704927 | 1.22E-07 | 6.45E-06 | 7.689074797 |
| EMP2 | -3.381909097 | 11.64523404 | -7.184588646 | 1.23E-07 | 6.46E-06 | 7.683175763 |
| CCDC48 | -3.3768017 | 8.192840663 | -7.173796327 | 1.26E-07 | 6.61E-06 | 7.657293372 |
| BEND5 | -2.39165459 | 5.51449164 | -7.165462742 | 1.29E-07 | 6.72E-06 | 7.637297353 |
| PCM1 | -1.835702033 | 7.514593736 | -7.158830664 | 1.31E-07 | 6.80E-06 | 7.621377658 |
| LYSMD2 | -2.659535251 | 6.964159199 | -7.14037449 | 1.37E-07 | 7.09E-06 | 7.577045717 |
| YIPF6 | -3.034787707 | 7.061443855 | -7.134028353 | 1.39E-07 | 7.17E-06 | 7.561792181 |
| GPM6B | -2.827780825 | 6.786754749 | -7.127829653 | 1.42E-07 | 7.25E-06 | 7.546888074 |
| ZNF2 | 2.557874085 | 8.214564444 | 7.118568554 | 1.45E-07 | 7.39E-06 | 7.524611654 |
| DGCR6 | -2.747946106 | 4.30711506 | -7.115592992 | 1.46E-07 | 7.41E-06 | 7.517451997 |
| SRI | -2.072694903 | 8.547172822 | -7.11217642 | 1.47E-07 | 7.45E-06 | 7.509229816 |
| ERGIC2 | -2.187432264 | 6.585072447 | -7.108730811 | 1.48E-07 | 7.47E-06 | 7.500936258 |
| SAP25 | 1.562632754 | 15.58868877 | 7.107987693 | 1.49E-07 | 7.47E-06 | 7.49914738 |
| PDCD10 | -2.642522756 | 6.055828017 | -7.106244301 | 1.49E-07 | 7.47E-06 | 7.494950309 |
| CNBP | -1.899738979 | 9.54386656 | -7.095576209 | 1.53E-07 | 7.64E-06 | 7.469259384 |
| MOB2 | -2.039325114 | 6.459770972 | -7.09103942 | 1.55E-07 | 7.69E-06 | 7.458329514 |
| KIAA0196 | -2.605584828 | 7.260140453 | -7.086531158 | 1.57E-07 | 7.75E-06 | 7.447465794 |
| TMEM203 | -2.630058096 | 6.283338429 | -7.083588136 | 1.58E-07 | 7.77E-06 | 7.440372505 |
| CIB1 | -2.497131904 | 7.831726248 | -7.081824491 | 1.58E-07 | 7.77E-06 | 7.436121233 |
| HIST1H2AB | 2.762053181 | 6.923572814 | 7.080594889 | 1.59E-07 | 7.77E-06 | 7.43315704 |
| E2F3 | 1.825200653 | 8.693950527 | 7.078963996 | 1.60E-07 | 7.78E-06 | 7.429225167 |
| CTNND2 | -3.403573325 | 4.693107356 | -7.077200854 | 1.60E-07 | 7.78E-06 | 7.42497408 |
| TGFBR2 | -2.698556366 | 8.691366667 | -7.070489292 | 1.63E-07 | 7.88E-06 | 7.408788345 |
| SLC25A5 | -2.338309519 | 9.801779621 | -7.066211125 | 1.65E-07 | 7.93E-06 | 7.398468073 |
| PDP1 | -2.556672226 | 6.701878844 | -7.063931428 | 1.66E-07 | 7.95E-06 | 7.392967794 |
| MAPK11 | 1.99534919 | 9.41587823 | 7.058664761 | 1.68E-07 | 8.02E-06 | 7.380258284 |
| ENDOD1 | -1.89253191 | 8.200163219 | -7.054592264 | 1.69E-07 | 8.06E-06 | 7.370428155 |
| MTCH1 | -2.923763971 | 13.29420031 | -7.053596898 | 1.70E-07 | 8.06E-06 | 7.368025238 |
| NR3C2 | -3.558565086 | 4.731783377 | -7.046882066 | 1.73E-07 | 8.17E-06 | 7.3518117 |
| ACTC1 | 2.095974399 | 9.368789863 | 7.041173219 | 1.75E-07 | 8.25E-06 | 7.338022757 |
| PMM2 | -2.993176343 | 5.91334717 | -7.035433358 | 1.78E-07 | 8.31E-06 | 7.324154793 |
| NUDT5 | -2.705382011 | 7.043216649 | -7.03533874 | 1.78E-07 | 8.31E-06 | 7.323926153 |
| ALG9 | -1.594578 | 6.329507887 | -7.033818883 | 1.78E-07 | 8.31E-06 | 7.320253348 |
| GUCY1A3 | -2.840072107 | 8.16014709 | -7.031351856 | 1.80E-07 | 8.33E-06 | 7.31429105 |
| GPATCH8 | -2.370302209 | 6.045701086 | -7.02588453 | 1.82E-07 | 8.42E-06 | 7.301074935 |
| SEC14L3 | -3.403442017 | 4.635542304 | -7.01856266 | 1.85E-07 | 8.54E-06 | 7.283370011 |
| PSMA5 | -1.798906385 | 8.76818912 | -6.999670275 | 1.94E-07 | 8.91E-06 | 7.23765574 |
| UBE2I | -1.810907861 | 8.301619345 | -6.998796938 | 1.95E-07 | 8.91E-06 | 7.235541437 |
| CYB5R1 | -2.536291117 | 8.340600798 | -6.990902496 | 1.98E-07 | 9.05E-06 | 7.216425118 |
| ZNF281 | -2.363768324 | 6.014154303 | -6.986248622 | 2.01E-07 | 9.12E-06 | 7.205152189 |
| C4orf48 | 1.868894768 | 9.575046253 | 6.984940048 | 2.01E-07 | 9.12E-06 | 7.201981988 |
| NDUFV2 | -3.368825509 | 8.268580928 | -6.979559169 | 2.04E-07 | 9.21E-06 | 7.188943847 |
| SULT1A1 | -2.580758344 | 5.789901654 | -6.969099171 | 2.09E-07 | 9.39E-06 | 7.163588509 |
| MEF2D | 2.683625386 | 9.40342247 | 6.969069365 | 2.09E-07 | 9.39E-06 | 7.163516239 |
| ATP10B | 3.109217456 | 12.4268288 | 6.946211873 | 2.22E-07 | 9.89E-06 | 7.108062034 |
| PPM1G | -1.719747973 | 6.457452846 | -6.945237627 | 2.22E-07 | 9.89E-06 | 7.105697005 |
| DAZAP2 | -2.83351891 | 12.03134348 | -6.941562697 | 2.24E-07 | 9.94E-06 | 7.096774888 |
| IMP4 | -2.249354564 | 6.265593583 | -6.940261933 | 2.25E-07 | 9.94E-06 | 7.093616452 |
| CD79A | 2.310916941 | 11.29979177 | 6.938426648 | 2.26E-07 | 9.95E-06 | 7.089159775 |
| C11orf73 | -2.40317319 | 7.46068789 | -6.935994248 | 2.27E-07 | 9.96E-06 | 7.083252469 |
| CEP350 | -2.165104754 | 6.946079256 | -6.935451692 | 2.28E-07 | 9.96E-06 | 7.081934722 |
| RTN3 | 2.602217154 | 13.26030364 | 6.932281257 | 2.29E-07 | 1.00E-05 | 7.074233731 |
| MAGED2 | -2.090991929 | 7.042098786 | -6.917551091 | 2.38E-07 | 1.03E-05 | 7.038438037 |
| PRPF19 | -2.221401514 | 4.614769687 | -6.916149016 | 2.39E-07 | 1.03E-05 | 7.035029483 |
| NKAP | -1.706694621 | 5.867655445 | -6.911266819 | 2.42E-07 | 1.04E-05 | 7.02315861 |
| MMP14 | 2.331455143 | 8.206264673 | 6.904434958 | 2.46E-07 | 1.06E-05 | 7.006542335 |
| RPA4 | 5.252096754 | 11.83122764 | 6.903833353 | 2.46E-07 | 1.06E-05 | 7.005078854 |
| SLC27A3 | -2.45390605 | 6.786685658 | -6.899303555 | 2.49E-07 | 1.06E-05 | 6.994058138 |
| DCTN6 | -2.052362059 | 6.280310852 | -6.898762077 | 2.49E-07 | 1.06E-05 | 6.992740591 |
| UNC50 | -2.345390191 | 7.321382419 | -6.89726464 | 2.50E-07 | 1.06E-05 | 6.989096775 |
| LTA4H | -2.695414901 | 11.53058211 | -6.893457899 | 2.53E-07 | 1.07E-05 | 6.979832344 |
| ARFIP1 | -2.237643968 | 9.080360465 | -6.893264266 | 2.53E-07 | 1.07E-05 | 6.979361054 |
| KRT8 | 2.496765294 | 10.94051228 | 6.874672935 | 2.65E-07 | 1.11E-05 | 6.934089863 |
| MYPOP | 2.72570803 | 9.102449087 | 6.874447002 | 2.65E-07 | 1.11E-05 | 6.933539444 |
| NTPCR | -2.194098676 | 5.461339746 | -6.874197832 | 2.65E-07 | 1.11E-05 | 6.932932408 |
| CFL2 | -2.462572846 | 7.498486986 | -6.869091301 | 2.68E-07 | 1.12E-05 | 6.920490045 |
| CETN2 | -3.701468309 | 6.861741994 | -6.867275051 | 2.70E-07 | 1.12E-05 | 6.916063889 |
| SNORA70 | 2.355041884 | 9.290662721 | 6.867004842 | 2.70E-07 | 1.12E-05 | 6.915405362 |
| IK | -1.697551766 | 9.608212942 | -6.860304254 | 2.74E-07 | 1.13E-05 | 6.899072538 |
| CCNB1IP1 | -3.258106612 | 6.413914314 | -6.859642484 | 2.75E-07 | 1.13E-05 | 6.897459168 |
| SERP1 | -2.549484696 | 9.476257203 | -6.85942182 | 2.75E-07 | 1.13E-05 | 6.896921186 |
| GOT2 | -1.953273636 | 6.467072695 | -6.856641642 | 2.77E-07 | 1.13E-05 | 6.890142562 |
| CDH7 | 3.558128239 | 9.920190183 | 6.849169417 | 2.82E-07 | 1.15E-05 | 6.871919204 |
| GSTK1 | 1.59469456 | 7.83333207 | 6.84687384 | 2.84E-07 | 1.15E-05 | 6.866319377 |
| DDX1 | -2.085939057 | 9.547245332 | -6.842439525 | 2.87E-07 | 1.16E-05 | 6.855500526 |
| METTL21A | 2.74954902 | 13.16819807 | 6.842053293 | 2.87E-07 | 1.16E-05 | 6.854558085 |
| CTTN | 2.184606668 | 8.4747068 | 6.839250376 | 2.89E-07 | 1.16E-05 | 6.847718179 |
| CCNE1 | 2.438874379 | 10.59893109 | 6.827042144 | 2.98E-07 | 1.19E-05 | 6.817915704 |
| ACTR10 | -1.563201091 | 7.96756868 | -6.826554135 | 2.99E-07 | 1.19E-05 | 6.816724018 |
| TAPT1 | -2.401708306 | 6.545949709 | -6.819655376 | 3.04E-07 | 1.21E-05 | 6.799874651 |
| NOSTRIN | -2.582450869 | 9.69155412 | -6.819271867 | 3.04E-07 | 1.21E-05 | 6.798937812 |
| CDH22 | 3.107998211 | 10.48131181 | 6.817423699 | 3.05E-07 | 1.21E-05 | 6.794422841 |
| HSD17B4 | -2.726007622 | 8.406742805 | -6.817053193 | 3.06E-07 | 1.21E-05 | 6.793517669 |
| KLK3 | 2.504551985 | 14.17726199 | 6.799669293 | 3.19E-07 | 1.26E-05 | 6.751029165 |
| CYBRD1 | -2.255097468 | 8.936958469 | -6.796418788 | 3.22E-07 | 1.26E-05 | 6.743080533 |
| FN3KRP | -2.996756811 | 7.350916987 | -6.788933381 | 3.28E-07 | 1.28E-05 | 6.724771317 |
| TMEM59 | -1.866306513 | 11.11223423 | -6.784472017 | 3.32E-07 | 1.29E-05 | 6.713855728 |
| CENPO | 1.825096682 | 6.96728258 | 6.777707391 | 3.37E-07 | 1.31E-05 | 6.697300285 |
| DYNC1LI1 | -2.135501247 | 4.985557144 | -6.773746989 | 3.41E-07 | 1.32E-05 | 6.687605274 |
| MS4A7 | -3.299287832 | 6.991351144 | -6.769148125 | 3.45E-07 | 1.33E-05 | 6.676344999 |
| C12orf70 | 2.620454475 | 6.98246307 | 6.768256346 | 3.45E-07 | 1.33E-05 | 6.674161198 |
| MPV17 | -1.851467719 | 5.624187858 | -6.767501446 | 3.46E-07 | 1.33E-05 | 6.672312519 |
| GSTA4 | -3.722471396 | 6.695619719 | -6.761803583 | 3.51E-07 | 1.34E-05 | 6.658356809 |
| C13orf15 | -3.033978155 | 11.55164516 | -6.757812809 | 3.54E-07 | 1.35E-05 | 6.648579986 |
| ENTPD1 | -2.813581805 | 5.331097336 | -6.757207978 | 3.55E-07 | 1.35E-05 | 6.647098075 |
| EN2 | 2.694048923 | 10.64678928 | 6.73828066 | 3.72E-07 | 1.41E-05 | 6.600702162 |
| MYO9B | 1.64079023 | 9.84952404 | 6.73078929 | 3.79E-07 | 1.43E-05 | 6.582327273 |
| PHF8 | 2.058309302 | 8.855599466 | 6.718227799 | 3.91E-07 | 1.48E-05 | 6.551501722 |
| PRKCH | -2.457169262 | 8.552405216 | -6.70773631 | 4.02E-07 | 1.51E-05 | 6.525741888 |
| STAU1 | -1.875309911 | 8.815483378 | -6.69829927 | 4.12E-07 | 1.54E-05 | 6.502560192 |
| SPIRE1 | -2.452945982 | 5.35018934 | -6.695188126 | 4.15E-07 | 1.55E-05 | 6.494915545 |
| ZNF385A | 2.42936901 | 10.13173509 | 6.692401226 | 4.18E-07 | 1.56E-05 | 6.488066682 |
| CASP8 | 3.186957596 | 8.998924216 | 6.691137828 | 4.19E-07 | 1.56E-05 | 6.48496156 |
| NOL11 | -2.373528085 | 5.494381088 | -6.686432389 | 4.24E-07 | 1.57E-05 | 6.473395135 |
| SNORD22 | 2.623450379 | 8.501304391 | 6.681325887 | 4.29E-07 | 1.59E-05 | 6.460839986 |
| RAE1 | -2.08127672 | 5.386739269 | -6.677034789 | 4.34E-07 | 1.60E-05 | 6.450287326 |
| TMED10 | -1.962486148 | 9.251095412 | -6.673149477 | 4.38E-07 | 1.61E-05 | 6.440730756 |
| HAT1 | -2.151891788 | 7.733492334 | -6.663583766 | 4.49E-07 | 1.65E-05 | 6.417194953 |
| GIPR | 2.528207505 | 14.18984121 | 6.64778674 | 4.67E-07 | 1.71E-05 | 6.378304586 |
| CLIC5 | -3.98264613 | 9.353151671 | -6.644187355 | 4.71E-07 | 1.71E-05 | 6.369439371 |
| RPLP1 | 1.335629664 | 15.66276819 | 6.643410369 | 4.72E-07 | 1.71E-05 | 6.367525477 |
| CDCA5 | 3.14141387 | 9.050067142 | 6.642558406 | 4.73E-07 | 1.71E-05 | 6.365426819 |
| PKDCC | -2.173855907 | 5.492656796 | -6.640301594 | 4.76E-07 | 1.71E-05 | 6.359867169 |
| PTPN13 | -3.04931234 | 7.62780686 | -6.640295172 | 4.76E-07 | 1.71E-05 | 6.359851348 |
| CTNNA1 | -2.034660376 | 9.542295698 | -6.639775134 | 4.77E-07 | 1.71E-05 | 6.358570153 |
| DAD1 | -2.103707302 | 10.8646447 | -6.637531494 | 4.79E-07 | 1.72E-05 | 6.353042251 |
| CDC6 | 2.457723576 | 7.233463552 | 6.635653177 | 4.82E-07 | 1.72E-05 | 6.348413993 |
| RPL36A | 1.71442455 | 14.10556943 | 6.634472094 | 4.83E-07 | 1.72E-05 | 6.345503548 |
| TMEM14B | -2.393814341 | 7.886988903 | -6.63380925 | 4.84E-07 | 1.72E-05 | 6.343870088 |
| CD2BP2 | -2.005032019 | 5.275688731 | -6.630537935 | 4.88E-07 | 1.73E-05 | 6.335807791 |
| HLA-DRB5 | -2.516660462 | 9.655762862 | -6.629412979 | 4.89E-07 | 1.73E-05 | 6.333035011 |
| TAX1BP1 | -2.058532918 | 10.58598877 | -6.627640442 | 4.92E-07 | 1.73E-05 | 6.328665787 |
| RPS19BP1 | -2.17101331 | 6.533113572 | -6.613955341 | 5.09E-07 | 1.79E-05 | 6.294920724 |
| AGER | -3.698381167 | 12.10577788 | -6.613521645 | 5.09E-07 | 1.79E-05 | 6.293850963 |
| C12orf47 | -2.426209347 | 5.654194585 | -6.608038667 | 5.16E-07 | 1.80E-05 | 6.280324729 |
| ADARB1 | -3.291663494 | 7.945226849 | -6.607945941 | 5.17E-07 | 1.80E-05 | 6.280095952 |
| NUCB1 | 2.303807565 | 8.889904941 | 6.60023107 | 5.27E-07 | 1.83E-05 | 6.261057968 |
| TBC1D2 | -2.228031957 | 5.973470712 | -6.599017839 | 5.28E-07 | 1.83E-05 | 6.258063473 |
| LOXL3 | 2.00879591 | 10.02651414 | 6.595683099 | 5.33E-07 | 1.84E-05 | 6.249831826 |
| NDUFB6 | -2.100251834 | 8.316925499 | -6.594685997 | 5.34E-07 | 1.84E-05 | 6.247370284 |
| TRAPPC8 | -3.089349831 | 6.447263835 | -6.590062441 | 5.40E-07 | 1.86E-05 | 6.235954689 |
| C4orf21 | 1.923071049 | 7.611018994 | 6.589611335 | 5.41E-07 | 1.86E-05 | 6.234840777 |
| MYL12A | -2.666705062 | 11.99762746 | -6.58617501 | 5.46E-07 | 1.87E-05 | 6.226354749 |
| ODC1 | -2.139795196 | 7.866716438 | -6.585223421 | 5.47E-07 | 1.87E-05 | 6.224004563 |
| BBS1 | -2.319461616 | 5.711516866 | -6.581472157 | 5.52E-07 | 1.88E-05 | 6.214738896 |
| GSG2 | 2.47208085 | 7.081445507 | 6.580859064 | 5.53E-07 | 1.88E-05 | 6.213224398 |
| VASH2 | 2.48007094 | 8.113901153 | 6.576317891 | 5.59E-07 | 1.89E-05 | 6.202005248 |
| THBD | -3.457690799 | 10.83209614 | -6.568650935 | 5.70E-07 | 1.93E-05 | 6.183058533 |
| SERF2 | 1.990456456 | 13.63636474 | 6.56484461 | 5.76E-07 | 1.94E-05 | 6.173649864 |
| HIATL1 | -2.982100749 | 5.973246787 | -6.56364675 | 5.78E-07 | 1.94E-05 | 6.170688599 |
| RWDD1 | -2.107166844 | 8.620925402 | -6.557978979 | 5.86E-07 | 1.96E-05 | 6.156674997 |
| TRAM1 | -2.686045819 | 10.06447243 | -6.555603776 | 5.89E-07 | 1.97E-05 | 6.150801239 |
| HDDC2 | -2.43786405 | 6.393738336 | -6.553154698 | 5.93E-07 | 1.98E-05 | 6.14474414 |
| UXT | -2.272638398 | 8.932375839 | -6.552006894 | 5.95E-07 | 1.98E-05 | 6.141905145 |
| HIST2H3A | 2.208448634 | 7.684014347 | 6.549032938 | 5.99E-07 | 1.99E-05 | 6.134548649 |
| SMO | 2.355203228 | 8.523626956 | 6.548340832 | 6.00E-07 | 1.99E-05 | 6.132836489 |
| ZNF516 | 2.139731846 | 8.703203819 | 6.536487741 | 6.19E-07 | 2.04E-05 | 6.103505664 |
| SP140 | 2.640656291 | 9.144144009 | 6.529787731 | 6.29E-07 | 2.07E-05 | 6.086919491 |
| PGCP | -2.608156831 | 7.214724691 | -6.525484706 | 6.36E-07 | 2.08E-05 | 6.076264579 |
| DDX11 | 2.473660666 | 8.502767966 | 6.525291082 | 6.36E-07 | 2.08E-05 | 6.07578509 |
| GPS1 | -2.448987887 | 5.47127058 | -6.523627914 | 6.39E-07 | 2.08E-05 | 6.071666267 |
| ZNF595 | -1.929144599 | 5.69789973 | -6.523430022 | 6.39E-07 | 2.08E-05 | 6.07117617 |
| USP53 | -2.057518272 | 7.62077225 | -6.515297487 | 6.53E-07 | 2.12E-05 | 6.051031512 |
| ADHFE1 | -3.371229481 | 5.316223884 | -6.513777429 | 6.55E-07 | 2.12E-05 | 6.047265462 |
| SGOL1 | 2.885024287 | 6.990592141 | 6.512955277 | 6.57E-07 | 2.12E-05 | 6.045228419 |
| WRB | -2.358879668 | 5.456419907 | -6.505407438 | 6.69E-07 | 2.16E-05 | 6.026523739 |
| UPB1 | 3.443532417 | 11.80237276 | 6.504236424 | 6.71E-07 | 2.16E-05 | 6.023621236 |
| CAMK2N2 | -4.083109308 | 7.545993004 | -6.497468568 | 6.83E-07 | 2.19E-05 | 6.006843375 |
| NEUROG1 | 3.262598664 | 9.967160914 | 6.495905484 | 6.86E-07 | 2.19E-05 | 6.002967709 |
| TULP3 | 2.335654187 | 9.463635643 | 6.495220059 | 6.87E-07 | 2.19E-05 | 6.001268113 |
| SLC39A8 | -2.625847699 | 10.96849404 | -6.493078323 | 6.91E-07 | 2.20E-05 | 5.995957092 |
| RAB4A | -1.639928788 | 5.551964361 | -6.489683674 | 6.96E-07 | 2.21E-05 | 5.987538117 |
| RCC1 | 2.159229075 | 7.515458062 | 6.485547411 | 7.04E-07 | 2.22E-05 | 5.977278211 |
| FLRT3 | -3.215973105 | 7.86981792 | -6.485427806 | 7.04E-07 | 2.22E-05 | 5.976981506 |
| ZNF641 | -2.418945727 | 5.321788958 | -6.484458137 | 7.06E-07 | 2.22E-05 | 5.974575986 |
| PC | 2.091889109 | 8.672356355 | 6.484442324 | 7.06E-07 | 2.22E-05 | 5.974536758 |
| RHBDL1 | 3.380106752 | 9.848827988 | 6.484095432 | 7.06E-07 | 2.22E-05 | 5.973676174 |
| SUN1 | -1.867576945 | 7.796616223 | -6.482867998 | 7.09E-07 | 2.22E-05 | 5.970631009 |
| GBAS | -2.817910863 | 5.22990637 | -6.481462508 | 7.11E-07 | 2.22E-05 | 5.9671439 |
| CD70 | 3.607207488 | 8.127039619 | 6.480174512 | 7.13E-07 | 2.22E-05 | 5.963948117 |
| FKBP1B | -2.504178694 | 7.100642752 | -6.476287889 | 7.21E-07 | 2.23E-05 | 5.954303523 |
| ARHGEF26 | -2.464971114 | 5.841586536 | -6.476124452 | 7.21E-07 | 2.23E-05 | 5.953897923 |
| PIGH | -2.352940166 | 5.490631537 | -6.475614987 | 7.22E-07 | 2.23E-05 | 5.952633564 |
| KLHL7 | -2.249605667 | 5.13874228 | -6.470514115 | 7.31E-07 | 2.26E-05 | 5.939973015 |
| RAF1 | -1.648985734 | 6.555847927 | -6.466689434 | 7.38E-07 | 2.27E-05 | 5.930478195 |
| C3 | -2.406889863 | 9.845287999 | -6.453118044 | 7.64E-07 | 2.35E-05 | 5.896774472 |
| TRIM44 | -1.582670005 | 7.478146848 | -6.448023561 | 7.74E-07 | 2.37E-05 | 5.884117583 |
| CD302 | -3.395914832 | 8.211388638 | -6.445062183 | 7.80E-07 | 2.38E-05 | 5.876758984 |
| KLK1 | -1.787181133 | 6.034719425 | -6.442251017 | 7.86E-07 | 2.39E-05 | 5.869772781 |
| ACAT1 | -2.802522519 | 8.046931936 | -6.440648062 | 7.89E-07 | 2.40E-05 | 5.865788802 |
| C1orf229 | 3.273109243 | 9.177498779 | 6.437149096 | 7.96E-07 | 2.41E-05 | 5.857091548 |
| TACSTD2 | -4.275477734 | 8.954476031 | -6.429555174 | 8.11E-07 | 2.46E-05 | 5.83821117 |
| MUC4 | 3.231537318 | 10.37759784 | 6.427381262 | 8.16E-07 | 2.46E-05 | 5.832805167 |
| RPS2 | 2.035697292 | 12.94471266 | 6.425815279 | 8.19E-07 | 2.47E-05 | 5.828910632 |
| FRY | -2.747883101 | 8.665443541 | -6.425036314 | 8.21E-07 | 2.47E-05 | 5.826973281 |
| EDF1 | -1.864622192 | 10.4237573 | -6.422063609 | 8.27E-07 | 2.48E-05 | 5.819579336 |
| C20orf96 | 2.24031102 | 8.611323356 | 6.421363415 | 8.28E-07 | 2.48E-05 | 5.817837624 |
| CPT1C | 2.154397194 | 8.787944588 | 6.418604892 | 8.34E-07 | 2.49E-05 | 5.810975377 |
| MDH2 | -2.124684079 | 8.254065737 | -6.416632449 | 8.38E-07 | 2.49E-05 | 5.806068138 |
| RARRES2 | -1.78857985 | 8.605043304 | -6.4149567 | 8.42E-07 | 2.49E-05 | 5.801898722 |
| GAS5 | -2.680888526 | 7.776411458 | -6.41483229 | 8.42E-07 | 2.49E-05 | 5.801589167 |
| NHP2L1 | -1.851757601 | 7.730306353 | -6.414348519 | 8.43E-07 | 2.49E-05 | 5.800385441 |
| LEMD2 | 1.37491403 | 10.17403447 | 6.413410962 | 8.45E-07 | 2.49E-05 | 5.798052528 |
| MFAP1 | -1.773595765 | 7.020967797 | -6.413121679 | 8.46E-07 | 2.49E-05 | 5.797332688 |
| MANBAL | -2.677700578 | 5.640543523 | -6.412792463 | 8.47E-07 | 2.49E-05 | 5.796513471 |
| CDC23 | -3.206469454 | 5.81227764 | -6.398576835 | 8.78E-07 | 2.57E-05 | 5.761128718 |
| UTS2R | 2.489809415 | 10.34119271 | 6.397379832 | 8.80E-07 | 2.57E-05 | 5.758148245 |
| C14orf132 | -2.807128889 | 7.211344454 | -6.39055885 | 8.96E-07 | 2.61E-05 | 5.741161533 |
| DLL1 | -2.25105618 | 6.63186423 | -6.382943336 | 9.13E-07 | 2.66E-05 | 5.722190463 |
| BPNT1 | -2.435400565 | 5.124403646 | -6.381915806 | 9.16E-07 | 2.66E-05 | 5.719630318 |
| AKR1B1 | -2.187512697 | 6.891780982 | -6.381304937 | 9.17E-07 | 2.66E-05 | 5.718108252 |
| FAM63B | -1.441455319 | 7.630336184 | -6.377991264 | 9.25E-07 | 2.67E-05 | 5.709851106 |
| MRPS23 | -2.348061639 | 5.83248279 | -6.377464357 | 9.26E-07 | 2.67E-05 | 5.708538034 |
| HIST1H2AG | 2.00278957 | 10.4793516 | 6.370692352 | 9.42E-07 | 2.71E-05 | 5.691659396 |
| ZNF182 | 1.609871135 | 8.132541985 | 6.367971845 | 9.49E-07 | 2.72E-05 | 5.684877441 |
| NCOA6 | -2.600039371 | 6.605663959 | -6.365983912 | 9.54E-07 | 2.73E-05 | 5.679921243 |
| PTTG1IP | -1.798277805 | 9.877832912 | -6.364815432 | 9.56E-07 | 2.73E-05 | 5.677007866 |
| H3F3C | -2.691784762 | 13.47842335 | -6.363985928 | 9.58E-07 | 2.73E-05 | 5.674939576 |
| CDKL3 | -1.606240608 | 4.955819394 | -6.363095324 | 9.61E-07 | 2.73E-05 | 5.672718861 |
| HDHD1 | -2.677937854 | 5.288150278 | -6.35809089 | 9.73E-07 | 2.76E-05 | 5.660238826 |
| C5orf51 | -1.719913056 | 7.078995422 | -6.357589509 | 9.74E-07 | 2.76E-05 | 5.658988344 |
| MTX1 | -2.579026996 | 5.169649659 | -6.356586304 | 9.77E-07 | 2.76E-05 | 5.656486198 |
| RPS3 | -1.861765622 | 10.34289592 | -6.347761402 | 9.99E-07 | 2.82E-05 | 5.634471122 |
| CETN3 | -1.807640625 | 6.656875206 | -6.346816049 | 1.00E-06 | 2.82E-05 | 5.632112325 |
| HIST1H2AH | 2.198848372 | 10.14461251 | 6.343330467 | 1.01E-06 | 2.84E-05 | 5.62341449 |
| DKFZP586I1420 | -2.146099644 | 5.89314511 | -6.34152327 | 1.01E-06 | 2.84E-05 | 5.618904369 |
| ARHGAP31 | -2.165580649 | 5.981841557 | -6.339698933 | 1.02E-06 | 2.85E-05 | 5.614351139 |
| ST6GAL1 | 2.512613533 | 8.40516777 | 6.338687179 | 1.02E-06 | 2.85E-05 | 5.611825829 |
| VPS11 | -1.575040464 | 5.695116482 | -6.33744648 | 1.03E-06 | 2.86E-05 | 5.608728938 |
| KIAA0494 | -2.559846555 | 9.012058256 | -6.332896009 | 1.04E-06 | 2.88E-05 | 5.597369243 |
| COASY | -2.029883531 | 5.92754512 | -6.331812032 | 1.04E-06 | 2.88E-05 | 5.59466292 |
| ECH1 | -2.479182409 | 5.441358825 | -6.326846282 | 1.05E-06 | 2.91E-05 | 5.582263604 |
| LOC100132287 | 2.310933414 | 8.97751795 | 6.325133064 | 1.06E-06 | 2.92E-05 | 5.577985177 |
| ANKRD20A9P | -3.122988469 | 5.929892475 | -6.319484666 | 1.07E-06 | 2.96E-05 | 5.563877323 |
| GOLT1B | -2.853901673 | 5.106979341 | -6.318281243 | 1.08E-06 | 2.96E-05 | 5.56087115 |
| NSMCE4A | -1.87851179 | 7.752843455 | -6.317526463 | 1.08E-06 | 2.96E-05 | 5.558985623 |
| RPL18A | -2.24795072 | 10.92752167 | -6.315925757 | 1.08E-06 | 2.96E-05 | 5.554986686 |
| GOLM1 | 2.180676881 | 7.934770235 | 6.315890916 | 1.08E-06 | 2.96E-05 | 5.554899641 |
| PEX12 | -1.944059419 | 5.470433707 | -6.312549337 | 1.09E-06 | 2.98E-05 | 5.546550757 |
| SYNJ2BP | -1.798796321 | 7.001964294 | -6.309699456 | 1.10E-06 | 2.99E-05 | 5.539429489 |
| CCDC121 | -2.29526678 | 5.131082552 | -6.308596272 | 1.10E-06 | 2.99E-05 | 5.536672641 |
| CCT3 | -1.919976731 | 8.62964258 | -6.308310519 | 1.10E-06 | 2.99E-05 | 5.535958528 |
| ADPGK | -1.601422184 | 7.097956596 | -6.305602123 | 1.11E-06 | 3.00E-05 | 5.529189675 |
| BEST1 | 2.46897226 | 9.492119497 | 6.305300794 | 1.11E-06 | 3.00E-05 | 5.528436547 |
| SLC4A1AP | -1.884614376 | 7.415910012 | -6.30005667 | 1.13E-06 | 3.03E-05 | 5.51532814 |
| ACBD7 | 2.237443147 | 7.524748943 | 6.299433168 | 1.13E-06 | 3.03E-05 | 5.51376943 |
| REXO4 | 1.502268801 | 7.321815368 | 6.29851952 | 1.13E-06 | 3.03E-05 | 5.511485307 |
| VAMP3 | -1.642714486 | 8.177757781 | -6.296801344 | 1.14E-06 | 3.04E-05 | 5.507189631 |
| RAB5A | -1.953823224 | 7.515175713 | -6.295830131 | 1.14E-06 | 3.04E-05 | 5.504761338 |
| GBP6 | 2.226951807 | 11.82616462 | 6.295507828 | 1.14E-06 | 3.04E-05 | 5.503955473 |
| HEG1 | -2.887446931 | 9.178967813 | -6.294066487 | 1.15E-06 | 3.04E-05 | 5.500351514 |
| HIGD1B | -3.041908414 | 7.201062502 | -6.293733278 | 1.15E-06 | 3.04E-05 | 5.499518322 |
| C19orf28 | 1.562100649 | 9.455512608 | 6.290313825 | 1.16E-06 | 3.06E-05 | 5.490967313 |
| KHDRBS3 | -2.661468008 | 5.853090358 | -6.290112469 | 1.16E-06 | 3.06E-05 | 5.490463746 |
| DGKZ | 2.298348661 | 7.624849222 | 6.284043665 | 1.18E-06 | 3.10E-05 | 5.475284547 |
| NEK6 | 2.575886014 | 10.63962263 | 6.282623166 | 1.18E-06 | 3.10E-05 | 5.471731094 |
| C20orf141 | 3.327614574 | 9.618176684 | 6.278876433 | 1.19E-06 | 3.13E-05 | 5.462357483 |
| CGRRF1 | -1.736359863 | 5.681690475 | -6.277610328 | 1.19E-06 | 3.13E-05 | 5.459189616 |
| MUC1 | -3.597102907 | 9.071183282 | -6.26844678 | 1.22E-06 | 3.20E-05 | 5.436257212 |
| EBF4 | 1.305260043 | 9.875766516 | 6.266386753 | 1.23E-06 | 3.21E-05 | 5.431100719 |
| LEMD3 | -1.855563835 | 7.429816139 | -6.257111562 | 1.26E-06 | 3.28E-05 | 5.407878675 |
| GIGYF1 | 1.69829099 | 8.719836819 | 6.254669855 | 1.27E-06 | 3.29E-05 | 5.401764046 |
| TGFBR3 | -2.168927368 | 6.527552556 | -6.251607245 | 1.28E-06 | 3.31E-05 | 5.394093701 |
| PDLIM4 | 2.560864089 | 10.55973258 | 6.250333185 | 1.28E-06 | 3.32E-05 | 5.390902533 |
| BRP44 | -2.034493613 | 8.094929582 | -6.24836745 | 1.29E-06 | 3.33E-05 | 5.385978603 |
| GNPTG | -1.938551952 | 8.486702416 | -6.242350713 | 1.31E-06 | 3.37E-05 | 5.370905071 |
| HLA-DRB4 | -1.999357171 | 8.89269654 | -6.241866743 | 1.31E-06 | 3.37E-05 | 5.369692444 |
| MTA1 | 1.61923074 | 10.29658507 | 6.241055643 | 1.31E-06 | 3.37E-05 | 5.367660115 |
| CD58 | -1.96945457 | 6.539896228 | -6.238989925 | 1.32E-06 | 3.38E-05 | 5.362483874 |
| LOC100506748 | -3.12090234 | 5.701778649 | -6.237341527 | 1.32E-06 | 3.39E-05 | 5.358353048 |
| CYB5A | -2.891351179 | 11.61699515 | -6.2334327 | 1.34E-06 | 3.41E-05 | 5.348556625 |
| TRAPPC2P1 | -2.17290866 | 5.709862195 | -6.230926385 | 1.35E-06 | 3.43E-05 | 5.342274448 |
| GJC1 | 2.136066831 | 8.244695029 | 6.227617973 | 1.36E-06 | 3.45E-05 | 5.333980856 |
| NARS2 | -2.079395078 | 5.322109781 | -6.225894833 | 1.36E-06 | 3.46E-05 | 5.32966084 |
| LIMS2 | -2.144258614 | 7.633805079 | -6.221689135 | 1.38E-06 | 3.48E-05 | 5.319115702 |
| ZNHIT3 | -1.636149659 | 8.93221339 | -6.22163381 | 1.38E-06 | 3.48E-05 | 5.318976973 |
| C17orf49 | -2.115178438 | 7.719991184 | -6.217536396 | 1.39E-06 | 3.51E-05 | 5.308701689 |
| C6orf130 | -1.616822146 | 8.283951113 | -6.216253513 | 1.40E-06 | 3.52E-05 | 5.305484213 |
| AP2S1 | -2.662535781 | 7.131779315 | -6.210886355 | 1.42E-06 | 3.56E-05 | 5.292021649 |
| FBXO28 | -2.247743869 | 5.438961152 | -6.208402371 | 1.43E-06 | 3.58E-05 | 5.285790087 |
| RHOT2 | -1.960528311 | 5.671334243 | -6.202374882 | 1.45E-06 | 3.62E-05 | 5.270666509 |
| ZNF664 | -1.741734038 | 7.851894366 | -6.201374014 | 1.45E-06 | 3.63E-05 | 5.268154898 |
| CPNE5 | 2.51689013 | 9.914602566 | 6.197289459 | 1.47E-06 | 3.65E-05 | 5.257903993 |
| C10orf116 | -2.587123152 | 11.2350517 | -6.196964224 | 1.47E-06 | 3.65E-05 | 5.257087691 |
| ABI2 | -2.426517542 | 6.11779074 | -6.196118282 | 1.47E-06 | 3.65E-05 | 5.254964428 |
| SELS | -2.618926145 | 10.05854263 | -6.191205491 | 1.49E-06 | 3.69E-05 | 5.242632287 |
| RNF19A | -2.013876427 | 6.75543845 | -6.190327794 | 1.49E-06 | 3.69E-05 | 5.240428844 |
| RETNLB | 3.118956151 | 9.500944182 | 6.190124354 | 1.49E-06 | 3.69E-05 | 5.239918101 |
| POLR3B | -2.568017232 | 4.484795512 | -6.180013493 | 1.53E-06 | 3.78E-05 | 5.21452953 |
| KIFAP3 | -2.181739274 | 8.309329745 | -6.178572027 | 1.54E-06 | 3.78E-05 | 5.210909201 |
| UGGT1 | 1.583386442 | 8.506880772 | 6.178145521 | 1.54E-06 | 3.78E-05 | 5.209837966 |
| MGC45922 | 3.006698372 | 9.187540908 | 6.176545719 | 1.55E-06 | 3.79E-05 | 5.205819673 |
| SNRPN | -2.243911559 | 6.229660041 | -6.171679644 | 1.57E-06 | 3.83E-05 | 5.193595872 |
| ABHD10 | -1.973200667 | 6.341915699 | -6.170481513 | 1.57E-06 | 3.83E-05 | 5.190585771 |
| FAM54B | -1.488342484 | 6.123943533 | -6.170309655 | 1.57E-06 | 3.83E-05 | 5.190153996 |
| AIMP1 | -1.687345386 | 7.151082285 | -6.168513448 | 1.58E-06 | 3.84E-05 | 5.185641054 |
| GALR3 | 2.7990148 | 10.07460181 | 6.16552459 | 1.59E-06 | 3.86E-05 | 5.17813093 |
| SPINT1 | -1.826869733 | 6.657108479 | -6.161662999 | 1.61E-06 | 3.89E-05 | 5.168426655 |
| ZNF22 | -1.976837465 | 7.554103128 | -6.155882283 | 1.63E-06 | 3.95E-05 | 5.153896984 |
| LOC100128843 | 3.227299408 | 10.19600558 | 6.151819309 | 1.65E-06 | 3.98E-05 | 5.143682963 |
| GPR135 | 1.672674245 | 7.882909739 | 6.149140555 | 1.66E-06 | 4.00E-05 | 5.136947936 |
| FBXW5 | -1.806978728 | 6.575318914 | -6.147332444 | 1.67E-06 | 4.01E-05 | 5.132401541 |
| MGC39584 | 5.267472822 | 8.071165492 | 6.144269873 | 1.68E-06 | 4.02E-05 | 5.124700186 |
| TPPP3 | -3.186283477 | 6.937041772 | -6.143514565 | 1.68E-06 | 4.02E-05 | 5.122800703 |
| KIF2C | 1.754078621 | 7.231124629 | 6.143441343 | 1.68E-06 | 4.02E-05 | 5.122616559 |
| ZNF496 | 2.81262533 | 10.66083502 | 6.143354569 | 1.69E-06 | 4.02E-05 | 5.122398333 |
| ACOT7 | -2.650983038 | 4.821771828 | -6.13962611 | 1.70E-06 | 4.05E-05 | 5.113021015 |
| ZFP91 | -2.374912653 | 7.114447972 | -6.13957411 | 1.70E-06 | 4.05E-05 | 5.112890224 |
| ZNF728 | -2.974441452 | 5.704869603 | -6.138427053 | 1.71E-06 | 4.05E-05 | 5.110005037 |
| KRT81 | 3.748622891 | 10.79384963 | 6.134625378 | 1.72E-06 | 4.08E-05 | 5.100441849 |
| EMP3 | -2.155770522 | 7.927758752 | -6.13441254 | 1.72E-06 | 4.08E-05 | 5.099906412 |
| ANXA2 | -3.10533233 | 11.76007263 | -6.124992577 | 1.77E-06 | 4.17E-05 | 5.076204441 |
| ASF1B | 1.740196634 | 7.006470369 | 6.123669911 | 1.77E-06 | 4.18E-05 | 5.072875779 |
| A2ML1 | 2.931331655 | 11.60993195 | 6.115378087 | 1.81E-06 | 4.26E-05 | 5.0520047 |
| GLT8D1 | -2.323955348 | 7.657405378 | -6.111191975 | 1.83E-06 | 4.28E-05 | 5.041465614 |
| DDRGK1 | -1.954712466 | 7.5912829 | -6.111103204 | 1.83E-06 | 4.28E-05 | 5.041242104 |
| KLHL25 | 1.593959955 | 9.674501905 | 6.110568447 | 1.83E-06 | 4.28E-05 | 5.039895664 |
| SART1 | -1.599626191 | 6.999633498 | -6.110497264 | 1.83E-06 | 4.28E-05 | 5.039716436 |
| IFT57 | -2.484192614 | 9.207202403 | -6.108385441 | 1.84E-06 | 4.30E-05 | 5.03439891 |
| CTTNBP2 | -2.613708495 | 4.492079076 | -6.101269601 | 1.88E-06 | 4.37E-05 | 5.016478429 |
| PAN3 | -2.31337077 | 5.776094716 | -6.098607622 | 1.89E-06 | 4.39E-05 | 5.009773354 |
| KIAA0408 | -2.829713379 | 6.575023386 | -6.093547165 | 1.92E-06 | 4.44E-05 | 4.997025171 |
| CCNB2 | 2.951696476 | 8.233518385 | 6.092908345 | 1.92E-06 | 4.44E-05 | 4.995415707 |
| HGS | 1.189676924 | 11.25739755 | 6.090020795 | 1.93E-06 | 4.46E-05 | 4.988140269 |
| PSMD9 | -1.983403133 | 8.224721403 | -6.089461623 | 1.94E-06 | 4.46E-05 | 4.9867313 |
| ATP6V1G1 | -2.292905871 | 11.00785922 | -6.08733177 | 1.95E-06 | 4.47E-05 | 4.981364367 |
| C7orf36 | -2.129585577 | 5.52210141 | -6.087132595 | 1.95E-06 | 4.47E-05 | 4.980862454 |
| DDX41 | -1.908002557 | 5.553328358 | -6.084412642 | 1.96E-06 | 4.49E-05 | 4.974007915 |
| AOC3 | -3.187001902 | 10.60891539 | -6.083907293 | 1.96E-06 | 4.49E-05 | 4.972734316 |
| BHLHE22 | 2.063988334 | 8.035954418 | 6.0820951 | 1.97E-06 | 4.51E-05 | 4.968166973 |
| PRPSAP2 | -2.285929881 | 5.93143664 | -6.07922805 | 1.99E-06 | 4.53E-05 | 4.960940436 |
| HEXB | -2.519433656 | 8.699278046 | -6.077480403 | 2.00E-06 | 4.54E-05 | 4.956535053 |
| BSCL2 | -2.059500951 | 5.128746297 | -6.073060062 | 2.02E-06 | 4.59E-05 | 4.945391267 |
| LAPTM4B | -2.015713161 | 8.523519457 | -6.071714778 | 2.03E-06 | 4.60E-05 | 4.941999433 |
| FFAR2 | 3.287051837 | 10.34191616 | 6.070784755 | 2.03E-06 | 4.60E-05 | 4.939654494 |
| C17orf42 | -1.695550572 | 5.746768321 | -6.063539692 | 2.07E-06 | 4.68E-05 | 4.921384347 |
| VGF | 2.980013961 | 8.858306844 | 6.061120306 | 2.08E-06 | 4.70E-05 | 4.915282267 |
| SEC23IP | -1.701502232 | 6.457550338 | -6.054110835 | 2.12E-06 | 4.78E-05 | 4.897600365 |
| KLF14 | 3.175079483 | 10.38529644 | 6.04821295 | 2.15E-06 | 4.84E-05 | 4.882719204 |
| PNRC2 | -2.471306978 | 8.146752373 | -6.046032881 | 2.16E-06 | 4.86E-05 | 4.877217833 |
| NKX2-8 | 3.094701357 | 7.643099225 | 6.044420302 | 2.17E-06 | 4.87E-05 | 4.873148248 |
| PNMT | -3.608055122 | 5.498698239 | -6.040235976 | 2.20E-06 | 4.92E-05 | 4.862587425 |
| SFN | 2.284237752 | 7.642157988 | 6.037277588 | 2.21E-06 | 4.94E-05 | 4.855119837 |
| TCF20 | 2.144935271 | 8.89006227 | 6.033629432 | 2.24E-06 | 4.97E-05 | 4.84591009 |
| TXNDC9 | -1.552344489 | 6.840096994 | -6.033535179 | 2.24E-06 | 4.97E-05 | 4.845672135 |
| TPH2 | 2.160553956 | 5.886347583 | 6.033126063 | 2.24E-06 | 4.97E-05 | 4.84463925 |
| PIBF1 | -1.48072136 | 5.95988203 | -6.031930606 | 2.25E-06 | 4.98E-05 | 4.841621027 |
| FABP5 | -2.239631411 | 7.524351865 | -6.031120126 | 2.25E-06 | 4.98E-05 | 4.8395747 |
| TRIM4 | -1.406578885 | 8.335802117 | -6.026635428 | 2.28E-06 | 5.03E-05 | 4.828250578 |
| UBE2D2 | -1.632650444 | 8.517409429 | -6.024786143 | 2.29E-06 | 5.05E-05 | 4.823580526 |
| DDX42 | -1.88649997 | 7.039245265 | -6.018577485 | 2.32E-06 | 5.12E-05 | 4.807899487 |
| TSPAN7 | -2.4146478 | 5.375111806 | -6.01364585 | 2.35E-06 | 5.17E-05 | 4.795441451 |
| IRX2 | -2.640124384 | 9.286769447 | -6.012708986 | 2.36E-06 | 5.18E-05 | 4.793074562 |
| LOC100190939 | 2.073469066 | 9.438610727 | 6.011365977 | 2.37E-06 | 5.19E-05 | 4.789681461 |
| DNMBP | -2.248442827 | 5.829310557 | -6.006703492 | 2.40E-06 | 5.24E-05 | 4.777900541 |
| RBX1 | -2.39930025 | 8.256423602 | -6.004182786 | 2.41E-06 | 5.27E-05 | 4.771530591 |
| KCND3 | 2.132212261 | 8.725667358 | 6.002198361 | 2.42E-06 | 5.28E-05 | 4.766515474 |
| SEC11A | -1.78253845 | 11.11942956 | -6.000505089 | 2.43E-06 | 5.30E-05 | 4.762235906 |
| DCAF6 | -2.511136763 | 5.753463012 | -5.997798943 | 2.45E-06 | 5.33E-05 | 4.755395906 |
| COX5A | -2.497446871 | 7.002339527 | -5.990752753 | 2.50E-06 | 5.42E-05 | 4.737583219 |
| CDK11B | 1.79637132 | 11.10959099 | 5.98946663 | 2.51E-06 | 5.42E-05 | 4.734331468 |
| SEC63 | -1.692295426 | 8.076112691 | -5.987780233 | 2.52E-06 | 5.44E-05 | 4.730067478 |
| C1orf96 | 1.470712474 | 7.828004932 | 5.987121577 | 2.52E-06 | 5.44E-05 | 4.72840203 |
| ATP5J | -2.131812776 | 10.01125015 | -5.985297378 | 2.53E-06 | 5.46E-05 | 4.723789245 |
| DHX29 | -1.541159728 | 6.439194728 | -5.982925317 | 2.55E-06 | 5.47E-05 | 4.717790693 |
| HIST3H3 | -2.372528552 | 10.81158753 | -5.982840035 | 2.55E-06 | 5.47E-05 | 4.717575019 |
| SYNGAP1 | 1.676565256 | 6.381262055 | 5.981569333 | 2.56E-06 | 5.48E-05 | 4.71436142 |
| CACNA2D2 | -4.079143261 | 8.521009126 | -5.981337165 | 2.56E-06 | 5.48E-05 | 4.713774254 |
| ZDHHC8 | 2.748185986 | 8.577957082 | 5.98041528 | 2.56E-06 | 5.48E-05 | 4.711442709 |
| HYAL2 | -2.426856087 | 8.587551265 | -5.979725645 | 2.57E-06 | 5.48E-05 | 4.709698504 |
| KLHL20 | -2.004663651 | 6.225145181 | -5.979364576 | 2.57E-06 | 5.48E-05 | 4.708785282 |
| MED16 | -1.704043708 | 5.16375827 | -5.976800482 | 2.59E-06 | 5.50E-05 | 4.702299826 |
| ELAVL1 | -1.619654294 | 7.662878948 | -5.973727433 | 2.61E-06 | 5.53E-05 | 4.694526333 |
| ITPKA | 2.801921475 | 7.975201479 | 5.97265405 | 2.62E-06 | 5.53E-05 | 4.691810952 |
| PRPF8 | -2.083071133 | 7.536068436 | -5.97219525 | 2.62E-06 | 5.53E-05 | 4.69065028 |
| DYNLL1 | -1.889285102 | 11.55123113 | -5.972030755 | 2.62E-06 | 5.53E-05 | 4.690234135 |
| SCAF8 | -1.888602754 | 6.361076707 | -5.966364481 | 2.66E-06 | 5.60E-05 | 4.675898067 |
| BAALC | 3.149772474 | 11.34878466 | 5.966193652 | 2.66E-06 | 5.60E-05 | 4.675465816 |
| PCSK7 | 3.023584967 | 9.020052856 | 5.963461419 | 2.68E-06 | 5.63E-05 | 4.668552094 |
| TMEM85 | -1.595642553 | 9.264532994 | -5.962828654 | 2.68E-06 | 5.63E-05 | 4.666950842 |
| MORF4L1 | -1.875715612 | 9.41188828 | -5.961192671 | 2.70E-06 | 5.65E-05 | 4.662810727 |
| OR5H1 | 3.087121544 | 8.575027867 | 5.959393676 | 2.71E-06 | 5.66E-05 | 4.658257828 |
| YPEL5 | -2.238886665 | 8.629534618 | -5.959019454 | 2.71E-06 | 5.66E-05 | 4.657310715 |
| LTBP4 | -1.531848537 | 7.889603938 | -5.95587787 | 2.73E-06 | 5.70E-05 | 4.649359265 |
| UBL5 | -1.778484868 | 11.26295357 | -5.952945161 | 2.75E-06 | 5.73E-05 | 4.641935757 |
| BBC3 | 2.481601968 | 12.87937977 | 5.950366494 | 2.77E-06 | 5.76E-05 | 4.635407852 |
| FXYD1 | -2.141767237 | 5.494604261 | -5.947070205 | 2.80E-06 | 5.79E-05 | 4.627062498 |
| LRP3 | 2.633205654 | 11.13427274 | 5.94664157 | 2.80E-06 | 5.79E-05 | 4.62597724 |
| RBM47 | -2.109917096 | 6.756964255 | -5.946492058 | 2.80E-06 | 5.79E-05 | 4.625598687 |
| DGUOK | -1.621543899 | 7.612710866 | -5.946072469 | 2.80E-06 | 5.79E-05 | 4.624536313 |
| ALAS2 | 3.976308715 | 9.447565703 | 5.940702144 | 2.84E-06 | 5.86E-05 | 4.610937717 |
| PYCR1 | 2.4010851 | 9.687831785 | 5.93174913 | 2.91E-06 | 5.98E-05 | 4.588261971 |
| RPF2 | -2.304697681 | 6.040997974 | -5.931221854 | 2.91E-06 | 5.98E-05 | 4.586926313 |
| CHRD | 1.517715078 | 7.700189036 | 5.92531681 | 2.96E-06 | 6.07E-05 | 4.571966549 |
| DAPK2 | -3.138123257 | 6.267584194 | -5.917799171 | 3.02E-06 | 6.18E-05 | 4.552917437 |
| FAM89A | -1.71675438 | 7.859608384 | -5.917069117 | 3.02E-06 | 6.18E-05 | 4.551067299 |
| COL6A3 | 1.511974444 | 10.56742399 | 5.916591133 | 3.02E-06 | 6.18E-05 | 4.549855945 |
| SCAF1 | 2.394184345 | 14.13819479 | 5.913754678 | 3.05E-06 | 6.21E-05 | 4.542667154 |
| FARSA | -2.223969571 | 4.947153545 | -5.910754743 | 3.07E-06 | 6.25E-05 | 4.535063344 |
| CDH24 | 2.695589432 | 10.23645961 | 5.906812615 | 3.10E-06 | 6.30E-05 | 4.525070317 |
| LCMT1 | -1.586250717 | 7.642566563 | -5.905076763 | 3.12E-06 | 6.32E-05 | 4.520669663 |
| FAM47C | 2.898164207 | 10.8650034 | 5.899197528 | 3.16E-06 | 6.41E-05 | 4.505763137 |
| CCNG2 | -2.212876065 | 6.390885668 | -5.893811884 | 3.21E-06 | 6.49E-05 | 4.492105721 |
| MAP2K2 | 1.4093436 | 7.395461933 | 5.892753798 | 3.22E-06 | 6.49E-05 | 4.489422262 |
| LYRM1 | -2.854981301 | 5.029335885 | -5.892235856 | 3.22E-06 | 6.49E-05 | 4.488108653 |
| NFATC3 | -2.060821052 | 6.542807 | -5.88999919 | 3.24E-06 | 6.51E-05 | 4.48243577 |
| MMGT1 | -1.47844228 | 9.097936466 | -5.889908005 | 3.24E-06 | 6.51E-05 | 4.482204488 |
| TMSB10 | -2.371134899 | 13.13847513 | -5.885467531 | 3.28E-06 | 6.58E-05 | 4.47094088 |
| ZFP36L1 | 1.804693491 | 12.42417604 | 5.884197118 | 3.29E-06 | 6.58E-05 | 4.467718099 |
| ANKS1A | -2.804821293 | 8.969916047 | -5.883980122 | 3.29E-06 | 6.58E-05 | 4.46716761 |
| DCTPP1 | -1.944681332 | 6.606727134 | -5.88241212 | 3.30E-06 | 6.60E-05 | 4.463189707 |
| EXOSC6 | 1.565813685 | 7.949788662 | 5.878690338 | 3.34E-06 | 6.65E-05 | 4.453747068 |
| TMEM134 | -2.175157025 | 6.798385986 | -5.878013885 | 3.34E-06 | 6.66E-05 | 4.452030705 |
| ADNP | -2.723529965 | 7.191293966 | -5.874081695 | 3.38E-06 | 6.71E-05 | 4.442052873 |
| C19orf70 | -2.21948477 | 8.150299031 | -5.871050823 | 3.40E-06 | 6.75E-05 | 4.434361305 |
| MED17 | -2.30053495 | 5.517788178 | -5.870758277 | 3.41E-06 | 6.75E-05 | 4.433618864 |
| FAF2 | -2.695279639 | 6.03340671 | -5.869453755 | 3.42E-06 | 6.76E-05 | 4.430308079 |
| ARHGEF35 | 1.755177597 | 8.007814104 | 5.866583457 | 3.44E-06 | 6.79E-05 | 4.42302301 |
| DHX15 | -1.775135192 | 8.290604618 | -5.866522901 | 3.44E-06 | 6.79E-05 | 4.422869307 |
| RPL3 | -2.459933381 | 11.21630423 | -5.866274717 | 3.45E-06 | 6.79E-05 | 4.422239364 |
| MRPS15 | -2.069890321 | 7.258988877 | -5.86492354 | 3.46E-06 | 6.80E-05 | 4.41880971 |
| NDUFA4 | -2.330888924 | 11.98103786 | -5.863567151 | 3.47E-06 | 6.81E-05 | 4.415366687 |
| MYL6 | -2.14437197 | 12.02413333 | -5.859779013 | 3.50E-06 | 6.87E-05 | 4.405750232 |
| NFKB1 | -2.207666251 | 7.074594644 | -5.858743036 | 3.51E-06 | 6.88E-05 | 4.403120143 |
| VWCE | 2.670825826 | 8.192402901 | 5.856680475 | 3.53E-06 | 6.91E-05 | 4.39788357 |
| C17orf65 | 2.232624236 | 7.577238876 | 5.855982742 | 3.54E-06 | 6.91E-05 | 4.396112044 |
| OTUD1 | -1.513890849 | 7.240103031 | -5.852665541 | 3.57E-06 | 6.96E-05 | 4.387689256 |
| ERGIC3 | -1.360097043 | 7.573568795 | -5.849982607 | 3.59E-06 | 6.98E-05 | 4.380876347 |
| DMWD | 2.232089096 | 8.668683953 | 5.849296565 | 3.60E-06 | 6.98E-05 | 4.379134159 |
| WBSCR22 | -1.624029481 | 5.847477063 | -5.849230874 | 3.60E-06 | 6.98E-05 | 4.378967336 |
| LOC642852 | 2.742840905 | 10.78250071 | 5.848808353 | 3.61E-06 | 6.98E-05 | 4.377894335 |
| OSBPL9 | -2.397623532 | 8.060211081 | -5.848649197 | 3.61E-06 | 6.98E-05 | 4.377490149 |
| C22orf46 | 3.243491505 | 10.0495565 | 5.84627826 | 3.63E-06 | 7.01E-05 | 4.371468824 |
| LMTK2 | -2.409651245 | 4.734644449 | -5.845006966 | 3.64E-06 | 7.02E-05 | 4.36824002 |
| AK1 | -2.126898537 | 7.682968812 | -5.843644389 | 3.65E-06 | 7.03E-05 | 4.364779246 |
| C20orf201 | 2.249089232 | 7.804698986 | 5.843559322 | 3.66E-06 | 7.03E-05 | 4.364563181 |
| KIAA1549 | -3.12847628 | 5.228176561 | -5.841661264 | 3.67E-06 | 7.05E-05 | 4.359742109 |
| TBC1D10B | 1.582221779 | 11.97376484 | 5.840307141 | 3.69E-06 | 7.07E-05 | 4.356302471 |
| PAPLN | 1.804424967 | 8.694836731 | 5.835756937 | 3.73E-06 | 7.14E-05 | 4.34474339 |
| KIAA0930 | 1.587763085 | 10.74453504 | 5.83447273 | 3.74E-06 | 7.15E-05 | 4.341480784 |
| PINK1 | -2.488153994 | 6.830378122 | -5.834189071 | 3.75E-06 | 7.15E-05 | 4.340760115 |
| LRRN2 | 2.744439183 | 9.869303171 | 5.832889792 | 3.76E-06 | 7.16E-05 | 4.337459067 |
| THOC7 | -1.464324716 | 9.049042814 | -5.832436204 | 3.76E-06 | 7.16E-05 | 4.336306618 |
| C20orf30 | -1.693902018 | 9.445811563 | -5.831372297 | 3.77E-06 | 7.17E-05 | 4.333603448 |
| CYB5R3 | 1.740660903 | 13.30682977 | 5.83069948 | 3.78E-06 | 7.17E-05 | 4.331893911 |
| RRP1 | 1.368495211 | 9.155208306 | 5.829842701 | 3.79E-06 | 7.17E-05 | 4.329716906 |
| JMJD8 | 1.378258953 | 7.842909421 | 5.823832609 | 3.85E-06 | 7.28E-05 | 4.314444237 |
| RPL31 | 1.753057336 | 14.30824298 | 5.821966453 | 3.87E-06 | 7.30E-05 | 4.309701478 |
| LOC100129104 | 1.964555587 | 9.086415404 | 5.820946725 | 3.88E-06 | 7.31E-05 | 4.307109772 |
| C3orf23 | -1.577127975 | 7.592279935 | -5.817646563 | 3.91E-06 | 7.36E-05 | 4.298721672 |
| KDR | -1.577434606 | 6.448166322 | -5.817285205 | 3.91E-06 | 7.36E-05 | 4.297803154 |
| MRPL34 | -1.505843508 | 9.569601818 | -5.810682641 | 3.98E-06 | 7.47E-05 | 4.281018706 |
| CAPSL | -3.871803026 | 4.922127053 | -5.807306626 | 4.02E-06 | 7.52E-05 | 4.27243528 |
| GRIN1 | 3.119580618 | 9.894913838 | 5.80715765 | 4.02E-06 | 7.52E-05 | 4.272056491 |
| ZNF385B | -2.543802808 | 5.239039318 | -5.805795055 | 4.03E-06 | 7.53E-05 | 4.268591884 |
| HIST1H4D | 1.944854619 | 7.919599476 | 5.805228473 | 4.04E-06 | 7.53E-05 | 4.267151222 |
| DUS1L | -2.208775879 | 5.67821969 | -5.804951885 | 4.04E-06 | 7.53E-05 | 4.266447927 |
| LOC389199 | 2.131545079 | 7.14472802 | 5.80413195 | 4.05E-06 | 7.54E-05 | 4.264362999 |
| CDK2AP1 | -1.921447043 | 9.743492045 | -5.802809494 | 4.06E-06 | 7.55E-05 | 4.261000165 |
| ENOPH1 | -2.818425418 | 5.832836733 | -5.800442888 | 4.09E-06 | 7.59E-05 | 4.254981874 |
| PROSC | -1.680168112 | 7.311551545 | -5.798233653 | 4.11E-06 | 7.61E-05 | 4.249363417 |
| RHEB | -1.70606162 | 7.476184303 | -5.797916042 | 4.12E-06 | 7.61E-05 | 4.24855565 |
| ZFYVE9 | -1.439544873 | 6.434931413 | -5.797650295 | 4.12E-06 | 7.61E-05 | 4.247879782 |
| SH3RF1 | -1.583541382 | 7.139996927 | -5.794547569 | 4.15E-06 | 7.66E-05 | 4.239988305 |
| LINC00312 | -2.579390982 | 5.802562882 | -5.794269798 | 4.15E-06 | 7.66E-05 | 4.239281789 |
| STARD7 | -3.256148371 | 7.633074384 | -5.79361782 | 4.16E-06 | 7.66E-05 | 4.237623448 |
| MMP17 | 2.724190928 | 9.247711561 | 5.791637628 | 4.18E-06 | 7.69E-05 | 4.232586534 |
| ARHGAP33 | 2.588290862 | 13.00484442 | 5.79078503 | 4.19E-06 | 7.69E-05 | 4.230417738 |
| PSMD13 | -1.360736787 | 7.61058363 | -5.790300103 | 4.20E-06 | 7.69E-05 | 4.229184181 |
| POLD1 | 1.378436811 | 8.737684303 | 5.789819625 | 4.20E-06 | 7.69E-05 | 4.227961926 |
| ERCC3 | -1.616260485 | 6.044613604 | -5.789014241 | 4.21E-06 | 7.69E-05 | 4.225913127 |
| MRPL14 | -1.619121766 | 10.36712854 | -5.782818165 | 4.28E-06 | 7.81E-05 | 4.21014953 |
| SNORA78 | 3.424665583 | 11.69301886 | 5.781732936 | 4.29E-06 | 7.82E-05 | 4.207388292 |
| FAM60A | 1.278902123 | 11.2207718 | 5.780701062 | 4.30E-06 | 7.83E-05 | 4.20476273 |
| RFXANK | -1.390495012 | 8.890491551 | -5.779859005 | 4.31E-06 | 7.83E-05 | 4.202620097 |
| KLC2 | 2.984429098 | 10.30628886 | 5.778925098 | 4.32E-06 | 7.83E-05 | 4.20024369 |
| DPYD | -2.112238025 | 6.385102199 | -5.778654051 | 4.33E-06 | 7.83E-05 | 4.199553975 |
| PCYOX1 | -3.114095168 | 8.137659728 | -5.778250462 | 4.33E-06 | 7.83E-05 | 4.198526982 |
| BOLA2B | 1.563539406 | 11.2920993 | 5.778046476 | 4.33E-06 | 7.83E-05 | 4.198007903 |
| TMUB2 | -1.780327902 | 6.244894334 | -5.777402675 | 4.34E-06 | 7.83E-05 | 4.196369618 |
| KAT6A | -1.825602935 | 9.001183791 | -5.776967802 | 4.35E-06 | 7.83E-05 | 4.195262981 |
| USP37 | -1.873013146 | 5.368508645 | -5.774955015 | 4.37E-06 | 7.86E-05 | 4.190140782 |
| C16orf59 | 1.372361777 | 5.789620866 | 5.773097752 | 4.39E-06 | 7.88E-05 | 4.185414115 |
| SF3B2 | -1.965733075 | 8.698516316 | -5.763112518 | 4.50E-06 | 8.08E-05 | 4.159997922 |
| TMEM219 | -1.807622105 | 6.542832119 | -5.761386574 | 4.53E-06 | 8.11E-05 | 4.155604042 |
| CRBN | -1.585937538 | 6.940234547 | -5.760202484 | 4.54E-06 | 8.12E-05 | 4.152589483 |
| PPM1F | 4.792892562 | 9.82790408 | 5.755518492 | 4.59E-06 | 8.21E-05 | 4.140663625 |
| NGFRAP1 | -1.874042562 | 12.14695542 | -5.752979888 | 4.63E-06 | 8.25E-05 | 4.134199484 |
| ASB3 | -2.359097056 | 6.913296408 | -5.752671196 | 4.63E-06 | 8.25E-05 | 4.133413418 |
| TMEM218 | -2.314974029 | 7.416407011 | -5.750416908 | 4.66E-06 | 8.28E-05 | 4.127672823 |
| PRX | -3.067221011 | 9.148481789 | -5.747682434 | 4.69E-06 | 8.32E-05 | 4.120708953 |
| C7orf23 | -1.849899738 | 8.576214262 | -5.747583665 | 4.69E-06 | 8.32E-05 | 4.120457411 |
| FAIM | -1.803241459 | 7.290499571 | -5.747304881 | 4.69E-06 | 8.32E-05 | 4.119747403 |
| PRDX2 | -1.974892707 | 7.963923211 | -5.743212297 | 4.74E-06 | 8.37E-05 | 4.109323785 |
| CHTF8 | -2.070759136 | 5.885641549 | -5.743116836 | 4.75E-06 | 8.37E-05 | 4.109080637 |
| SLC25A6 | -1.957857061 | 8.419765518 | -5.74270884 | 4.75E-06 | 8.37E-05 | 4.108041425 |
| CACNA1E | 2.906463434 | 9.671852454 | 5.742570876 | 4.75E-06 | 8.37E-05 | 4.107690012 |
| NDUFB10 | -2.141718179 | 8.933528308 | -5.740082638 | 4.78E-06 | 8.42E-05 | 4.101351922 |
| BTG2 | -2.538846928 | 10.45030597 | -5.735966023 | 4.83E-06 | 8.50E-05 | 4.090865076 |
| LOC493754 | -1.448472041 | 9.896932974 | -5.729145045 | 4.92E-06 | 8.64E-05 | 4.073486498 |
| DGCR6L | -2.093101417 | 4.76728331 | -5.725819731 | 4.96E-06 | 8.70E-05 | 4.065013087 |
| PTPRB | -2.315639433 | 5.832906009 | -5.724223957 | 4.98E-06 | 8.72E-05 | 4.06094655 |
| LOC100134663 | 2.651586601 | 8.983434426 | 5.720749809 | 5.03E-06 | 8.79E-05 | 4.052092726 |
| TERF2IP | -1.576538186 | 9.505149716 | -5.720310467 | 5.04E-06 | 8.79E-05 | 4.050973012 |
| SH3D21 | 2.465242526 | 9.244985652 | 5.719334185 | 5.05E-06 | 8.80E-05 | 4.048484797 |
| LYPD3 | 3.298787315 | 9.573381018 | 5.713145331 | 5.13E-06 | 8.93E-05 | 4.032710015 |
| FOXS1 | 2.959768189 | 9.405720935 | 5.711273536 | 5.16E-06 | 8.96E-05 | 4.027938498 |
| H3F3B | -1.920539933 | 11.18712081 | -5.709853127 | 5.17E-06 | 8.98E-05 | 4.024317481 |
| C1QTNF6 | 2.417509136 | 7.90323403 | 5.703152416 | 5.27E-06 | 9.12E-05 | 4.007233733 |
| POLR2I | -1.736618897 | 8.351044416 | -5.703130582 | 5.27E-06 | 9.12E-05 | 4.00717806 |
| ZNF592 | 1.254488125 | 9.952034293 | 5.696625486 | 5.36E-06 | 9.26E-05 | 3.99059023 |
| VDAC1 | -1.644719423 | 8.007163384 | -5.692569826 | 5.41E-06 | 9.35E-05 | 3.980247007 |
| ARF5 | -1.687120249 | 6.104576265 | -5.685104872 | 5.52E-06 | 9.52E-05 | 3.961206234 |
| CDC37L1 | -1.472907237 | 6.046404095 | -5.68447652 | 5.53E-06 | 9.52E-05 | 3.959603341 |
| DLG4 | 1.631973088 | 7.478200402 | 5.683777849 | 5.54E-06 | 9.52E-05 | 3.957821037 |
| GPR108 | -2.390876516 | 5.160621161 | -5.681714257 | 5.57E-06 | 9.56E-05 | 3.952556651 |
| MEF2BNB | 2.394682649 | 9.300491278 | 5.68020471 | 5.59E-06 | 9.59E-05 | 3.948705505 |
| MDK | 2.467720595 | 11.14397989 | 5.67898621 | 5.61E-06 | 9.61E-05 | 3.945596771 |
| MED28 | -1.50807916 | 5.904441219 | -5.67705864 | 5.64E-06 | 9.64E-05 | 3.940678809 |
| AHDC1 | 2.081987372 | 10.03952183 | 5.675491295 | 5.66E-06 | 9.65E-05 | 3.936679742 |
| NDUFA3 | -1.406682038 | 10.79233287 | -5.675270736 | 5.66E-06 | 9.65E-05 | 3.936116976 |
| TMOD1 | -2.142497262 | 4.836076746 | -5.675000832 | 5.67E-06 | 9.65E-05 | 3.935428299 |
| IGF2BP3 | 1.816648253 | 7.602257246 | 5.673870405 | 5.68E-06 | 9.67E-05 | 3.932543894 |
| ABHD6 | -2.681361418 | 7.819970837 | -5.669636616 | 5.75E-06 | 9.76E-05 | 3.921740209 |
| CCDC88B | 2.397568504 | 10.43436617 | 5.668676107 | 5.76E-06 | 9.76E-05 | 3.919289049 |
| PDPK1 | -1.982070573 | 7.282216009 | -5.668534562 | 5.76E-06 | 9.76E-05 | 3.91892783 |
| ZNF585A | -1.905166845 | 5.024599997 | -5.66802057 | 5.77E-06 | 9.76E-05 | 3.917616124 |
| VASP | 2.300766432 | 12.36160639 | 5.667605061 | 5.78E-06 | 9.76E-05 | 3.916555737 |
| CTSC | 1.674305081 | 11.27085804 | 5.664985434 | 5.82E-06 | 9.82E-05 | 3.909870137 |
| PID1 | -2.515083 | 6.682050039 | -5.663886537 | 5.83E-06 | 9.82E-05 | 3.907065491 |
| CDCA7L | -1.695715092 | 6.403907664 | -5.66386753 | 5.83E-06 | 9.82E-05 | 3.90701698 |
| SRSF8 | -2.270050001 | 7.653167777 | -5.663239809 | 5.84E-06 | 9.82E-05 | 3.905414853 |
| PSMA4 | -2.214942702 | 8.689858855 | -5.662946664 | 5.85E-06 | 9.82E-05 | 3.904666654 |
| PSMB3 | -2.537791209 | 6.92843205 | -5.660066612 | 5.89E-06 | 9.88E-05 | 3.897315557 |
| PIN1 | -1.479018548 | 6.501095595 | -5.65926313 | 5.90E-06 | 9.89E-05 | 3.895264643 |
| ZDHHC2 | -1.705071459 | 6.138451902 | -5.656910857 | 5.94E-06 | 9.93E-05 | 3.889260155 |
| SLC44A2 | -1.47559291 | 9.963532629 | -5.656609845 | 5.95E-06 | 9.93E-05 | 3.88849176 |
| STOM | -1.743687214 | 9.875949059 | -5.655700221 | 5.96E-06 | 9.94E-05 | 3.886169716 |
| POR | -1.645757946 | 5.842213084 | -5.653507596 | 5.99E-06 | 9.99E-05 | 3.880572279 |
| TIMM23 | -1.96384958 | 5.755059154 | -5.652803894 | 6.01E-06 | 9.99E-05 | 3.878775773 |
| ALDH6A1 | -2.294238678 | 7.99805553 | -5.652522867 | 6.01E-06 | 9.99E-05 | 3.87805832 |
| SHD | 3.306930235 | 8.857677042 | 5.65188057 | 6.02E-06 | 9.99E-05 | 3.87641854 |
| HIST1H2BO | 2.103384537 | 10.54288102 | 5.650529594 | 6.04E-06 | 1.00E-04 | 3.872969421 |
| LOC100507520 | 5.458350504 | 9.035359978 | 5.650189683 | 6.05E-06 | 1.00E-04 | 3.872101591 |
| NDUFA9 | -2.543663603 | 6.422860019 | -5.650042262 | 6.05E-06 | 1.00E-04 | 3.871725208 |
| SCP2 | -2.317468488 | 8.172523016 | -5.647082472 | 6.10E-06 | 0.000100552 | 3.864168215 |
| PLXNC1 | 1.696814709 | 7.582363561 | 5.646929803 | 6.10E-06 | 0.000100552 | 3.863778403 |
| ANKRD29 | -3.638929003 | 5.844819974 | -5.64585718 | 6.11E-06 | 0.000100705 | 3.86103962 |
| MAL | -1.755392457 | 8.934000944 | -5.644813946 | 6.13E-06 | 0.000100797 | 3.858375807 |
| MKKS | -2.004179531 | 6.421903716 | -5.644526063 | 6.14E-06 | 0.000100797 | 3.857640711 |
| ALG5 | -2.157550578 | 9.426968658 | -5.642977062 | 6.16E-06 | 0.000101076 | 3.853685315 |
| LOC284454 | 2.742248651 | 10.72020603 | 5.642006349 | 6.18E-06 | 0.000101121 | 3.851206513 |
| CRYZ | -2.494801482 | 6.395275857 | -5.641693732 | 6.18E-06 | 0.000101121 | 3.850408205 |
| HIST1H4L | 1.813246454 | 10.86451162 | 5.64134387 | 6.19E-06 | 0.000101121 | 3.849514778 |
| RNF219 | -2.584356184 | 5.74205004 | -5.639996272 | 6.21E-06 | 0.000101327 | 3.846073416 |
| PARL | -3.14570051 | 5.33833929 | -5.639276216 | 6.22E-06 | 0.000101327 | 3.844234563 |
| HIST1H2BH | 2.057566553 | 10.4969171 | 5.639105638 | 6.22E-06 | 0.000101327 | 3.843798944 |
| GPC3 | -2.539281328 | 6.960927035 | -5.638568176 | 6.23E-06 | 0.000101327 | 3.842426367 |
| YARS2 | -1.786535691 | 5.875896919 | -5.637912443 | 6.24E-06 | 0.000101327 | 3.840751723 |
| SLC22A18 | 1.471246339 | 8.275020259 | 5.637656894 | 6.25E-06 | 0.000101327 | 3.840099082 |
| ISLR | 2.770923844 | 12.07711159 | 5.635712033 | 6.28E-06 | 0.000101599 | 3.835132018 |
| SAP18 | -1.707413547 | 9.620330152 | -5.635664538 | 6.28E-06 | 0.000101599 | 3.835010716 |
| PLA2G16 | -2.466941928 | 11.78209133 | -5.634548184 | 6.30E-06 | 0.000101768 | 3.832159505 |
| IL4I1 | 3.166510443 | 9.543039484 | 5.631994199 | 6.34E-06 | 0.00010229 | 3.825636247 |
| ASPM | 3.195051402 | 8.032000103 | 5.631624674 | 6.35E-06 | 0.00010229 | 3.824692393 |
| GOLGA7 | -1.630454989 | 7.937425823 | -5.629656689 | 6.38E-06 | 0.000102689 | 3.819665554 |
| HIST1H3G | 2.194853276 | 8.230055276 | 5.627705264 | 6.41E-06 | 0.000103084 | 3.814680785 |
| ST6GALNAC2 | -1.833996492 | 6.627458268 | -5.624077809 | 6.47E-06 | 0.000103936 | 3.805414117 |
| RTN4RL2 | 2.288526922 | 9.075732935 | 5.622160516 | 6.51E-06 | 0.000104327 | 3.800515899 |
| MTMR11 | 2.476842931 | 10.24565846 | 5.619655109 | 6.55E-06 | 0.000104881 | 3.794114864 |
| NDUFAF4 | -1.770337775 | 6.291838516 | -5.619027463 | 6.56E-06 | 0.000104923 | 3.792511241 |
| TUSC1 | -1.652269485 | 7.642759463 | -5.615846122 | 6.61E-06 | 0.000105667 | 3.784382613 |
| SLC34A3 | 3.072911831 | 9.974555742 | 5.614912038 | 6.63E-06 | 0.000105794 | 3.781995829 |
| ELN | 2.158895787 | 9.326300374 | 5.613538921 | 6.65E-06 | 0.000106042 | 3.778487126 |
| CKAP5 | -2.920796186 | 5.299733142 | -5.61105518 | 6.70E-06 | 0.000106601 | 3.772140181 |
| CDKN2A | 2.004527559 | 8.74296055 | 5.610145185 | 6.71E-06 | 0.000106722 | 3.769814691 |
| ANGPTL2 | 1.70419987 | 9.140892415 | 5.608914073 | 6.73E-06 | 0.000106934 | 3.766668512 |
| FAP | 3.359937174 | 9.572169168 | 5.60356669 | 6.83E-06 | 0.000108289 | 3.753001931 |
| BIN1 | 2.709790685 | 10.72805932 | 5.603150556 | 6.84E-06 | 0.000108289 | 3.751938327 |
| ADAMTS14 | 3.289872116 | 8.360420017 | 5.600940764 | 6.88E-06 | 0.000108782 | 3.746290109 |
| SF3A1 | -1.672639714 | 8.012952977 | -5.599157657 | 6.91E-06 | 0.00010905 | 3.741732291 |
| CA4 | -3.979803806 | 5.715808877 | -5.599059714 | 6.91E-06 | 0.00010905 | 3.741481931 |
| ADIPOR1 | -1.282498498 | 9.288824882 | -5.597393666 | 6.94E-06 | 0.000109392 | 3.737223154 |
| HCRT | 2.599278186 | 10.56065476 | 5.594961787 | 6.98E-06 | 0.000109955 | 3.731006459 |
| CDC26 | -1.559078856 | 8.2071279 | -5.593115084 | 7.02E-06 | 0.000110352 | 3.726285443 |
| MYEOV | 3.355720388 | 9.494230233 | 5.587906145 | 7.11E-06 | 0.000111728 | 3.712967962 |
| PARD6G | 1.58379099 | 10.84713652 | 5.587386466 | 7.12E-06 | 0.000111743 | 3.711639237 |
| POLR2J2 | 2.208898055 | 8.476481735 | 5.586589528 | 7.14E-06 | 0.00011184 | 3.709601577 |
| SNX3 | -2.16367334 | 9.524433865 | -5.586119278 | 7.15E-06 | 0.000111841 | 3.708399197 |
| RPA2 | -2.504070761 | 7.603775982 | -5.585151092 | 7.17E-06 | 0.000111989 | 3.705923609 |
| PPP2R1A | -1.49027759 | 7.380637032 | -5.584272294 | 7.18E-06 | 0.00011211 | 3.703676532 |
| PARVB | -2.044838633 | 4.538930947 | -5.582520759 | 7.22E-06 | 0.000112488 | 3.699197746 |
| RORC | 3.073609411 | 9.529908797 | 5.57998481 | 7.26E-06 | 0.000113099 | 3.692712858 |
| PYROXD1 | -1.872659938 | 6.085516196 | -5.574045738 | 7.38E-06 | 0.000114706 | 3.677524144 |
| CTRB2 | 2.259535806 | 9.617420531 | 5.573269333 | 7.39E-06 | 0.000114706 | 3.675538401 |
| CCDC66 | 5.207157435 | 10.12593478 | 5.573205492 | 7.39E-06 | 0.000114706 | 3.675375122 |
| PI4K2A | 2.640983336 | 9.805748119 | 5.572079848 | 7.41E-06 | 0.000114776 | 3.672496098 |
| PSMC2 | -1.482766029 | 8.004301776 | -5.572048418 | 7.42E-06 | 0.000114776 | 3.67241571 |
| CLTA | -1.939129254 | 10.06347004 | -5.56893967 | 7.48E-06 | 0.000115574 | 3.664464197 |
| LOC401176 | 3.004932781 | 7.474367447 | 5.565545447 | 7.54E-06 | 0.000116465 | 3.655781888 |
| CDK2AP2 | -1.842171437 | 5.922158993 | -5.564925164 | 7.56E-06 | 0.000116514 | 3.654195154 |
| LOC100131262 | 2.514875934 | 9.095457718 | 5.563669871 | 7.58E-06 | 0.000116757 | 3.650983952 |
| EIF4H | -1.932058591 | 10.27684229 | -5.562480657 | 7.60E-06 | 0.000116949 | 3.647941709 |
| PARP10 | 2.77194344 | 12.70026928 | 5.562128729 | 7.61E-06 | 0.000116949 | 3.647041393 |
| FOSL2 | 2.0262124 | 9.730802782 | 5.558777354 | 7.68E-06 | 0.000117838 | 3.638467435 |
| ARL17B | 2.691126222 | 10.13571339 | 5.558147435 | 7.69E-06 | 0.000117892 | 3.63685582 |
| CSDC2 | 3.134280142 | 12.48159833 | 5.557393994 | 7.71E-06 | 0.000117984 | 3.634928151 |
| RBM6 | 2.141280299 | 9.798980417 | 5.55657312 | 7.72E-06 | 0.000118097 | 3.632827919 |
| ACTB | 1.527707365 | 14.10075854 | 5.554351477 | 7.77E-06 | 0.000118644 | 3.627143593 |
| C10orf54 | -2.129840209 | 7.985781325 | -5.553843303 | 7.78E-06 | 0.000118661 | 3.625843335 |
| SEPT6 | 2.15511302 | 8.806365172 | 5.553015937 | 7.79E-06 | 0.000118777 | 3.623726334 |
| ATMIN | -1.49641391 | 5.304098227 | -5.548325377 | 7.89E-06 | 0.000120102 | 3.611723786 |
| ID3 | -1.787022123 | 7.484746349 | -5.546353199 | 7.93E-06 | 0.000120519 | 3.606676877 |
| MLXIP | 2.325395658 | 8.095920718 | 5.546095647 | 7.94E-06 | 0.000120519 | 3.606017772 |
| LINC00339 | -1.355012581 | 6.162181937 | -5.545001235 | 7.96E-06 | 0.000120723 | 3.603217009 |
| BLVRB | -1.681950073 | 7.897294718 | -5.538058714 | 8.11E-06 | 0.000122791 | 3.585448571 |
| TMEM143 | 1.742985997 | 10.30927357 | 5.536880419 | 8.13E-06 | 0.000123026 | 3.58243263 |
| CBR1 | -2.035177977 | 8.669224396 | -5.535908706 | 8.15E-06 | 0.000123071 | 3.579945399 |
| C16orf89 | -2.939260012 | 7.089003591 | -5.535842891 | 8.15E-06 | 0.000123071 | 3.579776934 |
| LOC613126 | 1.301715919 | 15.57077885 | 5.534514289 | 8.18E-06 | 0.000123355 | 3.576376109 |
| APPL1 | -1.942290482 | 8.817852637 | -5.533034367 | 8.21E-06 | 0.000123689 | 3.572587841 |
| OBFC1 | -1.652270848 | 6.308173832 | -5.526482834 | 8.35E-06 | 0.000125681 | 3.555816004 |
| LCE3E | 2.681855343 | 9.239730565 | 5.524195675 | 8.40E-06 | 0.000126288 | 3.549960377 |
| ALG3 | 1.792496335 | 8.991503611 | 5.521241497 | 8.47E-06 | 0.000127121 | 3.542396638 |
| TSPO | -1.619096426 | 8.167309801 | -5.520722774 | 8.48E-06 | 0.000127145 | 3.541068476 |
| CCDC146 | -2.781945445 | 6.302187096 | -5.518635873 | 8.53E-06 | 0.000127693 | 3.53572494 |
| JOSD1 | -1.804719837 | 8.453987525 | -5.516657682 | 8.57E-06 | 0.00012785 | 3.530659552 |
| ZBTB38 | -2.4158961 | 7.759567824 | -5.516566769 | 8.57E-06 | 0.00012785 | 3.530426754 |
| POLM | 2.223681491 | 8.729492832 | 5.516403547 | 8.58E-06 | 0.00012785 | 3.530008796 |
| PRKRIR | -2.648519885 | 7.104834504 | -5.516392641 | 8.58E-06 | 0.00012785 | 3.529980871 |
| TRNT1 | 1.794890377 | 8.44821304 | 5.515682081 | 8.59E-06 | 0.000127904 | 3.528161339 |
| H2AFZ | -2.24363654 | 8.961413118 | -5.515347966 | 8.60E-06 | 0.000127904 | 3.527305762 |
| MRPL10 | -1.748771322 | 7.069988147 | -5.514568786 | 8.62E-06 | 0.000128017 | 3.525310475 |
| TTC15 | -2.196836888 | 5.719269684 | -5.513131819 | 8.65E-06 | 0.000128351 | 3.521630674 |
| CAMTA1 | -1.287837213 | 7.82974111 | -5.511215484 | 8.70E-06 | 0.000128849 | 3.516723137 |
| SDHA | -1.419831029 | 7.067883698 | -5.508971465 | 8.75E-06 | 0.000129459 | 3.510976199 |
| NDUFC1 | -1.554221936 | 11.71314268 | -5.508531024 | 8.76E-06 | 0.00012946 | 3.509848197 |
| RTKN2 | -4.319401043 | 6.947834652 | -5.507984832 | 8.77E-06 | 0.000129496 | 3.508449346 |
| TADA2B | -1.744541205 | 5.126391049 | -5.506088264 | 8.81E-06 | 0.000129992 | 3.503591936 |
| MMP9 | 3.311109552 | 10.35820409 | 5.503963656 | 8.86E-06 | 0.000130567 | 3.498150262 |
| EGLN2 | -2.067879304 | 6.680768968 | -5.502540656 | 8.90E-06 | 0.000130757 | 3.494505464 |
| SPATA7 | -2.604262484 | 4.743801246 | -5.502525447 | 8.90E-06 | 0.000130757 | 3.494466508 |
| AK4 | 2.310559969 | 9.914783909 | 5.501852611 | 8.91E-06 | 0.000130757 | 3.492743102 |
| HIST1H2BB | 1.748138043 | 8.708710099 | 5.501666101 | 8.92E-06 | 0.000130757 | 3.492265371 |
| RPS9 | -1.462018577 | 9.04196667 | -5.500339797 | 8.95E-06 | 0.000130925 | 3.488868095 |
| IL17A | 2.998812771 | 9.901475259 | 5.500306276 | 8.95E-06 | 0.000130925 | 3.488782231 |
| GDE1 | -2.787975277 | 6.309778469 | -5.4992559 | 8.97E-06 | 0.000131136 | 3.486091668 |
| LMF2 | 2.929579214 | 9.6003016 | 5.497471374 | 9.01E-06 | 0.000131601 | 3.481520434 |
| TRNP1 | 1.89857552 | 9.098760507 | 5.49651053 | 9.04E-06 | 0.000131783 | 3.479059077 |
| RAD17 | -1.556628757 | 7.613156719 | -5.493921995 | 9.10E-06 | 0.00013253 | 3.472427895 |
| CDV3 | 1.863374807 | 11.54231707 | 5.492596449 | 9.13E-06 | 0.000132841 | 3.469032045 |
| IMPA2 | -1.84137372 | 10.09381941 | -5.489371349 | 9.21E-06 | 0.000133817 | 3.460769466 |
| ADD1 | -1.446025273 | 9.79316538 | -5.487697712 | 9.25E-06 | 0.000134251 | 3.456481472 |
| DISP1 | -2.694275953 | 6.564304501 | -5.487272244 | 9.26E-06 | 0.000134251 | 3.455391367 |
| GP1BB | 2.178504839 | 9.157651301 | 5.486132854 | 9.29E-06 | 0.000134501 | 3.452472053 |
| MRAS | 1.610056114 | 8.88601498 | 5.483908429 | 9.34E-06 | 0.000135136 | 3.446772511 |
| SDPR | -2.177191517 | 6.595819246 | -5.481397419 | 9.40E-06 | 0.000135875 | 3.440338377 |
| NIF3L1 | -2.831839259 | 6.029587744 | -5.480388969 | 9.43E-06 | 0.000136033 | 3.437754269 |
| ASXL2 | -1.523415584 | 7.413965515 | -5.479473927 | 9.45E-06 | 0.000136033 | 3.435409472 |
| RAB7L1 | 1.485933974 | 6.697472355 | 5.479431179 | 9.45E-06 | 0.000136033 | 3.43529993 |
| DPAGT1 | -1.792360579 | 5.881527714 | -5.479241671 | 9.46E-06 | 0.000136033 | 3.43481431 |
| ZNF124 | -1.436299618 | 6.944275816 | -5.475907055 | 9.54E-06 | 0.000137074 | 3.426268971 |
| OSBP | 1.756875965 | 8.821374693 | 5.475087365 | 9.56E-06 | 0.000137216 | 3.424168337 |
| COQ7 | -1.47818984 | 5.483582278 | -5.474383002 | 9.58E-06 | 0.000137316 | 3.422363229 |
| SF3B1 | -1.788405788 | 9.707277338 | -5.465825079 | 9.79E-06 | 0.000140229 | 3.400429494 |
| NDOR1 | 2.661965728 | 12.27341847 | 5.465368877 | 9.81E-06 | 0.000140229 | 3.399260164 |
| PI4KA | -1.602625885 | 6.982782738 | -5.465103886 | 9.81E-06 | 0.000140229 | 3.398580936 |
| GALNT5 | -2.687274758 | 5.032361281 | -5.462723277 | 9.87E-06 | 0.00014095 | 3.392478797 |
| KRTAP4-11 | 3.071273241 | 10.33558773 | 5.461748091 | 9.90E-06 | 0.000141154 | 3.389979057 |
| NXN | 1.542789726 | 8.220585291 | 5.458580833 | 9.98E-06 | 0.000142174 | 3.38185997 |
| GSK3B | -1.541872741 | 7.045291161 | -5.457647968 | 1.00E-05 | 0.000142292 | 3.379468531 |
| C16orf3 | 1.944799736 | 8.155442289 | 5.457419807 | 1.00E-05 | 0.000142292 | 3.378883627 |
| NME7 | -1.846552827 | 4.965440711 | -5.452955103 | 1.01E-05 | 0.000143809 | 3.367437569 |
| TM9SF1 | -2.602950415 | 7.10204942 | -5.449590644 | 1.02E-05 | 0.000144689 | 3.358811573 |
| KHDRBS1 | -2.094849871 | 7.707562008 | -5.449425168 | 1.02E-05 | 0.000144689 | 3.358387304 |
| KIAA1244 | -2.084749957 | 6.520824509 | -5.449368292 | 1.02E-05 | 0.000144689 | 3.358241476 |
| C7orf60 | -2.31683153 | 5.220994942 | -5.446899387 | 1.03E-05 | 0.000145445 | 3.351911201 |
| TRIM10 | 2.700877748 | 8.792152083 | 5.446545343 | 1.03E-05 | 0.000145445 | 3.351003407 |
| FAM107B | -2.903542709 | 7.865948081 | -5.445901238 | 1.03E-05 | 0.000145531 | 3.349351865 |
| NOL8 | 1.494996794 | 10.3325609 | 5.443158495 | 1.04E-05 | 0.000146421 | 3.342319013 |
| SMAP2 | 1.84856443 | 7.382421256 | 5.43979245 | 1.05E-05 | 0.000147558 | 3.333687442 |
| C16orf80 | -1.938999882 | 6.919645792 | -5.434029102 | 1.06E-05 | 0.000149643 | 3.318907267 |
| ITGB3 | 2.342832463 | 8.528366943 | 5.432040046 | 1.07E-05 | 0.000150041 | 3.313805964 |
| SFTA3 | -3.987101465 | 9.802572302 | -5.43101957 | 1.07E-05 | 0.000150041 | 3.311188696 |
| ARTN | 2.65515023 | 8.598249284 | 5.430859055 | 1.07E-05 | 0.000150041 | 3.31077701 |
| GRAMD1C | 4.524102675 | 8.9436006 | 5.430393616 | 1.07E-05 | 0.000150041 | 3.309583255 |
| TTBK1 | 2.639577599 | 8.97606264 | 5.430347833 | 1.07E-05 | 0.000150041 | 3.309465831 |
| CORO6 | 1.910918147 | 8.415654882 | 5.430283752 | 1.08E-05 | 0.000150041 | 3.309301476 |
| SEPP1 | -2.445831187 | 11.82223507 | -5.430111764 | 1.08E-05 | 0.000150041 | 3.308860359 |
| CEND1 | 2.333267962 | 8.087828129 | 5.429167742 | 1.08E-05 | 0.00015025 | 3.306439097 |
| WBP5 | -1.697764447 | 7.397329524 | -5.427858737 | 1.08E-05 | 0.000150604 | 3.303081646 |
| PTTG1 | 2.284503472 | 10.79915927 | 5.427065464 | 1.08E-05 | 0.000150755 | 3.301046953 |
| CYB561 | -1.70300605 | 8.221024419 | -5.425137311 | 1.09E-05 | 0.000151356 | 3.296101256 |
| TRAIP | 1.517511723 | 7.028115414 | 5.423916308 | 1.09E-05 | 0.000151678 | 3.292969305 |
| RAPGEF2 | -1.60930592 | 7.725418174 | -5.423009833 | 1.10E-05 | 0.000151875 | 3.290644097 |
| TMEM123 | -1.617947154 | 9.395184432 | -5.421740873 | 1.10E-05 | 0.000152218 | 3.287389015 |
| PRRG4 | 2.801267333 | 9.861786975 | 5.420074137 | 1.10E-05 | 0.000152591 | 3.283113471 |
| HSPA12B | -2.474092776 | 7.66873548 | -5.419988401 | 1.10E-05 | 0.000152591 | 3.282893536 |
| GNG5 | -1.570305933 | 8.83248491 | -5.419221515 | 1.11E-05 | 0.000152734 | 3.28092626 |
| CPLX2 | 2.787106651 | 8.536340619 | 5.417301488 | 1.11E-05 | 0.000153341 | 3.276000745 |
| ACAP3 | 1.69955744 | 6.355958668 | 5.414536754 | 1.12E-05 | 0.000154293 | 3.268907993 |
| FOXH1 | 2.931538708 | 9.462600322 | 5.412286069 | 1.13E-05 | 0.000155041 | 3.263133756 |
| SURF4 | 1.472065092 | 10.09616009 | 5.409244827 | 1.14E-05 | 0.000155966 | 3.255330967 |
| FAM89B | 1.40455586 | 11.58934349 | 5.40920506 | 1.14E-05 | 0.000155966 | 3.255228934 |
| SLC22A18AS | 2.351462719 | 8.27689224 | 5.406263413 | 1.15E-05 | 0.000157008 | 3.24768129 |
| CHMP2A | -1.921832316 | 8.736197089 | -5.405308224 | 1.15E-05 | 0.000157137 | 3.245230398 |
| ATP5G1 | -2.270137664 | 6.516899415 | -5.405138057 | 1.15E-05 | 0.000157137 | 3.244793768 |
| ZBTB32 | 2.399331148 | 7.860073098 | 5.40193475 | 1.16E-05 | 0.000158296 | 3.236574189 |
| RAD54L | 2.977250628 | 7.506806482 | 5.401182966 | 1.16E-05 | 0.000158368 | 3.234645076 |
| PLK1 | 2.326093498 | 8.006043486 | 5.4008847 | 1.16E-05 | 0.000158368 | 3.233879704 |
| ALAS1 | -1.955968339 | 5.969650721 | -5.400550378 | 1.16E-05 | 0.000158368 | 3.233021805 |
| PHLDA2 | 2.138967923 | 10.50123128 | 5.400146741 | 1.16E-05 | 0.000158368 | 3.231986032 |
| PAIP1 | -1.50334099 | 7.235422637 | -5.398571915 | 1.17E-05 | 0.000158856 | 3.227944806 |
| HIST1H1D | 2.734001085 | 10.01264148 | 5.395454613 | 1.18E-05 | 0.000159913 | 3.219945061 |
| RPS15 | -2.279348773 | 13.3080929 | -5.395242318 | 1.18E-05 | 0.000159913 | 3.219400248 |
| PHPT1 | -1.436565257 | 8.693408194 | -5.389254994 | 1.20E-05 | 0.000162186 | 3.204034158 |
| LOC401127 | 2.21757476 | 8.178511402 | 5.388608207 | 1.20E-05 | 0.000162186 | 3.202374133 |
| GFRA3 | 3.103359218 | 9.277746203 | 5.388515949 | 1.20E-05 | 0.000162186 | 3.202137344 |
| LOC90499 | 2.426356953 | 8.111481815 | 5.38826486 | 1.20E-05 | 0.000162186 | 3.2014929 |
| MB21D1 | 2.337794021 | 9.364610849 | 5.385633204 | 1.21E-05 | 0.000162991 | 3.194738351 |
| SAFB | 1.556188895 | 8.680488338 | 5.385337045 | 1.21E-05 | 0.000162991 | 3.193978195 |
| C21orf104 | 2.830742604 | 9.062040658 | 5.385135579 | 1.21E-05 | 0.000162991 | 3.193461086 |
| PAPOLG | -1.976052707 | 5.991597681 | -5.384782746 | 1.21E-05 | 0.000162991 | 3.192555458 |
| LOC100130152 | 2.874142614 | 11.90342265 | 5.384069795 | 1.21E-05 | 0.000163126 | 3.190725486 |
| FAM195A | 1.492804895 | 9.517178845 | 5.383343371 | 1.22E-05 | 0.000163267 | 3.188860912 |
| KIF13B | -1.318551793 | 9.241929392 | -5.380045523 | 1.23E-05 | 0.000164214 | 3.180395775 |
| LIN37 | 1.772943836 | 9.495621663 | 5.379976969 | 1.23E-05 | 0.000164214 | 3.1802198 |
| CADPS2 | -1.601446271 | 5.837201818 | -5.379950306 | 1.23E-05 | 0.000164214 | 3.180151358 |
| NR5A1 | 3.440101451 | 8.95666232 | 5.379481061 | 1.23E-05 | 0.000164246 | 3.178946831 |
| POMZP3 | 1.37242594 | 7.732346386 | 5.378008867 | 1.23E-05 | 0.000164711 | 3.175167731 |
| PCCB | -1.724983955 | 5.377253771 | -5.376573666 | 1.24E-05 | 0.000164994 | 3.171483508 |
| PPM1N | 1.913609232 | 8.16665957 | 5.376566349 | 1.24E-05 | 0.000164994 | 3.171464723 |
| GPR150 | 2.004559564 | 10.379261 | 5.375411347 | 1.24E-05 | 0.000165324 | 3.168499723 |
| SLC35A1 | -1.931169857 | 6.583433995 | -5.371936416 | 1.25E-05 | 0.000166667 | 3.159578923 |
| SEPT11 | 1.695241709 | 9.517274183 | 5.370986122 | 1.26E-05 | 0.000166911 | 3.157139257 |
| ASPHD1 | 2.055058679 | 7.131179277 | 5.370564361 | 1.26E-05 | 0.000166924 | 3.15605647 |
| PIGF | -2.295839238 | 5.756152761 | -5.369844128 | 1.26E-05 | 0.000167068 | 3.154207397 |
| NFE2L1 | 2.713884187 | 12.92505786 | 5.367883295 | 1.27E-05 | 0.000167757 | 3.149173199 |
| TBXA2R | 1.50530739 | 8.462529148 | 5.366149807 | 1.27E-05 | 0.00016835 | 3.144722558 |
| POLR2C | -2.071859304 | 7.372814275 | -5.362803887 | 1.28E-05 | 0.000169662 | 3.136131747 |
| IPO7 | 1.41287837 | 9.264213969 | 5.362059018 | 1.29E-05 | 0.00016982 | 3.1342192 |
| ATXN7L3B | -1.817458341 | 7.025193811 | -5.360588649 | 1.29E-05 | 0.000170302 | 3.130443778 |
| FAM82A1 | -2.362832917 | 5.962623767 | -5.359301523 | 1.30E-05 | 0.000170704 | 3.127138794 |
| MTPN | -2.169973567 | 9.357135662 | -5.358899975 | 1.30E-05 | 0.00017071 | 3.126107718 |
| HPCAL1 | -2.140100016 | 7.562712965 | -5.357914994 | 1.30E-05 | 0.000170977 | 3.123578501 |
| DPEP3 | 3.030856751 | 11.8809419 | 5.354837487 | 1.31E-05 | 0.000172189 | 3.115675894 |
| PHLDB3 | 1.671693517 | 9.269290734 | 5.354153554 | 1.31E-05 | 0.000172323 | 3.1139196 |
| ZNF606 | -2.208980541 | 4.873624133 | -5.353387867 | 1.32E-05 | 0.000172494 | 3.111953347 |
| LOC92659 | 2.653123534 | 11.05069263 | 5.352993309 | 1.32E-05 | 0.000172497 | 3.11094013 |
| QPRT | 2.091614837 | 10.66469536 | 5.35128443 | 1.32E-05 | 0.000173098 | 3.106551694 |
| SHMT2 | 1.581799853 | 9.049649684 | 5.349840268 | 1.33E-05 | 0.000173505 | 3.102842969 |
| MRPL54 | -1.661192994 | 7.294876155 | -5.349615562 | 1.33E-05 | 0.000173505 | 3.102265901 |
| HIST1H4A | 1.902106861 | 7.105140568 | 5.349157403 | 1.33E-05 | 0.000173507 | 3.101089292 |
| C20orf108 | -1.471703577 | 7.923461622 | -5.348386496 | 1.33E-05 | 0.000173507 | 3.099109487 |
| COL3A1 | 3.755235468 | 11.53921143 | 5.348160312 | 1.33E-05 | 0.000173507 | 3.098528611 |
| STXBP6 | -2.369455518 | 6.966665208 | -5.347830465 | 1.33E-05 | 0.000173507 | 3.097681506 |
| MAN2C1 | -1.610423502 | 5.415367127 | -5.347686784 | 1.34E-05 | 0.000173507 | 3.097312505 |
| ADA | 1.907849221 | 10.1525115 | 5.345288811 | 1.34E-05 | 0.000174427 | 3.091153953 |
| RNF44 | -1.669683041 | 6.992318578 | -5.342276651 | 1.35E-05 | 0.000175637 | 3.083417724 |
| PSMD11 | -1.757964792 | 6.954359347 | -5.339990556 | 1.36E-05 | 0.000176517 | 3.077546046 |
| ICAM5 | 1.188125625 | 7.219974465 | 5.330728033 | 1.40E-05 | 0.000180687 | 3.053753947 |
| CSTB | -2.469544713 | 11.51438044 | -5.330114183 | 1.40E-05 | 0.000180797 | 3.052177076 |
| WWC2 | -2.984466116 | 9.019838488 | -5.325470383 | 1.42E-05 | 0.000182834 | 3.040247556 |
| ZNF655 | -1.982378322 | 7.588004335 | -5.32447705 | 1.42E-05 | 0.000182959 | 3.03769567 |
| HS1BP3 | -1.710714286 | 6.264977947 | -5.324447344 | 1.42E-05 | 0.000182959 | 3.037619355 |
| EIF2B2 | -1.457756082 | 7.817357183 | -5.323757215 | 1.42E-05 | 0.000183108 | 3.035846382 |
| FAM176A | -2.777392886 | 5.745416587 | -5.322638455 | 1.43E-05 | 0.000183464 | 3.032972202 |
| CEP78 | 1.434692949 | 7.742363432 | 5.320827176 | 1.43E-05 | 0.000184156 | 3.028318796 |
| TBCA | -2.015681033 | 12.24973568 | -5.319627811 | 1.44E-05 | 0.000184553 | 3.025237412 |
| TBKBP1 | 2.397074 | 8.112037917 | 5.318054952 | 1.44E-05 | 0.000185133 | 3.021196376 |
| LSM12 | 1.32109073 | 11.51424215 | 5.316355665 | 1.45E-05 | 0.000185632 | 3.016830421 |
| CDC123 | -1.995218024 | 6.393321865 | -5.316060929 | 1.45E-05 | 0.000185632 | 3.016073151 |
| GTF2A1 | 1.097027574 | 8.631824901 | 5.315896293 | 1.45E-05 | 0.000185632 | 3.015650147 |
| MED15 | -2.961200057 | 5.411631301 | -5.315498474 | 1.45E-05 | 0.000185642 | 3.014628016 |
| TMEM41B | -2.719803238 | 6.522927348 | -5.314831143 | 1.46E-05 | 0.000185783 | 3.012913405 |
| CREB3 | 1.257775982 | 7.844649074 | 5.31385495 | 1.46E-05 | 0.000186076 | 3.010405189 |
| MUTYH | 1.20140106 | 8.772810747 | 5.310387718 | 1.47E-05 | 0.000187595 | 3.001496274 |
| JAG1 | -2.185870078 | 5.965164777 | -5.309490374 | 1.48E-05 | 0.000187852 | 2.999190516 |
| CSRNP2 | -1.714046984 | 6.154844151 | -5.306551557 | 1.49E-05 | 0.000189123 | 2.991638933 |
| CYB5R4 | -1.76431857 | 5.523561112 | -5.303137061 | 1.50E-05 | 0.000190577 | 2.982864677 |
| COMMD1 | -1.581662363 | 7.287076299 | -5.302888923 | 1.50E-05 | 0.000190577 | 2.982227021 |
| BCL2L1 | 1.416473487 | 8.387714726 | 5.302175816 | 1.51E-05 | 0.000190747 | 2.980394489 |
| ATP6V1E1 | -1.682396949 | 7.099478937 | -5.300657713 | 1.51E-05 | 0.000191322 | 2.976493234 |
| ROBO3 | 1.970044733 | 9.061838347 | 5.294404708 | 1.54E-05 | 0.000194304 | 2.960423326 |
| PPIAL4A | -1.97063357 | 11.00986426 | -5.293553655 | 1.54E-05 | 0.000194548 | 2.958236062 |
| SPTBN2 | 2.859665195 | 8.508042198 | 5.292843285 | 1.54E-05 | 0.000194721 | 2.956410347 |
| ANAPC10 | -1.953191555 | 6.188303973 | -5.292247947 | 1.55E-05 | 0.000194835 | 2.954880259 |
| ASXL1 | -1.541752914 | 6.923441275 | -5.291731424 | 1.55E-05 | 0.000194909 | 2.953552728 |
| GRP | -2.998189399 | 5.242928583 | -5.290491758 | 1.55E-05 | 0.000195304 | 2.950366588 |
| ND6 | 2.899832362 | 9.639172298 | 5.290218734 | 1.55E-05 | 0.000195304 | 2.949664868 |
| NPM1 | 1.094558011 | 11.86519063 | 5.289141786 | 1.56E-05 | 0.000195621 | 2.946896888 |
| MPRIP | -1.574611443 | 7.217498396 | -5.288859623 | 1.56E-05 | 0.000195621 | 2.946171666 |
| RPS6KB1 | -2.360797008 | 6.269331504 | -5.286992194 | 1.57E-05 | 0.000196213 | 2.941371884 |
| RXRB | 2.613957876 | 11.32025998 | 5.286969885 | 1.57E-05 | 0.000196213 | 2.941314545 |
| PEX7 | -1.720090619 | 6.218120964 | -5.286572762 | 1.57E-05 | 0.000196227 | 2.940293819 |
| RTN4 | -2.126301634 | 9.53918601 | -5.285968511 | 1.57E-05 | 0.000196349 | 2.938740705 |
| BRWD1-IT2 | 2.384952016 | 8.670195081 | 5.283700461 | 1.58E-05 | 0.000197332 | 2.932910997 |
| SFTPD | -4.269375493 | 9.064635711 | -5.282755453 | 1.58E-05 | 0.000197499 | 2.930481939 |
| TPI1P2 | -1.819946882 | 8.905647754 | -5.28260246 | 1.58E-05 | 0.000197499 | 2.93008868 |
| EDNRA | -2.480893666 | 6.672950696 | -5.282274542 | 1.59E-05 | 0.000197499 | 2.929245789 |
| TYW1 | -1.740121722 | 6.499708719 | -5.281772387 | 1.59E-05 | 0.000197569 | 2.927955025 |
| FLOT2 | -1.973423735 | 7.364412291 | -5.276834899 | 1.61E-05 | 0.000199959 | 2.915263039 |
| GPR160 | -2.129944944 | 6.315199238 | -5.275008691 | 1.62E-05 | 0.000200476 | 2.910568518 |
| CYP11B1 | 2.468237969 | 9.223506922 | 5.274768045 | 1.62E-05 | 0.000200476 | 2.909949896 |
| CABP4 | 2.914075222 | 11.35232595 | 5.27475411 | 1.62E-05 | 0.000200476 | 2.909914073 |
| C4orf3 | -1.428335942 | 8.638557903 | -5.272453734 | 1.63E-05 | 0.0002015 | 2.904000471 |
| PMF1 | -1.991635294 | 5.849713141 | -5.270555294 | 1.64E-05 | 0.000202314 | 2.899120007 |
| ARX | 3.715465487 | 10.16963106 | 5.268516454 | 1.64E-05 | 0.000203207 | 2.893878485 |
| PRR11 | 1.542078238 | 8.350539109 | 5.267729861 | 1.65E-05 | 0.000203341 | 2.89185625 |
| GNA11 | -1.414940124 | 6.648476355 | -5.267537171 | 1.65E-05 | 0.000203341 | 2.891360865 |
| OAZ2 | -1.497924546 | 8.601587539 | -5.265886878 | 1.66E-05 | 0.000204031 | 2.887118093 |
| TCTA | -1.620568803 | 4.94220697 | -5.265299988 | 1.66E-05 | 0.000204151 | 2.885609227 |
| C16orf5 | -1.749250173 | 4.513921709 | -5.264431285 | 1.66E-05 | 0.000204423 | 2.883375811 |
| LOC100652913 | 2.797270889 | 8.360747492 | 5.263479637 | 1.67E-05 | 0.00020474 | 2.880929122 |
| SCGB1A1 | -3.59963746 | 12.41470302 | -5.263044475 | 1.67E-05 | 0.000204779 | 2.879810308 |
| PRMT1 | -1.739104212 | 5.622548393 | -5.262601155 | 1.67E-05 | 0.000204822 | 2.878670518 |
| RFX4 | 3.236637048 | 8.541725842 | 5.261850612 | 1.67E-05 | 0.000204982 | 2.876740829 |
| POTEF | 1.825218048 | 12.49471123 | 5.261582016 | 1.67E-05 | 0.000204982 | 2.87605025 |
| CFLAR | 1.781071653 | 10.91273363 | 5.260857552 | 1.68E-05 | 0.000205178 | 2.874187592 |
| LYSMD3 | -2.265421356 | 7.292611968 | -5.260018073 | 1.68E-05 | 0.000205436 | 2.872029202 |
| C10orf118 | -1.861176182 | 6.772340017 | -5.259156383 | 1.69E-05 | 0.000205708 | 2.869813681 |
| TWF1 | -2.16362403 | 6.780008634 | -5.25839953 | 1.69E-05 | 0.000205923 | 2.867867695 |
| HSP90AA1 | 2.276543124 | 13.36710011 | 5.2565496 | 1.70E-05 | 0.000206732 | 2.863111164 |
| SNRPE | -1.577826748 | 8.913662026 | -5.253769576 | 1.71E-05 | 0.000208054 | 2.855962996 |
| KRTAP2-4 | 2.749717811 | 9.418694276 | 5.2527953 | 1.71E-05 | 0.000208391 | 2.853457822 |
| MAZ | 1.635990596 | 7.060061852 | 5.252236446 | 1.72E-05 | 0.000208501 | 2.852020821 |
| RLTPR | 2.253777314 | 7.593628977 | 5.250809561 | 1.72E-05 | 0.000209088 | 2.848351775 |
| LRRTM1 | 2.87465467 | 8.877319052 | 5.250336126 | 1.73E-05 | 0.000209151 | 2.847134387 |
| HJURP | 2.183346585 | 5.08765593 | 5.248941684 | 1.73E-05 | 0.000209723 | 2.843548689 |
| ERCC2 | 2.736530025 | 10.58553972 | 5.24797132 | 1.74E-05 | 0.000210062 | 2.841053444 |
| FAHD2A | -1.402081679 | 6.101902052 | -5.245464472 | 1.75E-05 | 0.000211254 | 2.834607074 |
| SLIT2 | -2.142141017 | 6.533499584 | -5.244915952 | 1.75E-05 | 0.000211361 | 2.833196528 |
| C19orf24 | 1.353946158 | 7.484955169 | 5.242979793 | 1.76E-05 | 0.000212081 | 2.828217538 |
| GNG8 | 1.578305424 | 5.520997186 | 5.242912414 | 1.76E-05 | 0.000212081 | 2.828044265 |
| FIS1 | -1.742139344 | 8.42281769 | -5.24244588 | 1.76E-05 | 0.000212143 | 2.826844518 |
| LRFN3 | 1.443284057 | 8.448603269 | 5.240560713 | 1.77E-05 | 0.000212999 | 2.821996529 |
| CGB1 | 5.02683312 | 8.56093523 | 5.236102759 | 1.79E-05 | 0.000215313 | 2.810531844 |
| SMURF2 | -1.423426993 | 6.377345697 | -5.235310506 | 1.79E-05 | 0.000215561 | 2.808494321 |
| MIAT | 3.12623563 | 10.3295312 | 5.233111598 | 1.81E-05 | 0.000216611 | 2.80283906 |
| CCDC15 | 3.21163372 | 11.1621682 | 5.231728243 | 1.81E-05 | 0.000217072 | 2.799281214 |
| EPCAM | -4.060177381 | 8.898785054 | -5.231597263 | 1.81E-05 | 0.000217072 | 2.798944344 |
| C21orf88 | 1.787597733 | 7.373391561 | 5.231109575 | 1.81E-05 | 0.000217148 | 2.797690045 |
| PYY2 | 3.53374779 | 10.26638749 | 5.22733926 | 1.83E-05 | 0.000219112 | 2.787992859 |
| HIST1H2BI | 1.767573224 | 9.360546933 | 5.2264635 | 1.84E-05 | 0.000219239 | 2.785740365 |
| HMP19 | 3.103310181 | 8.70296614 | 5.226071225 | 1.84E-05 | 0.000219239 | 2.784731411 |
| ZAN | 3.208483193 | 9.840601088 | 5.226061643 | 1.84E-05 | 0.000219239 | 2.784706763 |
| ANKRD46 | -1.888884299 | 6.247675696 | -5.223290217 | 1.85E-05 | 0.000220641 | 2.77757837 |
| SYT9 | 2.906564836 | 8.138417119 | 5.221175095 | 1.86E-05 | 0.000221669 | 2.772137925 |
| RSPO3 | 2.036704188 | 8.950046895 | 5.220166656 | 1.87E-05 | 0.000222053 | 2.769544009 |
| HIST1H2BJ | 1.832690005 | 9.364469056 | 5.219168176 | 1.87E-05 | 0.000222432 | 2.766975685 |
| RPSA | -2.078171062 | 10.01866116 | -5.21825251 | 1.88E-05 | 0.000222735 | 2.764620357 |
| FCN3 | -4.431936035 | 10.40874465 | -5.217950798 | 1.88E-05 | 0.000222735 | 2.763844273 |
| DYNC1H1 | -2.709288386 | 9.253826887 | -5.217326824 | 1.88E-05 | 0.000222896 | 2.762239234 |
| ACTA2 | -3.256414639 | 8.872015805 | -5.216385707 | 1.89E-05 | 0.000223244 | 2.759818393 |
| CECR1 | 2.622677458 | 9.55738131 | 5.215072359 | 1.89E-05 | 0.000223734 | 2.756440025 |
| TTBK2 | 2.219520287 | 7.678997604 | 5.214856194 | 1.89E-05 | 0.000223734 | 2.755883971 |
| CLASRP | 1.641600936 | 9.468197193 | 5.213755645 | 1.90E-05 | 0.000224103 | 2.753052954 |
| KLHDC2 | -3.183619131 | 7.241321437 | -5.213534092 | 1.90E-05 | 0.000224103 | 2.752483034 |
| UMOD | 2.675281474 | 10.27731562 | 5.209296677 | 1.92E-05 | 0.000226411 | 2.74158253 |
| SAMD4A | 2.044075804 | 7.940511034 | 5.208089373 | 1.93E-05 | 0.000226925 | 2.738476729 |
| CTNND1 | -1.307121722 | 7.862209866 | -5.207066757 | 1.93E-05 | 0.000227329 | 2.735846012 |
| NT5DC1 | -2.713504673 | 6.254054881 | -5.206289999 | 1.94E-05 | 0.000227422 | 2.733847757 |
| HNRNPA1 | -1.82937051 | 11.04535707 | -5.20621779 | 1.94E-05 | 0.000227422 | 2.733661994 |
| VDAC3 | -2.00537848 | 7.546868908 | -5.204158628 | 1.95E-05 | 0.000228418 | 2.728364604 |
| FBXO30 | -1.946406371 | 6.83551476 | -5.203579642 | 1.95E-05 | 0.000228418 | 2.726875091 |
| DDX20 | -1.928872523 | 6.458568915 | -5.203387116 | 1.95E-05 | 0.000228418 | 2.726379789 |
| MTRNR2L2 | 1.130409086 | 15.62370588 | 5.203177558 | 1.95E-05 | 0.000228418 | 2.725840674 |
| NUMBL | 1.896897015 | 7.046734633 | 5.202760223 | 1.96E-05 | 0.000228461 | 2.724767016 |
| LOC100129113 | 2.763613988 | 10.42239687 | 5.199701162 | 1.97E-05 | 0.000229984 | 2.716897 |
| LOC100652791 | 3.013354963 | 10.48088138 | 5.199551078 | 1.97E-05 | 0.000229984 | 2.716510875 |
| AHCYL2 | -2.280407626 | 8.997913435 | -5.198449968 | 1.98E-05 | 0.000230239 | 2.713678001 |
| TMSB4X | -1.91215596 | 12.0641283 | -5.198443007 | 1.98E-05 | 0.000230239 | 2.713660092 |
| PEAR1 | 2.673657332 | 8.479401063 | 5.197711528 | 1.98E-05 | 0.00023047 | 2.711778168 |
| OR11A1 | 3.333541153 | 8.805541092 | 5.197036633 | 1.99E-05 | 0.00023047 | 2.71004181 |
| DEFB129 | 3.244484082 | 9.208848683 | 5.197032711 | 1.99E-05 | 0.00023047 | 2.71003172 |
| POLR3E | -1.659128932 | 6.455024193 | -5.196197006 | 1.99E-05 | 0.00023077 | 2.707881617 |
| TMEM188 | -1.988228612 | 6.943372954 | -5.19519822 | 2.00E-05 | 0.00023117 | 2.705311919 |
| LOC100507637 | 3.049300629 | 8.959864851 | 5.194315424 | 2.00E-05 | 0.0002315 | 2.703040624 |
| CAB39L | -1.885016511 | 6.601573577 | -5.191769948 | 2.01E-05 | 0.000232724 | 2.696491413 |
| APCS | 2.78556576 | 8.704443827 | 5.191630881 | 2.01E-05 | 0.000232724 | 2.696133605 |
| C19orf66 | 2.020904913 | 10.60955768 | 5.189995701 | 2.02E-05 | 0.00023352 | 2.691926393 |
| ACAN | 3.200563584 | 8.219442878 | 5.188957026 | 2.03E-05 | 0.00023395 | 2.689253918 |
| LEPROT | -2.436733032 | 11.45576867 | -5.186436766 | 2.04E-05 | 0.000235257 | 2.682769275 |
| PAXIP1 | -1.197370098 | 6.22860426 | -5.186162468 | 2.04E-05 | 0.000235257 | 2.682063496 |
| GCOM1 | -1.370789352 | 6.89115609 | -5.185372147 | 2.05E-05 | 0.000235453 | 2.680029961 |
| COX5B | -1.703185449 | 11.19978188 | -5.185168616 | 2.05E-05 | 0.000235453 | 2.679506266 |
| CD81 | -3.23320245 | 11.48959081 | -5.184665993 | 2.05E-05 | 0.000235554 | 2.678212982 |
| ALDH9A1 | -2.157929046 | 6.296535524 | -5.182310944 | 2.06E-05 | 0.000236809 | 2.672153206 |
| GALNT10 | 1.39481448 | 7.359199841 | 5.179539994 | 2.08E-05 | 0.000238333 | 2.665023117 |
| HAUS3 | -1.383740306 | 5.335018075 | -5.176444259 | 2.10E-05 | 0.000240072 | 2.657057109 |
| KCNH2 | 3.027598434 | 8.886597987 | 5.17535511 | 2.10E-05 | 0.000240548 | 2.654254439 |
| SH3GL3 | -2.266346306 | 4.748244193 | -5.174349215 | 2.11E-05 | 0.000240898 | 2.651665981 |
| ACYP1 | -1.700173499 | 7.687467116 | -5.174128798 | 2.11E-05 | 0.000240898 | 2.651098782 |
| EDEM1 | 1.493884285 | 8.949785389 | 5.171720539 | 2.12E-05 | 0.000242217 | 2.644901538 |
| ZNF823 | -2.013995376 | 5.338018815 | -5.170392408 | 2.13E-05 | 0.000242851 | 2.641483768 |
| CRIP2 | -1.990000241 | 6.151035584 | -5.168363566 | 2.14E-05 | 0.000243937 | 2.636262738 |
| RNASEK | -1.753062638 | 10.26088269 | -5.167974033 | 2.14E-05 | 0.000243972 | 2.635260301 |
| PAPSS2 | -2.755199015 | 7.264892811 | -5.167245384 | 2.15E-05 | 0.000244225 | 2.633385169 |
| ESD | -1.762560827 | 7.286990334 | -5.165578818 | 2.16E-05 | 0.000245083 | 2.629096319 |
| COG1 | -1.534567195 | 7.170469304 | -5.163087672 | 2.17E-05 | 0.000246263 | 2.622685335 |
| GMFB | 1.527314345 | 8.353611775 | 5.16253068 | 2.17E-05 | 0.000246408 | 2.621251894 |
| NAPRT1 | 1.275249829 | 9.848326893 | 5.161165182 | 2.18E-05 | 0.000247079 | 2.617737706 |
| CHRNA4 | 2.767516393 | 9.289969237 | 5.160523936 | 2.19E-05 | 0.000247279 | 2.616087409 |
| PSMD6 | -1.684416239 | 7.353953564 | -5.158526784 | 2.20E-05 | 0.000248209 | 2.610947531 |
| COQ5 | -2.015833609 | 6.628302188 | -5.158434517 | 2.20E-05 | 0.000248209 | 2.610710069 |
| EFNA2 | 2.601943256 | 8.475198084 | 5.157462247 | 2.20E-05 | 0.000248628 | 2.608207802 |
| CDH19 | -2.435658288 | 4.989755235 | -5.156691238 | 2.21E-05 | 0.000248915 | 2.606223491 |
| HIST2H2BC | 1.759636164 | 9.389324567 | 5.154165523 | 2.22E-05 | 0.000250041 | 2.599723096 |
| PABPN1 | -1.971465512 | 9.737750185 | -5.15414045 | 2.22E-05 | 0.000250041 | 2.599658565 |
| PFN1P2 | 1.931360845 | 8.128680006 | 5.153984256 | 2.22E-05 | 0.000250041 | 2.599256568 |
| CHST8 | 2.75356349 | 9.104907378 | 5.152524106 | 2.23E-05 | 0.000250786 | 2.59549854 |
| PLEKHA4 | 1.908690993 | 12.12618808 | 5.151264611 | 2.24E-05 | 0.000251393 | 2.592256911 |
| SYCE1L | 2.840621569 | 10.98329634 | 5.150945924 | 2.24E-05 | 0.000251393 | 2.591436683 |
| SETD6 | -2.031820357 | 6.144365315 | -5.150476981 | 2.24E-05 | 0.000251485 | 2.590229731 |
| TBC1D14 | -1.637898487 | 6.549405051 | -5.15008972 | 2.25E-05 | 0.000251522 | 2.589233003 |
| C17orf80 | -2.657822611 | 5.37996528 | -5.148602361 | 2.26E-05 | 0.000252291 | 2.585404836 |
| CIRBP | -1.91753173 | 8.433809709 | -5.142972804 | 2.29E-05 | 0.000255842 | 2.5709151 |
| PSMA3 | -1.848998829 | 10.08724864 | -5.141672215 | 2.30E-05 | 0.000256498 | 2.56756747 |
| BAZ1B | -1.833136773 | 6.465288557 | -5.1386228 | 2.32E-05 | 0.000258345 | 2.559718364 |
| TTYH3 | 1.274181889 | 7.941771204 | 5.137594784 | 2.32E-05 | 0.000258822 | 2.557072244 |
| YIPF3 | -2.193384327 | 9.229087291 | -5.134787159 | 2.34E-05 | 0.00026052 | 2.5498453 |
| CCDC22 | -2.063992607 | 5.468183081 | -5.134268648 | 2.34E-05 | 0.000260651 | 2.548510617 |
| ADRBK1 | 1.354575107 | 7.270767334 | 5.133742211 | 2.35E-05 | 0.000260788 | 2.547155526 |
| RIPK3 | -1.467365743 | 5.17640154 | -5.131625472 | 2.36E-05 | 0.000262021 | 2.541706823 |
| RHPN1 | 2.121274473 | 10.3041916 | 5.130558217 | 2.37E-05 | 0.000262533 | 2.538959571 |
| ATAD2 | 1.538701318 | 6.725792474 | 5.129610012 | 2.37E-05 | 0.000262964 | 2.536518751 |
| LPL | -2.790902739 | 9.70885153 | -5.129060566 | 2.38E-05 | 0.000263118 | 2.535104388 |
| UNC119B | -2.544458747 | 7.237307433 | -5.128478809 | 2.38E-05 | 0.000263295 | 2.533606847 |
| RAPSN | 2.329949864 | 8.926551274 | 5.127152375 | 2.39E-05 | 0.000263991 | 2.530192361 |
| MRPL51 | -1.608084022 | 10.63258155 | -5.126371386 | 2.39E-05 | 0.000264126 | 2.528181937 |
| CNFN | 2.174046516 | 8.698135772 | 5.126307865 | 2.39E-05 | 0.000264126 | 2.52801842 |
| TMED4 | -1.280899234 | 7.23404442 | -5.123068321 | 2.41E-05 | 0.000266164 | 2.519679066 |
| RPAIN | -1.663668582 | 6.846798541 | -5.122528632 | 2.42E-05 | 0.000266315 | 2.518289762 |
| NBL1 | 1.923168981 | 9.484987656 | 5.120803124 | 2.43E-05 | 0.000267234 | 2.51384781 |
| RNASEL | -1.904038854 | 6.480791129 | -5.120572867 | 2.43E-05 | 0.000267234 | 2.513255059 |
| BBS10 | -1.967813729 | 6.330181578 | -5.120128027 | 2.43E-05 | 0.000267319 | 2.512109905 |
| CYP4F30P | 3.005033165 | 9.220290026 | 5.115336355 | 2.46E-05 | 0.000270489 | 2.499774471 |
| ARL2 | -1.498127353 | 6.711613828 | -5.1136809 | 2.47E-05 | 0.000271441 | 2.495512668 |
| LAMTOR2 | -2.112219545 | 6.791621777 | -5.113161286 | 2.48E-05 | 0.000271581 | 2.494174966 |
| PDCD6 | -2.000755429 | 10.20372428 | -5.112000609 | 2.48E-05 | 0.000272182 | 2.491186885 |
| SEMA6A | -1.526932087 | 7.697078639 | -5.111387994 | 2.49E-05 | 0.000272391 | 2.489609743 |
| HOMER3 | 2.49753451 | 12.67231843 | 5.107824387 | 2.51E-05 | 0.00027473 | 2.480435332 |
| PCMT1 | -1.696961684 | 6.365641655 | -5.106140233 | 2.52E-05 | 0.000275719 | 2.476099458 |
| EPB41L5 | -2.519435418 | 5.843174273 | -5.105420729 | 2.53E-05 | 0.000276008 | 2.474247075 |
| ATG3 | -1.633009866 | 6.818564414 | -5.104762985 | 2.53E-05 | 0.000276253 | 2.472553685 |
| ITGA11 | 2.093492734 | 6.982350813 | 5.104234222 | 2.54E-05 | 0.000276404 | 2.47119236 |
| MRPL11 | -1.481033198 | 6.433099933 | -5.10288147 | 2.54E-05 | 0.000277158 | 2.467709616 |
| ACER1 | 2.491021201 | 7.961315268 | 5.10119546 | 2.56E-05 | 0.000278158 | 2.463368844 |
| ANLN | 2.552661997 | 6.640734801 | 5.100651777 | 2.56E-05 | 0.000278322 | 2.461969077 |
| RBBP8 | -1.631855545 | 5.714888901 | -5.097960651 | 2.58E-05 | 0.000280069 | 2.455040446 |
| DCP2 | 1.331632759 | 7.57345418 | 5.09672711 | 2.59E-05 | 0.000280745 | 2.451864512 |
| RAD23B | -1.270095256 | 7.839610591 | -5.095098584 | 2.60E-05 | 0.000281716 | 2.447671599 |
| AHNAK2 | 2.578492048 | 7.830191951 | 5.09405714 | 2.60E-05 | 0.000282253 | 2.444990207 |
| EML1 | -1.519895904 | 7.170283785 | -5.093047803 | 2.61E-05 | 0.000282767 | 2.442391467 |
| IL21R | 1.945450879 | 7.681253122 | 5.092729764 | 2.61E-05 | 0.000282767 | 2.441572611 |
| SEMA4B | 2.563849878 | 9.518598305 | 5.092224075 | 2.62E-05 | 0.000282907 | 2.440270605 |
| NDNF | -1.938820772 | 7.435841179 | -5.091405178 | 2.62E-05 | 0.000283047 | 2.438162174 |
| C6orf47 | 1.515726193 | 9.240006331 | 5.091399646 | 2.62E-05 | 0.000283047 | 2.438147928 |
| SGCB | -1.546237119 | 6.669986574 | -5.090908181 | 2.63E-05 | 0.000283177 | 2.436882539 |
| PIEZO1 | -2.81078573 | 6.062920219 | -5.089887602 | 2.63E-05 | 0.000283703 | 2.434254813 |
| HLA-DRA | -3.60660156 | 10.38315846 | -5.087711857 | 2.65E-05 | 0.000285098 | 2.428652787 |
| HOPX | -1.772364546 | 10.49853337 | -5.084816744 | 2.67E-05 | 0.000287044 | 2.421198469 |
| TSC2 | -1.817787202 | 5.154925854 | -5.08326733 | 2.68E-05 | 0.000287979 | 2.417209005 |
| FTSJ3 | 1.486367049 | 10.43017346 | 5.08005812 | 2.70E-05 | 0.000290186 | 2.40894577 |
| LCE3D | 2.823709192 | 11.01961171 | 5.078363514 | 2.71E-05 | 0.000291044 | 2.404582365 |
| RGS2 | -2.426096187 | 9.922430678 | -5.078278419 | 2.72E-05 | 0.000291044 | 2.404363254 |
| BEST4 | 2.754783175 | 12.17206481 | 5.077992978 | 2.72E-05 | 0.000291044 | 2.403628276 |
| PRNP | 1.156720555 | 15.74265086 | 5.076578503 | 2.73E-05 | 0.000291889 | 2.399986141 |
| CRY1 | -1.45636125 | 5.901791517 | -5.074028021 | 2.75E-05 | 0.000293438 | 2.39341884 |
| FGF3 | 2.863635416 | 9.672143516 | 5.073943149 | 2.75E-05 | 0.000293438 | 2.3932003 |
| C9orf25 | 2.031691244 | 9.25079137 | 5.07338239 | 2.75E-05 | 0.000293629 | 2.391756378 |
| SSR4 | -1.947802731 | 10.27692082 | -5.068633266 | 2.79E-05 | 0.000297086 | 2.379527515 |
| LOC151009 | 2.036399314 | 9.186558372 | 5.068015273 | 2.79E-05 | 0.000297325 | 2.37793618 |
| LOC157740 | 2.077254219 | 8.453372452 | 5.067629264 | 2.79E-05 | 0.000297382 | 2.376942205 |
| ELMOD3 | 1.226980637 | 8.460984335 | 5.066771926 | 2.80E-05 | 0.000297708 | 2.374734548 |
| TMCO1 | -1.928068281 | 7.213090042 | -5.066327992 | 2.80E-05 | 0.000297708 | 2.373591408 |
| LOC439951 | 2.824465118 | 10.85837219 | 5.066274032 | 2.80E-05 | 0.000297708 | 2.37345246 |
| RPP14 | -1.158467979 | 6.882754932 | -5.064305485 | 2.82E-05 | 0.000298456 | 2.368383384 |
| TMEM186 | -2.696592994 | 5.526706021 | -5.064287646 | 2.82E-05 | 0.000298456 | 2.368337449 |
| SF3B5 | -1.632040957 | 10.27612078 | -5.064150554 | 2.82E-05 | 0.000298456 | 2.36798443 |
| HBXIP | -2.047463055 | 9.63787705 | -5.064074833 | 2.82E-05 | 0.000298456 | 2.367789446 |
| RALGDS | 1.483224154 | 8.981711515 | 5.063621027 | 2.82E-05 | 0.000298568 | 2.366620874 |
| RPLP0 | -2.167996426 | 11.88868667 | -5.06318217 | 2.83E-05 | 0.000298668 | 2.365490793 |
| ZNF259 | -1.860208827 | 6.633618718 | -5.061874769 | 2.84E-05 | 0.000299413 | 2.362124154 |
| SERHL2 | 2.01502652 | 8.66189269 | 5.061615849 | 2.84E-05 | 0.000299413 | 2.361457418 |
| OR10A5 | 2.500889927 | 7.894153106 | 5.060642727 | 2.84E-05 | 0.000299809 | 2.358951556 |
| TFF1 | 2.965918696 | 8.729878333 | 5.060494728 | 2.85E-05 | 0.000299809 | 2.358570445 |
| PTMA | 1.343410992 | 13.41422669 | 5.060135404 | 2.85E-05 | 0.000299848 | 2.357645157 |
| MASP2 | 2.808343436 | 8.85091816 | 5.059360983 | 2.85E-05 | 0.000300148 | 2.355650952 |
| SNRPA | -1.482416118 | 7.108305659 | -5.059137355 | 2.86E-05 | 0.000300148 | 2.35507509 |
| PSMA7 | -1.506740284 | 9.239989258 | -5.058234165 | 2.86E-05 | 0.000300619 | 2.352749284 |
| GTF3C2 | -1.701848351 | 8.354196066 | -5.057726575 | 2.87E-05 | 0.000300776 | 2.351442187 |
| DHX37 | 2.45244819 | 9.320732989 | 5.051950886 | 2.91E-05 | 0.000305149 | 2.336569 |
| MRPS7 | -1.437403346 | 6.625648891 | -5.051114401 | 2.92E-05 | 0.000305574 | 2.334414912 |
| TMEM100 | -3.749573992 | 9.293484484 | -5.050642691 | 2.92E-05 | 0.000305706 | 2.333200177 |
| GPR12 | 2.245905218 | 7.899177154 | 5.049418441 | 2.93E-05 | 0.000306446 | 2.330047513 |
| RALY | -2.385990718 | 6.119247571 | -5.048720673 | 2.94E-05 | 0.000306762 | 2.328250631 |
| KIAA0232 | -1.532522231 | 7.127217456 | -5.047676578 | 2.94E-05 | 0.000307246 | 2.325561882 |
| FOLR1 | -2.738690405 | 12.03821623 | -5.047376871 | 2.95E-05 | 0.000307246 | 2.324790076 |
| SMOC1 | 2.796340186 | 11.49796494 | 5.047200957 | 2.95E-05 | 0.000307246 | 2.324337061 |
| MRPS26 | -1.206246603 | 8.412357907 | -5.046820157 | 2.95E-05 | 0.000307306 | 2.323356424 |
| LOC92249 | 2.35941316 | 11.14557806 | 5.046505131 | 2.95E-05 | 0.000307313 | 2.322545166 |
| SEMA5B | -1.834637305 | 4.710213421 | -5.043663347 | 2.98E-05 | 0.000309377 | 2.315226941 |
| TOMM20 | -1.597062373 | 8.577885263 | -5.039379654 | 3.01E-05 | 0.000312644 | 2.304195359 |
| NAP1L4 | -1.420564752 | 5.198499213 | -5.038950671 | 3.01E-05 | 0.000312745 | 2.303090611 |
| HIST1H4F | 1.603021072 | 7.659337904 | 5.037812769 | 3.02E-05 | 0.000313434 | 2.300160203 |
| IL11 | 3.537125977 | 7.103690538 | 5.037494601 | 3.02E-05 | 0.000313444 | 2.299340831 |
| HLA-DOB | 1.772269835 | 8.11988136 | 5.036778402 | 3.03E-05 | 0.000313565 | 2.297496414 |
| C10orf27 | 2.704561449 | 10.31020507 | 5.036738906 | 3.03E-05 | 0.000313565 | 2.2973947 |
| FCRLB | 2.851661925 | 12.25781061 | 5.036130917 | 3.04E-05 | 0.000313816 | 2.295828954 |
| RPL29P2 | -1.521806906 | 5.792196167 | -5.033778573 | 3.05E-05 | 0.000315361 | 2.289770961 |
| GLIS3 | 2.319314432 | 9.473782981 | 5.033662961 | 3.06E-05 | 0.000315361 | 2.289473225 |
| ITGA3 | -1.585832405 | 6.983877473 | -5.032357321 | 3.07E-05 | 0.000316196 | 2.286110793 |
| GSR | 1.401756145 | 8.581124853 | 5.024885611 | 3.13E-05 | 0.000322239 | 2.266868575 |
| MGLL | -1.727157061 | 10.92550291 | -5.023830359 | 3.14E-05 | 0.000322879 | 2.264150911 |
| TIMM50 | 1.293497678 | 9.034179919 | 5.022482291 | 3.15E-05 | 0.000323771 | 2.260679126 |
| LRRC32 | -2.462627548 | 7.672566019 | -5.021633025 | 3.15E-05 | 0.000324125 | 2.258491942 |
| EIF4A2 | -1.753885704 | 9.982554643 | -5.021463803 | 3.16E-05 | 0.000324125 | 2.258056129 |
| LOC729915 | 1.581514138 | 6.603793266 | 5.020176289 | 3.17E-05 | 0.000324969 | 2.254740277 |
| HSH2D | 2.18397555 | 9.270777083 | 5.016986106 | 3.19E-05 | 0.000327457 | 2.246524268 |
| NUP155 | -1.777584144 | 5.232477187 | -5.016150867 | 3.20E-05 | 0.000327919 | 2.244373183 |
| DAB2 | 1.704571743 | 10.88285114 | 5.011947804 | 3.24E-05 | 0.000331315 | 2.233548502 |
| C4orf52 | -1.849100628 | 6.428415006 | -5.011421483 | 3.24E-05 | 0.000331512 | 2.232192992 |
| DYSF | 1.715085031 | 8.86943867 | 5.010349947 | 3.25E-05 | 0.000332187 | 2.229433315 |
| C8orf38 | -1.694484861 | 4.566779036 | -5.009585361 | 3.26E-05 | 0.000332594 | 2.227464163 |
| CDC42SE2 | -1.462011568 | 8.135062002 | -5.008869125 | 3.26E-05 | 0.00033296 | 2.225619535 |
| NOL12 | -1.338912584 | 4.669240991 | -5.007233688 | 3.28E-05 | 0.000334136 | 2.221407539 |
| PQBP1 | -2.120715763 | 5.865641667 | -5.006349383 | 3.28E-05 | 0.000334652 | 2.219130048 |
| RARG | 2.31593539 | 8.987282302 | 5.00408655 | 3.30E-05 | 0.000336391 | 2.213302197 |
| RSPH6A | 2.251280791 | 7.636943547 | 5.003597396 | 3.31E-05 | 0.00033656 | 2.212042393 |
| ANKRD33 | 2.291993791 | 7.901352465 | 5.000715662 | 3.33E-05 | 0.000338863 | 2.20462055 |
| CRB3 | 2.488613148 | 10.18257167 | 4.99945716 | 3.34E-05 | 0.000339723 | 2.201379296 |
| DENND4C | -2.42294723 | 6.67812832 | -4.999069146 | 3.35E-05 | 0.000339803 | 2.200379971 |
| CT47A11 | 3.085833632 | 12.82462263 | 4.997860741 | 3.36E-05 | 0.000340594 | 2.197267734 |
| ZNF335 | -1.963014889 | 5.254599414 | -4.997591298 | 3.36E-05 | 0.000340594 | 2.196573787 |
| UBE2MP1 | -1.67470525 | 4.399447423 | -4.994679773 | 3.39E-05 | 0.000342953 | 2.189075156 |
| THRA | -1.604572283 | 9.872976358 | -4.993595494 | 3.40E-05 | 0.000343434 | 2.186282592 |
| RFX7 | 1.592862447 | 9.362677006 | 4.993554089 | 3.40E-05 | 0.000343434 | 2.186175953 |
| LETM1 | 1.78970331 | 8.445207507 | 4.990594046 | 3.42E-05 | 0.000345858 | 2.178552331 |
| ZNF415 | -1.700303268 | 5.610243047 | -4.986258934 | 3.46E-05 | 0.000349567 | 2.167387169 |
| SLAMF6 | 2.681391122 | 8.417737465 | 4.985725899 | 3.47E-05 | 0.000349785 | 2.166014327 |
| VGLL4 | 1.331477243 | 8.251105064 | 4.985032124 | 3.47E-05 | 0.000350152 | 2.164227491 |
| SNAPIN | -1.514974106 | 6.581244194 | -4.983242478 | 3.49E-05 | 0.000351536 | 2.159618208 |
| SIGLECP3 | -2.031564897 | 5.042132147 | -4.981202395 | 3.51E-05 | 0.000353159 | 2.154363912 |
| WFDC10B | 2.975389438 | 10.66348891 | 4.978417544 | 3.53E-05 | 0.000355488 | 2.147191434 |
| DYRK1A | -1.191342391 | 6.842568101 | -4.975625435 | 3.56E-05 | 0.00035784 | 2.140000257 |
| LRIG1 | -1.558747266 | 7.338281574 | -4.973587919 | 3.58E-05 | 0.000359335 | 2.134752554 |
| FBXO17 | 2.884646178 | 10.77031656 | 4.973456909 | 3.58E-05 | 0.000359335 | 2.134415134 |
| KANK3 | -2.118745446 | 7.705835084 | -4.97293357 | 3.59E-05 | 0.000359426 | 2.133067253 |
| R3HDM2 | 1.679539631 | 10.62288731 | 4.972770695 | 3.59E-05 | 0.000359426 | 2.132647761 |
| OGT | 1.909068318 | 10.36169201 | 4.970998777 | 3.60E-05 | 0.000360832 | 2.128084117 |
| GLUL | -2.277224298 | 9.96661932 | -4.969800873 | 3.62E-05 | 0.000361694 | 2.124998865 |
| DUSP14 | -1.718589085 | 5.950962 | -4.967307556 | 3.64E-05 | 0.000363802 | 2.118577226 |
| SRSF3 | -1.631656457 | 11.11007695 | -4.965973942 | 3.65E-05 | 0.000364802 | 2.115142447 |
| TMEM133 | -1.764916681 | 6.251195948 | -4.963472819 | 3.68E-05 | 0.000366896 | 2.108700703 |
| MORC1 | 2.672966855 | 8.18201767 | 4.963114874 | 3.68E-05 | 0.000366896 | 2.107778802 |
| FER1L4 | 2.748555982 | 8.643792122 | 4.962927266 | 3.68E-05 | 0.000366896 | 2.10729561 |
| MON1B | 1.531870119 | 9.309897093 | 4.962594188 | 3.69E-05 | 0.000366935 | 2.106437756 |
| MINK1 | 1.754485078 | 7.715956379 | 4.961881802 | 3.69E-05 | 0.000367342 | 2.104602978 |
| ZNF410 | -1.699211123 | 7.568560059 | -4.961208662 | 3.70E-05 | 0.000367711 | 2.102869279 |
| LENG8 | 1.872193964 | 11.06827665 | 4.96060178 | 3.71E-05 | 0.00036778 | 2.101306232 |
| PIPOX | 2.738017632 | 7.975266872 | 4.960553188 | 3.71E-05 | 0.00036778 | 2.101181081 |
| HMGN4 | -1.336622819 | 8.217759164 | -4.958240014 | 3.73E-05 | 0.00036975 | 2.095223417 |
| MAGEC2 | 3.137226547 | 9.170296494 | 4.956951348 | 3.74E-05 | 0.000370725 | 2.091904413 |
| DLGAP3 | 2.708785872 | 11.52071849 | 4.956241161 | 3.75E-05 | 0.000371135 | 2.090075302 |
| USP28 | 2.92698391 | 9.864566048 | 4.955948947 | 3.75E-05 | 0.000371136 | 2.089322696 |
| CRYBB2 | 3.096657918 | 8.980296834 | 4.954448531 | 3.77E-05 | 0.000372324 | 2.085458328 |
| OR4D2 | 2.49466295 | 9.015302111 | 4.952762678 | 3.78E-05 | 0.00037353 | 2.081116364 |
| TUBB3 | 2.370930235 | 8.577716496 | 4.952469576 | 3.79E-05 | 0.00037353 | 2.080361472 |
| CUL9 | 2.080923188 | 11.54320456 | 4.952352936 | 3.79E-05 | 0.00037353 | 2.080061063 |
| DIAPH3 | 1.684159453 | 6.820853374 | 4.950733653 | 3.80E-05 | 0.000374845 | 2.075890558 |
| ARV1 | -1.660960492 | 6.534035348 | -4.94638647 | 3.85E-05 | 0.000378885 | 2.064694302 |
| IDH3G | -2.073275339 | 6.256624929 | -4.941754198 | 3.89E-05 | 0.000383011 | 2.052763847 |
| COL12A1 | -3.047480258 | 8.652410617 | -4.941708804 | 3.90E-05 | 0.000383011 | 2.052646934 |
| MECP2 | 1.150211953 | 10.44841883 | 4.940202838 | 3.91E-05 | 0.000384244 | 2.048768321 |
| ASB4 | 2.680641289 | 8.29686217 | 4.939630757 | 3.92E-05 | 0.000384532 | 2.047294928 |
| FOS | -3.3353357 | 8.453616313 | -4.937925394 | 3.93E-05 | 0.000385974 | 2.042902776 |
| SENP3 | -1.674180789 | 6.140834918 | -4.937255818 | 3.94E-05 | 0.000386363 | 2.041178292 |
| POLR2G | -1.461626221 | 9.606062175 | -4.936774381 | 3.95E-05 | 0.000386438 | 2.039938356 |
| MIER2 | 1.590191889 | 10.78178202 | 4.936605464 | 3.95E-05 | 0.000386438 | 2.039503314 |
| LOC494150 | -2.033038929 | 5.628445776 | -4.936227187 | 3.95E-05 | 0.000386531 | 2.038529065 |
| GALNT12 | -2.32133731 | 4.741283707 | -4.934018282 | 3.97E-05 | 0.000388498 | 2.032840075 |
| PNPLA6 | -1.423911743 | 5.659093599 | -4.93330705 | 3.98E-05 | 0.000388933 | 2.031008315 |
| DECR1 | -1.80940989 | 9.912078774 | -4.929302141 | 4.02E-05 | 0.000392773 | 2.020693811 |
| IL2RB | -2.403555043 | 6.236539769 | -4.928077731 | 4.04E-05 | 0.000393748 | 2.017540401 |
| KIAA1875 | 2.57117157 | 9.144108149 | 4.927242131 | 4.05E-05 | 0.000394319 | 2.015388354 |
| ABHD15 | -1.921085723 | 4.5674932 | -4.925584713 | 4.06E-05 | 0.00039575 | 2.011119767 |
| NT5C | -2.085589333 | 6.670673807 | -4.92460386 | 4.08E-05 | 0.00039642 | 2.008593642 |
| TRABD | 1.403626351 | 10.15671744 | 4.924372407 | 4.08E-05 | 0.00039642 | 2.00799755 |
| TSPAN5 | 1.512674407 | 8.943008199 | 4.923635954 | 4.09E-05 | 0.000396754 | 2.006100865 |
| MUC3A | 2.504508309 | 7.963663828 | 4.923482234 | 4.09E-05 | 0.000396754 | 2.005704969 |
| SOX18 | 2.531280025 | 9.400720887 | 4.921353525 | 4.11E-05 | 0.000398692 | 2.00022264 |
| HYI | -1.823520925 | 8.6164534 | -4.920367384 | 4.12E-05 | 0.00039935 | 1.997682918 |
| ATRN | -1.719265728 | 6.197986659 | -4.920158729 | 4.12E-05 | 0.00039935 | 1.997145544 |
| VCY | 3.170033501 | 10.87820472 | 4.919461139 | 4.13E-05 | 0.000399518 | 1.995348964 |
| OXT | 2.465057076 | 8.58600126 | 4.919430778 | 4.13E-05 | 0.000399518 | 1.995270771 |
| TCF21 | -2.773922149 | 6.161355386 | -4.918975526 | 4.14E-05 | 0.000399698 | 1.994098311 |
| LYNX1 | 2.67812221 | 9.933329582 | 4.915700643 | 4.17E-05 | 0.000402617 | 1.985664189 |
| SOX3 | 3.186538293 | 12.40477013 | 4.915634266 | 4.17E-05 | 0.000402617 | 1.985493242 |
| GNL3L | 1.123814368 | 10.36000912 | 4.9153708 | 4.18E-05 | 0.000402617 | 1.984814715 |
| MECOM | -1.838802764 | 6.828149009 | -4.914476063 | 4.19E-05 | 0.000403268 | 1.982510425 |
| ZNF730 | -1.774063052 | 5.729230125 | -4.914032589 | 4.19E-05 | 0.000403438 | 1.981368311 |
| MAPRE2 | -1.431347473 | 7.816478189 | -4.912504459 | 4.21E-05 | 0.000404768 | 1.977432807 |
| STARD3NL | -1.230440936 | 9.225995264 | -4.910941645 | 4.22E-05 | 0.00040614 | 1.973407992 |
| F11R | -2.543903492 | 12.39617749 | -4.90872567 | 4.25E-05 | 0.00040822 | 1.967701078 |
| ENOX2 | -1.29201584 | 5.434538592 | -4.907480268 | 4.26E-05 | 0.000409261 | 1.964493746 |
| GIMAP7 | -2.78017151 | 7.503736394 | -4.907010191 | 4.27E-05 | 0.000409303 | 1.963283141 |
| NCKAP5L | 1.203176001 | 9.090548265 | 4.906841483 | 4.27E-05 | 0.000409303 | 1.962848661 |
| KLK14 | 2.570166208 | 9.373712133 | 4.906530915 | 4.27E-05 | 0.000409303 | 1.962048846 |
| MSI2 | 1.31986653 | 8.428098725 | 4.906197458 | 4.28E-05 | 0.000409303 | 1.961190085 |
| ANXA5 | -2.756875759 | 10.99684637 | -4.906031686 | 4.28E-05 | 0.000409303 | 1.96076317 |
| MRPS36 | -1.261968403 | 10.62741868 | -4.905042475 | 4.29E-05 | 0.00041007 | 1.958215631 |
| MT1F | 2.265533912 | 10.49517617 | 4.903235027 | 4.31E-05 | 0.000411119 | 1.95356089 |
| ETFA | -1.64444321 | 6.600269325 | -4.903196825 | 4.31E-05 | 0.000411119 | 1.953462508 |
| PML | 1.703966204 | 9.558142778 | 4.903145241 | 4.31E-05 | 0.000411119 | 1.953329663 |
| SFTA2 | -3.065274346 | 8.504298925 | -4.902953126 | 4.32E-05 | 0.000411119 | 1.952834909 |
| PPA2 | -2.025028276 | 7.069632203 | -4.901340883 | 4.33E-05 | 0.000412569 | 1.948682902 |
| GTPBP3 | 1.496774183 | 7.670695787 | 4.900272777 | 4.35E-05 | 0.00041343 | 1.945932222 |
| SF3B14 | -1.289195747 | 9.690490774 | -4.899437826 | 4.36E-05 | 0.000413803 | 1.94378199 |
| RAI2 | -1.407097796 | 8.290841883 | -4.899371959 | 4.36E-05 | 0.000413803 | 1.943612364 |
| CILP2 | 2.636762692 | 7.796522972 | 4.89665462 | 4.39E-05 | 0.000416478 | 1.936614493 |
| MYH9 | -1.571774587 | 10.78623282 | -4.896315685 | 4.39E-05 | 0.000416544 | 1.935741649 |
| TONSL | 2.421021038 | 7.144841737 | 4.895162221 | 4.40E-05 | 0.000417314 | 1.932771195 |
| ATG2B | -1.766115607 | 6.767951018 | -4.895059844 | 4.41E-05 | 0.000417314 | 1.932507547 |
| MYBPC3 | 2.838730931 | 8.397588708 | 4.893845339 | 4.42E-05 | 0.000418104 | 1.929379909 |
| KIN | -1.662841892 | 5.577838905 | -4.893787024 | 4.42E-05 | 0.000418104 | 1.929229735 |
| SMC3 | -1.303480908 | 7.603784561 | -4.892238102 | 4.44E-05 | 0.00041932 | 1.925240913 |
| CSRP1 | -2.118864042 | 7.282102455 | -4.892132386 | 4.44E-05 | 0.00041932 | 1.924968669 |
| GNRH2 | 2.021336639 | 6.795314163 | 4.889117823 | 4.48E-05 | 0.000422365 | 1.917205553 |
| ERVV-2 | 2.437292844 | 7.726908203 | 4.888011504 | 4.49E-05 | 0.000423082 | 1.914356576 |
| LINC00421 | 2.719484745 | 8.347198513 | 4.88792132 | 4.49E-05 | 0.000423082 | 1.914124336 |
| HCN4 | 2.257910852 | 13.68558622 | 4.886247252 | 4.51E-05 | 0.000424447 | 1.909813322 |
| CECR5-AS1 | 2.540705898 | 10.30553434 | 4.886148754 | 4.51E-05 | 0.000424447 | 1.909559672 |
| XCR1 | 2.158009924 | 7.807446988 | 4.884633326 | 4.53E-05 | 0.000425839 | 1.905657207 |
| TMEM55B | -1.69894323 | 6.979436321 | -4.883305811 | 4.55E-05 | 0.000427024 | 1.902238666 |
| KCNJ10 | 3.023905452 | 9.263677373 | 4.882972394 | 4.55E-05 | 0.000427088 | 1.901380069 |
| SCAPER | -1.662826625 | 5.44223257 | -4.882458379 | 4.56E-05 | 0.000427357 | 1.900056414 |
| TRIAP1 | -2.033864018 | 5.835787881 | -4.881617602 | 4.57E-05 | 0.000427996 | 1.897891309 |
| SNAPC2 | 1.438031835 | 10.44545703 | 4.879922257 | 4.59E-05 | 0.000429051 | 1.893525603 |
| NKX2-5 | 2.240356822 | 7.679819797 | 4.87990937 | 4.59E-05 | 0.000429051 | 1.893492417 |
| SULT1A4 | -1.585541426 | 9.06618176 | -4.879734583 | 4.59E-05 | 0.000429051 | 1.893042322 |
| ND1 | 1.962272697 | 14.36187777 | 4.879425253 | 4.59E-05 | 0.000429051 | 1.892245766 |
| COPS7A | -1.340034892 | 6.772637447 | -4.879310068 | 4.59E-05 | 0.000429051 | 1.891949154 |
| KCNAB2 | 1.641852452 | 8.223149516 | 4.878894617 | 4.60E-05 | 0.000429211 | 1.890879328 |
| CTBS | -1.758281373 | 7.026687467 | -4.876286366 | 4.63E-05 | 0.000431867 | 1.884162869 |
| KDM3B | -1.108535543 | 7.701050849 | -4.875608198 | 4.64E-05 | 0.000432178 | 1.882416543 |
| SF1 | -1.392303413 | 9.285762874 | -4.875466029 | 4.64E-05 | 0.000432178 | 1.882050449 |
| TBCB | -1.895940899 | 9.180961018 | -4.87514324 | 4.64E-05 | 0.000432234 | 1.88121925 |
| ACO1 | -2.120229922 | 7.887864588 | -4.874393952 | 4.65E-05 | 0.000432777 | 1.879289793 |
| POM121L1P | 2.544557937 | 8.22733353 | 4.871894627 | 4.68E-05 | 0.000435331 | 1.872853944 |
| YPEL4 | 1.932712438 | 7.896508908 | 4.871043882 | 4.69E-05 | 0.000435795 | 1.870663264 |
| PIK3R5 | 3.278565243 | 7.984806032 | 4.87094552 | 4.70E-05 | 0.000435795 | 1.870409981 |
| CAMSAP1 | -2.076838473 | 4.047102771 | -4.870217933 | 4.71E-05 | 0.00043632 | 1.868536439 |
| CDSN | 2.309473232 | 8.047636033 | 4.868189223 | 4.73E-05 | 0.00043835 | 1.863312529 |
| HSD17B10 | -1.874493383 | 8.728265473 | -4.865409522 | 4.77E-05 | 0.000441265 | 1.856154898 |
| PCSK4 | 1.574592467 | 7.070717836 | 4.864233182 | 4.78E-05 | 0.000442321 | 1.853125891 |
| EVI2A | 1.94841674 | 9.24702318 | 4.863657647 | 4.79E-05 | 0.000442676 | 1.851643928 |
| ABHD14A | -1.53139419 | 6.437264609 | -4.863385521 | 4.79E-05 | 0.000442676 | 1.850943224 |
| ASL | -2.500986245 | 5.423766708 | -4.862739665 | 4.80E-05 | 0.000443114 | 1.849280196 |
| MCM3AP-AS1 | 2.634946951 | 9.526801462 | 4.8617393 | 4.81E-05 | 0.000443969 | 1.846704345 |
| C1orf21 | -1.470845839 | 7.860356736 | -4.860923476 | 4.82E-05 | 0.000444609 | 1.84460368 |
| CD164 | -1.58369435 | 8.036210646 | -4.860020037 | 4.83E-05 | 0.000444888 | 1.842277427 |
| SSTR3 | 2.499637383 | 9.021946821 | 4.859796491 | 4.84E-05 | 0.000444888 | 1.841701823 |
| ZNF236 | -2.159119025 | 5.689584895 | -4.859713733 | 4.84E-05 | 0.000444888 | 1.841488729 |
| PTGER4 | -2.320982195 | 7.934394577 | -4.859453321 | 4.84E-05 | 0.000444888 | 1.8408182 |
| MT2A | 1.330770462 | 15.49599971 | 4.859332222 | 4.84E-05 | 0.000444888 | 1.840506386 |
| C4orf41 | -2.709520036 | 6.537561213 | -4.857255494 | 4.87E-05 | 0.000447018 | 1.835159096 |
| CHAC1 | 1.918495932 | 8.069502461 | 4.852983636 | 4.92E-05 | 0.000451773 | 1.824159823 |
| LEPREL1 | -2.730728959 | 10.1345899 | -4.851924252 | 4.94E-05 | 0.000452717 | 1.821432136 |
| SP140L | 1.642442743 | 7.458268325 | 4.851118502 | 4.95E-05 | 0.000453359 | 1.819357509 |
| NPBWR1 | 2.824605225 | 8.052973475 | 4.848010949 | 4.99E-05 | 0.000456528 | 1.81135634 |
| VENTX | 1.332481351 | 6.354250616 | 4.847881209 | 4.99E-05 | 0.000456528 | 1.811022297 |
| FAM43B | 2.624181551 | 8.663184745 | 4.84767639 | 4.99E-05 | 0.000456528 | 1.810494943 |
| LYPD1 | 2.386210627 | 7.476984454 | 4.846203542 | 5.01E-05 | 0.000457945 | 1.806702782 |
| CYB5D2 | -1.983465877 | 6.267093859 | -4.845542404 | 5.02E-05 | 0.000457945 | 1.805000552 |
| C18orf12 | 2.464830281 | 9.426382863 | 4.845451149 | 5.02E-05 | 0.000457945 | 1.804765599 |
| EIF6 | -1.388684154 | 8.928064467 | -4.845430824 | 5.02E-05 | 0.000457945 | 1.804713267 |
| CA14 | 2.542059808 | 9.536784156 | 4.844880788 | 5.03E-05 | 0.000458286 | 1.803297096 |
| CBLL1 | -1.462183041 | 5.707900225 | -4.844070379 | 5.04E-05 | 0.000458944 | 1.801210555 |
| LOC100652762 | 2.487646653 | 8.9600948 | 4.843738697 | 5.05E-05 | 0.000459022 | 1.800356582 |
| MRPS27 | -1.666584427 | 6.751498233 | -4.842632068 | 5.06E-05 | 0.000460041 | 1.797507388 |
| LCE1A | 2.571746114 | 10.30792099 | 4.842359806 | 5.06E-05 | 0.000460048 | 1.796806409 |
| TMEM132C | -1.712697941 | 4.70154044 | -4.841175594 | 5.08E-05 | 0.000461165 | 1.793757487 |
| SPINT2 | -1.476095074 | 11.44102617 | -4.83743588 | 5.13E-05 | 0.000465418 | 1.784129205 |
| SOCS1 | 1.834538212 | 9.058376802 | 4.83660093 | 5.14E-05 | 0.000465629 | 1.781979568 |
| MAP2K3 | 1.549414972 | 10.97181327 | 4.836491399 | 5.14E-05 | 0.000465629 | 1.781697573 |
| FBXO15 | -2.041629926 | 5.682420511 | -4.836464985 | 5.14E-05 | 0.000465629 | 1.781629569 |
| ARGLU1 | 2.16106306 | 11.56827092 | 4.836036086 | 5.15E-05 | 0.000465816 | 1.780525342 |
| HTR1B | 2.612869123 | 8.502111624 | 4.835781356 | 5.15E-05 | 0.000465816 | 1.779869528 |
| MOS | 2.808074692 | 9.562930496 | 4.834248166 | 5.17E-05 | 0.00046738 | 1.775922267 |
| TXNDC11 | -1.251819929 | 7.412394532 | -4.832959832 | 5.19E-05 | 0.000468645 | 1.772605428 |
| SYDE2 | -2.022789794 | 5.001555952 | -4.830703649 | 5.22E-05 | 0.000470851 | 1.766796907 |
| PION | -1.713928057 | 6.959373799 | -4.830653308 | 5.22E-05 | 0.000470851 | 1.766667303 |
| DHDDS | 2.757437696 | 9.192760579 | 4.829088711 | 5.25E-05 | 0.000472423 | 1.76263931 |
| TMEM158 | 2.441684588 | 11.22061333 | 4.828862782 | 5.25E-05 | 0.000472423 | 1.762057669 |
| AAGAB | 1.164275485 | 9.718749986 | 4.828266661 | 5.26E-05 | 0.000472838 | 1.760522991 |
| PPP2R2A | -1.756930771 | 6.940256747 | -4.82781851 | 5.26E-05 | 0.000473067 | 1.759369259 |
| TAF1B | -2.131048938 | 6.080805996 | -4.827190169 | 5.27E-05 | 0.000473523 | 1.757751643 |
| FZD5 | -2.066445412 | 8.301598686 | -4.826616289 | 5.28E-05 | 0.000473698 | 1.756274238 |
| PVRL1 | 1.902086832 | 8.351363117 | 4.826522506 | 5.28E-05 | 0.000473698 | 1.756032803 |
| PCGF2 | 2.472140694 | 8.651375912 | 4.825583863 | 5.29E-05 | 0.000474458 | 1.753616361 |
| TFB2M | -1.962600856 | 5.832803415 | -4.825389137 | 5.30E-05 | 0.000474458 | 1.75311506 |
| OR10H2 | 2.565447313 | 8.4518975 | 4.824738941 | 5.31E-05 | 0.000474943 | 1.751441207 |
| ZNF843 | 3.28865721 | 9.338883242 | 4.82298813 | 5.33E-05 | 0.000476815 | 1.746933984 |
| SEMA6B | 2.359995949 | 8.413978676 | 4.822385015 | 5.34E-05 | 0.000477244 | 1.74538136 |
| GP1BA | 2.794631221 | 7.869431062 | 4.821865452 | 5.35E-05 | 0.000477568 | 1.744043829 |
| RAB25 | -2.854412511 | 5.275006068 | -4.82129092 | 5.35E-05 | 0.000477935 | 1.742564798 |
| CCDC92 | -2.13213272 | 7.19604866 | -4.820961381 | 5.36E-05 | 0.000477935 | 1.741716461 |
| GCM1 | 2.565913482 | 7.992459962 | 4.820788295 | 5.36E-05 | 0.000477935 | 1.741270883 |
| COL5A1 | 2.680497852 | 10.06987781 | 4.820140268 | 5.37E-05 | 0.000478423 | 1.739602663 |
| CTU2 | 2.323322534 | 9.117851248 | 4.819726689 | 5.38E-05 | 0.000478615 | 1.738537988 |
| HGC6.3 | 2.956339824 | 9.86854011 | 4.817250635 | 5.41E-05 | 0.000481425 | 1.732163949 |
| RAB32 | -1.449216943 | 7.552120851 | -4.815795465 | 5.43E-05 | 0.000482345 | 1.728417994 |
| SETMAR | -1.678444217 | 6.202975872 | -4.815759613 | 5.43E-05 | 0.000482345 | 1.728325705 |
| HOOK2 | -1.969069665 | 4.925494471 | -4.815744829 | 5.43E-05 | 0.000482345 | 1.728287647 |
| MAGEB2 | 2.281665484 | 7.197849778 | 4.809282442 | 5.53E-05 | 0.000490319 | 1.711652381 |
| ABCF1 | -1.516358526 | 7.424010324 | -4.807579368 | 5.55E-05 | 0.000492192 | 1.707268508 |
| PLS3 | -2.736462905 | 8.860141974 | -4.807307635 | 5.56E-05 | 0.000492207 | 1.706569048 |
| LOC100499466 | -1.232374901 | 8.258868938 | -4.806960982 | 5.56E-05 | 0.000492319 | 1.705676737 |
| BATF | 2.054358148 | 10.00557513 | 4.805091817 | 5.59E-05 | 0.000494418 | 1.700865416 |
| HSP90B1 | 1.606638564 | 13.95232236 | 4.801322569 | 5.65E-05 | 0.000499027 | 1.691163387 |
| PPP1R9A | -2.184325761 | 8.468068697 | -4.799623909 | 5.67E-05 | 0.000500929 | 1.686791131 |
| KCNJ1 | 2.438059185 | 8.084597105 | 4.796176306 | 5.72E-05 | 0.000505169 | 1.677917368 |
| C4BPA | -4.49735606 | 7.482990196 | -4.795381896 | 5.73E-05 | 0.000505884 | 1.675872676 |
| UBA2 | -1.727926275 | 6.623838448 | -4.794370847 | 5.75E-05 | 0.000506891 | 1.673270405 |
| MOP-1 | 2.315870267 | 8.381441878 | 4.794082067 | 5.75E-05 | 0.000506931 | 1.672527137 |
| EBLN2 | 2.0018635 | 10.00494593 | 4.792993857 | 5.77E-05 | 0.000508043 | 1.669726295 |
| IGHA1 | 2.668179329 | 7.527101279 | 4.791650482 | 5.79E-05 | 0.000509502 | 1.66626874 |
| SYPL1 | -1.876521344 | 7.79144773 | -4.79052585 | 5.81E-05 | 0.00051067 | 1.663374211 |
| CREB1 | -1.269125354 | 6.899465728 | -4.79017697 | 5.81E-05 | 0.000510792 | 1.662476283 |
| NARS | -1.704700679 | 6.275598928 | -4.789534051 | 5.82E-05 | 0.000511312 | 1.660821584 |
| AGAP3 | 1.450992395 | 10.9093615 | 4.788420773 | 5.84E-05 | 0.00051247 | 1.657956324 |
| AKR1C1 | -2.158127246 | 6.934189648 | -4.787816256 | 5.85E-05 | 0.000512631 | 1.656400481 |
| ZNF675 | -1.844421899 | 9.088605125 | -4.787582403 | 5.85E-05 | 0.000512631 | 1.655798617 |
| LOC100653058 | 2.094247525 | 7.554442343 | 4.787528468 | 5.85E-05 | 0.000512631 | 1.655659807 |
| PIK3CA | -1.80340271 | 6.43307311 | -4.785760903 | 5.88E-05 | 0.000514682 | 1.651110687 |
| ATF1 | -1.269830187 | 5.815341886 | -4.785283177 | 5.89E-05 | 0.000514954 | 1.649881193 |
| PROP1 | 2.356880037 | 9.097440203 | 4.785047189 | 5.89E-05 | 0.000514954 | 1.649273845 |
| NPAS3 | 1.883089688 | 11.20935658 | 4.783760084 | 5.91E-05 | 0.000516359 | 1.64596133 |
| DOCK3 | 3.044865833 | 9.089919431 | 4.783444913 | 5.92E-05 | 0.000516439 | 1.645150207 |
| TRIM25 | 2.684497524 | 8.519999312 | 4.778632811 | 5.99E-05 | 0.00052267 | 1.632766046 |
| RPS26 | 1.483463758 | 13.63245963 | 4.778396037 | 6.00E-05 | 0.00052267 | 1.632156712 |
| ZNF678 | -1.654829849 | 6.11156065 | -4.778055034 | 6.00E-05 | 0.000522787 | 1.631279145 |
| ZNF12 | -1.253226173 | 7.290205782 | -4.776192167 | 6.03E-05 | 0.000525013 | 1.626485133 |
| MCRS1 | -2.376377747 | 5.844294044 | -4.775120608 | 6.05E-05 | 0.000526147 | 1.623727555 |
| DNTTIP1 | -1.479290592 | 7.843158609 | -4.77002122 | 6.13E-05 | 0.000532929 | 1.610605014 |
| ANO6 | -1.56890429 | 8.382312806 | -4.768715839 | 6.15E-05 | 0.00053441 | 1.607245898 |
| MZT2A | 2.405845333 | 9.875007203 | 4.765509019 | 6.21E-05 | 0.000538597 | 1.59899401 |
| RAMP3 | -1.881990296 | 5.419666434 | -4.763915753 | 6.23E-05 | 0.00054028 | 1.59489426 |
| SHISA9 | 2.368063415 | 8.3331819 | 4.763819015 | 6.23E-05 | 0.00054028 | 1.594645338 |
| NOS3 | 2.961601066 | 12.40965264 | 4.763230461 | 6.24E-05 | 0.000540757 | 1.593130902 |
| KCNS3 | -2.058641445 | 8.580832917 | -4.761234084 | 6.28E-05 | 0.000543254 | 1.587993996 |
| ZBTB16 | -3.77445395 | 7.424770914 | -4.760805802 | 6.28E-05 | 0.000543503 | 1.586891989 |
| BMI1 | -1.558819614 | 7.723672792 | -4.759257324 | 6.31E-05 | 0.000545367 | 1.582907659 |
| ITLN2 | -4.329225835 | 6.691298675 | -4.757985389 | 6.33E-05 | 0.000546836 | 1.579634937 |
| MAPK1IP1L | -2.184786559 | 9.080461804 | -4.756672209 | 6.35E-05 | 0.000548174 | 1.57625613 |
| SNRNP70 | 1.290181746 | 11.95422129 | 4.756553572 | 6.35E-05 | 0.000548174 | 1.575950879 |
| TBC1D17 | 2.526807028 | 9.431975735 | 4.756248493 | 6.36E-05 | 0.000548249 | 1.575165917 |
| MTMR12 | -1.416912441 | 5.583072051 | -4.754972283 | 6.38E-05 | 0.000549734 | 1.571882291 |
| EIF2B1 | -1.505151935 | 8.272474725 | -4.75437617 | 6.39E-05 | 0.000550232 | 1.570348533 |
| TOP2B | -2.50470447 | 9.255623759 | -4.753894034 | 6.40E-05 | 0.000550566 | 1.569108038 |
| LCE1D | 2.969363023 | 13.70132463 | 4.753507807 | 6.41E-05 | 0.00055076 | 1.568114312 |
| NCALD | -1.77418242 | 5.402371472 | -4.751732437 | 6.44E-05 | 0.000552982 | 1.5635465 |
| TRDN | 3.132491013 | 10.00587271 | 4.751064305 | 6.45E-05 | 0.00055359 | 1.561827499 |
| MALAT1 | 2.46615073 | 13.72486458 | 4.749965337 | 6.47E-05 | 0.00055483 | 1.559000047 |
| ZNF35 | -1.429166114 | 5.048653017 | -4.746147692 | 6.53E-05 | 0.000559755 | 1.549178154 |
| ZNF346 | 2.219718566 | 7.65891709 | 4.746119015 | 6.53E-05 | 0.000559755 | 1.549104377 |
| FBLN1 | 2.095163546 | 13.16081273 | 4.745698648 | 6.54E-05 | 0.000560004 | 1.548022896 |
| COPS4 | -1.749182708 | 8.538757176 | -4.74438329 | 6.56E-05 | 0.000561581 | 1.544638896 |
| OSBPL11 | -1.552190661 | 6.264862776 | -4.743460292 | 6.58E-05 | 0.000562579 | 1.542264341 |
| COG2 | -1.687598366 | 6.845438848 | -4.742311505 | 6.60E-05 | 0.000563916 | 1.539308938 |
| FNDC9 | 2.704765709 | 9.487798378 | 4.741571982 | 6.61E-05 | 0.000564644 | 1.53740644 |
| HIST1H2BE | 1.663058305 | 10.26361985 | 4.740859107 | 6.62E-05 | 0.000565334 | 1.535572509 |
| ADCK3 | -2.416490809 | 5.754694758 | -4.739961943 | 6.64E-05 | 0.000566301 | 1.533264501 |
| UQCRH | -1.666953771 | 10.51661466 | -4.73960977 | 6.65E-05 | 0.000566453 | 1.53235852 |
| C2orf47 | -1.493341506 | 7.256153817 | -4.738708234 | 6.66E-05 | 0.000567082 | 1.530039292 |
| ESYT3 | -2.39622827 | 4.758100733 | -4.738689426 | 6.66E-05 | 0.000567082 | 1.529990908 |
| HEXA | -1.951696366 | 7.074369536 | -4.738254177 | 6.67E-05 | 0.000567348 | 1.528871228 |
| MLH3 | -1.79909418 | 4.176608469 | -4.738011664 | 6.67E-05 | 0.000567348 | 1.528247363 |
| OLA1 | 1.102188304 | 8.995349907 | 4.737164647 | 6.69E-05 | 0.000568244 | 1.526068422 |
| PDK3 | -1.724449839 | 7.385539511 | -4.734611646 | 6.73E-05 | 0.000571716 | 1.519500984 |
| BRD2 | 1.270713208 | 12.32493597 | 4.731893031 | 6.78E-05 | 0.00057546 | 1.512507706 |
| SCEL | -2.690576347 | 7.433019562 | -4.731106943 | 6.80E-05 | 0.000576277 | 1.510485638 |
| HRSP12 | -1.773497152 | 7.337952748 | -4.729532108 | 6.83E-05 | 0.000578301 | 1.506434712 |
| COX7B | -1.986524459 | 9.833973419 | -4.728770123 | 6.84E-05 | 0.000579086 | 1.504474695 |
| EMX1 | 2.724125066 | 9.128944942 | 4.727985453 | 6.85E-05 | 0.000579907 | 1.502456341 |
| VAPA | -1.687274466 | 8.186353402 | -4.727557671 | 6.86E-05 | 0.000580181 | 1.501355996 |
| HS2ST1 | 2.490593353 | 13.55945067 | 4.727224701 | 6.87E-05 | 0.000580311 | 1.50049953 |
| HIST1H2BF | 1.796732366 | 11.18359075 | 4.723455355 | 6.94E-05 | 0.000585605 | 1.490804226 |
| NUB1 | -1.325269898 | 7.934089691 | -4.723054951 | 6.94E-05 | 0.000585605 | 1.489774353 |
| C11orf67 | -1.731572706 | 8.561686175 | -4.722987139 | 6.94E-05 | 0.000585605 | 1.489599935 |
| HOXA6 | 3.018044241 | 9.516301093 | 4.722797279 | 6.95E-05 | 0.000585605 | 1.4891116 |
| PDRG1 | -1.419557342 | 6.776005619 | -4.72245592 | 6.95E-05 | 0.00058575 | 1.488233603 |
| BCAS2 | -1.918160052 | 7.825986167 | -4.722073979 | 6.96E-05 | 0.000585958 | 1.487251228 |
| CLDN9 | 2.646205434 | 8.31622761 | 4.721487889 | 6.97E-05 | 0.000586483 | 1.485743781 |
| PRSS16 | -2.329807941 | 5.27182171 | -4.720481073 | 6.99E-05 | 0.000587662 | 1.483154232 |
| KCNE1L | 2.617012323 | 8.153068419 | 4.719998894 | 7.00E-05 | 0.00058771 | 1.481914069 |
| SPACA4 | 2.590496104 | 8.796091397 | 4.719956284 | 7.00E-05 | 0.00058771 | 1.481804475 |
| CENPN | 3.172203016 | 9.817254896 | 4.719592477 | 7.01E-05 | 0.000587767 | 1.480868771 |
| IRAK2 | 2.301266659 | 6.920510151 | 4.719426144 | 7.01E-05 | 0.000587767 | 1.480440966 |
| RAB20 | -1.887448916 | 4.984087983 | -4.718876571 | 7.02E-05 | 0.000588238 | 1.479027483 |
| ZNF426 | -1.538865256 | 6.394754294 | -4.718336685 | 7.03E-05 | 0.000588695 | 1.477638923 |
| HIST1H2AL | 1.926149814 | 6.961798796 | 4.717852653 | 7.04E-05 | 0.000589065 | 1.476394026 |
| FBXL17 | 3.184164138 | 11.10513713 | 4.717177932 | 7.05E-05 | 0.000589733 | 1.4746587 |
| AMN | 2.278592734 | 8.062644843 | 4.714165019 | 7.11E-05 | 0.000593904 | 1.466909902 |
| PFKL | 1.220658915 | 7.661517557 | 4.714020735 | 7.11E-05 | 0.000593904 | 1.46653883 |
| C6orf136 | -1.408296234 | 5.90691648 | -4.711477406 | 7.16E-05 | 0.000597524 | 1.459997962 |
| LOC648771 | -2.258771314 | 13.15871748 | -4.711217682 | 7.16E-05 | 0.000597546 | 1.45933002 |
| RELA | -1.881793866 | 6.62831021 | -4.709334547 | 7.20E-05 | 0.00060014 | 1.454487165 |
| UTP20 | 2.316089758 | 10.58118274 | 4.708911608 | 7.21E-05 | 0.000600233 | 1.453399506 |
| CLIC1 | -1.222116575 | 9.934451253 | -4.708785658 | 7.21E-05 | 0.000600233 | 1.453075608 |
| ASCC1 | -2.031657851 | 5.387114603 | -4.707096946 | 7.24E-05 | 0.000602529 | 1.448732869 |
| CIB2 | 1.699008893 | 7.437863155 | 4.706106546 | 7.26E-05 | 0.000603719 | 1.446185968 |
| RAB3IP | -1.205576593 | 9.589284343 | -4.705134905 | 7.28E-05 | 0.000604365 | 1.443687339 |
| PSMC3 | -1.663751801 | 6.02466386 | -4.705083763 | 7.28E-05 | 0.000604365 | 1.443555825 |
| SH3BP5 | -1.780378793 | 8.425274799 | -4.70496885 | 7.28E-05 | 0.000604365 | 1.443260322 |
| GBA2 | -1.448156318 | 4.866202669 | -4.704127233 | 7.30E-05 | 0.000605321 | 1.441096084 |
| USP13 | -2.354216817 | 5.233104287 | -4.703660786 | 7.31E-05 | 0.000605678 | 1.439896616 |
| HLA-DQB1 | -2.102501521 | 7.533824423 | -4.702297023 | 7.33E-05 | 0.000607474 | 1.436389735 |
| PIGY | -1.121864449 | 9.472420449 | -4.701047808 | 7.36E-05 | 0.000608869 | 1.433177463 |
| SOX21 | 2.668988059 | 8.141441728 | 4.700942856 | 7.36E-05 | 0.000608869 | 1.432907588 |
| RPL12 | 1.468411666 | 14.39118121 | 4.700494473 | 7.37E-05 | 0.000608918 | 1.431754617 |
| AKR1C3 | -2.746446157 | 7.342158857 | -4.700220567 | 7.38E-05 | 0.000608918 | 1.431050297 |
| CD4 | 1.986220734 | 12.5763558 | 4.700183409 | 7.38E-05 | 0.000608918 | 1.430954749 |
| RBM14 | 1.986297873 | 12.4333551 | 4.699003266 | 7.40E-05 | 0.000610429 | 1.427920169 |
| CCND3 | -1.584459656 | 6.833474338 | -4.698612322 | 7.41E-05 | 0.000610669 | 1.426914917 |
| ZBTB4 | -2.605633707 | 9.023535369 | -4.697988021 | 7.42E-05 | 0.000611286 | 1.425309637 |
| ZNF232 | -2.25021374 | 5.143325659 | -4.696269205 | 7.45E-05 | 0.000613677 | 1.420890068 |
| GRSF1 | -1.641125776 | 9.415970844 | -4.695288869 | 7.47E-05 | 0.000614876 | 1.418369384 |
| LOC157627 | 2.35511237 | 7.561781582 | 4.694917614 | 7.48E-05 | 0.000615086 | 1.417414807 |
| FER1L6-AS1 | 2.928913423 | 7.608772357 | 4.694340012 | 7.49E-05 | 0.000615632 | 1.415929673 |
| PTDSS1 | -1.325853558 | 10.14023534 | -4.693378318 | 7.51E-05 | 0.000616806 | 1.413456982 |
| TAS2R4 | 2.835089898 | 7.907140433 | 4.692794444 | 7.52E-05 | 0.000617364 | 1.411955751 |
| RNF213 | 2.032195301 | 9.572183985 | 4.690830762 | 7.56E-05 | 0.000619997 | 1.4069069 |
| SLC35B4 | 1.368894679 | 7.379047678 | 4.690702615 | 7.56E-05 | 0.000619997 | 1.406577423 |
| ZMYND10 | -3.442305612 | 5.107821291 | -4.690422973 | 7.57E-05 | 0.00062006 | 1.405858444 |
| POM121L8P | 2.384457304 | 6.99786719 | 4.689706019 | 7.58E-05 | 0.000620841 | 1.404015115 |
| NKAIN1 | 2.370792893 | 7.448157783 | 4.687522261 | 7.63E-05 | 0.000624039 | 1.398400653 |
| DERL2 | -1.499395103 | 7.166784546 | -4.686284207 | 7.65E-05 | 0.000625511 | 1.395217676 |
| GABPB1 | 2.006380522 | 7.94500535 | 4.686150227 | 7.65E-05 | 0.000625511 | 1.394873225 |
| FKBP15 | -1.785045319 | 6.336483149 | -4.685845893 | 7.66E-05 | 0.000625617 | 1.394090805 |
| GTF2A2 | -1.508032103 | 9.182416477 | -4.685348205 | 7.67E-05 | 0.000626043 | 1.392811295 |
| TAS1R1 | 2.380051655 | 8.318892468 | 4.684970737 | 7.68E-05 | 0.000626271 | 1.391840866 |
| MTHFS | -1.584145106 | 8.658057384 | -4.684076216 | 7.70E-05 | 0.000627356 | 1.389541168 |
| TMTC2 | -1.785038715 | 7.430687487 | -4.682862647 | 7.72E-05 | 0.000628973 | 1.38642128 |
| FKBP4 | 2.511806066 | 9.290917008 | 4.68184046 | 7.74E-05 | 0.000630276 | 1.383793443 |
| PPP6R2 | 2.235071674 | 9.157343704 | 4.678033257 | 7.82E-05 | 0.000635857 | 1.37400621 |
| PSMB6 | -1.590309006 | 8.89873872 | -4.678027573 | 7.82E-05 | 0.000635857 | 1.373991597 |
| OLFML2B | 2.183195737 | 8.848705497 | 4.675809686 | 7.87E-05 | 0.000639192 | 1.368290275 |
| SNCAIP | -1.938635432 | 4.763245204 | -4.67553297 | 7.87E-05 | 0.000639256 | 1.367578958 |
| EPHX3 | 2.796587912 | 12.63597149 | 4.674716517 | 7.89E-05 | 0.000640233 | 1.365480225 |
| LOC100290566 | 2.622358192 | 8.424948597 | 4.671411418 | 7.96E-05 | 0.000645445 | 1.356984547 |
| NUDT19 | -2.391273978 | 4.669774735 | -4.670616102 | 7.98E-05 | 0.000646396 | 1.354940266 |
| POLR1B | -1.89784796 | 5.628280266 | -4.669647835 | 8.00E-05 | 0.000647545 | 1.352451461 |
| SLC37A2 | 1.520952981 | 8.30758267 | 4.669467777 | 8.00E-05 | 0.000647545 | 1.351988647 |
| RYBP | -1.338294757 | 8.900383444 | -4.667991535 | 8.03E-05 | 0.000649668 | 1.348194232 |
| OSTM1 | -1.953674641 | 8.171119151 | -4.66680453 | 8.06E-05 | 0.000651301 | 1.345143302 |
| CR2 | 2.478046887 | 10.23926465 | 4.663686889 | 8.12E-05 | 0.000656278 | 1.337130354 |
| ATP8B2 | 1.137661646 | 9.070689759 | 4.663160215 | 8.13E-05 | 0.000656751 | 1.335776735 |
| KIAA1539 | 1.645983082 | 6.954566515 | 4.662939717 | 8.14E-05 | 0.000656751 | 1.335210029 |
| SUV420H1 | -1.707674405 | 5.46955737 | -4.660683619 | 8.19E-05 | 0.000660001 | 1.329411697 |
| SUGP2 | 1.24332289 | 8.196393407 | 4.66046729 | 8.19E-05 | 0.000660001 | 1.328855727 |
| ZC3HAV1L | 2.298001778 | 9.208531899 | 4.660360971 | 8.19E-05 | 0.000660001 | 1.328582484 |
| EIF3B | -1.135589512 | 8.379469576 | -4.659264483 | 8.22E-05 | 0.000661503 | 1.325764513 |
| APEX1 | -1.604794447 | 7.049322041 | -4.658695421 | 8.23E-05 | 0.000661945 | 1.324302045 |
| RPS19 | -2.029680522 | 11.933542 | -4.658538666 | 8.23E-05 | 0.000661945 | 1.323899191 |
| GPSM3 | 2.246948458 | 9.028470147 | 4.657738752 | 8.25E-05 | 0.000662932 | 1.321843466 |
| PARK7 | -1.478879633 | 11.99115367 | -4.657080019 | 8.27E-05 | 0.000663673 | 1.320150582 |
| TSG101 | -1.394908813 | 9.496921175 | -4.656508076 | 8.28E-05 | 0.000664262 | 1.318680755 |
| GGNBP2 | -1.488446478 | 8.561420604 | -4.653149052 | 8.35E-05 | 0.000669769 | 1.310048698 |
| ARAF | -1.683674383 | 5.125067232 | -4.652843213 | 8.36E-05 | 0.000669893 | 1.309262771 |
| TMEM14C | -1.765442047 | 10.2870285 | -4.652607816 | 8.36E-05 | 0.000669893 | 1.308657863 |
| CAP2 | -1.546994508 | 5.082227336 | -4.65129643 | 8.39E-05 | 0.000671801 | 1.305287994 |
| FGF7 | 1.827259574 | 7.833237652 | 4.650344301 | 8.41E-05 | 0.000673075 | 1.302841345 |
| PDZD4 | 2.403574498 | 7.222743225 | 4.649846986 | 8.43E-05 | 0.000673541 | 1.30156343 |
| FGGY | -2.46770514 | 6.861917679 | -4.646811151 | 8.49E-05 | 0.000678547 | 1.29376266 |
| LRRC8D | -1.183755414 | 6.387541255 | -4.645612356 | 8.52E-05 | 0.000680278 | 1.290682379 |
| MBD3 | 1.203879647 | 7.349575787 | 4.645118697 | 8.53E-05 | 0.000680744 | 1.289413947 |
| FAM113A | 1.731338423 | 7.650318406 | 4.643995737 | 8.56E-05 | 0.000682344 | 1.286528594 |
| IL12RB1 | 2.225494234 | 7.857076791 | 4.642917947 | 8.58E-05 | 0.000683867 | 1.283759349 |
| MIOS | -1.559514671 | 6.05724803 | -4.641364977 | 8.62E-05 | 0.000686255 | 1.279769268 |
| TTC30B | -1.471524031 | 5.472201327 | -4.640467081 | 8.64E-05 | 0.000686726 | 1.277462327 |
| TREML2 | 2.983745058 | 9.265993961 | 4.640419372 | 8.64E-05 | 0.000686726 | 1.27733975 |
| ORC5 | -1.48576298 | 7.248474382 | -4.64040307 | 8.64E-05 | 0.000686726 | 1.277297866 |
| C19orf43 | -1.330172395 | 8.475790673 | -4.639616047 | 8.66E-05 | 0.000687731 | 1.275275817 |
| MYC | 2.547760349 | 10.07625814 | 4.638339705 | 8.69E-05 | 0.000689274 | 1.271996642 |
| XAGE-4 | 4.429734457 | 7.20984825 | 4.63820995 | 8.69E-05 | 0.000689274 | 1.271663279 |
| PTCH2 | 2.536121126 | 8.554297278 | 4.637979159 | 8.69E-05 | 0.000689274 | 1.27107034 |
| APCDD1 | -2.695892927 | 7.085991832 | -4.637835047 | 8.70E-05 | 0.000689274 | 1.270700092 |
| ASH2L | -1.957499941 | 6.909280049 | -4.637471992 | 8.71E-05 | 0.000689511 | 1.269767352 |
| THOP1 | 1.14236546 | 10.84478994 | 4.636086623 | 8.74E-05 | 0.000691615 | 1.266208188 |
| FAM20A | 1.426423618 | 9.320770211 | 4.634614715 | 8.77E-05 | 0.000693884 | 1.262426778 |
| NAB2 | 2.258998927 | 8.750748576 | 4.633461135 | 8.80E-05 | 0.000695284 | 1.259463231 |
| DAB2IP | -1.625535239 | 6.308885414 | -4.633194345 | 8.80E-05 | 0.000695284 | 1.258777854 |
| SSBP1 | -1.743396144 | 7.827259447 | -4.633154566 | 8.81E-05 | 0.000695284 | 1.258675664 |
| PAX7 | 2.306962801 | 8.574444737 | 4.63122759 | 8.85E-05 | 0.000698406 | 1.253725408 |
| STRADB | -1.689701181 | 5.656486555 | -4.630605929 | 8.87E-05 | 0.000699125 | 1.252128439 |
| DNAJC22 | 2.212507297 | 7.130751747 | 4.630216083 | 8.87E-05 | 0.000699417 | 1.251126984 |
| TLE6 | 2.837276538 | 9.167414678 | 4.628358013 | 8.92E-05 | 0.00070243 | 1.246353961 |
| PBX3 | -1.659828163 | 6.390644636 | -4.627671742 | 8.93E-05 | 0.000703275 | 1.244591099 |
| MAP7D3 | 1.988691153 | 8.79437405 | 4.623838818 | 9.03E-05 | 0.000709982 | 1.23474562 |
| C1orf116 | -2.167774277 | 7.953994952 | -4.623617841 | 9.03E-05 | 0.000709982 | 1.234178024 |
| KLK9 | 2.391446083 | 7.903907118 | 4.62136424 | 9.08E-05 | 0.000713788 | 1.228389594 |
| BBS12 | -1.632211559 | 5.817217614 | -4.620950713 | 9.09E-05 | 0.000714132 | 1.227327463 |
| HIST1H3E | 1.660864997 | 7.785595003 | 4.619541206 | 9.13E-05 | 0.00071636 | 1.223707243 |
| CDCA8 | 1.76367891 | 7.42843011 | 4.617863847 | 9.17E-05 | 0.000719104 | 1.219399173 |
| PDCD2 | -1.244041081 | 8.416777419 | -4.617535581 | 9.18E-05 | 0.00071929 | 1.218556081 |
| BAIAP2L2 | 1.580893475 | 7.949903955 | 4.616057816 | 9.21E-05 | 0.000721665 | 1.214760759 |
| NR1D1 | 2.024292481 | 8.516523253 | 4.615430358 | 9.23E-05 | 0.000722423 | 1.213149297 |
| FEN1 | 1.673219992 | 6.695793989 | 4.614856924 | 9.24E-05 | 0.000723078 | 1.211676597 |
| MEF2BNB-MEF2B | 3.030620227 | 9.730346913 | 4.614572782 | 9.25E-05 | 0.000723182 | 1.210946866 |
| HIST1H2BG | 1.869119435 | 8.365628107 | 4.613558026 | 9.27E-05 | 0.000724683 | 1.208340804 |
| RPL30 | -1.846426267 | 12.85575865 | -4.612670278 | 9.30E-05 | 0.000725854 | 1.206060954 |
| COMMD6 | -1.383541715 | 12.04541869 | -4.612487999 | 9.30E-05 | 0.000725854 | 1.205592843 |
| KLHDC7B | 3.231774695 | 8.139546026 | 4.610868404 | 9.34E-05 | 0.000728525 | 1.201433622 |
| LOC399851 | 2.433036979 | 9.344860605 | 4.609615556 | 9.37E-05 | 0.000730499 | 1.198216305 |
| MUC6 | 2.412967696 | 9.008465396 | 4.609068272 | 9.38E-05 | 0.000731113 | 1.196810902 |
| PRDM1 | 2.018080068 | 8.448969199 | 4.608138358 | 9.41E-05 | 0.000732468 | 1.194422946 |
| WDR45 | -1.902829445 | 7.233711933 | -4.607766868 | 9.42E-05 | 0.000732744 | 1.193468998 |
| LINC00482 | 2.324104067 | 10.12953674 | 4.607352828 | 9.43E-05 | 0.000733103 | 1.192405791 |
| SLIT1 | 1.968696836 | 8.961694673 | 4.606727471 | 9.44E-05 | 0.000733872 | 1.190799961 |
| PARP4 | -1.467022115 | 4.811735317 | -4.606295257 | 9.45E-05 | 0.000734267 | 1.189690107 |
| MTX2 | -1.318861167 | 7.380387292 | -4.604352075 | 9.50E-05 | 0.000737602 | 1.184700431 |
| GNG4 | 1.641562223 | 6.367147173 | 4.602578565 | 9.55E-05 | 0.000740621 | 1.180146586 |
| MAP6 | -2.174671941 | 5.18514786 | -4.602203516 | 9.56E-05 | 0.000740908 | 1.17918359 |
| MANBA | -1.410673606 | 6.989483415 | -4.601762989 | 9.57E-05 | 0.000741324 | 1.178052475 |
| CYB561D1 | 2.911603438 | 10.68969424 | 4.59998206 | 9.61E-05 | 0.000744373 | 1.173479784 |
| EME1 | 1.802930414 | 5.879939071 | 4.599476527 | 9.63E-05 | 0.00074492 | 1.172181811 |
| PPM1A | -1.308451841 | 8.597108167 | -4.598783509 | 9.64E-05 | 0.000745837 | 1.170402482 |
| DHX16 | -1.517772877 | 6.594040921 | -4.597579676 | 9.67E-05 | 0.000747764 | 1.16731168 |
| HIRA | -1.520850686 | 8.031389423 | -4.59703603 | 9.69E-05 | 0.000748389 | 1.16591591 |
| ICOSLG | 2.682989154 | 9.502417393 | 4.596129905 | 9.71E-05 | 0.000749733 | 1.163589531 |
| POM121L4P | 1.689303107 | 6.249466494 | 4.594163203 | 9.76E-05 | 0.000752199 | 1.15854036 |
| C17orf61 | -1.506331338 | 9.596860257 | -4.594118391 | 9.76E-05 | 0.000752199 | 1.158425314 |
| OR6K2 | 2.359907137 | 7.114762788 | 4.594112053 | 9.76E-05 | 0.000752199 | 1.158409044 |
| PTF1A | 2.568516189 | 7.960981395 | 4.593980132 | 9.77E-05 | 0.000752199 | 1.158070366 |
| CNTLN | 1.494593508 | 9.232116642 | 4.593632511 | 9.78E-05 | 0.000752439 | 1.157177931 |
| SERPINA6 | 2.985349934 | 10.23838648 | 4.592706428 | 9.80E-05 | 0.000753832 | 1.154800453 |
| ENTPD2 | 2.100717318 | 7.743629433 | 4.592130654 | 9.81E-05 | 0.000754528 | 1.153322326 |
| TMUB1 | 1.978850598 | 9.52011834 | 4.590560168 | 9.86E-05 | 0.000757214 | 1.149290644 |
| EIF3L | -2.326900375 | 8.772469045 | -4.5888883 | 9.90E-05 | 0.000760113 | 1.144998827 |
| TEAD4 | -1.699039153 | 5.47134149 | -4.587486421 | 9.94E-05 | 0.000762478 | 1.141400194 |
| EIF5 | -1.513155359 | 9.133502524 | -4.586365882 | 9.97E-05 | 0.000764283 | 1.138523828 |
| LPAR1 | -1.671660848 | 5.16943263 | -4.585743829 | 9.98E-05 | 0.000765085 | 1.136927073 |
| KCNQ2 | 2.756533871 | 9.226207375 | 4.584371335 | 0.000100177 | 0.000767212 | 1.13340407 |
| HAVCR1 | 2.924812303 | 8.586771043 | 4.583899266 | 0.000100302 | 0.000767212 | 1.132192355 |
| C16orf11 | 1.757974416 | 8.459649756 | 4.583833741 | 0.000100319 | 0.000767212 | 1.132024166 |
| PPP3CA | -1.293669916 | 8.715865843 | -4.583791737 | 0.000100331 | 0.000767212 | 1.131916349 |
| LEPRE1 | 1.611855464 | 8.916871244 | 4.582346409 | 0.000100714 | 0.000769691 | 1.128206529 |
| DCAF7 | -1.309270867 | 6.237304447 | -4.581448076 | 0.000100954 | 0.000771062 | 1.125900766 |
| SF3A2 | 3.481035242 | 12.79638206 | 4.580985272 | 0.000101077 | 0.000771547 | 1.124712895 |
| PURA | -1.724907159 | 8.526005455 | -4.580564787 | 0.00010119 | 0.000771947 | 1.123633653 |
| ECHS1 | -2.615511299 | 8.031936043 | -4.580210953 | 0.000101284 | 0.000772211 | 1.122725489 |
| LYZL1 | 2.528282408 | 9.078664078 | 4.579781883 | 0.000101399 | 0.000772629 | 1.121624229 |
| SCNM1 | 1.198462434 | 11.13200258 | 4.579086799 | 0.000101586 | 0.000773591 | 1.119840228 |
| GAD1 | 2.282849406 | 7.184119595 | 4.578321383 | 0.000101791 | 0.000774261 | 1.117875742 |
| CHST13 | 2.214940849 | 8.566727502 | 4.578168435 | 0.000101832 | 0.000774261 | 1.117483194 |
| C5orf43 | -1.543051203 | 7.134900796 | -4.578073644 | 0.000101858 | 0.000774261 | 1.117239911 |
| CEP112 | -1.762120034 | 5.371845846 | -4.577863781 | 0.000101914 | 0.000774261 | 1.116701291 |
| PRPF31 | -1.489376526 | 7.440338754 | -4.577498588 | 0.000102013 | 0.000774551 | 1.115764019 |
| WIF1 | -4.534862135 | 7.926295574 | -4.576855095 | 0.000102186 | 0.000775412 | 1.114112502 |
| TCIRG1 | 2.329795794 | 12.17626221 | 4.576584983 | 0.000102259 | 0.000775508 | 1.11341927 |
| PAQR5 | -2.077575219 | 5.134222988 | -4.575046821 | 0.000102676 | 0.000778207 | 1.109471696 |
| KLF9 | -2.633523961 | 9.502691083 | -4.574202029 | 0.000102905 | 0.000779487 | 1.107303654 |
| ZNF500 | -2.040015933 | 6.239674565 | -4.573906398 | 0.000102986 | 0.000779637 | 1.106544965 |
| ACPL2 | -1.880971009 | 5.740243808 | -4.573226003 | 0.000103171 | 0.00078058 | 1.104798857 |
| ZNF630 | 2.517963778 | 9.723397531 | 4.572675692 | 0.000103321 | 0.000780925 | 1.103386605 |
| COX7A2L | -1.495184556 | 9.472604007 | -4.572613996 | 0.000103338 | 0.000780925 | 1.103228274 |
| PEX11B | -2.485719925 | 6.438255486 | -4.571510057 | 0.00010364 | 0.0007824 | 1.100395303 |
| PCBP4 | 1.52485109 | 11.06988104 | 4.571455029 | 0.000103655 | 0.0007824 | 1.100254089 |
| USP25 | -1.077366248 | 8.48558021 | -4.57108994 | 0.000103755 | 0.000782555 | 1.099317197 |
| LUC7L2 | -1.863919259 | 6.644728848 | -4.570936334 | 0.000103797 | 0.000782555 | 1.098923015 |
| LOC151534 | 2.502213156 | 9.077820007 | 4.569271187 | 0.000104255 | 0.000785545 | 1.094650006 |
| C16orf7 | 2.381368921 | 10.16283412 | 4.567435168 | 0.000104762 | 0.000788903 | 1.089938676 |
| TMEM222 | -1.680467882 | 5.576347714 | -4.565480069 | 0.000105304 | 0.000792332 | 1.08492196 |
| PACS1 | 1.051554135 | 8.987537428 | 4.565350891 | 0.00010534 | 0.000792332 | 1.084590501 |
| GFRA2 | 2.523495908 | 8.169599285 | 4.564256911 | 0.000105645 | 0.000794161 | 1.081783474 |
| KCTD9 | -1.456376308 | 7.21328462 | -4.563016493 | 0.000105992 | 0.000796302 | 1.07860078 |
| HIST2H3D | 1.617115294 | 10.07409105 | 4.562412641 | 0.000106161 | 0.000797108 | 1.07705143 |
| IL3RA | 2.159113773 | 9.789761665 | 4.561951771 | 0.000106291 | 0.000797543 | 1.075868951 |
| SGK3 | -2.336054697 | 6.215064561 | -4.561738716 | 0.00010635 | 0.000797543 | 1.075322309 |
| CDC45 | 2.248750071 | 7.756365002 | 4.561543587 | 0.000106405 | 0.000797543 | 1.074821662 |
| SLC38A9 | -1.274718438 | 7.506293831 | -4.560315658 | 0.000106751 | 0.000798957 | 1.071671178 |
| KRT31 | 2.301747019 | 8.12993361 | 4.560226599 | 0.000106776 | 0.000798957 | 1.071442682 |
| VPS41 | -2.140104929 | 6.159436335 | -4.560212077 | 0.00010678 | 0.000798957 | 1.071405423 |
| CXorf36 | 2.297325532 | 10.43832291 | 4.559300446 | 0.000107038 | 0.000800294 | 1.069066515 |
| FOXJ3 | -2.090031896 | 8.26681349 | -4.559139267 | 0.000107083 | 0.000800294 | 1.068652993 |
| CDK5R2 | 2.256010488 | 8.38680167 | 4.558611527 | 0.000107233 | 0.000800945 | 1.067299033 |
| IL28A | 2.661466634 | 8.132613125 | 4.557812853 | 0.000107459 | 0.000802088 | 1.065249993 |
| UHRF2 | -1.691513557 | 8.696237205 | -4.557632265 | 0.000107511 | 0.000802088 | 1.064786688 |
| CEACAM19 | 2.107257566 | 9.831237951 | 4.556624716 | 0.000107797 | 0.00080376 | 1.062201824 |
| EIF5A | 1.438625393 | 9.093989965 | 4.556053333 | 0.00010796 | 0.000804508 | 1.060735966 |
| ITPKC | 1.360000668 | 9.924504492 | 4.555589548 | 0.000108092 | 0.000805028 | 1.059546157 |
| GPS2 | -1.446309564 | 4.592562903 | -4.555249262 | 0.00010819 | 0.000805286 | 1.058673185 |
| ZBTB22 | 2.443792095 | 12.24192116 | 4.554691725 | 0.000108349 | 0.000806006 | 1.057242886 |
| MAPKAPK3 | -1.372607862 | 7.096772774 | -4.55268455 | 0.000108925 | 0.000809823 | 1.052093836 |
| CHCHD7 | -1.541919592 | 8.061083436 | -4.552204757 | 0.000109063 | 0.000810382 | 1.050863042 |
| GPR35 | 2.272238326 | 8.311988736 | 4.549004226 | 0.000109989 | 0.000816791 | 1.042653159 |
| TUB | 1.830252013 | 4.460578759 | 4.548777493 | 0.000110055 | 0.000816809 | 1.04207157 |
| LOC728978 | 1.963914649 | 8.46715541 | 4.548520067 | 0.00011013 | 0.000816893 | 1.041411258 |
| PTRHD1 | -2.517351729 | 6.098519075 | -4.54821166 | 0.00011022 | 0.00081703 | 1.040620178 |
| SMPD1 | -1.244282605 | 5.53420905 | -4.548020201 | 0.000110276 | 0.00081703 | 1.04012908 |
| MAPKBP1 | 1.478428129 | 8.613770735 | 4.546917417 | 0.000110597 | 0.000818942 | 1.037300439 |
| PLCB2 | 2.65540215 | 7.830860665 | 4.546245187 | 0.000110794 | 0.000819925 | 1.035576199 |
| SV2A | 1.987795638 | 7.464548118 | 4.545991424 | 0.000110868 | 0.000820003 | 1.034925313 |
| OR2H1 | 2.582499933 | 9.445255577 | 4.545192147 | 0.000111102 | 0.000821264 | 1.032875247 |
| TUBA3D | 3.289499501 | 10.70557906 | 4.544540252 | 0.000111294 | 0.000822109 | 1.031203225 |
| NDUFB9 | -1.480438258 | 12.49418871 | -4.544368293 | 0.000111345 | 0.000822109 | 1.030762176 |
| REEP5 | -1.321091167 | 10.34293042 | -4.544075294 | 0.000111431 | 0.000822273 | 1.030010683 |
| MAMDC4 | 1.78326644 | 7.284842926 | 4.542971704 | 0.000111756 | 0.000824201 | 1.027180203 |
| OR10P1 | 2.687143615 | 7.400693982 | 4.541988232 | 0.000112047 | 0.000825871 | 1.024657853 |
| CD300LB | 2.915446222 | 8.53549774 | 4.541141644 | 0.000112298 | 0.000827246 | 1.022486617 |
| BMPR1A | -1.176231645 | 4.945223691 | -4.540695882 | 0.00011243 | 0.000827747 | 1.021343392 |
| ANXA1 | -1.791609973 | 11.67776868 | -4.537320114 | 0.000113437 | 0.000834683 | 1.012686049 |
| LOC388387 | 2.752229252 | 7.909616032 | 4.535799292 | 0.000113894 | 0.000837564 | 1.008786016 |
| PMPCA | -1.205367578 | 7.036557769 | -4.53382127 | 0.00011449 | 0.00084147 | 1.003713715 |
| WFDC3 | 1.742897671 | 6.814631746 | 4.532649151 | 0.000114845 | 0.000843597 | 1.000708116 |
| DNAJA1 | -1.565642453 | 10.02843836 | -4.53040101 | 0.000115529 | 0.000848137 | 0.99494354 |
| RHCG | 2.569769745 | 10.34703112 | 4.52979619 | 0.000115714 | 0.000849009 | 0.993392735 |
| C20orf144 | 2.569600829 | 7.949020127 | 4.528932395 | 0.000115978 | 0.000850124 | 0.991177935 |
| FBXL3 | -2.020424362 | 7.627300339 | -4.528867254 | 0.000115998 | 0.000850124 | 0.991010913 |
| GJB3 | 2.405181267 | 8.181203836 | 4.527743747 | 0.000116343 | 0.000852165 | 0.988130267 |
| NRSN2 | 1.8877174 | 7.06221957 | 4.527189966 | 0.000116513 | 0.000852927 | 0.98671041 |
| ZFAND2B | 1.177971293 | 9.142673398 | 4.524880509 | 0.000117226 | 0.000857657 | 0.980789303 |
| FAM107A | -2.300561704 | 9.353731919 | -4.523619699 | 0.000117617 | 0.000860029 | 0.977556898 |
| ARAP2 | -2.082036591 | 7.457419577 | -4.521726001 | 0.000118207 | 0.00086385 | 0.97270209 |
| SMYD5 | 2.454242081 | 9.200964442 | 4.520651565 | 0.000118543 | 0.000865813 | 0.969947682 |
| ENPP1 | -1.739349316 | 6.341385133 | -4.518378285 | 0.000119257 | 0.000870532 | 0.964120153 |
| MRPL22 | -1.691213817 | 8.333798558 | -4.518002096 | 0.000119375 | 0.000870903 | 0.963155822 |
| CELA2B | 2.451703862 | 8.244599262 | 4.517652467 | 0.000119485 | 0.000871213 | 0.962259585 |
| NDUFA2 | -1.33765796 | 10.70268834 | -4.516996232 | 0.000119693 | 0.00087223 | 0.960577416 |
| TMC8 | 2.227365649 | 9.649862589 | 4.516306603 | 0.000119911 | 0.000873325 | 0.95880967 |
| DCST1 | 2.485014232 | 7.585213642 | 4.51480039 | 0.000120389 | 0.00087631 | 0.954948842 |
| LOC100653245 | 2.861432393 | 10.42055556 | 4.513322929 | 0.000120859 | 0.000878859 | 0.951161839 |
| CIC | -1.343817348 | 7.557957247 | -4.513098489 | 0.000120931 | 0.000878859 | 0.950586569 |
| LOC728175 | 1.629564185 | 6.638751795 | 4.513057921 | 0.000120944 | 0.000878859 | 0.950482589 |
| PIM1 | 2.204755703 | 11.54356927 | 4.511401133 | 0.000121474 | 0.000882215 | 0.946236111 |
| SH3KBP1 | 1.211320373 | 9.396930302 | 4.510763193 | 0.000121679 | 0.000883203 | 0.944601063 |
| PARP8 | -1.890489563 | 5.559455973 | -4.508778942 | 0.000122318 | 0.000887343 | 0.939515553 |
| KLHL28 | 3.023468092 | 9.510700123 | 4.508419896 | 0.000122434 | 0.000887684 | 0.938595364 |
| CLCN6 | 2.004681704 | 7.416021062 | 4.506993178 | 0.000122897 | 0.000890532 | 0.934938943 |
| UTF1 | 2.370903048 | 7.700734523 | 4.504212491 | 0.000123802 | 0.000895037 | 0.927812885 |
| MAFA | 2.508566575 | 7.127575377 | 4.50400439 | 0.00012387 | 0.000895037 | 0.927279602 |
| SYT11 | 1.637122158 | 6.946494297 | 4.503892691 | 0.000123907 | 0.000895037 | 0.926993362 |
| DNAJA2 | -1.480478517 | 5.740211534 | -4.503646788 | 0.000123987 | 0.000895037 | 0.926363213 |
| CPPED1 | -1.732439261 | 6.148667015 | -4.503629634 | 0.000123993 | 0.000895037 | 0.926319255 |
| SLC5A3 | 1.68324579 | 7.968645764 | 4.503598303 | 0.000124003 | 0.000895037 | 0.926238966 |
| MYBPC2 | 2.345644689 | 7.652673316 | 4.503591926 | 0.000124005 | 0.000895037 | 0.926222625 |
| DPY30 | -1.92394198 | 8.054705125 | -4.502725242 | 0.000124289 | 0.000896264 | 0.924001698 |
| GNL1 | -1.474379121 | 5.832181691 | -4.502648328 | 0.000124315 | 0.000896264 | 0.923804605 |
| PECAM1 | -1.793039567 | 11.42127623 | -4.50224335 | 0.000124448 | 0.000896365 | 0.922766842 |
| LONRF2 | -2.542847864 | 5.639463365 | -4.502181265 | 0.000124468 | 0.000896365 | 0.92260775 |
| IQSEC2 | 1.660828204 | 10.66714425 | 4.501969388 | 0.000124538 | 0.000896365 | 0.922064817 |
| HSCB | -1.293818444 | 6.413813107 | -4.499950665 | 0.000125203 | 0.000900652 | 0.916891987 |
| PSMD5 | -1.068905668 | 6.371517928 | -4.499086057 | 0.000125489 | 0.000902206 | 0.914676566 |
| RG9MTD2 | -1.538474324 | 5.490037841 | -4.497956072 | 0.000125864 | 0.000904396 | 0.911781225 |
| THAP6 | -1.744162026 | 4.743547071 | -4.497275428 | 0.000126091 | 0.000905517 | 0.910037258 |
| BCKDHB | -2.216539764 | 5.60777987 | -4.496627357 | 0.000126307 | 0.000906561 | 0.908376776 |
| RNASEH2A | 1.566613325 | 8.670162769 | 4.49620723 | 0.000126447 | 0.000907061 | 0.907300344 |
| ZBED5 | -1.733500969 | 8.587764173 | -4.49464885 | 0.000126968 | 0.000910294 | 0.903307616 |
| DPEP2 | -1.824324003 | 5.363731964 | -4.494140288 | 0.000127139 | 0.000911009 | 0.902004662 |
| PYGM | 1.991469002 | 7.5789801 | 4.493727287 | 0.000127278 | 0.000911495 | 0.900946548 |
| C9orf62 | 2.404511931 | 10.24766746 | 4.493439481 | 0.000127374 | 0.00091168 | 0.900209191 |
| USP35 | 1.297118148 | 9.912594892 | 4.492590167 | 0.00012766 | 0.000913219 | 0.898033287 |
| FLCN | 1.660967543 | 8.471241235 | 4.491281485 | 0.000128102 | 0.000914372 | 0.894680585 |
| GRK5 | -2.055052875 | 10.68715726 | -4.491244014 | 0.000128115 | 0.000914372 | 0.894584589 |
| DUSP7 | 2.201740327 | 8.415252545 | 4.491202082 | 0.000128129 | 0.000914372 | 0.894477166 |
| CCT6A | -1.444552702 | 8.600134722 | -4.491114524 | 0.000128159 | 0.000914372 | 0.894252856 |
| LINC00483 | 2.402455311 | 8.039956387 | 4.491060288 | 0.000128177 | 0.000914372 | 0.894113912 |
| F13A1 | 1.228582743 | 11.2082486 | 4.490140531 | 0.000128489 | 0.000916087 | 0.891757668 |
| LILRP2 | 1.814728442 | 7.383747164 | 4.489640818 | 0.000128658 | 0.000916789 | 0.890477518 |
| MLH1 | -1.595063358 | 10.08821849 | -4.48895467 | 0.000128892 | 0.000917942 | 0.888719789 |
| VWA1 | 1.300976253 | 11.38864471 | 4.487770907 | 0.000129295 | 0.000920042 | 0.885687371 |
| USE1 | -1.528847868 | 5.756834823 | -4.487669888 | 0.00012933 | 0.000920042 | 0.885428597 |
| SIPA1 | -1.665056482 | 6.973140223 | -4.487144737 | 0.000129509 | 0.000920404 | 0.884083359 |
| PTPRO | -1.992407459 | 5.221816455 | -4.487102429 | 0.000129523 | 0.000920404 | 0.883974983 |
| CALML3 | 2.557090499 | 10.89222893 | 4.486489462 | 0.000129733 | 0.0009213 | 0.882404817 |
| LINC00167 | 2.136746149 | 7.549996453 | 4.486315354 | 0.000129793 | 0.0009213 | 0.881958829 |
| CXXC5 | 1.214331754 | 9.840809499 | 4.485720013 | 0.000129997 | 0.00092224 | 0.880433842 |
| SMCR5 | 2.524250214 | 9.573316117 | 4.485101309 | 0.00013021 | 0.000923239 | 0.878849033 |
| C16orf86 | 2.457074063 | 7.76615789 | 4.483936095 | 0.000130611 | 0.000925573 | 0.875864403 |
| MAK16 | -2.338608779 | 5.86001564 | -4.483362164 | 0.000130809 | 0.000925725 | 0.87439434 |
| MC1R | 1.79154432 | 8.083550067 | 4.483312331 | 0.000130826 | 0.000925725 | 0.874266701 |
| HIST1H2BM | 1.540917538 | 9.451838851 | 4.483149897 | 0.000130882 | 0.000925725 | 0.873850648 |
| CAMK2N1 | -1.687083995 | 9.650780607 | -4.483039752 | 0.00013092 | 0.000925725 | 0.873568527 |
| CIITA | 2.008758553 | 9.51265739 | 4.482594509 | 0.000131074 | 0.000926305 | 0.872428109 |
| ADAM33 | 1.96816982 | 7.34199816 | 4.482331795 | 0.000131165 | 0.000926439 | 0.871755213 |
| HRH2 | 2.844040936 | 8.270080829 | 4.481227653 | 0.000131548 | 0.000928633 | 0.8689272 |
| IL17RE | 3.041675297 | 10.48706479 | 4.481015978 | 0.000131621 | 0.000928642 | 0.868385051 |
| SLC7A5 | 1.720145275 | 6.404252602 | 4.480214767 | 0.0001319 | 0.000930098 | 0.866332986 |
| C20orf194 | -1.8613129 | 5.10629823 | -4.479400876 | 0.000132184 | 0.000931588 | 0.864248484 |
| TMED10P1 | 3.99282271 | 8.310936384 | 4.475870951 | 0.000133421 | 0.000939795 | 0.855208273 |
| KIAA1656 | 2.667877548 | 6.9918239 | 4.475006767 | 0.000133726 | 0.000941425 | 0.852995196 |
| IFITM2 | -2.047835182 | 12.75694645 | -4.47474164 | 0.00013382 | 0.000941569 | 0.852316246 |
| CRABP1 | 3.528069037 | 10.9312474 | 4.47442388 | 0.000133932 | 0.000941844 | 0.851502517 |
| SRC | 2.011454573 | 10.59556592 | 4.473732781 | 0.000134177 | 0.000943046 | 0.849732749 |
| PPP1CC | -1.737857699 | 8.616992233 | -4.473526665 | 0.00013425 | 0.000943046 | 0.849204934 |
| ATN1 | 2.16649292 | 13.90074172 | 4.47219106 | 0.000134724 | 0.000945241 | 0.845784818 |
| SPSB4 | 1.903765194 | 10.51485768 | 4.472094245 | 0.000134758 | 0.000945241 | 0.845536908 |
| PCGF6 | -2.207702221 | 5.535433215 | -4.472025652 | 0.000134782 | 0.000945241 | 0.845361263 |
| BPIFA3 | 2.709572442 | 7.216733049 | 4.469967673 | 0.000135517 | 0.000949872 | 0.840091588 |
| LOC149703 | 2.304320483 | 8.209459515 | 4.46796508 | 0.000136235 | 0.000954138 | 0.83496399 |
| PRPSAP1 | -2.269341416 | 7.627142949 | -4.467857221 | 0.000136274 | 0.000954138 | 0.834687829 |
| XRCC6BP1 | -1.560139729 | 5.444334553 | -4.466347264 | 0.000136818 | 0.000957404 | 0.830821792 |
| SPATA2L | 2.077371379 | 10.9860367 | 4.465770407 | 0.000137026 | 0.000957404 | 0.82934487 |
| NAPSA | -2.721996237 | 9.596961604 | -4.465765703 | 0.000137028 | 0.000957404 | 0.829332826 |
| NOC2L | 1.390718546 | 8.461828931 | 4.465738427 | 0.000137038 | 0.000957404 | 0.82926299 |
| IFT43 | -1.338814306 | 8.373266724 | -4.465314725 | 0.000137191 | 0.000957955 | 0.828178205 |
| ALG6 | -1.742846291 | 5.149094814 | -4.464862147 | 0.000137355 | 0.00095858 | 0.827019499 |
| PHOSPHO2 | -1.681793733 | 6.030798181 | -4.464481545 | 0.000137493 | 0.000959023 | 0.826045081 |
| SFXN5 | 2.187431148 | 8.930196057 | 4.463814522 | 0.000137736 | 0.000959452 | 0.824337387 |
| INF2 | 1.501873058 | 7.990303639 | 4.463791625 | 0.000137744 | 0.000959452 | 0.824278765 |
| TOM1L2 | 3.389950125 | 11.92692455 | 4.463696033 | 0.000137779 | 0.000959452 | 0.824034036 |
| SHISA5 | 1.826206515 | 12.95716088 | 4.463458393 | 0.000137865 | 0.000959535 | 0.823425646 |
| RUNX2 | 1.223908043 | 6.639454722 | 4.463061274 | 0.00013801 | 0.000960022 | 0.822408974 |
| USP7 | -1.415894062 | 7.950649613 | -4.462115654 | 0.000138355 | 0.0009619 | 0.819988118 |
| CHAMP1 | -1.910652262 | 6.393278837 | -4.460856557 | 0.000138815 | 0.000964581 | 0.816764827 |
| MMP19 | 2.017304103 | 9.840164967 | 4.460325146 | 0.00013901 | 0.000965413 | 0.815404445 |
| STEAP1 | 2.280708596 | 8.40611351 | 4.457343167 | 0.000140108 | 0.000972516 | 0.807771082 |
| MOCS2 | -1.53959511 | 7.079824261 | -4.456873093 | 0.000140282 | 0.000973198 | 0.806567825 |
| IRAK1 | 1.298757291 | 6.774656364 | 4.455886842 | 0.000140648 | 0.000975208 | 0.804043347 |
| DOK3 | 1.739859748 | 9.175996988 | 4.455658131 | 0.000140733 | 0.000975271 | 0.803457929 |
| ILK | -2.152013316 | 8.610884984 | -4.454853168 | 0.000141032 | 0.000976819 | 0.801397546 |
| COL1A1 | 4.273228228 | 11.8541851 | 4.454330709 | 0.000141227 | 0.000977089 | 0.800060285 |
| NUP153 | -1.922873223 | 7.562522875 | -4.454075516 | 0.000141322 | 0.000977089 | 0.79940711 |
| TXNL4B | -1.646311243 | 4.739264732 | -4.454056746 | 0.000141329 | 0.000977089 | 0.799359068 |
| PLCH2 | 1.696376809 | 7.122152859 | 4.45393302 | 0.000141375 | 0.000977089 | 0.799042389 |
| CRMP1 | -1.845255444 | 4.965500906 | -4.45336223 | 0.000141588 | 0.000978036 | 0.797581457 |
| ZNF331 | -2.176951326 | 7.126697674 | -4.452404644 | 0.000141946 | 0.000979985 | 0.795130568 |
| BUD31 | -1.397408195 | 7.771806708 | -4.451713608 | 0.000142205 | 0.000981046 | 0.793361937 |
| LRRC8B | -1.735541026 | 5.384272441 | -4.451588089 | 0.000142252 | 0.000981046 | 0.793040688 |
| UCN2 | 2.883074751 | 11.15763815 | 4.451339786 | 0.000142346 | 0.000981163 | 0.792405194 |
| DUSP15 | 2.422214339 | 8.039219351 | 4.451068785 | 0.000142447 | 0.000981339 | 0.791711612 |
| PLEKHJ1 | 1.663091263 | 9.971608636 | 4.450770611 | 0.00014256 | 0.000981585 | 0.790948492 |
| U2AF2 | 1.227890486 | 7.678817026 | 4.450482177 | 0.000142668 | 0.000981807 | 0.790210303 |
| TPD52 | -1.397393214 | 7.469102334 | -4.449671937 | 0.000142974 | 0.00098298 | 0.78813669 |
| MRE11A | 1.191285289 | 8.358915565 | 4.449446309 | 0.000143059 | 0.00098298 | 0.787559256 |
| IL9R | 2.053289873 | 7.564786015 | 4.449421964 | 0.000143068 | 0.00098298 | 0.787496951 |
| GSTO1 | -2.785481689 | 9.641826905 | -4.448719044 | 0.000143333 | 0.000984279 | 0.785698043 |
| NDUFA13 | -1.847258189 | 8.650596376 | -4.447233978 | 0.000143896 | 0.000987617 | 0.78189758 |
| CPNE2 | -1.464304486 | 7.813507946 | -4.445403842 | 0.000144593 | 0.000991868 | 0.777214242 |
| C14orf109 | -1.619016088 | 6.199157612 | -4.445175153 | 0.00014468 | 0.000991938 | 0.776629042 |
| AFG3L2 | -1.597015522 | 5.35853538 | -4.444655624 | 0.000144879 | 0.00099277 | 0.775299608 |
| PRMT2 | -1.421136041 | 6.600338513 | -4.443311011 | 0.000145394 | 0.000995768 | 0.771858941 |
| ROM1 | 1.415567869 | 9.315371317 | 4.442886255 | 0.000145557 | 0.000996354 | 0.770772077 |
| HMBOX1 | 2.815131075 | 12.93569187 | 4.441454761 | 0.000146107 | 0.000999592 | 0.767109265 |
| DNPEP | -1.181545739 | 6.103239483 | -4.440692679 | 0.000146402 | 0.001001072 | 0.765159356 |
| MRPL50 | -1.567242714 | 7.212095209 | -4.439675623 | 0.000146795 | 0.001003229 | 0.762557116 |
| DYNLT1 | -1.328259849 | 9.904724865 | -4.439246088 | 0.000146961 | 0.001003557 | 0.761458128 |
| INTS10 | -2.064969058 | 8.961542413 | -4.439149009 | 0.000146999 | 0.001003557 | 0.761209748 |
| FBLN5 | -1.723237817 | 9.171866869 | -4.438557136 | 0.000147229 | 0.001004592 | 0.759695436 |
| PIGR | -3.114156715 | 9.768722794 | -4.438233055 | 0.000147355 | 0.001004919 | 0.758866282 |
| BCL2 | 2.341543996 | 9.801099299 | 4.437592727 | 0.000147604 | 0.001006084 | 0.757228035 |
| PAM | -1.683961056 | 10.35802091 | -4.436943328 | 0.000147857 | 0.001007276 | 0.755566611 |
| MYCT1 | -1.664835301 | 6.637851263 | -4.435443967 | 0.000148443 | 0.001010733 | 0.751730751 |
| MRPL15 | -1.596338397 | 7.676073413 | -4.435226217 | 0.000148528 | 0.001010779 | 0.751173686 |
| MLYCD | -1.757366972 | 8.092480396 | -4.434282623 | 0.000148899 | 0.001012762 | 0.748759754 |
| C20orf85 | -2.770895612 | 9.334327439 | -4.432629948 | 0.000149549 | 0.00101665 | 0.744531974 |
| TLN2 | 1.59214358 | 7.795341243 | 4.431087716 | 0.000150159 | 0.001020256 | 0.740586892 |
| LOC100128079 | 2.224382167 | 8.895316687 | 4.43074295 | 0.000150296 | 0.001020645 | 0.739704991 |
| RPL8 | -1.584138277 | 12.12526288 | -4.430326717 | 0.000150461 | 0.001021227 | 0.73864029 |
| RPS7P5 | -1.438521629 | 9.577901867 | -4.430074254 | 0.000150561 | 0.001021369 | 0.737994509 |
| NTRK2 | 2.563542734 | 8.546540385 | 4.429868467 | 0.000150643 | 0.001021385 | 0.737468126 |
| TBX4 | -1.439389478 | 6.383310394 | -4.429324287 | 0.000150859 | 0.001022256 | 0.73607618 |
| EEF1G | -1.729421322 | 10.97638022 | -4.429145826 | 0.00015093 | 0.001022256 | 0.735619702 |
| MAP2K1 | -1.99968063 | 6.955061957 | -4.427756246 | 0.000151485 | 0.00102547 | 0.732065434 |
| SRP14 | -1.779596718 | 12.99753211 | -4.426317059 | 0.000152061 | 0.00102883 | 0.728384424 |
| SPICE1 | -1.338226109 | 5.925566711 | -4.425473605 | 0.000152399 | 0.001030579 | 0.726227188 |
| EGFL8 | 1.455085951 | 8.776413593 | 4.42490164 | 0.00015263 | 0.001031593 | 0.724764345 |
| MCMBP | -1.673839218 | 7.759389875 | -4.424540775 | 0.000152775 | 0.001032033 | 0.723841417 |
| PRR7 | 1.814885983 | 8.617854046 | 4.423888745 | 0.000153038 | 0.001033267 | 0.722173848 |
| LATS2 | -1.134045817 | 6.295422279 | -4.423176428 | 0.000153326 | 0.001034172 | 0.720352127 |
| THBS1 | 2.172255159 | 10.31653297 | 4.423077119 | 0.000153366 | 0.001034172 | 0.72009815 |
| ZNF187 | -1.564758591 | 5.201971812 | -4.422960401 | 0.000153413 | 0.001034172 | 0.719799653 |
| HNRNPR | -2.190627167 | 7.802613368 | -4.422703162 | 0.000153517 | 0.001034332 | 0.719141787 |
| HNRNPH3 | -1.731486521 | 7.635301828 | -4.421055133 | 0.000154186 | 0.001038294 | 0.714927214 |
| SCNN1A | -2.126521391 | 7.515753493 | -4.420468185 | 0.000154425 | 0.001039044 | 0.713426232 |
| ZNF91 | -1.761348226 | 8.205052004 | -4.420384733 | 0.000154459 | 0.001039044 | 0.713212825 |
| TSTD2 | -1.193045667 | 5.987867355 | -4.419060838 | 0.000154999 | 0.001042134 | 0.709827373 |
| RCC2 | 1.315255063 | 10.44151138 | 4.418848917 | 0.000155086 | 0.001042172 | 0.70928546 |
| RPL36AL | -1.808446995 | 12.89579804 | -4.417486812 | 0.000155644 | 0.001045377 | 0.705802449 |
| BCAN | 2.197267319 | 7.71182965 | 4.416647958 | 0.000155989 | 0.001047146 | 0.703657496 |
| HOXB4 | 1.664035613 | 8.691495354 | 4.415878006 | 0.000156306 | 0.001048727 | 0.701688768 |
| ZNF532 | 1.197146377 | 7.639416405 | 4.413837452 | 0.00015715 | 0.001053837 | 0.696471385 |
| SLC6A6 | 1.005664226 | 8.484682095 | 4.413334767 | 0.000157358 | 0.001054685 | 0.695186142 |
| LY96 | 1.7492288 | 10.66293239 | 4.412676752 | 0.000157632 | 0.001055967 | 0.693503785 |
| NACA2 | -1.5180382 | 9.708909101 | -4.412054469 | 0.00015789 | 0.001057151 | 0.691912812 |
| ZNF439 | -2.257124937 | 4.699858516 | -4.411584518 | 0.000158086 | 0.001057911 | 0.690711322 |
| ODZ3 | 1.772796263 | 6.378365253 | 4.411110661 | 0.000158284 | 0.001058319 | 0.689499861 |
| LIMD1 | 1.463295983 | 7.876905496 | 4.411044226 | 0.000158312 | 0.001058319 | 0.689330013 |
| GNA14 | -1.989135586 | 5.324348622 | -4.410823513 | 0.000158404 | 0.001058385 | 0.688765747 |
| COX6B2 | 2.035579175 | 7.951225751 | 4.407724356 | 0.000159704 | 0.001066435 | 0.680842913 |
| MTERF | -1.36128126 | 5.187506869 | -4.407556598 | 0.000159775 | 0.001066435 | 0.680414069 |
| VKORC1L1 | -1.631894945 | 6.774532182 | -4.404013212 | 0.000161275 | 0.001075889 | 0.671356481 |
| ZNF77 | -1.695834987 | 5.504259469 | -4.403007567 | 0.000161703 | 0.001077179 | 0.668786021 |
| PTCRA | 2.517677508 | 8.382824507 | 4.4029975 | 0.000161707 | 0.001077179 | 0.668760289 |
| CALM2 | -1.464348993 | 12.89430025 | -4.402969192 | 0.000161719 | 0.001077179 | 0.668687934 |
| MLLT1 | 2.465777817 | 8.969575513 | 4.402454996 | 0.000161939 | 0.001078083 | 0.667373664 |
| ATP13A1 | -1.265489692 | 5.828953861 | -4.398234461 | 0.000163751 | 0.001089584 | 0.656586822 |
| CXorf26 | -1.843007257 | 5.053960333 | -4.397447403 | 0.000164091 | 0.001091283 | 0.654575404 |
| NOSIP | -1.141618015 | 8.43942519 | -4.39654587 | 0.000164482 | 0.001092663 | 0.652271487 |
| ST7 | -1.198313087 | 6.549067085 | -4.396463302 | 0.000164518 | 0.001092663 | 0.652060484 |
| TSPAN3 | -2.348162494 | 11.79983655 | -4.396248381 | 0.000164611 | 0.001092663 | 0.651511252 |
| USP17 | 2.353126473 | 8.294868387 | 4.396169208 | 0.000164645 | 0.001092663 | 0.651308926 |
| ATG9A | -1.382285482 | 7.003254777 | -4.395989208 | 0.000164723 | 0.001092663 | 0.650848938 |
| FOXF2 | -1.440794341 | 6.684944944 | -4.395134353 | 0.000165095 | 0.001094564 | 0.648664401 |
| H1FNT | 2.252768904 | 8.072449891 | 4.392768879 | 0.000166128 | 0.001100845 | 0.64261984 |
| IGDCC4 | 1.849336455 | 6.981250316 | 4.390726291 | 0.000167025 | 0.00110622 | 0.637400692 |
| RGS11 | 1.674329406 | 8.307797032 | 4.389655476 | 0.000167497 | 0.001108776 | 0.634664708 |
| SKIV2L | -1.645443831 | 4.18586059 | -4.389012754 | 0.000167782 | 0.001110085 | 0.633022562 |
| LOC100507165 | 2.15672942 | 7.718289174 | 4.388592136 | 0.000167968 | 0.001110746 | 0.631947906 |
| FIBIN | -2.147997684 | 5.407590875 | -4.387407154 | 0.000168493 | 0.001113649 | 0.628920416 |
| C1orf50 | -1.924603703 | 6.276797588 | -4.386422124 | 0.000168931 | 0.001115971 | 0.626403859 |
| HARS | -1.566842269 | 7.988993604 | -4.386053798 | 0.000169096 | 0.001116454 | 0.625462878 |
| TAOK2 | 2.508823542 | 11.27703838 | 4.385868547 | 0.000169178 | 0.001116454 | 0.624989611 |
| EIF2D | -1.368410852 | 6.878784383 | -4.385046209 | 0.000169545 | 0.001118304 | 0.62288879 |
| DPH3 | -1.222072157 | 7.72968389 | -4.383686682 | 0.000170154 | 0.001121744 | 0.619415729 |
| RAP1GAP | 4.088701988 | 8.324705519 | 4.381385799 | 0.000171189 | 0.00112799 | 0.613538189 |
| ATP5F1 | -2.151333068 | 8.860406921 | -4.381169115 | 0.000171287 | 0.001128057 | 0.612984696 |
| CHCHD4 | -1.750494636 | 6.775462061 | -4.378603252 | 0.00017245 | 0.001135132 | 0.606430791 |
| KRTAP13-2 | 2.113016943 | 7.568621271 | 4.378139692 | 0.000172661 | 0.001135938 | 0.605246787 |
| LRRC15 | 2.778491054 | 7.3417519 | 4.377770465 | 0.000172829 | 0.001136463 | 0.604303736 |
| PCNA | -1.828708122 | 7.677626159 | -4.37645218 | 0.00017343 | 0.001139837 | 0.600936759 |
| LSM4 | -1.286683392 | 7.408030762 | -4.376094304 | 0.000173594 | 0.00114033 | 0.600022748 |
| QSOX2 | 2.408085846 | 7.805913694 | 4.374496337 | 0.000174327 | 0.001144559 | 0.59594167 |
| ACTL6B | 2.572512794 | 8.154552144 | 4.373677631 | 0.000174703 | 0.001145919 | 0.593850839 |
| ZNF709 | -1.46045285 | 4.643546487 | -4.373658422 | 0.000174712 | 0.001145919 | 0.593801785 |
| LIME1 | 2.488615127 | 7.888239294 | 4.372467364 | 0.000175262 | 0.001148937 | 0.590760125 |
| GAK | -1.730083931 | 6.474295703 | -4.371798891 | 0.000175571 | 0.001150377 | 0.589053063 |
| METAP1 | -1.243023864 | 7.304092816 | -4.370132056 | 0.000176344 | 0.001154729 | 0.584796661 |
| ZNF738 | -1.452509825 | 9.477711246 | -4.369979901 | 0.000176414 | 0.001154729 | 0.584408131 |
| GDI2 | -1.346310999 | 7.926732293 | -4.369149064 | 0.000176801 | 0.001156672 | 0.58228661 |
| TXN | -1.497716216 | 11.63419806 | -4.366731242 | 0.000177931 | 0.001163474 | 0.576113072 |
| SLC5A6 | -1.531767797 | 5.060770893 | -4.365191361 | 0.000178655 | 0.001167612 | 0.57218146 |
| KLK11 | -2.751144001 | 7.067906185 | -4.363566047 | 0.000179422 | 0.001171678 | 0.568031922 |
| RRAGA | -1.949936127 | 10.79054165 | -4.36348687 | 0.000179459 | 0.001171678 | 0.567829784 |
| GAS7 | 1.893324643 | 7.986170352 | 4.362759234 | 0.000179804 | 0.001173332 | 0.565972152 |
| PKIG | -1.798139767 | 6.04930384 | -4.361923206 | 0.0001802 | 0.001175323 | 0.563837848 |
| DNLZ | -1.969888902 | 5.152319417 | -4.359681917 | 0.000181268 | 0.001181687 | 0.558116318 |
| C22orf25 | 2.326304682 | 8.74955677 | 4.358372171 | 0.000181895 | 0.001184605 | 0.554773003 |
| OSGEPL1 | -1.999171263 | 5.726858307 | -4.358361445 | 0.0001819 | 0.001184605 | 0.554745624 |
| PYCRL | 2.163235035 | 9.84829911 | 4.35583462 | 0.000183115 | 0.001191915 | 0.548295924 |
| RNF4 | -1.495939601 | 8.68091734 | -4.355377363 | 0.000183336 | 0.001192361 | 0.547128834 |
| C22orf23 | -2.754599201 | 4.444162512 | -4.355308761 | 0.000183369 | 0.001192361 | 0.546953738 |
| TARP | -2.127264437 | 4.53921757 | -4.353018796 | 0.000184479 | 0.001198972 | 0.54110916 |
| KCNG1 | 1.884600415 | 7.826213224 | 4.352821504 | 0.000184575 | 0.001198989 | 0.54060564 |
| TMEM50A | -1.030009584 | 9.0832136 | -4.352469714 | 0.000184746 | 0.001199495 | 0.539707824 |
| COPZ1 | 1.191075685 | 10.40717581 | 4.351963647 | 0.000184992 | 0.00120049 | 0.53841629 |
| UBE2S | 1.101846062 | 8.099935293 | 4.351002636 | 0.000185461 | 0.001202927 | 0.535963752 |
| GGT3P | 2.473271176 | 11.11985815 | 4.350239189 | 0.000185835 | 0.001204741 | 0.534015457 |
| POM121L12 | 2.721220363 | 10.48457285 | 4.350041314 | 0.000185932 | 0.001204762 | 0.533510493 |
| SPTLC2 | -1.244208145 | 5.250366126 | -4.349325078 | 0.000186283 | 0.00120643 | 0.531682733 |
| EGFEM1P | -2.171549808 | 4.03529597 | -4.348205499 | 0.000186833 | 0.001209385 | 0.528825769 |
| SLC12A9 | 1.124938469 | 10.24994532 | 4.347742099 | 0.000187061 | 0.001210223 | 0.527643283 |
| IGFBP7 | -2.766772402 | 13.31638402 | -4.347560972 | 0.000187151 | 0.001210223 | 0.527181096 |
| IGF1 | 1.890799099 | 8.48605642 | 4.347309956 | 0.000187275 | 0.001210415 | 0.526540575 |
| DCAF10 | 3.112115354 | 11.8040548 | 4.347072288 | 0.000187392 | 0.001210564 | 0.52593412 |
| ANK3 | -2.13930095 | 8.260047167 | -4.346512483 | 0.000187668 | 0.001211743 | 0.524505688 |
| WRNIP1 | -1.357016216 | 5.952081372 | -4.344928781 | 0.000188453 | 0.001216199 | 0.520464754 |
| ZNF773 | -1.614097616 | 4.822537769 | -4.344420304 | 0.000188706 | 0.001217135 | 0.519167382 |
| OBSL1 | -1.600474732 | 5.528564472 | -4.344255953 | 0.000188787 | 0.001217135 | 0.518748047 |
| LOC100129960 | 1.306897559 | 8.500044245 | 4.341639928 | 0.000190093 | 0.001224938 | 0.512073659 |
| FOXL1 | 2.277929239 | 7.839301579 | 4.340242131 | 0.000190794 | 0.001228643 | 0.508507624 |
| MKI67IP | -1.751010089 | 6.632874571 | -4.340113362 | 0.000190859 | 0.001228643 | 0.508179118 |
| TRAPPC2L | -1.78805627 | 7.79165106 | -4.339497845 | 0.000191169 | 0.001230021 | 0.506608879 |
| CSF1 | 2.826159816 | 7.789265951 | 4.338859968 | 0.00019149 | 0.001230389 | 0.504981631 |
| SAMD5 | -2.024835899 | 5.677475935 | -4.338782476 | 0.000191529 | 0.001230389 | 0.504783948 |
| KRTAP1-3 | 2.346434286 | 8.547969735 | 4.338719604 | 0.000191561 | 0.001230389 | 0.504623563 |
| MPHOSPH9 | 1.08554686 | 6.72681673 | 4.33862553 | 0.000191609 | 0.001230389 | 0.504383579 |
| ANGEL2 | -1.279694723 | 7.472300367 | -4.335964199 | 0.000192957 | 0.001238427 | 0.49759484 |
| DNM2 | -1.327461163 | 6.179823203 | -4.335158498 | 0.000193366 | 0.001240439 | 0.495539708 |
| LSM2 | -1.693860909 | 5.891423913 | -4.333929376 | 0.000193994 | 0.001243841 | 0.492404645 |
| CNNM3 | -2.027904279 | 6.085020413 | -4.332000271 | 0.000194982 | 0.001249554 | 0.487484421 |
| RNASEH2B | -1.462313948 | 6.215623448 | -4.331574328 | 0.000195201 | 0.001250127 | 0.486398087 |
| TOMM6 | -1.221511883 | 9.749947173 | -4.331448086 | 0.000195266 | 0.001250127 | 0.486076119 |
| CYP4B1 | -2.737286701 | 9.056020505 | -4.329341352 | 0.000196352 | 0.001256458 | 0.480703302 |
| LMAN2 | -1.136947023 | 7.687822457 | -4.328620085 | 0.000196725 | 0.001258221 | 0.478863935 |
| CENPBD1 | -1.735710973 | 6.470039026 | -4.328382546 | 0.000196849 | 0.001258383 | 0.478258176 |
| CLDN18 | -3.105937692 | 7.479767274 | -4.327821046 | 0.00019714 | 0.001259619 | 0.476826286 |
| LOC643406 | 1.932642757 | 6.880894823 | 4.327220082 | 0.000197452 | 0.001260988 | 0.475293789 |
| WDR34 | -1.524234616 | 7.542270134 | -4.326175139 | 0.000197996 | 0.001263835 | 0.472629191 |
| HFE | 2.315582049 | 6.99775286 | 4.325950732 | 0.000198113 | 0.001263854 | 0.472056965 |
| MBD2 | -1.460523737 | 9.851284441 | -4.325792919 | 0.000198196 | 0.001263854 | 0.471654553 |
| SERF1A | -1.844279136 | 6.507464392 | -4.325572213 | 0.000198311 | 0.001263962 | 0.471091773 |
| NDUFC2 | -1.580414701 | 11.71412258 | -4.325039257 | 0.000198589 | 0.00126511 | 0.4697328 |
| MLXIPL | 2.133385356 | 9.514845097 | 4.324547078 | 0.000198847 | 0.001266124 | 0.468477827 |
| ERBB4 | -1.925024466 | 5.808973869 | -4.323788308 | 0.000199245 | 0.001267607 | 0.466543129 |
| C6orf145 | -1.770764994 | 5.126947592 | -4.323726912 | 0.000199277 | 0.001267607 | 0.466386584 |
| LOC654433 | -1.309469249 | 7.068759157 | -4.322945324 | 0.000199688 | 0.001269591 | 0.464393762 |
| ZNF347 | -1.420866976 | 5.045152995 | -4.322441446 | 0.000199953 | 0.001270342 | 0.463109045 |
| ABCB6 | 1.793118029 | 9.305992395 | 4.322345429 | 0.000200003 | 0.001270342 | 0.462864238 |
| UBXN11 | 2.06664707 | 7.355759321 | 4.32150934 | 0.000200444 | 0.001272514 | 0.460732554 |
| ADAMTSL1 | 2.288870182 | 8.130918086 | 4.320020935 | 0.000201231 | 0.001276251 | 0.456937878 |
| CYP4X1 | -1.515793208 | 7.500520769 | -4.319146593 | 0.000201695 | 0.001278563 | 0.454708839 |
| NOL9 | -1.383457195 | 7.878742982 | -4.318247412 | 0.000202173 | 0.001280962 | 0.452416542 |
| TCTN1 | -1.389300297 | 7.638308295 | -4.317924868 | 0.000202345 | 0.00128142 | 0.451594295 |
| MUC8 | 2.105040753 | 9.001647256 | 4.317737044 | 0.000202445 | 0.001281423 | 0.451115486 |
| PRPF4 | 1.628025656 | 8.83232546 | 4.317260168 | 0.0002027 | 0.001282402 | 0.449899827 |
| ZNF792 | -1.739009002 | 4.865178678 | -4.31699546 | 0.000202841 | 0.001282665 | 0.449225038 |
| ZSCAN1 | 2.661312301 | 10.09863132 | 4.316565798 | 0.000203071 | 0.001283486 | 0.448129765 |
| CLDN11 | 2.436182621 | 8.781104565 | 4.316183771 | 0.000203275 | 0.001283717 | 0.447155934 |
| WIPF1 | 1.340787439 | 9.538734817 | 4.316124335 | 0.000203307 | 0.001283717 | 0.447004427 |
| NSMCE2 | -1.239354616 | 6.590467724 | -4.312544347 | 0.000205232 | 0.001295239 | 0.437879255 |
| C1orf186 | 2.346723091 | 9.152819696 | 4.311979479 | 0.000205538 | 0.001296175 | 0.436439543 |
| TMEM151A | 2.631588799 | 8.217371339 | 4.311897544 | 0.000205582 | 0.001296175 | 0.43623071 |
| ZMYND15 | 1.677159048 | 9.835264978 | 4.309380716 | 0.000206949 | 0.001303245 | 0.429816276 |
| PLXNA3 | 1.462612333 | 6.182698592 | 4.309316651 | 0.000206984 | 0.001303245 | 0.429653005 |
| TCEAL4 | -1.816857885 | 10.24381142 | -4.309273286 | 0.000207007 | 0.001303245 | 0.42954249 |
| TAF11 | -1.557205719 | 5.792406286 | -4.308518246 | 0.000207419 | 0.001305199 | 0.427618294 |
| TNRC6C | 1.076685544 | 9.496631743 | 4.306483894 | 0.000208533 | 0.001311567 | 0.422434063 |
| TCL6 | 2.2922144 | 7.917515195 | 4.30552583 | 0.00020906 | 0.001314236 | 0.41999271 |
| C9orf46 | -1.773274577 | 6.03980461 | -4.305331838 | 0.000209167 | 0.001314265 | 0.419498386 |
| CLDND1 | -1.439303638 | 8.828327292 | -4.303593305 | 0.000210126 | 0.001319649 | 0.415068466 |
| FOXD1 | 2.66084534 | 8.830183115 | 4.302451363 | 0.000210759 | 0.001322976 | 0.412158854 |
| BACE2 | 1.31919334 | 10.84397921 | 4.299897107 | 0.000212181 | 0.001331252 | 0.405651147 |
| PLEKHO1 | 1.22109219 | 11.65885201 | 4.299541647 | 0.000212379 | 0.00133159 | 0.404745557 |
| COX15 | -1.607125622 | 7.644244989 | -4.299430078 | 0.000212442 | 0.00133159 | 0.404461317 |
| C4orf38 | -1.74059776 | 5.284260936 | -4.297719351 | 0.0002134 | 0.001336948 | 0.400103141 |
| AP3M2 | -1.817547932 | 6.831051945 | -4.297029701 | 0.000213788 | 0.001338725 | 0.398346291 |
| UBE2Q1 | -2.224599221 | 4.430805193 | -4.296766098 | 0.000213936 | 0.001339003 | 0.397674785 |
| ZNF257 | 2.280265132 | 8.757122432 | 4.296423634 | 0.000214129 | 0.001339558 | 0.396802398 |
| B3GNT3 | 2.471051057 | 7.447061055 | 4.296019411 | 0.000214357 | 0.0013401 | 0.395772701 |
| T | 2.097471564 | 7.236623822 | 4.295900741 | 0.000214424 | 0.0013401 | 0.395470411 |
| HBM | 3.511347721 | 8.531047755 | 4.295379206 | 0.000214719 | 0.001341035 | 0.394141905 |
| MPND | -2.055398887 | 4.986090229 | -4.295266746 | 0.000214782 | 0.001341035 | 0.39385544 |
| GGPS1 | -1.239152751 | 8.11706111 | -4.294998808 | 0.000214934 | 0.001341288 | 0.393172934 |
| RNASEH2C | -1.008465173 | 8.914151254 | -4.294826133 | 0.000215032 | 0.001341288 | 0.392733092 |
| MED11 | -1.893286363 | 5.974359212 | -4.293838266 | 0.000215591 | 0.001343784 | 0.390216816 |
| FCRL5 | 2.620600556 | 7.41406327 | 4.293751403 | 0.000215641 | 0.001343784 | 0.389995563 |
| PPHLN1 | -1.228574446 | 6.421041849 | -4.293373161 | 0.000215856 | 0.001344472 | 0.389032138 |
| ARHGEF37 | -1.990793482 | 6.092771462 | -4.289175079 | 0.000218254 | 0.00135875 | 0.378340018 |
| ORC3 | -1.321756109 | 7.557076329 | -4.288037756 | 0.000218908 | 0.001362164 | 0.375443636 |
| ZNF503-AS1 | -1.212089078 | 4.992921353 | -4.287641725 | 0.000219136 | 0.001362925 | 0.374435106 |
| LOC388242 | 1.276071401 | 7.329577855 | 4.287335803 | 0.000219313 | 0.001363363 | 0.373656057 |
| TSEN2 | -1.370546867 | 6.536887408 | -4.286244139 | 0.000219944 | 0.001366625 | 0.370876132 |
| CREB3L3 | 1.819790544 | 6.263016966 | 4.285240137 | 0.000220525 | 0.001369579 | 0.368319535 |
| MESDC2 | -1.854728915 | 7.8713955 | -4.284171242 | 0.000221147 | 0.001372128 | 0.365597795 |
| DCTN4 | -2.368012883 | 7.449341941 | -4.284166669 | 0.000221149 | 0.001372128 | 0.365586151 |
| LOC100507316 | 2.097724495 | 8.121197001 | 4.283448446 | 0.000221568 | 0.001373695 | 0.363757391 |
| ADAM12 | 1.829917377 | 6.332022471 | 4.283366497 | 0.000221615 | 0.001373695 | 0.363548733 |
| GDNF | 2.519341407 | 8.401729223 | 4.282103967 | 0.000222353 | 0.001377602 | 0.360334165 |
| EDC3 | -1.320025386 | 4.46217076 | -4.28179973 | 0.000222531 | 0.001378042 | 0.359559559 |
| CLDN12 | -1.615442679 | 4.679033855 | -4.281033475 | 0.00022298 | 0.00137992 | 0.357608664 |
| KIF9 | -1.600284419 | 7.571225082 | -4.280916398 | 0.000223049 | 0.00137992 | 0.357310589 |
| FGD1 | 2.027490418 | 7.344900217 | 4.279734034 | 0.000223744 | 0.001383113 | 0.354300396 |
| SLC39A6 | -1.513802277 | 6.527852457 | -4.279613293 | 0.000223815 | 0.001383113 | 0.353993009 |
| ACLY | 1.09929304 | 9.060337419 | 4.279490011 | 0.000223887 | 0.001383113 | 0.353679151 |
| HIST1H2AM | 1.444764693 | 9.810726654 | 4.278896081 | 0.000224238 | 0.001384117 | 0.352167119 |
| MTSS1L | 2.539075338 | 9.373248699 | 4.278849274 | 0.000224265 | 0.001384117 | 0.35204796 |
| NEFM | 2.22545708 | 7.582981774 | 4.277950768 | 0.000224796 | 0.001386727 | 0.349760602 |
| C19orf79 | -1.783363701 | 10.17083844 | -4.277027556 | 0.000225343 | 0.001389433 | 0.347410425 |
| USP12 | -1.770478103 | 7.665822651 | -4.274798901 | 0.000226668 | 0.001395526 | 0.341737373 |
| PRAF2 | 1.049139367 | 8.495350909 | 4.274680961 | 0.000226738 | 0.001395526 | 0.341437169 |
| CCDC6 | -1.324847868 | 10.02186726 | -4.274636346 | 0.000226765 | 0.001395526 | 0.341323606 |
| AUP1 | -1.48227201 | 7.05248222 | -4.274310536 | 0.000226959 | 0.001396055 | 0.340494299 |
| SMUG1 | -1.696881776 | 4.956045319 | -4.273471152 | 0.000227461 | 0.001398472 | 0.3383578 |
| KRT10 | -1.235714759 | 10.97222487 | -4.273162505 | 0.000227646 | 0.00139894 | 0.33757221 |
| C21orf96 | 2.689093311 | 8.947632456 | 4.272603769 | 0.000227981 | 0.001400328 | 0.336150102 |
| GLIS2 | 1.984370716 | 7.304989931 | 4.271604246 | 0.000228581 | 0.001402727 | 0.333606166 |
| PDE1B | 1.58022547 | 7.280485862 | 4.271590469 | 0.000228589 | 0.001402727 | 0.3335711 |
| C16orf45 | -2.003246008 | 7.529992912 | -4.271141457 | 0.000228859 | 0.001403715 | 0.332428328 |
| NDUFA10 | -1.14861485 | 6.249903812 | -4.270631853 | 0.000229166 | 0.001404928 | 0.331131365 |
| TCP10L2 | 3.298684157 | 8.896556893 | 4.269849737 | 0.000229638 | 0.001407151 | 0.329140897 |
| LGALS3 | -1.941467345 | 12.64405413 | -4.268586425 | 0.000230403 | 0.001410356 | 0.325925916 |
| ZNF341 | 1.997379545 | 7.577342387 | 4.268499583 | 0.000230455 | 0.001410356 | 0.325704918 |
| C6orf64 | -1.142100429 | 6.548695919 | -4.268301803 | 0.000230575 | 0.001410356 | 0.325201607 |
| PSMB5 | -1.179937624 | 7.906614314 | -4.268256328 | 0.000230603 | 0.001410356 | 0.325085882 |
| CHMP4B | -1.417137059 | 9.768409349 | -4.268080348 | 0.00023071 | 0.001410356 | 0.32463805 |
| C9orf163 | 2.395828566 | 10.46019833 | 4.266700532 | 0.000231549 | 0.001414812 | 0.321126817 |
| EFNB3 | 1.989612239 | 8.575850337 | 4.26644477 | 0.000231705 | 0.001415092 | 0.320475997 |
| C11orf71 | 1.902589079 | 9.691674021 | 4.2659715 | 0.000231993 | 0.001416182 | 0.319271711 |
| ZDHHC16 | -1.660053748 | 5.988288749 | -4.265394788 | 0.000232345 | 0.001417659 | 0.317804239 |
| RBM25 | -1.082845555 | 8.095598484 | -4.261711578 | 0.000234607 | 0.001430781 | 0.308432861 |
| KIAA1274 | -1.083514526 | 7.378414364 | -4.261437392 | 0.000234776 | 0.001431135 | 0.307735287 |
| CFD | -2.060099705 | 12.92969337 | -4.261087432 | 0.000234993 | 0.001431774 | 0.306844942 |
| FAM105A | -1.726503763 | 6.414416581 | -4.260782612 | 0.000235181 | 0.001432244 | 0.306069448 |
| MID1 | -1.868272992 | 4.584959466 | -4.259740017 | 0.000235827 | 0.001435497 | 0.303417044 |
| FOXB1 | 2.209942129 | 8.327555222 | 4.259410877 | 0.000236031 | 0.001436042 | 0.302579721 |
| MEFV | 2.318066846 | 7.826287317 | 4.259236087 | 0.00023614 | 0.001436042 | 0.302135064 |
| TAB3 | -1.274775396 | 5.738993341 | -4.257945536 | 0.000236942 | 0.001440243 | 0.298852056 |
| CNOT7 | -1.140809448 | 8.456779372 | -4.257371658 | 0.0002373 | 0.001441153 | 0.29739223 |
| CYP2A13 | 2.179543599 | 7.169948183 | 4.257346172 | 0.000237316 | 0.001441153 | 0.2973274 |
| IRX4 | 3.138802925 | 8.631525025 | 4.256144175 | 0.000238068 | 0.001445033 | 0.294269874 |
| THTPA | -1.20154579 | 6.199212524 | -4.255690465 | 0.000238352 | 0.001446076 | 0.293115806 |
| DHX34 | 2.336298217 | 7.607095241 | 4.254911255 | 0.000238841 | 0.001448359 | 0.291133832 |
| TFF2 | 2.199738131 | 8.70519242 | 4.253130953 | 0.000239962 | 0.00145447 | 0.286605734 |
| SPINK7 | 2.058077496 | 8.23704034 | 4.252779952 | 0.000240183 | 0.001455127 | 0.285713018 |
| UFM1 | -1.490674944 | 6.567538066 | -4.252053684 | 0.000240642 | 0.001457223 | 0.283865908 |
| LIMD2 | 1.24029047 | 7.076879779 | 4.251838162 | 0.000240779 | 0.001457363 | 0.283317785 |
| STAT2 | 1.515507067 | 7.409054575 | 4.250939151 | 0.000241349 | 0.001460125 | 0.281031428 |
| KCNE4 | 2.299592684 | 8.861443509 | 4.250261232 | 0.000241779 | 0.001462042 | 0.279307401 |
| RELB | 2.042073796 | 12.47147511 | 4.249463106 | 0.000242287 | 0.001464425 | 0.277277733 |
| TK1 | 2.194199215 | 11.15869781 | 4.248952994 | 0.000242612 | 0.001465702 | 0.275980527 |
| LILRB4 | 2.282013643 | 7.228221365 | 4.248566625 | 0.000242859 | 0.001466503 | 0.274998015 |
| LOC100130331 | 1.174088986 | 9.830477317 | 4.247641994 | 0.00024345 | 0.001469383 | 0.272646794 |
| ZNF732 | -1.876551966 | 5.301247486 | -4.247170171 | 0.000243752 | 0.001470517 | 0.271447041 |
| MAB21L3 | 2.202632887 | 7.291713883 | 4.246052714 | 0.000244469 | 0.001474152 | 0.268605649 |
| HIST1H2BN | 1.626301507 | 10.65622046 | 4.245761892 | 0.000244656 | 0.001474588 | 0.267866188 |
| HMX1 | 2.233445319 | 6.52481944 | 4.243654759 | 0.000246016 | 0.001482086 | 0.26250871 |
| AOX1 | -1.906573734 | 7.046713278 | -4.243020248 | 0.000246426 | 0.001483512 | 0.260895523 |
| LOC100130811 | 3.151248164 | 6.315346898 | 4.242932599 | 0.000246483 | 0.001483512 | 0.260672685 |
| NPR1 | -1.512706721 | 6.204927328 | -4.242479769 | 0.000246777 | 0.001484585 | 0.259521434 |
| CFDP1 | -1.115769883 | 7.53687267 | -4.240237173 | 0.000248236 | 0.001492665 | 0.25382027 |
| NXPH4 | 2.083707446 | 7.226425609 | 4.238069304 | 0.000249655 | 0.001500494 | 0.248309546 |
| MXRA8 | 2.164153789 | 12.077685 | 4.237617314 | 0.000249951 | 0.001501577 | 0.247160644 |
| RPS18 | -1.798001743 | 12.25184743 | -4.235202538 | 0.000251543 | 0.001510433 | 0.241022935 |
| NUMB | -1.360992235 | 8.246715484 | -4.234806293 | 0.000251805 | 0.001511301 | 0.240015842 |
| METTL11A | -1.764757002 | 5.351734874 | -4.233587001 | 0.000252614 | 0.001515445 | 0.236916998 |
| C17orf62 | -1.382970479 | 4.289935652 | -4.231355543 | 0.000254099 | 0.001523649 | 0.231246105 |
| MRGPRE | 2.192332587 | 7.461648924 | 4.230975641 | 0.000254353 | 0.001524461 | 0.230280695 |
| HIST1H4B | 1.523967374 | 8.622514808 | 4.228600488 | 0.000255946 | 0.001533292 | 0.224245259 |
| WNK1 | -1.747448821 | 8.051645751 | -4.226721719 | 0.000257213 | 0.001540164 | 0.219471569 |
| PRIC285 | 1.647458226 | 7.592699766 | 4.22611018 | 0.000257627 | 0.001541924 | 0.21791781 |
| SLAMF9 | 1.64654003 | 6.411827877 | 4.225532248 | 0.000258018 | 0.001543549 | 0.216449473 |
| GLIPR1L2 | -1.477699285 | 4.972449099 | -4.224624469 | 0.000258634 | 0.001545897 | 0.214143169 |
| TRIM35 | 2.283743343 | 9.907763339 | 4.224600121 | 0.000258651 | 0.001545897 | 0.21408131 |
| ATPIF1 | -1.446714336 | 11.94124446 | -4.222735552 | 0.000259921 | 0.001552768 | 0.209344452 |
| CAP1 | -1.473532287 | 8.328683895 | -4.221600277 | 0.000260698 | 0.001556505 | 0.206460504 |
| C16orf48 | -1.564124635 | 7.084491707 | -4.221467545 | 0.000260789 | 0.001556505 | 0.206123332 |
| SRCRB4D | 1.789900113 | 7.959080226 | 4.221114272 | 0.000261031 | 0.001557228 | 0.205225942 |
| SYCP1 | 2.17793979 | 7.447721578 | 4.220735835 | 0.000261291 | 0.001558054 | 0.204264641 |
| CKAP4 | 1.555205581 | 8.511019527 | 4.219468624 | 0.000262162 | 0.001562526 | 0.201045802 |
| TCEA3 | -1.303895238 | 7.075906796 | -4.218551326 | 0.000262795 | 0.001565398 | 0.198715875 |
| ZNF193 | -1.726366966 | 5.614088358 | -4.218417171 | 0.000262888 | 0.001565398 | 0.198375131 |
| PDHB | -1.24482737 | 8.143230672 | -4.217096052 | 0.000263802 | 0.001570115 | 0.195019674 |
| GDF10 | -3.037878269 | 7.136178673 | -4.214276628 | 0.000265763 | 0.001580789 | 0.187859326 |
| TRMT112 | -1.316331674 | 12.62070538 | -4.214155103 | 0.000265848 | 0.001580789 | 0.187550713 |
| TMEM72 | 2.129382289 | 8.187291377 | 4.213989104 | 0.000265964 | 0.001580789 | 0.187129162 |
| ATAD3C | 2.123029496 | 9.280176753 | 4.21307888 | 0.000266601 | 0.001583341 | 0.184817712 |
| KIR2DS4 | 2.439484926 | 6.958604813 | 4.213023601 | 0.000266639 | 0.001583341 | 0.18467734 |
| AIM2 | 2.431141479 | 8.61166442 | 4.211621231 | 0.000267624 | 0.001587719 | 0.181116295 |
| AGBL5 | 1.05279876 | 8.529025546 | 4.21130449 | 0.000267846 | 0.001588308 | 0.180312021 |
| ZNF222 | -1.426467217 | 5.569575942 | -4.209941967 | 0.000268807 | 0.001593268 | 0.176852396 |
| C5orf32 | -1.901629448 | 11.68617072 | -4.208841665 | 0.000269585 | 0.001597144 | 0.174058728 |
| ADRA1D | 2.665759469 | 9.839960382 | 4.208234353 | 0.000270015 | 0.001598958 | 0.172516814 |
| METRN | -1.721676682 | 5.974828712 | -4.207492723 | 0.000270542 | 0.001601338 | 0.17063393 |
| LMX1A | 2.113525608 | 7.373817853 | 4.20622037 | 0.000271447 | 0.00160596 | 0.167403755 |
| IL2RA | 1.906437003 | 6.396728567 | 4.205497787 | 0.000271963 | 0.001608271 | 0.16556938 |
| PVT1 | 1.761731085 | 6.561755727 | 4.20486377 | 0.000272416 | 0.001609407 | 0.163959886 |
| SPRY3 | 2.007082849 | 8.109145377 | 4.204764424 | 0.000272488 | 0.001609407 | 0.163707691 |
| PPP1R9B | 1.534158349 | 10.37051169 | 4.204669048 | 0.000272556 | 0.001609407 | 0.163465577 |
| DPF3 | 1.763635482 | 6.475931391 | 4.204529591 | 0.000272656 | 0.001609407 | 0.163111565 |
| TSPAN6 | -1.814584405 | 6.3489595 | -4.203758165 | 0.000273209 | 0.001611931 | 0.161153332 |
| PELO | -1.599652728 | 7.335301748 | -4.203398391 | 0.000273467 | 0.001612715 | 0.160240082 |
| KIFC2 | 1.928782762 | 8.032248816 | 4.20145843 | 0.000274864 | 0.001620211 | 0.155315915 |
| ZNF274 | -1.235968397 | 5.770495059 | -4.200741655 | 0.000275382 | 0.00162252 | 0.153496639 |
| KRTAP4-8 | 2.861196211 | 8.158874315 | 4.200308448 | 0.000275695 | 0.001623623 | 0.152397124 |
| TAL1 | 2.262193393 | 7.279699216 | 4.19990056 | 0.000275991 | 0.001624083 | 0.15136189 |
| MAP3K3 | 1.061227047 | 11.25482681 | 4.199852038 | 0.000276026 | 0.001624083 | 0.15123874 |
| C12orf49 | -1.527567408 | 6.248542416 | -4.198555948 | 0.000276967 | 0.001628876 | 0.147949337 |
| DDT | -1.419562947 | 9.975734015 | -4.197691757 | 0.000277596 | 0.001631819 | 0.145756165 |
| SECISBP2 | -1.425878823 | 9.662192925 | -4.197517734 | 0.000277723 | 0.001631819 | 0.145314536 |
| UBR4 | -1.870469499 | 7.839725893 | -4.197346498 | 0.000277848 | 0.001631819 | 0.144879979 |
| ZEB2 | 1.485955092 | 7.744053691 | 4.196459392 | 0.000278496 | 0.001634879 | 0.142628768 |
| TFDP1 | 1.21415578 | 7.664948171 | 4.195068233 | 0.000279516 | 0.001640113 | 0.139098587 |
| LINC00304 | 3.207015144 | 12.05272979 | 4.194648711 | 0.000279824 | 0.001641173 | 0.138034057 |
| MRPL44 | 1.104517739 | 7.63884632 | 4.188226434 | 0.000284582 | 0.001668321 | 0.121739942 |
| RAD54L2 | -1.160373884 | 7.643603537 | -4.187929684 | 0.000284804 | 0.001668321 | 0.120987156 |
| FEZ1 | -1.621435499 | 5.976443142 | -4.187879439 | 0.000284842 | 0.001668321 | 0.120859697 |
| SH2D2A | 2.217960982 | 10.21572106 | 4.187680544 | 0.00028499 | 0.001668433 | 0.120355154 |
| RASSF4 | 2.124276901 | 11.07718357 | 4.186314625 | 0.000286014 | 0.001673665 | 0.116890292 |
| ELFN1 | 1.99306452 | 8.42723434 | 4.184745413 | 0.000287195 | 0.00167981 | 0.112909993 |
| PRELP | -1.724283044 | 8.253780491 | -4.18396651 | 0.000287783 | 0.001682483 | 0.110934407 |
| SYTL4 | -1.817265057 | 5.859830948 | -4.183067331 | 0.000288463 | 0.001685693 | 0.108653838 |
| POLR2A | 1.629120294 | 7.586477232 | 4.181525142 | 0.000289633 | 0.001691763 | 0.104742619 |
| FXYD6 | -1.7616162 | 7.172086043 | -4.181305398 | 0.0002898 | 0.001691971 | 0.104185337 |
| UAP1 | -1.595156799 | 5.668180103 | -4.1783337 | 0.00029207 | 0.001703733 | 0.096649466 |
| HIST1H3C | 1.269322418 | 9.255862789 | 4.178320444 | 0.00029208 | 0.001703733 | 0.096615852 |
| MXD3 | 2.312744282 | 8.724905032 | 4.177779355 | 0.000292495 | 0.001705381 | 0.095243819 |
| ATP6V1E2 | -1.472707082 | 4.782679174 | -4.176716405 | 0.000293312 | 0.001709371 | 0.0925486 |
| RAB8A | 1.179345964 | 8.280352601 | 4.174753014 | 0.000294828 | 0.001717424 | 0.087570544 |
| RPS6KA1 | 1.193289378 | 8.68314115 | 4.17446034 | 0.000295054 | 0.001717965 | 0.086828523 |
| PTS | -1.318426274 | 7.932922132 | -4.171730399 | 0.000297176 | 0.001729535 | 0.079907712 |
| MXI1 | -2.480177758 | 7.924768605 | -4.17094584 | 0.000297788 | 0.001732316 | 0.077918888 |
| C1orf144 | 1.534276532 | 10.70912085 | 4.169832246 | 0.00029866 | 0.0017366 | 0.075096088 |
| PIGP | -2.448212677 | 7.411561033 | -4.169299009 | 0.000299078 | 0.001738246 | 0.073744458 |
| PGLYRP3 | 2.177137415 | 6.885961552 | 4.168995586 | 0.000299316 | 0.001738845 | 0.072975366 |
| PUF60 | -1.959626014 | 8.677871868 | -4.167760675 | 0.000300288 | 0.001743701 | 0.069845318 |
| LOC100129648 | 1.869676283 | 8.858215646 | 4.16732241 | 0.000300633 | 0.00174492 | 0.068734519 |
| ABCG1 | -1.553600454 | 6.783902405 | -4.166949434 | 0.000300927 | 0.001745841 | 0.06778921 |
| DUSP4 | 1.9456081 | 8.95497593 | 4.166570597 | 0.000301227 | 0.001746009 | 0.066829067 |
| NYX | 2.242793443 | 8.492661696 | 4.166569095 | 0.000301228 | 0.001746009 | 0.066825258 |
| UBE2K | -1.595989634 | 10.788852 | -4.164485051 | 0.00030288 | 0.001754792 | 0.061543626 |
| FUT6 | 1.431818357 | 13.18710955 | 4.16392368 | 0.000303326 | 0.001756587 | 0.060121013 |
| FBXL19 | 2.211884299 | 8.614036369 | 4.16371106 | 0.000303495 | 0.00175665 | 0.059582207 |
| LCP1 | -2.609779498 | 10.61938346 | -4.163567026 | 0.00030361 | 0.00175665 | 0.059217208 |
| COX7A1 | -1.439590923 | 10.30581081 | -4.162673019 | 0.000304323 | 0.001759984 | 0.056951747 |
| C19orf44 | 1.406076123 | 8.244739865 | 4.159332557 | 0.000307002 | 0.001774678 | 0.048487623 |
| MEF2C | -1.657562507 | 7.83048297 | -4.15892974 | 0.000307327 | 0.001775756 | 0.047467042 |
| SLC29A4 | 2.879567076 | 11.44145799 | 4.157885101 | 0.00030817 | 0.00177983 | 0.044820417 |
| CCT5 | -1.72479673 | 11.09700779 | -4.156818967 | 0.000309033 | 0.001784014 | 0.042119463 |
| CRHR1 | 1.865795491 | 7.881819048 | 4.156221948 | 0.000309517 | 0.001786008 | 0.040607023 |
| BAI1 | 1.958471573 | 7.091932454 | 4.155761529 | 0.000309891 | 0.00178697 | 0.039440661 |
| EVX1 | 2.170894855 | 7.833831246 | 4.155485087 | 0.000310116 | 0.00178697 | 0.038740375 |
| HTT | 2.519716806 | 9.668644072 | 4.155424629 | 0.000310165 | 0.00178697 | 0.038587221 |
| THAP4 | -1.251784086 | 8.361497708 | -4.155333135 | 0.00031024 | 0.00178697 | 0.038355449 |
| MRPS12 | 1.088921018 | 10.39572084 | 4.154765566 | 0.000310702 | 0.001788832 | 0.036917709 |
| IDI1 | -1.438511738 | 8.351027237 | -4.154106833 | 0.000311239 | 0.001791124 | 0.035249084 |
| MUC2 | 2.169934011 | 7.532518898 | 4.153000523 | 0.000312144 | 0.001795525 | 0.032446817 |
| FAM111A | 1.821220119 | 12.01183635 | 4.151968646 | 0.00031299 | 0.001799587 | 0.029833211 |
| UGP2 | -1.662587124 | 9.123570034 | -4.150929694 | 0.000313844 | 0.001803691 | 0.027201806 |
| SLC14A2 | 2.789826673 | 9.957657938 | 4.149706752 | 0.000314852 | 0.001808553 | 0.024104557 |
| C9orf24 | -2.819534912 | 9.240827191 | -4.149562821 | 0.000314971 | 0.001808553 | 0.023740045 |
| HBQ1 | 2.797601892 | 9.552597983 | 4.148072786 | 0.000316204 | 0.001814824 | 0.019966604 |
| RTN4RL1 | 1.995306175 | 7.891969539 | 4.147685231 | 0.000316526 | 0.00181579 | 0.018985181 |
| SPCS1 | -1.686085965 | 8.828694941 | -4.147529728 | 0.000316655 | 0.00181579 | 0.0185914 |
| H2AFB2 | 2.250018634 | 7.372240451 | 4.145767987 | 0.000318121 | 0.001823386 | 0.014130309 |
| SPEF1 | 1.972529178 | 8.896225655 | 4.145481818 | 0.00031836 | 0.001823942 | 0.013405703 |
| SNX21 | 1.100111146 | 8.980941987 | 4.145062119 | 0.000318711 | 0.001825138 | 0.012343006 |
| PITPNB | -1.792898596 | 8.209166692 | -4.144805299 | 0.000318926 | 0.001825555 | 0.011692733 |
| PLB1 | 1.209696727 | 6.579249453 | 4.144406557 | 0.000319259 | 0.001826651 | 0.01068313 |
| APIP | -2.225928393 | 5.266547593 | -4.142295083 | 0.000321032 | 0.001835977 | 0.005337236 |
| QTRTD1 | -1.637090206 | 7.527231746 | -4.141189224 | 0.000321964 | 0.001839868 | 0.002537594 |
| PRCC | 1.033539455 | 6.674599063 | 4.141148595 | 0.000321998 | 0.001839868 | 0.002434739 |
| MRPL39 | -1.110035173 | 7.82322236 | -4.14097067 | 0.000322149 | 0.00183991 | 0.001984309 |
| RPS23 | -1.764845676 | 12.28534974 | -4.140064932 | 0.000322915 | 0.001843466 | -0.000308572 |
| VLDLR | -1.366181425 | 5.696345777 | -4.13989327 | 0.00032306 | 0.001843477 | -0.000743124 |
| SHPK | 2.265741212 | 10.65899604 | 4.139305464 | 0.000323558 | 0.00184507 | -0.002231096 |
| MFSD5 | -1.597918295 | 7.937529022 | -4.139225753 | 0.000323626 | 0.00184507 | -0.002432874 |
| HDAC7 | 1.319532573 | 10.68986473 | 4.139022624 | 0.000323798 | 0.001845235 | -0.002947064 |
| PSME1 | -1.597520735 | 10.48329171 | -4.135946442 | 0.00032642 | 0.001859353 | -0.010733364 |
| CTNNAL1 | -1.960703317 | 10.09895897 | -4.135590566 | 0.000326725 | 0.001859578 | -0.011634074 |
| ZNF552 | 2.778041177 | 7.793079355 | 4.135562595 | 0.000326749 | 0.001859578 | -0.011704865 |
| ASAH1 | -1.390594918 | 7.754501521 | -4.135157145 | 0.000327097 | 0.001860733 | -0.012731025 |
| UBD | 2.51873169 | 10.55955767 | 4.134658718 | 0.000327524 | 0.001862342 | -0.013992474 |
| TBP | -1.240023019 | 6.572391038 | -4.134257503 | 0.000327869 | 0.001863478 | -0.015007871 |
| LSM7 | 1.190910497 | 11.35345037 | 4.133775225 | 0.000328284 | 0.001865011 | -0.0162284 |
| LOC100652765 | 1.486219239 | 8.290630514 | 4.13203966 | 0.000329781 | 0.001872689 | -0.020620471 |
| RPL34 | 1.142115675 | 14.06520419 | 4.130392407 | 0.000331208 | 0.001879963 | -0.024788732 |
| LRMP | 1.354642548 | 8.033443057 | 4.129207602 | 0.000332238 | 0.001884591 | -0.027786607 |
| CCT2 | -1.44453857 | 11.01938486 | -4.128986334 | 0.000332431 | 0.001884591 | -0.028346455 |
| FAM103A1 | 2.011904152 | 8.580439383 | 4.128949738 | 0.000332463 | 0.001884591 | -0.028439049 |
| RCBTB2 | -1.298720336 | 7.05108766 | -4.128496917 | 0.000332858 | 0.001885997 | -0.029584752 |
| CHMP5 | -2.160194739 | 8.933318161 | -4.127927447 | 0.000333355 | 0.001887983 | -0.031025556 |
| PSMC6 | -1.834011385 | 7.930854156 | -4.126915499 | 0.00033424 | 0.001892164 | -0.033585777 |
| DCXR | -1.730306477 | 8.410416321 | -4.126552123 | 0.000334559 | 0.001893134 | -0.034505085 |
| ALG10 | -1.946826949 | 5.306224038 | -4.125627742 | 0.00033537 | 0.001896892 | -0.036843616 |
| C9orf91 | -1.317437751 | 6.384580703 | -4.12512826 | 0.00033581 | 0.001898542 | -0.038107181 |
| THSD1 | -2.095185792 | 9.175815741 | -4.124537929 | 0.000336329 | 0.001900646 | -0.039600534 |
| CISH | 3.217009025 | 9.05730838 | 4.123127616 | 0.000337575 | 0.001906652 | -0.043168019 |
| GPR114 | 1.355705689 | 6.81092109 | 4.1229989 | 0.000337689 | 0.001906652 | -0.043493603 |
| ARPC5 | 1.535221904 | 13.22826813 | 4.122652893 | 0.000337995 | 0.001907544 | -0.04436881 |
| LDHB | -1.635534676 | 11.54258905 | -4.122192302 | 0.000338403 | 0.001909011 | -0.045533832 |
| SNX7 | -2.238231782 | 6.957757411 | -4.121505098 | 0.000339013 | 0.001911613 | -0.047272002 |
| PFN1 | 1.311472503 | 9.828609814 | 4.119863978 | 0.000340474 | 0.001919011 | -0.05142272 |
| MCCD1 | 1.969039323 | 6.962845482 | 4.119585706 | 0.000340723 | 0.001919184 | -0.052126495 |
| CDYL | -1.179965566 | 5.576582796 | -4.119495147 | 0.000340804 | 0.001919184 | -0.052355525 |
| PRO2949 | 2.777270202 | 6.922666287 | 4.117681996 | 0.000342427 | 0.00192627 | -0.056940895 |
| USF2 | 1.788625108 | 8.124568667 | 4.117587904 | 0.000342511 | 0.00192627 | -0.057178838 |
| IFITM5 | 2.877178484 | 12.7248942 | 4.116774902 | 0.000343242 | 0.001929534 | -0.059234744 |
| COQ9 | -1.218179215 | 7.036023677 | -4.116528329 | 0.000343463 | 0.001929938 | -0.059858259 |
| CXCL11 | 3.785707059 | 8.518311256 | 4.116122456 | 0.000343829 | 0.001931147 | -0.060884585 |
| CABIN1 | 1.169270667 | 9.540690059 | 4.114629553 | 0.000345176 | 0.001937187 | -0.064659493 |
| C15orf57 | -2.036810651 | 6.213001223 | -4.114597545 | 0.000345205 | 0.001937187 | -0.064740425 |
| MGST3 | -1.584541386 | 11.86626035 | -4.113892919 | 0.000345843 | 0.001939843 | -0.066522027 |
| MEN1 | 2.509515035 | 10.92762824 | 4.113726469 | 0.000345994 | 0.001939843 | -0.066942877 |
| FAF1 | -2.109928435 | 6.646999482 | -4.113575344 | 0.000346131 | 0.001939843 | -0.067324975 |
| RNF181 | -1.233782164 | 9.311393846 | -4.112955509 | 0.000346694 | 0.001942149 | -0.068892117 |
| SERPINF1 | 1.575601205 | 10.77287081 | 4.112520719 | 0.000347089 | 0.001943517 | -0.069991376 |
| TNFSF8 | 2.019432769 | 6.847039112 | 4.110418236 | 0.000349006 | 0.001953401 | -0.075306672 |
| SLC9A5 | 1.665755733 | 7.856992452 | 4.109360209 | 0.000349975 | 0.001956415 | -0.077981276 |
| C7orf25 | -1.3607739 | 4.894583764 | -4.109356665 | 0.000349978 | 0.001956415 | -0.077990235 |
| TXLNA | 1.148226187 | 7.695204487 | 4.109331613 | 0.000350001 | 0.001956415 | -0.078053561 |
| RBCK1 | -1.288265469 | 5.76425058 | -4.107594735 | 0.000351597 | 0.001963711 | -0.082443946 |
| INHBC | 2.125947897 | 7.604013637 | 4.107578979 | 0.000351612 | 0.001963711 | -0.082483771 |
| GPAT2 | 1.594849026 | 8.715983525 | 4.107248299 | 0.000351916 | 0.00196456 | -0.083319604 |
| DNAJC15 | -1.251441952 | 9.734081003 | -4.106112082 | 0.000352965 | 0.001969349 | -0.086191427 |
| KRT16P2 | 2.168182421 | 4.851225094 | 4.10598755 | 0.00035308 | 0.001969349 | -0.086506176 |
| OPLAH | 1.400415217 | 8.6101507 | 4.101384735 | 0.000357363 | 0.001992371 | -0.098138265 |
| CITED2 | -2.056547276 | 9.007143805 | -4.099959042 | 0.0003587 | 0.001998957 | -0.10174071 |
| ZNF716 | -1.798529307 | 10.09956179 | -4.097941542 | 0.0003606 | 0.002008677 | -0.106838112 |
| SMYD2 | -1.926068063 | 7.92815464 | -4.097595978 | 0.000360926 | 0.002009625 | -0.107711163 |
| ATL1 | -1.798804299 | 5.370447702 | -4.097291293 | 0.000361214 | 0.002010359 | -0.108480921 |
| NEBL | -1.574401626 | 7.715973626 | -4.095606448 | 0.000362812 | 0.002018374 | -0.112737327 |
| VPS37D | 1.91386038 | 6.982079673 | 4.095341263 | 0.000363064 | 0.002018903 | -0.11340723 |
| FCAMR | 2.736400296 | 7.539295245 | 4.094232364 | 0.000364119 | 0.002023899 | -0.116208403 |
| MRC1 | -2.541267548 | 8.24350296 | -4.093912211 | 0.000364425 | 0.002024721 | -0.117017109 |
| C17orf70 | -1.75814201 | 5.492522405 | -4.092802475 | 0.000365485 | 0.002029736 | -0.119820202 |
| DEFB4A | 2.333300689 | 10.58900274 | 4.092176205 | 0.000366085 | 0.00203219 | -0.12140204 |
| PTPN3 | -1.675589415 | 5.052230356 | -4.091796743 | 0.000366449 | 0.002032665 | -0.12236046 |
| ITPRIP | 1.772563669 | 12.67373907 | 4.091757467 | 0.000366486 | 0.002032665 | -0.12245966 |
| B3GNT1 | -2.137694812 | 5.405014796 | -4.090933133 | 0.000367278 | 0.002036178 | -0.124541652 |
| SIPA1L1 | 1.089673005 | 8.039012669 | 4.090495915 | 0.000367699 | 0.002037632 | -0.125645882 |
| VCP | 1.046838526 | 11.25999239 | 4.087856747 | 0.000370248 | 0.002050876 | -0.132310827 |
| RABGGTB | -1.579379347 | 8.685410182 | -4.08590106 | 0.000372149 | 0.002060516 | -0.137249155 |
| LAMA1 | 1.267051022 | 5.851889881 | 4.085117357 | 0.000372913 | 0.002063859 | -0.139227959 |
| MOBP | 2.270216038 | 8.022557414 | 4.084610358 | 0.000373408 | 0.002064856 | -0.140508064 |
| WDR67 | 2.995327479 | 8.8877018 | 4.084604115 | 0.000373414 | 0.002064856 | -0.140523825 |
| RBMS1 | 1.419277389 | 12.76784087 | 4.083964688 | 0.00037404 | 0.002067426 | -0.142138243 |
| NMRAL1 | -1.482284378 | 6.160287588 | -4.083233878 | 0.000374756 | 0.002070495 | -0.143983324 |
| ACP1 | -1.315357846 | 8.207431092 | -4.082729052 | 0.000375252 | 0.002072343 | -0.145257821 |
| CAMSAP2 | -2.080028452 | 6.290846594 | -4.081121434 | 0.000376834 | 0.002079315 | -0.149316247 |
| HPGDS | -2.160147889 | 5.733894443 | -4.081117933 | 0.000376837 | 0.002079315 | -0.149325086 |
| LCE1C | 2.876232869 | 11.30873941 | 4.080246471 | 0.000377698 | 0.002083169 | -0.151524953 |
| SMARCD3 | 1.370148046 | 10.70009448 | 4.079827933 | 0.000378112 | 0.002084017 | -0.152581452 |
| SPOPL | -1.531135477 | 6.421850335 | -4.079763542 | 0.000378176 | 0.002084017 | -0.15274399 |
| NFATC2 | -1.180493261 | 4.669380787 | -4.077937168 | 0.000379988 | 0.002093105 | -0.157353961 |
| DISP2 | 1.631814759 | 6.853289561 | 4.076927904 | 0.000380993 | 0.002097743 | -0.159901278 |
| SLC17A5 | -1.702588537 | 5.881916328 | -4.075718963 | 0.0003822 | 0.002103489 | -0.162952398 |
| C19orf26 | 1.884356838 | 6.457142341 | 4.074688212 | 0.000383232 | 0.002108269 | -0.165553657 |
| FAM57B | 1.919630463 | 8.525976531 | 4.07442498 | 0.000383496 | 0.00210882 | -0.166217942 |
| PAX4 | 2.023069179 | 7.069847108 | 4.072719398 | 0.000385212 | 0.002116817 | -0.170521891 |
| TTLL12 | 1.940745719 | 9.560406203 | 4.072651888 | 0.00038528 | 0.002116817 | -0.17069224 |
| BTN2A3P | 1.591901657 | 7.971499402 | 4.071403384 | 0.00038654 | 0.002122836 | -0.173842534 |
| SDF2L1 | 1.793667081 | 12.87185312 | 4.070969942 | 0.000386979 | 0.002123987 | -0.174936171 |
| VCAN | 1.802000204 | 9.799332542 | 4.070870023 | 0.00038708 | 0.002123987 | -0.175188278 |
| CD47 | -1.833758852 | 7.6228372 | -4.069453808 | 0.000388517 | 0.002130728 | -0.178761414 |
| RAP1GDS1 | -1.799889107 | 6.070542425 | -4.069332955 | 0.00038864 | 0.002130728 | -0.179066315 |
| PDXK | -1.300737459 | 7.834537184 | -4.069126709 | 0.00038885 | 0.00213097 | -0.179586654 |
| CCDC115 | -2.090002576 | 8.522140483 | -4.067703696 | 0.0003903 | 0.002137186 | -0.183176625 |
| PBRM1 | -1.736125863 | 5.907079521 | -4.067687891 | 0.000390316 | 0.002137186 | -0.183216496 |
| MAP7D1 | -1.114042678 | 9.474748713 | -4.066416275 | 0.000391617 | 0.002143395 | -0.186424303 |
| SLC25A4 | -1.762194748 | 7.065703388 | -4.065713841 | 0.000392337 | 0.002145303 | -0.188196191 |
| NGF | 2.292832142 | 8.831540021 | 4.0655903 | 0.000392464 | 0.002145303 | -0.188507816 |
| USPL1 | -1.638581674 | 6.500599337 | -4.065588516 | 0.000392466 | 0.002145303 | -0.188512316 |
| NBEAL1 | -1.454065902 | 11.18476242 | -4.065211223 | 0.000392854 | 0.002146509 | -0.189464004 |
| SIVA1 | -1.638189439 | 8.987253362 | -4.064687696 | 0.000393392 | 0.002147796 | -0.190784527 |
| CAB39 | -1.732033908 | 7.414184103 | -4.064657445 | 0.000393423 | 0.002147796 | -0.190860827 |
| DPCD | -2.724132776 | 5.133617633 | -4.064467221 | 0.000393619 | 0.002147953 | -0.191340631 |
| LOC646513 | 2.426252092 | 12.22725215 | 4.063497829 | 0.000394618 | 0.002152494 | -0.193785661 |
| ING5 | 1.231841855 | 6.979742662 | 4.062683623 | 0.00039546 | 0.002155359 | -0.195839185 |
| TMEM70 | -1.236095365 | 8.387519414 | -4.062665187 | 0.000395479 | 0.002155359 | -0.195885681 |
| KCNK15 | 2.101139866 | 9.473084541 | 4.059750547 | 0.000398505 | 0.002170934 | -0.20323604 |
| ACAD10 | 2.279088862 | 8.77150706 | 4.058862451 | 0.000399432 | 0.002174141 | -0.205475492 |
| GCHFR | -2.419418986 | 6.734003509 | -4.058533343 | 0.000399776 | 0.002175093 | -0.206305356 |
| EIF5B | -1.442802382 | 6.554575024 | -4.055511045 | 0.000402948 | 0.002191037 | -0.213925586 |
| YIPF5 | -1.543936392 | 6.123566096 | -4.055417314 | 0.000403047 | 0.002191037 | -0.214161895 |
| TERT | 1.47204124 | 5.850843713 | 4.055137314 | 0.000403342 | 0.002191716 | -0.214867807 |
| EPT1 | -1.775970599 | 5.97776245 | -4.05490658 | 0.000403586 | 0.002192112 | -0.215449505 |
| LBX2 | 2.109274242 | 6.708753987 | 4.053222377 | 0.000405367 | 0.002200859 | -0.219695305 |
| FAM195B | 1.375786622 | 11.81524628 | 4.052227879 | 0.000406423 | 0.002204868 | -0.222202215 |
| GTF3C5 | -2.198246628 | 5.909301551 | -4.052203686 | 0.000406449 | 0.002204868 | -0.222263199 |
| CHMP4A | -1.023817848 | 9.134194398 | -4.051168338 | 0.00040755 | 0.00220881 | -0.224872945 |
| DEF6 | -1.364451429 | 5.379330891 | -4.051036999 | 0.00040769 | 0.00220881 | -0.225203994 |
| SLFN5 | 1.60523489 | 10.25797848 | 4.049315396 | 0.00040953 | 0.002217841 | -0.229543208 |
| KPNA5 | -1.593938624 | 4.418085241 | -4.049070568 | 0.000409792 | 0.002218327 | -0.230160252 |
| KRT38 | 1.712446171 | 5.756063894 | 4.047911546 | 0.000411036 | 0.002223909 | -0.233081256 |
| OTOS | 1.963078272 | 7.18583701 | 4.047787627 | 0.000411169 | 0.002223909 | -0.233393548 |
| GNAQ | -1.647846974 | 9.563134759 | -4.046819757 | 0.000412211 | 0.002228606 | -0.235832653 |
| SH3D19 | -1.857350087 | 9.376856749 | -4.04647526 | 0.000412582 | 0.002229677 | -0.236700782 |
| TMEM191B | 1.885949023 | 9.172322479 | 4.045696808 | 0.000413423 | 0.002233281 | -0.238662414 |
| SH3TC1 | -1.363289458 | 5.341003989 | -4.045203645 | 0.000413956 | 0.002235223 | -0.239905104 |
| S1PR3 | 2.426113587 | 11.34295781 | 4.043556584 | 0.000415742 | 0.002243926 | -0.24405519 |
| SYT17 | -1.962356694 | 5.772016035 | -4.04150792 | 0.000417975 | 0.002255029 | -0.249216693 |
| CDKN1C | -2.271439735 | 11.2809266 | -4.040584807 | 0.000418984 | 0.002258496 | -0.251542247 |
| SMS | -1.052621309 | 7.509965706 | -4.040280975 | 0.000419317 | 0.002258496 | -0.252307652 |
| CANT1 | 1.157555577 | 6.095378679 | 4.040172694 | 0.000419436 | 0.002258496 | -0.252580428 |
| MAP3K7 | -1.718748315 | 6.995204165 | -4.040118658 | 0.000419495 | 0.002258496 | -0.252716551 |
| CDH12 | 1.976621229 | 6.071536789 | 4.039919158 | 0.000419714 | 0.002258644 | -0.253219115 |
| CCDC102A | -1.314631047 | 5.361314712 | -4.039773466 | 0.000419874 | 0.002258644 | -0.253586128 |
| PTK7 | 1.378976508 | 7.02608581 | 4.03943283 | 0.000420248 | 0.002259711 | -0.254444211 |
| RABEP1 | 1.514921459 | 8.584175439 | 4.038596909 | 0.000421167 | 0.002263195 | -0.256549882 |
| ELAVL3 | 1.594820426 | 6.110435519 | 4.038385135 | 0.0004214 | 0.002263195 | -0.257083324 |
| PEPD | -2.112136678 | 10.32849456 | -4.038363864 | 0.000421424 | 0.002263195 | -0.257136904 |
| LPHN1 | 1.338511899 | 9.260197565 | 4.037676621 | 0.000422181 | 0.002266317 | -0.258867967 |
| SPG11 | -2.267496763 | 7.511700226 | -4.036851032 | 0.000423093 | 0.002268889 | -0.260947421 |
| EXOSC4 | 1.859316407 | 8.720655796 | 4.036615325 | 0.000423354 | 0.002268889 | -0.261541092 |
| ALDH1A1 | -1.818789004 | 9.988411362 | -4.036547142 | 0.000423429 | 0.002268889 | -0.261712824 |
| SYNGR4 | 2.021442465 | 7.459074729 | 4.036469217 | 0.000423516 | 0.002268889 | -0.261909089 |
| MARC2 | -1.673651817 | 7.662397743 | -4.03644498 | 0.000423542 | 0.002268889 | -0.261970134 |
| TCTEX1D2 | -1.267769496 | 7.798281729 | -4.034958336 | 0.000425191 | 0.002276773 | -0.265714315 |
| NOP58 | 1.196310812 | 12.83194253 | 4.034371792 | 0.000425843 | 0.002279317 | -0.26719147 |
| ALPPL2 | 2.019429105 | 9.320008604 | 4.033704034 | 0.000426587 | 0.002282348 | -0.268873103 |
| CLK2 | 1.028539069 | 6.933484885 | 4.033374063 | 0.000426955 | 0.002283367 | -0.269704054 |
| SLC35D2 | -1.791626479 | 5.451819479 | -4.032866229 | 0.000427522 | 0.002285449 | -0.270982883 |
| HSPC081 | 2.222913079 | 7.686747888 | 4.032554193 | 0.000427871 | 0.002286363 | -0.271768635 |
| SPRR2E | 2.031280907 | 7.715668393 | 4.031765365 | 0.000428754 | 0.002290129 | -0.273754964 |
| FRMPD3 | 2.10510512 | 8.746208119 | 4.031324721 | 0.000429248 | 0.002291816 | -0.274864502 |
| H2AFX | 1.80955729 | 10.6882092 | 4.030085387 | 0.00043064 | 0.002298296 | -0.277984995 |
| HDHD2 | -1.544616475 | 8.648886716 | -4.028960951 | 0.000431907 | 0.002304102 | -0.280816011 |
| MRPL24 | -1.286381797 | 7.673536089 | -4.028606747 | 0.000432307 | 0.002305271 | -0.281707764 |
| PTPRZ1 | 2.228537676 | 7.867324944 | 4.028449468 | 0.000432485 | 0.002305271 | -0.282103727 |
| AGBL3 | 2.563074906 | 8.699946596 | 4.028262709 | 0.000432696 | 0.002305441 | -0.282573905 |
| MYL7 | 2.358865581 | 7.645365993 | 4.026364466 | 0.000434847 | 0.00231565 | -0.28735259 |
| ITFG2 | -1.391306097 | 6.549428381 | -4.026254449 | 0.000434972 | 0.00231565 | -0.287629535 |
| TTC35 | -1.982775632 | 7.775870667 | -4.025336539 | 0.000436016 | 0.00232025 | -0.289940116 |
| OR10J5 | 2.179918019 | 6.636588778 | 4.021803417 | 0.000440059 | 0.002340796 | -0.298832707 |
| KDM4C | -1.397891256 | 6.558985052 | -4.02075356 | 0.000441268 | 0.002346253 | -0.301474788 |
| ROBO4 | -1.639305669 | 6.842757803 | -4.020296204 | 0.000441795 | 0.00234715 | -0.302625729 |
| DUSP22 | -1.563461953 | 6.542116974 | -4.02029083 | 0.000441801 | 0.00234715 | -0.302639253 |
| NETO2 | -1.119645212 | 6.40313652 | -4.019302362 | 0.000442944 | 0.002352246 | -0.305126643 |
| PRKRA | -1.390544571 | 8.27415406 | -4.014189611 | 0.000448898 | 0.002382883 | -0.317990302 |
| GLCCI1 | -2.686526433 | 9.054036211 | -4.013433072 | 0.000449786 | 0.00238661 | -0.31989345 |
| APOL2 | 1.754252711 | 11.50858282 | 4.012582756 | 0.000450785 | 0.002390929 | -0.322032406 |
| INSL3 | 2.777119881 | 7.909085497 | 4.011925243 | 0.00045156 | 0.00239405 | -0.323686302 |
| DEGS1 | -1.23855316 | 6.612546772 | -4.01101496 | 0.000452635 | 0.002398358 | -0.325975915 |
| HIST1H4I | 1.254231497 | 7.664466065 | 4.0107015 | 0.000453005 | 0.002398358 | -0.326764326 |
| RNMTL1 | -1.41492018 | 6.831606733 | -4.010693054 | 0.000453015 | 0.002398358 | -0.326785569 |
| KCNMB4 | -2.212418576 | 8.934698567 | -4.010538477 | 0.000453198 | 0.002398358 | -0.327174355 |
| SIGLEC15 | 2.495682405 | 9.971393058 | 4.010448226 | 0.000453305 | 0.002398358 | -0.32740135 |
| CNO | -1.625405626 | 5.434744114 | -4.009394372 | 0.000454554 | 0.002403978 | -0.330051858 |
| C19orf68 | 1.425255213 | 10.03083157 | 4.008984046 | 0.000455041 | 0.002405566 | -0.331083811 |
| FOXD2 | 1.439977426 | 7.477348635 | 4.007764848 | 0.000456492 | 0.002412244 | -0.33414991 |
| ZNF223 | -1.329939207 | 4.761374045 | -4.007422535 | 0.0004569 | 0.002413409 | -0.33501074 |
| APC2 | 1.298484595 | 5.876761785 | 4.006878467 | 0.00045755 | 0.002415848 | -0.336378897 |
| LTV1 | -1.408081591 | 7.352880762 | -4.003773281 | 0.000461274 | 0.002434512 | -0.344186664 |
| NUP98 | -1.335420223 | 6.767687971 | -4.003133204 | 0.000462045 | 0.002437583 | -0.345795927 |
| ANXA11 | -1.454991479 | 8.675661736 | -4.001648804 | 0.000463839 | 0.002445232 | -0.34952774 |
| RAB14 | -1.666410101 | 9.146931069 | -4.001618747 | 0.000463876 | 0.002445232 | -0.349603302 |
| PPP2R5A | -1.821794875 | 10.47944378 | -4.000215865 | 0.000465577 | 0.002452678 | -0.353129891 |
| POLD2 | -1.35019462 | 4.431835289 | -4.000140058 | 0.000465669 | 0.002452678 | -0.353320448 |
| PPP2R5E | -1.333418315 | 8.950816119 | -3.999768945 | 0.000466121 | 0.00245405 | -0.354253308 |
| THBS2 | 3.201605814 | 10.04804259 | 3.999367033 | 0.00046661 | 0.00245562 | -0.355263566 |
| ENHO | 2.272236413 | 11.221962 | 3.998956756 | 0.00046711 | 0.002457246 | -0.356294829 |
| MLF2 | -1.45894997 | 8.439433941 | -3.998791523 | 0.000467311 | 0.002457301 | -0.356710146 |
| ATXN10 | -2.186950397 | 6.660641786 | -3.998621348 | 0.000467519 | 0.002457388 | -0.357137882 |
| ESAM | -1.731472588 | 6.471643985 | -3.998433792 | 0.000467748 | 0.002457587 | -0.357609303 |
| GPR156 | 2.011771066 | 6.903290183 | 3.997873564 | 0.000468432 | 0.002460178 | -0.359017396 |
| VDAC2 | -1.351843913 | 11.00367815 | -3.997657269 | 0.000468697 | 0.002460562 | -0.359561028 |
| RAVER1 | 1.788490842 | 10.79413364 | 3.997128693 | 0.000469344 | 0.002462954 | -0.360889513 |
| POLQ | 1.670688783 | 5.566493139 | 3.996314011 | 0.000470343 | 0.002467189 | -0.362936997 |
| BCAS4 | 1.489055188 | 9.642853074 | 3.996055104 | 0.000470661 | 0.00246785 | -0.363587671 |
| GRPEL2 | 2.617686455 | 10.44063944 | 3.995102222 | 0.000471833 | 0.002472987 | -0.365982332 |
| C17orf46 | 1.652277752 | 5.5727476 | 3.994711105 | 0.000472314 | 0.002474504 | -0.366965202 |
| PLBD2 | 1.80552977 | 8.181909938 | 3.994052077 | 0.000473127 | 0.002477754 | -0.368621276 |
| PTP4A3 | 1.931218743 | 8.168019302 | 3.992190959 | 0.000475431 | 0.002488803 | -0.373297759 |
| TOMM20L | 2.869137221 | 13.03211845 | 3.991382295 | 0.000476435 | 0.002493045 | -0.375329561 |
| PSORS1C2 | 2.40478318 | 8.07622275 | 3.99043812 | 0.00047761 | 0.002498178 | -0.37770172 |
| MYCL1 | 2.015599839 | 6.574368593 | 3.990063705 | 0.000478077 | 0.002499604 | -0.378642374 |
| PDK4 | -3.620899651 | 8.189044153 | -3.989351112 | 0.000478967 | 0.002503238 | -0.380432583 |
| BCL6B | -1.749531151 | 6.409223879 | -3.98811669 | 0.000480512 | 0.002509941 | -0.383533587 |
| KHSRP | 1.213801242 | 12.22350218 | 3.988014773 | 0.000480639 | 0.002509941 | -0.383789604 |
| IMMP2L | -1.193754613 | 5.613025961 | -3.986824226 | 0.000482135 | 0.002516345 | -0.386780166 |
| FLJ11235 | 1.853771663 | 8.319455568 | 3.986726951 | 0.000482257 | 0.002516345 | -0.387024506 |
| LINC00315 | 2.03055224 | 7.173399248 | 3.986017108 | 0.000483151 | 0.002519987 | -0.388807472 |
| CCT4 | -1.474270533 | 12.43667487 | -3.985591269 | 0.000483688 | 0.002521498 | -0.38987705 |
| HS3ST4 | 2.865240905 | 8.09702515 | 3.985476553 | 0.000483833 | 0.002521498 | -0.390165177 |
| BRP44L | -1.288233264 | 10.67476575 | -3.984662391 | 0.000484861 | 0.002525376 | -0.392210018 |
| SEMA3B | -1.42277908 | 10.55041952 | -3.98457687 | 0.00048497 | 0.002525376 | -0.392424807 |
| EIF3E | -1.448097715 | 10.63026807 | -3.984127685 | 0.000485538 | 0.002527314 | -0.39355293 |
| RSF1 | -1.23603762 | 7.719256935 | -3.982882697 | 0.000487118 | 0.002534509 | -0.396679553 |
| EFCAB1 | -2.467231218 | 6.570680485 | -3.981828434 | 0.000488459 | 0.002540461 | -0.399327024 |
| LACTB2 | -2.118115676 | 5.602300795 | -3.980817419 | 0.000489749 | 0.002546139 | -0.401865741 |
| CPT2 | -1.926495643 | 5.283798125 | -3.980029807 | 0.000490756 | 0.002549314 | -0.403843379 |
| LOC158960 | -2.064896663 | 5.16471483 | -3.979463805 | 0.000491481 | 0.002552049 | -0.405264515 |
| MAP4K2 | -1.647821559 | 5.260366065 | -3.978856731 | 0.00049226 | 0.002555062 | -0.406788725 |
| TACR2 | 2.808808547 | 9.122376674 | 3.978449449 | 0.000492783 | 0.002556447 | -0.407811279 |
| ADK | -2.020153055 | 5.853174102 | -3.978339547 | 0.000492924 | 0.002556447 | -0.408087204 |
| EMCN | -2.09839681 | 8.528132247 | -3.977123345 | 0.00049449 | 0.002563535 | -0.411140533 |
| NGDN | -2.053298056 | 8.374657213 | -3.974055572 | 0.000498462 | 0.002582044 | -0.418841372 |
| NAT14 | -1.619077203 | 5.992934529 | -3.972785252 | 0.000500116 | 0.002589568 | -0.422029779 |
| WHSC1 | -1.034790996 | 5.256484973 | -3.971765327 | 0.000501447 | 0.002595419 | -0.424589546 |
| TAAR5 | 2.382080671 | 8.109736536 | 3.971568561 | 0.000501705 | 0.002595485 | -0.425083364 |
| C6orf203 | -1.010014654 | 7.659316622 | -3.971446971 | 0.000501864 | 0.002595485 | -0.425388513 |
| GPATCH3 | -1.682814717 | 5.183046202 | -3.968134812 | 0.000506216 | 0.002616943 | -0.433700049 |
| GPR116 | -1.285519863 | 8.216831796 | -3.9675935 | 0.000506931 | 0.002619586 | -0.435058267 |
| PHF19 | 1.129437582 | 7.351243733 | 3.966986109 | 0.000507734 | 0.002622683 | -0.436582233 |
| PTGFRN | 1.9635237 | 7.113525374 | 3.962736863 | 0.00051339 | 0.002648834 | -0.447242254 |
| MAVS | 1.148162169 | 8.497881066 | 3.962717843 | 0.000513415 | 0.002648834 | -0.447289964 |
| LHFPL5 | 1.731816247 | 7.192644321 | 3.961783274 | 0.000514667 | 0.00265423 | -0.449634148 |
| AKNAD1 | 2.751855371 | 7.317543167 | 3.960455126 | 0.000516452 | 0.002662366 | -0.452965329 |
| ENOSF1 | -1.192577607 | 8.530201322 | -3.959949784 | 0.000517132 | 0.002664807 | -0.454232729 |
| RRP7B | 2.046653879 | 8.501984916 | 3.95968266 | 0.000517493 | 0.002665594 | -0.454902662 |
| PFDN6 | -1.024226373 | 7.972930415 | -3.959287727 | 0.000518025 | 0.002667271 | -0.455893115 |
| PRPS1 | -1.478950941 | 7.644227905 | -3.959107304 | 0.000518269 | 0.002667458 | -0.45634559 |
| IKBKB | 1.115318322 | 7.315494457 | 3.958409846 | 0.000519212 | 0.002671242 | -0.458094671 |
| SLC22A17 | 2.055628882 | 8.136190476 | 3.957475852 | 0.000520477 | 0.002676681 | -0.460436822 |
| CD52 | -2.604828738 | 12.28185159 | -3.957142744 | 0.000520929 | 0.002677934 | -0.461272118 |
| EBI3 | 1.742594508 | 7.657259366 | 3.956635941 | 0.000521618 | 0.002680402 | -0.462542936 |
| SNX24 | -1.414938428 | 5.013944915 | -3.955503612 | 0.000523159 | 0.00268658 | -0.465382135 |
| NSMAF | -1.474292153 | 7.525044039 | -3.955445999 | 0.000523238 | 0.00268658 | -0.46552659 |
| DUSP9 | 2.07429683 | 7.00221765 | 3.954727437 | 0.000524218 | 0.00269054 | -0.467328207 |
| ZNF14 | -1.135267216 | 6.616526029 | -3.953885261 | 0.00052537 | 0.002695375 | -0.469439659 |
| UCP3 | 1.150819601 | 7.437023551 | 3.951146472 | 0.000529132 | 0.002712514 | -0.476305456 |
| KRTAP6-3 | 2.740642689 | 10.00741096 | 3.950775757 | 0.000529643 | 0.002714053 | -0.477234706 |
| HEBP2 | 1.190190578 | 11.91485864 | 3.950487932 | 0.000530041 | 0.002715007 | -0.477956166 |
| PCNXL2 | 1.276723921 | 8.415504343 | 3.949927261 | 0.000530815 | 0.002717893 | -0.479361507 |
| C22orf13 | -1.488077101 | 7.653899881 | -3.948978395 | 0.000532129 | 0.002723536 | -0.481739764 |
| NUDT12 | 2.25196173 | 8.705145756 | 3.948815821 | 0.000532355 | 0.002723606 | -0.482147229 |
| BNIP3L | -1.945768067 | 9.37654102 | -3.948625008 | 0.000532619 | 0.002723876 | -0.482625465 |
| ATR | -1.370841983 | 7.43632669 | -3.946685876 | 0.000535316 | 0.002735837 | -0.487485227 |
| RDH16 | 2.173364225 | 7.003286465 | 3.946637886 | 0.000535383 | 0.002735837 | -0.48760549 |
| TMEM2 | -1.448175153 | 8.796161088 | -3.945071617 | 0.000537572 | 0.00274593 | -0.491530378 |
| WBP2 | 2.984096378 | 10.68574495 | 3.943213198 | 0.00054018 | 0.002758158 | -0.496186886 |
| LOC100507780 | 1.545611455 | 6.504940013 | 3.942926185 | 0.000540584 | 0.002759125 | -0.49690599 |
| CHCHD1 | -1.397367783 | 9.523329111 | -3.942387868 | 0.000541343 | 0.002761743 | -0.498254692 |
| TBC1D9 | -1.185878823 | 7.481250288 | -3.942257383 | 0.000541527 | 0.002761743 | -0.498581603 |
| LRRC45 | 1.508987322 | 7.224656723 | 3.941986047 | 0.00054191 | 0.002762177 | -0.499261388 |
| ANGPTL1 | -1.530985828 | 6.939509457 | -3.941892681 | 0.000542041 | 0.002762177 | -0.499495299 |
| ITGB2 | 1.30199114 | 7.03757497 | 3.941633759 | 0.000542407 | 0.002762334 | -0.50014397 |
| C19orf6 | -1.417211531 | 8.323702672 | -3.941566735 | 0.000542502 | 0.002762334 | -0.50031188 |
| SAAL1 | -1.35682771 | 5.219482078 | -3.940949583 | 0.000543374 | 0.002765683 | -0.501857967 |
| TRMT12 | -1.725952969 | 6.144893379 | -3.939766302 | 0.000545051 | 0.002773121 | -0.504822161 |
| YWHAB | -1.363669401 | 11.98279267 | -3.93936356 | 0.000545623 | 0.002774933 | -0.505831006 |
| FRMD4A | 1.415410709 | 8.880923698 | 3.938805414 | 0.000546417 | 0.002777871 | -0.507229088 |
| MSRB2 | -1.069607978 | 9.091913158 | -3.938501806 | 0.000546849 | 0.002778436 | -0.507989567 |
| FOXG1 | 2.183624615 | 6.407031768 | 3.938256173 | 0.000547199 | 0.002778552 | -0.508604821 |
| PELP1 | 3.102601502 | 11.35422818 | 3.938058161 | 0.000547481 | 0.002778888 | -0.509100787 |
| DHRS7B | -1.109478111 | 7.062854808 | -3.937872488 | 0.000547746 | 0.002779135 | -0.509565843 |
| FLJ10038 | -1.64597991 | 7.554463733 | -3.937102594 | 0.000548845 | 0.002783614 | -0.511494144 |
| GXYLT1 | -1.814664896 | 4.937336184 | -3.936656427 | 0.000549483 | 0.002785751 | -0.512611585 |
| MSLNL | 2.219242888 | 7.626707522 | 3.936459385 | 0.000549765 | 0.002786082 | -0.513105075 |
| FOXC2 | 3.220800053 | 8.408062105 | 3.936107968 | 0.000550268 | 0.002787534 | -0.513985181 |
| PCDH12 | -1.904630685 | 4.826240401 | -3.935447455 | 0.000551215 | 0.002791079 | -0.515639353 |
| SLC5A2 | 2.779845225 | 9.072703258 | 3.935317498 | 0.000551402 | 0.002791079 | -0.515964806 |
| PPP1R3D | 1.272340133 | 9.098020166 | 3.93515242 | 0.000551639 | 0.00279118 | -0.516378208 |
| LOC220906 | -1.951521841 | 6.117326786 | -3.934060251 | 0.00055321 | 0.002798027 | -0.519113212 |
| LRFN1 | 2.762211769 | 8.156297674 | 3.933727773 | 0.000553689 | 0.00279935 | -0.519945766 |
| DTX2 | 1.346759676 | 6.175836671 | 3.93288448 | 0.000554906 | 0.0028044 | -0.522057372 |
| ZNF92 | -1.792923604 | 11.37605987 | -3.932230381 | 0.000555851 | 0.002807732 | -0.52369516 |
| TP53BP1 | -1.555224658 | 6.037119597 | -3.932126532 | 0.000556002 | 0.002807732 | -0.52395518 |
| HHIP | -4.095230618 | 6.430891216 | -3.931911776 | 0.000556313 | 0.0028082 | -0.524492886 |
| ST5 | -1.837227516 | 7.366642477 | -3.931741032 | 0.00055656 | 0.002808346 | -0.524920393 |
| GPT | 1.91287797 | 7.675049327 | 3.931040559 | 0.000557576 | 0.002812369 | -0.526674171 |
| CCDC107 | -1.513403279 | 6.991914255 | -3.929435577 | 0.00055991 | 0.002823036 | -0.530692297 |
| C16orf71 | 2.059243828 | 8.924626398 | 3.92771816 | 0.000562419 | 0.002834392 | -0.534991474 |
| HMGCR | -1.292382776 | 8.551571843 | -3.927592114 | 0.000562603 | 0.002834392 | -0.535306985 |
| LDB3 | 1.236986279 | 6.927058399 | 3.927315838 | 0.000563008 | 0.002835321 | -0.535998532 |
| MAFK | 1.157418189 | 7.994235521 | 3.926964099 | 0.000563524 | 0.002836807 | -0.536878956 |
| MDP1 | -1.46656575 | 7.862606398 | -3.926670896 | 0.000563954 | 0.002837862 | -0.537612848 |
| NOL6 | 1.801195693 | 6.707228901 | 3.926074749 | 0.00056483 | 0.002840537 | -0.53910497 |
| KRTAP19-1 | 1.375379693 | 6.647001193 | 3.926008335 | 0.000564927 | 0.002840537 | -0.539271197 |
| LOC100130856 | 2.212109612 | 10.88362424 | 3.925832651 | 0.000565186 | 0.002840726 | -0.539710912 |
| SOCS6 | -1.744941784 | 5.611940358 | -3.922908819 | 0.000569503 | 0.002861306 | -0.547028205 |
| USP31 | 1.107684756 | 7.744759915 | 3.921662027 | 0.000571353 | 0.002869483 | -0.550148075 |
| SEC14L4 | -2.726552562 | 7.095802634 | -3.920842042 | 0.000572574 | 0.00287449 | -0.552199808 |
| NME2P1 | 2.008327463 | 10.15343541 | 3.920148494 | 0.000573608 | 0.002878559 | -0.553935096 |
| DCAF4L2 | 2.862959952 | 6.290571399 | 3.919875246 | 0.000574016 | 0.002879483 | -0.554618752 |
| ZNF683 | 2.416460533 | 8.036880957 | 3.919695894 | 0.000574284 | 0.002879703 | -0.55506748 |
| LGR6 | 1.920555006 | 8.277554058 | 3.919252183 | 0.000574947 | 0.002881906 | -0.556177595 |
| OCEL1 | -1.848471935 | 5.2324592 | -3.91901267 | 0.000575306 | 0.00288258 | -0.556776816 |
| GBP2 | 2.18492598 | 10.9005135 | 3.917667039 | 0.000577323 | 0.002891563 | -0.560143196 |
| LMLN | -2.623532229 | 5.57337391 | -3.917515991 | 0.00057755 | 0.002891574 | -0.560521056 |
| THOC3 | -1.836363962 | 8.099618458 | -3.916223752 | 0.000579495 | 0.002900182 | -0.563753572 |
| FAM47A | 2.945189901 | 7.898993183 | 3.915207016 | 0.00058103 | 0.002906733 | -0.56629674 |
| ZNF524 | 1.072939605 | 9.773775001 | 3.915015207 | 0.00058132 | 0.002907053 | -0.566776493 |
| KRT3 | 2.705914762 | 11.12485374 | 3.914759616 | 0.000581707 | 0.002907856 | -0.567415772 |
| PANK2 | -1.198724492 | 7.613902082 | -3.914004417 | 0.000582851 | 0.002912442 | -0.5693046 |
| TSPAN13 | -1.277227796 | 8.917352303 | -3.913539032 | 0.000583557 | 0.002914322 | -0.570468531 |
| ASGR1 | 1.823149392 | 8.4264295 | 3.913457826 | 0.00058368 | 0.002914322 | -0.570671623 |
| TINF2 | -1.316384537 | 9.614253607 | -3.913142507 | 0.000584159 | 0.002914679 | -0.571460215 |
| NDUFS3 | -2.177027984 | 8.356348397 | -3.913112402 | 0.000584205 | 0.002914679 | -0.571535503 |
| NDP | 2.911564219 | 6.998209345 | 3.910617236 | 0.000588009 | 0.00293252 | -0.577775186 |
| LAPTM5 | -2.315855635 | 8.421997305 | -3.909889232 | 0.000589123 | 0.00293597 | -0.579595529 |
| CHRM4 | 1.808717111 | 7.212288244 | 3.909867081 | 0.000589157 | 0.00293597 | -0.579650916 |
| MRPS34 | 1.061922049 | 11.76078901 | 3.908133287 | 0.00059182 | 0.002948097 | -0.583985854 |
| ACHE | 1.836877407 | 7.123457385 | 3.906908229 | 0.000593708 | 0.002956359 | -0.587048538 |
| TOX3 | 2.011346565 | 7.480591742 | 3.906334377 | 0.000594595 | 0.002959629 | -0.588483106 |
| GYLTL1B | 1.927256091 | 7.76653131 | 3.90602253 | 0.000595077 | 0.002960884 | -0.589262668 |
| LOC100500938 | 2.277103799 | 8.078347064 | 3.905786261 | 0.000595443 | 0.002961558 | -0.589853289 |
| POLK | -1.787722275 | 4.965795492 | -3.905373082 | 0.000596083 | 0.002963596 | -0.590886122 |
| TCEA2 | -1.604792266 | 5.432038668 | -3.904970433 | 0.000596708 | 0.002965554 | -0.591892609 |
| HIST2H2AB | 1.023746287 | 7.001520478 | 3.904314801 | 0.000597726 | 0.002967811 | -0.593531414 |
| CCDC86 | 2.233199118 | 12.89203757 | 3.904232402 | 0.000597854 | 0.002967811 | -0.593737373 |
| CDK5R1 | 2.143571877 | 6.296553012 | 3.904021246 | 0.000598182 | 0.002968295 | -0.594265156 |
| IDH1 | -2.564913888 | 6.992305354 | -3.903326084 | 0.000599265 | 0.002972518 | -0.596002666 |
| LZTS2 | 1.58965686 | 13.42238216 | 3.90274773 | 0.000600166 | 0.002975844 | -0.597448161 |
| SPOCD1 | 2.225368955 | 9.313385696 | 3.901426823 | 0.000602231 | 0.002983396 | -0.600749346 |
| DDX5 | -1.61879089 | 12.09537822 | -3.901369982 | 0.00060232 | 0.002983396 | -0.600891395 |
| GBA3 | 2.42694242 | 8.449096653 | 3.901328362 | 0.000602385 | 0.002983396 | -0.600995406 |
| C19orf39 | -1.150447968 | 6.03364258 | -3.901170835 | 0.000602632 | 0.00298347 | -0.601389073 |
| STK33 | -2.125818413 | 6.377028156 | -3.899783963 | 0.000604809 | 0.002992075 | -0.604854762 |
| PPYR1 | 1.928487891 | 6.848526856 | 3.899767076 | 0.000604835 | 0.002992075 | -0.60489696 |
| EIF3A | -1.497976943 | 12.05843558 | -3.899291293 | 0.000605584 | 0.002994626 | -0.606085834 |
| CHD3 | 1.234034126 | 6.771058038 | 3.89879046 | 0.000606373 | 0.002997375 | -0.607337267 |
| ZFYVE19 | 1.121276578 | 9.109182185 | 3.898308054 | 0.000607134 | 0.002999984 | -0.608542618 |
| DHRS1 | -1.544094605 | 7.215517877 | -3.896813289 | 0.000609498 | 0.003010507 | -0.612277234 |
| CCDC8 | 1.126220567 | 7.891557227 | 3.895734728 | 0.000611209 | 0.0030178 | -0.614971761 |
| LRRN4 | -1.855596062 | 4.089229727 | -3.894993445 | 0.000612388 | 0.00302246 | -0.616823574 |
| STK35 | -1.59677896 | 4.615279688 | -3.894728089 | 0.00061281 | 0.003023385 | -0.617486444 |
| C5AR1 | 1.429207602 | 8.119020486 | 3.890716884 | 0.000619232 | 0.003052153 | -0.627505222 |
| ISOC1 | -1.424751148 | 7.944919766 | -3.890641872 | 0.000619353 | 0.003052153 | -0.627692555 |
| LIFR | -1.919410488 | 8.349986524 | -3.890472331 | 0.000619626 | 0.003052329 | -0.628115958 |
| MAOA | -2.054355049 | 9.196558418 | -3.890315994 | 0.000619878 | 0.0030524 | -0.628506382 |
| TTLL3 | 2.265117214 | 13.62194077 | 3.889658813 | 0.000620937 | 0.003056447 | -0.630147536 |
| ANKRD17 | -1.37546689 | 7.733957454 | -3.886042354 | 0.0006268 | 0.003084124 | -0.639177546 |
| PAPPA | 2.393751198 | 6.914971141 | 3.884641613 | 0.000629085 | 0.003093521 | -0.642674523 |
| AKD1 | -1.314747247 | 5.643455571 | -3.884577157 | 0.000629191 | 0.003093521 | -0.64283543 |
| NPTXR | 1.877775889 | 7.157649929 | 3.882181088 | 0.000633119 | 0.003111648 | -0.648816504 |
| C15orf24 | -2.013610875 | 10.48371027 | -3.881811366 | 0.000633728 | 0.003113448 | -0.649739325 |
| RASAL3 | 2.219181659 | 11.6295337 | 3.879633576 | 0.000637323 | 0.003129916 | -0.655174598 |
| PTRF | 1.672065054 | 13.54261283 | 3.879467617 | 0.000637598 | 0.00313007 | -0.655588764 |
| F12 | 1.899708215 | 7.335848001 | 3.878734315 | 0.000638814 | 0.003134842 | -0.657418727 |
| ARHGAP9 | 1.656511004 | 10.77648384 | 3.877742574 | 0.000640461 | 0.003140678 | -0.659893492 |
| TAOK1 | -1.168530854 | 9.041565215 | -3.876846544 | 0.000641953 | 0.003146648 | -0.662129286 |
| NES | -1.527338714 | 7.335254567 | -3.875214973 | 0.000644679 | 0.003158806 | -0.66620008 |
| KATNA1 | -1.861149186 | 7.571077421 | -3.874806554 | 0.000645364 | 0.003160954 | -0.667219024 |
| ASNA1 | -1.327636975 | 5.837869411 | -3.873569348 | 0.00064744 | 0.003169919 | -0.670305503 |
| C1orf182 | 2.465689809 | 10.77493151 | 3.873049119 | 0.000648316 | 0.003172997 | -0.671603252 |
| GDF1 | 1.868264599 | 6.401443681 | 3.872457396 | 0.000649313 | 0.003176667 | -0.673079296 |
| C3orf15 | 1.765257385 | 7.266426944 | 3.871700471 | 0.00065059 | 0.003181707 | -0.674967351 |
| DDX47 | -1.343292415 | 9.873035528 | -3.86983923 | 0.000653742 | 0.003195905 | -0.679609583 |
| C11orf88 | -2.39247239 | 8.555530799 | -3.869279485 | 0.000654693 | 0.003199337 | -0.681005565 |
| SUPT4H1 | 1.008311479 | 11.2247976 | 3.867143784 | 0.000658333 | 0.003214683 | -0.686331453 |
| ZFAND6 | -2.069120313 | 8.992464258 | -3.866441447 | 0.000659534 | 0.003219328 | -0.688082736 |
| FAM105B | 1.87156847 | 8.48141041 | 3.866247366 | 0.000659867 | 0.003219728 | -0.688566663 |
| CRIP1 | -1.568169399 | 10.15423988 | -3.863767211 | 0.000664129 | 0.003239295 | -0.694750225 |
| MCM2 | -1.644826361 | 4.528781934 | -3.863209796 | 0.00066509 | 0.003241743 | -0.696139842 |
| GPX5 | 1.915285508 | 6.89460473 | 3.863184121 | 0.000665135 | 0.003241743 | -0.696203847 |
| ZBED4 | -1.527948831 | 4.956753116 | -3.862950318 | 0.000665538 | 0.003242482 | -0.696786695 |
| CRHR2 | 2.950472396 | 8.848981839 | 3.861963566 | 0.000667245 | 0.003249567 | -0.699246466 |
| HBP1 | -1.110265032 | 7.775048405 | -3.861418928 | 0.000668189 | 0.003252932 | -0.70060407 |
| ZNF518A | -1.316176529 | 6.403950717 | -3.860881734 | 0.000669121 | 0.003255889 | -0.701943067 |
| FIGF | -2.325523538 | 8.847975815 | -3.860582237 | 0.000669641 | 0.003255889 | -0.702689566 |
| TNPO1 | -1.278154019 | 8.750786166 | -3.860378616 | 0.000669995 | 0.003255889 | -0.703197087 |
| CYP24A1 | 2.525207385 | 7.200676388 | 3.860340341 | 0.000670062 | 0.003255889 | -0.703292484 |
| HOMER1 | -1.742024667 | 6.450372041 | -3.858473956 | 0.000673315 | 0.003270462 | -0.707944062 |
| ProSAPiP1 | 2.743974872 | 8.971033465 | 3.858031083 | 0.00067409 | 0.003272033 | -0.709047748 |
| OPN4 | 2.302730614 | 6.624680509 | 3.857997982 | 0.000674148 | 0.003272033 | -0.709130236 |
| DPM1 | -1.65992759 | 8.279205458 | -3.857456334 | 0.000675096 | 0.003275399 | -0.710480032 |
| COX17 | 2.376420808 | 12.14429824 | 3.857197907 | 0.000675549 | 0.00327636 | -0.711124018 |
| CIAPIN1 | -1.065139926 | 7.25721297 | -3.856168668 | 0.000677355 | 0.003282731 | -0.713688717 |
| SP5 | 2.029298487 | 7.012163664 | 3.856158756 | 0.000677373 | 0.003282731 | -0.713713415 |
| ITGB7 | 1.810166258 | 8.844479497 | 3.855893891 | 0.000677838 | 0.003283751 | -0.714373389 |
| CORT | 1.628856935 | 7.71116933 | 3.855023914 | 0.00067937 | 0.003289933 | -0.716541055 |
| MRPL43 | -1.338051006 | 10.3495038 | -3.853978661 | 0.000681215 | 0.003297626 | -0.719145282 |
| SPATA13 | -1.457477417 | 6.256633025 | -3.853773818 | 0.000681577 | 0.003298137 | -0.719655621 |
| E2F7 | 2.388569941 | 7.25705091 | 3.852297687 | 0.000684193 | 0.003309548 | -0.72333301 |
| NKX1-2 | 2.614508546 | 9.350371722 | 3.851968417 | 0.000684777 | 0.003311131 | -0.72415325 |
| PRDM6 | -1.371314442 | 6.383549069 | -3.85096279 | 0.000686566 | 0.003318533 | -0.72665824 |
| ANXA4 | -1.537584219 | 10.20760049 | -3.850681652 | 0.000687067 | 0.003319266 | -0.727358515 |
| ASNS | 1.364654633 | 11.20621167 | 3.850588014 | 0.000687234 | 0.003319266 | -0.727591752 |
| CLIP3 | 1.34116623 | 5.443739783 | 3.850360186 | 0.00068764 | 0.003319982 | -0.728159227 |
| CIZ1 | 2.403926589 | 11.68524693 | 3.849437485 | 0.000689288 | 0.003326689 | -0.730457409 |
| SUPT5H | -1.091442183 | 7.536274028 | -3.848958215 | 0.000690146 | 0.003329578 | -0.731651075 |
| RASD2 | 2.023910311 | 7.345555084 | 3.848704735 | 0.0006906 | 0.003330519 | -0.732282376 |
| WFS1 | -1.801117108 | 7.385112001 | -3.848472435 | 0.000691016 | 0.003331277 | -0.732860916 |
| COPB2 | -1.816609153 | 9.247689134 | -3.848267069 | 0.000691384 | 0.003331803 | -0.733372372 |
| BCL7C | -1.423686264 | 5.559757758 | -3.848100237 | 0.000691683 | 0.003331997 | -0.733787855 |
| CORO7 | 1.276775672 | 7.025000401 | 3.847815581 | 0.000692194 | 0.00333321 | -0.734496757 |
| TNNC1 | -2.421210991 | 7.47051655 | -3.847287227 | 0.000693144 | 0.003336532 | -0.735812528 |
| ZNF418 | 1.494286754 | 8.246551242 | 3.847117237 | 0.000693449 | 0.003336754 | -0.736235845 |
| WDR46 | -1.333589732 | 3.941681785 | -3.84627477 | 0.000694966 | 0.003342803 | -0.738333735 |
| SORT1 | -1.928270612 | 8.373947216 | -3.845878454 | 0.000695681 | 0.00334499 | -0.739320589 |
| GLTPD1 | 2.102309181 | 9.411424792 | 3.845606384 | 0.000696172 | 0.0033461 | -0.739998047 |
| AKT2 | 1.313855818 | 10.51741987 | 3.845326147 | 0.000696678 | 0.003347282 | -0.740695828 |
| TMEM114 | 2.548965101 | 8.07674385 | 3.843945226 | 0.000699178 | 0.003358006 | -0.744134084 |
| IFITM4P | -1.555992343 | 9.163198012 | -3.843804857 | 0.000699432 | 0.003358006 | -0.74448356 |
| RFTN2 | -1.421297953 | 4.583000375 | -3.84268437 | 0.000701468 | 0.003366521 | -0.747273118 |
| SERPINA2 | 2.032757064 | 6.294929852 | 3.841273883 | 0.000704038 | 0.003377596 | -0.750784356 |
| SPSB1 | 1.350151156 | 10.36022802 | 3.840819585 | 0.000704868 | 0.003380255 | -0.751915205 |
| RASSF1 | 1.935028253 | 12.58838318 | 3.840682889 | 0.000705118 | 0.003380255 | -0.752255464 |
| CLDN3 | 1.667183541 | 7.984641655 | 3.840305023 | 0.000705809 | 0.003382308 | -0.753196021 |
| SPI1 | 1.369171017 | 8.264412119 | 3.839936922 | 0.000706483 | 0.003384277 | -0.754112248 |
| C11orf1 | -1.465586126 | 5.000191305 | -3.839565233 | 0.000707164 | 0.003386279 | -0.755037384 |
| OR6M1 | 1.849028645 | 6.659497369 | 3.838972518 | 0.000708252 | 0.003389274 | -0.756512605 |
| CXCL14 | 2.523321453 | 10.63161882 | 3.838505038 | 0.000709111 | 0.003391811 | -0.757676085 |
| TRIM29 | 2.015614403 | 7.827380031 | 3.838055243 | 0.000709938 | 0.003394507 | -0.758795513 |
| LINC00152 | 1.198548147 | 8.950856763 | 3.837394402 | 0.000711155 | 0.003398042 | -0.760440123 |
| ZBP1 | 1.738177373 | 5.730333274 | 3.837367041 | 0.000711206 | 0.003398042 | -0.760508212 |
| LOC113230 | 1.483220142 | 7.498417806 | 3.836590968 | 0.000712638 | 0.003403622 | -0.762439498 |
| INS | 2.731618649 | 7.613451934 | 3.83550707 | 0.000714644 | 0.003411932 | -0.765136642 |
| PLA2G1B | -3.454943482 | 8.944414595 | -3.835331074 | 0.00071497 | 0.003412223 | -0.765574566 |
| KIAA1609 | 1.0491797 | 8.005045893 | 3.833112374 | 0.000719094 | 0.003430631 | -0.771094835 |
| RNF19B | 2.08866131 | 8.286864246 | 3.832936212 | 0.000719422 | 0.003430925 | -0.771533103 |
| CLEC2D | 1.068138548 | 10.38366764 | 3.831471898 | 0.000722158 | 0.003442695 | -0.775175908 |
| ZNF467 | 2.766222376 | 9.552448526 | 3.830868242 | 0.000723288 | 0.003445987 | -0.776677527 |
| MCM5 | -1.518784836 | 6.698660483 | -3.830817312 | 0.000723384 | 0.003445987 | -0.776804215 |
| RSBN1 | -1.217736775 | 4.861585555 | -3.829465687 | 0.000725922 | 0.003456799 | -0.780166204 |
| RASSF3 | -1.106293373 | 9.348992826 | -3.828921853 | 0.000726946 | 0.003460393 | -0.781518832 |
| PSMF1 | -1.297205771 | 5.796211604 | -3.828431263 | 0.000727871 | 0.003461531 | -0.782738987 |
| SDHD | -1.181570047 | 7.305231901 | -3.828271768 | 0.000728172 | 0.003461531 | -0.78313566 |
| UROD | -1.357524972 | 9.270831514 | -3.828250548 | 0.000728212 | 0.003461531 | -0.783188435 |
| UBFD1 | 1.198728136 | 8.814550844 | 3.828224165 | 0.000728262 | 0.003461531 | -0.783254051 |
| SFR1 | -1.638308141 | 6.345558093 | -3.82779732 | 0.000729068 | 0.003464082 | -0.784315615 |
| BAD | -1.442816372 | 9.374368382 | -3.827137386 | 0.000730316 | 0.00346873 | -0.785956813 |
| DUSP2 | 2.17568134 | 9.782878978 | 3.826336969 | 0.000731832 | 0.003473367 | -0.787947276 |
| PIP4K2A | 1.378858673 | 9.660543549 | 3.824988963 | 0.000734393 | 0.003482722 | -0.791299228 |
| LAX1 | 1.673964038 | 6.739565594 | 3.824901044 | 0.00073456 | 0.003482722 | -0.791517838 |
| DDX11L2 | 1.808097843 | 7.007475719 | 3.824731879 | 0.000734882 | 0.003482722 | -0.791938458 |
| STAG3L1 | 1.128586572 | 7.211196033 | 3.824729626 | 0.000734886 | 0.003482722 | -0.791944059 |
| BAZ2A | -1.082439528 | 5.920767185 | -3.824260482 | 0.00073578 | 0.003485674 | -0.793110539 |
| MT3 | 1.927889776 | 9.804270712 | 3.822401136 | 0.000739333 | 0.003501217 | -0.797733243 |
| TCTE3 | 2.308275418 | 8.084674219 | 3.821884044 | 0.000740324 | 0.003503625 | -0.799018729 |
| ERN2 | 2.402903822 | 7.347766224 | 3.821851607 | 0.000740387 | 0.003503625 | -0.799099366 |
| MEX3D | 1.934501486 | 9.417344348 | 3.82146567 | 0.000741127 | 0.003505466 | -0.800058771 |
| KAT7 | -1.190401668 | 5.949345079 | -3.82136483 | 0.000741321 | 0.003505466 | -0.800309445 |
| LNX2 | -1.085103359 | 6.151554958 | -3.821112184 | 0.000741806 | 0.003506472 | -0.800937481 |
| GLUD2 | -1.164783294 | 6.878710911 | -3.820640024 | 0.000742714 | 0.003509474 | -0.802111166 |
| RHOH | 1.441177373 | 8.704325906 | 3.819962022 | 0.00074402 | 0.003514352 | -0.803796459 |
| SIAE | -1.195654696 | 3.930768293 | -3.817866063 | 0.00074807 | 0.003532187 | -0.809005828 |
| TNRC18 | 1.42499676 | 9.444441313 | 3.817534528 | 0.000748713 | 0.003533925 | -0.809829767 |
| IL1R1 | 1.249410208 | 10.06541427 | 3.816188958 | 0.000751327 | 0.003544961 | -0.813173613 |
| UQCR10 | -1.249017886 | 13.16428524 | -3.813892578 | 0.000755808 | 0.003564799 | -0.818879573 |
| HYOU1 | 1.541137379 | 7.136430592 | 3.813632847 | 0.000756317 | 0.00356589 | -0.819524883 |
| TLK2 | 1.473334127 | 8.230945117 | 3.813088321 | 0.000757384 | 0.003569614 | -0.820877742 |
| PIGK | -2.153564355 | 5.537578062 | -3.810822337 | 0.000761841 | 0.003589305 | -0.826506955 |
| PP14571 | 2.449986342 | 8.908788232 | 3.809890928 | 0.000763681 | 0.003596655 | -0.828820522 |
| COL4A1 | 1.300906996 | 10.26182394 | 3.809537885 | 0.000764379 | 0.003598626 | -0.829697424 |
| GGNBP1 | 2.030982776 | 5.11952031 | 3.808091528 | 0.000767247 | 0.003609858 | -0.833289704 |
| NSUN4 | -1.067001881 | 5.774145933 | -3.808051564 | 0.000767326 | 0.003609858 | -0.833388954 |
| NOXO1 | 1.845949727 | 8.061498412 | 3.807702266 | 0.000768021 | 0.003611803 | -0.834256441 |
| RNF168 | 1.510764819 | 8.047505253 | 3.807125874 | 0.000769168 | 0.003614941 | -0.835687868 |
| ACTN4 | -1.134320788 | 7.700000982 | -3.807084468 | 0.00076925 | 0.003614941 | -0.835790695 |
| TMEM161B | -1.569279834 | 6.243424669 | -3.806405623 | 0.000770603 | 0.003619977 | -0.83747648 |
| CYP3A7 | -1.892488459 | 6.409543356 | -3.805105045 | 0.000773202 | 0.003630859 | -0.840705995 |
| STAG3L2 | 1.358426313 | 7.576357882 | 3.804118135 | 0.00077518 | 0.003636632 | -0.843156428 |
| METTL9 | -1.280588821 | 6.866096147 | -3.804113455 | 0.000775189 | 0.003636632 | -0.843168048 |
| OR2A9P | -1.862319878 | 5.789281601 | -3.804012719 | 0.000775391 | 0.003636632 | -0.843418159 |
| IL11RA | -2.000016364 | 7.756372208 | -3.803927582 | 0.000775562 | 0.003636632 | -0.84362954 |
| OVOL2 | -2.003804267 | 6.091218961 | -3.803082767 | 0.00077726 | 0.003643265 | -0.845726997 |
| SEC16B | 3.180884461 | 12.95439103 | 3.801828514 | 0.000779787 | 0.00365378 | -0.84884075 |
| MSI1 | 1.675714413 | 5.845824299 | 3.799946189 | 0.000783595 | 0.00366895 | -0.853513207 |
| CCL19 | 2.903901603 | 11.98772027 | 3.799659659 | 0.000784177 | 0.003670335 | -0.8542244 |
| DBP | 1.898060501 | 7.792126079 | 3.799504586 | 0.000784491 | 0.003670473 | -0.854609296 |
| RBM22 | 3.030421923 | 10.5537459 | 3.799188451 | 0.000785134 | 0.003671831 | -0.855393946 |
| IREB2 | 1.652168127 | 8.975878761 | 3.799080554 | 0.000785353 | 0.003671831 | -0.855661742 |
| SIN3A | -1.28440859 | 5.218097095 | -3.797461059 | 0.000788651 | 0.003685912 | -0.859681032 |
| PKLR | 3.152048359 | 7.737858395 | 3.796005727 | 0.000791626 | 0.003698474 | -0.863292501 |
| UBAC1 | -1.792584905 | 7.577754661 | -3.795191393 | 0.000793296 | 0.003704929 | -0.86531314 |
| LOC400550 | -2.024135739 | 6.527593785 | -3.794777676 | 0.000794146 | 0.003707551 | -0.86633967 |
| ANKRD43 | 2.874120631 | 9.206818466 | 3.794535797 | 0.000794643 | 0.003708526 | -0.866939812 |
| TMEM200A | 1.405170081 | 7.946707307 | 3.793062846 | 0.000797677 | 0.003721335 | -0.870594234 |
| RNASET2 | -1.605689118 | 8.373705044 | -3.792651852 | 0.000798526 | 0.003722743 | -0.871613851 |
| IQGAP3 | 1.655104405 | 6.718282846 | 3.792636296 | 0.000798558 | 0.003722743 | -0.871652442 |
| USP19 | 1.789310317 | 7.87898322 | 3.791551667 | 0.000800801 | 0.003731851 | -0.8743431 |
| RPS27L | -1.384492163 | 11.12175382 | -3.791227159 | 0.000801474 | 0.003733632 | -0.875148072 |
| EPHA2 | -1.725866266 | 4.017016055 | -3.790659782 | 0.000802651 | 0.003737762 | -0.876555456 |
| ID1 | -1.896636328 | 12.24347384 | -3.789543273 | 0.000804973 | 0.003746547 | -0.879324801 |
| LOC388630 | 2.587917838 | 10.81753247 | 3.789472471 | 0.00080512 | 0.003746547 | -0.879500407 |
| FAM174B | -1.139387262 | 8.195959781 | -3.788697112 | 0.000806736 | 0.00375271 | -0.881423436 |
| TMEM95 | 1.853727511 | 7.831509382 | 3.787867108 | 0.00080847 | 0.003759416 | -0.883481874 |
| PHF16 | 3.020005548 | 11.7142445 | 3.787408968 | 0.000809429 | 0.003762513 | -0.884618025 |
| ANP32B | -1.171747338 | 7.762702387 | -3.787264249 | 0.000809732 | 0.003762561 | -0.884976909 |
| ZIC4 | 2.291414601 | 6.854096195 | 3.785833979 | 0.000812733 | 0.003775141 | -0.888523584 |
| ESPNL | 1.571994042 | 6.692033278 | 3.785105528 | 0.000814265 | 0.003780894 | -0.890329799 |
| CEL | 2.001626102 | 8.11106928 | 3.784576984 | 0.000815379 | 0.003782779 | -0.891640279 |
| MLEC | 1.480321756 | 10.92649877 | 3.784496354 | 0.000815549 | 0.003782779 | -0.89184019 |
| CROCCP3 | 1.639874501 | 7.613631395 | 3.784494248 | 0.000815553 | 0.003782779 | -0.891845412 |
| RPL6 | -1.194646369 | 12.26913202 | -3.784315696 | 0.00081593 | 0.003783162 | -0.892288101 |
| SGCD | 1.969017726 | 10.05503861 | 3.784071178 | 0.000816446 | 0.00378419 | -0.892894336 |
| MLL2 | 1.238858851 | 6.933127749 | 3.783742291 | 0.000817141 | 0.003786046 | -0.893709728 |
| HSF4 | 1.407214792 | 7.028677597 | 3.782958737 | 0.000818798 | 0.003792358 | -0.895652274 |
| KRT14 | 1.921397748 | 6.427453266 | 3.781892488 | 0.000821058 | 0.00380121 | -0.898295486 |
| TFR2 | 1.982441859 | 8.96636342 | 3.781778723 | 0.0008213 | 0.00380121 | -0.898577495 |
| F7 | 3.008569741 | 9.613754476 | 3.780851241 | 0.000823272 | 0.003808293 | -0.900876519 |
| DHX33 | -1.987873605 | 4.818730753 | -3.780780433 | 0.000823423 | 0.003808293 | -0.901052029 |
| IRF3 | 1.142598867 | 7.265310734 | 3.780596339 | 0.000823815 | 0.003808736 | -0.901508338 |
| GAMT | 1.023677417 | 7.158689391 | 3.780379167 | 0.000824277 | 0.003809506 | -0.902046626 |
| IFNGR2 | -1.504693157 | 6.883315121 | -3.779430159 | 0.000826302 | 0.003817493 | -0.904398767 |
| MS4A2 | -1.71253496 | 5.670264386 | -3.778390981 | 0.000828525 | 0.003826144 | -0.906974209 |
| ESCO1 | -1.178261558 | 8.688767269 | -3.776949778 | 0.000831618 | 0.003837914 | -0.910545683 |
| NDEL1 | -1.09546938 | 6.008275007 | -3.775330427 | 0.000835106 | 0.003852631 | -0.91455818 |
| UBE2E3 | -1.61414242 | 9.705309886 | -3.774373068 | 0.000837175 | 0.003860791 | -0.916930141 |
| ATP7B | -1.333225353 | 6.379799443 | -3.773745066 | 0.000838535 | 0.003865677 | -0.918485993 |
| NUP88 | -1.269233125 | 7.468477895 | -3.773526768 | 0.000839008 | 0.003865915 | -0.919026803 |
| PIWIL3 | 2.675252531 | 7.696179455 | 3.773443975 | 0.000839188 | 0.003865915 | -0.91923191 |
| LOC100505746 | 1.785178865 | 6.406264903 | 3.772582726 | 0.000841058 | 0.003873142 | -0.921365459 |
| CCDC61 | 2.817559819 | 10.39579678 | 3.771822205 | 0.000842713 | 0.003879374 | -0.923249365 |
| FBXO7 | -1.461860883 | 10.72776125 | -3.771463613 | 0.000843494 | 0.003879867 | -0.924137606 |
| TCEAL3 | -1.574316463 | 9.501453531 | -3.771169414 | 0.000844135 | 0.003879867 | -0.924866326 |
| EPB41L4A-AS1 | -1.320205039 | 11.34723248 | -3.771150123 | 0.000844178 | 0.003879867 | -0.92491411 |
| FMO1 | 2.65271083 | 10.88863639 | 3.771088668 | 0.000844312 | 0.003879867 | -0.925066328 |
| SYT3 | 2.047038542 | 8.359707704 | 3.771081118 | 0.000844328 | 0.003879867 | -0.92508503 |
| RNPC3 | 1.398125065 | 8.971115396 | 3.769853253 | 0.000847011 | 0.003890807 | -0.928126216 |
| CCM2 | -1.098988388 | 6.38142934 | -3.768653717 | 0.000849641 | 0.003901493 | -0.93109697 |
| AIP | -1.600833299 | 7.739910442 | -3.768427334 | 0.000850138 | 0.003902382 | -0.931657598 |
| GNA15 | 1.463944602 | 7.056137119 | 3.766048801 | 0.000855379 | 0.003922416 | -0.937547361 |
| TSEN15 | -1.106840551 | 8.187967344 | -3.765878454 | 0.000855755 | 0.003922416 | -0.937969138 |
| C11orf49 | -1.270283232 | 6.458805673 | -3.765824205 | 0.000855875 | 0.003922416 | -0.938103457 |
| MMRN1 | -1.921401471 | 5.517952175 | -3.765622745 | 0.000856321 | 0.003922416 | -0.938602258 |
| KRT86 | 1.809333483 | 6.784238272 | 3.765617605 | 0.000856332 | 0.003922416 | -0.938614985 |
| IGSF9 | 1.566564615 | 7.444963144 | 3.764077635 | 0.000859746 | 0.00393665 | -0.942427608 |
| CPT1A | 1.111924483 | 8.254574761 | 3.763624601 | 0.000860752 | 0.003939857 | -0.943549137 |
| BVES | -2.133567454 | 4.170237025 | -3.761769666 | 0.000864887 | 0.003956854 | -0.948140809 |
| XPO6 | 1.198042263 | 6.90276664 | 3.76168264 | 0.000865081 | 0.003956854 | -0.948356215 |
| RPL27 | -1.559294367 | 13.83077289 | -3.760964134 | 0.000866688 | 0.003962795 | -0.95013461 |
| LOC643037 | -2.888506448 | 6.358091409 | -3.760713809 | 0.000867249 | 0.003963949 | -0.950754174 |
| MAP7D2 | 2.566238504 | 7.662355375 | 3.760411887 | 0.000867925 | 0.003964252 | -0.951501426 |
| RTEL1 | 1.195818837 | 6.898998701 | 3.760409146 | 0.000867931 | 0.003964252 | -0.951508212 |
| LOC100506571 | 1.855869495 | 5.146108538 | 3.760055877 | 0.000868724 | 0.003966462 | -0.952382527 |
| FBP1 | -2.87005317 | 10.45702483 | -3.759788896 | 0.000869323 | 0.003967789 | -0.953043268 |
| DOK6 | -1.413019532 | 7.637349564 | -3.758794608 | 0.000871558 | 0.00397658 | -0.955503885 |
| ERBB2 | 1.152851527 | 10.03324305 | 3.757657287 | 0.000874122 | 0.003985879 | -0.958318249 |
| ARID1B | 1.097510465 | 8.964451871 | 3.757607827 | 0.000874233 | 0.003985879 | -0.958440635 |
| C6orf48 | -1.494435588 | 11.5694132 | -3.757478314 | 0.000874526 | 0.003985879 | -0.958761105 |
| UBB | -1.250382104 | 13.54728538 | -3.75700179 | 0.000875603 | 0.003989374 | -0.959940205 |
| ABLIM1 | -1.536422487 | 11.79746 | -3.752987844 | 0.000884725 | 0.004029509 | -0.969870525 |
| ZNF668 | 2.394715578 | 8.353342906 | 3.75240168 | 0.000886065 | 0.004034183 | -0.971320418 |
| ADI1 | -1.169637548 | 8.288851254 | -3.751903843 | 0.000887205 | 0.004037942 | -0.972551781 |
| PNISR | 1.648076339 | 13.56874337 | 3.750550702 | 0.000890309 | 0.004050639 | -0.975898442 |
| GNB1 | -2.166247729 | 8.39348279 | -3.74927709 | 0.000893241 | 0.004062541 | -0.979048095 |
| ITIH5 | -1.220388683 | 5.77806381 | -3.749007635 | 0.000893863 | 0.004063931 | -0.97971442 |
| CAMK2G | -1.24352723 | 7.663278579 | -3.748312226 | 0.000895469 | 0.004069794 | -0.981434009 |
| WDR74 | -1.245291383 | 5.58475999 | -3.747964911 | 0.000896272 | 0.004072005 | -0.982292805 |
| CD248 | 2.175567028 | 10.63981457 | 3.747151212 | 0.000898156 | 0.004077694 | -0.984304731 |
| LOC440335 | -1.77252496 | 7.308354872 | -3.747150376 | 0.000898158 | 0.004077694 | -0.984306797 |
| ECI2 | -1.619777406 | 8.928432226 | -3.746308993 | 0.000900111 | 0.004085117 | -0.986387043 |
| CSNK2B | -1.073615581 | 8.259524118 | -3.746067218 | 0.000900672 | 0.004086225 | -0.986984785 |
| YWHAH | -1.854263985 | 9.12016508 | -3.7450432 | 0.000903056 | 0.004095594 | -0.989516354 |
| C10orf91 | 1.891703379 | 7.098440535 | 3.744681226 | 0.0009039 | 0.004097977 | -0.990411175 |
| TAGLN3 | 2.908964742 | 7.08256317 | 3.743632218 | 0.00090635 | 0.004107209 | -0.993004246 |
| GPR25 | 1.95447393 | 8.250712299 | 3.743536085 | 0.000906575 | 0.004107209 | -0.993241872 |
| FAM53B | 1.762480074 | 6.90925671 | 3.742971419 | 0.000907897 | 0.00411175 | -0.994637588 |
| CHRM1 | 1.94540964 | 7.037978685 | 3.741487695 | 0.00091138 | 0.004125408 | -0.998304708 |
| ABHD11 | 2.836346166 | 10.9967597 | 3.741413504 | 0.000911554 | 0.004125408 | -0.998488067 |
| TGM3 | 2.016957936 | 6.517471302 | 3.740933714 | 0.000912683 | 0.004129065 | -0.999673802 |
| JDP2 | 1.49641161 | 12.25577818 | 3.739147126 | 0.0009169 | 0.004146684 | -1.004088733 |
| GAST | 2.249158577 | 6.499020653 | 3.738773186 | 0.000917785 | 0.004149227 | -1.005012718 |
| ARMCX6 | -1.455324633 | 7.58526486 | -3.738516048 | 0.000918394 | 0.004150522 | -1.005648078 |
| OR5F1 | 2.068539345 | 6.443918746 | 3.738103831 | 0.000919371 | 0.004153478 | -1.006666594 |
| ZMAT1 | -1.749428509 | 5.778152616 | -3.737753607 | 0.000920202 | 0.004154671 | -1.007531909 |
| LOC100129516 | 2.407236025 | 7.409340511 | 3.737720342 | 0.000920281 | 0.004154671 | -1.007614099 |
| DCST2 | 1.982624567 | 9.491147216 | 3.737524291 | 0.000920747 | 0.004155314 | -1.00809848 |
| TMEM194A | -1.271007194 | 4.540658991 | -3.736162089 | 0.000923988 | 0.004168384 | -1.011463864 |
| MPDZ | -1.273860202 | 6.735572143 | -3.736003637 | 0.000924366 | 0.004168384 | -1.011855305 |
| CEP164 | 1.45702223 | 7.456446069 | 3.735899048 | 0.000924615 | 0.004168384 | -1.012113678 |
| RGS3 | -1.589598197 | 8.055894804 | -3.734893573 | 0.000927016 | 0.004177745 | -1.014597474 |
| MME | -2.973276348 | 5.862203755 | -3.734052549 | 0.000929029 | 0.004184098 | -1.016674887 |
| TJAP1 | -1.155749707 | 6.507817724 | -3.734032894 | 0.000929077 | 0.004184098 | -1.016723433 |
| RSBN1L | 2.203092003 | 8.631061504 | 3.733671239 | 0.000929944 | 0.004186537 | -1.017616713 |
| CDR1 | 1.544113526 | 5.419717842 | 3.732448879 | 0.00093288 | 0.004196818 | -1.020635734 |
| PGD | -1.246831705 | 7.492887384 | -3.731955553 | 0.000934068 | 0.004199688 | -1.021854083 |
| TEP1 | 1.415060531 | 8.620743767 | 3.731912685 | 0.000934171 | 0.004199688 | -1.021959951 |
| TLX2 | 1.670313015 | 7.029141247 | 3.731484637 | 0.000935203 | 0.004202857 | -1.023017045 |
| IQCK | -1.363956758 | 7.881494886 | -3.730234999 | 0.000938221 | 0.004213793 | -1.026102911 |
| C18orf23 | 1.402811151 | 11.5158117 | 3.730155623 | 0.000938413 | 0.004213793 | -1.026298913 |
| C16orf57 | 1.120299452 | 10.39825192 | 3.730070694 | 0.000938619 | 0.004213793 | -1.026508625 |
| PAFAH1B3 | -1.853508433 | 4.618618316 | -3.729856087 | 0.000939139 | 0.004214655 | -1.027038539 |
| OR1D2 | 2.00892489 | 7.038653207 | 3.72965694 | 0.000939621 | 0.004215328 | -1.027530274 |
| SMEK1 | 1.004186007 | 9.50757674 | 3.729523604 | 0.000939944 | 0.004215328 | -1.027859501 |
| TTYH1 | 2.245520832 | 7.719431957 | 3.728371226 | 0.000942741 | 0.004226399 | -1.030704772 |
| VEGFC | -1.960790693 | 8.545512014 | -3.727867331 | 0.000943967 | 0.004230419 | -1.031948831 |
| RAC3 | 1.511325677 | 6.178858513 | 3.727122618 | 0.000945781 | 0.004237074 | -1.033787352 |
| LTB4R2 | 1.61807155 | 6.422929364 | 3.72631034 | 0.000947764 | 0.004244478 | -1.035792556 |
| TRIM28 | -1.260497105 | 7.043250188 | -3.723588389 | 0.000954438 | 0.004272879 | -1.042511083 |
| KRTAP4-1 | 2.30730273 | 7.818295644 | 3.723305284 | 0.000955135 | 0.004274511 | -1.043209785 |
| PLXNB2 | -1.15243138 | 8.531676099 | -3.723097237 | 0.000955647 | 0.004275317 | -1.043723233 |
| DPH5 | -1.481771965 | 5.601290262 | -3.722020422 | 0.000958304 | 0.004285709 | -1.046380615 |
| GJD3 | 1.579199045 | 7.943930226 | 3.721330604 | 0.000960009 | 0.004291844 | -1.048082842 |
| RARA | 1.404045077 | 10.16452525 | 3.720605834 | 0.000961804 | 0.004298375 | -1.049871219 |
| ZNF84 | -1.157967643 | 5.694110468 | -3.719763471 | 0.000963894 | 0.004306221 | -1.051949632 |
| UFSP2 | -1.157935081 | 7.645964631 | -3.719556732 | 0.000964408 | 0.00430702 | -1.05245971 |
| KCNK3 | -1.34474366 | 7.03648117 | -3.719290468 | 0.00096507 | 0.00430848 | -1.053116641 |
| VWF | -1.65297711 | 10.5902376 | -3.717913089 | 0.000968502 | 0.004321162 | -1.056514709 |
| FOXD3 | 1.815919219 | 6.775482735 | 3.71772106 | 0.000968981 | 0.004321162 | -1.056988426 |
| SFT2D1 | -1.781387547 | 7.12293134 | -3.717419879 | 0.000969734 | 0.004321162 | -1.057731396 |
| MEI1 | 1.417885876 | 9.104648347 | 3.717357228 | 0.00096989 | 0.004321162 | -1.057885945 |
| PGF | 1.715214821 | 6.559857763 | 3.717242926 | 0.000970176 | 0.004321162 | -1.058167904 |
| COA5 | -1.645564705 | 6.482159434 | -3.717217949 | 0.000970238 | 0.004321162 | -1.058229517 |
| PPARD | 1.115613603 | 9.580359605 | 3.717181976 | 0.000970328 | 0.004321162 | -1.058318255 |
| SOBP | -1.342326559 | 5.044948833 | -3.717073954 | 0.000970598 | 0.004321162 | -1.05858472 |
| NEDD4L | -2.150206409 | 10.42517738 | -3.715352282 | 0.000974914 | 0.004338873 | -1.062831383 |
| SNX2 | -1.313964381 | 9.867326149 | -3.713752547 | 0.000978941 | 0.004354228 | -1.066776759 |
| TNS1 | 1.499515589 | 10.32121038 | 3.713712748 | 0.000979041 | 0.004354228 | -1.066874909 |
| IRS4 | 2.322510616 | 7.826941486 | 3.713044566 | 0.000980728 | 0.004360222 | -1.068522674 |
| ATF2 | -1.624019019 | 7.254592002 | -3.712607674 | 0.000981832 | 0.004363624 | -1.06960002 |
| HN1L | -1.171126678 | 9.384635891 | -3.712294353 | 0.000982625 | 0.004364903 | -1.070372626 |
| SDK2 | 2.379274698 | 9.027785417 | 3.712225758 | 0.000982799 | 0.004364903 | -1.070541768 |
| C2orf68 | -1.379575546 | 6.213194151 | -3.711240093 | 0.000985298 | 0.00437449 | -1.072972144 |
| SLC17A7 | 2.587630943 | 9.317548824 | 3.710790873 | 0.000986439 | 0.004378044 | -1.074079731 |
| PDCD11 | 1.478025212 | 9.940096262 | 3.709677866 | 0.000989271 | 0.0043891 | -1.076823772 |
| AQP2 | 2.831138485 | 9.286132002 | 3.708397372 | 0.000992539 | 0.004402082 | -1.079980441 |
| ALDH2 | -1.786533321 | 11.85254906 | -3.707611853 | 0.000994549 | 0.004409347 | -1.081916741 |
| TMX2 | -1.081773831 | 7.414505496 | -3.707358542 | 0.000995198 | 0.004409347 | -1.082541125 |
| YBX2 | 1.227365648 | 6.073876407 | 3.707355652 | 0.000995206 | 0.004409347 | -1.082548247 |
| VPS39 | -1.257549189 | 5.171055399 | -3.706586529 | 0.000997179 | 0.004416569 | -1.084443977 |
| SNCB | 1.853960541 | 6.376959922 | 3.705456505 | 0.001000085 | 0.004425349 | -1.087229039 |
| SERBP1 | -1.037552063 | 9.79570498 | -3.705414389 | 0.001000194 | 0.004425349 | -1.087332834 |
| NCK1 | -1.92673808 | 6.963023032 | -3.704263965 | 0.001003161 | 0.004435969 | -1.090167909 |
| SSR3 | -1.388220256 | 5.640146975 | -3.704216564 | 0.001003284 | 0.004435969 | -1.090284718 |
| GYPE | -1.605888764 | 4.204388036 | -3.702673275 | 0.001007279 | 0.004452046 | -1.094087535 |
| LCN1 | 2.273065451 | 7.038043717 | 3.702544738 | 0.001007612 | 0.004452046 | -1.094404243 |
| CIAO1 | 1.05701917 | 10.80998175 | 3.702344537 | 0.001008132 | 0.004452812 | -1.094897518 |
| MAP3K8 | -1.216771117 | 6.741432463 | -3.700588573 | 0.0010127 | 0.004471453 | -1.099223707 |
| KRTAP2-1 | 2.419988928 | 7.512250473 | 3.699848549 | 0.001014631 | 0.004478443 | -1.10104673 |
| TCOF1 | 1.438980802 | 9.296948133 | 3.698797246 | 0.001017381 | 0.00448904 | -1.103636394 |
| SYT5 | 2.125586907 | 8.297125071 | 3.698403421 | 0.001018413 | 0.004492052 | -1.104606443 |
| KCNN2 | 1.748518055 | 7.560800129 | 3.697513957 | 0.001020747 | 0.004500806 | -1.106797209 |
| LOC100129726 | 2.538216994 | 10.91736972 | 3.696578899 | 0.001023207 | 0.004510105 | -1.109100108 |
| RUFY1 | -1.614479225 | 9.536748073 | -3.696418018 | 0.001023631 | 0.004510428 | -1.109496313 |
| CDC37 | -1.245713872 | 7.930340293 | -3.69600176 | 0.001024728 | 0.004513717 | -1.110521422 |
| ZNF688 | 1.666370221 | 11.62990565 | 3.695181915 | 0.001026893 | 0.004520798 | -1.112540332 |
| ZNF79 | -1.289096584 | 4.59109031 | -3.694993756 | 0.00102739 | 0.004520798 | -1.113003665 |
| TWF2 | -1.102792762 | 5.442240955 | -3.693579012 | 0.001031138 | 0.004535737 | -1.116487178 |
| CAD | -1.082429181 | 5.704843703 | -3.693370278 | 0.001031692 | 0.004536622 | -1.117001108 |
| MNX1 | 2.201107449 | 6.720073598 | 3.693232704 | 0.001032057 | 0.004536678 | -1.117339829 |
| ODF3L2 | 1.772190644 | 7.743873154 | 3.690679144 | 0.001038861 | 0.004564335 | -1.123626259 |
| TNNI1 | 2.105171101 | 6.858176256 | 3.690605322 | 0.001039059 | 0.004564335 | -1.123807979 |
| C7orf43 | 1.756941673 | 6.911602606 | 3.689132794 | 0.001043003 | 0.004580097 | -1.12743248 |
| SBNO2 | 2.118776904 | 8.5281618 | 3.688929012 | 0.00104355 | 0.004580935 | -1.127934037 |
| PLK3 | 1.646108571 | 10.22981959 | 3.688558532 | 0.001044545 | 0.004583739 | -1.12884586 |
| TMEM86A | 2.060658419 | 7.508069241 | 3.688245934 | 0.001045386 | 0.004584343 | -1.129615202 |
| GRIN2D | 2.105457429 | 10.27214584 | 3.688242189 | 0.001045396 | 0.004584343 | -1.129624419 |
| COX6C | -1.356962589 | 12.07613924 | -3.687274339 | 0.001048002 | 0.004594206 | -1.13200629 |
| PLEKHM3 | -1.213440682 | 6.158669562 | -3.685203871 | 0.001053599 | 0.004617168 | -1.137101072 |
| LOC100506123 | 1.535085008 | 6.058007434 | 3.68440287 | 0.001055772 | 0.004624018 | -1.139071857 |
| CHTOP | -1.47513633 | 8.235667201 | -3.684362741 | 0.001055881 | 0.004624018 | -1.139170587 |
| CSTF2 | -1.503222901 | 5.732703184 | -3.683606948 | 0.001057936 | 0.004631439 | -1.141030019 |
| HLA-F | -1.764520884 | 8.817435479 | -3.682564438 | 0.001060776 | 0.004642294 | -1.143594653 |
| PDHX | -1.374315091 | 6.737215637 | -3.682078516 | 0.001062103 | 0.004646518 | -1.144789975 |
| KRTAP5-9 | 2.094082861 | 6.854524479 | 3.681926546 | 0.001062518 | 0.004646754 | -1.145163798 |
| PSMB8 | -1.456029404 | 5.701034241 | -3.681457781 | 0.0010638 | 0.004650778 | -1.146316856 |
| MDFI | 1.623699201 | 6.69003312 | 3.681137058 | 0.001064677 | 0.004653034 | -1.147105739 |
| EEF2 | -1.219309854 | 9.579239996 | -3.679406661 | 0.001069426 | 0.004672199 | -1.151361637 |
| DSTN | -1.354115224 | 13.5368497 | -3.678663568 | 0.001071471 | 0.004679545 | -1.153189086 |
| C9orf53 | 2.230296341 | 6.711313261 | 3.678402908 | 0.00107219 | 0.004681093 | -1.153830085 |
| SUMF1 | -1.755904381 | 8.682965669 | -3.677812357 | 0.001073819 | 0.004686616 | -1.155282285 |
| CDR2L | -1.056387147 | 8.026095979 | -3.676619891 | 0.001077116 | 0.004699413 | -1.158214413 |
| ILF2 | 1.765288107 | 12.75023184 | 3.675813478 | 0.001079352 | 0.00470757 | -1.160197119 |
| JARID2 | 1.142154643 | 9.835966771 | 3.67498219 | 0.001081661 | 0.004713379 | -1.16224085 |
| USP33 | 1.151139113 | 10.99099564 | 3.674938304 | 0.001081783 | 0.004713379 | -1.162348741 |
| RTBDN | 2.948883584 | 12.55307053 | 3.674600953 | 0.001082722 | 0.004713695 | -1.163178079 |
| EIF3G | 1.827538277 | 11.99316038 | 3.674517066 | 0.001082955 | 0.004713695 | -1.163384301 |
| C16orf55 | -1.310332988 | 5.295913982 | -3.67401738 | 0.001084347 | 0.004718158 | -1.164612671 |
| LRRC59 | -1.6046938 | 5.767506936 | -3.673491711 | 0.001085814 | 0.00472294 | -1.165904857 |
| GUK1 | 1.186051644 | 13.60856967 | 3.673281177 | 0.001086401 | 0.004723899 | -1.166422372 |
| NUP133 | -1.327592405 | 8.458043886 | -3.672743909 | 0.001087903 | 0.004728829 | -1.167742993 |
| ZSWIM6 | 1.108216319 | 8.960142163 | 3.672155082 | 0.001089551 | 0.004734392 | -1.169190276 |
| SPAG5 | 1.550809433 | 7.617794124 | 3.671706319 | 0.001090808 | 0.004738256 | -1.17029325 |
| ATP5J2 | -1.017805162 | 9.161524351 | -3.670834631 | 0.001093255 | 0.00474728 | -1.172435575 |
| FAAH2 | -1.533327572 | 6.56928075 | -3.669945743 | 0.001095755 | 0.004756532 | -1.174620012 |
| MYOG | 1.875578484 | 6.418769623 | 3.668413272 | 0.001100079 | 0.004773692 | -1.178385674 |
| SIX6 | 2.698309847 | 12.1636067 | 3.666865028 | 0.001104465 | 0.004791106 | -1.18218961 |
| SLITRK4 | -1.231766017 | 4.760242661 | -3.666528319 | 0.001105421 | 0.004792145 | -1.183016818 |
| LOC730755 | 1.880790041 | 7.12518399 | 3.666352263 | 0.001105921 | 0.004792145 | -1.183449332 |
| SLC15A3 | -1.150907405 | 8.193222081 | -3.666263685 | 0.001106173 | 0.004792145 | -1.183666939 |
| GOLGA6L10 | 1.514161422 | 7.814801931 | 3.666255975 | 0.001106195 | 0.004792145 | -1.183685881 |
| TMBIM4 | -1.686504076 | 7.509828746 | -3.665667234 | 0.00110787 | 0.004797784 | -1.185132177 |
| NAPSB | -1.981725181 | 9.802665721 | -3.664721278 | 0.001110566 | 0.004806223 | -1.187455857 |
| GRIN2C | 1.561285402 | 5.241581641 | 3.662413748 | 0.001117169 | 0.004833174 | -1.193123391 |
| C2orf73 | -1.784677658 | 4.754258003 | -3.661986913 | 0.001118395 | 0.00483685 | -1.194171622 |
| MAS1L | 2.423837458 | 6.17306242 | 3.661574734 | 0.00111958 | 0.004839348 | -1.195183823 |
| WIBG | 1.016529536 | 9.129351676 | 3.661410222 | 0.001120053 | 0.004839348 | -1.195587812 |
| ACVR1B | 1.866807178 | 7.29694488 | 3.661393438 | 0.001120101 | 0.004839348 | -1.195629029 |
| TMEM9 | -1.115163758 | 8.506977546 | -3.660186729 | 0.001123578 | 0.004851113 | -1.198592144 |
| ZNF691 | -1.243825936 | 5.795959 | -3.658966412 | 0.001127106 | 0.004861447 | -1.201588369 |
| KIF26A | -1.15754867 | 6.061567689 | -3.658379351 | 0.001128807 | 0.00486715 | -1.203029662 |
| DRD5 | 2.287164093 | 6.998807278 | 3.656258077 | 0.001134973 | 0.004892099 | -1.208237009 |
| CD97 | -1.447408069 | 9.223198635 | -3.654968809 | 0.001138737 | 0.004906662 | -1.211401478 |
| NKX2-3 | 1.97290433 | 7.259083131 | 3.654775132 | 0.001139303 | 0.004906662 | -1.211876822 |
| MRPS25 | 1.181968937 | 11.64221571 | 3.654709234 | 0.001139496 | 0.004906662 | -1.212038555 |
| ARHGAP5 | -1.515868523 | 6.751818336 | -3.654082827 | 0.00114133 | 0.004912916 | -1.213575893 |
| GPHA2 | 1.765883992 | 6.640453906 | 3.653533101 | 0.001142943 | 0.004918209 | -1.214924975 |
| SIGLEC1 | 1.621765953 | 7.169917488 | 3.652618409 | 0.00114563 | 0.004928125 | -1.217169578 |
| ITGA5 | 1.697092131 | 8.787720321 | 3.652109604 | 0.001147128 | 0.004932917 | -1.218418081 |
| TCEAL6 | -1.485621726 | 9.016909131 | -3.650409169 | 0.001152146 | 0.004952843 | -1.222590215 |
| BAIAP2L1 | 1.035645976 | 7.02912491 | 3.648675266 | 0.001157286 | 0.004973275 | -1.226843847 |
| C6orf165 | -1.815522187 | 6.667790368 | -3.648093975 | 0.001159014 | 0.004979038 | -1.228269738 |
| C1orf31 | -1.236074751 | 8.684245544 | -3.647631559 | 0.001160391 | 0.004983287 | -1.229403982 |
| PNMA5 | 2.012984402 | 7.725208244 | 3.647408086 | 0.001161056 | 0.004984482 | -1.229952113 |
| LOC390705 | 1.164780838 | 7.577484959 | 3.646481092 | 0.001163822 | 0.004993229 | -1.232225726 |
| LMF1 | 1.944940388 | 7.526537125 | 3.646464953 | 0.00116387 | 0.004993229 | -1.232265308 |
| HIST2H4B | 1.187187788 | 9.104342793 | 3.646246746 | 0.001164522 | 0.00499436 | -1.232800471 |
| LOC100287525 | -1.887517929 | 4.872436159 | -3.645650653 | 0.001166305 | 0.00500034 | -1.234262369 |
| EFTUD2 | 1.487205007 | 10.88399477 | 3.645133237 | 0.001167855 | 0.005005315 | -1.235531254 |
| SNF8 | 1.054670374 | 9.619445372 | 3.644940857 | 0.001168432 | 0.005006119 | -1.236003024 |
| BARHL1 | 2.075585433 | 8.41665855 | 3.644486959 | 0.001169794 | 0.005010285 | -1.237116075 |
| CACNB3 | 1.756295438 | 7.50263298 | 3.644138777 | 0.001170839 | 0.005013094 | -1.23796986 |
| ARL4C | 1.243963056 | 7.885789054 | 3.643181379 | 0.001173719 | 0.005023753 | -1.240317387 |
| NUPR1 | -2.071031892 | 12.88241052 | -3.642454786 | 0.00117591 | 0.005031454 | -1.242098858 |
| RBBP7 | -1.817585195 | 9.969468151 | -3.641361768 | 0.001179212 | 0.005043907 | -1.244778522 |
| TEAD3 | 1.056241083 | 8.634722266 | 3.640981145 | 0.001180365 | 0.005045679 | -1.245711608 |
| BECN1 | -1.56702698 | 8.558808765 | -3.64096576 | 0.001180411 | 0.005045679 | -1.245749323 |
| GYPC | 1.473735213 | 8.297779623 | 3.640611911 | 0.001181483 | 0.005048584 | -1.246616744 |
| EPS8L1 | 2.212562187 | 10.8701946 | 3.640097137 | 0.001183045 | 0.005053577 | -1.247878607 |
| TOMM22 | 1.085441679 | 9.197518561 | 3.6398994 | 0.001183645 | 0.005054463 | -1.248363305 |
| TMEM108 | -1.434783988 | 6.875186725 | -3.639641373 | 0.001184429 | 0.005056131 | -1.248995774 |
| HMOX1 | 2.185295123 | 12.37723319 | 3.639408294 | 0.001185137 | 0.005057477 | -1.249567078 |
| TMEM45A | -1.609917691 | 5.822484308 | -3.639211158 | 0.001185737 | 0.005058357 | -1.250050274 |
| ABCC10 | 2.037311052 | 9.111077901 | 3.638116922 | 0.00118907 | 0.005070895 | -1.252732182 |
| WASH7P | 1.215252444 | 5.896427725 | 3.637559019 | 0.001190773 | 0.005076474 | -1.254099473 |
| EPHX1 | -2.006802846 | 7.401289094 | -3.637408814 | 0.001191232 | 0.005076748 | -1.25446758 |
| TMEM38A | 2.269655457 | 8.228195211 | 3.637267381 | 0.001191664 | 0.005076908 | -1.254814184 |
| TMEM121 | 1.139349656 | 8.873844939 | 3.636550468 | 0.001193858 | 0.005084569 | -1.256571032 |
| ROR2 | 1.992045972 | 5.820860043 | 3.634910691 | 0.00119889 | 0.005103854 | -1.260589018 |
| TRHR | 2.1409615 | 5.62232527 | 3.634816366 | 0.00119918 | 0.005103854 | -1.260820128 |
| SECTM1 | 2.178164433 | 11.84403815 | 3.634653214 | 0.001199681 | 0.005104301 | -1.26121987 |
| SIRPG | 1.71998874 | 8.026726582 | 3.634172718 | 0.001201161 | 0.005108906 | -1.262397109 |
| SSC5D | 2.684992863 | 7.505394176 | 3.633649407 | 0.001202774 | 0.005113073 | -1.26367919 |
| GIMAP6 | -2.0565283 | 7.894518741 | -3.633539654 | 0.001203113 | 0.005113073 | -1.26394807 |
| MAP3K13 | -1.071935172 | 8.242850535 | -3.633468135 | 0.001203333 | 0.005113073 | -1.264123281 |
| TSTA3 | -1.778498081 | 5.63462121 | -3.633192367 | 0.001204185 | 0.005115001 | -1.264798863 |
| MDH1 | -1.54255605 | 11.58472188 | -3.63287861 | 0.001205154 | 0.005117428 | -1.265567492 |
| IGF2-AS | 2.772383522 | 11.12104902 | 3.631871803 | 0.00120827 | 0.005128965 | -1.268033785 |
| CYP3A43 | 2.207954561 | 9.515987584 | 3.631233067 | 0.00121025 | 0.005135677 | -1.269598331 |
| AMDHD2 | 2.026914658 | 10.76073309 | 3.630009574 | 0.001214053 | 0.005150115 | -1.272594968 |
| FARSB | 1.136417953 | 10.41204027 | 3.629670959 | 0.001215107 | 0.005152889 | -1.273424264 |
| ATP5G3 | -1.30062015 | 10.7844917 | -3.629484562 | 0.001215688 | 0.005153653 | -1.273880754 |
| DAB1 | 2.909664047 | 9.044489217 | 3.62928197 | 0.00121632 | 0.005154632 | -1.2743769 |
| HIC2 | 1.858564648 | 6.806387298 | 3.628604869 | 0.001218433 | 0.005161887 | -1.276035047 |
| PLRG1 | -1.789785486 | 6.463932532 | -3.628133831 | 0.001219906 | 0.005166423 | -1.277188508 |
| ARMCX1 | -1.729463125 | 8.433894873 | -3.627653969 | 0.001221407 | 0.00517108 | -1.278363532 |
| USP45 | 1.606297109 | 7.575706001 | 3.627370664 | 0.001222295 | 0.005173135 | -1.27905723 |
| ULK2 | -1.197460568 | 8.020068779 | -3.626940792 | 0.001223643 | 0.005177135 | -1.280109776 |
| COL23A1 | 2.8206566 | 8.14790942 | 3.626568308 | 0.001224812 | 0.005180377 | -1.281021777 |
| DENND4A | -1.601500536 | 5.445783733 | -3.626330528 | 0.001225558 | 0.005181832 | -1.281603948 |
| NDUFS2 | -1.60348264 | 6.760355027 | -3.625928465 | 0.001226822 | 0.00518547 | -1.282588317 |
| HCG18 | 1.045499833 | 8.037282983 | 3.625626552 | 0.001227772 | 0.00518778 | -1.283327466 |
| ANKDD1A | 3.521945593 | 13.02009557 | 3.623125622 | 0.001235668 | 0.005219428 | -1.289449545 |
| MS4A15 | -1.816526453 | 5.05375041 | -3.622993784 | 0.001236085 | 0.005219478 | -1.289772236 |
| TCF23 | 2.05970102 | 7.054230649 | 3.622172009 | 0.001238691 | 0.005228765 | -1.291783564 |
| CPNE7 | 1.290264724 | 6.116767776 | 3.621782685 | 0.001239928 | 0.005232268 | -1.2927364 |
| CHGA | 1.766132498 | 6.46292423 | 3.620218556 | 0.001244908 | 0.00525156 | -1.296564136 |
| SPEG | 1.607911072 | 9.604245793 | 3.619992931 | 0.001245628 | 0.005252874 | -1.297116245 |
| LOC100127891 | 1.63137159 | 8.407898163 | 3.619668412 | 0.001246664 | 0.005255521 | -1.297910325 |
| LOC100652774 | 2.343838451 | 6.569186149 | 3.619256091 | 0.001247982 | 0.005259353 | -1.298919222 |
| WDR1 | -1.241504466 | 9.08824038 | -3.618518005 | 0.001250344 | 0.005267583 | -1.300725135 |
| IL18BP | 1.515059581 | 8.567163393 | 3.617896425 | 0.001252337 | 0.005274251 | -1.302245895 |
| C13orf33 | 2.339144563 | 9.368331268 | 3.617767245 | 0.001252752 | 0.00527427 | -1.302561936 |
| C1orf133 | -1.769808918 | 7.284191052 | -3.617471298 | 0.001253702 | 0.005276543 | -1.303285964 |
| TLR3 | -1.804202092 | 6.23144794 | -3.6159724 | 0.001258526 | 0.005295112 | -1.306952696 |
| HPS5 | -1.692671264 | 6.512380315 | -3.615592284 | 0.001259752 | 0.005298538 | -1.307882492 |
| ADIPOR2 | -2.629673922 | 8.158910375 | -3.613114354 | 0.001267774 | 0.005330534 | -1.313942956 |
| S1PR1 | -1.744538905 | 8.77468497 | -3.612751367 | 0.001268953 | 0.005333749 | -1.31483063 |
| RIPK2 | 1.95456287 | 10.00764526 | 3.612473239 | 0.001269857 | 0.005335806 | -1.315510764 |
| ARHGAP23 | -1.751283958 | 10.89135902 | -3.611962882 | 0.001271518 | 0.005341041 | -1.316758747 |
| OCLN | -1.703107021 | 6.737635915 | -3.610365071 | 0.001276732 | 0.005359441 | -1.320665541 |
| RBM12 | -1.353947928 | 6.776625726 | -3.609239543 | 0.001280417 | 0.005372782 | -1.323417227 |
| CHCHD2 | 1.104198952 | 12.44279167 | 3.609090937 | 0.001280905 | 0.005372782 | -1.323780518 |
| HIST1H2AK | 1.353713652 | 10.71505374 | 3.609012077 | 0.001281163 | 0.005372782 | -1.3239733 |
| C9orf103 | -1.550248772 | 7.775280409 | -3.608420725 | 0.001283105 | 0.005379171 | -1.325418897 |
| ACAD8 | -1.50204302 | 6.067857133 | -3.608265878 | 0.001283614 | 0.00537955 | -1.325797417 |
| WRAP53 | 1.717446437 | 6.967644804 | 3.607465911 | 0.001286246 | 0.00538707 | -1.327752838 |
| DHRS12 | -1.334400448 | 7.335175475 | -3.606777492 | 0.001288515 | 0.005394818 | -1.329435483 |
| DOK7 | -2.529159536 | 6.998587618 | -3.606639703 | 0.00128897 | 0.005394965 | -1.329772257 |
| SUGP1 | -2.088593809 | 5.574687683 | -3.606343618 | 0.001289947 | 0.0053973 | -1.330495913 |
| TRPC4AP | -1.091832036 | 5.791925275 | -3.605983481 | 0.001291137 | 0.005400234 | -1.331376089 |
| C19orf52 | -1.492149841 | 6.046914252 | -3.605877338 | 0.001291488 | 0.005400234 | -1.331635499 |
| TBC1D2B | -1.423534794 | 8.939001911 | -3.605209702 | 0.001293697 | 0.005407714 | -1.333267116 |
| SRPRB | -1.809971647 | 7.649450178 | -3.605035088 | 0.001294276 | 0.005408375 | -1.333693836 |
| TFEC | 2.177779103 | 9.429521224 | 3.604524857 | 0.001295968 | 0.005413601 | -1.334940691 |
| CST3 | -1.363745841 | 9.288255806 | -3.604294401 | 0.001296732 | 0.005413601 | -1.335503839 |
| CUL5 | -1.463542286 | 7.763145502 | -3.604277291 | 0.001296789 | 0.005413601 | -1.335545651 |
| POT1 | -1.38827936 | 6.230065306 | -3.604073515 | 0.001297466 | 0.005414668 | -1.336043592 |
| WDR7 | -1.094875673 | 8.107439038 | -3.603548021 | 0.001299212 | 0.005420198 | -1.337327636 |
| LOC100130557 | 2.841084989 | 8.645780478 | 3.60270822 | 0.001302008 | 0.005430101 | -1.339379564 |
| C1orf198 | -1.566234232 | 13.33007537 | -3.599869578 | 0.001311503 | 0.005467925 | -1.346314221 |
| C1orf183 | 2.288280076 | 8.879132567 | 3.598719387 | 0.001315369 | 0.005480491 | -1.34912358 |
| SFTA1P | -1.381226651 | 8.517627453 | -3.59839527 | 0.00131646 | 0.005483262 | -1.349915187 |
| E2F1 | 1.592521518 | 6.680547975 | 3.597912029 | 0.001318089 | 0.00548827 | -1.351095391 |
| TBC1D20 | 1.15161066 | 7.289031426 | 3.597019012 | 0.001321105 | 0.005499046 | -1.353276241 |
| MPZ | 1.954487446 | 5.8107787 | 3.59530612 | 0.001326908 | 0.005521413 | -1.357458831 |
| ORMDL3 | 2.304065787 | 12.47465167 | 3.595120112 | 0.001327539 | 0.005522255 | -1.357912993 |
| RNF146 | -1.154406221 | 7.079748171 | -3.592829064 | 0.001335343 | 0.005552921 | -1.363506242 |
| HIST1H3F | 1.377445039 | 7.429950591 | 3.591713287 | 0.00133916 | 0.005566994 | -1.366229829 |
| MAGEF1 | -1.809899002 | 8.726323316 | -3.590830234 | 0.001342188 | 0.00557778 | -1.368385147 |
| SRSF7 | -1.341220362 | 9.234996184 | -3.590474809 | 0.001343409 | 0.005581049 | -1.369252602 |
| PCNT | 1.148696483 | 7.908476226 | 3.590269319 | 0.001344115 | 0.005581308 | -1.369754114 |
| CGNL1 | -1.899483311 | 9.320943836 | -3.590204206 | 0.001344339 | 0.005581308 | -1.369913024 |
| CDH10 | 3.139018815 | 8.943547944 | 3.589325226 | 0.001347365 | 0.005592065 | -1.372058108 |
| USMG5 | -1.243084306 | 12.88785478 | -3.588110794 | 0.001351556 | 0.005607651 | -1.375021559 |
| CHKB | -1.463837818 | 7.116332431 | -3.587128459 | 0.001354956 | 0.005619943 | -1.377418407 |
| ZBTB11 | 2.159713915 | 14.42969 | 3.586709505 | 0.001356408 | 0.005622863 | -1.378440571 |
| LGALSL | -1.176141165 | 8.338230844 | -3.586648405 | 0.00135662 | 0.005622863 | -1.37858964 |
| KHNYN | -1.309569211 | 7.604664469 | -3.586421206 | 0.001357409 | 0.005622863 | -1.37914394 |
| RBPMS | -1.215441639 | 7.62446614 | -3.585768364 | 0.001359677 | 0.005630292 | -1.380736624 |
| ACTG1 | 1.132896111 | 13.78996986 | 3.585653025 | 0.001360078 | 0.005630292 | -1.381017995 |
| SPPL2B | 1.42704927 | 8.57076789 | 3.58418829 | 0.001365181 | 0.0056496 | -1.384591005 |
| SNW1 | -1.689498316 | 8.77606513 | -3.58398069 | 0.001365906 | 0.005650781 | -1.385097376 |
| SLC39A2 | 1.93864184 | 6.5430734 | 3.58345508 | 0.001367742 | 0.00565656 | -1.386379387 |
| PARP6 | -1.378513746 | 8.241263377 | -3.582836967 | 0.001369906 | 0.005661634 | -1.38788694 |
| RAD21 | -2.245913757 | 9.796171095 | -3.582727239 | 0.00137029 | 0.005661634 | -1.388154554 |
| SEMA6C | 1.75785084 | 9.984896349 | 3.582106126 | 0.001372467 | 0.00566881 | -1.389669324 |
| FAHD1 | -1.257497445 | 5.776489601 | -3.581817886 | 0.001373479 | 0.005669911 | -1.390372254 |
| LOC286272 | 2.895693876 | 8.536454254 | 3.581779037 | 0.001373616 | 0.005669911 | -1.390466996 |
| HMBS | 1.187998906 | 9.505187804 | 3.579674872 | 0.001381024 | 0.005697925 | -1.395597844 |
| INO80 | -1.29142951 | 8.271681989 | -3.579599915 | 0.001381288 | 0.005697925 | -1.395780604 |
| FDXR | 1.343540836 | 10.1777397 | 3.579440701 | 0.00138185 | 0.005698416 | -1.396168792 |
| CDH3 | 2.180794059 | 8.157788887 | 3.579157592 | 0.001382851 | 0.005700714 | -1.396859042 |
| ASCL2 | 1.597034829 | 8.769477225 | 3.578483435 | 0.001385236 | 0.005708715 | -1.398502638 |
| THYN1 | -1.447653343 | 7.157170213 | -3.577892693 | 0.001387329 | 0.005715509 | -1.399942784 |
| DKFZP434F142 | 1.532479058 | 8.515150198 | 3.57637434 | 0.001392722 | 0.005735485 | -1.40364396 |
| RAB5B | -1.474255042 | 5.98497562 | -3.576205304 | 0.001393324 | 0.005735485 | -1.404055975 |
| GHRHR | 1.886008233 | 7.339956428 | 3.57615166 | 0.001393515 | 0.005735485 | -1.404186726 |
| PLXNA2 | -1.355022432 | 6.763706659 | -3.575699248 | 0.001395127 | 0.005738846 | -1.405289417 |
| EIF3F | -1.345527754 | 8.558988296 | -3.573525611 | 0.001402897 | 0.005767515 | -1.410586706 |
| SLC25A19 | 1.264002872 | 7.295614299 | 3.573364956 | 0.001403473 | 0.005767515 | -1.41097819 |
| C11orf54 | -1.60017744 | 8.163228098 | -3.573346514 | 0.001403539 | 0.005767515 | -1.411023128 |
| OR5V1 | 1.636697036 | 5.402867358 | 3.572819168 | 0.001405432 | 0.005773447 | -1.412308123 |
| ID2 | -1.251500395 | 10.43502049 | -3.572557826 | 0.00140637 | 0.005775458 | -1.412944919 |
| SMARCAL1 | -1.653775398 | 6.088820849 | -3.571485342 | 0.001410229 | 0.005789456 | -1.415558006 |
| LOC541471 | 1.131481001 | 9.388437698 | 3.570877763 | 0.00141242 | 0.005795386 | -1.417038248 |
| LOC100134167 | 1.476998203 | 8.257410235 | 3.570834814 | 0.001412575 | 0.005795386 | -1.417142881 |
| CMIP | 1.226657889 | 9.50439854 | 3.570628703 | 0.001413318 | 0.005796589 | -1.417645007 |
| MORN2 | -1.89746014 | 6.434215949 | -3.570060159 | 0.001415373 | 0.005803163 | -1.419030041 |
| USHBP1 | -2.162080915 | 4.681961159 | -3.569119358 | 0.001418778 | 0.005815272 | -1.421321772 |
| VAV1 | -1.631262186 | 6.014635468 | -3.567028511 | 0.001426375 | 0.005840807 | -1.426414232 |
| FOXJ1 | -2.149283598 | 6.693307358 | -3.566920381 | 0.001426769 | 0.005840807 | -1.426677567 |
| TOLLIP | 2.148190184 | 7.762866336 | 3.566912101 | 0.001426799 | 0.005840807 | -1.42669773 |
| C8orf73 | 1.844055986 | 11.80468683 | 3.566863563 | 0.001426976 | 0.005840807 | -1.426815937 |
| BBS4 | -1.415453237 | 6.707865597 | -3.566755912 | 0.001427368 | 0.005840807 | -1.427078099 |
| TMEM125 | -2.279408616 | 12.57553631 | -3.566625841 | 0.001427843 | 0.005840807 | -1.427394859 |
| LIMA1 | -2.308116685 | 8.611998849 | -3.566531486 | 0.001428187 | 0.005840807 | -1.42762464 |
| FAM70B | 1.779992301 | 7.224137268 | 3.56607092 | 0.001429867 | 0.005844787 | -1.428746209 |
| TMEM214 | 1.832909386 | 7.286219728 | 3.565840396 | 0.001430709 | 0.005844787 | -1.429307563 |
| PSMG2 | -1.012405488 | 7.714339564 | -3.565767015 | 0.001430978 | 0.005844787 | -1.42948625 |
| TNFRSF6B | 1.768795317 | 7.511919278 | 3.565062046 | 0.001433556 | 0.005852668 | -1.431202847 |
| ZMYM1 | -1.222366643 | 5.059002638 | -3.564990718 | 0.001433817 | 0.005852668 | -1.431376525 |
| CEP72 | -1.5924217 | 5.275525575 | -3.564257092 | 0.001436506 | 0.005861782 | -1.433162771 |
| GAR1 | -1.77988718 | 7.744356191 | -3.562772681 | 0.001441961 | 0.005880311 | -1.436776671 |
| PRDM4 | 1.727778208 | 8.869270898 | 3.562451805 | 0.001443143 | 0.005883265 | -1.437557801 |
| PPTC7 | -1.726791616 | 7.846434824 | -3.562181753 | 0.001444138 | 0.005884914 | -1.438215188 |
| FAM176B | 1.440330964 | 7.865436589 | 3.561938288 | 0.001445036 | 0.005885387 | -1.43880784 |
| NPEPPS | 1.365534963 | 10.94053198 | 3.560921181 | 0.001448793 | 0.005898333 | -1.441283576 |
| AMY1C | -2.482310814 | 8.606054063 | -3.560829601 | 0.001449132 | 0.005898333 | -1.44150648 |
| GUCY1B3 | -1.376852531 | 6.276854465 | -3.560464889 | 0.001450482 | 0.00590196 | -1.442394159 |
| ZNF706 | -1.187989527 | 10.67237821 | -3.560055731 | 0.001451998 | 0.005906259 | -1.443389982 |
| DCUN1D2 | -1.204379192 | 4.515824934 | -3.558839674 | 0.001456512 | 0.005920877 | -1.446349436 |
| UPK2 | 2.003201641 | 8.16777427 | 3.557275993 | 0.001462337 | 0.005942677 | -1.450154392 |
| SNHG11 | -1.185603674 | 5.204083164 | -3.556691969 | 0.001464518 | 0.005949662 | -1.451575374 |
| TMEM37 | -1.458048985 | 5.977677317 | -3.556329266 | 0.001465874 | 0.005951684 | -1.452457822 |
| ZC3H14 | 1.045737999 | 8.305432346 | 3.556311386 | 0.001465941 | 0.005951684 | -1.452501324 |
| TSPAN10 | 3.134705288 | 10.47069445 | 3.554413678 | 0.001473058 | 0.005978691 | -1.457117915 |
| FAM96A | -1.61062566 | 8.606004648 | -3.553438351 | 0.001476729 | 0.005991698 | -1.45949029 |
| ESYT1 | -1.557343877 | 6.788732854 | -3.551844825 | 0.001482745 | 0.006012316 | -1.463365903 |
| SUSD2 | -2.605672968 | 11.7967248 | -3.551698257 | 0.0014833 | 0.006012669 | -1.463722343 |
| DKK3 | -1.854648495 | 9.815049059 | -3.551414801 | 0.001484373 | 0.006015124 | -1.464411666 |
| KISS1 | 2.524099702 | 8.236438629 | 3.550991213 | 0.001485978 | 0.006019731 | -1.465441734 |
| RPS4X | -1.777192683 | 13.73353076 | -3.550382999 | 0.001488285 | 0.006025183 | -1.466920699 |
| CHAD | 2.078827657 | 7.084936231 | 3.550295554 | 0.001488617 | 0.006025183 | -1.467133328 |
| GPR84 | 1.980403593 | 6.730672717 | 3.550251289 | 0.001488785 | 0.006025183 | -1.46724096 |
| ZNF829 | -1.380980151 | 5.591404199 | -3.550142864 | 0.001489197 | 0.006025183 | -1.467504599 |
| CACNB2 | 1.460828807 | 6.650154507 | 3.549982858 | 0.001489805 | 0.006025747 | -1.467893656 |
| C6orf226 | -1.218080362 | 5.328838847 | -3.548545709 | 0.001495277 | 0.006045977 | -1.471387836 |
| FAM46A | -1.977902927 | 9.138390771 | -3.548390358 | 0.00149587 | 0.006046473 | -1.471765516 |
| ADAP2 | 1.823003366 | 10.45257064 | 3.54754894 | 0.001499084 | 0.006056495 | -1.473811028 |
| C3orf38 | -1.029192042 | 6.579750647 | -3.547494744 | 0.001499291 | 0.006056495 | -1.473942775 |
| KIR3DL1 | 1.618426428 | 6.272145674 | 3.546238513 | 0.001504103 | 0.006074024 | -1.476996391 |
| SRPX2 | 1.823320764 | 9.06707833 | 3.54513259 | 0.001508351 | 0.006089268 | -1.479684343 |
| WNT6 | 1.811778165 | 8.984107432 | 3.544531196 | 0.001510666 | 0.006096701 | -1.481145917 |
| MEST | -1.66071304 | 5.972967653 | -3.544040475 | 0.001512558 | 0.00610242 | -1.482338459 |
| SPEF2 | -1.594686366 | 5.204881394 | -3.543242107 | 0.00151564 | 0.006112939 | -1.484278521 |
| SPHK1 | 1.571093854 | 7.902466683 | 3.542906603 | 0.001516937 | 0.006116253 | -1.485093765 |
| CUL4A | -1.082903532 | 8.650132353 | -3.541947371 | 0.001520652 | 0.006129309 | -1.487424463 |
| FLJ25694 | 2.798826777 | 7.736416423 | 3.541614407 | 0.001521943 | 0.006131753 | -1.488233435 |
| FNIP2 | -2.301203365 | 6.74065244 | -3.541438577 | 0.001522626 | 0.006131753 | -1.488660622 |
| TRAF6 | -1.33939986 | 5.995608802 | -3.541422469 | 0.001522688 | 0.006131753 | -1.488699757 |
| ABT1 | -1.738464122 | 5.207269925 | -3.541024275 | 0.001524235 | 0.00613606 | -1.48966716 |
| KCNQ4 | 2.038221774 | 6.195153559 | 3.539157908 | 0.001531505 | 0.006163396 | -1.494200971 |
| SMCR7L | -1.052278793 | 8.468688538 | -3.538384132 | 0.001534529 | 0.006173634 | -1.496080404 |
| LCA5 | -1.077505925 | 5.846370925 | -3.536519152 | 0.001541841 | 0.006201112 | -1.500609705 |
| COPS6 | -1.388840961 | 9.22831509 | -3.536059035 | 0.00154365 | 0.006206448 | -1.501727023 |
| BRD7 | -1.015020988 | 9.677066665 | -3.535676015 | 0.001545158 | 0.006209934 | -1.502657087 |
| ASH1L | 1.292116586 | 10.05510927 | 3.535593454 | 0.001545483 | 0.006209934 | -1.502857561 |
| TBC1D3P5 | 1.815225101 | 6.22246467 | 3.53499091 | 0.001547858 | 0.006216477 | -1.504320602 |
| OR7E47P | -1.715952168 | 7.802891088 | -3.534935185 | 0.001548078 | 0.006216477 | -1.504455902 |
| SELENBP1 | -1.878569138 | 10.4229198 | -3.534691156 | 0.001549041 | 0.006217257 | -1.505048403 |
| NET1 | -1.036710778 | 9.033594008 | -3.534608012 | 0.001549369 | 0.006217257 | -1.505250274 |
| DDN | 1.724021885 | 6.095008943 | 3.534518617 | 0.001549722 | 0.006217257 | -1.505467318 |
| RP9 | -1.026573586 | 6.875063868 | -3.533967002 | 0.001551902 | 0.006224061 | -1.506806564 |
| SLC38A1 | -2.267173878 | 7.940212667 | -3.532896653 | 0.001556141 | 0.006239115 | -1.50940502 |
| BMP8B | 1.535290942 | 11.78751293 | 3.530781955 | 0.001564548 | 0.006268915 | -1.514538032 |
| EID1 | -1.19526916 | 12.00021814 | -3.530129063 | 0.001567153 | 0.006277396 | -1.516122588 |
| ATXN7L2 | 1.211871259 | 7.691684375 | 3.529903723 | 0.001568053 | 0.006279045 | -1.51666946 |
| EGFL6 | 1.949055206 | 9.163527077 | 3.528860687 | 0.001572225 | 0.006293793 | -1.519200628 |
| PKI55 | -1.900136446 | 5.919190978 | -3.528662824 | 0.001573018 | 0.006295007 | -1.519680758 |
| LAMA3 | -2.002573216 | 5.932203565 | -3.528504012 | 0.001573654 | 0.006295595 | -1.520066123 |
| DUSP1 | -2.444760161 | 11.46800531 | -3.527683684 | 0.001576946 | 0.006306803 | -1.522056591 |
| SPCS2 | -2.084253544 | 8.700722043 | -3.527561296 | 0.001577438 | 0.006306808 | -1.522353542 |
| SFI1 | 1.655357745 | 9.106746881 | 3.526824776 | 0.0015804 | 0.006316688 | -1.524140501 |
| ROPN1L | -1.850669984 | 6.653735627 | -3.526368098 | 0.001582239 | 0.006322075 | -1.525248438 |
| FAM40A | -1.484434766 | 6.065909407 | -3.525424335 | 0.001586047 | 0.006333863 | -1.527537925 |
| ESRRG | 1.961688468 | 6.665962317 | 3.52539285 | 0.001586174 | 0.006333863 | -1.527614303 |
| GFM1 | -1.902959841 | 6.381322043 | -3.524437473 | 0.001590038 | 0.006345445 | -1.529931747 |
| C21orf67 | 1.65268352 | 6.778069495 | 3.524422548 | 0.001590099 | 0.006345445 | -1.529967947 |
| ZNF362 | 1.772391229 | 13.49800803 | 3.524309904 | 0.001590555 | 0.006345445 | -1.530241172 |
| ABI3BP | -2.049976062 | 7.082169259 | -3.523808783 | 0.001592586 | 0.006351578 | -1.531456634 |
| VPS16 | -1.404475731 | 6.427828579 | -3.523142677 | 0.00159529 | 0.006360389 | -1.533072172 |
| TSHB | 2.367369585 | 7.75576738 | 3.522857379 | 0.001596449 | 0.006361068 | -1.533764086 |
| SGSM3 | 1.268648493 | 9.695793214 | 3.522651512 | 0.001597286 | 0.006362433 | -1.534263351 |
| CHUK | -1.371934791 | 4.409566006 | -3.522222846 | 0.001599031 | 0.006367409 | -1.535302909 |
| PLA2G6 | 2.152850668 | 8.875037749 | 3.521375664 | 0.001602484 | 0.006379184 | -1.537357287 |
| DDX56 | 1.408728335 | 7.540679063 | 3.520922437 | 0.001604334 | 0.006384574 | -1.538456271 |
| C6orf106 | 1.252963135 | 12.08798004 | 3.519959534 | 0.001608272 | 0.006396931 | -1.54079096 |
| SHISA7 | 2.019979437 | 7.839395991 | 3.519820073 | 0.001608843 | 0.006396931 | -1.541129083 |
| MIIP | -1.322013579 | 4.774095131 | -3.51979855 | 0.001608931 | 0.006396931 | -1.541181265 |
| IL10RB | -1.60931907 | 6.114820367 | -3.519046406 | 0.001612015 | 0.006405749 | -1.543004759 |
| FPGT | -1.245593748 | 5.44097842 | -3.518903842 | 0.0016126 | 0.006405749 | -1.543350376 |
| RCN3 | 1.159857529 | 6.517270674 | 3.518893344 | 0.001612643 | 0.006405749 | -1.543375826 |
| LIPT2 | -1.743296136 | 4.606583856 | -3.517507003 | 0.001618344 | 0.006426411 | -1.546736464 |
| KLHL8 | -1.609458221 | 6.17816199 | -3.516210378 | 0.001623694 | 0.006445666 | -1.549879208 |
| YPEL3 | -1.392445306 | 7.693199289 | -3.515452197 | 0.001626831 | 0.006456124 | -1.551716697 |
| FAM100A | 1.247560212 | 8.049782224 | 3.515172204 | 0.00162799 | 0.006457436 | -1.552395237 |
| SLC4A11 | 1.237385927 | 6.581851661 | 3.515129924 | 0.001628166 | 0.006457436 | -1.5524977 |
| NUDCD2 | -1.152406331 | 8.697926916 | -3.514705204 | 0.001629926 | 0.006462427 | -1.553526936 |
| LLGL1 | 2.911299281 | 10.10910442 | 3.512702891 | 0.001638253 | 0.006493439 | -1.558378628 |
| KCNJ8 | -1.31375073 | 7.806870804 | -3.512148832 | 0.001640564 | 0.006500597 | -1.559720969 |
| KAT8 | -1.315621255 | 5.980753261 | -3.510973964 | 0.001645476 | 0.006518051 | -1.562567129 |
| CNTN2 | 2.275727358 | 6.950066573 | 3.51039113 | 0.001647918 | 0.006525714 | -1.563978944 |
| HIST1H2AE | 1.524174971 | 8.829983923 | 3.509825946 | 0.001650289 | 0.006533093 | -1.565347927 |
| LOC641746 | -1.445921033 | 6.722037599 | -3.508044775 | 0.001657784 | 0.006560474 | -1.569661766 |
| RBM26 | -1.409062916 | 6.795804956 | -3.507939982 | 0.001658226 | 0.006560474 | -1.56991554 |
| NINJ1 | 1.791948561 | 10.57257775 | 3.507499433 | 0.001660085 | 0.006565811 | -1.570982384 |
| CROCC | 1.678712634 | 5.891416335 | 3.506949942 | 0.001662407 | 0.006572686 | -1.572312979 |
| MRS2 | -1.725879738 | 5.972864684 | -3.506846294 | 0.001662846 | 0.006572686 | -1.572563956 |
| C19orf38 | 1.563916838 | 7.303296836 | 3.5064093 | 0.001664695 | 0.006577974 | -1.573622076 |
| HADH | -1.524919933 | 9.767264087 | -3.503488538 | 0.001677107 | 0.006624984 | -1.580693128 |
| TEF | 2.647273081 | 8.464861296 | 3.502814679 | 0.001679983 | 0.006634309 | -1.582324226 |
| STX3 | -1.739297391 | 4.515049847 | -3.502646063 | 0.001680704 | 0.006635117 | -1.582732348 |
| SEPT3 | 1.927498346 | 6.91178225 | 3.500721136 | 0.00168895 | 0.006663878 | -1.58739102 |
| LOC100130456 | 1.995740133 | 6.598282385 | 3.500703649 | 0.001689025 | 0.006663878 | -1.587433338 |
| RSU1 | -1.387425279 | 8.029477408 | -3.500507742 | 0.001689867 | 0.006665153 | -1.587907417 |
| PASK | 2.154825233 | 7.451970252 | 3.500220045 | 0.001691103 | 0.0066667 | -1.588603605 |
| CALM1 | -1.226536172 | 7.74891224 | -3.500175333 | 0.001691295 | 0.0066667 | -1.5887118 |
| SPAG5-AS1 | 1.270978669 | 6.229334162 | 3.49903723 | 0.001696196 | 0.006683969 | -1.591465647 |
| FIBP | -1.122173031 | 6.874895544 | -3.498351892 | 0.001699154 | 0.006693574 | -1.5931238 |
| MT1G | 1.998842278 | 12.84633221 | 3.498032714 | 0.001700533 | 0.006696956 | -1.593895999 |
| ILF3 | -1.292660275 | 7.353865542 | -3.497325979 | 0.001703591 | 0.006706944 | -1.595605748 |
| SLC25A39 | -1.645882639 | 7.137453303 | -3.497077008 | 0.001704669 | 0.006709136 | -1.596208035 |
| NPRL2 | -1.350151204 | 5.900013318 | -3.496949995 | 0.00170522 | 0.00670925 | -1.596515285 |
| ASB16 | 2.583867866 | 7.356974465 | 3.496661392 | 0.001706471 | 0.00671212 | -1.597213419 |
| PFN2 | -1.646622102 | 5.377007208 | -3.494199328 | 0.001717183 | 0.006750877 | -1.603168361 |
| TOMM5 | -1.10120086 | 9.627418457 | -3.494073935 | 0.00171773 | 0.006750877 | -1.603471606 |
| TRIB3 | 1.744042318 | 9.146709187 | 3.494035219 | 0.001717899 | 0.006750877 | -1.603565235 |
| BTBD3 | -1.889214262 | 5.471219628 | -3.493877783 | 0.001718587 | 0.006751516 | -1.603945967 |
| PNMA3 | 1.348850867 | 8.352813518 | 3.493188766 | 0.001721599 | 0.006761283 | -1.605612164 |
| FBXO21 | -1.641220596 | 8.353848524 | -3.492076871 | 0.00172647 | 0.006778343 | -1.608300728 |
| ARHGAP24 | -1.212353108 | 5.075259506 | -3.491624992 | 0.001728453 | 0.00678406 | -1.609393289 |
| ZFHX2 | 1.418116394 | 7.239026559 | 3.491242024 | 0.001730136 | 0.006788593 | -1.610319196 |
| LOC100506655 | 1.815914355 | 5.956598161 | 3.490512629 | 0.001733345 | 0.006798067 | -1.612082566 |
| GKAP1 | -1.529118475 | 6.285450089 | -3.490453057 | 0.001733607 | 0.006798067 | -1.61222658 |
| ZZEF1 | 1.917604592 | 9.058605077 | 3.490035422 | 0.001735448 | 0.00680321 | -1.613236183 |
| UBAP1 | -1.13809015 | 7.546161677 | -3.4897519 | 0.001736698 | 0.006806038 | -1.613921553 |
| UBE2T | 1.562664081 | 8.65006187 | 3.48885005 | 0.001740682 | 0.006819571 | -1.616101502 |
| LCE2C | 1.353807379 | 5.652624833 | 3.487832477 | 0.001745187 | 0.00683514 | -1.61856094 |
| PSMB9 | -1.081539808 | 8.602756335 | -3.487463919 | 0.001746822 | 0.00683946 | -1.619451671 |
| SPACA3 | 1.689606145 | 6.629309367 | 3.487148555 | 0.001748222 | 0.006842858 | -1.620213815 |
| WDR45L | -1.89610395 | 10.26539635 | -3.486786277 | 0.001749831 | 0.006846525 | -1.621089309 |
| CLTC | -1.447429276 | 11.26062387 | -3.48669807 | 0.001750223 | 0.006846525 | -1.621302468 |
| KIAA0913 | 1.625211996 | 6.15458589 | 3.48646464 | 0.001751261 | 0.006846641 | -1.621866559 |
| TMEM50B | -1.395829748 | 8.217865009 | -3.486451974 | 0.001751318 | 0.006846641 | -1.621897169 |
| BIRC7 | 1.723620597 | 5.126525431 | 3.485386151 | 0.001756065 | 0.006863114 | -1.624472598 |
| ITPA | -1.155898359 | 7.940787072 | -3.484882069 | 0.001758315 | 0.006869819 | -1.625690555 |
| RPL27A | 1.078094116 | 15.32014554 | 3.484707144 | 0.001759096 | 0.006870785 | -1.626113191 |
| CADM3 | 1.925652353 | 7.3450532 | 3.484290257 | 0.001760959 | 0.006873099 | -1.627120403 |
| TPX2 | 1.362788931 | 6.352210293 | 3.484204304 | 0.001761344 | 0.006873099 | -1.627328063 |
| ATP2A3 | 1.270653364 | 5.894263668 | 3.484096501 | 0.001761826 | 0.006873099 | -1.62758851 |
| CHRNB2 | 1.746111329 | 7.243442303 | 3.483243921 | 0.001765645 | 0.006885908 | -1.6296482 |
| PAFAH1B1 | -1.328864367 | 9.131944306 | -3.482412842 | 0.001769375 | 0.006898363 | -1.631655774 |
| KLF3 | -1.674295685 | 8.010045201 | -3.481748151 | 0.001772364 | 0.006907922 | -1.633261298 |
| SLMO1 | 1.353659629 | 8.045245197 | 3.481539054 | 0.001773305 | 0.006909497 | -1.633766338 |
| C11orf42 | 1.966049403 | 8.089120598 | 3.480791247 | 0.001776675 | 0.006920531 | -1.635572456 |
| MYH14 | 2.375203791 | 10.14754457 | 3.480035629 | 0.001780087 | 0.006931721 | -1.6373973 |
| LAT2 | 1.257766439 | 10.57942858 | 3.478930515 | 0.001785088 | 0.006949092 | -1.640065939 |
| XRCC5 | -1.280746685 | 6.366063505 | -3.477735426 | 0.001790512 | 0.006963979 | -1.642951514 |
| HOXB7 | 2.328101645 | 9.976986731 | 3.477616479 | 0.001791052 | 0.006963979 | -1.643238697 |
| SIRT6 | -1.25753374 | 6.140587279 | -3.477610845 | 0.001791078 | 0.006963979 | -1.6432523 |
| BCAT2 | -1.275375908 | 7.974225619 | -3.477128891 | 0.00179327 | 0.006970397 | -1.644415877 |
| R3HDM1 | -1.565256524 | 5.653001768 | -3.476365101 | 0.00179675 | 0.006981813 | -1.646259771 |
| SVEP1 | 1.762104651 | 9.280691564 | 3.475192547 | 0.001802105 | 0.006997947 | -1.649090201 |
| GUF1 | 1.084336204 | 8.181209844 | 3.475098693 | 0.001802535 | 0.006997947 | -1.649316742 |
| GKN2 | -2.048794767 | 8.622917638 | -3.474762527 | 0.001804073 | 0.007001807 | -1.650128144 |
| NUDT6 | -1.511280554 | 4.887409333 | -3.474097513 | 0.00180712 | 0.007011518 | -1.651733202 |
| CLCNKB | 2.331695713 | 6.374733816 | 3.473945748 | 0.001807816 | 0.007011945 | -1.652099482 |
| PRKAR2B | -1.607591738 | 6.901173241 | -3.473816428 | 0.00180841 | 0.007011945 | -1.652411588 |
| UCHL1 | 1.854251772 | 10.53219089 | 3.473717048 | 0.001808866 | 0.007011945 | -1.652651433 |
| LPPR2 | 1.467072258 | 6.917317891 | 3.472365596 | 0.00181508 | 0.007033913 | -1.655912795 |
| KIAA1755 | 2.336088281 | 6.70490447 | 3.471380001 | 0.001819625 | 0.007049401 | -1.658290979 |
| LOC100132832 | 1.033189346 | 6.697244728 | 3.471124719 | 0.001820803 | 0.007051844 | -1.658906923 |
| DHX9 | -1.060122471 | 8.688625429 | -3.470116773 | 0.001825466 | 0.007065824 | -1.661338726 |
| DCAF5 | -1.124394659 | 8.551583526 | -3.470106829 | 0.001825512 | 0.007065824 | -1.661362717 |
| IMP3 | -1.592210997 | 8.66173003 | -3.469649965 | 0.001827629 | 0.007068618 | -1.662464878 |
| C2orf66 | 2.026952791 | 6.762948036 | 3.469599884 | 0.001827861 | 0.007068618 | -1.662585692 |
| POU2AF1 | 2.165550647 | 10.69236534 | 3.469595287 | 0.001827882 | 0.007068618 | -1.662596783 |
| SMAD5 | -1.186817616 | 7.427499449 | -3.469366262 | 0.001828945 | 0.0070706 | -1.663149271 |
| LAMC1 | -1.779508755 | 8.687567368 | -3.467506353 | 0.001837594 | 0.007101903 | -1.667635549 |
| PLAC8 | -2.105466197 | 7.611328963 | -3.466977387 | 0.001840061 | 0.007109302 | -1.668911308 |
| SPARCL1 | -1.894306154 | 12.53000473 | -3.465818372 | 0.001845478 | 0.00712595 | -1.671706378 |
| STRBP | -1.150457507 | 7.236756306 | -3.465597826 | 0.001846511 | 0.007126695 | -1.672238207 |
| RECQL4 | 1.389953604 | 6.305582846 | 3.465540517 | 0.001846779 | 0.007126695 | -1.672376402 |
| KIAA1147 | -1.877069872 | 6.470370011 | -3.46512444 | 0.001848729 | 0.00712994 | -1.673379701 |
| TMEM126A | -1.245570376 | 8.843915642 | -3.464450482 | 0.001851891 | 0.007139996 | -1.675004743 |
| GPX8 | 1.479748894 | 9.235178199 | 3.464123131 | 0.001853429 | 0.007141644 | -1.675794008 |
| CCDC108 | 1.839640888 | 7.665334169 | 3.463941706 | 0.001854282 | 0.007142791 | -1.676231426 |
| LOC100507246 | 1.449453224 | 7.902557663 | 3.462575801 | 0.001860716 | 0.007161974 | -1.679524367 |
| CREB3L1 | 2.401937324 | 10.51902298 | 3.462464654 | 0.00186124 | 0.007161974 | -1.679792302 |
| WDR19 | -1.092018255 | 6.292056842 | -3.462347713 | 0.001861792 | 0.007161974 | -1.680074199 |
| ZNF770 | -1.278376541 | 4.816907968 | -3.462293868 | 0.001862046 | 0.007161974 | -1.680203997 |
| OLFM1 | -1.374242596 | 6.475687048 | -3.461840677 | 0.001864187 | 0.007168065 | -1.681296422 |
| FGF13 | -1.441958303 | 5.950857938 | -3.461675711 | 0.001864967 | 0.007168279 | -1.681694062 |
| CLPS | 2.866512971 | 7.881647452 | 3.461593157 | 0.001865358 | 0.007168279 | -1.681893052 |
| CD6 | 1.931101848 | 7.228924257 | 3.460958128 | 0.001868363 | 0.007177685 | -1.683423675 |
| IRF4 | 1.323510592 | 6.070144213 | 3.460728942 | 0.001869449 | 0.007179712 | -1.683976062 |
| SLC16A3 | 2.100455596 | 12.85764154 | 3.460132445 | 0.001872278 | 0.007186555 | -1.685413686 |
| SRM | -1.12460895 | 5.574764607 | -3.460117683 | 0.001872348 | 0.007186555 | -1.685449263 |
| UTRN | -1.316124611 | 9.734595088 | -3.459764801 | 0.001874024 | 0.007190841 | -1.686299704 |
| DNAJB3 | 1.627962396 | 5.328309828 | 3.458296604 | 0.001881012 | 0.007215501 | -1.689837713 |
| PSMC4 | -1.331670472 | 6.754172718 | -3.457620315 | 0.001884239 | 0.007225726 | -1.691467226 |
| GGT1 | 2.179055196 | 10.87380525 | 3.456848085 | 0.001887931 | 0.007237724 | -1.693327772 |
| NDUFA5 | -1.157133811 | 8.545207801 | -3.456244058 | 0.001890823 | 0.007246653 | -1.694782959 |
| MED19 | 2.527367986 | 10.74337316 | 3.455633542 | 0.001893751 | 0.007253772 | -1.696253686 |
| CYTH4 | 1.46930097 | 6.984830523 | 3.455255916 | 0.001895564 | 0.007258335 | -1.697163339 |
| TNNC2 | -1.276213013 | 5.676458637 | -3.453100173 | 0.001905948 | 0.007295922 | -1.702355557 |
| SEC16A | -1.036876522 | 7.057075194 | -3.452822969 | 0.001907287 | 0.007298876 | -1.703023132 |
| WDR92 | -1.387544464 | 6.012789827 | -3.452442156 | 0.001909128 | 0.007303749 | -1.703940193 |
| ARMC3 | -2.044657569 | 6.22502086 | -3.452085065 | 0.001910856 | 0.007308186 | -1.704800094 |
| B3GNT6 | 2.001314751 | 6.523512807 | 3.451916853 | 0.001911671 | 0.007309128 | -1.70520515 |
| PTAFR | 1.713517633 | 8.62897399 | 3.451613654 | 0.00191314 | 0.007312571 | -1.705935237 |
| NDUFS8 | -1.230418749 | 8.767042596 | -3.451372858 | 0.001914307 | 0.00731486 | -1.706515044 |
| FPGS | 2.147157353 | 14.13605935 | 3.450608041 | 0.00191802 | 0.007324695 | -1.708356534 |
| RSPH1 | -2.641251894 | 9.541047433 | -3.450288939 | 0.001919571 | 0.007325506 | -1.70912481 |
| HLA-C | 1.655641753 | 14.4722981 | 3.4502183 | 0.001919915 | 0.007325506 | -1.709294877 |
| LOC727869 | 1.130765249 | 6.004345825 | 3.450212861 | 0.001919941 | 0.007325506 | -1.709307972 |
| PNPLA2 | -1.223873254 | 6.189263352 | -3.449595203 | 0.001922947 | 0.007334801 | -1.710794973 |
| SLC26A9 | -2.311567197 | 6.009927774 | -3.447579663 | 0.00193279 | 0.007367974 | -1.715646678 |
| MMP1 | 3.46437248 | 9.205723223 | 3.446849542 | 0.001936367 | 0.007379424 | -1.717403935 |
| MKRN2 | -1.319931619 | 6.439420725 | -3.446030308 | 0.001940389 | 0.00739256 | -1.719375513 |
| GABRR3 | 2.17768299 | 6.923172332 | 3.444988925 | 0.001945513 | 0.007409887 | -1.72188147 |
| YWHAQ | -1.076316045 | 11.91811673 | -3.444356852 | 0.001948629 | 0.007419559 | -1.72340234 |
| GHSR | 1.517955991 | 6.783754135 | 3.4440101 | 0.001950341 | 0.007423879 | -1.72423664 |
| CCDC9 | 2.043467994 | 8.011723083 | 3.443565276 | 0.001952539 | 0.007430047 | -1.725306861 |
| CLEC14A | -1.817798894 | 10.55707562 | -3.443360516 | 0.001953552 | 0.007431701 | -1.725799484 |
| CD276 | 1.183988043 | 7.01620175 | 3.442715514 | 0.001956745 | 0.007441647 | -1.727351199 |
| OPHN1 | -1.43863048 | 7.979374303 | -3.442042826 | 0.00196008 | 0.007452128 | -1.728969406 |
| SNRNP48 | 1.312243957 | 10.63402781 | 3.441895666 | 0.00196081 | 0.007452702 | -1.729323398 |
| METTL22 | 2.744207494 | 12.26405053 | 3.441658946 | 0.001961986 | 0.007453729 | -1.729892809 |
| CALHM2 | -1.634920094 | 6.593728415 | -3.441607897 | 0.00196224 | 0.007453729 | -1.730015604 |
| FAM189A2 | -2.135777841 | 8.392893148 | -3.440666269 | 0.001966923 | 0.007469312 | -1.732280474 |
| CARD9 | 2.092472044 | 6.859939823 | 3.440499032 | 0.001967756 | 0.007470269 | -1.732682701 |
| ATP8A2 | -1.869886202 | 5.943421139 | -3.440131459 | 0.001969587 | 0.007475017 | -1.733566736 |
| HPDL | 1.465280737 | 6.252121883 | 3.43987531 | 0.001970865 | 0.007477659 | -1.734182771 |
| CDCA3 | 1.648695059 | 6.300844191 | 3.438450059 | 0.001977988 | 0.007502471 | -1.73761017 |
| RPUSD4 | -1.420971703 | 6.986666548 | -3.438107053 | 0.001979706 | 0.007506774 | -1.738434944 |
| TBC1D10C | 1.541245867 | 10.60804271 | 3.437612584 | 0.001982185 | 0.007510278 | -1.739623865 |
| MEX3A | 1.508260527 | 7.087595541 | 3.43757336 | 0.001982382 | 0.007510278 | -1.739718175 |
| MICB | 1.124132429 | 10.49753634 | 3.437396432 | 0.00198327 | 0.007511429 | -1.740143571 |
| KIAA0125 | 2.072250215 | 7.138263889 | 3.436770439 | 0.001986414 | 0.007519886 | -1.74164861 |
| RNF207 | 1.697010849 | 6.656118627 | 3.436670183 | 0.001986918 | 0.007519886 | -1.74188964 |
| C2CD2 | 1.725067752 | 8.467960203 | 3.436432121 | 0.001988116 | 0.007519886 | -1.742461967 |
| GPR97 | 1.885750609 | 6.865522806 | 3.436370518 | 0.001988426 | 0.007519886 | -1.742610065 |
| RPL11 | -1.687745655 | 13.01400129 | -3.435168348 | 0.001994484 | 0.007538364 | -1.74549997 |
| FILIP1L | -1.775245233 | 6.609976185 | -3.434928903 | 0.001995693 | 0.007540717 | -1.746075532 |
| JOSD2 | 1.524429685 | 8.702519148 | 3.434398274 | 0.001998374 | 0.007548631 | -1.747350966 |
| AHSP | 1.988413997 | 5.098240439 | 3.430464732 | 0.002018361 | 0.007619652 | -1.756803497 |
| LOC100652973 | -1.732908744 | 6.481937345 | -3.429930682 | 0.002021089 | 0.007626351 | -1.758086546 |
| INTS12 | -1.002464714 | 6.278857797 | -3.429816127 | 0.002021675 | 0.007626351 | -1.758361753 |
| LOC729173 | 1.738887772 | 6.432098072 | 3.429769356 | 0.002021914 | 0.007626351 | -1.758474116 |
| TMEM176A | 1.428014935 | 9.220372408 | 3.429516488 | 0.002023207 | 0.007628993 | -1.759081592 |
| WWP2 | -1.048042981 | 5.524554983 | -3.429342149 | 0.0020241 | 0.007630121 | -1.759500405 |
| CDKL1 | -1.254261676 | 6.613358542 | -3.427341703 | 0.002034367 | 0.007665161 | -1.764305502 |
| RNF7 | -1.103342215 | 10.35148751 | -3.427298851 | 0.002034587 | 0.007665161 | -1.764408422 |
| FAM64A | 1.232934672 | 7.033033575 | 3.426450115 | 0.002038959 | 0.007679381 | -1.766446778 |
| ALAD | -1.416197978 | 5.410109421 | -3.426289502 | 0.002039787 | 0.007680252 | -1.766832493 |
| LOC100505794 | -1.407718374 | 4.4682251 | -3.425720062 | 0.002042726 | 0.007689068 | -1.768199955 |
| CDHR3 | -2.08745626 | 5.383263045 | -3.424954758 | 0.002046683 | 0.00769951 | -1.770037636 |
| SORBS1 | -1.171660326 | 7.355836698 | -3.424951935 | 0.002046698 | 0.00769951 | -1.770044414 |
| SMG5 | -1.057173448 | 7.748996782 | -3.424172555 | 0.002050735 | 0.007711821 | -1.77191574 |
| HSPB11 | -1.25421733 | 8.380548487 | -3.421807392 | 0.002063034 | 0.007752753 | -1.777593646 |
| TPD52L1 | -1.819582088 | 7.331455654 | -3.421763699 | 0.002063262 | 0.007752753 | -1.777698523 |
| VPRBP | -1.032239849 | 4.812065063 | -3.421573908 | 0.002064252 | 0.007754208 | -1.778154078 |
| SLC45A4 | 3.268761657 | 9.329490683 | 3.420583581 | 0.002069426 | 0.007771373 | -1.780531011 |
| FANCI | 1.554620589 | 7.48608018 | 3.420261928 | 0.002071109 | 0.007775424 | -1.78130297 |
| OXER1 | 1.875753147 | 6.849347619 | 3.419712368 | 0.002073988 | 0.00778396 | -1.782621841 |
| KALRN | -1.235030593 | 6.79285276 | -3.419423998 | 0.0020755 | 0.007787363 | -1.783313859 |
| NKX2-1 | -2.052854817 | 8.992726021 | -3.419225928 | 0.002076539 | 0.00778899 | -1.783789168 |
| PLCD4 | 1.405324212 | 6.255052179 | 3.419034956 | 0.002077542 | 0.007790479 | -1.784247432 |
| TFAP2C | -1.255771139 | 4.481054662 | -3.418187138 | 0.002081998 | 0.007803087 | -1.786281783 |
| DYNC1LI2 | -1.357829677 | 9.653816434 | -3.418164413 | 0.002082118 | 0.007803087 | -1.786336309 |
| SIK1 | 1.573031227 | 10.08861186 | 3.417802365 | 0.002084023 | 0.007807955 | -1.787204988 |
| PTPN1 | -1.611840416 | 9.291798781 | -3.417501889 | 0.002085607 | 0.007810073 | -1.78792591 |
| KCNQ1OT1 | 1.32341871 | 10.4922329 | 3.417464599 | 0.002085803 | 0.007810073 | -1.788015376 |
| SPRR1A | 2.084772055 | 9.501356917 | 3.416167313 | 0.002092652 | 0.007831158 | -1.791127626 |
| CCDC47 | -1.107900422 | 8.033914249 | -3.415979288 | 0.002093646 | 0.0078326 | -1.791578672 |
| EEF2K | -1.38970986 | 6.008345759 | -3.414864231 | 0.002099553 | 0.007852415 | -1.794253344 |
| LOXL4 | 2.402160102 | 8.010400752 | 3.413991158 | 0.00210419 | 0.007867467 | -1.796347349 |
| TG | 1.371364388 | 6.843289813 | 3.41372555 | 0.002105602 | 0.00787046 | -1.796984352 |
| RIMBP2 | 1.627507066 | 5.860988191 | 3.412997667 | 0.002109477 | 0.007882655 | -1.798729928 |
| TRMT5 | -1.181331637 | 9.973880518 | -3.411761934 | 0.002116073 | 0.007905003 | -1.80169309 |
| TEKT2 | -2.331219354 | 5.861824512 | -3.411578785 | 0.002117052 | 0.007906364 | -1.802132228 |
| MYCN | 1.953457758 | 6.641897419 | 3.411317838 | 0.002118448 | 0.007909281 | -1.80275789 |
| NSUN7 | -2.040280253 | 5.866179172 | -3.410971729 | 0.0021203 | 0.007913901 | -1.803587713 |
| GPR39 | 1.716231654 | 7.489278517 | 3.41004046 | 0.002125293 | 0.007930236 | -1.805820348 |
| MOCS1 | -1.166683059 | 6.015114296 | -3.409327841 | 0.002129122 | 0.007942217 | -1.807528636 |
| PRKY | -1.775796526 | 5.108107442 | -3.408421747 | 0.002133999 | 0.007958104 | -1.809700532 |
| PIGV | -1.646941549 | 6.420920083 | -3.407376052 | 0.002139642 | 0.007976833 | -1.812206783 |
| RPL10A | -1.262120462 | 13.44641935 | -3.406618546 | 0.002143738 | 0.007989231 | -1.814022145 |
| SLC15A1 | 1.709430437 | 5.368038731 | 3.40653148 | 0.00214421 | 0.007989231 | -1.814230788 |
| KRT8P41 | 1.327501019 | 8.650009531 | 3.405661763 | 0.002148923 | 0.008004475 | -1.816314859 |
| TNFSF13B | 1.617653214 | 8.981768454 | 3.405350353 | 0.002150613 | 0.008007742 | -1.81706103 |
| MGC4473 | 2.328778027 | 7.47177946 | 3.405270758 | 0.002151046 | 0.008007742 | -1.817251744 |
| MRPS28 | -1.054021934 | 8.974876601 | -3.404530829 | 0.002155068 | 0.008020394 | -1.819024578 |
| LOC283861 | -1.335365053 | 4.004693671 | -3.404243689 | 0.00215663 | 0.008023888 | -1.819712513 |
| ATF4 | 1.363023872 | 13.59857779 | 3.40400259 | 0.002157943 | 0.008026451 | -1.820290126 |
| FANCB | 2.024552458 | 7.377907783 | 3.401049121 | 0.002174091 | 0.008084174 | -1.827364655 |
| TCF3 | 1.220137089 | 10.30647578 | 3.40045074 | 0.002177377 | 0.008094052 | -1.828797695 |
| PPP2R2C | 2.227206684 | 6.9000697 | 3.40022876 | 0.002178597 | 0.008094614 | -1.829329281 |
| FLJ36848 | 1.695858663 | 7.63117588 | 3.400194267 | 0.002178787 | 0.008094614 | -1.829411884 |
| ELF3 | -1.47068571 | 6.481056915 | -3.399112661 | 0.002184742 | 0.008114395 | -1.832001872 |
| DNAJC5G | 2.552027691 | 7.605165037 | 3.398882243 | 0.002186012 | 0.00811677 | -1.832553584 |
| TMEM68 | -1.430716651 | 5.914846302 | -3.398514715 | 0.002188041 | 0.008120526 | -1.833433566 |
| C12orf62 | -1.149492235 | 12.78620928 | -3.398470155 | 0.002188287 | 0.008120526 | -1.833540254 |
| FTL | -1.394340811 | 11.50644552 | -3.398306852 | 0.002189189 | 0.00812153 | -1.833931241 |
| KRT7 | 1.318074653 | 9.615364511 | 3.396642159 | 0.002198403 | 0.008152454 | -1.837916521 |
| SOX8 | 1.668951028 | 7.515550483 | 3.396572083 | 0.002198792 | 0.008152454 | -1.838084267 |
| BCAR3 | -1.955431087 | 6.731810595 | -3.39525567 | 0.002206107 | 0.008177217 | -1.841235224 |
| ZFPM1 | 1.498954709 | 11.06025192 | 3.394748317 | 0.002208932 | 0.00818533 | -1.842449498 |
| LOC100507800 | 2.281928665 | 7.270546046 | 3.394404843 | 0.002210847 | 0.008187758 | -1.843271513 |
| NUP210 | 1.015817231 | 5.748779717 | 3.394402403 | 0.00221086 | 0.008187758 | -1.843277352 |
| LOC401357 | 2.028753362 | 10.79017422 | 3.39269228 | 0.002220418 | 0.00821842 | -1.847369622 |
| PDDC1 | 1.283715083 | 10.62852704 | 3.39251452 | 0.002221413 | 0.00821974 | -1.847794953 |
| CHEK1 | 1.578255488 | 6.369843168 | 3.391318973 | 0.002228121 | 0.008242191 | -1.850655344 |
| FASTK | 1.450829342 | 12.00410244 | 3.391096058 | 0.002229374 | 0.008244455 | -1.851188636 |
| ARHGAP10 | -1.360658562 | 5.961255972 | -3.389201956 | 0.002240048 | 0.008274411 | -1.855719457 |
| PLAC4 | 1.759587716 | 8.213320835 | 3.388549824 | 0.002243735 | 0.008284055 | -1.857279185 |
| MTA2 | 1.168854887 | 7.700061553 | 3.388512251 | 0.002243947 | 0.008284055 | -1.857369044 |
| PRRX2 | 2.092317338 | 7.715636617 | 3.388040283 | 0.002246619 | 0.008291539 | -1.85849779 |
| COL20A1 | 1.709648294 | 6.302941212 | 3.387050448 | 0.002252233 | 0.008309873 | -1.860864864 |
| LOC727820 | -1.464382293 | 6.01724613 | -3.385519307 | 0.002260943 | 0.008334837 | -1.86452589 |
| SRRM3 | 1.823690922 | 6.526371239 | 3.384098252 | 0.002269056 | 0.008362349 | -1.867923138 |
| LINC00085 | 1.213571887 | 9.291713621 | 3.383810688 | 0.002270701 | 0.008366015 | -1.868610539 |
| CDC42 | -1.071772658 | 10.21449798 | -3.383621777 | 0.002271783 | 0.008367602 | -1.869062105 |
| C10orf32 | -1.268505564 | 8.633415027 | -3.383387103 | 0.002273127 | 0.008370156 | -1.869623047 |
| P2RX3 | 2.16629597 | 6.909586346 | 3.383246382 | 0.002273933 | 0.008370729 | -1.869959406 |
| C17orf96 | 2.249915471 | 11.74231866 | 3.383013066 | 0.002275271 | 0.008373256 | -1.870517079 |
| ZNF83 | -1.822516612 | 5.390685563 | -3.382547332 | 0.002277943 | 0.008380692 | -1.871630236 |
| PSG5 | 1.838872493 | 5.378823858 | 3.382349339 | 0.00227908 | 0.008382477 | -1.872103444 |
| AGR2 | -1.562798217 | 9.662667051 | -3.382155291 | 0.002280194 | 0.00838418 | -1.872567213 |
| NDUFB3 | -1.187708853 | 10.23941269 | -3.38146854 | 0.002284144 | 0.008396302 | -1.874208449 |
| AKR7L | 1.478472882 | 11.30562001 | 3.38113601 | 0.002286059 | 0.00840094 | -1.875003101 |
| AMZ2 | -1.03213014 | 9.393441655 | -3.380335688 | 0.002290674 | 0.008413091 | -1.876915524 |
| SH3BGRL3 | -1.326422409 | 10.78838453 | -3.380016984 | 0.002292514 | 0.008417447 | -1.877677043 |
| OR5L2 | 2.250956166 | 7.524708808 | 3.37809064 | 0.002303668 | 0.008455985 | -1.882279307 |
| IVD | -1.405568288 | 5.210110731 | -3.376784409 | 0.002311261 | 0.008481436 | -1.885399483 |
| OBSCN | 1.157787705 | 7.823533273 | 3.376214693 | 0.00231458 | 0.008490949 | -1.886760211 |
| C14orf162 | 1.755248486 | 6.806326666 | 3.376112912 | 0.002315173 | 0.008490949 | -1.8870033 |
| STAG3 | 1.033920563 | 8.9453814 | 3.373461405 | 0.002330686 | 0.008542865 | -1.893335033 |
| LACC1 | 1.754560251 | 8.063198953 | 3.373284225 | 0.002331726 | 0.008542865 | -1.893758067 |
| TMEM80 | -1.347314945 | 8.926212637 | -3.373240065 | 0.002331986 | 0.008542865 | -1.893863502 |
| KCTD5 | -1.611382414 | 6.910814987 | -3.373098108 | 0.00233282 | 0.008543486 | -1.894202429 |
| CDK5RAP2 | -1.552896588 | 7.053307737 | -3.372772941 | 0.002334731 | 0.008548051 | -1.89497876 |
| DHCR7 | 1.109113111 | 10.45312365 | 3.372156142 | 0.00233836 | 0.008558902 | -1.896451274 |
| GPR37L1 | 1.8849796 | 7.197850045 | 3.371987129 | 0.002339355 | 0.008560109 | -1.896854752 |
| MAGEA6 | 2.073996546 | 6.355079385 | 3.371489346 | 0.002342289 | 0.008565972 | -1.898043037 |
| DDX46 | -1.69390641 | 5.774252095 | -3.369996821 | 0.002351108 | 0.008593336 | -1.901605527 |
| RPL37A | -1.482559207 | 13.98393126 | -3.369531791 | 0.002353862 | 0.008599307 | -1.90271538 |
| PLEKHG4B | 2.044025172 | 11.24430905 | 3.368960037 | 0.002357253 | 0.008606013 | -1.904079862 |
| LOC647979 | -1.827650765 | 11.97748791 | -3.365974836 | 0.002375032 | 0.008663546 | -1.911202569 |
| HEATR8 | 1.735666479 | 8.258295557 | 3.365799994 | 0.002376078 | 0.008664902 | -1.911619667 |
| TOX2 | -1.565596475 | 10.68795915 | -3.365599034 | 0.00237728 | 0.008666828 | -1.912099062 |
| THAP8 | 1.486110311 | 9.753462077 | 3.365380092 | 0.00237859 | 0.008669148 | -1.912621344 |
| MYO5C | -1.93119158 | 8.007033084 | -3.364909507 | 0.002381409 | 0.008676962 | -1.913743866 |
| NCR3 | 1.41359629 | 7.080101223 | 3.364764006 | 0.002382281 | 0.008677682 | -1.91409093 |
| ANGPT4 | 2.360942725 | 6.799887686 | 3.364561807 | 0.002383493 | 0.00867964 | -1.914573223 |
| PITPNA | -1.227436018 | 9.134690048 | -3.363763823 | 0.002388284 | 0.008694369 | -1.916476503 |
| MBD4 | -1.584181666 | 7.269590806 | -3.363531738 | 0.00238968 | 0.008694783 | -1.91703002 |
| DLST | -1.620956442 | 6.913344247 | -3.362907885 | 0.002393434 | 0.008702326 | -1.918517822 |
| LOC100130713 | 2.042910222 | 5.7489336 | 3.362850016 | 0.002393782 | 0.008702326 | -1.918655825 |
| SNED1 | 1.507080325 | 7.577826658 | 3.361685631 | 0.002400806 | 0.008724787 | -1.921432416 |
| LOC100144602 | 1.365175022 | 4.778892124 | 3.361600902 | 0.002401318 | 0.008724787 | -1.921634447 |
| PCOLCE2 | -1.7537696 | 7.623965827 | -3.361274368 | 0.002403291 | 0.008729492 | -1.922413024 |
| HOXC10 | 1.565828658 | 4.068636489 | 3.360956272 | 0.002405215 | 0.008734014 | -1.923171457 |
| DHPS | -1.170688601 | 5.921868378 | -3.360584865 | 0.002407464 | 0.008738623 | -1.924056961 |
| OTUD6A | 2.218217478 | 6.766043587 | 3.360522201 | 0.002407843 | 0.008738623 | -1.924206361 |
| ADCY9 | -1.218153727 | 8.067207745 | -3.359882993 | 0.002411718 | 0.008749732 | -1.925730258 |
| TMEM11 | -1.223498462 | 9.19197922 | -3.359792929 | 0.002412265 | 0.008749732 | -1.925944963 |
| SLC36A1 | 1.631755754 | 6.490122548 | 3.359390326 | 0.002414709 | 0.008756128 | -1.926904718 |
| SRSF11 | -1.774501078 | 8.413474053 | -3.359144899 | 0.0024162 | 0.008759066 | -1.927489763 |
| ARFGAP3 | -1.301788751 | 8.565715443 | -3.357932483 | 0.00242358 | 0.008783343 | -1.93037966 |
| TSR2 | -1.005191038 | 6.036797007 | -3.357450946 | 0.002426517 | 0.00879095 | -1.931527332 |
| RPL21P44 | -1.554850502 | 10.09162864 | -3.357364336 | 0.002427046 | 0.00879095 | -1.93173375 |
| SKA3 | 1.447701935 | 6.311148898 | 3.35722319 | 0.002427907 | 0.008791595 | -1.932070136 |
| CD3E | 2.222367432 | 7.507713109 | 3.356396202 | 0.002432962 | 0.00880742 | -1.934040942 |
| OSMR | 2.077161455 | 9.788764523 | 3.355876908 | 0.002436142 | 0.008816448 | -1.935278383 |
| ZFP82 | -1.483069868 | 4.439356698 | -3.355698218 | 0.002437237 | 0.008817929 | -1.935704172 |
| SCARF2 | 1.315344994 | 7.158812768 | 3.354621663 | 0.002443844 | 0.008836862 | -1.938269242 |
| SP2 | -1.198371999 | 7.175486768 | -3.354426493 | 0.002445043 | 0.008838715 | -1.938734233 |
| CDC16 | -1.117625301 | 7.688161469 | -3.353304132 | 0.002451953 | 0.008861203 | -1.941408046 |
| CCDC158 | 2.830447142 | 5.14272895 | 3.352817685 | 0.002454954 | 0.008869555 | -1.942566809 |
| HAMP | 1.68778527 | 6.481338946 | 3.351695926 | 0.002461887 | 0.008892106 | -1.945238693 |
| LOC284570 | 2.920174998 | 11.25265901 | 3.349606646 | 0.00247485 | 0.008935982 | -1.950214172 |
| SERPIND1 | 1.597101742 | 6.61561443 | 3.348645795 | 0.002480834 | 0.008953 | -1.952501972 |
| INO80D | 2.276400892 | 13.45028629 | 3.348395786 | 0.002482394 | 0.008955043 | -1.953097205 |
| MYCNOS | 1.671288033 | 6.202779085 | 3.348331788 | 0.002482793 | 0.008955043 | -1.953249573 |
| CRISPLD2 | 2.018966155 | 8.177892299 | 3.348213096 | 0.002483534 | 0.008955203 | -1.953532152 |
| DGKQ | 1.660528168 | 6.219188643 | 3.347881083 | 0.002485607 | 0.008960167 | -1.954322581 |
| ZNF681 | -1.198685461 | 8.713618056 | -3.347728846 | 0.002486558 | 0.008961084 | -1.954685004 |
| PLA2G5 | 1.610953234 | 8.813953706 | 3.34761048 | 0.002487298 | 0.008961239 | -1.954966789 |
| SENP7 | -1.24035933 | 8.802459008 | -3.347347375 | 0.002488943 | 0.008964655 | -1.955593125 |
| C6orf132 | -1.119445679 | 6.367815443 | -3.346746593 | 0.002492703 | 0.008973174 | -1.957023254 |
| HHIPL1 | 1.767607291 | 7.029753921 | 3.344238319 | 0.002508463 | 0.009024853 | -1.962992985 |
| SLC40A1 | -2.126763686 | 9.958690774 | -3.343355094 | 0.002514035 | 0.009039903 | -1.965094663 |
| HOXA2 | -1.527488141 | 5.218090886 | -3.343352499 | 0.002514052 | 0.009039903 | -1.965100836 |
| RASA3 | 1.01533535 | 5.29636212 | 3.341769125 | 0.002524071 | 0.009073394 | -1.968868013 |
| LMAN1L | 2.617288338 | 7.365903695 | 3.341577677 | 0.002525285 | 0.009075222 | -1.969323461 |
| DTD1 | -1.490455855 | 8.440090033 | -3.341073297 | 0.002528487 | 0.009083293 | -1.970523314 |
| ZBTB7A | 1.107912835 | 9.055795414 | 3.341001336 | 0.002528944 | 0.009083293 | -1.970694494 |
| IGFBP2 | 1.753690778 | 12.23794867 | 3.340883235 | 0.002529694 | 0.009083452 | -1.970975428 |
| THEM5 | 1.930735852 | 10.77656026 | 3.337987529 | 0.002548158 | 0.009142093 | -1.977862415 |
| MUC20 | 1.417413599 | 8.746078975 | 3.337174286 | 0.002553366 | 0.009158226 | -1.97979617 |
| PPP3R2 | 2.142647692 | 6.404422264 | 3.336119609 | 0.002560137 | 0.009177612 | -1.982303742 |
| FGFBP2 | -1.692845654 | 6.174979064 | -3.336110055 | 0.002560198 | 0.009177612 | -1.982326456 |
| ALDH5A1 | -2.168367488 | 5.609007828 | -3.335742072 | 0.002562565 | 0.009183535 | -1.983201289 |
| EVPL | 1.980048396 | 10.37087456 | 3.33344288 | 0.002577398 | 0.009232458 | -1.988666475 |
| RCOR2 | 1.394133109 | 14.93671062 | 3.333403687 | 0.002577652 | 0.009232458 | -1.988759624 |
| KRT76 | 2.728091663 | 7.03835755 | 3.333220598 | 0.002578837 | 0.009233452 | -1.989194763 |
| INPPL1 | -1.150136814 | 6.819334882 | -3.333138973 | 0.002579365 | 0.009233452 | -1.989388753 |
| SNRPB2 | -1.02586312 | 6.73059068 | -3.332726256 | 0.002582039 | 0.009240451 | -1.990369593 |
| C3orf54 | 1.656648241 | 8.776930619 | 3.332532693 | 0.002583293 | 0.009242369 | -1.990829587 |
| CYP20A1 | -1.202957983 | 6.104838994 | -3.331351054 | 0.002590966 | 0.009267243 | -1.993637476 |
| COL5A2 | 1.979119072 | 9.492548515 | 3.330873102 | 0.002594076 | 0.009275786 | -1.994773106 |
| PRDX3 | -1.196686435 | 6.276953292 | -3.330333225 | 0.002597593 | 0.00928578 | -1.996055796 |
| GAN | 1.641355783 | 7.79931444 | 3.329910819 | 0.002600348 | 0.009292893 | -1.997059332 |
| SNCA | -1.457945803 | 5.503248382 | -3.329806651 | 0.002601027 | 0.009292893 | -1.997306802 |
| SUCLG2 | -1.31207213 | 5.722782009 | -3.329514783 | 0.002602933 | 0.009297119 | -1.998000172 |
| CA1 | 2.428027319 | 7.190391136 | 3.327970665 | 0.002613037 | 0.009328029 | -2.00166803 |
| ZNF584 | -1.440052958 | 4.647194386 | -3.32782938 | 0.002613964 | 0.009328747 | -2.002003602 |
| P2RY13 | 2.184669289 | 8.380985396 | 3.32749739 | 0.002616142 | 0.009333931 | -2.002792103 |
| PLCXD2 | 1.88370861 | 8.086743311 | 3.326472803 | 0.002622875 | 0.009352765 | -2.005225378 |
| LAG3 | 1.772191964 | 8.056801795 | 3.32583408 | 0.002627081 | 0.009365167 | -2.006742122 |
| COMP | 2.328941076 | 7.115355705 | 3.324138338 | 0.00263828 | 0.009402481 | -2.010768363 |
| ZNF727 | -1.575402494 | 11.65839028 | -3.323915817 | 0.002639753 | 0.009405124 | -2.01129664 |
| GAS1 | -1.382172666 | 6.41139085 | -3.322760587 | 0.002647412 | 0.009429801 | -2.014038999 |
| ATG5 | -1.556209727 | 7.177196455 | -3.322595452 | 0.002648508 | 0.009431095 | -2.014430976 |
| KLHDC8B | -1.528684787 | 6.203694511 | -3.322151874 | 0.002651456 | 0.009438477 | -2.01548385 |
| PCCA | -1.251777986 | 7.733765056 | -3.322062718 | 0.002652049 | 0.009438477 | -2.015695462 |
| UBL3 | -1.711895928 | 8.464425429 | -3.320779075 | 0.0026606 | 0.009466288 | -2.018741963 |
| EIF3I | -1.595780891 | 7.268485721 | -3.320175023 | 0.002664632 | 0.009478015 | -2.020175416 |
| RRH | 2.595211806 | 9.331203789 | 3.319474957 | 0.002669314 | 0.009492041 | -2.021836586 |
| QARS | -1.936753619 | 8.17863041 | -3.318307076 | 0.002677141 | 0.009514613 | -2.024607516 |
| MGC23284 | 1.750239752 | 6.567746348 | 3.318098893 | 0.002678538 | 0.00951695 | -2.025101413 |
| SMAD4 | -1.118812158 | 6.484909449 | -3.317311802 | 0.002683829 | 0.009533113 | -2.026968612 |
| NFASC | 1.631084887 | 7.150696785 | 3.316437633 | 0.002689716 | 0.009551388 | -2.029042183 |
| FAS | -1.283354135 | 6.924931786 | -3.316318403 | 0.00269052 | 0.009551605 | -2.029324984 |
| C19orf63 | -1.688316244 | 9.742744643 | -3.315298347 | 0.002697407 | 0.009573406 | -2.031744298 |
| RAB1B | 1.87802713 | 10.44433544 | 3.315188612 | 0.002698149 | 0.009573406 | -2.032004545 |
| C21orf58 | 1.624526055 | 7.766467195 | 3.312656159 | 0.002715327 | 0.009631699 | -2.038009529 |
| FAM82A2 | -1.438779701 | 8.528736154 | -3.312541686 | 0.002716106 | 0.009631806 | -2.038280926 |
| CTSA | 1.722984271 | 12.03995325 | 3.312174866 | 0.002718604 | 0.009638005 | -2.039150571 |
| EEF1E1 | -1.155886375 | 6.873713194 | -3.311849884 | 0.002720819 | 0.009638443 | -2.039920997 |
| ALX3 | 1.89615581 | 6.245402426 | 3.311818223 | 0.002721034 | 0.009638443 | -2.039996054 |
| TGOLN2 | -1.865004002 | 10.19184674 | -3.311810747 | 0.002721085 | 0.009638443 | -2.040013776 |
| LHB | 1.943525684 | 9.90723597 | 3.311716962 | 0.002721725 | 0.009638443 | -2.040236103 |
| CREBL2 | -1.34769281 | 6.998062315 | -3.311412633 | 0.002723801 | 0.00964314 | -2.040957529 |
| IFI27L2 | 1.083883427 | 12.33015621 | 3.311065798 | 0.002726169 | 0.009648867 | -2.041779684 |
| ZNF625 | 1.108935349 | 8.737450851 | 3.31083692 | 0.002727733 | 0.009651746 | -2.04232221 |
| WDR3 | -1.196446616 | 6.4516195 | -3.309753942 | 0.002735143 | 0.009673444 | -2.044889069 |
| ZKSCAN1 | 1.239990695 | 13.04533899 | 3.309696547 | 0.002735537 | 0.009673444 | -2.045025098 |
| SPAG17 | -1.986941211 | 5.417571989 | -3.309611281 | 0.002736121 | 0.009673444 | -2.045227179 |
| AGTPBP1 | -2.3784578 | 7.244298764 | -3.308848248 | 0.002741356 | 0.009689288 | -2.047035479 |
| C3orf55 | -1.193390286 | 4.604492903 | -3.308513869 | 0.002743653 | 0.009694289 | -2.047827866 |
| MYOD1 | 1.821758883 | 6.469294449 | 3.308422906 | 0.002744278 | 0.009694289 | -2.048043417 |
| LOC100506458 | 1.716761656 | 6.069013626 | 3.306069435 | 0.002760502 | 0.009744363 | -2.053619545 |
| GABRE | 1.52542403 | 11.057167 | 3.305963958 | 0.002761231 | 0.009744363 | -2.053869418 |
| C4orf26 | 1.93593934 | 5.477840343 | 3.305927452 | 0.002761484 | 0.009744363 | -2.053955897 |
| GAL | 1.960838074 | 5.725706618 | 3.30505168 | 0.002767547 | 0.009763079 | -2.056030445 |
| PDE3B | 1.220007286 | 9.01097237 | 3.301021653 | 0.002795612 | 0.009852691 | -2.065574042 |
| ACTL8 | 1.406641436 | 3.652410103 | 3.30096435 | 0.002796013 | 0.009852691 | -2.06570971 |
| TTC39B | 1.170308035 | 7.942499894 | 3.30084698 | 0.002796835 | 0.009852887 | -2.065987584 |
| C10orf46 | -1.334802154 | 6.151437565 | -3.300398833 | 0.002799974 | 0.009861245 | -2.067048542 |
| COBLL1 | -1.159532477 | 5.66497711 | -3.299648047 | 0.002805241 | 0.00987709 | -2.06882585 |
| MICALCL | -1.555214297 | 7.051999892 | -3.299229304 | 0.002808183 | 0.009884741 | -2.069817055 |
| R3HDML | 3.055913283 | 6.411117177 | 3.296872373 | 0.002824795 | 0.009937779 | -2.075395197 |
| TMEM98 | -1.237121799 | 8.612583623 | -3.296354092 | 0.002828461 | 0.009947955 | -2.0766216 |
| LRP2BP | -1.021067032 | 4.760558613 | -3.295947404 | 0.002831341 | 0.009955297 | -2.077583885 |
| KRTAP13-4 | 1.83220456 | 6.627662452 | 3.295731681 | 0.00283287 | 0.009955297 | -2.078094299 |
| PAX5 | 1.767631251 | 6.840361596 | 3.295731486 | 0.002832871 | 0.009955297 | -2.078094762 |
| C19orf23 | 1.719963739 | 7.408936644 | 3.294875488 | 0.002838945 | 0.009973917 | -2.080119976 |
| CRNKL1 | -1.033421088 | 5.318539353 | -3.294397289 | 0.002842343 | 0.009980348 | -2.081251261 |
| MRPL12 | -1.051638627 | 6.543328164 | -3.294290445 | 0.002843103 | 0.009980348 | -2.081504013 |
| KANK2 | -1.204062492 | 11.03934988 | -3.293939778 | 0.002845599 | 0.009986382 | -2.082333541 |
| TAC1 | 1.647480302 | 5.914993943 | 3.293295608 | 0.002850188 | 0.009995951 | -2.083857278 |
| BGLAP | 1.407801394 | 8.515527391 | 3.293232705 | 0.002850636 | 0.009995951 | -2.084006062 |
| ACTN1 | -1.423804053 | 9.024446509 | -3.293229855 | 0.002850657 | 0.009995951 | -2.084012805 |
| ACPT | 2.101696404 | 10.1964282 | 3.291299497 | 0.002864454 | 0.010041595 | -2.088578172 |
| CLPX | -1.022652326 | 7.813636033 | -3.2911638 | 0.002865427 | 0.010042267 | -2.088899061 |
| ADRB3 | 1.881710906 | 6.994441372 | 3.290581124 | 0.002869606 | 0.01005201 | -2.090276876 |
| KIAA0664 | -1.23056733 | 4.757425107 | -3.290558245 | 0.00286977 | 0.01005201 | -2.090330976 |
| STRN4 | 1.345896612 | 10.41510883 | 3.289878021 | 0.002874656 | 0.010066383 | -2.09193933 |
| TDP2 | -1.548548388 | 8.987136455 | -3.289592991 | 0.002876706 | 0.010067428 | -2.092613229 |
| GIPC1 | -1.141690628 | 5.615327587 | -3.289541931 | 0.002877073 | 0.010067428 | -2.092733947 |
| IMMP1L | -1.476800107 | 5.227587286 | -3.289510051 | 0.002877303 | 0.010067428 | -2.09280932 |
| TMEM5 | -1.174200846 | 7.203971329 | -3.288580975 | 0.002883995 | 0.0100881 | -2.095005758 |
| PPP2CB | -1.252134404 | 10.08045726 | -3.288248968 | 0.00288639 | 0.010092603 | -2.095790599 |
| RNF11 | -1.582362678 | 10.01107811 | -3.288185075 | 0.002886852 | 0.010092603 | -2.095941633 |
| EFTUD1 | 3.127526797 | 7.322013127 | 3.287621482 | 0.002890923 | 0.010104089 | -2.097273842 |
| FABP6 | -2.44607316 | 5.144286158 | -3.287400396 | 0.002892521 | 0.010106929 | -2.097796416 |
| HSBP1 | -1.00815462 | 10.6623073 | -3.285503626 | 0.00290627 | 0.010152213 | -2.102279167 |
| TAOK3 | -1.138046218 | 9.25327384 | -3.285344183 | 0.002907429 | 0.010153503 | -2.102655942 |
| PTPRA | -1.221253043 | 8.549026498 | -3.285213626 | 0.002908378 | 0.01015406 | -2.102964449 |
| RANBP17 | -1.645007826 | 5.108914814 | -3.285020822 | 0.00290978 | 0.010156198 | -2.10342004 |
| ZHX2 | -1.127469251 | 6.742584223 | -3.284479084 | 0.002913723 | 0.010167202 | -2.104700095 |
| EIF3H | -1.193502177 | 13.33985432 | -3.284130264 | 0.002916265 | 0.010171416 | -2.105524268 |
| MMAA | -1.389589776 | 4.878481953 | -3.284096296 | 0.002916512 | 0.010171416 | -2.105604523 |
| F8 | 1.270262019 | 6.662031794 | 3.283964275 | 0.002917475 | 0.010172015 | -2.105916442 |
| SLC10A1 | 2.454889218 | 6.388212057 | 3.28258408 | 0.002927557 | 0.010201635 | -2.109177072 |
| CLUAP1 | -1.027913316 | 8.579890968 | -3.282372087 | 0.002929108 | 0.01020301 | -2.109677843 |
| CYP51A1 | -1.735025407 | 8.608161736 | -3.282313398 | 0.002929538 | 0.01020301 | -2.109816478 |
| WSB1 | -2.266000129 | 11.09212992 | -3.281303577 | 0.00293694 | 0.010226022 | -2.11220169 |
| UBL4B | 1.718099269 | 5.32554661 | 3.280919893 | 0.002939757 | 0.010229166 | -2.113107881 |
| MAMDC2 | -1.711685501 | 7.43132677 | -3.280900828 | 0.002939897 | 0.010229166 | -2.113152907 |
| C7orf44 | 1.536869669 | 8.427423473 | 3.280855707 | 0.002940229 | 0.010229166 | -2.113259472 |
| GPIHBP1 | -1.812587305 | 10.03507289 | -3.280266654 | 0.00294456 | 0.010241463 | -2.11465061 |
| ADAMTSL2 | -1.299641595 | 6.092196504 | -3.277430177 | 0.002965501 | 0.010311511 | -2.121347973 |
| FBRSL1 | 1.885699293 | 12.22571965 | 3.276797101 | 0.002970195 | 0.01032225 | -2.122842443 |
| IQSEC3 | 2.094750578 | 8.850514666 | 3.275878341 | 0.002977019 | 0.010343132 | -2.125011105 |
| VAMP2 | 1.304786826 | 9.052272552 | 3.275771706 | 0.002977812 | 0.010343132 | -2.125262794 |
| SDC4 | -1.21279228 | 7.919833703 | -3.275654592 | 0.002978683 | 0.010343365 | -2.12553921 |
| PTPN5 | 2.152559848 | 8.067247633 | 3.275200656 | 0.002982062 | 0.010352304 | -2.126610573 |
| TMEM220 | -1.079780536 | 6.131566118 | -3.274772703 | 0.002985251 | 0.010359337 | -2.127620554 |
| PTPMT1 | -1.158024337 | 8.183393576 | -3.274639386 | 0.002986246 | 0.010359337 | -2.127935175 |
| TMEM63B | 1.530072256 | 7.860883833 | 3.274604651 | 0.002986505 | 0.010359337 | -2.128017146 |
| PWWP2B | 1.801692216 | 11.22662328 | 3.2742928 | 0.002988832 | 0.010364613 | -2.128753076 |
| KCNMB1 | 1.175774859 | 8.627055137 | 3.273634688 | 0.002993748 | 0.010378864 | -2.130306044 |
| LOC100509861 | 1.943198427 | 6.439809484 | 3.272766691 | 0.003000244 | 0.010398582 | -2.13235409 |
| LOC401847 | 1.971532629 | 6.06796642 | 3.272420371 | 0.00300284 | 0.010404753 | -2.133171172 |
| ALPP | 2.0179534 | 8.74816023 | 3.272313342 | 0.003003642 | 0.010404753 | -2.133423682 |
| TET1 | 1.751093078 | 6.918332377 | 3.272191191 | 0.003004559 | 0.010405124 | -2.133711865 |
| CST7 | -1.730742021 | 4.56652079 | -3.27122606 | 0.003011808 | 0.010427421 | -2.135988679 |
| JMJD4 | -1.233812856 | 4.344646085 | -3.270395338 | 0.003018061 | 0.010446258 | -2.137948193 |
| SSFA2 | -1.531746215 | 8.918858776 | -3.269656826 | 0.00302363 | 0.01046272 | -2.139690033 |
| ROCK1 | -1.158767765 | 8.112851843 | -3.269524022 | 0.003024633 | 0.010463374 | -2.140003244 |
| EBPL | -1.101602214 | 9.508781509 | -3.26922009 | 0.003026928 | 0.0104685 | -2.140720034 |
| AP1S2 | -1.243108288 | 7.610305959 | -3.267940913 | 0.003036609 | 0.010499157 | -2.143736532 |
| FNTA | -1.493106092 | 9.668340569 | -3.267361842 | 0.003041001 | 0.010511517 | -2.145101912 |
| LOC100509683 | -1.610645864 | 5.942365228 | -3.266842353 | 0.003044946 | 0.010521593 | -2.146326723 |
| CCNT1 | 1.231459873 | 7.730263275 | 3.265414664 | 0.003055815 | 0.010554213 | -2.149692407 |
| LINC00176 | 1.86907595 | 7.128483247 | 3.262739731 | 0.003076278 | 0.010615004 | -2.155996773 |
| HUWE1 | -1.281422995 | 6.507038571 | -3.262660901 | 0.003076884 | 0.010615004 | -2.15618253 |
| ACCN4 | 2.20001514 | 5.472434524 | 3.262575095 | 0.003077542 | 0.010615004 | -2.156384724 |
| MST1R | -1.056241458 | 5.320589598 | -3.262447786 | 0.00307852 | 0.010615529 | -2.156684712 |
| VPS4B | -1.270904974 | 5.777020581 | -3.262016417 | 0.003081835 | 0.010624111 | -2.157701141 |
| SNRPB | -1.245463772 | 6.975145555 | -3.26173329 | 0.003084012 | 0.010628769 | -2.15836824 |
| CPLX3 | 1.738977609 | 5.584254647 | 3.261594143 | 0.003085083 | 0.01062961 | -2.158696086 |
| EID2 | -1.38459683 | 7.350135759 | -3.261101137 | 0.003088879 | 0.010639841 | -2.159857622 |
| SAR1A | 1.394898953 | 13.98564271 | 3.260974248 | 0.003089857 | 0.010640359 | -2.160156564 |
| LPXN | 1.17219878 | 7.135652608 | 3.259846043 | 0.003098565 | 0.010667489 | -2.162814334 |
| ARSD | 2.074558267 | 7.957427825 | 3.259081435 | 0.003104479 | 0.010684991 | -2.164615344 |
| GCNT3 | 1.907602233 | 7.288315231 | 3.258593534 | 0.003108259 | 0.010692915 | -2.16576449 |
| AKR1A1 | -1.647052505 | 8.869050194 | -3.258569664 | 0.003108444 | 0.010692915 | -2.165820711 |
| EFCAB4A | 1.143985562 | 8.535361053 | 3.258363001 | 0.003110047 | 0.010693902 | -2.166307436 |
| FLJ35390 | 1.577231983 | 7.066448232 | 3.258318217 | 0.003110394 | 0.010693902 | -2.16641291 |
| HIST1H2BL | 1.512753974 | 11.74110114 | 3.256925421 | 0.003121216 | 0.010728242 | -2.169692842 |
| WISP1 | 2.259532593 | 7.437629897 | 3.254115175 | 0.003143162 | 0.010800021 | -2.17630901 |
| TTN | -2.462087023 | 7.142821424 | -3.253694242 | 0.003146462 | 0.010806353 | -2.177299813 |
| OSR2 | 1.79665898 | 6.436862 | 3.251905185 | 0.003160525 | 0.010848857 | -2.18151035 |
| LOC100652987 | -1.178557715 | 9.120555771 | -3.25126179 | 0.003165597 | 0.010863369 | -2.183024345 |
| FASTKD5 | -1.316385493 | 9.097243291 | -3.251140983 | 0.00316655 | 0.010863742 | -2.183308606 |
| MCART3P | 1.120475238 | 6.754127245 | 3.250858413 | 0.003168781 | 0.010868496 | -2.183973482 |
| TCAM1P | 2.966822484 | 6.758855818 | 3.249710216 | 0.003177861 | 0.010893829 | -2.186674899 |
| TUBB4A | 1.832850764 | 6.536207041 | 3.249320281 | 0.00318095 | 0.010896802 | -2.187592228 |
| LOC100128644 | 1.381052918 | 6.143165689 | 3.249301148 | 0.003181102 | 0.010896802 | -2.187637237 |
| ZNF292 | 1.617452923 | 10.39956312 | 3.249279991 | 0.00318127 | 0.010896802 | -2.187687008 |
| HNRPLL | -1.19895758 | 6.083576743 | -3.24844058 | 0.003187931 | 0.01091671 | -2.189661572 |
| ZNF131 | -1.779953502 | 7.821765547 | -3.247731253 | 0.00319357 | 0.01093311 | -2.191329976 |
| PDE4DIP | -1.334567733 | 6.259198135 | -3.247453783 | 0.003195778 | 0.010937759 | -2.191982568 |
| EIF4E | -1.106509445 | 7.568852725 | -3.247241462 | 0.003197469 | 0.010937925 | -2.192481922 |
| GRIN2A | 2.508608565 | 6.714205985 | 3.24723412 | 0.003197528 | 0.010937925 | -2.192499188 |
| DQX1 | 1.161221044 | 5.045703335 | 3.246355128 | 0.003204537 | 0.010956076 | -2.194566318 |
| FADD | -1.119626526 | 8.220598537 | -3.246190259 | 0.003205854 | 0.010957664 | -2.194954018 |
| C7orf28B | -1.178193196 | 9.803025019 | -3.245063986 | 0.00321486 | 0.010985424 | -2.197602287 |
| TBC1D23 | -1.160648188 | 7.68646295 | -3.244961228 | 0.003215683 | 0.010985424 | -2.197843889 |
| LRP4 | -1.435364999 | 6.239783294 | -3.243730007 | 0.00322556 | 0.011013313 | -2.200738457 |
| C8orf58 | -1.087604301 | 6.833111569 | -3.243616374 | 0.003226473 | 0.011013507 | -2.201005581 |
| GAFA2 | 1.644586126 | 4.911499139 | 3.242077557 | 0.003238861 | 0.011048757 | -2.204622611 |
| LOC100653189 | -1.079506917 | 6.549043941 | -3.241931621 | 0.003240038 | 0.011048757 | -2.2049656 |
| TLE2 | -1.252053446 | 8.328323781 | -3.241923511 | 0.003240104 | 0.011048757 | -2.204984661 |
| POTEE | -1.187332925 | 10.17013473 | -3.241907187 | 0.003240236 | 0.011048757 | -2.205023026 |
| SNRPD1 | 1.099411664 | 10.89613315 | 3.241605021 | 0.003242675 | 0.011053141 | -2.205733176 |
| SIN3B | 1.582034527 | 7.759274058 | 3.241534988 | 0.00324324 | 0.011053141 | -2.205897762 |
| ATP1A3 | -1.207465985 | 6.247641197 | -3.241358324 | 0.003244667 | 0.011055075 | -2.206312942 |
| DNASE1L3 | -1.741940683 | 7.428522547 | -3.240430013 | 0.003252176 | 0.011074249 | -2.208494417 |
| PRRT2 | 1.739741455 | 8.395869673 | 3.240343303 | 0.003252878 | 0.011074249 | -2.208698167 |
| NDUFB2 | -1.109524226 | 12.83522253 | -3.239840956 | 0.003256949 | 0.01108284 | -2.209878532 |
| NPR2 | 1.009620305 | 7.517147524 | 3.239819274 | 0.003257125 | 0.01108284 | -2.209929476 |
| KGFLP1 | 1.452701137 | 9.478611936 | 3.239098015 | 0.003262979 | 0.011099422 | -2.211624081 |
| NIM1 | -1.975481434 | 4.73564527 | -3.238625562 | 0.003266819 | 0.01110701 | -2.212734027 |
| ZNF512 | -1.632731533 | 6.154309036 | -3.238324585 | 0.003269267 | 0.011112398 | -2.213441087 |
| CHI3L2 | -2.507543619 | 5.31170544 | -3.238184319 | 0.003270409 | 0.011113342 | -2.213770592 |
| ARMC5 | -1.282306335 | 7.01528522 | -3.237421018 | 0.003276629 | 0.011131537 | -2.215563595 |
| TAF15 | -1.016011595 | 7.717310077 | -3.236190054 | 0.003286683 | 0.011156854 | -2.218454772 |
| RBM10 | -1.12524172 | 7.543762888 | -3.235266335 | 0.003294248 | 0.011179582 | -2.220624022 |
| C4orf34 | -1.246502305 | 9.200669368 | -3.234966777 | 0.003296704 | 0.011184968 | -2.221327445 |
| GRPR | 1.943145239 | 6.249856626 | 3.234486467 | 0.003300647 | 0.011195392 | -2.222455253 |
| DENND1A | 1.240295149 | 8.797778061 | 3.2341405 | 0.00330349 | 0.01120208 | -2.223267569 |
| PLCH1 | -1.469074851 | 5.361087327 | -3.233698614 | 0.003307124 | 0.011211448 | -2.224305047 |
| CD27 | 1.11073932 | 7.710951621 | 3.233217433 | 0.003311086 | 0.011221193 | -2.225434716 |
| SCARNA17 | 1.717825117 | 12.10773575 | 3.233137649 | 0.003311744 | 0.011221193 | -2.225622019 |
| H3F3A | -1.014403991 | 12.60585486 | -3.232793253 | 0.003314583 | 0.011225261 | -2.226430506 |
| ADRB2 | -1.60897566 | 8.319696449 | -3.232780264 | 0.00331469 | 0.011225261 | -2.226460998 |
| TRAK1 | 1.878014382 | 11.26826544 | 3.232635425 | 0.003315885 | 0.011225928 | -2.226801003 |
| RRS1 | -1.371368531 | 6.344954369 | -3.23254481 | 0.003316632 | 0.011225928 | -2.227013717 |
| OXSR1 | -1.552983314 | 8.664665771 | -3.232175057 | 0.003319685 | 0.011233303 | -2.227881664 |
| HLA-B | -1.341910297 | 11.27861883 | -3.231824595 | 0.003322581 | 0.011240145 | -2.228704291 |
| LOC100506542 | -1.28104591 | 6.047803501 | -3.230953654 | 0.003329787 | 0.011261464 | -2.230748456 |
| CTSH | -2.219103596 | 12.86768992 | -3.230851504 | 0.003330634 | 0.011261464 | -2.230988194 |
| ARHGAP29 | -1.49461648 | 6.399422223 | -3.230379202 | 0.003334549 | 0.01126976 | -2.232096612 |
| ADSSL1 | -1.367746853 | 5.052054249 | -3.23034418 | 0.00333484 | 0.01126976 | -2.232178801 |
| CRNN | 1.913139014 | 7.305896567 | 3.229925284 | 0.003338316 | 0.011278546 | -2.233161824 |
| KRTAP4-12 | 2.053614205 | 7.273432224 | 3.229285783 | 0.003343631 | 0.011290569 | -2.234662434 |
| MED7 | -1.150883437 | 5.935566704 | -3.227673253 | 0.003357067 | 0.011332966 | -2.238445746 |
| C3orf51 | 2.254830059 | 6.694093065 | 3.227355191 | 0.003359724 | 0.011336765 | -2.239191888 |
| KRT85 | 2.346305544 | 7.408384614 | 3.22732744 | 0.003359956 | 0.011336765 | -2.239256987 |
| ESM1 | 2.055582451 | 7.327226746 | 3.22691391 | 0.003363413 | 0.011345453 | -2.240227039 |
| BDH2 | -1.840318547 | 9.174691604 | -3.226774843 | 0.003364576 | 0.011346402 | -2.240553249 |
| TBC1D4 | -1.50207399 | 6.088676675 | -3.226477693 | 0.003367063 | 0.011351641 | -2.241250254 |
| TMEM71 | -2.069739786 | 5.47092896 | -3.226378365 | 0.003367895 | 0.011351641 | -2.241483236 |
| MAPRE1 | -1.317250276 | 8.067477207 | -3.225918015 | 0.003371752 | 0.011358689 | -2.24256298 |
| ZNF646 | 2.200935739 | 10.70961378 | 3.225676768 | 0.003373775 | 0.011362529 | -2.243128797 |
| ACOT11 | 1.061975963 | 7.194966276 | 3.223149764 | 0.003395038 | 0.011431146 | -2.249054522 |
| HERPUD1 | -1.839566367 | 12.72180163 | -3.22237462 | 0.003401586 | 0.011447308 | -2.250871816 |
| TPR | -1.229083699 | 9.451374232 | -3.222370832 | 0.003401618 | 0.011447308 | -2.250880696 |
| DSP | -1.514865685 | 7.652018806 | -3.221241371 | 0.003411181 | 0.011476486 | -2.25352834 |
| ABAT | 1.566508376 | 7.261598167 | 3.221028738 | 0.003412984 | 0.01147955 | -2.254026745 |
| UBOX5 | 1.003173227 | 11.01007269 | 3.220767545 | 0.0034152 | 0.011484002 | -2.254638952 |
| TXNDC12 | -1.063938754 | 6.429982064 | -3.220188597 | 0.003420118 | 0.011495962 | -2.255995868 |
| MRPL17 | -1.177632874 | 5.291556632 | -3.219976829 | 0.003421918 | 0.011495962 | -2.256492177 |
| PDE4B | -1.186418589 | 8.417077304 | -3.219818763 | 0.003423263 | 0.011495962 | -2.256862618 |
| RNF8 | -1.410605376 | 5.810304204 | -3.219806805 | 0.003423364 | 0.011495962 | -2.256890642 |
| LSM6 | -1.0510208 | 9.297337293 | -3.219805332 | 0.003423377 | 0.011495962 | -2.256894095 |
| MT1B | 2.127579133 | 12.83309376 | 3.219717996 | 0.00342412 | 0.011495962 | -2.257098769 |
| COG3 | -1.186366626 | 4.83925947 | -3.217988749 | 0.003438865 | 0.011539442 | -2.261150858 |
| TRAM2 | 1.371384809 | 7.347753137 | 3.217397105 | 0.003443924 | 0.011552472 | -2.262537026 |
| ASGR2 | 1.845920911 | 7.4409912 | 3.215894885 | 0.003456801 | 0.011590555 | -2.266056113 |
| RGPD2 | 1.396912184 | 7.284841895 | 3.214250776 | 0.003470947 | 0.011630213 | -2.269906797 |
| TPRKB | -1.165447342 | 7.477247626 | -3.21420242 | 0.003471364 | 0.011630213 | -2.270020041 |
| OR10H3 | 1.924861476 | 5.46363093 | 3.214100226 | 0.003472245 | 0.011630213 | -2.270259361 |
| TMEM87A | -1.460694199 | 12.25476817 | -3.213602306 | 0.003476542 | 0.011641573 | -2.271425357 |
| CARKD | -1.325338577 | 6.724165587 | -3.213292292 | 0.00347922 | 0.011647507 | -2.27215129 |
| SPTBN1 | -1.100521267 | 10.08166329 | -3.212545922 | 0.003485675 | 0.011666081 | -2.273898877 |
| ACRC | 1.643043264 | 8.441456478 | 3.212411105 | 0.003486842 | 0.011666951 | -2.274214526 |
| FMO5 | -2.034558663 | 6.534060463 | -3.212067484 | 0.003489819 | 0.011673875 | -2.275019026 |
| TMEM86B | 1.126033587 | 7.189321556 | 3.211621388 | 0.003493687 | 0.011681128 | -2.276063391 |
| VPREB3 | 1.808671649 | 8.194677217 | 3.211607943 | 0.003493803 | 0.011681128 | -2.276094866 |
| TEX2 | -1.271384017 | 6.197604263 | -3.21122766 | 0.003497104 | 0.011689125 | -2.276985105 |
| DMAP1 | -1.620045747 | 4.81247281 | -3.208725117 | 0.0035189 | 0.011755869 | -2.282842418 |
| FOXQ1 | 1.71923841 | 12.71281571 | 3.208398404 | 0.003521756 | 0.011759299 | -2.283606962 |
| FDX1 | -1.422902084 | 7.953268514 | -3.207860913 | 0.003526458 | 0.011771944 | -2.28486468 |
| IGBP1 | -1.343251846 | 9.459232861 | -3.207704596 | 0.003527826 | 0.011773457 | -2.28523044 |
| ZMIZ2 | 1.199916593 | 10.34385515 | 3.207374294 | 0.00353072 | 0.011778809 | -2.286003279 |
| CACNA1I | 1.990059981 | 12.88942392 | 3.207312499 | 0.003531262 | 0.011778809 | -2.286147864 |
| TBX10 | 1.829208564 | 6.339078954 | 3.206741539 | 0.00353627 | 0.011789398 | -2.287483703 |
| CYP21A2 | 1.7738996 | 7.006579243 | 3.204973949 | 0.003551817 | 0.011835095 | -2.291618584 |
| MTMR9LP | 1.400576154 | 10.29183346 | 3.203875595 | 0.003561511 | 0.011861251 | -2.294187452 |
| ARHGAP20 | -1.193370996 | 6.640930708 | -3.203200478 | 0.003567482 | 0.011878062 | -2.295766252 |
| RIPK1 | 1.109965346 | 9.546935626 | 3.202856623 | 0.003570527 | 0.011881759 | -2.296570324 |
| VBP1 | -1.02058801 | 6.822125551 | -3.202827072 | 0.003570789 | 0.011881759 | -2.296639425 |
| SHC3 | 1.416325784 | 6.94918534 | 3.202762173 | 0.003571364 | 0.011881759 | -2.296791178 |
| DHX36 | -1.870607644 | 7.58680436 | -3.202022851 | 0.003577921 | 0.011900495 | -2.298519865 |
| LOC554206 | -1.638378484 | 3.828505636 | -3.200942295 | 0.003587525 | 0.011926271 | -2.301046123 |
| MILR1 | 1.284346883 | 7.511038268 | 3.200834405 | 0.003588485 | 0.011926381 | -2.30129834 |
| ATP6 | -1.493908201 | 13.61181559 | -3.200424098 | 0.003592139 | 0.011935442 | -2.302257498 |
| TRIP6 | -1.564487809 | 8.477199615 | -3.200086207 | 0.003595151 | 0.011941945 | -2.303047333 |
| GPC2 | 1.267254671 | 6.407595813 | 3.199996272 | 0.003595953 | 0.011941945 | -2.303257553 |
| PDE1C | -1.309612088 | 4.423808819 | -3.199179113 | 0.003603249 | 0.011963085 | -2.305167529 |
| DLAT | 1.883964207 | 8.345605583 | 3.198652295 | 0.00360796 | 0.011975635 | -2.30639877 |
| CDKN1A | 1.29282474 | 9.376250369 | 3.198180545 | 0.003612184 | 0.011985167 | -2.307501237 |
| ZNF680 | -1.32760964 | 9.7274258 | -3.198123436 | 0.003612696 | 0.011985167 | -2.307634695 |
| B4GALT3 | -1.729246285 | 8.720736846 | -3.1963637 | 0.003628495 | 0.012032688 | -2.311746509 |
| KAZN | 1.011925848 | 9.200722503 | 3.196319728 | 0.003628891 | 0.012032688 | -2.311849242 |
| DHCR24 | -1.793903242 | 8.162397145 | -3.196106129 | 0.003630814 | 0.01203596 | -2.312348271 |
| ITIH4 | 1.392591091 | 9.408234085 | 3.194914253 | 0.003641559 | 0.012062254 | -2.315132574 |
| MPEG1 | 2.205405924 | 9.815553643 | 3.194388082 | 0.003646312 | 0.012068618 | -2.316361606 |
| RSPO1 | -1.74855232 | 6.25451837 | -3.194316243 | 0.003646962 | 0.012068618 | -2.316529401 |
| PDCD5 | -1.153372321 | 9.58653407 | -3.194286192 | 0.003647234 | 0.012068618 | -2.316599591 |
| SLC22A12 | 1.742494748 | 6.869680615 | 3.194159516 | 0.003648379 | 0.012069304 | -2.316895466 |
| SULT4A1 | 2.018571435 | 8.12305312 | 3.193539079 | 0.003653995 | 0.012084773 | -2.318344528 |
| RNGTT | -1.818317398 | 5.004576702 | -3.191146718 | 0.003675727 | 0.012153522 | -2.323930887 |
| FOSL1 | 1.664747139 | 5.780317217 | 3.190037107 | 0.003685849 | 0.012183857 | -2.326521313 |
| HBB | 1.477980925 | 15.47172025 | 3.188961459 | 0.003695686 | 0.012212102 | -2.329032085 |
| ZNF100 | -1.473740186 | 10.67351051 | -3.18875677 | 0.003697561 | 0.012212102 | -2.329509828 |
| LOC389791 | 1.594699957 | 5.952894242 | 3.188687996 | 0.003698191 | 0.012212102 | -2.329670343 |
| PLAGL2 | -1.225002771 | 6.016762959 | -3.187179306 | 0.003712041 | 0.012254631 | -2.333191181 |
| ANKRD33B | 1.60986744 | 6.197896272 | 3.187077632 | 0.003712976 | 0.012254631 | -2.333428431 |
| RAPGEF3 | 2.086775533 | 10.35328807 | 3.186824851 | 0.003715302 | 0.012259162 | -2.334018271 |
| GAL3ST2 | 1.768521708 | 5.758657834 | 3.186103314 | 0.003721949 | 0.012277944 | -2.335701791 |
| AKR7A2 | -1.422244785 | 10.06692434 | -3.18565224 | 0.00372611 | 0.012288519 | -2.336754174 |
| SORBS2 | -2.111464515 | 6.050752201 | -3.185117963 | 0.003731044 | 0.012301638 | -2.338000592 |
| MIXL1 | 2.18186777 | 6.27238632 | 3.18432081 | 0.003738418 | 0.012322792 | -2.339860109 |
| CXCL17 | -2.084602805 | 9.154280213 | -3.18263476 | 0.003754061 | 0.012371184 | -2.343792495 |
| RANBP10 | -1.079485699 | 8.110067059 | -3.182251619 | 0.003757624 | 0.012379755 | -2.344685973 |
| ST6GALNAC6 | -1.428184644 | 7.048271016 | -3.181513296 | 0.0037645 | 0.012399233 | -2.3464076 |
| WBP11 | -1.913869007 | 7.759665224 | -3.181079467 | 0.003768546 | 0.01240552 | -2.347419124 |
| C6orf70 | -1.166533791 | 6.73068513 | -3.1809984 | 0.003769303 | 0.01240552 | -2.347608135 |
| WHSC1L1 | -1.436707441 | 7.675366677 | -3.180818717 | 0.00377098 | 0.012407865 | -2.348027067 |
| EFCAB2 | -1.661374076 | 5.729716958 | -3.18066383 | 0.003772426 | 0.01240945 | -2.348388177 |
| CNOT6 | -1.289577146 | 7.797073751 | -3.178514282 | 0.003792555 | 0.012472472 | -2.353398953 |
| SLC18A2 | -1.223183762 | 4.283896406 | -3.178082716 | 0.003796608 | 0.012482612 | -2.354404794 |
| ZNF469 | 2.260550833 | 8.365396984 | 3.177118386 | 0.003805681 | 0.012509244 | -2.356652122 |
| MBTPS1 | -1.563597744 | 9.318433324 | -3.175784074 | 0.003818269 | 0.012541536 | -2.359761194 |
| DERL3 | 1.801069195 | 8.475416988 | 3.175767006 | 0.003818431 | 0.012541536 | -2.359800962 |
| HNF1A | 1.473101578 | 6.369902465 | 3.175561975 | 0.003820368 | 0.012544605 | -2.360278652 |
| BHLHE23 | 1.374351443 | 6.545460313 | 3.175461792 | 0.003821316 | 0.012544605 | -2.360512058 |
| DCTD | -1.102545851 | 6.727398211 | -3.174223968 | 0.003833038 | 0.012579877 | -2.363395675 |
| MTFP1 | -1.176055368 | 5.190228438 | -3.173295944 | 0.00384185 | 0.012600073 | -2.365557267 |
| NFE2L2 | -2.055430325 | 9.527583019 | -3.173233394 | 0.003842444 | 0.012600073 | -2.365702953 |
| PPFIA1 | -1.509222091 | 7.586168776 | -3.173061013 | 0.003844083 | 0.012600073 | -2.366104435 |
| RBM3 | -1.951619452 | 9.013241827 | -3.172932486 | 0.003845306 | 0.012600846 | -2.366403775 |
| MAFIP | 2.198511593 | 7.761031678 | 3.17076039 | 0.003866023 | 0.012665509 | -2.371461798 |
| SLC6A16 | -1.624341052 | 5.579694838 | -3.170583193 | 0.003867718 | 0.012666816 | -2.371874357 |
| CLIC3 | -1.508419621 | 6.522750312 | -3.170512761 | 0.003868392 | 0.012666816 | -2.372038338 |
| PAG1 | -1.057558159 | 10.70421706 | -3.168289584 | 0.00388972 | 0.012733413 | -2.377213586 |
| SMA5 | -1.34056993 | 4.964705923 | -3.167705133 | 0.003895346 | 0.012746059 | -2.378573846 |
| RPL36 | -1.396667158 | 12.99084517 | -3.167682344 | 0.003895565 | 0.012746059 | -2.378626883 |
| UBE2G2 | 2.168245176 | 12.17950915 | 3.166607566 | 0.003905931 | 0.012770231 | -2.381128045 |
| ABCE1 | -1.043970076 | 7.282499708 | -3.166326445 | 0.003908647 | 0.012775863 | -2.381782192 |
| CPNE6 | 1.916690926 | 8.296914452 | 3.166189707 | 0.003909969 | 0.012776936 | -2.382100364 |
| ATP6V0E1 | -1.373773177 | 10.21761777 | -3.165738711 | 0.003914331 | 0.012787941 | -2.383149727 |
| SLC22A10 | 2.511008256 | 7.794167343 | 3.165397585 | 0.003917634 | 0.01279548 | -2.383943404 |
| MAST4 | -1.038387806 | 7.005834175 | -3.164463772 | 0.003926688 | 0.012821797 | -2.386115861 |
| OSTC | -1.645180398 | 7.976834894 | -3.163401522 | 0.003937012 | 0.012852244 | -2.388586781 |
| CDS1 | -1.337992384 | 7.020761072 | -3.162617537 | 0.003944648 | 0.012870639 | -2.390410195 |
| RC3H2 | -1.025621904 | 6.333875147 | -3.162165668 | 0.003949056 | 0.012881753 | -2.391461073 |
| NME2 | -1.300908891 | 13.06088371 | -3.161980044 | 0.003950868 | 0.012884396 | -2.391892746 |
| ACTR6 | -1.377426163 | 7.075080163 | -3.161767587 | 0.003952943 | 0.012887895 | -2.392386807 |
| SLC6A8 | 1.437638606 | 6.787607856 | 3.161240118 | 0.003958099 | 0.01289872 | -2.393613355 |
| TCP1 | -1.013828703 | 10.05251782 | -3.161160681 | 0.003958876 | 0.01289872 | -2.393798066 |
| SLC26A1 | 1.902176325 | 7.65901634 | 3.161120243 | 0.003959271 | 0.01289872 | -2.393892092 |
| KIAA1009 | -1.028913173 | 5.764117126 | -3.160982284 | 0.003960621 | 0.012899851 | -2.394212876 |
| EXOC3 | -1.274949824 | 6.113596038 | -3.160861913 | 0.0039618 | 0.012900422 | -2.394492756 |
| ZNF700 | -1.496428491 | 7.310213466 | -3.159958511 | 0.003970653 | 0.012925979 | -2.396593161 |
| HADHB | -1.316535342 | 10.68499653 | -3.159312559 | 0.003976996 | 0.012942528 | -2.398094836 |
| IL27RA | 1.273928014 | 9.513604012 | 3.159230097 | 0.003977806 | 0.012942528 | -2.398286529 |
| DKFZp434L192 | 3.30133194 | 10.77537021 | 3.159133483 | 0.003978756 | 0.012942528 | -2.398511119 |
| SLC15A4 | -1.240668342 | 7.637213234 | -3.158531617 | 0.003984676 | 0.01295851 | -2.39991015 |
| KISS1R | 1.603167584 | 7.051076382 | 3.158207685 | 0.003987866 | 0.01296233 | -2.400663078 |
| SORBS3 | -1.034117331 | 9.251548541 | -3.15768687 | 0.003993001 | 0.012975739 | -2.401873561 |
| ZNF493 | -1.283693742 | 8.982728192 | -3.157261328 | 0.0039972 | 0.012986105 | -2.402862543 |
| PGAM1 | -1.534192065 | 8.180996829 | -3.156003156 | 0.004009642 | 0.013023236 | -2.405786264 |
| OR10C1 | 1.503109006 | 5.264475674 | 3.15580359 | 0.004011619 | 0.013026368 | -2.406249964 |
| COX1 | -1.256074767 | 14.81268525 | -3.155693507 | 0.00401271 | 0.013026621 | -2.406505742 |
| MTM1 | -1.268261249 | 5.00200115 | -3.154335151 | 0.004026194 | 0.013067097 | -2.409661564 |
| ZNF784 | 1.616375127 | 6.679030472 | 3.153744731 | 0.004032068 | 0.013082861 | -2.411033082 |
| IL1RAP | 1.229045312 | 7.469154017 | 3.153083124 | 0.004038661 | 0.013100947 | -2.412569829 |
| OBFC2A | 1.584125071 | 9.451484828 | 3.152853663 | 0.00404095 | 0.013105067 | -2.413102777 |
| SOX2 | -1.410033805 | 4.778352889 | -3.15265385 | 0.004042944 | 0.013106081 | -2.413566849 |
| OR4X2 | 1.557260768 | 5.782844227 | 3.152618133 | 0.0040433 | 0.013106081 | -2.413649803 |
| DEFT1P | 1.725243076 | 5.796081503 | 3.151577026 | 0.004053707 | 0.013136502 | -2.416067593 |
| LCN12 | 1.858485787 | 5.673273221 | 3.148620377 | 0.004083401 | 0.013224693 | -2.422931995 |
| GBP3 | 1.839334575 | 12.15729873 | 3.148604267 | 0.004083563 | 0.013224693 | -2.42296939 |
| HCCS | -1.125957128 | 6.017772542 | -3.148458344 | 0.004085034 | 0.013224693 | -2.423308103 |
| KRT15 | 1.582466275 | 9.805102142 | 3.147335864 | 0.004096366 | 0.013254707 | -2.425913348 |
| NSRP1 | -1.13497798 | 7.603182622 | -3.145845538 | 0.004111458 | 0.013298824 | -2.429371724 |
| PER3 | 2.216937483 | 8.649115231 | 3.1457478 | 0.00411245 | 0.013298824 | -2.429598504 |
| ODAM | -2.049548749 | 5.256255493 | -3.145683518 | 0.004113102 | 0.013298824 | -2.429747656 |
| STRADA | -1.682283846 | 5.723135698 | -3.145079073 | 0.004119241 | 0.013315325 | -2.431150065 |
| C6orf192 | 1.226925274 | 7.787132353 | 3.144736834 | 0.004122721 | 0.013323224 | -2.431944062 |
| WASH2P | 1.346602748 | 11.31943468 | 3.144514125 | 0.004124986 | 0.013327198 | -2.432460727 |
| HSD17B11 | -1.381305475 | 11.02976003 | -3.14433562 | 0.004126803 | 0.01332972 | -2.432874832 |
| FTSJ1 | 1.820874626 | 7.424743241 | 3.144088177 | 0.004129323 | 0.013331888 | -2.433448846 |
| MGC27345 | -1.061859759 | 5.796665087 | -3.143953894 | 0.004130691 | 0.013331888 | -2.433760345 |
| ACRBP | -1.215552317 | 5.102090079 | -3.143941005 | 0.004130823 | 0.013331888 | -2.433790243 |
| PCIF1 | 1.390908014 | 8.360765682 | 3.143862704 | 0.004131621 | 0.013331888 | -2.433971877 |
| ENKUR | -1.929119935 | 4.8987285 | -3.143680499 | 0.004133478 | 0.013334537 | -2.434394525 |
| HIF3A | 1.608473935 | 9.933306263 | 3.14223797 | 0.004148213 | 0.013374881 | -2.43774029 |
| FAM134C | -1.515862465 | 5.724688324 | -3.14215087 | 0.004149104 | 0.013374881 | -2.437942287 |
| INTS3 | -1.431373737 | 6.170501405 | -3.141903604 | 0.004151635 | 0.01337703 | -2.438515715 |
| SARDH | 1.04468933 | 6.943366232 | 3.141825795 | 0.004152432 | 0.01337703 | -2.438696156 |
| PRSS2 | 1.975348589 | 6.502601176 | 3.141758623 | 0.00415312 | 0.01337703 | -2.438851927 |
| APOA5 | 1.642195129 | 6.528930927 | 3.141679472 | 0.004153931 | 0.01337703 | -2.439035475 |
| FADS2 | 1.56628409 | 10.60895153 | 3.141357123 | 0.004157235 | 0.013384319 | -2.439782973 |
| KCNH4 | 2.988392477 | 8.632181966 | 3.139192367 | 0.004179487 | 0.01343914 | -2.444801967 |
| ENGASE | 1.270188569 | 9.032541465 | 3.138749875 | 0.00418405 | 0.013450449 | -2.445827699 |
| JAZF1 | -1.428966137 | 5.758285271 | -3.138346142 | 0.004188217 | 0.013457489 | -2.446763529 |
| UQCRC1 | -1.12252528 | 7.205663798 | -3.138334944 | 0.004188332 | 0.013457489 | -2.446789485 |
| XPNPEP2 | 1.722833573 | 6.07778242 | 3.136829968 | 0.004203901 | 0.01350414 | -2.450277459 |
| HP07349 | 1.263708114 | 6.979765216 | 3.135352665 | 0.004219238 | 0.01354664 | -2.453700579 |
| HSPB2 | 1.401445505 | 10.29403986 | 3.134479441 | 0.004228329 | 0.013564274 | -2.455723628 |
| FNDC3A | -1.208617139 | 9.222589304 | -3.134472473 | 0.004228401 | 0.013564274 | -2.45573977 |
| DNAH11 | -1.067327039 | 5.629027216 | -3.134313559 | 0.004230058 | 0.013564448 | -2.456107909 |
| OSBPL3 | 1.204725191 | 9.155303815 | 3.134148511 | 0.004231779 | 0.013566584 | -2.456490247 |
| CCDC153 | -1.755840317 | 6.129306144 | -3.133246127 | 0.0042412 | 0.0135934 | -2.458580494 |
| ADH1A | -1.960297924 | 8.771513776 | -3.132454931 | 0.004249477 | 0.013616535 | -2.460412971 |
| CSF2RB | 1.536431521 | 6.607580132 | 3.130640353 | 0.004268518 | 0.013674143 | -2.464614913 |
| FKBP8 | 1.049961952 | 6.695940058 | 3.130217611 | 0.004272966 | 0.013684983 | -2.465593686 |
| ISCU | -1.142791129 | 12.24219318 | -3.130044092 | 0.004274793 | 0.013687426 | -2.465995415 |
| RADIL | -2.168039376 | 9.033081242 | -3.129894757 | 0.004276366 | 0.013689055 | -2.466341147 |
| TTLL5 | 1.320029613 | 6.737178349 | 3.129399346 | 0.004281588 | 0.013702361 | -2.467488041 |
| SPATA5 | -1.249433612 | 4.900298427 | -3.128325329 | 0.00429293 | 0.013731825 | -2.469974154 |
| RNF215 | 1.141187781 | 6.512134139 | 3.127903988 | 0.004297388 | 0.013742665 | -2.470949363 |
| NICN1 | -1.351301977 | 7.324661633 | -3.127399242 | 0.004302733 | 0.013755449 | -2.472117538 |
| HIST2H2BE | 1.035149137 | 9.052839383 | 3.127290808 | 0.004303882 | 0.013755449 | -2.472368483 |
| BRE | -1.122695729 | 5.709397404 | -3.126178297 | 0.00431569 | 0.013785546 | -2.474942925 |
| DNAJC3 | -1.369989802 | 9.007457143 | -3.126134503 | 0.004316156 | 0.013785546 | -2.475044258 |
| MAPK8IP3 | 1.078806837 | 5.626555073 | 3.12460251 | 0.004332468 | 0.013830162 | -2.478588727 |
| MUC12 | 1.703861523 | 5.64509619 | 3.12451981 | 0.00433335 | 0.013830162 | -2.478780043 |
| SPDYE1 | 2.464678829 | 8.307261822 | 3.123399734 | 0.004345317 | 0.013864912 | -2.481370969 |
| TMEM239 | 1.884398287 | 7.117771373 | 3.123161452 | 0.004347866 | 0.013869607 | -2.481922102 |
| STAM2 | -1.160332241 | 7.424625764 | -3.122697856 | 0.004352831 | 0.013882002 | -2.48299432 |
| FAM179A | 1.639597371 | 6.499700825 | 3.122482349 | 0.004355141 | 0.013885925 | -2.483492728 |
| S100A4 | -1.462528397 | 12.27962462 | -3.121932361 | 0.004361041 | 0.013901291 | -2.484764626 |
| LOC541472 | 2.153119573 | 13.10072472 | 3.119427265 | 0.004388012 | 0.013976872 | -2.49055663 |
| COPS8 | -1.017084494 | 5.573252841 | -3.118421776 | 0.004398882 | 0.014008027 | -2.492880828 |
| NCAPH | 1.547434475 | 6.233440821 | 3.117285826 | 0.004411194 | 0.014043757 | -2.495506186 |
| PNRC1 | -1.348445392 | 11.94530701 | -3.116329612 | 0.004421583 | 0.014073349 | -2.497715813 |
| TAGLN2 | -1.073922921 | 9.577726632 | -3.11529481 | 0.004432853 | 0.014104679 | -2.500106704 |
| DYNC1I2 | -1.07973473 | 9.149551881 | -3.115180082 | 0.004434104 | 0.014104679 | -2.500371758 |
| COL18A1 | 1.721555445 | 12.16547542 | 3.114948145 | 0.004436635 | 0.014107291 | -2.500907586 |
| FAM118B | -1.444741807 | 5.227435378 | -3.114794043 | 0.004438317 | 0.014108301 | -2.501263589 |
| PLCE1 | -1.500696779 | 7.112016381 | -3.114718093 | 0.004439146 | 0.014108301 | -2.501439043 |
| MYO9A | -1.404815982 | 8.877344961 | -3.11355294 | 0.004451885 | 0.014138308 | -2.504130459 |
| GNAO1 | 1.626309781 | 5.729460927 | 3.113367602 | 0.004453915 | 0.014141263 | -2.504558535 |
| TTC22 | 2.736452748 | 7.680742682 | 3.112887654 | 0.004459175 | 0.014154469 | -2.505667018 |
| INA | 1.155070073 | 5.108382475 | 3.112734248 | 0.004460858 | 0.014156317 | -2.506021307 |
| DCLRE1A | -1.184683065 | 6.482783654 | -3.111135882 | 0.004478425 | 0.014205055 | -2.509712247 |
| C1orf54 | 1.274912571 | 11.47159281 | 3.110902487 | 0.004480995 | 0.014209705 | -2.51025113 |
| CNIH | -1.193852025 | 10.12005794 | -3.110792535 | 0.004482207 | 0.014210043 | -2.51050499 |
| C15orf62 | 2.227544666 | 8.55093686 | 3.110095905 | 0.00448989 | 0.014230894 | -2.512113299 |
| TMEM69 | -1.274229855 | 6.439291669 | -3.107803508 | 0.004515263 | 0.014304264 | -2.517404617 |
| NUDT9 | -1.406692189 | 9.058723357 | -3.107498012 | 0.004518655 | 0.014311484 | -2.518109632 |
| COL13A1 | -1.137243622 | 8.25636586 | -3.106935366 | 0.004524907 | 0.01432776 | -2.519408007 |
| GPX4 | -1.527533081 | 9.261465474 | -3.105991849 | 0.004535412 | 0.014355187 | -2.521585055 |
| GNG13 | 2.787539774 | 7.534716791 | 3.105956882 | 0.004535802 | 0.014355187 | -2.521665731 |
| ATP6V1H | -1.654977044 | 9.341224299 | -3.105441698 | 0.004541547 | 0.014367475 | -2.522854323 |
| AKIP1 | 1.107145888 | 9.931567179 | 3.104825214 | 0.004548432 | 0.014384543 | -2.524276507 |
| CNTN1 | 2.156125237 | 10.04984169 | 3.103192499 | 0.004566715 | 0.014438812 | -2.528042455 |
| HDAC10 | 1.233768472 | 10.14687027 | 3.102726906 | 0.004571941 | 0.014448234 | -2.529116208 |
| MRPL27 | -1.275641422 | 7.740078358 | -3.102241496 | 0.004577396 | 0.014461919 | -2.530235586 |
| PPCS | -1.299454807 | 9.033653182 | -3.101698587 | 0.004583505 | 0.014472035 | -2.531487465 |
| LAMA4 | -1.268757019 | 9.729724424 | -3.101656937 | 0.004583974 | 0.014472035 | -2.531583502 |
| GPD1 | 1.776583111 | 8.968884236 | 3.101518538 | 0.004585532 | 0.014473403 | -2.531902615 |
| MTRF1L | 1.760183935 | 8.397124155 | 3.099727716 | 0.004605746 | 0.014533637 | -2.536031217 |
| CLCN1 | 1.752816775 | 7.028563295 | 3.099279944 | 0.004610813 | 0.014546058 | -2.537063352 |
| MB | 1.66255488 | 6.549815732 | 3.098117606 | 0.004623992 | 0.014580483 | -2.539742284 |
| PXMP2 | -1.395590159 | 6.439066023 | -3.097252397 | 0.004633826 | 0.014607909 | -2.541736105 |
| ACCS | -2.169554594 | 5.924605022 | -3.096806201 | 0.004638905 | 0.014620337 | -2.542764236 |
| OR4A15 | 1.394309741 | 6.326130902 | 3.096497013 | 0.004642427 | 0.01462671 | -2.543476634 |
| PNMA2 | 1.279425395 | 4.878793419 | 3.096429104 | 0.004643202 | 0.01462671 | -2.543633099 |
| SMNDC1 | -1.062538074 | 8.658235074 | -3.095483741 | 0.00465399 | 0.014650232 | -2.54581108 |
| GTF2H5 | -1.077331241 | 9.219842768 | -3.095472659 | 0.004654116 | 0.014650232 | -2.54583661 |
| FOXE3 | 1.609902285 | 6.50918638 | 3.095403714 | 0.004654904 | 0.014650232 | -2.545995438 |
| ELOVL1 | 1.568231867 | 10.92907855 | 3.095375669 | 0.004655225 | 0.014650232 | -2.546060045 |
| ABCA3 | -2.206466739 | 9.096364079 | -3.09256526 | 0.004687446 | 0.014748027 | -2.552532948 |
| PTPN4 | -1.298984191 | 6.840796169 | -3.09155584 | 0.004699071 | 0.014780987 | -2.554857184 |
| C1R | 1.494291164 | 12.36147129 | 3.089464704 | 0.004723242 | 0.01485289 | -2.559671029 |
| BRI3 | -1.10163662 | 13.17899623 | -3.08937863 | 0.00472424 | 0.01485289 | -2.559869142 |
| THBS3 | 2.149183947 | 10.81714392 | 3.089050226 | 0.004728047 | 0.014861228 | -2.560624993 |
| HNRNPM | -1.39092053 | 11.41792444 | -3.088880433 | 0.004730017 | 0.014863786 | -2.561015772 |
| ZRANB2 | -1.590767106 | 7.777427936 | -3.088641126 | 0.004732794 | 0.014868882 | -2.561566519 |
| EMD | -1.243592283 | 5.941429023 | -3.087739309 | 0.004743275 | 0.014894646 | -2.563641818 |
| SYN1 | 1.537485697 | 7.100077469 | 3.087736192 | 0.004743311 | 0.014894646 | -2.56364899 |
| PMS2L2 | 1.388129097 | 8.016124936 | 3.087346679 | 0.004747845 | 0.014905244 | -2.564545266 |
| ATIC | -1.502482509 | 5.528079221 | -3.087029605 | 0.004751539 | 0.0149132 | -2.565274822 |
| LOC100507205 | 1.661590114 | 6.732991995 | 3.086840252 | 0.004753746 | 0.014916487 | -2.565710489 |
| GGA3 | -1.450424444 | 7.125553838 | -3.085270443 | 0.004772082 | 0.01496307 | -2.569321862 |
| SEC22A | -1.066465822 | 6.437471819 | -3.084289803 | 0.00478357 | 0.014991783 | -2.571577418 |
| CHN1 | -1.60038954 | 8.202499722 | -3.084091026 | 0.004785902 | 0.014995438 | -2.572034583 |
| MADCAM1 | 1.520413373 | 6.738079861 | 3.083918843 | 0.004787923 | 0.014997658 | -2.572430575 |
| CCDC125 | 1.062100375 | 9.78107096 | 3.083831972 | 0.004788943 | 0.014997658 | -2.572630358 |
| P2RY14 | -1.30838904 | 6.911380386 | -3.083233416 | 0.004795975 | 0.015016026 | -2.574006834 |
| C4B | -1.094028782 | 8.588723733 | -3.082817834 | 0.004800864 | 0.015027675 | -2.574962461 |
| LOC100505894 | -1.583828108 | 4.946649802 | -3.081631004 | 0.004814852 | 0.015064126 | -2.577691243 |
| XAGE2B | -1.869521486 | 6.140455561 | -3.080802861 | 0.004824635 | 0.015091064 | -2.579595041 |
| PTPLAD1 | -1.264070738 | 9.9697271 | -3.0774672 | 0.004864234 | 0.015211227 | -2.587260952 |
| SMR3A | 1.902782976 | 6.19604625 | 3.075875031 | 0.004883245 | 0.015263255 | -2.590918688 |
| EPHA10 | 1.694158766 | 5.771349093 | 3.074377633 | 0.004901189 | 0.015315619 | -2.594357916 |
| CSTF1 | -1.366426763 | 7.421976511 | -3.074208265 | 0.004903222 | 0.015318252 | -2.594746873 |
| SPRR1B | 2.724121848 | 6.816990435 | 3.073455037 | 0.004912276 | 0.01534281 | -2.596476555 |
| DUSP10 | -1.351256309 | 4.800653259 | -3.073206448 | 0.004915267 | 0.015348427 | -2.597047363 |
| FMR1NB | 2.559209955 | 5.615933591 | 3.0727827 | 0.004920371 | 0.015360633 | -2.59802032 |
| DKFZP564C196 | 2.217806858 | 8.010900192 | 3.072392565 | 0.004925074 | 0.015371584 | -2.598916044 |
| STXBP2 | 1.172520926 | 6.313096869 | 3.072267425 | 0.004926583 | 0.015372565 | -2.599203347 |
| CHST7 | -1.426590129 | 5.937416749 | -3.072146949 | 0.004928037 | 0.015373371 | -2.599479935 |
| FAIM2 | 1.795860586 | 6.682511048 | 3.071549444 | 0.004935252 | 0.015388415 | -2.600851616 |
| PRDM16 | -1.640084779 | 7.558809111 | -3.071240196 | 0.004938991 | 0.015395489 | -2.601561502 |
| RPS6KC1 | -1.36847244 | 6.779896135 | -3.071163786 | 0.004939915 | 0.015395489 | -2.6017369 |
| CDO1 | -1.303334017 | 7.030828062 | -3.070958238 | 0.004942401 | 0.015399508 | -2.602208717 |
| HLA-DQA1 | -4.17081478 | 8.337051817 | -3.069726646 | 0.004957326 | 0.01544227 | -2.605035424 |
| MGC39372 | 1.911521426 | 5.965295821 | 3.069467127 | 0.004960477 | 0.015448342 | -2.605630999 |
| DSCR3 | -1.173463624 | 7.272658273 | -3.068786254 | 0.004968752 | 0.015466622 | -2.607193432 |
| WWP1 | -1.636352331 | 8.600751811 | -3.068143652 | 0.004976573 | 0.015487221 | -2.608667896 |
| PRKACA | 1.027586872 | 7.069023696 | 3.067510484 | 0.004984291 | 0.015507488 | -2.610120575 |
| EVL | -1.013286887 | 8.896736423 | -3.066854847 | 0.004992296 | 0.015528636 | -2.611624662 |
| WISP2 | -1.677241558 | 11.5588063 | -3.066627229 | 0.004995077 | 0.015529777 | -2.6121468 |
| NTN3 | 1.458227404 | 5.035466082 | 3.066456094 | 0.00499717 | 0.015532528 | -2.61253936 |
| RAP1A | -1.178827467 | 8.358112058 | -3.066118746 | 0.005001297 | 0.0155416 | -2.613313162 |
| C2orf49 | -1.896298441 | 4.387155815 | -3.065716101 | 0.005006227 | 0.015553163 | -2.614236688 |
| LOC100505540 | 1.879622844 | 6.536287845 | 3.06496517 | 0.005015435 | 0.015574244 | -2.615958909 |
| FAM129B | 1.299972068 | 13.60514023 | 3.064033879 | 0.005026876 | 0.015602238 | -2.618094508 |
| PHYH | -1.315589708 | 7.159477959 | -3.062075487 | 0.005051017 | 0.015673384 | -2.622584442 |
| TNS4 | 2.689378851 | 8.177154329 | 3.061713161 | 0.005055495 | 0.015683497 | -2.623414987 |
| SLC37A4 | 1.474310867 | 8.731507781 | 3.06157465 | 0.005057208 | 0.015685028 | -2.62373248 |
| GMPR | -1.480241609 | 4.421227023 | -3.06075576 | 0.005067347 | 0.015708898 | -2.625609385 |
| LOC100506710 | -1.44143173 | 7.761623098 | -3.059852113 | 0.005078558 | 0.015739859 | -2.627680287 |
| PJA2 | -1.948191193 | 8.660102356 | -3.05585529 | 0.005128429 | 0.015882942 | -2.636836478 |
| CAGE1 | 2.075238663 | 5.606346407 | 3.05303889 | 0.005163852 | 0.015981104 | -2.643285157 |
| U2AF1L4 | -1.321004316 | 5.441837166 | -3.05266658 | 0.005168553 | 0.015991802 | -2.644137425 |
| PPAP2A | -1.702747915 | 9.167679746 | -3.050192813 | 0.005199886 | 0.016073284 | -2.64979899 |
| MET | -1.616764253 | 8.794850059 | -3.049798723 | 0.005204894 | 0.0160849 | -2.650700724 |
| COL6A1 | 1.798328554 | 13.57660864 | 3.049110995 | 0.005213646 | 0.016108074 | -2.652274213 |
| BIRC3 | 1.684295745 | 11.03562515 | 3.047036566 | 0.005240128 | 0.016185148 | -2.657019417 |
| ACOT4 | -1.453660564 | 5.16965965 | -3.046495679 | 0.005247054 | 0.016199617 | -2.658256434 |
| AP1M2 | -1.180518095 | 5.354090049 | -3.046232219 | 0.00525043 | 0.016206152 | -2.658858935 |
| FLJ23867 | 1.277846227 | 8.158851549 | 3.045986977 | 0.005253575 | 0.016211969 | -2.659419752 |
| C5orf58 | 2.249043176 | 7.616898339 | 3.045862966 | 0.005255167 | 0.016212989 | -2.659703331 |
| OR6Y1 | 2.254621799 | 6.059331479 | 3.045041164 | 0.005265722 | 0.016241658 | -2.66158243 |
| LDB1 | 1.000644324 | 7.405445821 | 3.044229952 | 0.005276161 | 0.016267093 | -2.663437085 |
| KIAA0319L | -1.276714933 | 5.549700741 | -3.044105675 | 0.005277762 | 0.016267093 | -2.663721196 |
| APOBR | -1.716689723 | 5.625242094 | -3.043150259 | 0.005290086 | 0.016297266 | -2.665905203 |
| FOXN2 | 1.302305462 | 10.64122665 | 3.041135793 | 0.005316161 | 0.016369753 | -2.67050907 |
| C1orf172 | -1.393356271 | 6.342316834 | -3.040984824 | 0.00531812 | 0.016371866 | -2.670854039 |
| SLAMF8 | 2.058397889 | 10.8347063 | 3.040298157 | 0.005327039 | 0.016391477 | -2.672422988 |
| CD68 | 1.479730734 | 11.22654043 | 3.040014208 | 0.005330731 | 0.016398916 | -2.673071729 |
| ZBTB48 | -1.151718259 | 5.238730401 | -3.039320791 | 0.005339759 | 0.016422758 | -2.674655867 |
| FLJ36116 | 1.478867069 | 5.248451911 | 3.038917257 | 0.005345019 | 0.01643125 | -2.675577677 |
| SCT | 1.60895869 | 6.630800493 | 3.038904638 | 0.005345183 | 0.01643125 | -2.675606503 |
| HIST1H2BC | 1.407790487 | 8.499980129 | 3.038717105 | 0.00534763 | 0.01643125 | -2.676034872 |
| KCTD7 | 1.120966413 | 6.447939581 | 3.038103704 | 0.005355639 | 0.016451929 | -2.677435939 |
| SCARA3 | 1.119800616 | 8.656441769 | 3.036588107 | 0.005375477 | 0.016497108 | -2.680897141 |
| MYLK2 | 1.172925987 | 5.423150051 | 3.036291656 | 0.005379365 | 0.016505104 | -2.681574059 |
| SBF1 | -1.248530999 | 5.161664816 | -3.036096418 | 0.005381928 | 0.01650816 | -2.682019849 |
| KLK6 | 1.877120818 | 5.672438661 | 3.036020186 | 0.005382928 | 0.01650816 | -2.682193907 |
| ZNF213 | 1.441207838 | 7.729979124 | 3.03545887 | 0.005390303 | 0.016526836 | -2.683475482 |
| IRF2BPL | 1.559155539 | 7.218304882 | 3.034845728 | 0.00539837 | 0.016547624 | -2.684875256 |
| MYL12B | -1.049830896 | 12.19824501 | -3.034235844 | 0.005406405 | 0.016568306 | -2.686267459 |
| SDR39U1 | -1.203804245 | 6.832479004 | -3.033901242 | 0.005410818 | 0.016577881 | -2.687031213 |
| N6AMT2 | -1.226881127 | 6.675911786 | -3.033207906 | 0.005419974 | 0.016601978 | -2.688613675 |
| FLJ35946 | 1.510362635 | 5.448914855 | 3.031753283 | 0.005439231 | 0.016656996 | -2.691933143 |
| LOC100329109 | -1.056403513 | 7.314277842 | -3.031137424 | 0.005447403 | 0.016678053 | -2.693338315 |
| ATP8A1 | -1.222947354 | 6.256399886 | -3.030413183 | 0.005457029 | 0.016703548 | -2.694990605 |
| SON | -1.140169323 | 8.856027417 | -3.029633328 | 0.005467412 | 0.016731348 | -2.696769567 |
| GRK6 | 1.058768632 | 7.809218169 | 3.029254683 | 0.00547246 | 0.016742813 | -2.697633235 |
| MSL3 | -1.010320384 | 6.609469876 | -3.029053491 | 0.005475145 | 0.01674306 | -2.698092122 |
| SNX20 | 1.208606856 | 8.956041184 | 3.028479929 | 0.005482804 | 0.016762496 | -2.699400245 |
| MYOZ2 | 2.880913735 | 6.070370127 | 3.028317546 | 0.005484974 | 0.016765134 | -2.699770572 |
| NPEPL1 | 1.006094946 | 10.25555192 | 3.028220328 | 0.005486273 | 0.016765134 | -2.69999228 |
| POLD4 | 1.128648606 | 12.26151911 | 3.027521292 | 0.005495627 | 0.016782392 | -2.701586351 |
| ALG14 | -1.314048281 | 6.438787398 | -3.027408333 | 0.00549714 | 0.016782392 | -2.701843924 |
| STMN2 | 1.515446519 | 6.203231169 | 3.026338847 | 0.005511484 | 0.0168182 | -2.704282385 |
| C16orf74 | 1.022364734 | 5.909406605 | 3.02597415 | 0.005516384 | 0.016829158 | -2.705113814 |
| TYSND1 | -1.079220502 | 4.304169955 | -3.025862703 | 0.005517882 | 0.016829736 | -2.705367881 |
| AACS | -1.412557431 | 6.159812579 | -3.024927166 | 0.005530473 | 0.016864139 | -2.707500445 |
| SLC36A4 | 1.031093883 | 8.103575681 | 3.024771482 | 0.005532571 | 0.016866538 | -2.707855297 |
| SEMA4C | 1.118603108 | 11.06654859 | 3.024428779 | 0.005537191 | 0.016876625 | -2.708636396 |
| LUC7L | 1.354778326 | 10.10925703 | 3.023592982 | 0.005548476 | 0.016907012 | -2.710541193 |
| EZH2 | 1.663600676 | 8.725585809 | 3.022752914 | 0.005559841 | 0.016937629 | -2.712455474 |
| LRRC36 | -2.20363909 | 7.989054125 | -3.022479933 | 0.005563538 | 0.01694488 | -2.713077466 |
| TSPYL2 | 1.880487942 | 13.04612351 | 3.022001791 | 0.005570021 | 0.016960607 | -2.714166862 |
| PREX2 | 1.27030617 | 6.907230881 | 3.021547066 | 0.005576192 | 0.016975381 | -2.715202828 |
| PGP | 1.556251044 | 7.592483506 | 3.021120178 | 0.005581992 | 0.016984996 | -2.716175306 |
| METTL3 | -1.711464546 | 6.382975665 | -3.020069274 | 0.005596294 | 0.017024487 | -2.718569062 |
| SEC24B | -1.185247651 | 6.690088999 | -3.018938293 | 0.005611725 | 0.017067393 | -2.721144778 |
| HIST1H2BD | 1.046043002 | 8.613670435 | 3.017954672 | 0.005625178 | 0.017104265 | -2.723384526 |
| SNPH | 2.052814916 | 7.594920244 | 3.017593278 | 0.005630129 | 0.017115273 | -2.724207347 |
| PPP4C | -1.175505276 | 6.603412265 | -3.016243773 | 0.005648652 | 0.017167525 | -2.727279493 |
| PSTPIP1 | 1.302988441 | 8.176155904 | 3.015683194 | 0.005656364 | 0.017183836 | -2.72855546 |
| MARC1 | -1.263572678 | 4.984431802 | -3.015659419 | 0.005656691 | 0.017183836 | -2.728609572 |
| CD37 | -1.235482292 | 7.067362019 | -3.014504911 | 0.005672606 | 0.017224045 | -2.73123705 |
| ZDHHC3 | -1.239570335 | 6.940452844 | -3.013786215 | 0.005682534 | 0.017249537 | -2.73287245 |
| AKNA | 1.265885391 | 6.471791685 | 3.013307924 | 0.005689151 | 0.017262057 | -2.733960703 |
| FUCA2 | -1.27274503 | 7.868073174 | -3.012204749 | 0.00570444 | 0.017304365 | -2.736470438 |
| OR1S2 | 2.569296892 | 7.063877069 | 3.012099491 | 0.005705901 | 0.017304715 | -2.73670988 |
| C9orf150 | -2.251562027 | 6.895488959 | -3.01191044 | 0.005708526 | 0.017308594 | -2.737139922 |
| KIR2DL4 | 1.491228573 | 6.075451308 | 3.011262796 | 0.005717526 | 0.017331798 | -2.738613049 |
| S100A2 | 3.325481161 | 9.416391055 | 3.010956265 | 0.00572179 | 0.017340638 | -2.739310231 |
| JPH2 | -1.391675284 | 4.608444849 | -3.010586797 | 0.005726935 | 0.01735214 | -2.740150513 |
| TSPYL5 | -1.517838939 | 8.241947838 | -3.010202721 | 0.005732287 | 0.017361336 | -2.741023967 |
| CCT6P1 | -1.204730205 | 5.242133802 | -3.010172565 | 0.005732707 | 0.017361336 | -2.741092543 |
| C1orf88 | -1.643118346 | 5.911959347 | -3.010078474 | 0.005734019 | 0.017361336 | -2.741306513 |
| SLC4A1 | 1.376297221 | 5.364581615 | 3.008498381 | 0.005756095 | 0.017424075 | -2.744899283 |
| VCL | -1.042128997 | 9.077797877 | -3.008082182 | 0.005761923 | 0.017437614 | -2.745845476 |
| ZNF398 | -1.827718801 | 7.598303843 | -3.005572262 | 0.005797191 | 0.017536093 | -2.751550244 |
| NPL | 1.297855626 | 8.925003493 | 3.005203733 | 0.005802386 | 0.017547682 | -2.752387678 |
| CYP4A11 | 2.230583493 | 6.994359032 | 3.004611161 | 0.00581075 | 0.017568844 | -2.75373412 |
| CCL14 | -2.059082483 | 9.218422527 | -3.004140357 | 0.005817403 | 0.017579487 | -2.75480379 |
| C17orf105 | 2.401241238 | 6.075200493 | 3.00413555 | 0.005817471 | 0.017579487 | -2.754814711 |
| ZFP2 | -1.021866284 | 5.089646849 | -3.004071945 | 0.00581837 | 0.017579487 | -2.754959217 |
| APLP2 | -1.402178047 | 11.15132975 | -3.001148456 | 0.005859852 | 0.017700661 | -2.761599548 |
| OPTN | 1.11315439 | 12.61381743 | 3.000818884 | 0.005864546 | 0.017710681 | -2.762347936 |
| AUH | -1.506992118 | 7.448759927 | -3.000574437 | 0.00586803 | 0.017717042 | -2.762902998 |
| FYN | -1.147022327 | 10.00228446 | -2.999219508 | 0.005887376 | 0.017771283 | -2.76597922 |
| GOLGA6L9 | 1.307584127 | 10.77867882 | 2.999040678 | 0.005889934 | 0.017774832 | -2.766385187 |
| PPT1 | -1.487353566 | 10.38149862 | -2.998327222 | 0.00590015 | 0.017801485 | -2.7680047 |
| C17orf75 | -1.17461457 | 6.659799674 | -2.998129013 | 0.005902991 | 0.01780588 | -2.768454594 |
| SPECC1L | -1.640957844 | 8.309825838 | -2.997879277 | 0.005906573 | 0.017810094 | -2.769021423 |
| ECHDC3 | -1.107482124 | 8.469636825 | -2.996581551 | 0.005925217 | 0.01785199 | -2.771966523 |
| MAMSTR | 1.564887459 | 5.899806422 | 2.99612499 | 0.00593179 | 0.017863424 | -2.773002512 |
| CCDC28A | -1.051459127 | 9.746080669 | -2.994447372 | 0.005956001 | 0.017923745 | -2.776808573 |
| CDH13 | -2.059388778 | 7.013689358 | -2.994153437 | 0.005960253 | 0.01792815 | -2.777475326 |
| ACBD4 | 1.555743356 | 6.99333942 | 2.993550816 | 0.005968979 | 0.017950199 | -2.778842196 |
| RAI1 | -1.219942307 | 7.702155032 | -2.993221403 | 0.005973753 | 0.017960359 | -2.779589318 |
| ELMO3 | -1.224560464 | 5.258592793 | -2.992561383 | 0.005983332 | 0.017984953 | -2.781086151 |
| LRRC49 | -1.092746258 | 6.440707408 | -2.992096508 | 0.005990087 | 0.017999769 | -2.782140329 |
| CRYM | 1.350258074 | 8.986974987 | 2.992029574 | 0.00599106 | 0.017999769 | -2.782292106 |
| HSD17B2 | -1.739200251 | 4.647757607 | -2.991531074 | 0.005998313 | 0.018017351 | -2.783422433 |
| CORO1C | -1.257548846 | 8.330926965 | -2.991027993 | 0.006005641 | 0.018033287 | -2.784563057 |
| ATF6B | 1.377833197 | 8.040144697 | 2.990881444 | 0.006007777 | 0.018033287 | -2.784895306 |
| TBCCD1 | -1.007690085 | 5.417703973 | -2.990723101 | 0.006010086 | 0.018035867 | -2.785254287 |
| ZNF574 | 1.156347965 | 8.077379298 | 2.990521726 | 0.006013024 | 0.018040474 | -2.785710812 |
| C3orf22 | 1.551401042 | 6.081315661 | 2.989798374 | 0.006023587 | 0.018059529 | -2.787350557 |
| TUBGCP6 | -1.481764384 | 5.29983253 | -2.989698198 | 0.006025052 | 0.01805971 | -2.787577628 |
| ACCN1 | 2.389233903 | 9.331305047 | 2.988965859 | 0.006035767 | 0.018085733 | -2.789237523 |
| GPHB5 | 1.521862493 | 6.057303986 | 2.9889127 | 0.006036546 | 0.018085733 | -2.789358005 |
| DNAJA3 | 2.273054523 | 11.92881614 | 2.988813264 | 0.006038002 | 0.018085884 | -2.789583365 |
| LMOD2 | 1.703803018 | 6.611811884 | 2.98757973 | 0.006056099 | 0.018128742 | -2.792378746 |
| FRMD1 | 2.549394203 | 7.769468281 | 2.987552139 | 0.006056504 | 0.018128742 | -2.792441266 |
| SIGLEC8 | 1.496365948 | 6.589917579 | 2.987549768 | 0.006056539 | 0.018128742 | -2.792446639 |
| KBTBD3 | -1.732540022 | 5.095153549 | -2.987101434 | 0.006063129 | 0.018144246 | -2.79346249 |
| PRKAR1B | 1.480930468 | 7.04098946 | 2.986112016 | 0.006077698 | 0.018183612 | -2.795704093 |
| CKS2 | 1.045880477 | 10.27334169 | 2.984383562 | 0.006103228 | 0.018251505 | -2.799619183 |
| ATG16L2 | 1.857951542 | 11.91829271 | 2.981630831 | 0.006144099 | 0.018369456 | -2.805852093 |
| DNASE1L1 | -1.838972649 | 6.578929717 | -2.981533079 | 0.006145555 | 0.018369539 | -2.80607338 |
| VSIG1 | 2.224181556 | 6.596657072 | 2.981390246 | 0.006147683 | 0.018371631 | -2.80639671 |
| IFT172 | 1.075769956 | 12.06037653 | 2.978696599 | 0.006187951 | 0.018483378 | -2.812492919 |
| PDCD2L | -1.374086974 | 6.64984906 | -2.978260064 | 0.006194501 | 0.018495336 | -2.813480628 |
| RPIA | -1.025982561 | 9.061179665 | -2.978238107 | 0.00619483 | 0.018495336 | -2.813530306 |
| C9orf100 | 1.785295668 | 7.378027516 | 2.976533396 | 0.006220471 | 0.018567579 | -2.817386716 |
| DGCR2 | -1.991599396 | 11.71575716 | -2.976319223 | 0.0062237 | 0.018572906 | -2.817871145 |
| RAB11FIP5 | -1.2118124 | 6.682369098 | -2.97599651 | 0.006228567 | 0.01858192 | -2.818601042 |
| YIPF2 | 1.361807015 | 7.601294835 | 2.975353408 | 0.006238279 | 0.018596875 | -2.820055469 |
| FOXE1 | 2.163563289 | 6.964722561 | 2.975208303 | 0.006240472 | 0.018596875 | -2.820383615 |
| GNG7 | 1.084289524 | 7.240129838 | 2.975123139 | 0.006241759 | 0.018596875 | -2.820576205 |
| ZNF16 | -1.525088351 | 4.528241245 | -2.975121731 | 0.006241781 | 0.018596875 | -2.820579388 |
| EXOC8 | -1.364734773 | 8.007940888 | -2.975116951 | 0.006241853 | 0.018596875 | -2.820590198 |
| LYRM7 | -1.479760007 | 8.023407272 | -2.974752996 | 0.006247358 | 0.018608967 | -2.821413213 |
| LOC90246 | 1.394280836 | 6.835648164 | 2.972749524 | 0.006277746 | 0.018695155 | -2.825942807 |
| PUS7L | -1.292538268 | 5.81350632 | -2.97224531 | 0.006285416 | 0.018709332 | -2.827082538 |
| OR4K17 | 1.986325484 | 5.524433731 | 2.971991822 | 0.006289276 | 0.018716488 | -2.827655492 |
| CDC42EP1 | 1.335275791 | 7.839044474 | 2.971419522 | 0.006297997 | 0.018738108 | -2.828948958 |
| MTSS1 | -1.538385127 | 6.519616884 | -2.969739127 | 0.006323672 | 0.018805796 | -2.832746157 |
| TSGA10 | -1.3210599 | 5.084157318 | -2.9678109 | 0.006353256 | 0.018885043 | -2.837102099 |
| GLB1L | -1.457229476 | 4.505609375 | -2.96604296 | 0.006380497 | 0.018957254 | -2.841094742 |
| DBN1 | 1.165437758 | 9.562913969 | 2.965480537 | 0.006389186 | 0.018977816 | -2.842364652 |
| CXCR7 | -2.048988777 | 8.061388432 | -2.965404067 | 0.006390368 | 0.018977816 | -2.842537306 |
| DACT3 | 1.209760852 | 8.846011665 | 2.964222708 | 0.00640866 | 0.019027743 | -2.845204316 |
| STK11 | 1.104993419 | 6.67631005 | 2.963846994 | 0.006414488 | 0.019040651 | -2.846052412 |
| PBOV1 | 2.652368672 | 7.717998712 | 2.962174607 | 0.006440489 | 0.019095799 | -2.849826839 |
| SPINK1 | 2.282077608 | 7.61061558 | 2.961472041 | 0.006451443 | 0.019123866 | -2.851412157 |
| CYP26A1 | 2.444615141 | 7.859277352 | 2.960988797 | 0.006458987 | 0.019138444 | -2.852502475 |
| TWIST1 | 1.526568823 | 6.410277318 | 2.960316069 | 0.006469503 | 0.019164152 | -2.854020175 |
| FLJ22184 | 1.710156552 | 8.889115214 | 2.958892404 | 0.006491812 | 0.01921888 | -2.857231459 |
| VPS26B | 1.279957051 | 5.553776417 | 2.958851056 | 0.006492461 | 0.01921888 | -2.857324714 |
| LOC100507588 | 1.3871954 | 5.752895311 | 2.957740073 | 0.006509923 | 0.019257003 | -2.859830159 |
| GAL3ST4 | 1.109862441 | 5.473829734 | 2.956903957 | 0.006523093 | 0.019279396 | -2.861715433 |
| RFNG | 1.33229634 | 6.702340531 | 2.955885277 | 0.006539174 | 0.019321586 | -2.864011998 |
| FBXO18 | 1.110536354 | 7.944501349 | 2.955642133 | 0.006543018 | 0.019328503 | -2.8645601 |
| SYNGR2 | 1.32052762 | 13.2931462 | 2.955147093 | 0.006550851 | 0.019347198 | -2.86567596 |
| ITPKB | -1.221850018 | 8.060070215 | -2.954277948 | 0.006564625 | 0.019383426 | -2.867634861 |
| VPS37B | -1.247375981 | 7.366450248 | -2.952878879 | 0.006586854 | 0.019444598 | -2.870787529 |
| ELL2 | 1.483952665 | 11.04253366 | 2.952520839 | 0.006592554 | 0.019455223 | -2.871594221 |
| LOC100288594 | 1.736685609 | 8.2067666 | 2.951686275 | 0.006605859 | 0.019487285 | -2.873474373 |
| POLE | 2.442586308 | 7.745510797 | 2.949360214 | 0.006643078 | 0.019588093 | -2.878713275 |
| CGREF1 | 1.61487799 | 5.977881433 | 2.947638393 | 0.006670756 | 0.019656189 | -2.882589968 |
| PROS1 | -1.580815336 | 6.342672171 | -2.947056186 | 0.00668014 | 0.019677696 | -2.883900559 |
| TBX19 | 1.761738863 | 10.57933504 | 2.946995728 | 0.006681115 | 0.019677696 | -2.884036648 |
| TMEM176B | -1.169234744 | 8.036048039 | -2.945712393 | 0.006701846 | 0.019729721 | -2.886925053 |
| NR1H3 | 1.541282661 | 12.46033568 | 2.944369727 | 0.006723602 | 0.019789238 | -2.889946335 |
| CHM | -1.290184601 | 6.687406543 | -2.941641731 | 0.006768011 | 0.019901728 | -2.896082817 |
| C21orf33 | -1.302751683 | 10.72832787 | -2.940983645 | 0.006778766 | 0.019914711 | -2.897562728 |
| FLJ10661 | -1.10240257 | 3.815693738 | -2.940897924 | 0.006780168 | 0.019914711 | -2.897755486 |
| TBL1Y | -1.378009015 | 5.129284178 | -2.938792625 | 0.006814689 | 0.020011537 | -2.902488742 |
| CD24 | -1.426886281 | 8.695312509 | -2.938295111 | 0.006822872 | 0.020030992 | -2.903607038 |
| OSBPL6 | -1.212002864 | 7.3872364 | -2.938124393 | 0.006825681 | 0.020032868 | -2.903990752 |
| LOC646808 | -1.048723877 | 4.41913423 | -2.938067034 | 0.006826626 | 0.020032868 | -2.904119672 |
| UQCRB | -1.053555616 | 11.49079649 | -2.937238758 | 0.006840276 | 0.020068345 | -2.905981165 |
| ZSWIM7 | -1.516857023 | 7.176654693 | -2.936492312 | 0.006852599 | 0.020099916 | -2.907658532 |
| CACNG8 | 1.566498113 | 6.488021208 | 2.936293314 | 0.006855888 | 0.020104979 | -2.90810567 |
| ANTXR1 | -1.637652091 | 8.829274669 | -2.935791056 | 0.006864196 | 0.020124753 | -2.909234155 |
| HBE1 | 1.444365781 | 4.921812222 | 2.935018777 | 0.006876989 | 0.020148481 | -2.910969146 |
| CHAT | 2.274180917 | 6.746584997 | 2.93382562 | 0.006896798 | 0.020201918 | -2.913649231 |
| GPC6 | 1.151243646 | 6.071018191 | 2.931013373 | 0.006943703 | 0.020334679 | -2.919964005 |
| UNC93A | 1.086131096 | 5.122464508 | 2.930827903 | 0.006946807 | 0.020339029 | -2.920380362 |
| ELAC1 | -1.462273475 | 6.265039971 | -2.930735688 | 0.006948351 | 0.020339029 | -2.920587371 |
| MUC5B | 2.134084298 | 8.195433601 | 2.930472294 | 0.006952762 | 0.020347311 | -2.921178629 |
| GPX3 | -1.974142764 | 10.02370673 | -2.92964064 | 0.006966709 | 0.020383487 | -2.923045326 |
| ADAD1 | 2.72794741 | 6.741550915 | 2.929311145 | 0.006972241 | 0.020395035 | -2.923784826 |
| C22orf24 | 1.793898019 | 6.589425899 | 2.928679053 | 0.006982867 | 0.020421472 | -2.925203338 |
| C19orf22 | 1.165227294 | 9.231502355 | 2.928564441 | 0.006984795 | 0.020422468 | -2.92546053 |
| FUT5 | 1.196344084 | 6.53858099 | 2.927295428 | 0.007006179 | 0.020480336 | -2.928307883 |
| NDUFA1 | -1.007977182 | 12.48188244 | -2.926144111 | 0.007025634 | 0.020532539 | -2.930890623 |
| DNAJC27 | -1.017419198 | 5.879979237 | -2.925404411 | 0.00703816 | 0.020564475 | -2.932549722 |
| ATP5O | -1.008677037 | 13.31124354 | -2.924782533 | 0.007048708 | 0.020590615 | -2.933944392 |
| LOC100506174 | 1.428063284 | 6.08267408 | 2.924562901 | 0.007052436 | 0.02059683 | -2.93443692 |
| CYB561D2 | -1.165852112 | 7.167598707 | -2.924051869 | 0.007061119 | 0.020617507 | -2.935582846 |
| LAMP3 | -2.473487309 | 11.27944933 | -2.923797204 | 0.00706545 | 0.02062547 | -2.936153865 |
| WNT11 | 1.364069036 | 5.914764751 | 2.923082367 | 0.00707762 | 0.020656309 | -2.937756561 |
| TMC6 | -1.262341426 | 7.169019522 | -2.922465133 | 0.007088145 | 0.020682332 | -2.939140272 |
| CUEDC1 | -1.069451616 | 5.521344518 | -2.922061649 | 0.007095033 | 0.020697734 | -2.94004472 |
| SH3BP1 | 1.329360319 | 6.503445991 | 2.921123107 | 0.007111079 | 0.020733959 | -2.942148313 |
| EFHA1 | -1.147571137 | 8.998946724 | -2.921052563 | 0.007112286 | 0.020733959 | -2.942306413 |
| ZNF419 | -1.400369148 | 5.7211929 | -2.919741469 | 0.007134763 | 0.020794771 | -2.945244424 |
| RRM2 | 1.352271638 | 6.186612227 | 2.919599759 | 0.007137197 | 0.020797151 | -2.945561942 |
| CELSR1 | -1.969113658 | 7.691616232 | -2.916595272 | 0.007188976 | 0.020938545 | -2.952292013 |
| CCNG1 | -1.08531138 | 7.088990059 | -2.9157039 | 0.007204407 | 0.020973988 | -2.954288028 |
| CRYBB3 | 1.592050736 | 6.532734543 | 2.915425385 | 0.007209235 | 0.020983294 | -2.954911633 |
| ZNF358 | -1.075655545 | 4.856733106 | -2.915321547 | 0.007211035 | 0.020983786 | -2.955144123 |
| IGSF21 | -1.413779595 | 7.678265064 | -2.915030213 | 0.00721609 | 0.020988997 | -2.955796387 |
| PCMTD1 | -1.121762643 | 8.556596474 | -2.914645294 | 0.007222773 | 0.021003687 | -2.956658128 |
| H6PD | 1.547054372 | 8.740747112 | 2.913282421 | 0.007246483 | 0.021067873 | -2.959708816 |
| KIF16B | -1.513309522 | 4.836608729 | -2.911233946 | 0.007282261 | 0.021162322 | -2.964292834 |
| UXS1 | -1.52110308 | 8.070053239 | -2.909997048 | 0.007303945 | 0.021216724 | -2.967059947 |
| ACSF2 | -1.477054777 | 7.636377743 | -2.909883968 | 0.00730593 | 0.021216724 | -2.967312894 |
| CPT1B | 1.470113048 | 7.105850962 | 2.909451795 | 0.007313523 | 0.02123398 | -2.968279566 |
| STX1B | 1.915821309 | 5.091363155 | 2.909008854 | 0.007321313 | 0.021248828 | -2.969270252 |
| DOCK6 | -1.315767151 | 7.776870933 | -2.908940784 | 0.00732251 | 0.021248828 | -2.96942249 |
| SIGLEC9 | 2.31057552 | 8.682298927 | 2.908879264 | 0.007323593 | 0.021248828 | -2.96956008 |
| ZDHHC12 | -1.221933563 | 5.015783489 | -2.908517348 | 0.007329966 | 0.021262521 | -2.970369469 |
| EDA2R | 1.577933859 | 5.819817637 | 2.908029502 | 0.007338564 | 0.021277864 | -2.971460409 |
| AFAP1L1 | -1.028269271 | 7.024809821 | -2.907908082 | 0.007340705 | 0.021278071 | -2.971731918 |
| LYG2 | 1.445431623 | 5.276639096 | 2.907837877 | 0.007341944 | 0.021278071 | -2.971888903 |
| CSN1S2AP | 2.142654223 | 8.989144233 | 2.907292719 | 0.007351567 | 0.021301163 | -2.973107857 |
| C21orf81 | 1.166031717 | 7.539485352 | 2.906808089 | 0.007360133 | 0.021321177 | -2.974191378 |
| KIAA1257 | -1.155860949 | 5.115801332 | -2.906532734 | 0.007365004 | 0.021330483 | -2.974806967 |
| MORC2 | 1.047135747 | 10.70250593 | 2.904525774 | 0.007400598 | 0.021428746 | -2.979292886 |
| LOC100128737 | 1.393651548 | 7.468578709 | 2.904085383 | 0.00740843 | 0.021446596 | -2.980277033 |
| BPIFC | 1.722535939 | 5.565611584 | 2.903283495 | 0.007422712 | 0.021481944 | -2.982068828 |
| TOR1AIP2 | -1.20516669 | 8.092623451 | -2.903212302 | 0.007423981 | 0.021481944 | -2.982227893 |
| PRMT6 | -1.41724528 | 6.184645222 | -2.902456053 | 0.007437476 | 0.021516153 | -2.983917461 |
| FAM131C | 1.895372547 | 14.02803754 | 2.902128114 | 0.007443335 | 0.021528261 | -2.984650053 |
| CD36 | -1.804830106 | 9.283381683 | -2.901221654 | 0.007459553 | 0.021565469 | -2.986674807 |
| C7orf54 | 1.712602568 | 7.397422901 | 2.90011281 | 0.007479438 | 0.021613305 | -2.989151192 |
| MYL10 | 2.317886574 | 7.032006127 | 2.900111546 | 0.007479461 | 0.021613305 | -2.989154014 |
| OSCP1 | -1.35622668 | 7.951371787 | -2.899264522 | 0.007494684 | 0.021649995 | -2.991045354 |
| NFAM1 | 2.472037814 | 7.063494364 | 2.896819501 | 0.007538793 | 0.021765198 | -2.996503341 |
| SESN2 | 2.198643888 | 13.35073378 | 2.895899011 | 0.007555463 | 0.021799401 | -2.998557537 |
| MPL | -1.449675014 | 4.65783924 | -2.895884565 | 0.007555725 | 0.021799401 | -2.998589774 |
| TMEM145 | 1.562830282 | 6.85892361 | 2.895404823 | 0.007564427 | 0.021811964 | -2.999660249 |
| MTMR9 | -1.358174691 | 4.490379341 | -2.895270647 | 0.007566862 | 0.021811964 | -2.999959628 |
| ALOXE3 | 2.217024343 | 7.027433787 | 2.894584114 | 0.007579336 | 0.02183813 | -3.001491336 |
| FYTTD1 | -1.260286682 | 7.26840287 | -2.893915367 | 0.007591505 | 0.021868294 | -3.002983185 |
| ZNF654 | -1.002362763 | 5.59879645 | -2.893462609 | 0.007599754 | 0.021887155 | -3.003993106 |
| ALKBH7 | 1.042540012 | 10.09231782 | 2.893099141 | 0.007606382 | 0.021896439 | -3.004803799 |
| SLC25A3 | -1.405681521 | 13.29110109 | -2.892685964 | 0.007613924 | 0.021903442 | -3.005725302 |
| KCNK9 | 1.903423778 | 6.856328838 | 2.891504809 | 0.007635523 | 0.021960664 | -3.008359251 |
| KCTD1 | 1.083007115 | 8.877677798 | 2.889580679 | 0.007670832 | 0.022052355 | -3.012648853 |
| PCGF1 | 1.798052893 | 9.426338335 | 2.887151074 | 0.007715638 | 0.022171254 | -3.018063282 |
| LYPD4 | 1.613863098 | 5.647662456 | 2.885948422 | 0.007737908 | 0.022226857 | -3.020742567 |
| SPDYE2 | 2.082377152 | 7.591651122 | 2.885919546 | 0.007738444 | 0.022226857 | -3.02080689 |
| NPBWR2 | 2.189862425 | 7.061126497 | 2.88500395 | 0.007755441 | 0.022260763 | -3.022846278 |
| SCRT2 | 2.187258869 | 5.270629892 | 2.88467605 | 0.007761536 | 0.022273289 | -3.02357656 |
| TPP1 | -1.407226795 | 9.09590214 | -2.884371055 | 0.00776721 | 0.022284599 | -3.024255789 |
| MTRR | -1.392552312 | 7.253361061 | -2.883882274 | 0.007776311 | 0.022305735 | -3.025344239 |
| KCNQ3 | 1.658957118 | 6.10583579 | 2.882757223 | 0.007797298 | 0.022355963 | -3.027849223 |
| MUC3 | 1.778386788 | 6.384833083 | 2.881459833 | 0.007821567 | 0.022405566 | -3.030737312 |
| XBP1 | -1.335222722 | 10.30660882 | -2.879837694 | 0.00785201 | 0.022487766 | -3.034347388 |
| COL16A1 | 1.889872312 | 11.64863406 | 2.878582733 | 0.00787564 | 0.022540383 | -3.037139599 |
| SLC2A5 | 1.53360738 | 6.278686744 | 2.878410286 | 0.007878892 | 0.022544675 | -3.037523235 |
| TTC25 | -1.79999286 | 8.024327897 | -2.878112233 | 0.007884516 | 0.02255575 | -3.038186274 |
| STOX2 | -1.067062321 | 7.565023251 | -2.877480238 | 0.007896454 | 0.022581345 | -3.039592072 |
| ANO1 | -1.498008036 | 5.557641252 | -2.877452716 | 0.007896975 | 0.022581345 | -3.039653288 |
| LRRC18 | 1.242245789 | 6.394038164 | 2.877191013 | 0.007901924 | 0.022590473 | -3.040235367 |
| RHOQ | 1.011263929 | 10.26495939 | 2.876063174 | 0.007923284 | 0.022646507 | -3.042743595 |
| FAT2 | 1.83703708 | 6.313204364 | 2.875749951 | 0.007929226 | 0.022658456 | -3.043440089 |
| SLC1A4 | 1.147901046 | 6.919862535 | 2.87565386 | 0.00793105 | 0.022658633 | -3.043653755 |
| ZNF10 | -1.23100199 | 5.771604932 | -2.875420002 | 0.00793549 | 0.022666284 | -3.044173737 |
| MMAB | -1.203193525 | 7.387278784 | -2.8748385 | 0.007946541 | 0.022692809 | -3.045466609 |
| CHD1L | -1.984733404 | 6.908898522 | -2.873291412 | 0.007976013 | 0.022766862 | -3.048905655 |
| WNT3A | -2.050621128 | 7.572954537 | -2.872806916 | 0.007985264 | 0.02278821 | -3.049982454 |
| PNMAL1 | 1.295019348 | 8.876178812 | 2.8723685 | 0.007993644 | 0.022802006 | -3.050956759 |
| KIAA0415 | -1.227363064 | 5.572054141 | -2.871501485 | 0.00801024 | 0.022841653 | -3.052883332 |
| FOXI3 | 1.287361186 | 7.799889494 | 2.871456895 | 0.008011095 | 0.022841653 | -3.052982405 |
| GSPT2 | -1.451853056 | 5.632781226 | -2.869449637 | 0.008049647 | 0.022936322 | -3.057441509 |
| FLJ20021 | 1.216471485 | 8.549009013 | 2.867791454 | 0.008081627 | 0.023017247 | -3.061123944 |
| HEY1 | -1.757869093 | 7.310489013 | -2.866730772 | 0.008102147 | 0.02307058 | -3.063478901 |
| E2F6 | 1.53574105 | 9.06262159 | 2.865879527 | 0.00811865 | 0.023112457 | -3.065368536 |
| ROR1 | -1.220171755 | 7.129891431 | -2.865679252 | 0.008122538 | 0.023114258 | -3.065813073 |
| PSME4 | -1.111382288 | 6.276804075 | -2.865661769 | 0.008122877 | 0.023114258 | -3.065851879 |
| CTF1 | 1.572542073 | 6.489749356 | 2.863908382 | 0.008156988 | 0.023201058 | -3.069743077 |
| KIFC3 | 1.011652215 | 6.546943754 | 2.863725576 | 0.008160553 | 0.023206064 | -3.070148697 |
| SPATA6 | -1.307599339 | 5.386777349 | -2.863506189 | 0.008164832 | 0.023207971 | -3.070635469 |
| PLEKHN1 | 1.514350218 | 6.172746377 | 2.86245465 | 0.008185373 | 0.023261216 | -3.072968341 |
| LAMP1 | 1.420892755 | 13.3945333 | 2.862246329 | 0.008189448 | 0.023267656 | -3.073430456 |
| CALD1 | -1.224058767 | 10.98710582 | -2.861913486 | 0.008195963 | 0.023278699 | -3.074168758 |
| TERF1 | -1.03565346 | 8.331276391 | -2.86146238 | 0.0082048 | 0.023295837 | -3.07516932 |
| MCART2 | 1.750816548 | 5.892547354 | 2.861327535 | 0.008207444 | 0.023296565 | -3.075468392 |
| RAB38 | 2.160608801 | 8.367939817 | 2.861264545 | 0.008208679 | 0.023296565 | -3.075608094 |
| GZF1 | -1.226994588 | 5.968156882 | -2.860911754 | 0.0082156 | 0.023311063 | -3.076390507 |
| PANX3 | 2.192198875 | 7.444355656 | 2.860676266 | 0.008220223 | 0.023319036 | -3.076912739 |
| DEPDC4 | 1.276412465 | 7.95112536 | 2.860190637 | 0.008229764 | 0.023340954 | -3.07798963 |
| PEX19 | -1.034744263 | 7.741060624 | -2.860012235 | 0.008233272 | 0.023345754 | -3.078385218 |
| CAMK1G | 1.406470394 | 5.689592705 | 2.859771495 | 0.008238007 | 0.023350543 | -3.07891901 |
| NLRC5 | 1.118065209 | 6.695647432 | 2.859741791 | 0.008238592 | 0.023350543 | -3.078984871 |
| PSMA1 | -1.054395726 | 11.19114006 | -2.858272915 | 0.008267544 | 0.023422279 | -3.082241298 |
| KCNH1 | 1.519118887 | 6.300726913 | 2.857463165 | 0.008283545 | 0.023462444 | -3.084036106 |
| TDRD10 | 1.486323075 | 6.928470146 | 2.857153268 | 0.008289677 | 0.023474642 | -3.084722921 |
| TAB2 | -1.091997563 | 10.59070476 | -2.856861904 | 0.008295446 | 0.023485807 | -3.08536863 |
| CRNDE | -1.882194032 | 8.957856648 | -2.856181723 | 0.008308928 | 0.023513625 | -3.086875883 |
| ADAMTS13 | 1.022254012 | 7.839226757 | 2.855449681 | 0.008323461 | 0.023548982 | -3.088497853 |
| MTMR2 | -1.054746198 | 6.671154018 | -2.854940843 | 0.008333577 | 0.023567824 | -3.089625149 |
| PRPF40B | 1.332455375 | 7.594927201 | 2.852636571 | 0.008379533 | 0.023672301 | -3.094728809 |
| ADH1B | -1.609909377 | 6.47877482 | -2.852627122 | 0.008379722 | 0.023672301 | -3.094749733 |
| VSTM4 | -1.298912852 | 5.08207182 | -2.849843101 | 0.008435568 | 0.02381437 | -3.100913132 |
| ACTR8 | -1.419037601 | 5.61292271 | -2.847425746 | 0.008484343 | 0.023941559 | -3.10626227 |
| CRAMP1L | -1.055453411 | 8.289844614 | -2.846862111 | 0.008495754 | 0.0239685 | -3.107509149 |
| GJA3 | 1.337905337 | 4.730174038 | 2.846589993 | 0.008501269 | 0.023978798 | -3.108111085 |
| SMG1 | 1.354334998 | 10.43004278 | 2.846331206 | 0.008506516 | 0.023988338 | -3.108683505 |
| FAM22A | 1.487964549 | 6.569290616 | 2.845966008 | 0.008513926 | 0.024003971 | -3.109491253 |
| FBXL14 | 2.307581294 | 9.2156534 | 2.844620363 | 0.008541283 | 0.024070548 | -3.112467101 |
| PCP2 | 1.340663354 | 7.63894867 | 2.844160799 | 0.008550645 | 0.024086058 | -3.113483243 |
| ASB13 | -1.130686548 | 6.559567556 | -2.844074468 | 0.008552405 | 0.024086058 | -3.113674121 |
| SP1 | 1.102884095 | 8.22266027 | 2.842372547 | 0.008587166 | 0.024169172 | -3.117436443 |
| KRTAP10-10 | 1.054724446 | 6.738401588 | 2.84235362 | 0.008587554 | 0.024169172 | -3.117478278 |
| NECAP1 | 1.000034303 | 6.273236106 | 2.841632906 | 0.008602315 | 0.024194834 | -3.119071154 |
| TSPAN18 | -1.229907874 | 6.16496589 | -2.841188212 | 0.008611435 | 0.02421519 | -3.120053884 |
| CXCR5 | 1.629085828 | 5.722187701 | 2.840274296 | 0.008630207 | 0.024262671 | -3.122073299 |
| COG7 | -1.573811523 | 5.088841307 | -2.840013989 | 0.008635561 | 0.024272417 | -3.122648421 |
| SLC26A10 | 1.200987468 | 6.665081046 | 2.839450003 | 0.008647171 | 0.024299741 | -3.123894394 |
| MRPL23 | -1.137624538 | 7.060096634 | -2.838540013 | 0.008665935 | 0.024347152 | -3.125904497 |
| C9orf174 | -1.507895038 | 5.766888476 | -2.837847322 | 0.008680245 | 0.024376705 | -3.127434378 |
| ZNF117 | -1.447334858 | 7.186294531 | -2.837568285 | 0.008686015 | 0.024387585 | -3.128050606 |
| CAT | -1.161157783 | 8.723601517 | -2.83722648 | 0.008693089 | 0.024400112 | -3.128805409 |
| LOC387723 | -1.190190202 | 6.008478176 | -2.837111248 | 0.008695475 | 0.024400112 | -3.129059862 |
| ADAM19 | 1.132893295 | 8.672043157 | 2.83707777 | 0.008696168 | 0.024400112 | -3.129133787 |
| OXCT2 | 2.03643467 | 7.859559217 | 2.836366648 | 0.008710907 | 0.024434607 | -3.130703952 |
| C9orf116 | -1.191923027 | 6.852344168 | -2.835742743 | 0.008723857 | 0.024461795 | -3.132081373 |
| ASTL | 1.808520495 | 8.662972155 | 2.834913645 | 0.008741096 | 0.024499449 | -3.133911565 |
| OFD1 | -1.074055721 | 7.465947628 | -2.834502297 | 0.00874966 | 0.02451811 | -3.134819491 |
| CXCL3 | 1.461133398 | 9.149309052 | 2.83439534 | 0.008751888 | 0.024519012 | -3.135055553 |
| NEURL4 | 1.101737361 | 9.532815023 | 2.834259939 | 0.00875471 | 0.024521576 | -3.13535439 |
| CERS4 | -1.282026177 | 5.681615048 | -2.834124478 | 0.008757533 | 0.024522115 | -3.135653351 |
| TMEM174 | 2.736436449 | 6.218286228 | 2.832792469 | 0.008785344 | 0.024591316 | -3.138592683 |
| CD3EAP | 1.384668579 | 7.135789406 | 2.832572671 | 0.008789941 | 0.024598831 | -3.13907764 |
| ST8SIA1 | -1.542292001 | 5.860734535 | -2.831439471 | 0.008813679 | 0.024654532 | -3.141577594 |
| ESRP1 | -1.496523641 | 5.310311768 | -2.831087208 | 0.00882107 | 0.024669842 | -3.142354616 |
| PKP1 | 2.169629485 | 6.475487938 | 2.830590534 | 0.008831501 | 0.024693646 | -3.143450094 |
| KRTAP3-1 | 1.744281462 | 5.794536567 | 2.829580267 | 0.008852755 | 0.024747694 | -3.14567806 |
| APLN | 1.613731177 | 7.972733399 | 2.829003309 | 0.008864915 | 0.024776301 | -3.146950251 |
| LYN | -1.625363405 | 8.907305542 | -2.828289976 | 0.008879971 | 0.024812988 | -3.148522964 |
| ERLEC1 | -1.496197157 | 7.938847153 | -2.827941489 | 0.008887335 | 0.024822947 | -3.149291212 |
| GP9 | 1.097481424 | 6.678457101 | 2.827938646 | 0.008887395 | 0.024822947 | -3.14929748 |
| MED24 | 1.851299824 | 8.01017765 | 2.827320938 | 0.008900462 | 0.024854047 | -3.150659109 |
| ASPG | -1.187816864 | 5.029841237 | -2.827008902 | 0.00890707 | 0.0248671 | -3.151346878 |
| UBXN8 | -1.345630425 | 6.212930903 | -2.825820409 | 0.00893228 | 0.024932072 | -3.153966116 |
| SOX1 | 1.318197917 | 8.486239568 | 2.824281039 | 0.008965034 | 0.025018066 | -3.157357771 |
| UQCRFS1 | -1.073222426 | 9.835172963 | -2.823253565 | 0.008986959 | 0.025068372 | -3.159621043 |
| SP110 | -1.157269895 | 7.394918761 | -2.823131622 | 0.008989564 | 0.025070203 | -3.159889625 |
| TMEM132A | 1.607708661 | 9.773143481 | 2.823001289 | 0.00899235 | 0.025072535 | -3.160176679 |
| GMCL1 | -1.217334475 | 6.124112849 | -2.821408979 | 0.009026447 | 0.025156698 | -3.163683129 |
| PON2 | -1.51721973 | 10.94557641 | -2.821098714 | 0.009033105 | 0.0251698 | -3.164366249 |
| FAM181B | 1.409278423 | 6.514434512 | 2.819912088 | 0.009058612 | 0.025224479 | -3.166978511 |
| GZMM | 1.217417345 | 7.259725553 | 2.818918675 | 0.009080018 | 0.025273143 | -3.169164989 |
| CCDC111 | -1.091172077 | 6.070124724 | -2.818774357 | 0.009083132 | 0.02527634 | -3.169482596 |
| SGK1 | -2.360522657 | 8.731439289 | -2.817449383 | 0.009111766 | 0.025342947 | -3.172398125 |
| IQCA1 | -1.364272363 | 6.94187776 | -2.817402065 | 0.00911279 | 0.025342947 | -3.172502233 |
| LOC100509100 | -1.230443563 | 6.564606008 | -2.817393371 | 0.009112979 | 0.025342947 | -3.172521362 |
| SELM | 1.007586537 | 9.299230316 | 2.81674048 | 0.009127122 | 0.025376791 | -3.173957731 |
| SSPN | -1.591222821 | 7.706955632 | -2.81635763 | 0.009135424 | 0.025391577 | -3.174799926 |
| C17orf76 | -1.426196326 | 6.197660356 | -2.816281313 | 0.00913708 | 0.025391577 | -3.174967801 |
| MAST1 | 1.143035672 | 5.997066671 | 2.816222256 | 0.009138362 | 0.025391577 | -3.175097707 |
| LRPPRC | -1.112605102 | 8.122775219 | -2.81611285 | 0.009140737 | 0.02539269 | -3.175338361 |
| MYBPH | 1.997500125 | 5.715741229 | 2.815732739 | 0.009148992 | 0.025410134 | -3.176174435 |
| MBP | -1.880543017 | 9.058146721 | -2.815392 | 0.009156398 | 0.025419725 | -3.176923854 |
| ALPI | 1.249433161 | 5.468772878 | 2.815154718 | 0.009161558 | 0.025428564 | -3.177445703 |
| MLST8 | 1.405766615 | 7.469777115 | 2.81444491 | 0.009177013 | 0.025460853 | -3.179006632 |
| JAM2 | -1.594438101 | 7.416207576 | -2.812897177 | 0.009210796 | 0.025543177 | -3.182409514 |
| PRELID1 | 1.140108609 | 9.996671008 | 2.812391392 | 0.009221861 | 0.02556835 | -3.183521332 |
| FUT11 | 1.328114947 | 8.633693598 | 2.812029879 | 0.009229778 | 0.025584785 | -3.184315945 |
| TMEM106C | 1.185077084 | 11.49160099 | 2.811705638 | 0.009236884 | 0.025598966 | -3.185028588 |
| C17orf50 | 1.725510621 | 7.420900067 | 2.811592327 | 0.009239368 | 0.025600335 | -3.185277622 |
| NPIPL2 | 1.084304932 | 9.678115973 | 2.811290652 | 0.009245986 | 0.025607638 | -3.185940615 |
| CCT8 | -1.125213745 | 9.516153472 | -2.811128005 | 0.009249556 | 0.02561201 | -3.18629805 |
| ZNF189 | -1.132563034 | 7.213881472 | -2.810987416 | 0.009252643 | 0.025615043 | -3.186607001 |
| MEGF10 | 1.655640818 | 7.039985302 | 2.810511228 | 0.009263105 | 0.025638488 | -3.187653387 |
| CATSPER1 | -1.109273254 | 4.720664545 | -2.809009086 | 0.009296181 | 0.025724502 | -3.190953617 |
| LOC149837 | 1.43538096 | 6.247757679 | 2.808892242 | 0.009298759 | 0.025726099 | -3.191210286 |
| HHLA3 | 1.506596089 | 7.130158444 | 2.808458455 | 0.009308334 | 0.025747051 | -3.192163129 |
| AHR | -2.086925579 | 9.110373015 | -2.808308527 | 0.009311645 | 0.025750673 | -3.192492437 |
| PERP | -1.646683303 | 10.51396559 | -2.805589896 | 0.009371886 | 0.025906125 | -3.198462165 |
| RUNX1T1 | -1.242510746 | 7.799844334 | -2.805430221 | 0.009375436 | 0.025910368 | -3.198812694 |
| SYT13 | -1.621448897 | 4.53852347 | -2.805246592 | 0.009379519 | 0.025916085 | -3.199215795 |
| TRPV5 | 1.458743518 | 4.772085361 | 2.805066285 | 0.009383531 | 0.025921601 | -3.199611589 |
| SRPR | -1.220823219 | 10.58167689 | -2.804200157 | 0.009402823 | 0.025958168 | -3.201512657 |
| LOC653061 | 1.094091544 | 8.679976503 | 2.804000441 | 0.009407276 | 0.025964856 | -3.201950969 |
| C3orf14 | -1.581838386 | 5.681392061 | -2.803910495 | 0.009409283 | 0.025964856 | -3.202148366 |
| IGFBPL1 | 1.481339083 | 6.681006654 | 2.803593938 | 0.009416347 | 0.025978778 | -3.202843062 |
| C19orf42 | -1.264505952 | 8.376709244 | -2.803258368 | 0.009423842 | 0.025993879 | -3.203579438 |
| CNTNAP1 | 1.138050061 | 6.846135256 | 2.802670809 | 0.009436978 | 0.026024531 | -3.204868668 |
| ATG4C | -1.338226857 | 5.356519168 | -2.801484621 | 0.00946355 | 0.026086623 | -3.207470982 |
| C2CD4A | 1.281677228 | 6.477688721 | 2.801070528 | 0.009472843 | 0.026101869 | -3.208379302 |
| EPN1 | 1.246294715 | 5.094766102 | 2.801057311 | 0.00947314 | 0.026101869 | -3.208408292 |
| HES1 | -1.407470499 | 10.93004777 | -2.800288943 | 0.009490406 | 0.026143844 | -3.210093528 |
| LOC338799 | 1.352099174 | 9.926033622 | 2.800145232 | 0.009493639 | 0.026147149 | -3.210408696 |
| BTN3A3 | -1.378900815 | 7.482554154 | -2.799996749 | 0.00949698 | 0.026150751 | -3.210734322 |
| VEZT | -1.524500479 | 7.604104516 | -2.798846479 | 0.009522901 | 0.026199691 | -3.213256573 |
| NCKAP5 | -1.494494808 | 7.470046609 | -2.797960216 | 0.009542918 | 0.026239569 | -3.215199548 |
| LRRC58 | -1.146682294 | 6.240758661 | -2.797933775 | 0.009543516 | 0.026239569 | -3.21525751 |
| BEND7 | -1.552272817 | 5.820395535 | -2.796850751 | 0.009568033 | 0.026301354 | -3.217631398 |
| DDX55 | -1.268874598 | 5.730494198 | -2.796481146 | 0.009576413 | 0.026318766 | -3.218441426 |
| LPIN1 | -1.147849534 | 7.188532609 | -2.795962065 | 0.009588194 | 0.026334264 | -3.219578948 |
| TIMD4 | 1.580755529 | 6.510296888 | 2.795684357 | 0.009594503 | 0.026345964 | -3.220187477 |
| LRRC16B | 1.330740864 | 5.522715431 | 2.795544382 | 0.009597684 | 0.026349075 | -3.220494186 |
| PRINS | -1.678261941 | 8.463167446 | -2.795245297 | 0.009604485 | 0.026362118 | -3.221149503 |
| SLC38A4 | 1.198306291 | 4.578204314 | 2.794244053 | 0.009627285 | 0.02641906 | -3.223343036 |
| CALML6 | 1.222580373 | 4.316350183 | 2.792643529 | 0.009663837 | 0.026513709 | -3.226848606 |
| LRRC31 | 1.640623485 | 6.98645718 | 2.792043565 | 0.009677572 | 0.026540071 | -3.228162414 |
| NMB | -1.066582424 | 8.841156426 | -2.790579929 | 0.009711158 | 0.026623943 | -3.231366865 |
| MXD1 | 1.381331351 | 10.21213567 | 2.79053043 | 0.009712295 | 0.026623943 | -3.231475221 |
| SLC2A4RG | 1.049410727 | 10.98379242 | 2.789870288 | 0.009727481 | 0.026654209 | -3.232920216 |
| ZNF605 | 1.104003659 | 6.259593528 | 2.788808014 | 0.009751964 | 0.026709915 | -3.235245061 |
| KIAA2026 | -1.181797294 | 5.47635269 | -2.787745196 | 0.009776517 | 0.026765766 | -3.237570625 |
| UBE2L6 | 1.036099079 | 7.360864005 | 2.787490003 | 0.009782422 | 0.026776231 | -3.238128946 |
| AQP10 | 1.330792986 | 6.823796506 | 2.787274421 | 0.009787412 | 0.02678419 | -3.238600584 |
| ZNF786 | -1.088037531 | 5.5776418 | -2.787047283 | 0.009792673 | 0.026789792 | -3.239097481 |
| SLC4A7 | 1.299897327 | 7.74334376 | 2.787006183 | 0.009793625 | 0.026789792 | -3.239187391 |
| ZNF157 | 1.726207872 | 5.961417804 | 2.786653873 | 0.00980179 | 0.026800729 | -3.239958072 |
| ALMS1 | -1.072393926 | 5.555761933 | -2.786429233 | 0.009807 | 0.02680202 | -3.240449447 |
| LOC440905 | 2.640998043 | 7.002232962 | 2.786233963 | 0.009811531 | 0.026804569 | -3.240876562 |
| TMCO3 | -1.503341912 | 7.763230524 | -2.785808236 | 0.009821416 | 0.026825876 | -3.241807699 |
| TAS2R60 | 1.663860668 | 5.915809675 | 2.78555053 | 0.009827404 | 0.026836533 | -3.242371308 |
| C18orf55 | -1.125170336 | 7.229436509 | -2.784766104 | 0.009845653 | 0.026880405 | -3.244086698 |
| FAN1 | -1.959116579 | 7.32695717 | -2.784680348 | 0.00984765 | 0.026880405 | -3.244274214 |
| FAM206A | -1.059990366 | 7.091941518 | -2.78441537 | 0.009853822 | 0.026891547 | -3.244853604 |
| FANK1 | -1.186147898 | 6.457053618 | -2.783872063 | 0.00986649 | 0.026919032 | -3.246041481 |
| ACY1 | -1.084744169 | 7.151175648 | -2.783803945 | 0.009868079 | 0.026919032 | -3.246190404 |
| CERS2 | -1.230307888 | 9.466316688 | -2.783458916 | 0.009876133 | 0.02693529 | -3.246944697 |
| CPS1-IT1 | 1.489558874 | 5.627669976 | 2.78287088 | 0.009889874 | 0.026961328 | -3.24823013 |
| SLC25A1 | -1.079668315 | 6.723078234 | -2.782781296 | 0.009891969 | 0.026961328 | -3.248425944 |
| SSX3 | 3.049914865 | 7.101311182 | 2.782539751 | 0.00989762 | 0.026971013 | -3.248953905 |
| ASMTL-AS1 | 1.098292835 | 5.653911163 | 2.781619269 | 0.009919181 | 0.027018319 | -3.250965637 |
| CXorf38 | -1.209907665 | 5.634594082 | -2.780908191 | 0.009935867 | 0.027052312 | -3.25251947 |
| CCL13 | 2.273241869 | 9.746628115 | 2.780487882 | 0.009945743 | 0.02706774 | -3.25343782 |
| HHAT | 1.00735491 | 7.75345069 | 2.780237901 | 0.009951621 | 0.027078008 | -3.253983978 |
| SMARCC2 | 1.153520111 | 13.69544208 | 2.779955901 | 0.009958255 | 0.027084601 | -3.254600061 |
| PIP4K2C | 1.774261523 | 9.268722212 | 2.779630164 | 0.009965924 | 0.027099729 | -3.255311655 |
| FBN3 | 1.872651351 | 10.84366232 | 2.779522863 | 0.009968452 | 0.027100872 | -3.25554605 |
| LCE1F | 1.406235188 | 5.855038437 | 2.778413155 | 0.009994626 | 0.027154523 | -3.257969889 |
| KRTAP11-1 | 1.558199003 | 5.831168834 | 2.778379925 | 0.009995411 | 0.027154523 | -3.258042464 |
| PDCD6IP | -1.069945997 | 9.326051812 | -2.778328257 | 0.009996631 | 0.027154523 | -3.258155306 |
| FMO4 | -1.098947381 | 6.121456055 | -2.776499299 | 0.010039919 | 0.02726416 | -3.262148947 |
| C9orf140 | 2.328442611 | 7.017939583 | 2.776443947 | 0.010041232 | 0.02726416 | -3.26226979 |
| CREBBP | -1.205140409 | 7.339319314 | -2.776346583 | 0.010043542 | 0.027264676 | -3.262482349 |
| SSX2 | 1.493168757 | 5.806522225 | 2.775361741 | 0.010066932 | 0.027311196 | -3.264632168 |
| SLC30A1 | 1.014112366 | 7.286825602 | 2.775236746 | 0.010069905 | 0.027311196 | -3.264904991 |
| EZH1 | -1.272830032 | 7.084500229 | -2.774023477 | 0.010098799 | 0.027375105 | -3.267552818 |
| LOC100509196 | 1.841413151 | 7.636428206 | 2.774010051 | 0.010099119 | 0.027375105 | -3.267582114 |
| PRR5 | 1.91692956 | 11.94199058 | 2.773836578 | 0.010103257 | 0.02738055 | -3.267960649 |
| PRSS1 | 1.80197549 | 5.650834107 | 2.773411171 | 0.010113411 | 0.027402294 | -3.268888873 |
| CCDC42B | -1.572713186 | 4.888680417 | -2.773239959 | 0.0101175 | 0.027407601 | -3.26926243 |
| OR10H4 | 1.695240139 | 5.003508885 | 2.772930985 | 0.010124884 | 0.027421827 | -3.269936532 |
| HSPA4 | 1.064103135 | 9.67143476 | 2.772065336 | 0.010145598 | 0.02746636 | -3.271824939 |
| PRR3 | 1.337324473 | 6.870864674 | 2.771209929 | 0.010166105 | 0.027516087 | -3.273690692 |
| KGFLP2 | 1.096554955 | 5.161749994 | 2.771003552 | 0.010171059 | 0.027523703 | -3.274140779 |
| SLC1A7 | 1.630796466 | 7.704405112 | 2.769325102 | 0.010211431 | 0.027609716 | -3.277800653 |
| RPS11 | -1.221870066 | 13.75611666 | -2.768878337 | 0.010222202 | 0.027626107 | -3.278774627 |
| ABL2 | 1.835800554 | 7.872758908 | 2.768806442 | 0.010223936 | 0.027626107 | -3.278931355 |
| PPP1R3C | -1.342710222 | 9.488715 | -2.768252688 | 0.010237304 | 0.027656418 | -3.280138438 |
| HILPDA | 2.726992192 | 10.96829651 | 2.767006881 | 0.010267439 | 0.027732003 | -3.282853593 |
| TDRD6 | 1.959473402 | 6.887657144 | 2.766588411 | 0.01027758 | 0.027753565 | -3.283765475 |
| PTPRF | -1.347159994 | 9.207723476 | -2.765598163 | 0.010301614 | 0.027806792 | -3.285923013 |
| ANGPTL4 | 3.018841313 | 10.31078373 | 2.764501817 | 0.010328286 | 0.027866507 | -3.288311232 |
| RBM5 | -1.448079409 | 10.3427488 | -2.764421741 | 0.010330237 | 0.027866507 | -3.288485646 |
| GPR143 | -1.140745342 | 4.677026354 | -2.763649021 | 0.010349078 | 0.027911477 | -3.290168563 |
| PFKFB2 | 1.179745946 | 6.580409803 | 2.763134358 | 0.010361644 | 0.027933655 | -3.291289315 |
| SPIC | 1.527299892 | 6.084035428 | 2.762745092 | 0.010371159 | 0.027953446 | -3.292136921 |
| PPP1R13B | -1.581038294 | 5.688964438 | -2.76255651 | 0.010375771 | 0.027960018 | -3.292547527 |
| FFAR3 | 1.184713395 | 4.790528697 | 2.760909592 | 0.010416133 | 0.028062903 | -3.296132762 |
| EVI5L | 1.371652164 | 6.670483281 | 2.760049448 | 0.010437271 | 0.028113965 | -3.298004784 |
| TAAR2 | 1.725398157 | 7.49078483 | 2.759894447 | 0.010441085 | 0.028118349 | -3.298342094 |
| TTC23 | -1.298646785 | 4.901927501 | -2.759624991 | 0.010447718 | 0.028130321 | -3.298928457 |
| LOC652276 | 1.723305001 | 6.821601707 | 2.758916472 | 0.010465177 | 0.028165537 | -3.300470118 |
| MTHFD1 | -1.390543579 | 7.967616458 | -2.758419961 | 0.010477428 | 0.028186714 | -3.301550343 |
| CCDC74B | -1.178695527 | 5.644025159 | -2.757970302 | 0.010488535 | 0.028210693 | -3.302528546 |
| C11orf75 | 1.025567208 | 10.72899457 | 2.757592872 | 0.010497866 | 0.028229888 | -3.303349554 |
| MAOB | -1.173839647 | 9.362605327 | -2.757399444 | 0.010502651 | 0.028236852 | -3.303770286 |
| ZNF813 | 1.753907974 | 7.723189758 | 2.756565062 | 0.010523316 | 0.028286498 | -3.305584996 |
| FAM200B | -1.604987264 | 4.312426491 | -2.756032979 | 0.010536514 | 0.028316057 | -3.306742079 |
| HIST3H2BB | 1.018851566 | 9.780784297 | 2.7555261 | 0.010549102 | 0.028339568 | -3.307844238 |
| NPNT | -1.118638882 | 11.1646137 | -2.754614825 | 0.010571767 | 0.028392662 | -3.309825443 |
| FAR2 | -1.229032955 | 5.459914509 | -2.754531198 | 0.010573849 | 0.028392662 | -3.310007237 |
| PRR15L | -1.56611814 | 9.651483268 | -2.754175424 | 0.010582712 | 0.028410529 | -3.310780619 |
| TTC9B | 2.694296659 | 7.565225653 | 2.754075922 | 0.010585192 | 0.028410687 | -3.310996906 |
| MRVI1 | 1.135958913 | 8.885340957 | 2.753995833 | 0.010587189 | 0.028410687 | -3.311170991 |
| ZNF429 | -1.287519228 | 9.790127705 | -2.752075688 | 0.010635163 | 0.028527521 | -3.315343914 |
| MAPK8IP1 | 1.373529986 | 6.343633333 | 2.751055146 | 0.010660744 | 0.028590176 | -3.317561149 |
| ATP6V0A4 | -1.123951807 | 4.356057785 | -2.750045658 | 0.010686104 | 0.028640274 | -3.319753929 |
| CCL24 | 1.681005178 | 14.43480745 | 2.749859596 | 0.010690785 | 0.028646849 | -3.32015804 |
| MTHFD1L | 1.105027639 | 6.519970374 | 2.748814626 | 0.010717108 | 0.028705422 | -3.322427351 |
| LOC145694 | 1.853192891 | 6.301430275 | 2.748329485 | 0.010729349 | 0.028730823 | -3.323480748 |
| KLHL14 | 1.434001424 | 4.66310697 | 2.748085664 | 0.010735506 | 0.028730823 | -3.324010124 |
| SNRNP200 | -1.797274537 | 8.902783294 | -2.748084883 | 0.010735526 | 0.028730823 | -3.32401182 |
| RPUSD1 | 1.363927181 | 8.119904409 | 2.747515992 | 0.010749905 | 0.02876332 | -3.32524688 |
| MYBL2 | 1.298478345 | 5.368225603 | 2.746760368 | 0.010769032 | 0.028805604 | -3.326887119 |
| SH3BP2 | 1.128130797 | 7.498194088 | 2.746714766 | 0.010770187 | 0.028805604 | -3.3269861 |
| C5orf44 | -1.620212654 | 6.193749281 | -2.745869442 | 0.010791625 | 0.028856939 | -3.328820746 |
| BMP2 | -1.236611616 | 5.452468601 | -2.745465298 | 0.010801888 | 0.028875867 | -3.32969777 |
| OMA1 | -1.586444201 | 6.499179248 | -2.745413924 | 0.010803193 | 0.028875867 | -3.32980925 |
| JUNB | 1.596248889 | 13.48628442 | 2.745222088 | 0.010808069 | 0.028882897 | -3.330225522 |
| SIX3 | 1.694407939 | 6.057351803 | 2.744849358 | 0.010817548 | 0.028902222 | -3.331034274 |
| C5orf4 | -1.250834664 | 10.46531597 | -2.742932632 | 0.010866415 | 0.029026756 | -3.335192259 |
| DHRS3 | -1.283655689 | 9.935883832 | -2.742595446 | 0.010875033 | 0.029040428 | -3.335923559 |
| RAB40C | -1.329855384 | 5.474647856 | -2.740795131 | 0.010921156 | 0.029136674 | -3.339827311 |
| APBB1 | 1.672844371 | 8.817251972 | 2.739197392 | 0.010962242 | 0.029240225 | -3.343290636 |
| NEK11 | -1.003907674 | 4.689836677 | -2.738572018 | 0.010978364 | 0.029277154 | -3.344645923 |
| WDHD1 | 1.542635878 | 7.111520364 | 2.738417568 | 0.010982349 | 0.02928171 | -3.344980615 |
| SH3PXD2A | 1.165421474 | 10.5296028 | 2.736610476 | 0.011029075 | 0.02938451 | -3.348895807 |
| SULF1 | 1.797282161 | 9.862350168 | 2.736573092 | 0.011030043 | 0.02938451 | -3.348976788 |
| PKNOX2 | -1.548665863 | 6.775121993 | -2.735911419 | 0.0110472 | 0.029417782 | -3.350409986 |
| NEU4 | 1.884014972 | 6.885497473 | 2.735738757 | 0.011051681 | 0.029417782 | -3.350783945 |
| CCDC57 | 1.20028824 | 7.360288157 | 2.734860426 | 0.011074503 | 0.029472432 | -3.352686072 |
| HRG | 1.960948577 | 6.088339576 | 2.731673956 | 0.011157672 | 0.02966483 | -3.35958394 |
| GORASP2 | -1.459671267 | 9.381870026 | -2.7316011 | 0.01115958 | 0.02966483 | -3.359741603 |
| TPTE2P1 | 1.71325223 | 6.461192437 | 2.73158212 | 0.011160077 | 0.02966483 | -3.359782676 |
| KCNH7 | 1.838239557 | 4.647964952 | 2.730542036 | 0.011187356 | 0.029729706 | -3.36203319 |
| ZNF192 | -1.124393563 | 4.281817007 | -2.730164646 | 0.01119727 | 0.029749903 | -3.362849664 |
| ACSL3 | -1.05435335 | 6.823268899 | -2.729888443 | 0.01120453 | 0.029761818 | -3.363447183 |
| TAF7 | -1.246811579 | 10.84085136 | -2.729758045 | 0.01120796 | 0.029761818 | -3.363729266 |
| STT3A | -1.359679526 | 7.719031129 | -2.729548026 | 0.011213485 | 0.029768389 | -3.364183572 |
| PLXNA4 | 1.606289778 | 5.262614264 | 2.729255785 | 0.011221178 | 0.029780637 | -3.364815709 |
| HELLS | 1.467649579 | 6.127465481 | 2.729196865 | 0.01122273 | 0.029780637 | -3.364943152 |
| HIST1H3H | 1.374729325 | 6.543434717 | 2.728280745 | 0.011246881 | 0.029832415 | -3.366924513 |
| C7orf51 | 1.193659253 | 4.05141061 | 2.725794863 | 0.01131266 | 0.030000709 | -3.372299081 |
| AARSD1 | -1.251257213 | 5.37342372 | -2.725571285 | 0.011318594 | 0.030010257 | -3.372782333 |
| CTSE | 2.111456973 | 9.478039243 | 2.724981455 | 0.011334262 | 0.030045607 | -3.374057117 |
| SH3PXD2B | 1.197559974 | 6.596449169 | 2.724201724 | 0.011355007 | 0.030094394 | -3.375742095 |
| TATDN3 | -1.144110986 | 5.353156828 | -2.722211741 | 0.01140811 | 0.030216457 | -3.380041196 |
| CYP1B1 | 1.417523663 | 12.34940905 | 2.72145835 | 0.011428276 | 0.030263636 | -3.38166835 |
| HIST1H3I | 1.150314669 | 6.117483789 | 2.718523988 | 0.011507136 | 0.03045366 | -3.388003549 |
| NOXA1 | 1.185012197 | 8.00877825 | 2.718143578 | 0.011517397 | 0.030468507 | -3.388824568 |
| MORC2-AS1 | -1.235636872 | 4.959971451 | -2.718140354 | 0.011517484 | 0.030468507 | -3.388831524 |
| PALLD | -1.296363087 | 11.92120213 | -2.71795538 | 0.011522477 | 0.030475446 | -3.389230721 |
| GUCY2GP | 2.81914454 | 7.704789901 | 2.717641605 | 0.01153095 | 0.030491588 | -3.389907853 |
| STAB1 | 1.114934568 | 8.053393661 | 2.715532011 | 0.011588072 | 0.03063004 | -3.394459265 |
| POLR1C | -1.098485334 | 4.714801565 | -2.713826107 | 0.011634456 | 0.030740012 | -3.398138301 |
| CD55 | -1.433835329 | 8.940098715 | -2.712720308 | 0.011664617 | 0.030788472 | -3.400522439 |
| ST8SIA2 | 1.585417062 | 7.196936521 | 2.710596634 | 0.011722745 | 0.030922467 | -3.405099647 |
| CRLF1 | 1.857143061 | 9.488700803 | 2.71042282 | 0.011727515 | 0.030923165 | -3.405474185 |
| TNF | 1.150915776 | 5.281570634 | 2.710411764 | 0.011727818 | 0.030923165 | -3.405498009 |
| LMO7 | -1.195206033 | 8.362521174 | -2.709533226 | 0.011751955 | 0.030971173 | -3.407390893 |
| FXYD4 | 1.620240202 | 7.181042401 | 2.708370062 | 0.011783982 | 0.031045797 | -3.409896508 |
| TMEM204 | -1.576942962 | 11.44691347 | -2.708012615 | 0.011793841 | 0.031065407 | -3.410666378 |
| WDR25 | 1.695225767 | 7.42526395 | 2.707188047 | 0.011816613 | 0.031112645 | -3.412442122 |
| STARD10 | -1.24707157 | 6.789114386 | -2.705929716 | 0.011851443 | 0.031197966 | -3.415151416 |
| AGFG1 | -1.517194311 | 7.972835432 | -2.704675263 | 0.011886262 | 0.031276821 | -3.417851667 |
| TBC1D5 | -1.199589986 | 6.842397413 | -2.703892103 | 0.011908048 | 0.031321332 | -3.419537091 |
| SMAD9 | 1.329388453 | 8.815532138 | 2.701261881 | 0.011981491 | 0.031495184 | -3.425195569 |
| NEMF | -1.58227162 | 9.361642488 | -2.701094903 | 0.011986167 | 0.031501039 | -3.425554691 |
| LOC729164 | 1.737303593 | 8.365763708 | 2.700799481 | 0.011994446 | 0.031516356 | -3.426190031 |
| SYT1 | 1.78454281 | 6.592979421 | 2.700356426 | 0.012006871 | 0.031533884 | -3.427142797 |
| OPRM1 | 2.755193248 | 6.679501332 | 2.700351606 | 0.012007007 | 0.031533884 | -3.427153162 |
| LOC646938 | 1.009882404 | 5.723878892 | 2.700240298 | 0.01201013 | 0.031533884 | -3.42739251 |
| CENPF | 1.618621986 | 7.452808104 | 2.700212038 | 0.012010923 | 0.031533884 | -3.427453278 |
| IQSEC1 | 1.150572373 | 8.158342507 | 2.69914949 | 0.012040781 | 0.031605823 | -3.429737812 |
| FAM83A | 2.235883571 | 6.161788042 | 2.698171853 | 0.012068315 | 0.03166517 | -3.431839341 |
| LTA | 1.731807484 | 6.119091182 | 2.696824512 | 0.012106357 | 0.031752032 | -3.434734895 |
| C15orf50 | 1.369957321 | 4.803746215 | 2.696669011 | 0.012110755 | 0.03175709 | -3.435069029 |
| ERVH-3 | 1.588239967 | 6.440943884 | 2.696490538 | 0.012115804 | 0.031763855 | -3.435452511 |
| TP53I3 | -1.880439771 | 6.555844883 | -2.695995941 | 0.012129807 | 0.031794087 | -3.436515168 |
| PPY | 1.639301773 | 7.256844137 | 2.695676814 | 0.012138851 | 0.031811308 | -3.437200765 |
| TM6SF1 | 1.186887434 | 7.782956852 | 2.695556323 | 0.012142267 | 0.031813778 | -3.43745961 |
| MAGED1 | -1.482489026 | 9.018498527 | -2.695090033 | 0.012155496 | 0.031841952 | -3.438461258 |
| CRADD | 1.363226687 | 10.55964035 | 2.694432079 | 0.012174184 | 0.031877924 | -3.439874463 |
| WDR63 | -1.742773314 | 4.836804576 | -2.694105104 | 0.012183482 | 0.031895776 | -3.440576691 |
| FBF1 | -1.194112945 | 4.951790099 | -2.69361131 | 0.012197536 | 0.03192607 | -3.441637102 |
| C11orf30 | -1.127098999 | 5.060818881 | -2.693144234 | 0.012210843 | 0.031954398 | -3.442640036 |
| DENND1C | 1.391606469 | 8.458551109 | 2.69304772 | 0.012213595 | 0.031955097 | -3.442847266 |
| LENG9 | 1.211332545 | 5.905227758 | 2.690309034 | 0.012291913 | 0.032146926 | -3.448725874 |
| LOC100133039 | 1.132783676 | 6.96030536 | 2.690189274 | 0.012295348 | 0.032149374 | -3.448982865 |
| FIZ1 | -1.45989414 | 4.79156797 | -2.689431329 | 0.012317112 | 0.03218665 | -3.450609169 |
| UNC5C | -1.508635198 | 4.73803334 | -2.6867496 | 0.012394405 | 0.032368901 | -3.456361244 |
| NOS1 | 2.264767515 | 7.6538055 | 2.686375683 | 0.012405218 | 0.032389406 | -3.457163007 |
| LOC100288721 | 1.816352431 | 5.971106915 | 2.685760771 | 0.012423019 | 0.032423879 | -3.458481387 |
| MATN2 | -1.2547388 | 5.670120452 | -2.684491012 | 0.012459854 | 0.032506825 | -3.461203231 |
| LONP1 | -1.013182898 | 8.669402745 | -2.683810981 | 0.012479624 | 0.032537546 | -3.462660642 |
| HBA2 | 1.981243485 | 13.37911369 | 2.683396001 | 0.012491702 | 0.032543705 | -3.463549909 |
| C2orf3 | 1.106364046 | 8.124270527 | 2.68276544 | 0.012510076 | 0.032575618 | -3.464900999 |
| PAPD7 | -1.538585149 | 7.255571079 | -2.682510057 | 0.012517525 | 0.032575618 | -3.465448151 |
| TUBA1C | 1.029574037 | 11.79223351 | 2.682505304 | 0.012517664 | 0.032575618 | -3.465458334 |
| HEATR5A | -1.076482227 | 6.415688352 | -2.682454434 | 0.012519148 | 0.032575618 | -3.465567319 |
| SLC29A2 | 1.710568852 | 7.433569155 | 2.682085424 | 0.01252992 | 0.032597052 | -3.466357856 |
| MSLN | -1.943010378 | 6.135133597 | -2.680093243 | 0.012588223 | 0.032742108 | -3.470624687 |
| DNHD1 | 1.074006579 | 6.159860904 | 2.6789189 | 0.01262271 | 0.032811904 | -3.47313905 |
| SLC17A4 | 1.536256473 | 7.033974265 | 2.677584045 | 0.012662019 | 0.032898556 | -3.475996332 |
| WDR38 | 1.67065382 | 8.775809514 | 2.677458448 | 0.012665723 | 0.032898556 | -3.476265133 |
| VGLL2 | 1.845359668 | 6.429181386 | 2.677439698 | 0.012666277 | 0.032898556 | -3.476305262 |
| MT1L | 1.694574201 | 12.85351371 | 2.677256552 | 0.01267168 | 0.032905947 | -3.476697216 |
| RBP4 | -1.873890114 | 7.089524733 | -2.677116459 | 0.012675815 | 0.03291004 | -3.476997019 |
| SLC22A7 | 3.334865675 | 7.358844521 | 2.676593335 | 0.012691267 | 0.032943508 | -3.47811645 |
| VKORC1 | -1.111259794 | 8.877155823 | -2.676260172 | 0.012701117 | 0.032955774 | -3.478829318 |
| CYC1 | -1.740604036 | 5.996123021 | -2.676038718 | 0.012707669 | 0.032966122 | -3.479303136 |
| KAT2A | 1.269828793 | 10.1306092 | 2.675520888 | 0.012723 | 0.032999239 | -3.480410989 |
| POLR3H | -1.311868248 | 8.570951059 | -2.674455526 | 0.012754597 | 0.033067854 | -3.482689857 |
| WDTC1 | 1.448079865 | 7.72692796 | 2.6730257 | 0.012797118 | 0.033151366 | -3.485747535 |
| FEV | 1.931050889 | 6.51402984 | 2.67184163 | 0.012832431 | 0.033234185 | -3.488278968 |
| LOC100128288 | 1.691466445 | 6.690148981 | 2.671780545 | 0.012834256 | 0.033234185 | -3.488409545 |
| MMS19 | -1.309447338 | 7.877808736 | -2.671468881 | 0.012843567 | 0.033251602 | -3.489075741 |
| ATP6V1B2 | -1.144741143 | 8.159579173 | -2.670937353 | 0.012859462 | 0.03327266 | -3.490211806 |
| ZBTB17 | -1.024974664 | 6.148546304 | -2.670606597 | 0.012869362 | 0.033291579 | -3.490918684 |
| UCN3 | 2.208212507 | 6.432659859 | 2.670365815 | 0.012876573 | 0.033300266 | -3.491433243 |
| KDM5C | 1.315079913 | 9.825031014 | 2.669284694 | 0.012909 | 0.033366858 | -3.493743312 |
| SEZ6L | 1.38379539 | 5.74771926 | 2.668908724 | 0.012920294 | 0.03338305 | -3.494546538 |
| ZAP70 | 1.433110962 | 7.831614313 | 2.668785569 | 0.012923996 | 0.033385908 | -3.494809634 |
| METRNL | 1.261073365 | 11.40382753 | 2.668676454 | 0.012927277 | 0.033387677 | -3.495042729 |
| LGI3 | 1.318756952 | 7.177714103 | 2.667405707 | 0.012965539 | 0.033479776 | -3.497756957 |
| TDRD3 | -1.264759009 | 4.67954372 | -2.666923507 | 0.012980086 | 0.033510611 | -3.498786712 |
| IARS | -1.685775281 | 7.794304896 | -2.666262354 | 0.013000056 | 0.033555433 | -3.500198458 |
| KCTD2 | 1.50311886 | 7.274871924 | 2.665365465 | 0.013027193 | 0.033618731 | -3.502113251 |
| ATCAY | 1.382735496 | 6.23836216 | 2.665088618 | 0.01303558 | 0.033633627 | -3.502704226 |
| FAM59A | -1.082917244 | 7.072290615 | -2.66477014 | 0.013045234 | 0.033651786 | -3.503384027 |
| PTPRE | 1.14004965 | 10.40129718 | 2.664336997 | 0.013058376 | 0.033678931 | -3.504308509 |
| SLC39A3 | 1.024555493 | 6.41182995 | 2.66308193 | 0.013096524 | 0.033760426 | -3.506986792 |
| GPR45 | 2.702003424 | 7.043083919 | 2.66190746 | 0.013132316 | 0.033842487 | -3.509492438 |
| FAM123C | 1.174005294 | 5.377230125 | 2.659755046 | 0.01319815 | 0.033978104 | -3.514082837 |
| LCE4A | 2.001747175 | 6.809574658 | 2.659478355 | 0.013206635 | 0.033980047 | -3.514672776 |
| OR8H1 | 1.107769626 | 5.031622989 | 2.658445692 | 0.013238349 | 0.034040712 | -3.516874237 |
| EPB42 | 1.648381407 | 5.412962502 | 2.657851184 | 0.013256639 | 0.034080929 | -3.518141404 |
| AMIGO2 | -1.280976298 | 7.844376622 | -2.657600595 | 0.013264355 | 0.034087698 | -3.518675477 |
| FKBP9 | -1.357999648 | 10.4029081 | -2.657593546 | 0.013264572 | 0.034087698 | -3.5186905 |
| ZNF642 | -1.379415825 | 4.418708361 | -2.657467153 | 0.013268466 | 0.034090893 | -3.518959865 |
| TSEN34 | -1.254867158 | 10.94343596 | -2.657031742 | 0.013281887 | 0.034106237 | -3.51988775 |
| C15orf26 | -1.838106658 | 4.979752277 | -2.656104133 | 0.013310523 | 0.034171646 | -3.521864245 |
| REEP2 | 1.362169089 | 6.846180321 | 2.655572004 | 0.013326976 | 0.034207058 | -3.522997898 |
| ANKRD36BP2 | 1.209569056 | 9.905471033 | 2.654793489 | 0.013351082 | 0.034257775 | -3.524656221 |
| ABCD3 | -2.360690162 | 4.817723515 | -2.654761842 | 0.013352062 | 0.034257775 | -3.524723628 |
| ACR | 1.320530281 | 7.223864947 | 2.654156314 | 0.013370841 | 0.034299114 | -3.526013271 |
| RAB3D | -1.24660171 | 9.641304232 | -2.653498244 | 0.013391277 | 0.034344687 | -3.527414628 |
| FUT7 | 1.243463963 | 5.485666783 | 2.651170175 | 0.013463809 | 0.034516338 | -3.532370663 |
| CNN2 | 1.019546408 | 11.45111152 | 2.651091846 | 0.013466256 | 0.034516338 | -3.532537368 |
| SOX5 | -1.289024403 | 4.979181689 | -2.650468225 | 0.01348575 | 0.034559419 | -3.533864502 |
| NXNL1 | 1.580684227 | 5.02194923 | 2.650227471 | 0.013493284 | 0.03456634 | -3.534376805 |
| LPIN3 | 1.242489294 | 5.324622669 | 2.650210135 | 0.013493826 | 0.03456634 | -3.534413694 |
| IFI6 | 1.352319973 | 11.69014582 | 2.649783674 | 0.01350718 | 0.034586771 | -3.535321097 |
| LDLRAD1 | -2.25413975 | 5.909453575 | -2.649661884 | 0.013510996 | 0.034589656 | -3.535580221 |
| HIVEP3 | 1.037627823 | 8.22823247 | 2.649241804 | 0.013524166 | 0.034616482 | -3.536473943 |
| SERPINA9 | 1.538412186 | 5.79519352 | 2.648341326 | 0.013552437 | 0.034681943 | -3.538389442 |
| CLEC18B | 1.31786093 | 7.439400757 | 2.647433865 | 0.013580983 | 0.03474117 | -3.540319422 |
| BTN2A2 | 1.318955424 | 8.718057992 | 2.646494464 | 0.013610593 | 0.034797032 | -3.542316934 |
| TST | -1.179817194 | 10.65122966 | -2.646483566 | 0.013610937 | 0.034797032 | -3.542340105 |
| LCN2 | -1.791372519 | 6.573252042 | -2.64529725 | 0.013648417 | 0.034865131 | -3.544862069 |
| PTPRK | -1.425712166 | 9.074766377 | -2.64448487 | 0.013674138 | 0.034923901 | -3.546588717 |
| DNAJB5 | 1.280423871 | 8.969898582 | 2.6441299 | 0.013685392 | 0.034931833 | -3.547343086 |
| HLA-G | 1.235335839 | 13.07845923 | 2.643747999 | 0.013697508 | 0.034955823 | -3.54815462 |
| TMEM179B | -1.117630544 | 7.251728241 | -2.642417742 | 0.013739792 | 0.035056775 | -3.550980875 |
| CREB3L2 | 1.516958725 | 9.365351895 | 2.641913009 | 0.013755867 | 0.035083872 | -3.552053017 |
| SPRED2 | -1.118779326 | 5.315399195 | -2.640686533 | 0.013795003 | 0.035155801 | -3.554657779 |
| CDX4 | 3.073902912 | 6.723931972 | 2.640141664 | 0.013812422 | 0.035187724 | -3.555814738 |
| ZNF658 | -1.11614252 | 5.845324954 | -2.640039022 | 0.013815706 | 0.035187724 | -3.55603267 |
| LOC389043 | 2.199078761 | 4.993363047 | 2.639592512 | 0.01383 | 0.035217077 | -3.556980654 |
| RAB11FIP1 | -1.290116298 | 8.551389192 | -2.638463487 | 0.013866203 | 0.035302277 | -3.559377276 |
| TNAP | -1.255721709 | 5.992434012 | -2.638240481 | 0.013873365 | 0.03531352 | -3.559850589 |
| MAP9 | -1.144552228 | 7.579468898 | -2.637973607 | 0.013881939 | 0.035328354 | -3.560416979 |
| MFRP | 1.208118085 | 6.362857607 | 2.63771027 | 0.013890405 | 0.035337037 | -3.560975831 |
| LOC730091 | -1.410241368 | 5.367367207 | -2.637591071 | 0.013894239 | 0.035338679 | -3.561228783 |
| NCS1 | 1.919253292 | 7.592757709 | 2.637239823 | 0.013905541 | 0.035360434 | -3.561974131 |
| CCNDBP1 | -1.391986376 | 10.07113178 | -2.635964943 | 0.013946637 | 0.035450917 | -3.564678943 |
| HLA-F-AS1 | -1.345909837 | 7.17480645 | -2.635588212 | 0.013958802 | 0.035467822 | -3.565478082 |
| ITGAM | 2.767147598 | 12.41451567 | 2.635325107 | 0.013967304 | 0.035475409 | -3.566036153 |
| KCNJ14 | 1.164004694 | 7.044611266 | 2.635126247 | 0.013973734 | 0.035484731 | -3.566457932 |
| P4HB | 1.200598501 | 13.68799721 | 2.634895635 | 0.013981193 | 0.035495027 | -3.566947035 |
| ATXN1L | 2.050506715 | 7.010394283 | 2.634400885 | 0.013997209 | 0.035523303 | -3.567996263 |
| DRAM2 | -1.208997768 | 8.438496387 | -2.632872407 | 0.014046796 | 0.035626024 | -3.57123703 |
| FAM124A | 1.529953823 | 7.518012049 | 2.632811861 | 0.014048763 | 0.035626024 | -3.571365381 |
| OR51D1 | 1.438186256 | 4.920684024 | 2.632381871 | 0.014062744 | 0.035647422 | -3.572276864 |
| EEF1A1 | -1.05828456 | 11.87279784 | -2.631772393 | 0.014082584 | 0.035690678 | -3.573568677 |
| CTGF | -1.739722339 | 7.700782984 | -2.63025173 | 0.014132197 | 0.035802309 | -3.576791035 |
| FOXN1 | 1.909681191 | 5.962787911 | 2.630052898 | 0.014138697 | 0.03581172 | -3.577212291 |
| RXRA | -1.158146232 | 8.577750252 | -2.629609452 | 0.014153201 | 0.035841401 | -3.578151732 |
| PCLO | 1.384545121 | 7.260589826 | 2.628688398 | 0.014183373 | 0.035910737 | -3.580102699 |
| PRRC1 | -1.026496616 | 7.266339441 | -2.627350815 | 0.014227296 | 0.03599131 | -3.582935252 |
| MGC14436 | 1.45542692 | 5.342560653 | 2.627295446 | 0.014229117 | 0.03599131 | -3.583052489 |
| DDB2 | 1.522972608 | 10.09069302 | 2.627293307 | 0.014229188 | 0.03599131 | -3.583057018 |
| FAM162A | -1.008173413 | 10.64522303 | -2.627164233 | 0.014233433 | 0.035994971 | -3.583330306 |
| CADM1 | -1.468404614 | 10.34260631 | -2.626881636 | 0.014242733 | 0.036011408 | -3.583928623 |
| C7orf31 | -1.739300152 | 5.123271145 | -2.625796555 | 0.014278494 | 0.036094731 | -3.586225619 |
| CXorf65 | 1.04192874 | 6.072293537 | 2.624781368 | 0.014312027 | 0.036172391 | -3.588374165 |
| CASQ1 | 2.060692101 | 7.580829506 | 2.624276164 | 0.014328743 | 0.036193471 | -3.589443204 |
| TIGD5 | -1.259492028 | 5.495944061 | -2.624274122 | 0.01432881 | 0.036193471 | -3.589447524 |
| SDSL | -1.155144408 | 6.016875982 | -2.623990595 | 0.014338199 | 0.036210075 | -3.590047429 |
| DEFB123 | 1.576657421 | 4.940787495 | 2.623686665 | 0.01434827 | 0.036228395 | -3.590690465 |
| LINC00476 | 1.813849687 | 7.136600768 | 2.622665327 | 0.01438216 | 0.036306839 | -3.592851029 |
| C20orf29 | 1.259796749 | 8.835535442 | 2.622152396 | 0.014399209 | 0.036342744 | -3.593935914 |
| LOC100507008 | -1.343733595 | 4.954859989 | -2.621840567 | 0.014409582 | 0.03636179 | -3.594595395 |
| C2orf57 | 1.646809472 | 4.579128157 | 2.621482307 | 0.014421509 | 0.036377613 | -3.595353016 |
| SRMS | 2.174543297 | 6.233537897 | 2.621364431 | 0.014425435 | 0.036379717 | -3.595602277 |
| EPS15 | 1.112899972 | 9.188782231 | 2.621022694 | 0.014436823 | 0.03639483 | -3.596324884 |
| TP53TG5 | 2.211099545 | 6.26289419 | 2.619533963 | 0.014486532 | 0.036498684 | -3.599472182 |
| SLC25A47 | 1.267589765 | 8.011814503 | 2.618989822 | 0.014504741 | 0.036523097 | -3.600622284 |
| MAFB | -1.112673784 | 9.157920048 | -2.615996031 | 0.014605306 | 0.036754734 | -3.606947529 |
| HECW2 | -1.126915315 | 6.820656348 | -2.61565248 | 0.014616888 | 0.036776684 | -3.607673112 |
| AGGF1 | -1.406862868 | 6.140492944 | -2.615209536 | 0.014631833 | 0.036807087 | -3.608608534 |
| CD8B | 1.08567819 | 7.2241425 | 2.613347513 | 0.014694814 | 0.036958291 | -3.612539807 |
| ATP2B3 | 1.409502394 | 4.704555378 | 2.612764858 | 0.014714574 | 0.036979064 | -3.61376963 |
| C16orf82 | 1.248062897 | 4.568215427 | 2.612420755 | 0.014726255 | 0.03699836 | -3.614495862 |
| SNCG | 1.401599264 | 6.741119572 | 2.612369226 | 0.014728005 | 0.03699836 | -3.614604609 |
| CBLN3 | 1.112259872 | 4.929056491 | 2.61161482 | 0.014753648 | 0.03705554 | -3.616196575 |
| CAPS | -1.951897046 | 9.747346929 | -2.611072201 | 0.014772118 | 0.037094686 | -3.617341459 |
| HLA-DPB1 | -1.636177814 | 11.38391238 | -2.610559058 | 0.014789604 | 0.037104401 | -3.618424025 |
| FAM3C | -1.416603726 | 7.866606458 | -2.610519755 | 0.014790944 | 0.037104401 | -3.618506937 |
| PADI4 | 1.533243853 | 4.983738792 | 2.610418037 | 0.014794413 | 0.037104401 | -3.618721512 |
| CHID1 | 1.038584649 | 9.888730617 | 2.61028165 | 0.014799065 | 0.037104401 | -3.619009216 |
| SPN | 1.005220171 | 8.335240119 | 2.609733066 | 0.014817792 | 0.037143672 | -3.620166342 |
| CCDC166 | 1.366048254 | 6.330606963 | 2.609584951 | 0.014822852 | 0.037143672 | -3.620478737 |
| ETV3 | 1.326394459 | 7.247610497 | 2.609567572 | 0.014823446 | 0.037143672 | -3.620515393 |
| CTAGE9 | -1.227323271 | 7.768787032 | -2.609484627 | 0.01482628 | 0.037143672 | -3.620690329 |
| GCET2 | 2.085443852 | 6.190660267 | 2.609247074 | 0.0148344 | 0.037156778 | -3.621191327 |
| SCGB3A1 | -2.535770546 | 11.92669235 | -2.608100967 | 0.014873636 | 0.037247801 | -3.623608096 |
| HLA-J | -1.509033804 | 12.6015107 | -2.607239146 | 0.014903203 | 0.037314581 | -3.625424993 |
| C2orf81 | -1.421363083 | 5.549188677 | -2.607047544 | 0.014909784 | 0.037323793 | -3.625828881 |
| UBQLN1 | 1.271249862 | 9.71032301 | 2.606570745 | 0.014926173 | 0.037357547 | -3.626833881 |
| FAM131B | 1.154042259 | 4.275715223 | 2.60614649 | 0.014940769 | 0.037386804 | -3.627728038 |
| LOC100292680 | 1.484750683 | 5.444420574 | 2.605843597 | 0.014951198 | 0.037405623 | -3.628366361 |
| CYYR1 | -1.195679205 | 6.934647102 | -2.605292917 | 0.014970176 | 0.037438539 | -3.629526766 |
| CCDC144A | 1.315544729 | 7.080689366 | 2.605006782 | 0.014980046 | 0.037451642 | -3.63012966 |
| OR2H2 | 1.693324333 | 4.683143534 | 2.604923367 | 0.014982925 | 0.037451642 | -3.63030541 |
| BTBD19 | 1.423902353 | 4.959993257 | 2.604887833 | 0.014984151 | 0.037451642 | -3.630380277 |
| PRSS53 | 1.385293727 | 4.390909581 | 2.603875498 | 0.015019128 | 0.037524481 | -3.63251293 |
| TMPRSS3 | 2.140263072 | 5.798194142 | 2.602643482 | 0.015061798 | 0.037623781 | -3.635107728 |
| F3 | -1.230270922 | 7.819933493 | -2.602377643 | 0.01507102 | 0.037639507 | -3.63566753 |
| C6orf208 | 1.669446727 | 5.602015277 | 2.602044893 | 0.015082571 | 0.037652274 | -3.636368185 |
| ZNF75D | -1.108216352 | 6.53608944 | -2.601977405 | 0.015084914 | 0.037652274 | -3.636510285 |
| IMPAD1 | -1.034655053 | 5.454590096 | -2.601485983 | 0.01510199 | 0.037687582 | -3.637544935 |
| RAB26 | 1.234559319 | 5.097254134 | 2.601326753 | 0.015107527 | 0.037692507 | -3.637880157 |
| ZFP36 | -1.519807929 | 12.25764566 | -2.601228275 | 0.015110952 | 0.037692507 | -3.638087475 |
| CCNH | -1.284656184 | 7.70746646 | -2.601176445 | 0.015112755 | 0.037692507 | -3.638196585 |
| C19orf20 | -1.144266009 | 4.284410116 | -2.600891568 | 0.015122669 | 0.03770992 | -3.638796279 |
| MYL5 | -1.707153529 | 5.275835123 | -2.600802619 | 0.015125766 | 0.037710331 | -3.638983518 |
| ATP2C1 | -1.005952633 | 8.974685116 | -2.600656421 | 0.015130857 | 0.037713038 | -3.639291257 |
| GRIA2 | 2.284661004 | 5.479007545 | 2.600603042 | 0.015132716 | 0.037713038 | -3.639403615 |
| ZNF430 | -1.240697424 | 10.49735303 | -2.600477495 | 0.015137089 | 0.03771663 | -3.639667875 |
| CCDC142 | -1.186491948 | 5.477335193 | -2.600128203 | 0.015149264 | 0.037739654 | -3.640403047 |
| POLI | -1.236080578 | 6.730864462 | -2.599407596 | 0.015174409 | 0.037787657 | -3.641919567 |
| BNIP1 | -1.155478272 | 5.576958266 | -2.598777216 | 0.015196438 | 0.037835188 | -3.643246001 |
| CCDC120 | 1.068533655 | 6.294310813 | 2.598653476 | 0.015200765 | 0.037838639 | -3.643506352 |
| PRKCI | -1.142447068 | 7.913343125 | -2.598181121 | 0.015217296 | 0.037872457 | -3.644500123 |
| BAK1 | 1.029362885 | 7.25284278 | 2.596785513 | 0.015266234 | 0.037975558 | -3.647435682 |
| KLK15 | 1.679230856 | 5.391498456 | 2.596747193 | 0.01526758 | 0.037975558 | -3.647516272 |
| LOC645431 | 1.569155149 | 6.014187263 | 2.595128158 | 0.015324541 | 0.03810987 | -3.650920615 |
| PKIA | -1.559206012 | 7.334120501 | -2.594746788 | 0.015337987 | 0.038128564 | -3.65172234 |
| ZFPL1 | -1.005446013 | 4.884683051 | -2.593867477 | 0.015369031 | 0.038198354 | -3.653570591 |
| HAR1A | 1.204851318 | 6.65784975 | 2.593631096 | 0.015377387 | 0.038211737 | -3.654067386 |
| GPR144 | 1.283098332 | 6.115027143 | 2.593511085 | 0.01538163 | 0.038214899 | -3.654319597 |
| MYOZ3 | 1.051364107 | 6.712115904 | 2.591578766 | 0.015450109 | 0.038370208 | -3.658379591 |
| SPRR2D | 1.548726494 | 5.949084913 | 2.591458453 | 0.015454382 | 0.03837341 | -3.658632323 |
| C11orf51 | -1.177013948 | 5.724865086 | -2.591151623 | 0.015465285 | 0.038384556 | -3.659276823 |
| OR1N2 | 1.547461202 | 5.303409749 | 2.589248315 | 0.015533075 | 0.038536074 | -3.663273753 |
| MAGIX | -1.013742336 | 4.23349032 | -2.58919792 | 0.015534873 | 0.038536074 | -3.663379557 |
| IL32 | 1.25285062 | 10.2325291 | 2.58796027 | 0.015579108 | 0.03863835 | -3.665977664 |
| SPTAN1 | -1.257743335 | 9.598649168 | -2.587467225 | 0.015596762 | 0.038674678 | -3.667012474 |
| ASF1A | -1.210368779 | 7.135003491 | -2.587073701 | 0.015610866 | 0.03870219 | -3.667838324 |
| MLL3 | 1.004902853 | 11.04645558 | 2.586823537 | 0.015619838 | 0.038716971 | -3.668363282 |
| CD83 | -1.268993856 | 8.280945047 | -2.586235899 | 0.015640933 | 0.038761689 | -3.669596296 |
| FAM101A | 2.009760512 | 7.723371271 | 2.586135376 | 0.015644544 | 0.038761689 | -3.669807202 |
| HEPACAM | 2.359933839 | 6.644007741 | 2.586069249 | 0.01564692 | 0.038761689 | -3.669945939 |
| OR51Q1 | 2.508403248 | 5.899310443 | 2.585748459 | 0.015658451 | 0.038782784 | -3.670618945 |
| COMMD8 | -1.04573557 | 8.567927035 | -2.58526619 | 0.015675801 | 0.038818282 | -3.671630633 |
| A2M | -1.71464217 | 14.1987354 | -2.584558774 | 0.015701283 | 0.038866418 | -3.673114428 |
| GGTA1P | -1.405444826 | 7.60369464 | -2.584016014 | 0.015720861 | 0.038904891 | -3.6742527 |
| PRPF3 | -1.10291921 | 6.170204941 | -2.583837889 | 0.015727291 | 0.038908331 | -3.674626232 |
| UROS | -1.060079821 | 5.170585707 | -2.582159136 | 0.01578801 | 0.039021019 | -3.678145871 |
| DDIT4 | -1.541004597 | 11.07927874 | -2.580082753 | 0.015863413 | 0.039184793 | -3.68249732 |
| HNRNPD | -1.236442886 | 9.328476568 | -2.579648028 | 0.015879242 | 0.03921261 | -3.683408108 |
| BTNL9 | -1.288584267 | 7.350225361 | -2.579293121 | 0.015892176 | 0.039233237 | -3.684151603 |
| IL26 | 1.332584349 | 4.733656248 | 2.577692049 | 0.015950645 | 0.039370025 | -3.687504946 |
| TEKT4 | -1.26086548 | 5.115604891 | -2.576913034 | 0.015979166 | 0.039432854 | -3.689136102 |
| ADRA1B | -1.636401123 | 7.464843218 | -2.576493823 | 0.015994534 | 0.039463206 | -3.690013756 |
| WDR93 | 1.480479383 | 7.033873307 | 2.576406093 | 0.015997752 | 0.039463575 | -3.690197416 |
| NFKBIL1 | 1.598939924 | 6.209548039 | 2.575722415 | 0.016022848 | 0.039510329 | -3.691628546 |
| PTPN12 | -2.253848376 | 10.0694611 | -2.575590286 | 0.016027703 | 0.039514724 | -3.691905103 |
| NME3 | 1.007537695 | 11.62739176 | 2.575160988 | 0.016043485 | 0.039546052 | -3.692803602 |
| GPR132 | 1.9120536 | 7.111378523 | 2.574823317 | 0.016055909 | 0.039569093 | -3.69351027 |
| FZD10 | -1.009506745 | 6.746706966 | -2.573623186 | 0.016100137 | 0.039655296 | -3.696021425 |
| C1orf177 | 1.389505562 | 4.946623951 | 2.573524908 | 0.016103764 | 0.039656635 | -3.696227033 |
| PTCH1 | 2.331563331 | 10.16863365 | 2.573258573 | 0.016113596 | 0.039673253 | -3.696784205 |
| C12orf45 | -1.289615409 | 7.179107943 | -2.571538892 | 0.016177218 | 0.039814653 | -3.700380972 |
| ST6GALNAC1 | -1.291301325 | 6.533201674 | -2.571390039 | 0.016182736 | 0.039820614 | -3.700692235 |
| TRPM5 | 1.79721738 | 6.666110163 | 2.571145842 | 0.016191792 | 0.039835277 | -3.701202849 |
| C7orf34 | 1.607087683 | 6.440437351 | 2.570803708 | 0.016204488 | 0.03985169 | -3.7019182 |
| C1orf53 | -1.473513106 | 5.800912654 | -2.570799082 | 0.01620466 | 0.03985169 | -3.701927872 |
| EMILIN3 | 1.409453691 | 6.514787608 | 2.569927163 | 0.016237058 | 0.039923731 | -3.703750667 |
| ZNF747 | 1.764701165 | 7.131897736 | 2.569117121 | 0.016267211 | 0.039990226 | -3.70544378 |
| GTDC1 | -1.39945753 | 7.248067592 | -2.568518919 | 0.016289511 | 0.040037396 | -3.706693913 |
| IGDCC3 | 1.461393241 | 6.163369358 | 2.568076811 | 0.016306011 | 0.040062639 | -3.707617729 |
| COL10A1 | 1.718733222 | 7.076342208 | 2.567624034 | 0.016322925 | 0.040096536 | -3.708563739 |
| C1orf170 | 1.057710786 | 5.228694942 | 2.566272366 | 0.016373516 | 0.040213129 | -3.711387269 |
| ST6GALNAC5 | -1.07418979 | 6.815410447 | -2.566149648 | 0.016378116 | 0.040216748 | -3.711643573 |
| ARHGEF4 | -1.287901955 | 7.709882912 | -2.56546808 | 0.016403688 | 0.040271851 | -3.713066943 |
| KIAA1467 | -1.098869894 | 4.494328264 | -2.564626007 | 0.016435332 | 0.04033058 | -3.714825199 |
| FAM189A1 | 1.376262029 | 7.665511654 | 2.564581245 | 0.016437016 | 0.04033058 | -3.714918653 |
| TOMM7 | -1.053333343 | 13.42701323 | -2.56431294 | 0.016447112 | 0.040341021 | -3.715478801 |
| SUN5 | 2.292118221 | 6.514682731 | 2.564301453 | 0.016447544 | 0.040341021 | -3.715502782 |
| PBXIP1 | -1.308851403 | 7.098566477 | -2.564214652 | 0.016450812 | 0.040341342 | -3.71568399 |
| PLEKHG2 | 1.319584984 | 8.953051757 | 2.564112792 | 0.016454647 | 0.040343055 | -3.715896632 |
| MAPK8 | -1.220945393 | 6.686970911 | -2.563097524 | 0.016492917 | 0.040421476 | -3.718015817 |
| APOM | -1.320927143 | 5.570474406 | -2.562085577 | 0.016531145 | 0.040507447 | -3.720127579 |
| AQP5 | 1.349609789 | 10.16198539 | 2.561454865 | 0.016555013 | 0.040558205 | -3.72144352 |
| CPA4 | 1.735842021 | 7.787502996 | 2.561077923 | 0.016569293 | 0.040585458 | -3.722229893 |
| LHX1 | 1.227009885 | 6.502660132 | 2.560415735 | 0.016594406 | 0.040639232 | -3.72361118 |
| ACAA2 | -1.010305838 | 7.330529639 | -2.559721072 | 0.016620789 | 0.040696095 | -3.725059981 |
| FBXO6 | 1.574164701 | 7.361522368 | 2.557079852 | 0.016721457 | 0.040919213 | -3.730566432 |
| PRRT1 | 1.567302142 | 7.343582307 | 2.555617207 | 0.016777448 | 0.041032809 | -3.733614328 |
| TTC26 | -1.403960623 | 3.913279571 | -2.55532456 | 0.016788672 | 0.041052452 | -3.734224029 |
| EI24 | -1.66332856 | 6.586334367 | -2.555165203 | 0.016794786 | 0.041053181 | -3.734556015 |
| FUS | -1.526470306 | 7.086820109 | -2.553558562 | 0.01685655 | 0.041171963 | -3.737902432 |
| SOX13 | -1.422480029 | 6.688429277 | -2.553519167 | 0.016858067 | 0.041171963 | -3.737984472 |
| ADAM6 | 1.798823088 | 5.675648498 | 2.55347016 | 0.016859954 | 0.041171963 | -3.738086526 |
| LRRC46 | -1.323557497 | 6.17287268 | -2.553263224 | 0.016867926 | 0.041183611 | -3.738517447 |
| B3GNT7 | 1.616298912 | 10.06093393 | 2.552621642 | 0.016892664 | 0.041220537 | -3.739853336 |
| CELA2A | 1.451037917 | 7.890462035 | 2.551777712 | 0.016925256 | 0.041284401 | -3.741610246 |
| SCRT1 | 1.316027919 | 5.646714746 | 2.551506437 | 0.016935745 | 0.041295383 | -3.742174919 |
| KCNC4 | 1.952049512 | 6.908176729 | 2.551495197 | 0.01693618 | 0.041295383 | -3.742198315 |
| KLK4 | 1.419194178 | 4.784353958 | 2.549800179 | 0.017001855 | 0.04142572 | -3.745725764 |
| H19 | -1.717351522 | 8.082745346 | -2.548407307 | 0.017056001 | 0.041540292 | -3.748623387 |
| LRRC20 | 1.082911758 | 8.230434828 | 2.548242932 | 0.017062401 | 0.041548013 | -3.748965278 |
| MOK | -1.308965082 | 7.10881294 | -2.547906457 | 0.01707551 | 0.041564195 | -3.749665086 |
| WBSCR17 | -1.465786671 | 4.467711952 | -2.547526353 | 0.017090329 | 0.041584528 | -3.750455569 |
| DBF4B | 1.344737939 | 6.517825519 | 2.546340167 | 0.017136653 | 0.041684316 | -3.75292197 |
| FOXA3 | 1.602784036 | 6.657135594 | 2.546310264 | 0.017137822 | 0.041684316 | -3.752984139 |
| TRIM46 | 1.174571591 | 5.098478012 | 2.546115427 | 0.017145442 | 0.041694967 | -3.753389188 |
| ZFR2 | 1.25773974 | 7.164628963 | 2.546007255 | 0.017149675 | 0.041697375 | -3.753614062 |
| MARS | 1.019225259 | 9.816335282 | 2.545588326 | 0.017166074 | 0.04172936 | -3.754484897 |
| PBK | 1.4586442 | 5.86747853 | 2.543499031 | 0.01724808 | 0.04190495 | -3.75882668 |
| RASGRP2 | 1.121114888 | 10.92773075 | 2.54260515 | 0.017283276 | 0.041970143 | -3.760683615 |
| SIGLEC7 | -1.078748846 | 5.880651595 | -2.542529776 | 0.017286247 | 0.041970143 | -3.760840179 |
| ZNF714 | -1.102791705 | 8.87469235 | -2.542486176 | 0.017287966 | 0.041970143 | -3.760930741 |
| LOXL2 | 1.037261694 | 8.634173239 | 2.541615795 | 0.017322309 | 0.042021792 | -3.762738433 |
| TCEAL5 | -1.003955243 | 7.040363261 | -2.541507355 | 0.017326593 | 0.042024257 | -3.762963628 |
| OTOF | 1.665244194 | 8.297122827 | 2.541395399 | 0.017331016 | 0.04202706 | -3.763196115 |
| TOB1 | -1.508167586 | 7.981674683 | -2.540974245 | 0.017347664 | 0.042054567 | -3.764070633 |
| LPAR3 | -1.090130974 | 4.805549205 | -2.540943034 | 0.017348898 | 0.042054567 | -3.764135436 |
| SH2B2 | 1.169269634 | 6.844206748 | 2.540815688 | 0.017353936 | 0.042058851 | -3.764399849 |
| GJA1 | -1.332476463 | 7.290054109 | -2.539968756 | 0.017387471 | 0.042132189 | -3.766158156 |
| CASP1 | -1.572016834 | 8.522360667 | -2.538835448 | 0.017432441 | 0.042225245 | -3.768510459 |
| GBP5 | 1.924563842 | 9.534026334 | 2.537530722 | 0.017484345 | 0.042319091 | -3.771217784 |
| GZMH | 1.356526048 | 9.664497748 | 2.536066134 | 0.01754278 | 0.042432375 | -3.774255837 |
| CHRNG | 1.881100865 | 5.347946485 | 2.536026971 | 0.017544345 | 0.042432375 | -3.774337058 |
| EXOC3L4 | 1.281439952 | 5.792956765 | 2.535847618 | 0.017551514 | 0.042441733 | -3.774709023 |
| SYNPO2 | -1.088341369 | 5.497670112 | -2.535758205 | 0.017555089 | 0.042442399 | -3.774894453 |
| ADH1C | -1.368828838 | 10.44776665 | -2.534885778 | 0.017590007 | 0.042518825 | -3.776703533 |
| CGN | -1.073691543 | 7.011579236 | -2.533733762 | 0.017636213 | 0.042622505 | -3.779091803 |
| SLC25A24 | -1.193214911 | 8.583594874 | -2.53331871 | 0.017652888 | 0.042646777 | -3.779952098 |
| SH3BP4 | -1.050183518 | 4.888418513 | -2.531966364 | 0.01770732 | 0.042770244 | -3.782754576 |
| ZFYVE21 | -1.18758015 | 9.679072503 | -2.531193198 | 0.01773851 | 0.042837536 | -3.784356412 |
| DMBT1 | -2.653970324 | 12.13771947 | -2.530360266 | 0.017772168 | 0.042910761 | -3.786081743 |
| CES1 | -1.01090222 | 7.83415387 | -2.528893536 | 0.017831581 | 0.04302998 | -3.789119093 |
| FHL2 | 1.065553731 | 9.9644529 | 2.528808269 | 0.01783504 | 0.043030255 | -3.789295635 |
| SLC23A3 | 1.278804886 | 5.540228065 | 2.528672977 | 0.017840531 | 0.043035429 | -3.789575742 |
| DAAM1 | -1.226417232 | 7.974275518 | -2.528364164 | 0.017853069 | 0.0430576 | -3.790215073 |
| PDK2 | 2.179148251 | 11.1563778 | 2.527921863 | 0.017871042 | 0.043092865 | -3.791130683 |
| SH3RF2 | 1.056983124 | 5.96615074 | 2.52768712 | 0.017880587 | 0.0431078 | -3.791616587 |
| FKBP5 | 1.432520401 | 10.65994585 | 2.527235435 | 0.017898967 | 0.043144025 | -3.79255147 |
| WDR66 | -1.302613868 | 5.741766857 | -2.526634234 | 0.017923459 | 0.043194965 | -3.793795662 |
| FYB | 1.025773046 | 9.424518416 | 2.526164783 | 0.017942605 | 0.043233006 | -3.794767074 |
| FAM115C | 1.127763123 | 6.610095137 | 2.525404465 | 0.017973654 | 0.043298937 | -3.796340131 |
| ZNF280B | 2.064161155 | 6.923291757 | 2.525282693 | 0.017978631 | 0.043298937 | -3.796592044 |
| SLC20A1 | 1.202604826 | 10.93859657 | 2.525247567 | 0.017980067 | 0.043298937 | -3.796664709 |
| RAB3GAP1 | 1.318035233 | 8.576192387 | 2.524977836 | 0.017991098 | 0.04331739 | -3.797222678 |
| BCDIN3D | -1.226915008 | 6.342664127 | -2.523530042 | 0.018050411 | 0.043427683 | -3.800216997 |
| WDR5B | -1.144082115 | 5.013423907 | -2.523156226 | 0.018065755 | 0.043445342 | -3.800989955 |
| FBLN2 | 1.438585694 | 8.219102786 | 2.520970791 | 0.018155703 | 0.0436254 | -3.805507504 |
| LOC730102 | -1.400468037 | 6.525151186 | -2.520955507 | 0.018156334 | 0.0436254 | -3.805539089 |
| ZNRF3 | -1.061367838 | 8.856056178 | -2.52035723 | 0.018181031 | 0.043676581 | -3.806775383 |
| RASIP1 | -1.37437637 | 9.576643155 | -2.520156219 | 0.018189335 | 0.043688372 | -3.807190717 |
| PLEKHB2 | -1.06137562 | 9.565469053 | -2.519765558 | 0.018205485 | 0.043718998 | -3.807997855 |
| ZC3H12A | 1.800626094 | 10.71605377 | 2.51892227 | 0.018240393 | 0.043778314 | -3.8097399 |
| TPP2 | 1.046110708 | 10.15885539 | 2.518840016 | 0.018243801 | 0.043778314 | -3.809909799 |
| UBA7 | -1.093962418 | 6.36707022 | -2.518027571 | 0.018277495 | 0.043850988 | -3.811587765 |
| C4orf19 | -1.411220159 | 5.260542323 | -2.517864368 | 0.018284271 | 0.043859063 | -3.811924794 |
| RNF39 | 1.006232902 | 5.676244242 | 2.517369845 | 0.018304816 | 0.043891973 | -3.812945947 |
| CLDN5 | 1.334491873 | 13.28691634 | 2.513911811 | 0.018449077 | 0.044199148 | -3.820083152 |
| PTMS | 1.183084226 | 15.02587808 | 2.513451345 | 0.018468366 | 0.04423466 | -3.821033085 |
| GABRQ | 2.843202931 | 7.363454051 | 2.510726481 | 0.018582892 | 0.044484117 | -3.826652278 |
| STK11IP | 1.27148353 | 9.670750769 | 2.508163909 | 0.018691197 | 0.04469347 | -3.831933439 |
| PGLYRP1 | 1.798066348 | 6.870317463 | 2.506916034 | 0.018744149 | 0.044792363 | -3.834503981 |
| SYT12 | 1.73276317 | 4.776042806 | 2.506888589 | 0.018745315 | 0.044792363 | -3.834560508 |
| PHKB | -1.089036103 | 8.870012766 | -2.506817549 | 0.018748334 | 0.044792363 | -3.834706823 |
| SLC29A3 | 1.102503069 | 5.649412591 | 2.506779096 | 0.018749968 | 0.044792363 | -3.834786018 |
| MAN1A1 | 1.285427285 | 8.93307854 | 2.505949325 | 0.018785265 | 0.044843369 | -3.836494811 |
| ZNF276 | 1.235783982 | 9.519169305 | 2.505434954 | 0.018807176 | 0.044887344 | -3.837553911 |
| BHLHA15 | 1.359854866 | 5.768044834 | 2.505325861 | 0.018811826 | 0.044890112 | -3.837778518 |
| INSIG1 | -1.217809039 | 8.998994108 | -2.501948589 | 0.018956313 | 0.045192974 | -3.844728935 |
| FBXW11 | -1.056565627 | 8.286008369 | -2.501601684 | 0.018971212 | 0.045220113 | -3.845442544 |
| C20orf165 | 1.291707358 | 4.954126352 | 2.50132047 | 0.018983298 | 0.045232156 | -3.846020977 |
| PLIN5 | 1.610053828 | 8.770972494 | 2.501059539 | 0.018994518 | 0.045242128 | -3.846557654 |
| CKLF | -1.272864346 | 8.267207975 | -2.499198733 | 0.019074714 | 0.045416323 | -3.850383937 |
| PCDHB13 | 1.803749716 | 8.9812822 | 2.499036318 | 0.019081729 | 0.045424615 | -3.850717821 |
| RTDR1 | -1.182997824 | 5.925364667 | -2.498440688 | 0.019107474 | 0.045477485 | -3.851942173 |
| DOCK4 | -1.281482774 | 7.403228159 | -2.497588497 | 0.019144364 | 0.045556857 | -3.853693593 |
| PTH1R | -1.036192171 | 5.779317548 | -2.497143555 | 0.019163651 | 0.045579719 | -3.854607891 |
| GNG11 | -1.122628718 | 12.89102806 | -2.49712162 | 0.019164603 | 0.045579719 | -3.854652961 |
| ITGA2 | 1.421147646 | 8.27734762 | 2.496050616 | 0.019211104 | 0.045681867 | -3.85685332 |
| NDRG4 | -1.770294335 | 9.258081661 | -2.495880235 | 0.019218511 | 0.045689655 | -3.857203312 |
| SLFNL1 | -1.469448132 | 4.758507195 | -2.495597689 | 0.0192308 | 0.045694911 | -3.857783678 |
| CAV2 | -1.295187364 | 7.080955973 | -2.493883664 | 0.019305508 | 0.045838554 | -3.861303532 |
| D2HGDH | 1.028996613 | 8.909362243 | 2.493755285 | 0.019311115 | 0.045843403 | -3.861567105 |
| CHRND | 1.428279347 | 6.432339101 | 2.492729143 | 0.019355981 | 0.045941433 | -3.863673582 |
| JMJD5 | 1.621970473 | 6.871581595 | 2.491647664 | 0.01940337 | 0.046045415 | -3.865893082 |
| LOC100507487 | -1.193144318 | 5.773459134 | -2.49154813 | 0.019407737 | 0.046047282 | -3.866097323 |
| ARHGAP25 | -1.165205006 | 7.917233825 | -2.490511855 | 0.019453256 | 0.046146769 | -3.868223452 |
| GABRG2 | 2.037208174 | 6.717303735 | 2.490149403 | 0.0194692 | 0.0461687 | -3.868966969 |
| ARSA | 1.263591116 | 8.136202697 | 2.490138498 | 0.01946968 | 0.0461687 | -3.868989338 |
| MSMB | -2.495879444 | 7.159683273 | -2.488773269 | 0.019529846 | 0.046270207 | -3.871789302 |
| C17orf74 | 1.493117222 | 5.764310703 | 2.488720114 | 0.019532192 | 0.046270207 | -3.871898301 |
| HES4 | 1.166876308 | 8.333094848 | 2.488677548 | 0.019534071 | 0.046270207 | -3.871985583 |
| NME6 | 1.513529215 | 7.908634649 | 2.487216327 | 0.019598671 | 0.046414676 | -3.874981313 |
| CYP2C19 | 1.339205075 | 5.295375459 | 2.485457597 | 0.019676685 | 0.046579995 | -3.878585561 |
| PLK1S1 | -1.213223259 | 7.056038695 | -2.485316339 | 0.019682963 | 0.046579995 | -3.87887498 |
| PMEL | 1.03225426 | 6.543507474 | 2.484731876 | 0.01970896 | 0.04662436 | -3.880072362 |
| KIAA1429 | -1.145248559 | 9.810545906 | -2.484078931 | 0.01973804 | 0.046667403 | -3.881409839 |
| ENO2 | -1.295073632 | 6.850972279 | -2.483348503 | 0.019770619 | 0.046728915 | -3.882905775 |
| LALBA | 1.110502474 | 4.077611423 | 2.482812169 | 0.019794571 | 0.046758086 | -3.884004031 |
| TM7SF3 | -1.152724832 | 8.258176995 | -2.481545633 | 0.019851242 | 0.046883341 | -3.886596952 |
| PPP1CB | -1.087208625 | 9.047483815 | -2.480186976 | 0.019912201 | 0.046985743 | -3.889377572 |
| SMA4 | -1.082415568 | 6.989368652 | -2.478918617 | 0.019969264 | 0.047110176 | -3.891972545 |
| RNF24 | 1.341987188 | 7.578418325 | 2.478240616 | 0.01999983 | 0.047164981 | -3.893359355 |
| C12orf56 | 2.224192298 | 6.344123638 | 2.47650451 | 0.020078293 | 0.047341338 | -3.896909398 |
| CLPTM1L | -1.061910683 | 8.310284812 | -2.476031794 | 0.020099706 | 0.047383141 | -3.897875757 |
| PRCD | 1.362883904 | 5.640311291 | 2.475478208 | 0.02012481 | 0.047424936 | -3.899007296 |
| HAVCR2 | 1.310553961 | 8.689922738 | 2.474387304 | 0.020174364 | 0.047533004 | -3.901236664 |
| C1orf187 | 2.06674047 | 6.861803299 | 2.472995981 | 0.020237728 | 0.047656107 | -3.904079093 |
| NR2F1 | 1.372324489 | 12.93822347 | 2.472587911 | 0.020256347 | 0.04769122 | -3.904912582 |
| ALOX15B | -1.542927946 | 7.878801492 | -2.471073558 | 0.020325582 | 0.047836712 | -3.90800493 |
| PMPCB | -1.013664327 | 10.13653445 | -2.47010907 | 0.020369791 | 0.047923221 | -3.909973834 |
| STOX1 | -1.127342198 | 5.818309305 | -2.469184814 | 0.020412239 | 0.048005667 | -3.911860166 |
| PRM3 | 1.346089986 | 5.867038743 | 2.469121322 | 0.020415158 | 0.048005667 | -3.911989734 |
| WDR77 | -1.153167492 | 5.473234469 | -2.469102322 | 0.020416032 | 0.048005667 | -3.912028504 |
| KRT6A | 2.204539872 | 5.495961065 | 2.468486491 | 0.020444365 | 0.048063503 | -3.9132851 |
| HOXB5 | -1.112838214 | 7.469545685 | -2.467581398 | 0.020486073 | 0.048152754 | -3.915131582 |
| PPAPDC3 | 1.122833087 | 8.008719348 | 2.467314073 | 0.020498406 | 0.04817294 | -3.915676872 |
| SLC28A1 | 1.369523979 | 4.898751632 | 2.466195923 | 0.020550069 | 0.04826988 | -3.917957283 |
| LOC100131195 | 1.172727267 | 4.528301635 | 2.464959895 | 0.020607318 | 0.048384688 | -3.920477361 |
| ALDH1L1 | -1.339300884 | 4.250742207 | -2.46378264 | 0.020661981 | 0.048501614 | -3.922876888 |
| MCTP1 | -1.350346676 | 6.879735072 | -2.462222674 | 0.020734621 | 0.048639193 | -3.926055383 |
| C5orf46 | 1.372262477 | 4.373702886 | 2.457363229 | 0.020962413 | 0.049070653 | -3.935948754 |
| ZNF205 | 1.178746395 | 6.197764116 | 2.457241528 | 0.020968147 | 0.049070653 | -3.936196371 |
| AP3B1 | -1.537442817 | 7.333616201 | -2.457032722 | 0.020977989 | 0.049078668 | -3.936621196 |
| UPF1 | -1.551270016 | 11.36383199 | -2.456679492 | 0.020994648 | 0.049105867 | -3.93733981 |
| VPS26A | -1.60941899 | 8.269355966 | -2.453418858 | 0.021149003 | 0.049439927 | -3.943970249 |
| CCS | -1.17880397 | 5.98238435 | -2.452947471 | 0.021171404 | 0.0494833 | -3.944928356 |
| B9D1 | -1.327953339 | 5.628911705 | -2.452755295 | 0.021180543 | 0.049486675 | -3.945318927 |
| ZGPAT | 1.020881 | 9.234070541 | 2.452046498 | 0.021214281 | 0.049556499 | -3.946759293 |
| TIAM1 | 1.517785638 | 9.354166328 | 2.451790244 | 0.021226491 | 0.049576017 | -3.947279969 |
| DAAM2 | 1.227503273 | 10.2188099 | 2.45161498 | 0.021234845 | 0.049586525 | -3.947636065 |
| DUS3L | 1.077007884 | 6.431735848 | 2.450601753 | 0.021283203 | 0.049681408 | -3.9496944 |
| DUSP6 | -1.490397019 | 10.91961073 | -2.450018491 | 0.021311086 | 0.049728445 | -3.950879036 |
| GTPBP4 | -1.262605262 | 7.71761502 | -2.449716101 | 0.021325555 | 0.049753182 | -3.951493138 |
