# Supplementary material for: Identification of SLC15A3 as a shared prognostic biomarker for AML and COVID-19 via integrative bioinformatics, machine learning and Mendelian randomization
Source: Front Oncol. 2026 Oct 14;16:1911550. doi: 10.3389/fonc.2026.1911550 (PMC13713349; doi:10.3389/fonc.2026.1911550)
Supplement: Supplementary file 2 [file Table2.doc]

**Supplementary Table S2 The differentially expressed mRNAs of GSEA**

| id | logFC | lfcSE | pvalue | padj |
| --- | --- | --- | --- | --- |
| IFI6 | 6.677475021 | 0.484675766 | 3.50E-43 | 3.67E-39 |
| CLEC4E | 7.220102319 | 0.532553509 | 7.15E-42 | 3.76E-38 |
| FFAR2 | 6.696786636 | 0.572414022 | 1.29E-31 | 4.50E-28 |
| FDCSP | 8.93497864 | 0.798969248 | 4.93E-29 | 1.29E-25 |
| ST20 | 7.327976626 | 0.667206284 | 4.61E-28 | 9.68E-25 |
| ALOX5AP | 5.744300773 | 0.533248192 | 4.65E-27 | 8.14E-24 |
| DAPP1 | 5.733348471 | 0.541181474 | 3.17E-26 | 4.76E-23 |
| SELL | 7.543971337 | 0.719225193 | 9.70E-26 | 1.27E-22 |
| STAP1 | 6.609513914 | 0.641932692 | 7.32E-25 | 8.11E-22 |
| RNASE2 | 8.651036873 | 0.840629259 | 7.73E-25 | 8.11E-22 |
| CASP5 | 6.27569213 | 0.61598106 | 2.24E-24 | 2.14E-21 |
| FCER1G | 6.293400784 | 0.621920916 | 4.54E-24 | 3.97E-21 |
| CCL8 | 6.336748391 | 0.665623668 | 1.73E-21 | 1.40E-18 |
| PLEK | 5.965629372 | 0.62897959 | 2.43E-21 | 1.82E-18 |
| BCL2A1 | 5.70453056 | 0.622955016 | 5.33E-20 | 3.73E-17 |
| MX1 | 4.487496735 | 0.495682807 | 1.39E-19 | 9.12E-17 |
| OAS1 | 5.778695045 | 0.643908543 | 2.85E-19 | 1.76E-16 |
| CYP19A1 | 7.40473325 | 0.837014096 | 9.02E-19 | 5.26E-16 |
| LILRA6 | 5.611713455 | 0.63677306 | 1.22E-18 | 6.75E-16 |
| IFIT1 | 5.825044562 | 0.671028367 | 3.93E-18 | 2.06E-15 |
| OLR1 | 5.789694107 | 0.690197642 | 4.93E-17 | 2.46E-14 |
| TNFSF10 | 4.214109982 | 0.502949937 | 5.35E-17 | 2.55E-14 |
| MXD1 | 4.640085616 | 0.555771773 | 6.89E-17 | 3.15E-14 |
| SP140 | 5.335153996 | 0.641050436 | 8.61E-17 | 3.77E-14 |
| EIF2AK2 | 5.465168599 | 0.667043332 | 2.55E-16 | 1.07E-13 |
| TREM1 | 4.240284726 | 0.518345785 | 2.83E-16 | 1.12E-13 |
| IFIT3 | 5.330357535 | 0.652095193 | 2.98E-16 | 1.12E-13 |
| CCL11 | 5.573268067 | 0.681876235 | 3.00E-16 | 1.12E-13 |
| P2RY13 | 6.473980762 | 0.794600477 | 3.72E-16 | 1.35E-13 |
| IL2RG | 6.02418252 | 0.751033516 | 1.05E-15 | 3.67E-13 |
| HSH2D | 4.648619632 | 0.580180459 | 1.13E-15 | 3.81E-13 |
| TREML3P | 5.447243842 | 0.682215081 | 1.41E-15 | 4.62E-13 |
| DDX60L | 4.918380669 | 0.616822078 | 1.54E-15 | 4.90E-13 |
| FOS | -5.30919798 | 0.679621513 | 5.63E-15 | 1.70E-12 |
| ISG15 | 4.793468715 | 0.613641437 | 5.65E-15 | 1.70E-12 |
| IFITM2 | 5.525559448 | 0.708973197 | 6.50E-15 | 1.87E-12 |
| FYB | 4.256581825 | 0.546285227 | 6.60E-15 | 1.87E-12 |
| CPLX2 | 6.555787694 | 0.854998713 | 1.75E-14 | 4.84E-12 |
| EMR1 | 4.710662771 | 0.61781122 | 2.44E-14 | 6.58E-12 |
| NT5C3 | 5.186969594 | 0.682585668 | 2.98E-14 | 7.83E-12 |
| IFITM1 | 4.40410048 | 0.581447478 | 3.61E-14 | 9.24E-12 |
| KCNJ2-AS1 | 5.382227151 | 0.719483945 | 7.39E-14 | 1.85E-11 |
| S100A11 | 3.958728701 | 0.532402911 | 1.04E-13 | 2.54E-11 |
| GPR84 | 5.381385179 | 0.731413032 | 1.87E-13 | 4.47E-11 |
| CXorf21 | 4.96136025 | 0.675406255 | 2.05E-13 | 4.77E-11 |
| IGSF6 | 5.293261347 | 0.724478617 | 2.75E-13 | 6.27E-11 |
| CLEC4D | 5.892883608 | 0.808610126 | 3.15E-13 | 7.05E-11 |
| HESX1 | 5.728904215 | 0.787364602 | 3.44E-13 | 7.52E-11 |
| TRIM38 | 3.968299731 | 0.547158452 | 4.09E-13 | 8.76E-11 |
| PADI2 | 4.567948823 | 0.637584814 | 7.81E-13 | 1.63E-10 |
| MX2 | 5.043544598 | 0.704159456 | 7.92E-13 | 1.63E-10 |
| IFIT2 | 5.272399227 | 0.737159525 | 8.53E-13 | 1.72E-10 |
| RAC2 | 3.546640483 | 0.498585453 | 1.13E-12 | 2.24E-10 |
| GAPT | 5.638545301 | 0.797996063 | 1.60E-12 | 3.08E-10 |
| CLC | 5.583055791 | 0.790308131 | 1.61E-12 | 3.08E-10 |
| CCL4 | 6.148571204 | 0.875088123 | 2.12E-12 | 3.98E-10 |
| PLAC8 | 3.870851808 | 0.553517256 | 2.69E-12 | 4.95E-10 |
| PTK2B | 3.589922593 | 0.516071164 | 3.49E-12 | 6.33E-10 |
| BASP1 | 3.719445766 | 0.538152384 | 4.80E-12 | 8.53E-10 |
| NBN | 4.261320051 | 0.625743898 | 9.76E-12 | 1.71E-09 |
| LCP1 | 3.718795135 | 0.549116421 | 1.27E-11 | 2.18E-09 |
| C19orf66 | 3.663239613 | 0.541123122 | 1.29E-11 | 2.19E-09 |
| ZBP1 | 4.442503307 | 0.662762091 | 2.04E-11 | 3.40E-09 |
| KLK10 | 4.184694961 | 0.625286121 | 2.19E-11 | 3.60E-09 |
| TPSB2 | 4.613526962 | 0.692329264 | 2.67E-11 | 4.31E-09 |
| AQP9 | 4.972646266 | 0.755627397 | 4.68E-11 | 7.44E-09 |
| S100P | 3.789731127 | 0.579906452 | 6.36E-11 | 9.96E-09 |
| WAS | 4.625665833 | 0.710528743 | 7.51E-11 | 1.16E-08 |
| CARD16 | 4.432430848 | 0.68143732 | 7.79E-11 | 1.19E-08 |
| LGALS9 | 3.264107443 | 0.503722497 | 9.17E-11 | 1.38E-08 |
| GPR141 | 5.569486227 | 0.860019749 | 9.42E-11 | 1.39E-08 |
| TMEM71 | 4.104233721 | 0.634067893 | 9.62E-11 | 1.40E-08 |
| CLEC4A | 4.222238128 | 0.655943568 | 1.22E-10 | 1.75E-08 |
| GPX2 | 5.153063557 | 0.802551401 | 1.36E-10 | 1.92E-08 |
| CEACAM3 | 5.155094534 | 0.80400539 | 1.44E-10 | 2.01E-08 |
| OAS2 | 4.813709031 | 0.75310853 | 1.64E-10 | 2.27E-08 |
| SYN1 | 4.723802523 | 0.739582717 | 1.69E-10 | 2.31E-08 |
| C9orf66 | 5.281767108 | 0.834569244 | 2.47E-10 | 3.33E-08 |
| ZC3HAV1 | 3.200524909 | 0.509211757 | 3.27E-10 | 4.35E-08 |
| SRGN | 4.510162933 | 0.71832719 | 3.41E-10 | 4.48E-08 |
| DOK3 | 3.95846481 | 0.633731136 | 4.20E-10 | 5.45E-08 |
| TYROBP | 3.942940082 | 0.631962126 | 4.40E-10 | 5.59E-08 |
| LY96 | 4.85605745 | 0.778404539 | 4.42E-10 | 5.59E-08 |
| CCL19 | 4.049376894 | 0.654857581 | 6.27E-10 | 7.83E-08 |
| SAMD9L | 4.696179198 | 0.760833576 | 6.73E-10 | 8.31E-08 |
| SAMD9 | 4.72427163 | 0.765948849 | 6.92E-10 | 8.45E-08 |
| OAS3 | 5.068974785 | 0.824536674 | 7.86E-10 | 9.49E-08 |
| TFEC | 4.755711295 | 0.782556995 | 1.22E-09 | 1.46E-07 |
| FAM65B | 4.285239076 | 0.709477949 | 1.54E-09 | 1.82E-07 |
| LOC731424 | 3.753432269 | 0.624607418 | 1.86E-09 | 2.15E-07 |
| CD37 | 4.088989326 | 0.680427451 | 1.86E-09 | 2.15E-07 |
| IFITM3 | 3.664114561 | 0.610545353 | 1.96E-09 | 2.23E-07 |
| IFI44L | 4.234473176 | 0.706283098 | 2.03E-09 | 2.29E-07 |
| CXCL16 | 2.970445705 | 0.502592795 | 3.42E-09 | 3.82E-07 |
| RHOH | 4.721485612 | 0.80262486 | 4.04E-09 | 4.47E-07 |
| BCL2L14 | 4.680122963 | 0.796881887 | 4.28E-09 | 4.67E-07 |
| SERPINA1 | 3.212583632 | 0.547124836 | 4.31E-09 | 4.67E-07 |
| HAMP | 4.659449749 | 0.795922477 | 4.79E-09 | 5.14E-07 |
| ANKRD22 | 4.578985811 | 0.783562169 | 5.10E-09 | 5.41E-07 |
| MMP8 | 5.103860577 | 0.874279497 | 5.29E-09 | 5.55E-07 |
| IL1RN | 4.941216915 | 0.846798122 | 5.37E-09 | 5.59E-07 |
| SMTN | -5.300781147 | 0.91037325 | 5.79E-09 | 5.96E-07 |
| HERC5 | 4.424176341 | 0.761658744 | 6.30E-09 | 6.42E-07 |
| LOC100506229 | 5.351241967 | 0.924507781 | 7.11E-09 | 7.18E-07 |
| RTP4 | 3.687195452 | 0.637493883 | 7.30E-09 | 7.30E-07 |
| SERPINB9 | 4.065429558 | 0.70487133 | 8.04E-09 | 7.96E-07 |
| GCH1 | 3.929385698 | 0.689842993 | 1.23E-08 | 1.20E-06 |
| LOC147646 | 5.530827348 | 0.979132047 | 1.62E-08 | 1.57E-06 |
| HIST2H2BE | 3.72245385 | 0.660895732 | 1.78E-08 | 1.71E-06 |
| KCNJ2 | 4.424204722 | 0.786759156 | 1.87E-08 | 1.79E-06 |
| APOBEC3A | 5.122773404 | 0.913754721 | 2.07E-08 | 1.96E-06 |
| CHI3L2 | 4.354277384 | 0.781298079 | 2.50E-08 | 2.35E-06 |
| RRAD | -5.278725738 | 0.947578805 | 2.54E-08 | 2.36E-06 |
| CASP4 | 3.047757962 | 0.551629851 | 3.29E-08 | 3.03E-06 |
| TAOK3 | 2.986766849 | 0.542721283 | 3.73E-08 | 3.40E-06 |
| TNFSF14 | 3.877113759 | 0.705370575 | 3.87E-08 | 3.51E-06 |
| CSF3R | 4.012569246 | 0.730925809 | 4.03E-08 | 3.61E-06 |
| PLXNA2 | -5.04565836 | 0.920471523 | 4.21E-08 | 3.75E-06 |
| SLC26A8 | 4.718861275 | 0.861805409 | 4.36E-08 | 3.85E-06 |
| KRT81 | 5.285215098 | 0.966245189 | 4.50E-08 | 3.94E-06 |
| UBE2L6 | 2.9468131 | 0.539686587 | 4.76E-08 | 4.13E-06 |
| LILRB2 | 3.398897269 | 0.62356156 | 5.01E-08 | 4.32E-06 |
| KCNH7 | 4.754814122 | 0.875420316 | 5.59E-08 | 4.77E-06 |
| METTL7B | 4.862246874 | 0.896333189 | 5.81E-08 | 4.92E-06 |
| TRIM21 | 2.889534633 | 0.536879227 | 7.36E-08 | 6.19E-06 |
| CD53 | 3.914309239 | 0.728612433 | 7.77E-08 | 6.48E-06 |
| EVI2B | 3.619535537 | 0.674563304 | 8.06E-08 | 6.67E-06 |
| S100A12 | 4.994113819 | 0.935982444 | 9.52E-08 | 7.81E-06 |
| NDST1 | -4.658292247 | 0.876273499 | 1.06E-07 | 8.63E-06 |
| RASSF1 | -4.711777525 | 0.886615868 | 1.07E-07 | 8.65E-06 |
| PILRA | 3.303880232 | 0.62250353 | 1.11E-07 | 8.91E-06 |
| PHLDB1 | -4.972168548 | 0.939190713 | 1.20E-07 | 9.51E-06 |
| CACNA1A | 4.767567272 | 0.902752881 | 1.28E-07 | 1.01E-05 |
| DNAAF1 | 4.126146495 | 0.786359737 | 1.54E-07 | 1.21E-05 |
| S100A13 | -4.90393404 | 0.936744327 | 1.65E-07 | 1.28E-05 |
| SH3BGRL3 | 2.607499793 | 0.500571462 | 1.90E-07 | 1.47E-05 |
| EPSTI1 | 3.818368551 | 0.735723765 | 2.10E-07 | 1.61E-05 |
| LAMP3 | 3.633527711 | 0.700603549 | 2.15E-07 | 1.63E-05 |
| GBP1P1 | 3.327724104 | 0.642145393 | 2.19E-07 | 1.66E-05 |
| JUN | -3.572780312 | 0.691893864 | 2.42E-07 | 1.82E-05 |
| GNG5 | 3.277021086 | 0.637647121 | 2.76E-07 | 2.05E-05 |
| SAMSN1 | 3.508232616 | 0.68329491 | 2.83E-07 | 2.09E-05 |
| TCF25 | -4.817480815 | 0.938397231 | 2.84E-07 | 2.09E-05 |
| MPV17 | 3.048325347 | 0.595899069 | 3.13E-07 | 2.28E-05 |
| TNFSF13B | 4.534454708 | 0.88677312 | 3.16E-07 | 2.29E-05 |
| CCL7 | 4.595510311 | 0.899956393 | 3.28E-07 | 2.36E-05 |
| HIST1H2BC | 3.833938635 | 0.75179705 | 3.40E-07 | 2.43E-05 |
| SP110 | 3.436997082 | 0.674547564 | 3.48E-07 | 2.47E-05 |
| APBB1IP | 3.793105893 | 0.744964753 | 3.55E-07 | 2.50E-05 |
| FAM26F | 3.645500581 | 0.717203889 | 3.72E-07 | 2.60E-05 |
| RPL3 | -3.019516081 | 0.595342159 | 3.94E-07 | 2.74E-05 |
| SIRPB1 | 4.078625983 | 0.806243971 | 4.22E-07 | 2.91E-05 |
| PSTPIP2 | 3.704044151 | 0.73501688 | 4.67E-07 | 3.19E-05 |
| RPSA | -2.994000772 | 0.59423086 | 4.69E-07 | 3.19E-05 |
| DDX58 | 3.418396645 | 0.678535851 | 4.71E-07 | 3.19E-05 |
| GAK | -4.711245921 | 0.936003797 | 4.82E-07 | 3.22E-05 |
| CRISP3 | 4.951717087 | 0.983794258 | 4.82E-07 | 3.22E-05 |
| PATL1 | 3.462667483 | 0.688538699 | 4.93E-07 | 3.28E-05 |
| CYSTM1 | 2.855603326 | 0.5685961 | 5.11E-07 | 3.37E-05 |
| ARRB2 | 3.620481966 | 0.723080719 | 5.53E-07 | 3.63E-05 |
| PRELID1 | -4.666708365 | 0.934449255 | 5.91E-07 | 3.86E-05 |
| C1QC | 3.244610162 | 0.650148368 | 6.02E-07 | 3.90E-05 |
| EGR1 | -3.161701619 | 0.634390555 | 6.23E-07 | 4.00E-05 |
| F5 | 4.056293534 | 0.813981466 | 6.25E-07 | 4.00E-05 |
| DOCK6 | -4.723658348 | 0.948333309 | 6.33E-07 | 4.03E-05 |
| RBP4 | 3.728624247 | 0.749115176 | 6.45E-07 | 4.08E-05 |
| LCP2 | 3.704100608 | 0.745197759 | 6.67E-07 | 4.20E-05 |
| HCK | 2.565894602 | 0.516519951 | 6.78E-07 | 4.23E-05 |
| ACTB | 2.746756358 | 0.55303499 | 6.81E-07 | 4.23E-05 |
| LILRB3 | 3.16971823 | 0.642544245 | 8.09E-07 | 5.00E-05 |
| IL27 | 4.710266079 | 0.955716629 | 8.29E-07 | 5.09E-05 |
| CDC37 | -3.372451853 | 0.685635697 | 8.71E-07 | 5.32E-05 |
| IER2 | -3.200922953 | 0.651054587 | 8.81E-07 | 5.35E-05 |
| RGL4 | 3.320765478 | 0.676406754 | 9.13E-07 | 5.51E-05 |
| SPI1 | 3.622675424 | 0.738772676 | 9.41E-07 | 5.65E-05 |
| CSTA | 3.980491501 | 0.813250546 | 9.85E-07 | 5.88E-05 |
| C3AR1 | 3.615484309 | 0.739427365 | 1.01E-06 | 6.00E-05 |
| GPR65 | 3.052591714 | 0.625362605 | 1.05E-06 | 6.22E-05 |
| BCL3 | 2.850412795 | 0.584735611 | 1.09E-06 | 6.39E-05 |
| SLC25A44 | -4.57445071 | 0.938996863 | 1.11E-06 | 6.46E-05 |
| SERF2 | 2.535152971 | 0.521000037 | 1.14E-06 | 6.61E-05 |
| CCR1 | 3.388594075 | 0.697764223 | 1.20E-06 | 6.90E-05 |
| RNF19B | 3.363618324 | 0.693910621 | 1.25E-06 | 7.16E-05 |
| RNU11 | 4.298313856 | 0.886829859 | 1.25E-06 | 7.16E-05 |
| RAB8A | 2.834088781 | 0.58880354 | 1.48E-06 | 8.43E-05 |
| IFIT5 | 3.347458603 | 0.695761668 | 1.50E-06 | 8.47E-05 |
| MARCKS | 3.74988128 | 0.78280802 | 1.67E-06 | 9.35E-05 |
| ATG4B | -4.482447889 | 0.943326115 | 2.02E-06 | 0.000112643 |
| ARRDC2 | -4.598897899 | 0.968913755 | 2.07E-06 | 0.000115023 |
| PLEKHM2 | -4.21443541 | 0.889361099 | 2.15E-06 | 0.000118866 |
| MIER1 | 2.812481778 | 0.593798802 | 2.18E-06 | 0.000119448 |
| LOC257358 | 4.260154883 | 0.899600137 | 2.18E-06 | 0.000119448 |
| TNF | 4.005237859 | 0.84738979 | 2.28E-06 | 0.000124247 |
| SNORA12 | 4.511540597 | 0.955577955 | 2.34E-06 | 0.000126866 |
| BLOC1S1 | 3.351501085 | 0.71064447 | 2.40E-06 | 0.000129435 |
| FPR1 | 3.933572944 | 0.834701025 | 2.45E-06 | 0.000130588 |
| CYBB | 2.891233686 | 0.613690098 | 2.46E-06 | 0.000130588 |
| GTSF1 | 4.48305539 | 0.95155506 | 2.46E-06 | 0.000130588 |
| LILRA5 | 3.761468367 | 0.801930584 | 2.73E-06 | 0.000143804 |
| KCTD20 | -4.436405692 | 0.946820493 | 2.79E-06 | 0.000146575 |
| FGR | 3.395223121 | 0.726368486 | 2.95E-06 | 0.00015414 |
| IRF9 | 2.639856207 | 0.566704488 | 3.19E-06 | 0.000165778 |
| ARHGEF15 | -4.489184031 | 0.964492188 | 3.25E-06 | 0.000168037 |
| TLE4 | 2.592273313 | 0.557316985 | 3.30E-06 | 0.000169573 |
| PIK3IP1 | -4.399647591 | 0.946048477 | 3.31E-06 | 0.000169573 |
| SEMA3F | -4.436789111 | 0.954239744 | 3.33E-06 | 0.000169573 |
| CD82 | -4.438207248 | 0.955654266 | 3.41E-06 | 0.000173226 |
| ACTR1B | -4.408945107 | 0.950089507 | 3.47E-06 | 0.000175422 |
| NFKBID | 2.9319399 | 0.632211192 | 3.52E-06 | 0.000177103 |
| CYP4F3 | 4.471342409 | 0.972122844 | 4.23E-06 | 0.000211707 |
| MCM7 | -4.088694072 | 0.891777471 | 4.54E-06 | 0.000226067 |
| GUCD1 | -4.357843563 | 0.95404607 | 4.93E-06 | 0.00024417 |
| FBXW5 | -4.352174202 | 0.953006909 | 4.95E-06 | 0.00024417 |
| BAP1 | -4.361884262 | 0.955961323 | 5.05E-06 | 0.000247202 |
| EDC4 | -4.331188782 | 0.949410966 | 5.07E-06 | 0.000247202 |
| HLA-J | 4.115138744 | 0.902193826 | 5.08E-06 | 0.000247202 |
| BIN2 | 2.858506502 | 0.628077324 | 5.33E-06 | 0.000258123 |
| NES | -3.977177014 | 0.874651222 | 5.44E-06 | 0.000261924 |
| PPP1R13B | -4.316112889 | 0.950045737 | 5.54E-06 | 0.000265853 |
| TRAK1 | -4.312448959 | 0.949817513 | 5.62E-06 | 0.00026814 |
| TXNDC11 | -4.315286212 | 0.952246381 | 5.85E-06 | 0.000278032 |
| TCERG1 | -4.312299964 | 0.955121471 | 6.33E-06 | 0.000299608 |
| SPHK1 | -4.142845396 | 0.918475646 | 6.47E-06 | 0.000304452 |
| CSF2RB | 2.940907806 | 0.652140766 | 6.49E-06 | 0.000304452 |
| ARSE | 4.563778775 | 1.013350668 | 6.68E-06 | 0.000311742 |
| ECRP | 4.732769835 | 1.051183095 | 6.72E-06 | 0.000312305 |
| PODXL | -3.853542021 | 0.856533728 | 6.83E-06 | 0.00031584 |
| PYCR2 | -4.277115092 | 0.951741222 | 6.99E-06 | 0.000321943 |
| MOAP1 | -4.26067264 | 0.952368646 | 7.69E-06 | 0.000352423 |
| GBP5 | 4.021608705 | 0.899412019 | 7.77E-06 | 0.000354834 |
| RGS18 | 3.914287911 | 0.875938076 | 7.87E-06 | 0.000357777 |
| TIMM23 | 2.592356177 | 0.58081731 | 8.07E-06 | 0.00036532 |
| PER1 | -4.338156344 | 0.973110089 | 8.27E-06 | 0.000372787 |
| WDR74 | -3.987089975 | 0.894642004 | 8.33E-06 | 0.000373627 |
| ITPKB | -3.855901733 | 0.866762783 | 8.64E-06 | 0.000386178 |
| XAB2 | -4.24025744 | 0.954409389 | 8.88E-06 | 0.000394492 |
| ANXA2R | 3.121186311 | 0.702618035 | 8.90E-06 | 0.000394492 |
| DMBT1 | 2.881673288 | 0.649315695 | 9.08E-06 | 0.000397754 |
| UCP2 | 3.285411024 | 0.74029769 | 9.08E-06 | 0.000397754 |
| S100A8 | 3.975430204 | 0.895822238 | 9.09E-06 | 0.000397754 |
| SLC25A4 | -4.239183474 | 0.956580611 | 9.35E-06 | 0.000406096 |
| FGL2 | 3.301826015 | 0.745083512 | 9.36E-06 | 0.000406096 |
| GNG2 | 3.281354718 | 0.743413177 | 1.02E-05 | 0.00043872 |
| RASA3 | -3.815597599 | 0.864953394 | 1.03E-05 | 0.000440339 |
| XAF1 | 2.695889806 | 0.611063715 | 1.03E-05 | 0.000440339 |
| HLA-DRB5 | 2.645936974 | 0.600353261 | 1.05E-05 | 0.000446793 |
| FAM32A | -3.685198184 | 0.838443337 | 1.11E-05 | 0.000470322 |
| ARAP3 | -4.257658885 | 0.969043765 | 1.11E-05 | 0.000471922 |
| OASL | 3.331232101 | 0.759599348 | 1.16E-05 | 0.000487994 |
| STRN4 | 2.833011004 | 0.646205109 | 1.16E-05 | 0.000488131 |
| HS3ST3A1 | 4.026721568 | 0.918564884 | 1.17E-05 | 0.000488131 |
| CR1 | 3.262437555 | 0.745024246 | 1.19E-05 | 0.000496891 |
| USB1 | 2.442956657 | 0.558020269 | 1.20E-05 | 0.00049736 |
| TCN1 | 4.358951226 | 0.996006138 | 1.21E-05 | 0.000498751 |
| CERS6 | 2.892988865 | 0.66195681 | 1.24E-05 | 0.000510812 |
| TARP | 4.1835294 | 0.957575142 | 1.25E-05 | 0.000512278 |
| SREK1 | -3.910605877 | 0.896522598 | 1.29E-05 | 0.000526667 |
| PI4K2B | 2.803026566 | 0.643063538 | 1.31E-05 | 0.000532129 |
| LMBR1L | -4.18637071 | 0.961372562 | 1.33E-05 | 0.000540555 |
| CPSF3L | -4.181996246 | 0.960819917 | 1.35E-05 | 0.000543533 |
| TUBA4A | -4.062545461 | 0.933814774 | 1.36E-05 | 0.000546526 |
| ATP7B | 3.862036277 | 0.888430316 | 1.38E-05 | 0.000553071 |
| CARD17 | 4.444555822 | 1.023309079 | 1.40E-05 | 0.000560375 |
| MAFG | -4.19929747 | 0.969140994 | 1.47E-05 | 0.000582843 |
| CXCL10 | 4.038769832 | 0.931933798 | 1.47E-05 | 0.000582843 |
| CEACAM1 | 3.850550353 | 0.890557579 | 1.53E-05 | 0.000605574 |
| PIEZO1 | -3.16762316 | 0.733151355 | 1.56E-05 | 0.000612106 |
| ATP5J2 | 2.60431316 | 0.60342376 | 1.59E-05 | 0.000622843 |
| NOP10 | 2.393551146 | 0.555094443 | 1.62E-05 | 0.000631644 |
| PPP1R14B | -4.187257074 | 0.97171386 | 1.64E-05 | 0.000637395 |
| RXRB | -4.144776865 | 0.963964807 | 1.71E-05 | 0.000662669 |
| DCHS1 | -4.141237097 | 0.963488846 | 1.72E-05 | 0.000664866 |
| TBCD | -4.183467687 | 0.973914607 | 1.74E-05 | 0.000670394 |
| MICAL3 | -4.116162495 | 0.958704346 | 1.76E-05 | 0.00067416 |
| TFIP11 | -4.112985072 | 0.960085459 | 1.84E-05 | 0.000701012 |
| RALGDS | -3.800173358 | 0.887536213 | 1.85E-05 | 0.000705629 |
| RND1 | -3.282653149 | 0.767866933 | 1.91E-05 | 0.000724503 |
| CRY2 | -4.100353396 | 0.95947386 | 1.92E-05 | 0.000726724 |
| PFN1 | 2.216908686 | 0.519532505 | 1.98E-05 | 0.000745273 |
| CA1 | 4.315623541 | 1.012540565 | 2.02E-05 | 0.000759253 |
| CDHR5 | 4.026598451 | 0.945144807 | 2.04E-05 | 0.000762928 |
| ZC3H7A | -3.847193031 | 0.903385158 | 2.06E-05 | 0.000763892 |
| PSENEN | 2.756546396 | 0.647315173 | 2.06E-05 | 0.000763892 |
| TPP1 | 2.726413821 | 0.642646819 | 2.21E-05 | 0.000817403 |
| CHI3L1 | 3.813524678 | 0.899213046 | 2.23E-05 | 0.000820065 |
| ARHGEF7 | -3.458722838 | 0.816925541 | 2.30E-05 | 0.000843553 |
| JUND | 2.907497579 | 0.687416401 | 2.34E-05 | 0.000856565 |
| PLP2 | 2.161873685 | 0.51182889 | 2.40E-05 | 0.000875793 |
| PRKAB1 | -4.05641898 | 0.961630391 | 2.46E-05 | 0.000894515 |
| CYP1A1 | -3.818750708 | 0.905547775 | 2.48E-05 | 0.000896229 |
| MADD | -4.056391215 | 0.963085857 | 2.53E-05 | 0.000913936 |
| NOTCH2NL | -4.049458119 | 0.964128063 | 2.67E-05 | 0.00095936 |
| CRYAB | 2.413429436 | 0.576042927 | 2.79E-05 | 0.001001219 |
| PEX5 | -3.506203673 | 0.838511067 | 2.90E-05 | 0.001034521 |
| VTRNA1-1 | 3.877610796 | 0.927766087 | 2.92E-05 | 0.001039896 |
| PRMT1 | -2.96095963 | 0.709014431 | 2.96E-05 | 0.001051272 |
| BLCAP | -3.785968512 | 0.906710101 | 2.97E-05 | 0.001051272 |
| CD38 | 3.017829914 | 0.723267751 | 3.01E-05 | 0.001061673 |
| MAP2K6 | 3.952975639 | 0.947870685 | 3.04E-05 | 0.00106801 |
| RSAD1 | -4.016867247 | 0.963419132 | 3.05E-05 | 0.001069072 |
| ITPR1 | -4.021776789 | 0.964918861 | 3.07E-05 | 0.001072047 |
| C2CD2 | -4.02216174 | 0.967274904 | 3.21E-05 | 0.001112155 |
| PCGF5 | 3.031180479 | 0.728982103 | 3.21E-05 | 0.001112155 |
| C6 | 3.879146929 | 0.933252983 | 3.23E-05 | 0.001115861 |
| PPP6R2 | -4.006210326 | 0.964098714 | 3.25E-05 | 0.001118019 |
| C7orf41 | -4.048549251 | 0.977141221 | 3.42E-05 | 0.00117503 |
| C3orf37 | -4.002390947 | 0.966250306 | 3.44E-05 | 0.001176684 |
| PSME3 | 2.254400497 | 0.544487529 | 3.47E-05 | 0.001182006 |
| EIF1B | 2.24591688 | 0.542727226 | 3.50E-05 | 0.001189544 |
| AKAP17A | -3.990520413 | 0.964494685 | 3.51E-05 | 0.001189769 |
| RPS28 | -2.539650599 | 0.614048276 | 3.54E-05 | 0.001193755 |
| ZDHHC19 | 3.720724415 | 0.899912971 | 3.56E-05 | 0.001197078 |
| XPC | -3.984026187 | 0.964881999 | 3.64E-05 | 0.001222201 |
| MAF1 | -3.631330134 | 0.879877182 | 3.67E-05 | 0.001228639 |
| H2AFJ | -3.610434532 | 0.875537039 | 3.73E-05 | 0.001243008 |
| STARD10 | -4.001841138 | 0.972854325 | 3.90E-05 | 0.001295001 |
| WIPF1 | 2.178128013 | 0.530101303 | 3.98E-05 | 0.001314865 |
| STAT4 | 2.802003448 | 0.681995904 | 3.98E-05 | 0.001314865 |
| GBP4 | 3.0001335 | 0.730708583 | 4.03E-05 | 0.001326422 |
| SPRED1 | -3.963914817 | 0.965947843 | 4.07E-05 | 0.001334537 |
| LTF | 3.058507254 | 0.745757833 | 4.11E-05 | 0.001344523 |
| PCID2 | -3.976431242 | 0.971394578 | 4.25E-05 | 0.001385539 |
| ASB1 | -3.954122914 | 0.966642663 | 4.30E-05 | 0.001398966 |
| STC1 | -3.705258557 | 0.907035409 | 4.41E-05 | 0.001428423 |
| MYO1D | -3.960412386 | 0.969834534 | 4.43E-05 | 0.001432792 |
| ZFAND2B | -3.962902461 | 0.97138815 | 4.51E-05 | 0.001452997 |
| ARFRP1 | -3.336884805 | 0.818133242 | 4.53E-05 | 0.001454617 |
| C16orf80 | -3.943918655 | 0.968108636 | 4.62E-05 | 0.001472788 |
| IRG1 | 4.193808796 | 1.029338181 | 4.62E-05 | 0.001472788 |
| RNU4ATAC | 3.68740162 | 0.905185032 | 4.63E-05 | 0.001472788 |
| ENTPD6 | -3.942886035 | 0.968860468 | 4.71E-05 | 0.001493982 |
| ADCY6 | -3.942199341 | 0.968873894 | 4.72E-05 | 0.001494381 |
| ADAM15 | -3.931060194 | 0.967064625 | 4.80E-05 | 0.001515068 |
| SIGLEC7 | 3.289573105 | 0.81027847 | 4.91E-05 | 0.001544137 |
| F8A1 | 3.166685699 | 0.780819476 | 5.00E-05 | 0.001567544 |
| TLR1 | 3.174177863 | 0.783058664 | 5.04E-05 | 0.001575734 |
| ZSWIM8 | -3.939836282 | 0.972083174 | 5.06E-05 | 0.001575734 |
| EIF4E2 | -3.9197216 | 0.967819132 | 5.12E-05 | 0.001584247 |
| FCGR2A | 2.776696432 | 0.685564938 | 5.12E-05 | 0.001584247 |
| ADAMTS2 | -3.920479228 | 0.968267843 | 5.14E-05 | 0.001584247 |
| SLC25A3 | -2.408660856 | 0.594861674 | 5.14E-05 | 0.001584247 |
| CCDC104 | -3.663769707 | 0.905314991 | 5.19E-05 | 0.001593206 |
| S100A9 | 3.39147117 | 0.838242492 | 5.21E-05 | 0.001595528 |
| RRP7A | -3.005447567 | 0.743197692 | 5.26E-05 | 0.00160446 |
| CCDC140 | 4.254014777 | 1.052534631 | 5.31E-05 | 0.001615273 |
| GATA2 | -3.946767909 | 0.97703297 | 5.36E-05 | 0.001625384 |
| SGSM2 | -3.935095906 | 0.975126654 | 5.45E-05 | 0.001649073 |
| FZD4 | -3.961818888 | 0.983917178 | 5.66E-05 | 0.001707757 |
| CC2D1B | -3.905313609 | 0.970177313 | 5.69E-05 | 0.00171092 |
| FAM160B2 | -3.922753429 | 0.974647018 | 5.70E-05 | 0.00171092 |
| XRCC6 | -2.655636701 | 0.660051325 | 5.74E-05 | 0.001716331 |
| MMP25 | 2.915549809 | 0.725056301 | 5.79E-05 | 0.001725248 |
| STAT2 | 2.1093889 | 0.524616478 | 5.80E-05 | 0.001725248 |
| THOC6 | -4.001017767 | 0.995415908 | 5.83E-05 | 0.001730465 |
| IDO1 | 3.529088149 | 0.878717171 | 5.91E-05 | 0.001749631 |
| SKIV2L | -3.893211832 | 0.971646345 | 6.15E-05 | 0.001811082 |
| ZNF451 | -3.906739512 | 0.975096816 | 6.16E-05 | 0.001811082 |
| VPS13D | -3.48500836 | 0.869936985 | 6.17E-05 | 0.001811082 |
| TMBIM1 | -2.741390762 | 0.684710947 | 6.24E-05 | 0.001823904 |
| SH3PXD2B | -3.88801401 | 0.972550486 | 6.39E-05 | 0.001865309 |
| CDA | 3.780778372 | 0.948709098 | 6.74E-05 | 0.001961095 |
| GAS6 | -4.005879788 | 1.005416776 | 6.77E-05 | 0.001961095 |
| SIVA1 | -3.511429328 | 0.881403422 | 6.78E-05 | 0.001961095 |
| B4GALT3 | -3.277565371 | 0.82435189 | 7.01E-05 | 0.002022445 |
| KLHDC3 | -3.705151958 | 0.93272966 | 7.12E-05 | 0.002041658 |
| TMEM154 | 3.374421146 | 0.849469988 | 7.12E-05 | 0.002041658 |
| DBNDD1 | 3.122679349 | 0.786699509 | 7.21E-05 | 0.002062185 |
| GLT8D1 | -3.856873322 | 0.972356006 | 7.29E-05 | 0.002081038 |
| CFL1 | 2.298058377 | 0.579659362 | 7.36E-05 | 0.002093102 |
| LILRA1 | 3.218707402 | 0.812324632 | 7.42E-05 | 0.002106397 |
| SIN3B | -3.670066624 | 0.92741966 | 7.58E-05 | 0.002145644 |
| NDRG1 | -2.724941987 | 0.68871798 | 7.60E-05 | 0.002146561 |
| PQLC1 | -3.88316453 | 0.981768422 | 7.64E-05 | 0.002152185 |
| NEBL | -3.852628765 | 0.974471392 | 7.70E-05 | 0.002161907 |
| MKL1 | -3.842054085 | 0.971976336 | 7.72E-05 | 0.00216274 |
| LINC00657 | -2.724324019 | 0.689388415 | 7.76E-05 | 0.00216628 |
| ARHGAP10 | -3.86571228 | 0.978824935 | 7.84E-05 | 0.002182844 |
| CHFR | -3.833762249 | 0.971973709 | 8.00E-05 | 0.002223302 |
| PRKY | -3.834119866 | 0.972227779 | 8.03E-05 | 0.002223577 |
| MCTP2 | 3.01066933 | 0.764341876 | 8.19E-05 | 0.002261989 |
| ATRX | -3.830338953 | 0.972712661 | 8.22E-05 | 0.002266542 |
| NAP1L4 | -2.91129098 | 0.740937829 | 8.52E-05 | 0.002342264 |
| MYO9B | -3.565032478 | 0.907447708 | 8.54E-05 | 0.002342264 |
| CHIT1 | 2.931938271 | 0.746832563 | 8.64E-05 | 0.00236357 |
| NMI | 2.509948021 | 0.639852944 | 8.76E-05 | 0.002388335 |
| CRIP1 | -3.900225384 | 0.995350531 | 8.91E-05 | 0.002424545 |
| METTL3 | -3.810794077 | 0.973442048 | 9.05E-05 | 0.00245548 |
| MNDA | 3.554803554 | 0.908594531 | 9.14E-05 | 0.002473033 |
| SP140L | 2.732543468 | 0.699057906 | 9.27E-05 | 0.00250295 |
| RPS5 | -2.120886251 | 0.543035688 | 9.40E-05 | 0.002530675 |
| CTCF | -3.801054744 | 0.973573022 | 9.45E-05 | 0.002538641 |
| PPP1R16B | -3.813845167 | 0.977111751 | 9.49E-05 | 0.002543176 |
| SDCCAG3 | -3.79764913 | 0.973778009 | 9.62E-05 | 0.002571206 |
| EXOSC7 | -3.795753877 | 0.974223858 | 9.77E-05 | 0.002604482 |
| RNF31 | -3.799016295 | 0.976828456 | 0.000100603 | 0.002665151 |
| C1orf194 | 3.238444647 | 0.832711661 | 0.000100644 | 0.002665151 |
| BCOR | -3.823884597 | 0.983442504 | 0.000100964 | 0.002665151 |
| LCN10 | -3.844511075 | 0.989008507 | 0.000101391 | 0.002665151 |
| RPLP0 | -2.456030619 | 0.631868691 | 0.000101519 | 0.002665151 |
| HBA1 | 3.644449759 | 0.937620076 | 0.000101526 | 0.002665151 |
| CXCL17 | 3.19186491 | 0.821306966 | 0.000101774 | 0.002665151 |
| FHL3 | 2.337502232 | 0.601747029 | 0.00010253 | 0.002678266 |
| MFAP1 | -3.810792348 | 0.981510744 | 0.000103355 | 0.002686991 |
| LETM1 | -3.786599893 | 0.97529189 | 0.000103375 | 0.002686991 |
| PPP3R1 | -3.293143406 | 0.848735081 | 0.000104431 | 0.002689599 |
| LOC100505817 | 5.11151658 | 1.317387006 | 0.000104439 | 0.002689599 |
| PLEKHM1 | -3.811905803 | 0.982651074 | 0.000104798 | 0.002689599 |
| KDM1A | -3.28280315 | 0.84627451 | 0.000104834 | 0.002689599 |
| DTX4 | -3.810043459 | 0.982197838 | 0.000104844 | 0.002689599 |
| CTF1 | 3.439739938 | 0.886826061 | 0.000105012 | 0.002689599 |
| P2RY14 | 3.240402176 | 0.836070685 | 0.000106296 | 0.002715853 |
| C5AR1 | 2.47312557 | 0.639782511 | 0.000110829 | 0.002824794 |
| CLEC12B | 3.659618154 | 0.947570443 | 0.000112413 | 0.002858241 |
| PPIG | -3.530561023 | 0.915298346 | 0.000114656 | 0.002908227 |
| LPAR6 | 2.383864754 | 0.618546178 | 0.000116213 | 0.002940608 |
| VNN3 | 2.930874608 | 0.76125145 | 0.000118082 | 0.002974192 |
| MGAM | 3.429951536 | 0.890891142 | 0.000118107 | 0.002974192 |
| SMPD4 | -3.757634949 | 0.976167933 | 0.00011842 | 0.002974957 |
| TET2 | 2.273373326 | 0.590962914 | 0.000119623 | 0.0029915 |
| NME1 | 2.316023454 | 0.60205791 | 0.000119649 | 0.0029915 |
| MFNG | -3.809451813 | 0.99078422 | 0.000120609 | 0.003008344 |
| MGLL | -2.630299836 | 0.684809426 | 0.000122573 | 0.00305009 |
| MPHOSPH10 | -3.746907426 | 0.976909487 | 0.000125324 | 0.003111181 |
| FAM118A | -3.778470938 | 0.985384312 | 0.000125812 | 0.003115931 |
| GCA | 2.952565489 | 0.770304964 | 0.000126599 | 0.003124339 |
| SNX11 | -3.744432904 | 0.976971146 | 0.000126747 | 0.003124339 |
| DLL1 | -3.741695277 | 0.976490869 | 0.000127221 | 0.003127882 |
| MLXIP | -3.74361379 | 0.977264579 | 0.000127776 | 0.003127882 |
| CHMP1A | -3.240895462 | 0.846034271 | 0.000127784 | 0.003127882 |
| DDX60 | 2.343544323 | 0.612261741 | 0.000129355 | 0.003158965 |
| ULK3 | -3.749009531 | 0.979792265 | 0.000130067 | 0.003168996 |
| CD74 | 2.339169362 | 0.612359248 | 0.00013349 | 0.003244015 |
| SPTLC2 | 2.353694542 | 0.616297987 | 0.000133947 | 0.003244015 |
| ITPA | -3.746356007 | 0.981153784 | 0.000134365 | 0.003244015 |
| CTR9 | -3.753256808 | 0.982969028 | 0.000134382 | 0.003244015 |
| CCDC130 | -3.732931392 | 0.978060715 | 0.000135267 | 0.003257885 |
| WDR37 | -3.728622478 | 0.977165965 | 0.000135769 | 0.003258058 |
| NCAPH2 | -3.755778064 | 0.984341539 | 0.000135895 | 0.003258058 |
| TSR1 | -3.74325443 | 0.981224551 | 0.000136249 | 0.003259109 |
| TAF1 | -3.725021343 | 0.977168139 | 0.000137815 | 0.003289073 |
| SF3A2 | 2.634672018 | 0.691296564 | 0.000138292 | 0.003292974 |
| NASP | -2.660343269 | 0.698411807 | 0.000139455 | 0.00330496 |
| GET4 | -3.481959091 | 0.914119775 | 0.000139483 | 0.00330496 |
| CBX4 | 2.472963242 | 0.649385805 | 0.000140006 | 0.00330496 |
| FIBP | -3.376552196 | 0.886682661 | 0.000140054 | 0.00330496 |
| TMEM255B | -3.730536528 | 0.979926912 | 0.000140689 | 0.0033125 |
| DDX5 | -1.995463526 | 0.524430258 | 0.000141797 | 0.003331124 |
| SERTAD1 | -2.637167169 | 0.693275337 | 0.000142421 | 0.003338322 |
| INPP5A | -3.795558745 | 0.998994285 | 0.000145059 | 0.003392563 |
| PHC2 | -2.757773136 | 0.725958055 | 0.000145395 | 0.003392882 |
| RAPGEF3 | -3.793521059 | 0.99954497 | 0.000147496 | 0.003427467 |
| SLC9A1 | -3.733136483 | 0.983649358 | 0.00014753 | 0.003427467 |
| CCL18 | 3.021183782 | 0.796210715 | 0.00014797 | 0.003430085 |
| HARS2 | -3.714950833 | 0.979307771 | 0.000148571 | 0.003436444 |
| EIF2B5 | -3.153095988 | 0.831881839 | 0.000150455 | 0.003472371 |
| TNS1 | -2.949850317 | 0.77849912 | 0.000151163 | 0.003481066 |
| C19orf43 | 2.297990141 | 0.606858862 | 0.000152664 | 0.003507922 |
| RNMT | -3.762439896 | 0.994402282 | 0.000154564 | 0.003543825 |
| TNFSF8 | 2.583449831 | 0.682896313 | 0.000154901 | 0.003543825 |
| HERC6 | 2.879456593 | 0.761606149 | 0.000156345 | 0.003569083 |
| BCR | -3.664360941 | 0.969756854 | 0.000157688 | 0.003591891 |
| RAPGEF1 | -2.699799502 | 0.714591474 | 0.000158028 | 0.003591891 |
| FAM8A1 | 2.730123015 | 0.723519636 | 0.000161043 | 0.003652514 |
| EDC3 | -3.698904974 | 0.980786816 | 0.000162357 | 0.003669402 |
| SLC25A29 | -3.746600115 | 0.993587784 | 0.000162739 | 0.003669402 |
| HBP1 | -3.558646437 | 0.94379149 | 0.000162865 | 0.003669402 |
| MDC1 | -3.69193461 | 0.979373216 | 0.00016345 | 0.003669402 |
| BAG3 | -2.496482251 | 0.662273991 | 0.000163535 | 0.003669402 |
| EIF2B4 | -3.291503892 | 0.873796339 | 0.000165287 | 0.003700804 |
| HRASLS2 | 3.300552955 | 0.876972469 | 0.0001675 | 0.003727875 |
| TMEM208 | -3.345659323 | 0.888973327 | 0.000167546 | 0.003727875 |
| OLIG1 | 3.549366703 | 0.94310605 | 0.000167561 | 0.003727875 |
| ZNF385A | 2.619593107 | 0.696288272 | 0.000168408 | 0.003733853 |
| SPRYD3 | -3.317108993 | 0.881734121 | 0.000168541 | 0.003733853 |
| C21orf7 | 2.457424303 | 0.653652253 | 0.000170234 | 0.003763423 |
| AGAP3 | -3.202677558 | 0.852254842 | 0.000171354 | 0.003774261 |
| PLBD1 | 2.688116384 | 0.715351227 | 0.000171443 | 0.003774261 |
| CCDC60 | 3.651462194 | 0.97192672 | 0.000172009 | 0.003778811 |
| CECR5 | -3.677325836 | 0.979853455 | 0.000174777 | 0.003824713 |
| MBIP | -3.683148918 | 0.981424118 | 0.000174827 | 0.003824713 |
| F2RL3 | -3.710115177 | 0.988778185 | 0.000175274 | 0.003826516 |
| ZNF160 | -3.677835948 | 0.981623549 | 0.000179186 | 0.003903793 |
| VPS54 | -3.670619128 | 0.979917212 | 0.000179787 | 0.00390878 |
| IFI30 | 2.153031142 | 0.575302733 | 0.000182248 | 0.003954107 |
| CTDSP2 | -2.767208288 | 0.739702131 | 0.000183307 | 0.003968873 |
| PCDH12 | -3.423380154 | 0.915479205 | 0.00018443 | 0.003983708 |
| GNLY | 2.602974893 | 0.696231565 | 0.000185002 | 0.003983708 |
| SLAMF7 | 3.021735428 | 0.808385432 | 0.000185498 | 0.003983708 |
| TGFBR2 | -2.524626204 | 0.675399656 | 0.000185509 | 0.003983708 |
| FCAR | 2.68539843 | 0.718711125 | 0.000186667 | 0.003985612 |
| RSAD2 | 3.391736479 | 0.907779039 | 0.000186747 | 0.003985612 |
| CLDN2 | 3.647546301 | 0.976307386 | 0.000186924 | 0.003985612 |
| AMFR | -3.155620304 | 0.844696364 | 0.000187116 | 0.003985612 |
| STAT1 | 2.834752931 | 0.759478015 | 0.000189587 | 0.004030077 |
| TTL | -3.419711239 | 0.916640131 | 0.000190947 | 0.004050775 |
| CYP7B1 | 3.273365732 | 0.877676137 | 0.000191797 | 0.004060609 |
| TBC1D9 | -3.41513508 | 0.916705586 | 0.000194972 | 0.004119518 |
| CLEC7A | 3.138152093 | 0.84329427 | 0.000198199 | 0.004179287 |
| KAT5 | -3.648873608 | 0.980760599 | 0.000198866 | 0.00418495 |
| CLDN5 | -3.555324812 | 0.956310772 | 0.000201005 | 0.0042215 |
| PXDN | -3.337747822 | 0.899118851 | 0.00020543 | 0.004305839 |
| KCTD10 | -2.889428585 | 0.780016004 | 0.000211959 | 0.004433825 |
| GMFG | 2.493554711 | 0.673857053 | 0.000215242 | 0.004493555 |
| LDB1 | -3.648686828 | 0.986661329 | 0.000217293 | 0.004527377 |
| USP15 | 2.673840385 | 0.723810849 | 0.000220654 | 0.004588294 |
| DNAJC7 | -3.277739437 | 0.887805877 | 0.000222537 | 0.004618297 |
| LZTR1 | -3.660434367 | 0.991706285 | 0.000223333 | 0.00462568 |
| JAK3 | 2.410858638 | 0.653571378 | 0.000225362 | 0.004658523 |
| IRF7 | 2.351789031 | 0.637721117 | 0.0002262 | 0.004666649 |
| TTC7A | -3.641195188 | 0.987782812 | 0.0002276 | 0.004684028 |
| ABLIM1 | -2.871784126 | 0.779231519 | 0.000228339 | 0.004684028 |
| LRFN1 | 3.415837665 | 0.926866972 | 0.00022838 | 0.004684028 |
| CMC2 | 2.46933721 | 0.670310223 | 0.000229717 | 0.004702266 |
| SGSM3 | -3.651526746 | 0.991534999 | 0.000230776 | 0.004714744 |
| SLC25A36 | -3.37629599 | 0.917251969 | 0.000232429 | 0.00473437 |
| SNAI1 | -3.104679052 | 0.843513334 | 0.000232638 | 0.00473437 |
| MEIS1 | -3.63087367 | 0.986717433 | 0.000233463 | 0.004741954 |
| RHOC | -2.070441541 | 0.563141817 | 0.000236373 | 0.004788854 |
| PHF11 | 2.598897648 | 0.707010155 | 0.000237014 | 0.004788854 |
| AHNAK | -2.883099965 | 0.784354182 | 0.00023714 | 0.004788854 |
| UBE2J2 | -3.23845915 | 0.88133427 | 0.000238319 | 0.004803441 |
| COL12A1 | -3.346527602 | 0.912646874 | 0.000245569 | 0.004940075 |
| BAALC | -3.604065811 | 0.983142214 | 0.000246505 | 0.004949419 |
| ANP32A | 2.120931473 | 0.578733941 | 0.000247553 | 0.004960983 |
| TRIM22 | 2.79237882 | 0.76253292 | 0.000250275 | 0.005001735 |
| ILVBL | -3.605495708 | 0.984648587 | 0.000250539 | 0.005001735 |
| LRRC47 | -3.610864427 | 0.986990876 | 0.000253738 | 0.005044321 |
| FKBP1A | 2.078190118 | 0.568084035 | 0.000253949 | 0.005044321 |
| LINC00605 | 3.55554444 | 0.972015575 | 0.000254281 | 0.005044321 |
| NFATC2 | -3.596367476 | 0.983288186 | 0.000254696 | 0.005044321 |
| ZSCAN18 | -3.632491948 | 0.993282391 | 0.000255126 | 0.005044321 |
| ASL | -3.401578154 | 0.93038139 | 0.00025607 | 0.005044321 |
| GALNT18 | -3.328558199 | 0.910479491 | 0.000256351 | 0.005044321 |
| MSMO1 | 2.050808737 | 0.560994587 | 0.000256515 | 0.005044321 |
| GMPPA | -3.601984264 | 0.98545758 | 0.000257043 | 0.005045247 |
| IFI44 | 2.642506762 | 0.723309757 | 0.000258837 | 0.005062048 |
| NR1D2 | -3.632637321 | 0.994492819 | 0.000259443 | 0.005062048 |
| CTPS1 | -3.434123135 | 0.940307727 | 0.000260077 | 0.005062048 |
| OSBPL5 | -3.598676238 | 0.985377895 | 0.000260128 | 0.005062048 |
| MAT2A | -2.728314769 | 0.747094971 | 0.000260309 | 0.005062048 |
| SF3B4 | 2.279911672 | 0.624401186 | 0.000260858 | 0.005063351 |
| ATP8B2 | -3.349979717 | 0.918066903 | 0.000263315 | 0.005101605 |
| CAMTA2 | -3.394499307 | 0.930604057 | 0.00026467 | 0.005118423 |
| ZBTB44 | -3.59045576 | 0.98461437 | 0.000265774 | 0.005130318 |
| IFT43 | -3.361915484 | 0.922184574 | 0.000266769 | 0.005140082 |
| PTCD3 | -3.377009709 | 0.926760375 | 0.000268552 | 0.00515977 |
| ZDHHC7 | -3.080559024 | 0.845454077 | 0.000268774 | 0.00515977 |
| ATP6V0A2 | -3.58952477 | 0.985712653 | 0.000270998 | 0.005192983 |
| KIF3B | -3.182540837 | 0.874987876 | 0.000275575 | 0.005271066 |
| WDR85 | -3.580622344 | 0.985709411 | 0.000280652 | 0.005358417 |
| VASP | 1.969953112 | 0.542528348 | 0.000282259 | 0.005371053 |
| GRPEL2 | -3.665468027 | 1.009495716 | 0.000282337 | 0.005371053 |
| NOL8 | -3.572192574 | 0.984462237 | 0.000284993 | 0.005395539 |
| C19orf48 | -3.599460484 | 0.991992818 | 0.000285057 | 0.005395539 |
| SEPHS1 | -3.590112278 | 0.989443428 | 0.000285166 | 0.005395539 |
| TMEM194B | 2.590147132 | 0.714908119 | 0.000291151 | 0.005498872 |
| FAM178A | -3.567242549 | 0.984758948 | 0.000291823 | 0.005499605 |
| PYGO2 | -3.569879557 | 0.985586625 | 0.000292237 | 0.005499605 |
| S1PR2 | -3.566882205 | 0.985370003 | 0.000294785 | 0.005537628 |
| ELK3 | -3.60859117 | 0.999029481 | 0.000303731 | 0.005695502 |
| THAP2 | -3.582430246 | 0.99192755 | 0.000304332 | 0.005696592 |
| CIRBP | -2.942913188 | 0.814965011 | 0.000304911 | 0.00569728 |
| MLH1 | -3.561091638 | 0.986700168 | 0.000307271 | 0.005731171 |
| GBP3 | 3.327258348 | 0.922369478 | 0.000309406 | 0.005760585 |
| DEXI | -3.555232652 | 0.985690938 | 0.000309945 | 0.005760585 |
| PIM2 | 2.347245135 | 0.651107493 | 0.000312148 | 0.005791285 |
| KIAA0930 | -3.346190151 | 0.928554862 | 0.000313775 | 0.005801171 |
| USP19 | -3.570815607 | 0.990890015 | 0.000313786 | 0.005801171 |
| PDE2A | -3.488004679 | 0.968620918 | 0.000316995 | 0.005850194 |
| ZMYM2 | -3.210998004 | 0.892012173 | 0.000318555 | 0.00586867 |
| WIPI1 | -3.203923826 | 0.89076497 | 0.000322128 | 0.005917494 |
| CXorf40B | -3.560984751 | 0.990093656 | 0.000322386 | 0.005917494 |
| NAB2 | -3.554457003 | 0.988459347 | 0.000323202 | 0.005917494 |
| VSIG2 | -3.544950566 | 0.985872484 | 0.000323459 | 0.005917494 |
| LINC00116 | -3.308016 | 0.920526217 | 0.000326123 | 0.005955852 |
| PGLYRP1 | 3.554804659 | 0.989853087 | 0.000329102 | 0.005996238 |
| BSDC1 | -2.960876563 | 0.824538757 | 0.000329476 | 0.005996238 |
| STARD9 | -3.557880129 | 0.990947578 | 0.000330194 | 0.005998905 |
| ACP2 | -3.54277059 | 0.987165512 | 0.000332164 | 0.006024265 |
| FAM13B | -3.536774502 | 0.98617503 | 0.000335331 | 0.006062719 |
| DHRS9 | 2.571244318 | 0.716968484 | 0.000335439 | 0.006062719 |
| FAM214B | -3.353477543 | 0.935535196 | 0.000337653 | 0.006092261 |
| DNAJB4 | -3.300554042 | 0.921305153 | 0.000340352 | 0.006121876 |
| KANK2 | -3.46357113 | 0.966859414 | 0.000340595 | 0.006121876 |
| DHX29 | -3.542195051 | 0.988902243 | 0.000341043 | 0.006121876 |
| SPRR2E | 3.488435939 | 0.974169501 | 0.000342369 | 0.006135185 |
| LRIG1 | -3.382248593 | 0.944731148 | 0.00034344 | 0.006142955 |
| NCF4 | 2.495706027 | 0.697180621 | 0.000343973 | 0.006142955 |
| GCNT3 | 3.386120407 | 0.946044398 | 0.000344594 | 0.006143607 |
| MYZAP | -3.530858412 | 0.987216868 | 0.000348121 | 0.006195965 |
| LOC344887 | 3.357562916 | 0.93986088 | 0.000353719 | 0.006265094 |
| NFATC4 | -3.573370297 | 1.000275455 | 0.000353743 | 0.006265094 |
| NDNF | -3.524672158 | 0.986766515 | 0.000354345 | 0.006265094 |
| HEXA | -2.93868378 | 0.822721302 | 0.000354392 | 0.006265094 |
| SPSB3 | -3.585155315 | 1.004020102 | 0.000355892 | 0.006281047 |
| UBXN1 | -2.549415083 | 0.714790512 | 0.000361559 | 0.006370361 |
| SLC39A8 | 2.301024845 | 0.645684703 | 0.000365668 | 0.006428097 |
| IL18RAP | 2.252377032 | 0.632083748 | 0.000366061 | 0.006428097 |
| CCL3 | 3.536516753 | 0.992898592 | 0.000368306 | 0.00645673 |
| SFN | -3.580148265 | 1.005326259 | 0.000369191 | 0.006461461 |
| SMAD7 | -3.288583177 | 0.924171802 | 0.000373105 | 0.006519095 |
| CXCR2P1 | 3.567842954 | 1.003348901 | 0.000376638 | 0.006566401 |
| ALPK1 | 2.649050548 | 0.745028214 | 0.000377063 | 0.006566401 |
| RPL41 | -2.242902644 | 0.630984723 | 0.000378545 | 0.006581286 |
| RPL18 | -1.880843324 | 0.529280936 | 0.000380022 | 0.006596058 |
| DAAM1 | -3.509459794 | 0.987797815 | 0.000381137 | 0.006604487 |
| CRAMP1L | -3.507631858 | 0.987589001 | 0.000382733 | 0.006621223 |
| RPS6KA2 | -2.705672251 | 0.76238402 | 0.000386752 | 0.006679746 |
| SULT1B1 | 3.537269359 | 0.996855751 | 0.00038754 | 0.006682363 |
| URB1 | -3.525506165 | 0.993933745 | 0.00038961 | 0.006707042 |
| MICAL1 | -3.300753699 | 0.931303382 | 0.000393761 | 0.006759476 |
| PAN2 | -3.519066857 | 0.993041966 | 0.000394518 | 0.006759476 |
| AKAP1 | -3.515078716 | 0.991997046 | 0.000394948 | 0.006759476 |
| ROBO3 | -3.338412439 | 0.942189873 | 0.000395231 | 0.006759476 |
| CTSE | 3.013616057 | 0.851018188 | 0.000398328 | 0.006801378 |
| RP2 | 2.425199667 | 0.68497935 | 0.000399303 | 0.006806945 |
| SLC38A5 | -3.397799591 | 0.959990217 | 0.000401022 | 0.006825165 |
| RNASE3 | 3.705960895 | 1.047449944 | 0.000403049 | 0.006848574 |
| MGC12916 | 3.482792797 | 0.984647645 | 0.000404553 | 0.006863028 |
| UTP6 | -3.078039083 | 0.870671735 | 0.000407395 | 0.006900091 |
| KLHL42 | -3.501747199 | 0.990973669 | 0.000409874 | 0.006930901 |
| TLR7 | 3.408823817 | 0.965566111 | 0.000414949 | 0.007005439 |
| GADD45G | -3.513568908 | 0.996676982 | 0.00042303 | 0.007130395 |
| MEG3 | -3.494974792 | 0.991570497 | 0.000423985 | 0.007135039 |
| ANKZF1 | -3.105359401 | 0.881582056 | 0.000427522 | 0.007183046 |
| ME3 | -3.495299781 | 0.992430885 | 0.000428372 | 0.007184447 |
| GOLGA8A | -2.999885221 | 0.851956276 | 0.000429643 | 0.007184447 |
| LUZP1 | -3.145079967 | 0.893193365 | 0.000429657 | 0.007184447 |
| RGS12 | -3.492957486 | 0.992319076 | 0.000431556 | 0.007204722 |
| TSSC4 | -3.350027082 | 0.952445129 | 0.000435975 | 0.00725576 |
| PGF | -3.52833137 | 1.003142287 | 0.000435995 | 0.00725576 |
| LYN | 2.547134497 | 0.724730613 | 0.000440425 | 0.007317686 |
| CD69 | 3.001837711 | 0.854234662 | 0.000441302 | 0.007317686 |
| HCFC1R1 | -3.318024083 | 0.944293641 | 0.000441807 | 0.007317686 |
| TRAF5 | -3.477269068 | 0.989943685 | 0.000443757 | 0.007336616 |
| CECR1 | 2.676536131 | 0.76212668 | 0.000444864 | 0.007336616 |
| EHMT1 | -3.245766348 | 0.924239943 | 0.000445046 | 0.007336616 |
| MUL1 | -3.266790571 | 0.930579537 | 0.000447281 | 0.007344579 |
| ATP5EP2 | 3.46203708 | 0.98626183 | 0.000447666 | 0.007344579 |
| HPS4 | -3.479479545 | 0.991239255 | 0.000447716 | 0.007344579 |
| SETD8 | -3.473909195 | 0.989842442 | 0.000448853 | 0.007344579 |
| C4orf3 | 2.324150715 | 0.662254003 | 0.000449026 | 0.007344579 |
| PHF17 | -3.535121512 | 1.007539181 | 0.000450355 | 0.007354864 |
| ZNF330 | -3.029124391 | 0.863593696 | 0.000452202 | 0.007361656 |
| ASAP2 | -3.484213718 | 0.993343603 | 0.000452234 | 0.007361656 |
| OBFC1 | -3.483403993 | 0.993330736 | 0.000453544 | 0.007361656 |
| INPP5K | -3.308151893 | 0.943360579 | 0.000453575 | 0.007361656 |
| GRAP | -3.467855053 | 0.989866563 | 0.000459435 | 0.00743907 |
| ROBO4 | -2.814664831 | 0.80347067 | 0.000459819 | 0.00743907 |
| HSPB6 | 3.326963385 | 0.949812889 | 0.00046047 | 0.00743907 |
| BAIAP2L1 | -3.102275479 | 0.886282198 | 0.000464692 | 0.007495742 |
| TUBA1C | -2.834400945 | 0.80985367 | 0.000465445 | 0.007496385 |
| ITSN1 | -3.462931525 | 0.989785582 | 0.000467588 | 0.007511648 |
| POLR2K | 1.964142179 | 0.561418665 | 0.000467824 | 0.007511648 |
| SLC9B2 | 2.060385838 | 0.589231047 | 0.000470986 | 0.007550883 |
| REV3L | -3.227183076 | 0.923231998 | 0.000473125 | 0.00757361 |
| LY6E | 2.194194305 | 0.627911051 | 0.000475066 | 0.007593109 |
| DNAAF3 | 3.269277454 | 0.936115208 | 0.000478722 | 0.00763309 |
| HSPA8 | -2.101862406 | 0.601869808 | 0.000479022 | 0.00763309 |
| MZT2B | -3.487491023 | 0.999573414 | 0.000484869 | 0.00770647 |
| TANC1 | -2.900081644 | 0.831241696 | 0.000485094 | 0.00770647 |
| EEF1A1 | -2.072754538 | 0.594608624 | 0.000490459 | 0.007779918 |
| SMAD1 | -3.450257242 | 0.990604731 | 0.000495864 | 0.007853797 |
| ATAD3B | -3.465653648 | 0.995168049 | 0.000496791 | 0.007856627 |
| DNMT1 | -3.092168845 | 0.888097382 | 0.000498074 | 0.007865069 |
| CANT1 | -3.136772647 | 0.901262537 | 0.000500627 | 0.007893524 |
| QARS | -3.123585714 | 0.898027532 | 0.000504654 | 0.007945085 |
| RALBP1 | -3.161381337 | 0.909638114 | 0.00051004 | 0.008017856 |
| FCGR3B | 3.480793726 | 1.002361945 | 0.000515459 | 0.008090934 |
| G6PD | 2.120347108 | 0.610826645 | 0.000517994 | 0.008118585 |
| SSH1 | -2.50541644 | 0.72185378 | 0.000518893 | 0.008120565 |
| EIF3G | -2.66115361 | 0.767150714 | 0.000522633 | 0.008156428 |
| HDAC3 | -2.868901255 | 0.827142177 | 0.00052347 | 0.008156428 |
| PER3 | -3.443142408 | 0.992710126 | 0.000523515 | 0.008156428 |
| PJA1 | -3.444525992 | 0.993398746 | 0.000525489 | 0.008175057 |
| PMM2 | -3.033551644 | 0.875190284 | 0.000527944 | 0.008201099 |
| PSMC3 | -2.361120604 | 0.681392427 | 0.000529954 | 0.008220161 |
| TSC1 | -3.212417104 | 0.927360251 | 0.000532119 | 0.00824156 |
| TSPAN18 | -3.481598236 | 1.006803758 | 0.000544059 | 0.008414089 |
| ATP6V0A1 | -2.93973145 | 0.850206227 | 0.000544871 | 0.008414251 |
| SHFM1 | -2.489885327 | 0.720189035 | 0.000545685 | 0.008414449 |
| PDRG1 | -3.200544912 | 0.925955152 | 0.000547281 | 0.008426683 |
| AIM2 | 3.221476317 | 0.932456572 | 0.000550646 | 0.008466076 |
| PTK7 | -3.431070654 | 0.993732397 | 0.000554984 | 0.008490446 |
| SSH2 | -3.156902577 | 0.91442909 | 0.000555786 | 0.008490446 |
| DYNLT1 | 2.353518741 | 0.681724151 | 0.000555822 | 0.008490446 |
| ARHGDIB | 2.675234443 | 0.774958022 | 0.000556237 | 0.008490446 |
| NT5DC2 | -3.489866501 | 1.010944679 | 0.000556273 | 0.008490446 |
| C10orf2 | -3.423279556 | 0.992107866 | 0.000559525 | 0.008527687 |
| CCDC84 | -3.422603601 | 0.992242519 | 0.000561913 | 0.008551663 |
| AFG3L2 | -2.572720907 | 0.746602991 | 0.000569156 | 0.008641287 |
| SESN2 | -3.441627486 | 0.998825462 | 0.000569636 | 0.008641287 |
| CCDC137 | -3.418000677 | 0.992055167 | 0.000570271 | 0.008641287 |
| MRPL55 | -3.446665128 | 1.000617744 | 0.000572038 | 0.008655574 |
| MLLT4 | -2.75890984 | 0.801048823 | 0.000572917 | 0.008656408 |
| COL16A1 | -3.415160661 | 0.992275718 | 0.000577973 | 0.008720256 |
| LDLRAP1 | -3.425671524 | 0.995516077 | 0.000579352 | 0.008728514 |
| ATP6V0C | 1.900847439 | 0.553035671 | 0.000587945 | 0.008828502 |
| TSFM | -3.410686744 | 0.99234636 | 0.000588212 | 0.008828502 |
| CBX7 | -3.492022528 | 1.016079921 | 0.000588718 | 0.008828502 |
| PIK3AP1 | 2.949277036 | 0.858229103 | 0.000589351 | 0.008828502 |
| CCDC85C | -3.409759129 | 0.992503837 | 0.000591435 | 0.00883884 |
| GSK3A | 1.912798086 | 0.556859426 | 0.000592609 | 0.00883884 |
| TREML4 | 3.491636391 | 1.016536822 | 0.000592914 | 0.00883884 |
| CCM2 | -2.791000755 | 0.812610816 | 0.000593408 | 0.00883884 |
| SNHG15 | -2.783800277 | 0.81090524 | 0.000597044 | 0.00888039 |
| BRD9 | -2.984531095 | 0.869553723 | 0.000598582 | 0.008890678 |
| B4GALT2 | -3.406934722 | 0.993318591 | 0.000603913 | 0.008957188 |
| ZNF3 | -3.404215267 | 0.992875689 | 0.000606609 | 0.008976436 |
| SELENBP1 | -3.20105992 | 0.93366116 | 0.00060692 | 0.008976436 |
| TRMT61A | -3.401719805 | 0.992745857 | 0.000611241 | 0.009027628 |
| SLC4A1AP | -3.401143508 | 0.992774452 | 0.000612772 | 0.00903752 |
| NR4A1 | -3.136370834 | 0.916049521 | 0.000617519 | 0.009092603 |
| DPP7 | -2.869574177 | 0.838224077 | 0.000618436 | 0.009092603 |
| DDX56 | -2.617346471 | 0.764612038 | 0.000619104 | 0.009092603 |
| PSMD2 | -2.61161698 | 0.763133319 | 0.0006211 | 0.009109174 |
| GINS4 | 3.233445876 | 0.945082226 | 0.000623138 | 0.009126321 |
| FBXO31 | -3.29654973 | 0.963910227 | 0.000626267 | 0.009159371 |
| CEACAM6 | 2.367876088 | 0.692673911 | 0.000629772 | 0.009197829 |
| CD48 | 2.634951207 | 0.771346023 | 0.000635382 | 0.009266871 |
| UCK1 | -3.420743518 | 1.001536 | 0.000636657 | 0.009272581 |
| NF2 | -3.160689741 | 0.926524873 | 0.000646448 | 0.009402154 |
| HBA2 | 3.209885728 | 0.941268047 | 0.00064922 | 0.009429401 |
| OTUD7B | -3.202658422 | 0.940645433 | 0.000662257 | 0.009605472 |
| NSUN4 | -3.384990898 | 0.994495754 | 0.000664734 | 0.009628104 |
| PDXK | -2.549058196 | 0.749014087 | 0.000665971 | 0.009632734 |
| FAM199X | -3.384153534 | 0.994755626 | 0.000668959 | 0.009646812 |
| NDUFS6 | -2.588476861 | 0.760886686 | 0.000669138 | 0.009646812 |
| TMEM243 | -3.384170033 | 0.994849032 | 0.000669701 | 0.009646812 |
| HCAR2 | 2.777695166 | 0.816711507 | 0.00067122 | 0.009655452 |
| FUT1 | -3.42386937 | 1.00693267 | 0.000673129 | 0.009669666 |
| CLCN6 | -3.37995791 | 0.994403117 | 0.000676373 | 0.009696774 |
| UBIAD1 | -3.381189178 | 0.994892209 | 0.000677445 | 0.009696774 |
| MAP4 | -2.629513198 | 0.773747941 | 0.000677786 | 0.009696774 |
| DHDDS | -3.37952092 | 0.994600984 | 0.000679137 | 0.009702876 |
| FLNC | -2.43064896 | 0.71573628 | 0.000683754 | 0.009754245 |
| EPC1 | -2.920311783 | 0.860151441 | 0.000686002 | 0.009754245 |
| URGCP | -2.887820864 | 0.850592604 | 0.000686112 | 0.009754245 |
| NOTCH4 | -3.479845179 | 1.025010688 | 0.000686448 | 0.009754245 |
| ICAM4 | -3.393234735 | 0.999801891 | 0.000689031 | 0.009777717 |
| NUDCD3 | -2.771365853 | 0.816663062 | 0.000689995 | 0.009778183 |
| NFRKB | -3.379649972 | 0.996477068 | 0.000694862 | 0.009833892 |
| POLR1E | -3.150714339 | 0.929280957 | 0.000697687 | 0.009839706 |
| BANP | -3.377084967 | 0.99608459 | 0.00069801 | 0.009839706 |
| KRI1 | -3.144392951 | 0.927459126 | 0.000698084 | 0.009839706 |
| UNC13B | -2.82233679 | 0.832921881 | 0.000702819 | 0.009893163 |
| DOCK1 | -2.88198949 | 0.850771813 | 0.000705327 | 0.009915183 |
| H19 | -3.310543686 | 0.977816117 | 0.000710097 | 0.009961496 |
| GGA3 | -2.988432275 | 0.882718407 | 0.000710519 | 0.009961496 |
| NOD1 | -3.431717781 | 1.013886547 | 0.00071252 | 0.009969729 |
| SLC35B2 | -3.366212928 | 0.99458832 | 0.000713005 | 0.009969729 |
| RPRD1A | -3.143444214 | 0.929101292 | 0.000716159 | 0.010000515 |
| ASMTL | -3.389965537 | 1.002686802 | 0.000722536 | 0.010076166 |
| HIRA | -3.151519502 | 0.932899165 | 0.000729621 | 0.010161476 |
| PRPF39 | -3.138194986 | 0.92938585 | 0.000733789 | 0.01020598 |
| NR2C2 | -3.227803256 | 0.956045606 | 0.000734939 | 0.010208457 |
| ATF6B | -3.211925696 | 0.951520937 | 0.000736629 | 0.010213827 |
| ABTB1 | -3.424874282 | 1.014678192 | 0.000737271 | 0.010213827 |
| SFTPA2 | 1.872393499 | 0.555048733 | 0.000742498 | 0.010272696 |
| SNORA48 | 2.619780242 | 0.776879537 | 0.000745748 | 0.010304076 |
| ADNP2 | -3.223997165 | 0.956313176 | 0.000748204 | 0.010312109 |
| LCN2 | 2.968912558 | 0.880734368 | 0.000749091 | 0.010312109 |
| CTDP1 | -3.355539492 | 0.995501873 | 0.000749771 | 0.010312109 |
| PSMC1 | -2.772707737 | 0.822634344 | 0.000750257 | 0.010312109 |
| SRCAP | -2.739144974 | 0.813038114 | 0.000754348 | 0.010315032 |
| NELFA | -3.352866575 | 0.995221584 | 0.00075451 | 0.010315032 |
| DAAM2 | -3.411320731 | 1.012587007 | 0.000754644 | 0.010315032 |
| DPH1 | -3.362763124 | 0.998195829 | 0.000754849 | 0.010315032 |
| ACTL6A | -3.367366132 | 0.999619803 | 0.000755381 | 0.010315032 |
| CLCN7 | -3.351768323 | 0.995375665 | 0.00075897 | 0.010350583 |
| FCGR3A | 2.047958887 | 0.608359292 | 0.000761661 | 0.010366637 |
| HLA-H | -2.925000177 | 0.868960797 | 0.000762419 | 0.010366637 |
| ACSL1 | 2.668318254 | 0.792764408 | 0.000763109 | 0.010366637 |
| UPK3B | -3.350220747 | 0.995529397 | 0.0007647 | 0.01037483 |
| MYCT1 | -2.953508534 | 0.878014635 | 0.000768636 | 0.010414773 |
| TEX264 | -3.380836081 | 1.005164064 | 0.000769701 | 0.010415755 |
| PML | 1.860677871 | 0.553598942 | 0.000776447 | 0.010490364 |
| USP40 | -2.971496843 | 0.884167397 | 0.000777212 | 0.010490364 |
| CKLF | 3.053603611 | 0.909162431 | 0.000783101 | 0.010556283 |
| LOC152217 | -3.354626009 | 0.99988257 | 0.000793592 | 0.010683993 |
| KCNE1 | 2.821042396 | 0.841324152 | 0.000799124 | 0.010744686 |
| REST | -3.343810877 | 0.997410907 | 0.000800879 | 0.010754515 |
| MCAM | -2.917062107 | 0.870408217 | 0.000804121 | 0.010784256 |
| RFC1 | -3.114780734 | 0.92956119 | 0.000805763 | 0.01079249 |
| TBRG4 | -2.872021363 | 0.857273827 | 0.000807592 | 0.010803208 |
| THAP7 | -3.351386671 | 1.000599768 | 0.000809933 | 0.010820742 |
| TRAK2 | -2.921387516 | 0.872763884 | 0.000816079 | 0.010889006 |
| ARPC1B | -2.599506003 | 0.776689643 | 0.000817194 | 0.010890041 |
| IRF2 | 2.456457695 | 0.734137391 | 0.000819727 | 0.010909955 |
| ELAC2 | -2.996171899 | 0.895606463 | 0.000821607 | 0.010921128 |
| TOR1B | 2.236788286 | 0.668705233 | 0.000822962 | 0.010925315 |
| CLN3 | -3.348003528 | 1.001359233 | 0.000827409 | 0.010964406 |
| GKN2 | -3.335392868 | 0.997646071 | 0.000827995 | 0.010964406 |
| GOLGA3 | -2.774593805 | 0.830596416 | 0.000836326 | 0.011060778 |
| TPD52L2 | -2.291537779 | 0.686237547 | 0.000839965 | 0.011094938 |
| UROD | -3.105976838 | 0.930392074 | 0.000842767 | 0.011117963 |
| SPG7 | -3.042781766 | 0.911558694 | 0.000843841 | 0.011118166 |
| CDKN2AIP | -3.3827902 | 1.013636169 | 0.000846019 | 0.011132892 |
| CD320 | -3.3844821 | 1.014297066 | 0.000847562 | 0.011139239 |
| SGTA | -2.774272144 | 0.832357616 | 0.00085906 | 0.011276231 |
| TUBGCP2 | -2.784274704 | 0.835508762 | 0.00086091 | 0.011286417 |
| MRPL11 | -3.359316239 | 1.00896469 | 0.000870119 | 0.011392914 |
| MMP1 | -3.440619186 | 1.033576313 | 0.000872058 | 0.011402567 |
| HMGXB3 | -2.757392862 | 0.828409351 | 0.000873028 | 0.011402567 |
| PCNXL3 | -3.319033236 | 0.997464898 | 0.000876388 | 0.011432241 |
| GLRX | 2.445570222 | 0.735221903 | 0.000880065 | 0.011465954 |
| TGFBR1 | -2.957363936 | 0.88936899 | 0.00088343 | 0.011495531 |
| ESF1 | -3.314671334 | 0.996998342 | 0.000885293 | 0.011505528 |
| ORAI2 | -3.328424309 | 1.001510593 | 0.00088926 | 0.011542796 |
| NELFE | -2.393747866 | 0.720435469 | 0.000891698 | 0.011560154 |
| ECSIT | -3.317614642 | 0.998833896 | 0.000895389 | 0.011593685 |
| ADORA2A | -3.207514772 | 0.966195707 | 0.000901026 | 0.011652305 |
| ALPL | 2.822233648 | 0.850683649 | 0.000907923 | 0.011727057 |
| RHBDF1 | -2.8275016 | 0.852627231 | 0.000912432 | 0.011770815 |
| DNAJC18 | -3.318175715 | 1.000872533 | 0.000915504 | 0.011795958 |
| TMEM88 | -3.330668219 | 1.004810608 | 0.000917342 | 0.011802516 |
| HMGN3 | -3.367548085 | 1.016022488 | 0.000918261 | 0.011802516 |
| ZNF76 | -3.330820282 | 1.005227583 | 0.000921365 | 0.011827944 |
| LCN6 | -2.946208988 | 0.889270514 | 0.000922803 | 0.011831932 |
| SMAP2 | 2.325705014 | 0.702172016 | 0.000925791 | 0.011855767 |
| ARHGAP29 | -2.867587251 | 0.866087947 | 0.000929747 | 0.011891928 |
| ZNF593 | -2.754919072 | 0.833066712 | 0.00094314 | 0.012048562 |
| OR7E14P | 2.913457258 | 0.881489577 | 0.000949248 | 0.012111853 |
| MSC | -3.100729297 | 0.938791984 | 0.00095693 | 0.012195046 |
| RIOK2 | -3.302381155 | 1.000070669 | 0.000959475 | 0.012212663 |
| CAMK1 | -3.331221109 | 1.009203537 | 0.000963953 | 0.012247306 |
| DIDO1 | -2.638464477 | 0.799371456 | 0.000964529 | 0.012247306 |
| MTHFR | -3.166563075 | 0.959959583 | 0.000971537 | 0.012313651 |
| CPAMD8 | -3.29758209 | 0.999727908 | 0.000972099 | 0.012313651 |
| METTL23 | -3.291518296 | 0.99814019 | 0.000974971 | 0.012335149 |
| AMBRA1 | -3.290236691 | 0.998870335 | 0.000987873 | 0.012474741 |
| VPS8 | -3.288205366 | 0.998297378 | 0.000988381 | 0.012474741 |
| TCF21 | -3.063328333 | 0.930150047 | 0.000989941 | 0.012479434 |
| MTIF3 | -3.299160146 | 1.002120708 | 0.000994145 | 0.01251741 |
| FAM219B | -2.736409902 | 0.831382363 | 0.000996909 | 0.012537176 |
| GADD45B | -2.255624512 | 0.685701168 | 0.001003601 | 0.012606235 |
| ZSWIM8-AS1 | 3.197351876 | 0.972387054 | 0.001008491 | 0.01265252 |
| YPEL3 | 2.402621161 | 0.731360716 | 0.001019323 | 0.012773163 |
| TMEM141 | -3.301709559 | 1.005369608 | 0.001023176 | 0.012806158 |
| LEPRE1 | -2.952175938 | 0.899279979 | 0.001027736 | 0.012847924 |
| SLED1 | 3.344961955 | 1.019429558 | 0.001033629 | 0.012906233 |
| AGO1 | -3.290467244 | 1.00295059 | 0.001035179 | 0.01291023 |
| AEBP2 | -3.276433975 | 0.998867832 | 0.001037528 | 0.012915794 |
| PANK4 | -3.29008381 | 1.003075502 | 0.001038085 | 0.012915794 |
| PUS1 | -2.931021801 | 0.894737246 | 0.001053458 | 0.013091557 |
| PSMA4 | 2.156976066 | 0.658522239 | 0.001054828 | 0.013093082 |
| COG1 | -3.275899363 | 1.000348271 | 0.001057522 | 0.013111026 |
| STK19 | -3.272717056 | 0.999985133 | 0.001065007 | 0.013181059 |
| CLEC12A | 3.124778488 | 0.9548344 | 0.001065681 | 0.013181059 |
| RPS3A | -2.244262975 | 0.686115414 | 0.001071793 | 0.013223943 |
| PTBP2 | -3.26846308 | 0.999245467 | 0.00107194 | 0.013223943 |
| LYRM1 | 1.830767614 | 0.559770936 | 0.001073326 | 0.013223943 |
| ZGPAT | -2.956280258 | 0.903967448 | 0.001074186 | 0.013223943 |
| WDR91 | -3.274927834 | 1.001884133 | 0.001080164 | 0.013281974 |
| TTC14 | -3.273502833 | 1.001616357 | 0.001082261 | 0.013292193 |
| SSNA1 | -3.102474498 | 0.949400646 | 0.001083776 | 0.01329525 |
| PHF13 | -3.135955337 | 0.959870287 | 0.0010867 | 0.013315567 |
| HPGDS | 2.164307593 | 0.662890758 | 0.00109482 | 0.013399422 |
| SFTPC | -1.841864885 | 0.564565016 | 0.001104538 | 0.01349392 |
| RNF144A | -3.2726874 | 1.003183273 | 0.001105111 | 0.01349392 |
| ANKRD54 | -3.260133919 | 1.000165659 | 0.001115718 | 0.013607616 |
| MPRIP | -1.988513361 | 0.610421612 | 0.001123561 | 0.013687374 |
| OSGEP | -3.279132057 | 1.006714886 | 0.001124934 | 0.013688214 |
| MLH3 | -3.256153174 | 1.000064342 | 0.001130162 | 0.013735912 |
| KANK3 | -3.173311463 | 0.975222932 | 0.001138186 | 0.013817441 |
| PCNXL4 | -3.262895681 | 1.0029661 | 0.001140946 | 0.013827672 |
| MPHOSPH8 | -2.712278211 | 0.833760083 | 0.001141662 | 0.013827672 |
| HLA-B | 1.866566606 | 0.573911745 | 0.001144517 | 0.013846278 |
| TNIP3 | 2.699993183 | 0.830326053 | 0.001147064 | 0.013861122 |
| ITGB3 | -3.357595664 | 1.032668784 | 0.001148474 | 0.013862214 |
| KCNAB2 | -3.268152895 | 1.005265547 | 0.001149859 | 0.013863 |
| CDH2 | 3.116525092 | 0.960458961 | 0.001175212 | 0.014146272 |
| BIN1 | -2.698695916 | 0.831819169 | 0.001177273 | 0.014146272 |
| 2-Mar | -3.31656647 | 1.022274898 | 0.001177397 | 0.014146272 |
| OTUD3 | -3.306703509 | 1.019534656 | 0.001181347 | 0.014177515 |
| RPS6KB2 | -3.271285533 | 1.009461034 | 0.001192676 | 0.014297137 |
| SEMA6D | -3.238303277 | 1.000883274 | 0.001214531 | 0.014526963 |
| PLXNB1 | -3.043533271 | 0.94069022 | 0.001214615 | 0.014526963 |
| PHLDA3 | -3.258697447 | 1.007391153 | 0.001217328 | 0.014542847 |
| ISG20L2 | -3.017186917 | 0.933047952 | 0.001222024 | 0.014578321 |
| TUBA1A | -2.682475972 | 0.829679692 | 0.001224348 | 0.014578321 |
| GBP1 | 2.917759124 | 0.902459351 | 0.001224462 | 0.014578321 |
| TNFAIP6 | 2.754840348 | 0.852658467 | 0.001234081 | 0.01464908 |
| HERC2 | -2.765956214 | 0.856125554 | 0.001234515 | 0.01464908 |
| SH3BP5 | -3.067908375 | 0.949591697 | 0.001234591 | 0.01464908 |
| KCTD11 | -3.234452362 | 1.001442743 | 0.0012388 | 0.014667592 |
| ACSS1 | -3.236001069 | 1.00193255 | 0.001238944 | 0.014667592 |
| ACAD8 | -3.232614765 | 1.000988729 | 0.001240408 | 0.014668386 |
| DENND4C | -3.011416918 | 0.932855819 | 0.00124585 | 0.014716169 |
| PRKX | -3.010700479 | 0.93296919 | 0.001250913 | 0.014759367 |
| KLHL3 | -3.230639819 | 1.001312716 | 0.001253561 | 0.014774008 |
| SLC41A3 | -3.070357258 | 0.9518467 | 0.001256718 | 0.014786755 |
| FASTKD2 | -2.941610639 | 0.912013696 | 0.00125796 | 0.014786755 |
| NLRP1 | -3.095113454 | 0.959666921 | 0.001258867 | 0.014786755 |
| UQCC | -3.230577742 | 1.001913745 | 0.001262338 | 0.014810966 |
| FAR2 | 2.075546537 | 0.643927623 | 0.001267402 | 0.014853781 |
| PITPNB | -3.265445906 | 1.013690056 | 0.001275902 | 0.014936734 |
| XRN1 | 2.442913726 | 0.758554998 | 0.001279746 | 0.014965052 |
| SERPINB6 | -2.519531663 | 0.782654462 | 0.001285428 | 0.015014774 |
| RANBP10 | -3.017521753 | 0.93759304 | 0.001289212 | 0.01503698 |
| NEIL2 | -3.238383773 | 1.006286693 | 0.001290193 | 0.01503698 |
| MPLKIP | -3.22842393 | 1.004389839 | 0.001307568 | 0.015222582 |
| ARHGAP9 | 2.053102992 | 0.638898715 | 0.001311264 | 0.015248707 |
| ATAD3A | -3.224652027 | 1.003660513 | 0.001314061 | 0.015264326 |
| EGR2 | -3.223170798 | 1.003394643 | 0.001316922 | 0.015275711 |
| SIDT2 | -3.240301767 | 1.008798003 | 0.00131795 | 0.015275711 |
| CCL2 | 2.542431284 | 0.791752451 | 0.001322076 | 0.015306635 |
| TPT1-AS1 | -3.21597985 | 1.001801855 | 0.001326447 | 0.015326575 |
| STARD3 | -2.680213347 | 0.834921789 | 0.001326717 | 0.015326575 |
| MCTS1 | 2.068264572 | 0.644785955 | 0.001338121 | 0.015426525 |
| JAM3 | -2.261579052 | 0.705060982 | 0.001338307 | 0.015426525 |
| CERS5 | -2.64383046 | 0.824937457 | 0.00135116 | 0.015557601 |
| ZBTB5 | -3.277585332 | 1.022876949 | 0.001354002 | 0.015573246 |
| PPAP2B | -2.72593152 | 0.851301219 | 0.001364408 | 0.015675766 |
| RPIA | -2.846802315 | 0.889369494 | 0.001369886 | 0.015721505 |
| LRRC32 | -2.510886362 | 0.784719861 | 0.001375596 | 0.015769799 |
| CEP250 | -3.209495002 | 1.003259774 | 0.001378732 | 0.015788515 |
| GPSM3 | 2.216770213 | 0.693212013 | 0.001384686 | 0.015839423 |
| ENDOD1 | -3.254199664 | 1.018374446 | 0.001395963 | 0.015951045 |
| PTMS | 2.461756885 | 0.770759161 | 0.001403463 | 0.016019311 |
| DGKE | -3.198753082 | 1.002929964 | 0.001425644 | 0.016239059 |
| ZNF263 | -2.59554216 | 0.813840413 | 0.001426415 | 0.016239059 |
| RNF168 | -3.199192633 | 1.003176811 | 0.001427355 | 0.016239059 |
| LINC00312 | -3.251709298 | 1.020118768 | 0.001434692 | 0.016287976 |
| RAB8B | 2.266427186 | 0.711065681 | 0.001435745 | 0.016287976 |
| QSOX1 | -2.450910274 | 0.76897246 | 0.001436308 | 0.016287976 |
| CSPG4 | -2.955281786 | 0.927488616 | 0.001440918 | 0.016322628 |
| EIF2AK4 | -2.986040947 | 0.937304785 | 0.001443675 | 0.016336237 |
| SERTAD3 | -2.638551601 | 0.828385777 | 0.001446677 | 0.016346924 |
| SRSF10 | -3.204602552 | 1.006166799 | 0.001447733 | 0.016346924 |
| STX11 | 1.97258848 | 0.619554765 | 0.001453149 | 0.016390456 |
| VSIG10L | 3.04441642 | 0.956765606 | 0.00146268 | 0.016464833 |
| PIGR | 2.159532498 | 0.678682435 | 0.001462879 | 0.016464833 |
| PPP2R5B | -2.995762052 | 0.941838609 | 0.001468895 | 0.016497889 |
| GCFC2 | -3.191854519 | 1.003492068 | 0.001468958 | 0.016497889 |
| MYO1G | -2.861725643 | 0.899968989 | 0.001473743 | 0.016520419 |
| MGC2752 | -3.190429533 | 1.00336411 | 0.00147411 | 0.016520419 |
| LST1 | 2.851727128 | 0.89742896 | 0.00148467 | 0.016621026 |
| ING5 | -3.188011829 | 1.003961189 | 0.001496129 | 0.016718213 |
| IL17C | 4.221613155 | 1.329493504 | 0.001496536 | 0.016718213 |
| LMNB1 | 2.617374495 | 0.824377976 | 0.001498525 | 0.01672265 |
| HCST | 2.318975639 | 0.730712018 | 0.001505696 | 0.01678483 |
| MTCH1 | -2.185722031 | 0.689521798 | 0.001524862 | 0.016980459 |
| ZNF394 | -2.825072487 | 0.891474191 | 0.001529698 | 0.01699683 |
| FOXK2 | -2.522232501 | 0.796006111 | 0.001531701 | 0.01699683 |
| SNORA70 | -3.18063814 | 1.003806831 | 0.001531877 | 0.01699683 |
| ITGA10 | -3.184617444 | 1.005118586 | 0.001532806 | 0.01699683 |
| GNA12 | -2.571684226 | 0.812101981 | 0.001541787 | 0.017070641 |
| FAM168B | -2.186978697 | 0.690703655 | 0.001543886 | 0.017070641 |
| EMP1 | -1.920219422 | 0.606470625 | 0.001544339 | 0.017070641 |
| SEPT8 | -2.628567382 | 0.830414841 | 0.001548877 | 0.017102792 |
| CLPP | -2.639240476 | 0.833934712 | 0.001551871 | 0.01710776 |
| PTGIS | -3.002459168 | 0.948742965 | 0.001552585 | 0.01710776 |
| ZBTB47 | -3.195432964 | 1.01006991 | 0.001558436 | 0.017154235 |
| ADRA2A | 2.499828207 | 0.790270103 | 0.00156014 | 0.017155007 |
| PPP2R4 | -3.092634534 | 0.977808306 | 0.001562473 | 0.017162691 |
| SSSCA1 | -2.7098461 | 0.856902142 | 0.001564878 | 0.017171141 |
| TMEM39B | -3.186316305 | 1.008373403 | 0.001578463 | 0.017302129 |
| HP | 2.24247731 | 0.709910405 | 0.001584107 | 0.017345893 |
| TPCN1 | -2.959143873 | 0.936888052 | 0.001585932 | 0.017347784 |
| MAPKBP1 | -3.055974667 | 0.9679464 | 0.001593066 | 0.017401387 |
| CTSC | 1.886101435 | 0.59743934 | 0.001594147 | 0.017401387 |
| SLC22A23 | -3.192283827 | 1.011408946 | 0.001597986 | 0.017425186 |
| TRMU | -3.170018412 | 1.004623542 | 0.001602622 | 0.017457606 |
| TAZ | -3.210199798 | 1.01751345 | 0.001605279 | 0.017459895 |
| ITM2A | -3.228300848 | 1.023302566 | 0.001606157 | 0.017459895 |
| NCALD | -2.875985781 | 0.912067177 | 0.001614574 | 0.017529581 |
| SERGEF | -3.18258672 | 1.009377223 | 0.001615907 | 0.017529581 |
| TMEM125 | -3.17899136 | 1.008613924 | 0.001622442 | 0.017557673 |
| PLEKHO1 | -2.77868089 | 0.881645253 | 0.001623232 | 0.017557673 |
| APOL6 | 2.114181139 | 0.67081741 | 0.001623512 | 0.017557673 |
| DLGAP1-AS1 | 2.316743431 | 0.735372227 | 0.001630267 | 0.017612591 |
| TNXB | -3.257438297 | 1.034712392 | 0.001643027 | 0.017732201 |
| SFTA3 | -3.203382215 | 1.018019515 | 0.001651353 | 0.017789332 |
| ACVR1 | -3.183662355 | 1.011772939 | 0.001651709 | 0.017789332 |
| LIN37 | -3.160532179 | 1.004607706 | 0.001654995 | 0.017806462 |
| SEZ6L2 | 2.927111961 | 0.930795746 | 0.001662337 | 0.017867142 |
| RAB11FIP3 | -3.180712906 | 1.011765229 | 0.001668114 | 0.017910906 |
| DCAF5 | -2.541665502 | 0.809110385 | 0.001681946 | 0.018026421 |
| STUB1 | -3.200698999 | 1.018926518 | 0.001682306 | 0.018026421 |
| AFG3L1P | -3.155957963 | 1.004783554 | 0.001684105 | 0.018027303 |
| HECTD4 | -2.940637923 | 0.936354732 | 0.001686499 | 0.018034552 |
| SURF6 | -2.795488173 | 0.890388617 | 0.001691631 | 0.01807102 |
| VHL | 2.109680996 | 0.672722418 | 0.001712488 | 0.018275241 |
| TRIT1 | -3.164197672 | 1.009095835 | 0.001714585 | 0.018279039 |
| TXLNG2P | -3.159752858 | 1.007892606 | 0.001718486 | 0.018302047 |
| SLC6A8 | -3.159550631 | 1.007955161 | 0.001720802 | 0.01830815 |
| SUPT6H | -2.165969275 | 0.6912758 | 0.001728571 | 0.01837219 |
| KCNK3 | -3.244323907 | 1.036071306 | 0.001739921 | 0.018435522 |
| C1orf216 | -3.1680281 | 1.011721039 | 0.00174019 | 0.018435522 |
| REPS2 | 2.682852915 | 0.85680746 | 0.001740815 | 0.018435522 |
| PIAS2 | -3.150508865 | 1.006200014 | 0.001741552 | 0.018435522 |
| ZCCHC11 | -3.15665498 | 1.008264542 | 0.001743424 | 0.018436754 |
| LMNA | -2.481274893 | 0.792780293 | 0.00174902 | 0.018474797 |
| PCGF3 | -2.504724147 | 0.800354284 | 0.001750926 | 0.018474797 |
| MUM1 | -3.179884 | 1.016245215 | 0.001753713 | 0.018474797 |
| NUPR1 | -2.337113528 | 0.746966131 | 0.00175517 | 0.018474797 |
| PAK1 | 2.042938542 | 0.652967343 | 0.001755818 | 0.018474797 |
| PRKCZ | -3.150876502 | 1.007292539 | 0.001759613 | 0.01849619 |
| TOE1 | -3.150063414 | 1.00727964 | 0.001764211 | 0.018517495 |
| TBP | -3.14530686 | 1.005809842 | 0.001765166 | 0.018517495 |
| PPP2R5D | -2.948824545 | 0.943272583 | 0.001771029 | 0.018560456 |
| CCDC22 | -3.142562437 | 1.005506333 | 0.001775917 | 0.018593123 |
| ANKRD11 | -2.818985792 | 0.902280305 | 0.001782344 | 0.018641826 |
| SOS1 | -2.780662943 | 0.890448818 | 0.001791604 | 0.01872003 |
| PALD1 | -2.933577562 | 0.939685049 | 0.001797043 | 0.018758202 |
| C11orf70 | 2.902467696 | 0.929969229 | 0.001802158 | 0.018792915 |
| ISYNA1 | -3.204034288 | 1.027040274 | 0.001810492 | 0.018852549 |
| SEMA3B | -2.922635081 | 0.936951093 | 0.00181279 | 0.018852549 |
| GGA1 | -2.757182448 | 0.88395931 | 0.00181387 | 0.018852549 |
| POU2AF1 | 2.752524759 | 0.88252064 | 0.001815058 | 0.018852549 |
| DDB2 | -3.159271118 | 1.013058124 | 0.001817441 | 0.018858643 |
| DDX47 | -2.514460957 | 0.806447811 | 0.00182116 | 0.018878579 |
| RPS19BP1 | -2.208675994 | 0.708723997 | 0.001830663 | 0.018940381 |
| CISD2 | 1.971349822 | 0.632621963 | 0.001832242 | 0.018940381 |
| CD8A | -3.143002474 | 1.008629821 | 0.001832533 | 0.018940381 |
| RBM19 | -2.806382405 | 0.900724389 | 0.00183512 | 0.018948473 |
| ST6GAL2 | 3.189965839 | 1.024088494 | 0.001839874 | 0.018961281 |
| FASTK | -3.004018235 | 0.964468081 | 0.001841389 | 0.018961281 |
| TACC1 | -2.806567168 | 0.901162066 | 0.001843277 | 0.018961281 |
| RBM10 | -2.339860566 | 0.751318824 | 0.001843583 | 0.018961281 |
| CST7 | 2.533581617 | 0.813597874 | 0.001845404 | 0.018961436 |
| CENPT | -2.904824945 | 0.933013747 | 0.001849586 | 0.018985833 |
| ATG16L1 | -2.772073543 | 0.890611225 | 0.001854775 | 0.019013943 |
| FLAD1 | -2.792014371 | 0.897071523 | 0.001855946 | 0.019013943 |
| GABRE | -3.130246946 | 1.005985103 | 0.001860616 | 0.01903798 |
| OXR1 | -3.129972226 | 1.005970998 | 0.001862063 | 0.01903798 |
| CWC22 | -2.967973411 | 0.953985794 | 0.001863731 | 0.01903798 |
| FAM160A2 | -2.657225557 | 0.854951954 | 0.001883321 | 0.019200913 |
| FLYWCH1 | -3.152767159 | 1.014391459 | 0.001883339 | 0.019200913 |
| NFATC3 | -2.67727432 | 0.861578326 | 0.001887365 | 0.019203994 |
| ITGB2-AS1 | 2.739447603 | 0.8816329 | 0.001888411 | 0.019203994 |
| MRPL4 | -2.678545089 | 0.86210076 | 0.001889979 | 0.019203994 |
| MILR1 | 2.906340608 | 0.935593268 | 0.00189371 | 0.019203994 |
| PPTC7 | -3.158043618 | 1.016636838 | 0.001894037 | 0.019203994 |
| HECTD1 | -2.517169129 | 0.810350119 | 0.001894614 | 0.019203994 |
| RAB4A | -3.156257182 | 1.016420351 | 0.001901069 | 0.019227236 |
| NAT9 | -2.598528982 | 0.836850161 | 0.001901951 | 0.019227236 |
| MAP3K6 | -3.236349313 | 1.042282131 | 0.0019024 | 0.019227236 |
| SMC3 | -2.968572324 | 0.956780147 | 0.001917839 | 0.019364638 |
| SNX25 | -3.128747855 | 1.008632274 | 0.001922369 | 0.019391738 |
| FAM105A | -3.15022127 | 1.015907616 | 0.001929377 | 0.019443751 |
| CWF19L2 | -3.14674623 | 1.01506728 | 0.001934964 | 0.019481359 |
| SFTPA1 | 1.673131794 | 0.539769616 | 0.001937072 | 0.019482282 |
| NDUFA11 | -2.534206241 | 0.817629467 | 0.001938766 | 0.019482282 |
| CCDC9 | -3.119759213 | 1.006726502 | 0.001942312 | 0.019490008 |
| MICALL2 | -3.123736588 | 1.008056388 | 0.001943247 | 0.019490008 |
| ISY1 | -3.12806801 | 1.009625765 | 0.001946704 | 0.019506048 |
| EIF4G1 | -1.851779517 | 0.597824863 | 0.001951417 | 0.019529222 |
| SYAP1 | 2.038052576 | 0.658047287 | 0.001954096 | 0.019529222 |
| VOPP1 | -2.746454756 | 0.886798202 | 0.001954596 | 0.019529222 |
| KCTD2 | -2.887736669 | 0.932651471 | 0.001959747 | 0.019554907 |
| BUD13 | -2.921664628 | 0.943661896 | 0.001960891 | 0.019554907 |
| COG4 | -2.985612467 | 0.964426339 | 0.001963229 | 0.019559649 |
| TEF | -3.118615654 | 1.007517611 | 0.001965834 | 0.019567035 |
| MECP2 | -2.478624579 | 0.800894945 | 0.001969333 | 0.019581082 |
| GK | 2.620559141 | 0.846824571 | 0.001970975 | 0.019581082 |
| ST3GAL3 | -3.120905294 | 1.008894377 | 0.001978829 | 0.019640532 |
| DCUN1D3 | 2.26288239 | 0.731598567 | 0.001981003 | 0.019643545 |
| C8orf58 | -3.113034667 | 1.006860525 | 0.001989313 | 0.019707339 |
| ATP1A1 | -2.043194346 | 0.661432766 | 0.002008023 | 0.019873937 |
| C19orf24 | -2.899764585 | 0.938940632 | 0.002012804 | 0.019902504 |
| PLAGL1 | -3.116340972 | 1.009283638 | 0.002017282 | 0.019914372 |
| TJAP1 | -2.595198178 | 0.840538167 | 0.002018182 | 0.019914372 |
| TRPC6 | -3.141612295 | 1.017585257 | 0.002019694 | 0.019914372 |
| ATP10A | -3.115099061 | 1.009295174 | 0.00202589 | 0.019917533 |
| C9orf114 | -2.899862711 | 0.939567511 | 0.002026095 | 0.019917533 |
| INTS10 | -2.709126133 | 0.877786708 | 0.002026541 | 0.019917533 |
| RNMTL1 | -3.107866173 | 1.007069047 | 0.002028342 | 0.019917533 |
| VKORC1L1 | 2.422282412 | 0.784971342 | 0.002029898 | 0.019917533 |
| FAM20B | -2.561359636 | 0.830205406 | 0.002034071 | 0.019917533 |
| RFXANK | -3.123329379 | 1.012407693 | 0.002035172 | 0.019917533 |
| GLDN | 2.809442471 | 0.910708979 | 0.002036234 | 0.019917533 |
| PRR14L | -2.93220812 | 0.9505506 | 0.002037254 | 0.019917533 |
| TEAD3 | -2.675896867 | 0.867531656 | 0.002038982 | 0.019917533 |
| PLEKHG3 | -3.130924034 | 1.015189297 | 0.002041833 | 0.019918506 |
| VPS11 | -2.649412524 | 0.859103431 | 0.002042875 | 0.019918506 |
| SLC35F2 | -3.121323779 | 1.012248825 | 0.002045441 | 0.019925022 |
| SVIP | -3.105572159 | 1.00735409 | 0.002049939 | 0.01995033 |
| PLLP | -3.155564349 | 1.023781574 | 0.002054331 | 0.019952856 |
| RASD2 | -3.155502685 | 1.02382826 | 0.002055717 | 0.019952856 |
| SNORA74B | 3.23871379 | 1.050835747 | 0.002055898 | 0.019952856 |
| PIN4 | -3.115036179 | 1.010849707 | 0.002058901 | 0.019963548 |
| KIAA1462 | -2.481622701 | 0.805742318 | 0.002070555 | 0.020058024 |
| TOP2A | 2.812153473 | 0.913287711 | 0.002075894 | 0.020091207 |
| ARC | -3.121690987 | 1.013938718 | 0.002078524 | 0.020091593 |
| PSEN1 | 1.966532127 | 0.638789067 | 0.002080237 | 0.020091593 |
| SMIM3 | -2.176886209 | 0.707165683 | 0.002081674 | 0.020091593 |
| PLEKHJ1 | -2.894719697 | 0.940498465 | 0.00208495 | 0.020104739 |
| RBM5 | -2.125240584 | 0.690702605 | 0.002091474 | 0.020149149 |
| PDE8B | -3.098853488 | 1.007490693 | 0.002099291 | 0.020205916 |
| PDGFRL | 2.591642078 | 0.843093635 | 0.002112327 | 0.020312775 |
| TMPRSS2 | -2.889094344 | 0.940022844 | 0.002116134 | 0.02033076 |
| ANKDD1A | -3.097440139 | 1.008097116 | 0.002122303 | 0.02037139 |
| CAPZA1 | 2.226521921 | 0.724767567 | 0.002125944 | 0.020387705 |
| TRAF2 | -2.935979212 | 0.95596259 | 0.002131802 | 0.02041049 |
| HINFP | -3.094621649 | 1.007699829 | 0.002133605 | 0.02041049 |
| GUSBP11 | -3.096524674 | 1.008409348 | 0.002135561 | 0.02041049 |
| MAP2K7 | -3.103115705 | 1.010640421 | 0.002137401 | 0.02041049 |
| SURF1 | -3.016269008 | 0.98238416 | 0.002138038 | 0.02041049 |
| KAZN | -2.823897155 | 0.920174708 | 0.002148696 | 0.020487606 |
| RUFY3 | -2.514456501 | 0.819391688 | 0.002150018 | 0.020487606 |
| FCGR1A | 2.600307494 | 0.848517232 | 0.002180115 | 0.020755561 |
| MTX1 | -3.110575057 | 1.01524831 | 0.002185037 | 0.020774721 |
| RMDN1 | -3.128893949 | 1.021275088 | 0.002186084 | 0.020774721 |
| TECPR1 | -3.137108114 | 1.024240235 | 0.0021923 | 0.020779923 |
| ORAOV1 | -3.088056702 | 1.008278793 | 0.002193489 | 0.020779923 |
| HLA-A | 1.998671809 | 0.652595459 | 0.002193861 | 0.020779923 |
| LAGE3 | -3.087594598 | 1.008246383 | 0.002196128 | 0.020779923 |
| LITAF | 1.95451989 | 0.638254913 | 0.002196525 | 0.020779923 |
| TNFRSF14 | -2.217528383 | 0.724381522 | 0.002203993 | 0.020831798 |
| NMRAL1 | -3.121247251 | 1.019962107 | 0.002212188 | 0.020885112 |
| ICAM2 | -3.131668429 | 1.023447158 | 0.002213947 | 0.020885112 |
| TNIP2 | -2.146941757 | 0.701684114 | 0.0022156 | 0.020885112 |
| RPL7A | -1.601246191 | 0.523388603 | 0.002217934 | 0.020888364 |
| PILRB | -2.679382316 | 0.875920379 | 0.002221263 | 0.020900974 |
| VSTM4 | -3.122110846 | 1.021156091 | 0.002232455 | 0.020987479 |
| DYRK3 | -3.132683817 | 1.024983854 | 0.002240682 | 0.021045977 |
| PELI1 | 2.343744929 | 0.767404807 | 0.002257232 | 0.021163196 |
| RNF113A | -2.876984453 | 0.942020103 | 0.002257681 | 0.021163196 |
| WDR5 | -2.579082633 | 0.844574834 | 0.00226034 | 0.021163196 |
| FAM207A | -3.082459699 | 1.009454983 | 0.002261223 | 0.021163196 |
| TMEM55B | -2.625440639 | 0.859945711 | 0.002265428 | 0.021183666 |
| GPRC5B | -3.091439699 | 1.012702126 | 0.002268195 | 0.021190669 |
| NCSTN | 1.777839139 | 0.582593322 | 0.002276287 | 0.021247364 |
| PKIA | -3.079795808 | 1.009934386 | 0.00229222 | 0.021377085 |
| RMND5B | -2.886628895 | 0.946752894 | 0.002296212 | 0.021395315 |
| DHX32 | -3.087728205 | 1.013795911 | 0.002321317 | 0.021592646 |
| POLR3C | -2.870291128 | 0.942412075 | 0.002321502 | 0.021592646 |
| PDLIM2 | -2.60196347 | 0.854551791 | 0.002328132 | 0.021614115 |
| GMPR | 2.323884137 | 0.763234807 | 0.002328485 | 0.021614115 |
| NPRL2 | -3.073521912 | 1.009503092 | 0.002329986 | 0.021614115 |
| KIAA1671 | -2.863664402 | 0.940689875 | 0.002332866 | 0.021621732 |
| EMC9 | -3.106746303 | 1.02066188 | 0.00233568 | 0.02162873 |
| PSMB1 | -2.019967308 | 0.664173986 | 0.002355414 | 0.02178185 |
| ZNF700 | -3.06888025 | 1.009101403 | 0.002356364 | 0.02178185 |
| PPM1M | -3.103088362 | 1.020478177 | 0.002359364 | 0.021789352 |
| NAPA | 1.850234255 | 0.608516048 | 0.002361326 | 0.021789352 |
| WHSC1 | -2.70244674 | 0.889293719 | 0.002374684 | 0.021893379 |
| PARP9 | 2.325653028 | 0.765446136 | 0.00237919 | 0.021915679 |
| BAIAP2 | -2.909545122 | 0.957726287 | 0.002381765 | 0.021920172 |
| CTSS | 2.000708837 | 0.659496129 | 0.002415801 | 0.022200505 |
| YWHAE | 1.6551088 | 0.545590219 | 0.002416453 | 0.022200505 |
| ZHX3 | -3.096454104 | 1.021457214 | 0.002434157 | 0.022309283 |
| KIAA0355 | -2.494992991 | 0.823063297 | 0.002434628 | 0.022309283 |
| RENBP | -3.066105957 | 1.011467098 | 0.002434667 | 0.022309283 |
| USP36 | -2.307701742 | 0.761404557 | 0.002438677 | 0.02231103 |
| CRYBB2P1 | -3.060254075 | 1.009719878 | 0.002439107 | 0.02231103 |
| MIR5047 | -2.979379691 | 0.983968212 | 0.002462411 | 0.02249696 |
| ARMC6 | -3.07365486 | 1.015157262 | 0.002463718 | 0.02249696 |
| SULT1A1 | -3.108153843 | 1.02720111 | 0.002479374 | 0.022588448 |
| MAPKAPK5 | -3.054870089 | 1.009595829 | 0.002479478 | 0.022588448 |
| NAA35 | -3.067777877 | 1.013890794 | 0.00248019 | 0.022588448 |
| DSTYK | -3.054697569 | 1.009757327 | 0.002484853 | 0.022611299 |
| STK16 | -3.061280599 | 1.012284707 | 0.002493492 | 0.02267027 |
| CTDSPL2 | -3.058544135 | 1.011924984 | 0.002506954 | 0.022743103 |
| CD55 | 2.097807852 | 0.694141099 | 0.00250975 | 0.022743103 |
| ARHGEF10L | -3.053585102 | 1.010411893 | 0.002510117 | 0.022743103 |
| EVI5 | -3.054234804 | 1.010628843 | 0.002510166 | 0.022743103 |
| C19orf25 | -3.057793059 | 1.011978697 | 0.00251444 | 0.022762187 |
| ARL13B | -2.896376992 | 0.959006985 | 0.002526217 | 0.022809749 |
| PACSIN3 | -3.055161213 | 1.011706327 | 0.00252933 | 0.022809749 |
| CA12 | 2.746821476 | 0.909625735 | 0.002530025 | 0.022809749 |
| ORC2 | -3.052837029 | 1.011015561 | 0.002531298 | 0.022809749 |
| CACNA1C | -3.071167411 | 1.017095685 | 0.002531537 | 0.022809749 |
| LOC399715 | 2.844488241 | 0.942069504 | 0.002532727 | 0.022809749 |
| PWWP2A | -3.05120638 | 1.011706964 | 0.002562183 | 0.023053848 |
| POU6F1 | -3.056234031 | 1.013455073 | 0.002564222 | 0.023053848 |
| JAG1 | -2.08021504 | 0.690075438 | 0.002574246 | 0.023124169 |
| GIMAP6 | -2.676369049 | 0.888001967 | 0.002578937 | 0.023146513 |
| ZNF513 | -3.043846761 | 1.010122401 | 0.002583854 | 0.023170841 |
| VPS18 | -2.834986897 | 0.941261678 | 0.002596173 | 0.023261447 |
| FAM53B | -3.062246154 | 1.017068603 | 0.002605132 | 0.023285867 |
| DCAF16 | -3.042267451 | 1.010447072 | 0.00260549 | 0.023285867 |
| PIK3R4 | -3.041845967 | 1.01035945 | 0.00260683 | 0.023285867 |
| R3HCC1 | -3.075528749 | 1.021584417 | 0.002607769 | 0.023285867 |
| NLE1 | -3.050370089 | 1.013727067 | 0.002620535 | 0.023379982 |
| NUMA1 | -2.212296233 | 0.735356404 | 0.002625684 | 0.023406036 |
| TTF1 | -3.042734419 | 1.011551411 | 0.002629836 | 0.023423162 |
| GPN2 | -3.038176207 | 1.01046476 | 0.002640901 | 0.023483731 |
| C15orf48 | 2.931411134 | 0.974963607 | 0.002641109 | 0.023483731 |
| SNX21 | -3.055946422 | 1.016491878 | 0.002643907 | 0.023488723 |
| PXN | -2.0590031 | 0.68502436 | 0.00264938 | 0.023517446 |
| CALML4 | -3.046091244 | 1.013759319 | 0.00265801 | 0.023574125 |
| EXOC2 | -2.837754204 | 0.944802864 | 0.002668575 | 0.023647856 |
| CDC34 | -2.49250272 | 0.829941232 | 0.002671322 | 0.023652241 |
| TBX2 | -3.107939147 | 1.035410507 | 0.002685214 | 0.023736562 |
| BPI | 2.823160344 | 0.940601589 | 0.002687049 | 0.023736562 |
| DYRK2 | -3.038833381 | 1.012480158 | 0.002687627 | 0.023736562 |
| EDNRA | -2.561131703 | 0.853863135 | 0.002704551 | 0.023865959 |
| CCDC86 | -2.626886803 | 0.8760847 | 0.002713662 | 0.023926251 |
| ARRB1 | -2.511952179 | 0.838256439 | 0.002729735 | 0.024047775 |
| LSM7 | -2.24127003 | 0.748232627 | 0.002740683 | 0.024119427 |
| PDE9A | -3.059123605 | 1.021347537 | 0.002742795 | 0.024119427 |
| FLJ23867 | -3.0277149 | 1.010934827 | 0.002744759 | 0.024119427 |
| DACT1 | -3.038901961 | 1.014963269 | 0.002752553 | 0.024159438 |
| MEDAG | -2.46075991 | 0.821910912 | 0.002753914 | 0.024159438 |
| CREBZF | -2.398257059 | 0.80144671 | 0.002767844 | 0.024245533 |
| C7orf26 | -3.033108205 | 1.013619254 | 0.002768345 | 0.024245533 |
| TMEM51 | -2.881558495 | 0.963574298 | 0.00278531 | 0.024373785 |
| CHMP6 | -3.033762106 | 1.014689501 | 0.00279121 | 0.02440508 |
| SNAPC2 | -3.025723453 | 1.012381891 | 0.00280151 | 0.02444493 |
| CCDC71L | 1.923720364 | 0.643751991 | 0.002805393 | 0.02444493 |
| SNHG9 | -3.040589434 | 1.017518937 | 0.002805903 | 0.02444493 |
| KHDRBS3 | -3.062033125 | 1.024817138 | 0.002809176 | 0.02444493 |
| RTKN2 | -2.633666417 | 0.881483056 | 0.002810233 | 0.02444493 |
| FOXJ2 | -2.544882242 | 0.851795896 | 0.002811159 | 0.02444493 |
| CUL9 | -3.03660519 | 1.016413594 | 0.002812063 | 0.02444493 |
| EZR | -1.948143069 | 0.652228517 | 0.002818195 | 0.024469054 |
| ZNF333 | -3.025238194 | 1.012900496 | 0.002819988 | 0.024469054 |
| HSDL1 | -3.021228977 | 1.011625733 | 0.002821829 | 0.024469054 |
| ZNF337 | -2.686233072 | 0.899894402 | 0.002835286 | 0.02456546 |
| C1QB | 2.025116823 | 0.678506122 | 0.002838845 | 0.024576016 |
| POPDC3 | 2.854913847 | 0.956619209 | 0.00284155 | 0.024579172 |
| MPST | -3.04309332 | 1.019787146 | 0.002844626 | 0.024585527 |
| UBL4A | -2.683455816 | 0.899353167 | 0.002847281 | 0.024588242 |
| MORC2 | -2.67372341 | 0.896205057 | 0.002850805 | 0.024598442 |
| GFER | -3.034521251 | 1.0173227 | 0.002855777 | 0.024621112 |
| FCN1 | 2.975826681 | 0.997982819 | 0.002865202 | 0.024682106 |
| RNF185 | -2.356064687 | 0.790553856 | 0.002879935 | 0.024788689 |
| RAI2 | -3.061741764 | 1.027708348 | 0.002890085 | 0.024855674 |
| TBC1D2B | -2.394770562 | 0.804140577 | 0.00290089 | 0.024928187 |
| EPHB4 | -3.030733757 | 1.01792788 | 0.002907461 | 0.024947333 |
| COL9A2 | 2.483134089 | 0.834018474 | 0.002907869 | 0.024947333 |
| CAB39L | -3.05475476 | 1.02611069 | 0.002910625 | 0.024950591 |
| ZBTB34 | -3.021765288 | 1.015175595 | 0.0029147 | 0.024965142 |
| HMOX1 | -2.422588392 | 0.814099096 | 0.002922344 | 0.0250029 |
| FAM174B | -3.014804586 | 1.013271324 | 0.002926848 | 0.0250029 |
| SIGLEC5 | 2.856067043 | 0.959947209 | 0.002927657 | 0.0250029 |
| ZMYND8 | -2.813853414 | 0.945791355 | 0.002928632 | 0.0250029 |
| SEMA6B | -3.056952467 | 1.027851538 | 0.002938314 | 0.025065179 |
| E4F1 | -3.018286112 | 1.015111114 | 0.002945632 | 0.025107206 |
| APC | -3.008546263 | 1.011966964 | 0.002949344 | 0.025118458 |
| RCC2 | -2.072697385 | 0.697479345 | 0.002961587 | 0.025201149 |
| GADD45A | -2.437440583 | 0.820283201 | 0.002963853 | 0.025201149 |
| PARP2 | -3.010893399 | 1.013415768 | 0.002967983 | 0.025215845 |
| CLEC3B | -3.119433867 | 1.051266637 | 0.00300418 | 0.025497254 |
| ARL5B | 2.738592468 | 0.922977641 | 0.003005961 | 0.025497254 |
| RCN2 | -2.863597624 | 0.96519716 | 0.003008652 | 0.025499483 |
| STAP2 | -3.038927284 | 1.024641643 | 0.003018535 | 0.025562614 |
| RNF123 | -2.796305174 | 0.943103958 | 0.003026809 | 0.025575054 |
| EED | -3.010651071 | 1.015399815 | 0.00302692 | 0.025575054 |
| PDE4B | 2.287317456 | 0.771452012 | 0.003027311 | 0.025575054 |
| CLEC1A | 2.06709705 | 0.697342305 | 0.003034215 | 0.025583725 |
| LSMD1 | -2.872381347 | 0.969038207 | 0.003035134 | 0.025583725 |
| GPD1L | -3.054344031 | 1.030443916 | 0.003035646 | 0.025583725 |
| TPSAB1 | 1.843543379 | 0.622282207 | 0.003051003 | 0.025692528 |
| HMGCL | -2.661539004 | 0.898707057 | 0.003061245 | 0.025751916 |
| CHRAC1 | 2.065520787 | 0.69749352 | 0.00306296 | 0.025751916 |
| PFKL | -2.114324296 | 0.714430144 | 0.003081831 | 0.025889846 |
| DCAF15 | -3.012010153 | 1.018167875 | 0.003093763 | 0.025969306 |
| FLOT1 | 1.787402766 | 0.604259199 | 0.003096354 | 0.025970297 |
| CDK10 | -2.691480432 | 0.910064417 | 0.003101835 | 0.025995509 |
| ZNF622 | -2.545543129 | 0.861222564 | 0.00311928 | 0.026120857 |
| TRIM11 | -2.992211436 | 1.012741627 | 0.003131097 | 0.026198925 |
| SFT2D1 | 1.936328389 | 0.655482447 | 0.003136325 | 0.026213648 |
| NADSYN1 | -2.482047873 | 0.84027725 | 0.003138427 | 0.026213648 |
| PLK2 | -2.194081648 | 0.742836056 | 0.003140346 | 0.026213648 |
| ST6GALNAC4 | -2.698330923 | 0.914174274 | 0.003160715 | 0.026357028 |
| NDRG2 | -3.023910704 | 1.024540196 | 0.003162542 | 0.026357028 |
| MGRN1 | -2.644659836 | 0.896421926 | 0.003175272 | 0.02642204 |
| GCHFR | -3.0464306 | 1.032619438 | 0.003175715 | 0.02642204 |
| SLC30A4 | 2.797435106 | 0.948287837 | 0.003177891 | 0.02642204 |
| MFSD11 | -2.987091473 | 1.012953028 | 0.00318913 | 0.026494502 |
| ASB6 | -2.67019878 | 0.905689442 | 0.003195786 | 0.026528817 |
| WISP2 | -3.074792131 | 1.043043952 | 0.003199377 | 0.026537644 |
| EIF3D | -1.999259256 | 0.67833633 | 0.003205642 | 0.026568624 |
| ILKAP | -2.428729095 | 0.824201867 | 0.003211173 | 0.026593475 |
| NME3 | -2.841487214 | 0.964554124 | 0.003220087 | 0.026646283 |
| NUP188 | -2.406957197 | 0.817696239 | 0.003244362 | 0.026826023 |
| DDB1 | -2.328387055 | 0.79138112 | 0.003259086 | 0.026926566 |
| YIPF1 | 1.777076876 | 0.604191667 | 0.003268939 | 0.026986732 |
| DUSP7 | -2.842347283 | 0.966534941 | 0.003274081 | 0.027007954 |
| GPN3 | -2.980254573 | 1.013550958 | 0.00327779 | 0.027017329 |
| CBFA2T2 | -2.984738351 | 1.015162988 | 0.003280462 | 0.027018143 |
| CAPRIN2 | -2.976641561 | 1.01334265 | 0.003309254 | 0.027233918 |
| PTDSS2 | -2.99939556 | 1.021290346 | 0.003315445 | 0.027262966 |
| MAST2 | -2.666464001 | 0.908000748 | 0.003317976 | 0.027262966 |
| ENOSF1 | -2.975922829 | 1.01352864 | 0.0033226 | 0.02727961 |
| CSNK1D | -1.942466361 | 0.661998409 | 0.003343588 | 0.027430482 |
| SNHG3 | -2.422911394 | 0.825954224 | 0.003351968 | 0.027444606 |
| CDR1 | 3.077076952 | 1.048979838 | 0.003352722 | 0.027444606 |
| FAM131A | -2.974691267 | 1.014090132 | 0.00335315 | 0.027444606 |
| EMP2 | -1.866186901 | 0.636427765 | 0.00336479 | 0.027518277 |
| PUF60 | -2.020700605 | 0.689178173 | 0.003367392 | 0.027518277 |
| GPATCH3 | -2.982099112 | 1.017252222 | 0.003373035 | 0.027531804 |
| METTL21D | -2.974263525 | 1.014619376 | 0.003374291 | 0.027531804 |
| SRL | -2.973646923 | 1.014636259 | 0.00338143 | 0.027568627 |
| C17orf96 | -3.003599273 | 1.024975878 | 0.003385153 | 0.027577577 |
| TRABD | -2.529761032 | 0.863493824 | 0.003393107 | 0.02762094 |
| PEX26 | -2.972661254 | 1.014814827 | 0.003397649 | 0.027636496 |
| RFX1 | 2.234276424 | 0.763061583 | 0.003411039 | 0.027705227 |
| MS4A7 | 2.097562163 | 0.716377743 | 0.003411376 | 0.027705227 |
| KCTD3 | -2.875939509 | 0.982510725 | 0.003421026 | 0.027757723 |
| C14orf132 | -3.008194596 | 1.027760215 | 0.003423127 | 0.027757723 |
| CYP3A5 | -3.056636892 | 1.044525361 | 0.003429753 | 0.027789997 |
| HIST1H2BD | 2.621913271 | 0.89657319 | 0.003451523 | 0.02793041 |
| ZCCHC7 | -2.972574815 | 1.016510773 | 0.003452402 | 0.02793041 |
| PKN1 | -2.552779682 | 0.873236878 | 0.003462836 | 0.027993261 |
| CAMP | 2.902890578 | 0.9937484 | 0.003487391 | 0.028162116 |
| PPP1R3C | -3.02276923 | 1.034840218 | 0.003489088 | 0.028162116 |
| TPST1 | -2.977347665 | 1.019421243 | 0.003493295 | 0.028174415 |
| RPL13 | -1.894404214 | 0.649127097 | 0.003518471 | 0.028355694 |
| NIPAL3 | -2.971252635 | 1.018536464 | 0.003532136 | 0.028443989 |
| STK40 | -2.143917101 | 0.735017187 | 0.003536132 | 0.028454346 |
| HIF3A | -3.035984071 | 1.041149325 | 0.003545585 | 0.028499354 |
| PWP2 | -2.395365746 | 0.82149687 | 0.003547153 | 0.028499354 |
| PLCG1 | -2.601189326 | 0.892231218 | 0.003552604 | 0.02852133 |
| LUC7L3 | -2.311367579 | 0.793151043 | 0.003566492 | 0.028610947 |
| ANKRD29 | -2.965679186 | 1.017877624 | 0.003572976 | 0.028641082 |
| ENKD1 | -2.960000765 | 1.016588631 | 0.003594681 | 0.028774804 |
| SEC23IP | -2.607505805 | 0.89556481 | 0.003596094 | 0.028774804 |
| HNRNPC | 1.878582812 | 0.645298509 | 0.003600617 | 0.028774804 |
| ZNF133 | -2.958853331 | 1.016411731 | 0.003601847 | 0.028774804 |
| KIAA0513 | -2.963288373 | 1.017981091 | 0.003603358 | 0.028774804 |
| TRUB2 | -2.953307779 | 1.014907358 | 0.003615115 | 0.028846752 |
| NELFB | -2.809480806 | 0.965675888 | 0.003621911 | 0.028879037 |
| UNC5B | -2.950571145 | 1.014716173 | 0.003640046 | 0.028998123 |
| RPL8 | -1.609562331 | 0.553574533 | 0.00364237 | 0.028998123 |
| PLCD1 | -2.971775594 | 1.022201288 | 0.003646434 | 0.029008491 |
| IRAK3 | 1.689713987 | 0.58134256 | 0.003654128 | 0.029047692 |
| C3orf58 | -2.948857674 | 1.014679595 | 0.003658523 | 0.02906063 |
| SLC26A6 | -2.952570387 | 1.016185002 | 0.003666151 | 0.02909921 |
| RPL7L1 | -2.949679825 | 1.015294637 | 0.003669655 | 0.029099691 |
| TIMM44 | -2.511116119 | 0.864504384 | 0.003676185 | 0.029099691 |
| ILK | -1.842324274 | 0.634273598 | 0.003676983 | 0.029099691 |
| ATF4 | -1.962846763 | 0.675773169 | 0.003677296 | 0.029099691 |
| RASL11A | -2.828582993 | 0.97413196 | 0.003687862 | 0.029161321 |
| ZNF10 | -2.945979166 | 1.014936997 | 0.003700522 | 0.029239412 |
| IDH2 | -2.396549798 | 0.825867022 | 0.003709553 | 0.02928873 |
| BTN3A2 | -2.176641779 | 0.750653416 | 0.003735643 | 0.029439181 |
| SSBP4 | -2.975135648 | 1.026230469 | 0.003742461 | 0.029439181 |
| FCRLA | 2.987541763 | 1.030529954 | 0.003743139 | 0.029439181 |
| MYOM2 | -2.948558039 | 1.017118067 | 0.003744338 | 0.029439181 |
| TNNC1 | -2.679116283 | 0.924173174 | 0.003744347 | 0.029439181 |
| MYCBP2 | -2.428029312 | 0.837585767 | 0.003745429 | 0.029439181 |
| FREM2 | 2.867506769 | 0.989484727 | 0.003755748 | 0.029489584 |
| HSPA2 | -2.872352366 | 0.991205624 | 0.003757458 | 0.029489584 |
| SGCA | -2.988731674 | 1.031608779 | 0.003765623 | 0.029531598 |
| ARHGAP4 | -2.98336601 | 1.030011913 | 0.003774248 | 0.029577147 |
| PDCD11 | -2.300514627 | 0.794408749 | 0.003780936 | 0.029607463 |
| KLHL24 | -2.621461346 | 0.905592622 | 0.003794645 | 0.029692671 |
| DCAF13 | -2.480912507 | 0.857234414 | 0.003802606 | 0.029724253 |
| ING2 | -2.938986856 | 1.015564001 | 0.003804342 | 0.029724253 |
| RASIP1 | -2.713702444 | 0.937839898 | 0.003808932 | 0.029737989 |
| NEAT1 | 1.77085224 | 0.612153949 | 0.003817979 | 0.02978648 |
| ZNF343 | -2.937086519 | 1.015422561 | 0.00382217 | 0.029797038 |
| ZNF318 | -2.938419431 | 1.016237556 | 0.003834454 | 0.029870622 |
| NFX1 | -2.382251412 | 0.824102733 | 0.003843587 | 0.029919574 |
| AFMID | -2.94556136 | 1.019475504 | 0.003861116 | 0.030033764 |
| GAPVD1 | -2.498492658 | 0.864945866 | 0.003869467 | 0.030076442 |
| TUBGCP6 | -2.543536843 | 0.880618715 | 0.003872662 | 0.030079012 |
| FAM49A | 1.665381782 | 0.576711187 | 0.003880422 | 0.030117011 |
| SYNE1 | -2.436938759 | 0.844663308 | 0.003912884 | 0.030346525 |
| ZBTB10 | -2.969018267 | 1.029383274 | 0.003923235 | 0.030404345 |
| ALAS1 | -2.393817756 | 0.830059558 | 0.003927699 | 0.030416498 |
| ARMC5 | -2.928913084 | 1.016290961 | 0.00395206 | 0.030582593 |
| LONP1 | -2.429678667 | 0.843218102 | 0.003958681 | 0.030611274 |
| USP20 | -2.753064381 | 0.956290455 | 0.003990651 | 0.030835783 |
| RFT1 | -2.924888495 | 1.016205932 | 0.003998959 | 0.030877254 |
| CNST | -2.629382048 | 0.913818479 | 0.004010216 | 0.03094142 |
| LMAN2L | -2.926232695 | 1.017240668 | 0.00401937 | 0.030989283 |
| INPP5B | -2.928537275 | 1.018179815 | 0.004024339 | 0.031004828 |
| ASB8 | -2.932928055 | 1.019903035 | 0.004031416 | 0.031015942 |
| IFFO1 | -2.955870137 | 1.027888605 | 0.004031688 | 0.031015942 |
| TBX4 | -2.95850454 | 1.029168569 | 0.004044692 | 0.031079928 |
| KCTD15 | -2.964673661 | 1.031349139 | 0.004045925 | 0.031079928 |
| MCF2L | -2.957061186 | 1.028913252 | 0.004053532 | 0.031115602 |
| SNED1 | -2.955760145 | 1.02879459 | 0.004065525 | 0.031163852 |
| PIH1D1 | -2.328144005 | 0.810348829 | 0.004065753 | 0.031163852 |
| BEST1 | 2.420209407 | 0.842550624 | 0.004072645 | 0.031193905 |
| ZZEF1 | -2.295770984 | 0.799910115 | 0.004104248 | 0.031413054 |
| PTRH1 | -2.916213093 | 1.016343791 | 0.004113586 | 0.031461589 |
| CPSF7 | -2.034712872 | 0.709456816 | 0.004130926 | 0.031571219 |
| XYLT1 | -2.928021495 | 1.021266545 | 0.004143185 | 0.03163743 |
| FLYWCH2 | -2.953176457 | 1.03010703 | 0.004145615 | 0.03163743 |
| STK24 | 1.760041719 | 0.614247015 | 0.004165292 | 0.031751087 |
| IL1A | 2.717456166 | 0.948412333 | 0.004166555 | 0.031751087 |
| SFRP4 | -2.927835388 | 1.021927726 | 0.004169932 | 0.031753775 |
| RBM6 | -2.307851625 | 0.805648734 | 0.004175522 | 0.031773304 |
| FTH1 | 1.69008456 | 0.590263375 | 0.004192905 | 0.031882476 |
| ANGEL1 | -2.711233534 | 0.947025048 | 0.004197893 | 0.031897303 |
| CEP68 | -2.926062444 | 1.022270564 | 0.00420556 | 0.031899135 |
| TRNAU1AP | -2.602384986 | 0.909195146 | 0.004205849 | 0.031899135 |
| ANKRD10 | -2.165302372 | 0.756519459 | 0.004207247 | 0.031899135 |
| DDHD2 | -2.601667748 | 0.909587096 | 0.00423276 | 0.0320581 |
| ADAMTS9 | -1.628540783 | 0.569388685 | 0.004234319 | 0.0320581 |
| VPS16 | -2.605084845 | 0.911102319 | 0.004246211 | 0.032124976 |
| DDX23 | -1.89991545 | 0.66484425 | 0.004267438 | 0.032262321 |
| VWA7 | 3.010279614 | 1.053527555 | 0.004272167 | 0.032274835 |
| GDPD5 | -2.905550538 | 1.017218158 | 0.004285166 | 0.032349771 |
| DCUN1D2 | -2.90822527 | 1.01831269 | 0.004291157 | 0.032371723 |
| DEAF1 | -2.738759334 | 0.959136133 | 0.004297673 | 0.032383949 |
| PPARA | -2.923663875 | 1.023924946 | 0.004298945 | 0.032383949 |
| MFGE8 | -2.723154638 | 0.95409004 | 0.004314666 | 0.032479076 |
| CALHM2 | -2.943832452 | 1.031556424 | 0.004320279 | 0.032488967 |
| MAP3K13 | 1.960073003 | 0.686868007 | 0.004322168 | 0.032488967 |
| STXBP6 | -2.703180964 | 0.94748974 | 0.004330967 | 0.032531817 |
| TMEM176B | 1.883714808 | 0.660366749 | 0.004337296 | 0.032546392 |
| LOC150776 | -2.707144959 | 0.94907765 | 0.004339106 | 0.032546392 |
| ABR | -2.215475194 | 0.777067963 | 0.004357237 | 0.0326478 |
| PRSS16 | 2.438894437 | 0.855522325 | 0.004361398 | 0.0326478 |
| ARHGAP44 | -2.912194319 | 1.021562301 | 0.004361953 | 0.0326478 |
| TUBGCP3 | -2.901876824 | 1.018405208 | 0.004379731 | 0.032744988 |
| RABL6 | -2.749404613 | 0.964931023 | 0.004381174 | 0.032744988 |
| RANBP3 | -2.379684292 | 0.835411391 | 0.004392338 | 0.032805082 |
| ZNF542 | -2.900719369 | 1.018590507 | 0.004402566 | 0.032835572 |
| TMEM160 | -2.933902662 | 1.030245692 | 0.004402675 | 0.032835572 |
| RIC8B | -2.896122528 | 1.01728784 | 0.004414644 | 0.032884897 |
| SLC9A8 | 1.741044834 | 0.6115871 | 0.004416593 | 0.032884897 |
| HSPA12B | -2.943937762 | 1.034189111 | 0.004418683 | 0.032884897 |
| LENG1 | -2.893501979 | 1.017409621 | 0.004455256 | 0.033106695 |
| DENND6A | -2.908075745 | 1.022543037 | 0.004455606 | 0.033106695 |
| PPFIBP2 | -2.902975293 | 1.020809595 | 0.004457944 | 0.033106695 |
| BCL2L1 | -2.238414738 | 0.787380449 | 0.004471029 | 0.033137484 |
| AKR1B10 | 2.994996491 | 1.053527598 | 0.004471536 | 0.033137484 |
| BCAT2 | -2.750591961 | 0.967555728 | 0.004471556 | 0.033137484 |
| ANGPTL2 | -2.549030932 | 0.897176242 | 0.004494823 | 0.033277224 |
| ZC3H11A | -2.253309307 | 0.793212589 | 0.004500924 | 0.033277224 |
| GPATCH2 | 2.479718744 | 0.872949938 | 0.004502598 | 0.033277224 |
| ST6GALNAC2 | -2.909608444 | 1.024343715 | 0.004504837 | 0.033277224 |
| EP400 | -2.428500279 | 0.855092084 | 0.004510712 | 0.033277224 |
| TP73-AS1 | -2.914815152 | 1.026395204 | 0.004513382 | 0.033277224 |
| PALM | -2.928210018 | 1.031171488 | 0.004515703 | 0.033277224 |
| WDR75 | -2.445158921 | 0.86106598 | 0.004515765 | 0.033277224 |
| AMOTL1 | -2.370733644 | 0.835241784 | 0.004534315 | 0.033390495 |
| EPHA4 | -2.898513016 | 1.021508506 | 0.004547077 | 0.033461005 |
| R3HDM4 | 1.648458256 | 0.581018986 | 0.004551326 | 0.033468816 |
| FAM118B | -2.886075446 | 1.017706916 | 0.004570232 | 0.033584328 |
| LRRC57 | -2.886405159 | 1.017959261 | 0.004575659 | 0.033600699 |
| FAM129B | -1.824599258 | 0.643648154 | 0.004585762 | 0.033651355 |
| FES | -2.901956175 | 1.023801388 | 0.00458987 | 0.033657979 |
| FENDRR | -2.954133442 | 1.042426441 | 0.004598356 | 0.03368842 |
| DTX2 | -2.900466977 | 1.023541367 | 0.004600438 | 0.03368842 |
| PRR3 | -2.889454192 | 1.019759912 | 0.004604633 | 0.033695642 |
| ACADVL | -2.050297299 | 0.723788448 | 0.004615234 | 0.033727478 |
| TTC33 | -2.884027059 | 1.018112975 | 0.004615407 | 0.033727478 |
| EPB41L4A-AS1 | -2.272758224 | 0.802555301 | 0.004627198 | 0.03376943 |
| PPM1F | -2.286153099 | 0.807334014 | 0.004629672 | 0.03376943 |
| CCDC114 | 2.70015565 | 0.9536119 | 0.004632969 | 0.03376943 |
| MBTD1 | -2.883776659 | 1.018487051 | 0.004634011 | 0.03376943 |
| NOP16 | -2.048969157 | 0.723745863 | 0.004639378 | 0.033785094 |
| SGK1 | -2.348409711 | 0.829680847 | 0.004647565 | 0.033792203 |
| C5orf4 | -2.93451715 | 1.036825456 | 0.004650577 | 0.033792203 |
| GNG11 | -2.022647015 | 0.714681964 | 0.004652825 | 0.033792203 |
| ENGASE | -2.687771875 | 0.949706388 | 0.004653226 | 0.033792203 |
| VWF | -2.91439886 | 1.029936739 | 0.004659353 | 0.033813311 |
| HLA-DRB6 | 1.683561805 | 0.595066579 | 0.004666466 | 0.03384155 |
| SAA1 | 2.661120602 | 0.940983134 | 0.004683665 | 0.033942834 |
| TARBP2 | -2.893974271 | 1.023416742 | 0.004687531 | 0.033947426 |
| ID3 | -2.339077395 | 0.827299124 | 0.004693266 | 0.033965529 |
| TARSL2 | -2.879488568 | 1.018532461 | 0.004697231 | 0.033970817 |
| OXCT1 | -2.884111624 | 1.020432606 | 0.004708007 | 0.034025315 |
| MBD2 | 1.714643967 | 0.606730605 | 0.004712762 | 0.034036254 |
| FCGR2C | 2.633310139 | 0.93194806 | 0.004719252 | 0.0340597 |
| USMG5 | 1.584222906 | 0.560725715 | 0.004723517 | 0.03406707 |
| SARS | -2.038691847 | 0.721649808 | 0.004727425 | 0.034071852 |
| KDM4C | -2.684809257 | 0.950794058 | 0.004746469 | 0.034185643 |
| NCKIPSD | -2.77685946 | 0.983825595 | 0.004764905 | 0.034294907 |
| SMG6 | -2.447590653 | 0.867543716 | 0.004783124 | 0.034402459 |
| INMT | -2.910954848 | 1.031940885 | 0.004789596 | 0.034425423 |
| MNAT1 | -2.87201565 | 1.018443525 | 0.004802293 | 0.034493076 |
| TSEN54 | -2.887792243 | 1.024214825 | 0.004809582 | 0.034521817 |
| UNC45A | -2.299553909 | 0.815670909 | 0.004814085 | 0.034527399 |
| CDK4 | 1.670948597 | 0.592739324 | 0.004816936 | 0.034527399 |
| ARHGEF5 | -2.873946479 | 1.019561369 | 0.004820253 | 0.034527608 |
| IFIH1 | 2.457927618 | 0.872838609 | 0.004862322 | 0.034802194 |
| SNRNP35 | -2.887758763 | 1.02568312 | 0.004870909 | 0.034802194 |
| GPR146 | -2.942178276 | 1.045026854 | 0.004871515 | 0.034802194 |
| PRMT10 | -2.867398454 | 1.018483183 | 0.004872238 | 0.034802194 |
| TBC1D17 | -2.869459805 | 1.01928506 | 0.004875157 | 0.034802194 |
| KIAA0556 | -2.86722475 | 1.018594658 | 0.0048795 | 0.034809531 |
| FAM20C | -2.762828595 | 0.981803665 | 0.00489241 | 0.034877934 |
| AQP1 | -2.703384614 | 0.961046205 | 0.004908774 | 0.034970855 |
| NFKBIL1 | -2.88156969 | 1.024769298 | 0.004924669 | 0.035060304 |
| THAP5 | -2.866793945 | 1.019687422 | 0.00493197 | 0.035088491 |
| MMAB | -2.889828542 | 1.028114818 | 0.004941798 | 0.035134615 |
| GMDS | -2.863945734 | 1.019260301 | 0.004956804 | 0.035168543 |
| CLIC3 | -2.866678443 | 1.020239581 | 0.00495709 | 0.035168543 |
| FLJ34208 | 2.630433913 | 0.936172217 | 0.004957604 | 0.035168543 |
| CALR | 1.539994529 | 0.54811443 | 0.004959967 | 0.035168543 |
| LRRC1 | -2.883050996 | 1.026416811 | 0.004971879 | 0.035229215 |
| DTX3 | -2.904115826 | 1.034075463 | 0.004978561 | 0.035252775 |
| ZCCHC14 | -2.456004718 | 0.874624038 | 0.004983953 | 0.035254141 |
| ENG | -2.609104702 | 0.929177861 | 0.004985468 | 0.035254141 |
| DOK4 | -2.876414281 | 1.024761205 | 0.005001894 | 0.035346494 |
| PAMR1 | -2.864836185 | 1.020757247 | 0.005007058 | 0.035359192 |
| ZSCAN29 | -2.859383191 | 1.019032488 | 0.005016405 | 0.035363987 |
| RABGAP1L | 2.078467242 | 0.740748343 | 0.005017595 | 0.035363987 |
| FRA10AC1 | -2.865244343 | 1.021154848 | 0.00501784 | 0.035363987 |
| MROH8 | 2.600215525 | 0.926980533 | 0.005031066 | 0.035433419 |
| FAM212B | -2.890480608 | 1.030542764 | 0.005034565 | 0.035434293 |
| C1D | -2.858261051 | 1.019280471 | 0.005044241 | 0.035471253 |
| MCCC2 | 1.73647743 | 0.619275679 | 0.005046572 | 0.035471253 |
| DDX26B | -2.859725881 | 1.020291052 | 0.005065274 | 0.035578894 |
| GPM6B | -2.872155232 | 1.024967011 | 0.005075652 | 0.035627957 |
| ID2 | -2.208834229 | 0.788387841 | 0.005083252 | 0.035657467 |
| RNF216 | -2.44878107 | 0.874125266 | 0.005088023 | 0.035667106 |
| C22orf29 | -2.856401857 | 1.019757785 | 0.005093518 | 0.035681813 |
| ZRSR2 | -2.85688282 | 1.020135358 | 0.005102448 | 0.035720535 |
| RASL12 | -2.917902243 | 1.04219773 | 0.005114081 | 0.035778122 |
| ATXN7L3 | -2.363857112 | 0.844528703 | 0.005125714 | 0.035814984 |
| SIRT1 | -2.576350244 | 0.920455004 | 0.005126171 | 0.035814984 |
| CACNA1E | 2.947849778 | 1.053347654 | 0.005133206 | 0.035840292 |
| SOWAHC | -2.852543113 | 1.019820841 | 0.005156322 | 0.035977766 |
| RABGGTA | -2.698150354 | 0.96482404 | 0.005165611 | 0.036018646 |
| DOCK9 | -2.206309176 | 0.789091516 | 0.005173751 | 0.036051469 |
| ZNF814 | -2.854194088 | 1.020928283 | 0.005178982 | 0.036051556 |
| DAZAP2 | 1.611093994 | 0.57629996 | 0.00518063 | 0.036051556 |
| CYHR1 | -2.872494828 | 1.027898496 | 0.005197496 | 0.036144971 |
| SLC35G2 | -2.853344146 | 1.021297716 | 0.005208597 | 0.036198197 |
| BEND7 | -2.854397068 | 1.021789177 | 0.005213645 | 0.036209319 |
| TRRAP | -2.392609086 | 0.856642713 | 0.005222056 | 0.036243763 |
| FBXO18 | -2.017730232 | 0.722486528 | 0.005226073 | 0.036247682 |
| SH3RF1 | -2.539984447 | 0.909679919 | 0.005235522 | 0.036289255 |
| RGS4 | 2.53190645 | 0.907011246 | 0.005246712 | 0.036342824 |
| HOOK2 | -2.850619094 | 1.021288148 | 0.005251307 | 0.036350678 |
| LOC100506548 | -2.354979008 | 0.843823298 | 0.005257084 | 0.036366691 |
| ZCCHC24 | -2.829285564 | 1.013953245 | 0.005265091 | 0.036389636 |
| LAT | -2.850125003 | 1.021472057 | 0.005267331 | 0.036389636 |
| SOCS7 | -2.843433444 | 1.019601665 | 0.00529088 | 0.036528291 |
| LOC643733 | -2.84346882 | 1.019909379 | 0.005304073 | 0.036595315 |
| RAB34 | -2.072332142 | 0.74350036 | 0.005315508 | 0.036650129 |
| VPRBP | -2.860613272 | 1.026518598 | 0.005324552 | 0.036688403 |
| LARGE | -2.842842723 | 1.0202595 | 0.00532984 | 0.036700757 |
| CCAR1 | -2.300605577 | 0.825789482 | 0.005337163 | 0.036726198 |
| SHC2 | -2.740770669 | 0.983856477 | 0.00534053 | 0.036726198 |
| DUSP1 | -2.280702643 | 0.81880697 | 0.005346219 | 0.03674126 |
| UBE2O | -2.688494766 | 0.965365788 | 0.005353609 | 0.036751903 |
| TSSC1 | -2.845424621 | 1.021839913 | 0.00535923 | 0.036751903 |
| GSS | -2.417955228 | 0.868363813 | 0.005361105 | 0.036751903 |
| AP1S2 | 1.739302656 | 0.624647305 | 0.005361767 | 0.036751903 |
| RECK | -2.847838261 | 1.023421495 | 0.005391459 | 0.036931319 |
| FAM13A | -2.839597472 | 1.021081746 | 0.005419678 | 0.037100418 |
| HABP4 | -2.720898267 | 0.978585181 | 0.005428514 | 0.037136693 |
| POLE | -2.840324835 | 1.022134609 | 0.005455728 | 0.037285291 |
| DLG4 | -2.838737941 | 1.021598744 | 0.005457337 | 0.037285291 |
| ZNF395 | -2.075482012 | 0.747185382 | 0.005473946 | 0.037374453 |
| LOC730227 | 2.903695674 | 1.045448685 | 0.005478498 | 0.037381223 |
| ZNF497 | 2.923136426 | 1.052591444 | 0.005484875 | 0.037385392 |
| ABCF1 | -2.086622389 | 0.751393022 | 0.005486229 | 0.037385392 |
| SCAMP4 | -2.834763567 | 1.02093417 | 0.005492449 | 0.037403504 |
| SDHAF1 | -2.839982538 | 1.022972546 | 0.005499735 | 0.037428849 |
| TMOD1 | -2.504165189 | 0.902126453 | 0.005505808 | 0.037445911 |
| GNL3 | -2.359626447 | 0.850243914 | 0.005516188 | 0.037492225 |
| OSBPL10 | -2.839198032 | 1.02314801 | 0.005520796 | 0.037499276 |
| HTATSF1 | -2.433600522 | 0.877292503 | 0.00553734 | 0.037587336 |
| CLIP4 | -2.642174524 | 0.952559065 | 0.005541177 | 0.037589085 |
| DHX9 | -2.075218231 | 0.748755147 | 0.005578875 | 0.03782038 |
| EIF3B | -2.279917183 | 0.822824669 | 0.005591154 | 0.037859164 |
| IQGAP1 | 1.671929156 | 0.603409383 | 0.005591807 | 0.037859164 |
| TRIM5 | 2.111199299 | 0.762075427 | 0.005599978 | 0.037890055 |
| MRS2 | -2.82717119 | 1.020827922 | 0.005614439 | 0.037953641 |
| SPARCL1 | -2.683389587 | 0.968955568 | 0.005616604 | 0.037953641 |
| METTL14 | -2.826354404 | 1.020742497 | 0.005624243 | 0.037980819 |
| LY6G5C | 2.531383204 | 0.914733869 | 0.00565151 | 0.038126246 |
| OAF | -2.568301296 | 0.928104069 | 0.005653039 | 0.038126246 |
| LBH | -2.240677751 | 0.809941002 | 0.005666674 | 0.038193675 |
| SEC31B | -2.824172534 | 1.020959113 | 0.005671452 | 0.038201357 |
| CDC14A | -2.830910144 | 1.023591565 | 0.005680707 | 0.038239167 |
| MTMR9LP | -2.869181367 | 1.037715273 | 0.005693984 | 0.038303991 |
| KATNB1 | -2.824571654 | 1.021879906 | 0.005708117 | 0.038322539 |
| ACTR5 | -2.822038986 | 1.020984264 | 0.005709094 | 0.038322539 |
| C12orf44 | -2.1517766 | 0.778515096 | 0.005710633 | 0.038322539 |
| DISP1 | -2.839908795 | 1.02749706 | 0.005711339 | 0.038322539 |
| EHD4 | -1.788381894 | 0.647179213 | 0.005721159 | 0.038362429 |
| LRSAM1 | -2.823184354 | 1.021725631 | 0.005724591 | 0.038362429 |
| BHLHE40 | -1.696660444 | 0.614124365 | 0.005731998 | 0.03838757 |
| LMO2 | 2.141691447 | 0.77543322 | 0.005746098 | 0.038457476 |
| PAIP1 | -2.246027106 | 0.813366524 | 0.005755484 | 0.038495753 |
| CYB5D1 | -2.404898782 | 0.871379024 | 0.005782316 | 0.038650603 |
| UQCRC1 | -2.242352949 | 0.812862538 | 0.005805165 | 0.03875592 |
| CNTROB | -2.397563014 | 0.869131961 | 0.005805453 | 0.03875592 |
| MAP4K5 | -2.39707588 | 0.869049896 | 0.005810785 | 0.038761696 |
| RHBDD2 | -2.307747538 | 0.836784654 | 0.005817843 | 0.038761696 |
| POFUT2 | -2.280833892 | 0.827026758 | 0.005817898 | 0.038761696 |
| FLJ45513 | 2.603647096 | 0.944139561 | 0.005821083 | 0.038761696 |
| EPAS1 | -2.244623742 | 0.814082008 | 0.005829062 | 0.038790225 |
| DKK3 | -2.276567047 | 0.825755657 | 0.005834327 | 0.038795569 |
| LY86 | 2.258438989 | 0.819228973 | 0.005837253 | 0.038795569 |
| ZNF282 | -2.816539939 | 1.022135457 | 0.00585945 | 0.038918458 |
| CRELD2 | -2.349927538 | 0.85288251 | 0.005864247 | 0.0389257 |
| CYBASC3 | -2.696274679 | 0.978747559 | 0.005872419 | 0.03895532 |
| STRIP1 | -2.407652843 | 0.874281857 | 0.005889634 | 0.039044851 |
| SRBD1 | 2.149134992 | 0.780837519 | 0.005916998 | 0.039201509 |
| THEM4 | -2.825865273 | 1.02696126 | 0.005929104 | 0.039242393 |
| TBCEL | -2.811465714 | 1.021765533 | 0.005930922 | 0.039242393 |
| ZKSCAN5 | -2.809663394 | 1.021181359 | 0.00593438 | 0.039242393 |
| AXIN2 | -2.816946754 | 1.023915551 | 0.005938618 | 0.039245706 |
| CSRNP1 | -1.835165939 | 0.667308539 | 0.005957688 | 0.039346971 |
| ZMYM6 | -2.80865277 | 1.021386139 | 0.00596236 | 0.039353077 |
| TCF7L1 | -2.823283337 | 1.02791307 | 0.006021344 | 0.039677205 |
| NPR2 | -2.810380991 | 1.023215625 | 0.006021348 | 0.039677205 |
| NAMPT | 1.907233745 | 0.69441393 | 0.006022804 | 0.039677205 |
| CENPC1 | -2.805253051 | 1.021612992 | 0.006034409 | 0.03972873 |
| MXI1 | -2.324973494 | 0.846849652 | 0.006043012 | 0.039760447 |
| ZSWIM7 | -2.816880009 | 1.026184912 | 0.006051046 | 0.039788376 |
| PHLPP2 | -2.828035582 | 1.030667458 | 0.006071634 | 0.039891336 |
| DNAJC11 | -2.803523001 | 1.021787651 | 0.006074302 | 0.039891336 |
| TWF2 | -2.282334397 | 0.831939573 | 0.006080849 | 0.03990937 |
| BBS4 | -2.802518233 | 1.02175388 | 0.006090839 | 0.039946797 |
| WDR13 | -2.498643051 | 0.911025232 | 0.006094159 | 0.039946797 |
| MSANTD4 | -2.801680683 | 1.02162184 | 0.006099475 | 0.039956701 |
| AFF4 | -2.055281254 | 0.749612412 | 0.006110504 | 0.04000399 |
| ZC3HC1 | -2.800584872 | 1.021575624 | 0.006117115 | 0.040017451 |
| B3GALNT2 | -2.618746301 | 0.955303332 | 0.006120181 | 0.040017451 |
| MAFK | -2.38274687 | 0.869305289 | 0.006125657 | 0.040028326 |
| ZNF155 | -2.799640074 | 1.021706661 | 0.006140922 | 0.040103122 |
| RNF40 | -2.088914018 | 0.76257899 | 0.006157473 | 0.040167694 |
| EXOSC8 | -2.483802317 | 0.906754354 | 0.00615846 | 0.040167694 |
| HELZ2 | 1.843125016 | 0.673054587 | 0.006172991 | 0.040237482 |
| POMT2 | -2.610092691 | 0.953397265 | 0.006187503 | 0.04030705 |
| ZCCHC9 | -2.796837193 | 1.021801713 | 0.00619717 | 0.040344997 |
| SENP3 | -2.803875911 | 1.024492527 | 0.006203176 | 0.040349905 |
| SPAG6 | 2.531288824 | 0.924937051 | 0.006205609 | 0.040349905 |
| PRICKLE2 | -2.223592177 | 0.813067789 | 0.006241483 | 0.040558055 |
| ANXA3 | 1.454291482 | 0.532557713 | 0.006318696 | 0.041034403 |
| CFB | 1.940165998 | 0.710641379 | 0.006330323 | 0.041084503 |
| MFHAS1 | -2.617130804 | 0.958845948 | 0.006343832 | 0.041146743 |
| HLA-C | 1.586795219 | 0.581712855 | 0.006375862 | 0.041328967 |
| PTPN13 | -2.797313144 | 1.025694375 | 0.006386687 | 0.041373594 |
| ABT1 | -2.105772103 | 0.772200745 | 0.006391789 | 0.041381118 |
| ZNF25 | -2.794500298 | 1.024941555 | 0.006401049 | 0.041415538 |
| ZMYND19 | -2.787454078 | 1.022541938 | 0.00641061 | 0.041433554 |
| GJC1 | -2.62636999 | 0.963470598 | 0.006411725 | 0.041433554 |
| SF3B5 | -2.143226401 | 0.786335076 | 0.006418683 | 0.041453006 |
| SLK | -2.211805366 | 0.811782597 | 0.006437403 | 0.041497805 |
| CREB3L2 | -2.325273258 | 0.853595198 | 0.006447822 | 0.041497805 |
| FAM73B | -2.793798977 | 1.025634965 | 0.006450235 | 0.041497805 |
| ASXL1 | -2.07901135 | 0.763232155 | 0.006450496 | 0.041497805 |
| PRDM15 | -2.786892942 | 1.023131519 | 0.00645189 | 0.041497805 |
| RCAN1 | -2.057630855 | 0.755416266 | 0.006452832 | 0.041497805 |
| COG7 | -2.793712382 | 1.025726745 | 0.006456646 | 0.041497805 |
| RCOR1 | -2.407935114 | 0.884096271 | 0.006457234 | 0.041497805 |
| DENND2A | -2.84024965 | 1.043129271 | 0.006472799 | 0.041568971 |
| DOK2 | -2.81545652 | 1.034089976 | 0.006476225 | 0.041568971 |
| NTNG2 | 2.486725906 | 0.913723541 | 0.006498051 | 0.041667038 |
| MGC39372 | 2.460326993 | 0.904046958 | 0.006499439 | 0.041667038 |
| TOM1L2 | -2.450918514 | 0.900782615 | 0.006510902 | 0.041713513 |
| MOB3C | -2.171116144 | 0.798002786 | 0.006514633 | 0.041713513 |
| ZNF585B | -2.780831388 | 1.022727142 | 0.006547258 | 0.041896868 |
| TDRD7 | 1.958475626 | 0.720345352 | 0.006551902 | 0.041901051 |
| SPRYD7 | -2.78102002 | 1.02310988 | 0.006563762 | 0.041936114 |
| ZNF197 | -2.779456862 | 1.022639121 | 0.006569264 | 0.041936114 |
| PHLPP1 | -2.77985428 | 1.022787261 | 0.006569366 | 0.041936114 |
| CBY1 | -2.806512993 | 1.032737335 | 0.006576767 | 0.041957856 |
| LAMA2 | -2.619626844 | 0.964640007 | 0.006614535 | 0.042173181 |
| CCDC124 | -2.007580164 | 0.73935303 | 0.006621173 | 0.042176382 |
| KIAA1967 | -1.866103103 | 0.68727376 | 0.00662307 | 0.042176382 |
| ZFYVE19 | -2.781805957 | 1.024837834 | 0.006639865 | 0.042219435 |
| C17orf70 | -2.781040363 | 1.024580536 | 0.00664118 | 0.042219435 |
| HCLS1 | 1.768884287 | 0.651694288 | 0.006641892 | 0.042219435 |
| CCDC57 | -2.784962313 | 1.026268659 | 0.006654088 | 0.042271373 |
| ZDHHC14 | -2.782475532 | 1.025844543 | 0.006680288 | 0.042412158 |
| SIGLEC14 | 2.853612951 | 1.05219307 | 0.006686606 | 0.042426619 |
| AP4M1 | -2.773266366 | 1.022827895 | 0.006700551 | 0.042489427 |
| JUNB | -1.712264049 | 0.631945377 | 0.006738212 | 0.042617094 |
| PKLR | 2.821643696 | 1.041448978 | 0.006741637 | 0.042617094 |
| GAB4 | 2.821643696 | 1.041448978 | 0.006741637 | 0.042617094 |
| SLC25A23 | -2.80863123 | 1.036647307 | 0.006741697 | 0.042617094 |
| SLC15A3 | -2.222953139 | 0.82048902 | 0.006742479 | 0.042617094 |
| AKAP11 | -2.593728948 | 0.957386305 | 0.006745035 | 0.042617094 |
| DUSP10 | -2.800715485 | 1.033970211 | 0.006754731 | 0.042652696 |
| SEPT9 | -2.205876857 | 0.814462239 | 0.006761165 | 0.042666768 |
| UGGT2 | -2.59191409 | 0.957064356 | 0.006765086 | 0.042666768 |
| GLT25D1 | -1.907104443 | 0.704339001 | 0.006776117 | 0.042689135 |
| OAZ2 | -2.409329824 | 0.889833175 | 0.006776763 | 0.042689135 |
| TOP3A | -2.769238587 | 1.023143282 | 0.006797631 | 0.042793962 |
| FPR2 | 2.476722034 | 0.915185287 | 0.006804738 | 0.042793962 |
| SLC5A7 | 2.838355527 | 1.048903426 | 0.006809458 | 0.042793962 |
| LOC93622 | -2.781513981 | 1.027902428 | 0.006809705 | 0.042793962 |
| CIR1 | 1.850440327 | 0.684030259 | 0.006826277 | 0.042872452 |
| ABCC2 | 2.58392807 | 0.955402822 | 0.00683984 | 0.042907836 |
| PCBP4 | -2.803448111 | 1.036574493 | 0.006840084 | 0.042907836 |
| LTBP3 | -2.653684544 | 0.981310773 | 0.006846404 | 0.042921841 |
| ATG9A | -2.207891106 | 0.816713063 | 0.006863688 | 0.043004524 |
| ARL6IP4 | -2.268744511 | 0.839359759 | 0.006872781 | 0.043016718 |
| GABARAP | 1.689154924 | 0.624988868 | 0.006877962 | 0.043016718 |
| TOMM34 | -2.540671394 | 0.94017996 | 0.006885651 | 0.043016718 |
| EIF3A | -1.864087734 | 0.689832916 | 0.006887594 | 0.043016718 |
| PDE4DIP | -2.498658789 | 0.924691388 | 0.006889179 | 0.043016718 |
| RBM33 | -2.159730795 | 0.799329331 | 0.006893856 | 0.043016718 |
| TBC1D16 | -2.28596314 | 0.846055517 | 0.006894309 | 0.043016718 |
| LAS1L | -2.202787837 | 0.815506355 | 0.006910454 | 0.043065182 |
| ARG2 | -2.789702579 | 1.032836936 | 0.006912932 | 0.043065182 |
| ZNF407 | -2.764444059 | 1.023511802 | 0.006914379 | 0.043065182 |
| TRIP10 | -1.878628451 | 0.695702106 | 0.006926978 | 0.043098835 |
| KCNJ8 | -2.766596164 | 1.024556754 | 0.006927991 | 0.043098835 |
| MON2 | -2.321356756 | 0.860086721 | 0.006955238 | 0.043242721 |
| SLC35A3 | -2.769948058 | 1.026598031 | 0.006971937 | 0.043260886 |
| WRAP73 | -2.593583766 | 0.961338224 | 0.006978072 | 0.043260886 |
| PRRG1 | -2.762245937 | 1.023915252 | 0.006981423 | 0.043260886 |
| PRG2 | -2.815146665 | 1.043729055 | 0.006992513 | 0.043260886 |
| ZBTB46 | -2.76742809 | 1.026040997 | 0.006992724 | 0.043260886 |
| TRIP11 | -2.61113544 | 0.968095935 | 0.006992806 | 0.043260886 |
| A2M | -2.373301872 | 0.87991785 | 0.006992826 | 0.043260886 |
| ZFYVE27 | -2.232068739 | 0.827628134 | 0.006997853 | 0.043260886 |
| ARHGEF1 | -2.240590984 | 0.830798727 | 0.006998578 | 0.043260886 |
| LOC100129250 | 2.577918126 | 0.955890894 | 0.006999357 | 0.043260886 |
| SIAH2 | 1.787903605 | 0.66314514 | 0.007015724 | 0.043336539 |
| USP48 | -2.285387015 | 0.847814453 | 0.007025741 | 0.043347925 |
| LOC282997 | 2.439338163 | 0.904927311 | 0.007025823 | 0.043347925 |
| SLC43A1 | -2.763004291 | 1.025593175 | 0.007058854 | 0.043513085 |
| MAST3 | -2.764024775 | 1.02601431 | 0.007061209 | 0.043513085 |
| C20orf194 | -2.457084943 | 0.912138263 | 0.007065023 | 0.043513085 |
| IL15 | -2.765118068 | 1.026652358 | 0.007074129 | 0.043543626 |
| DENND3 | -2.107664875 | 0.782922221 | 0.007101454 | 0.043686215 |
| SMARCB1 | -2.436292291 | 0.905180098 | 0.007113148 | 0.043708338 |
| MAML1 | -2.257177911 | 0.838732851 | 0.007120057 | 0.043708338 |
| RPS3 | -1.379291976 | 0.51255489 | 0.007123538 | 0.043708338 |
| CDK11B | -2.58028145 | 0.958876803 | 0.007125067 | 0.043708338 |
| CD97 | -2.235267895 | 0.830675336 | 0.007125862 | 0.043708338 |
| CCDC38 | 2.833324997 | 1.05349472 | 0.007156913 | 0.043873172 |
| HIST2H2AC | 2.598100258 | 0.966154257 | 0.007164166 | 0.043885929 |
| CCZ1B | -2.753000364 | 1.023813406 | 0.007167352 | 0.043885929 |
| PAWR | -2.76536354 | 1.028619235 | 0.007179041 | 0.043931879 |
| PIAS3 | -2.27750155 | 0.847309254 | 0.00718981 | 0.043948585 |
| PISD | -2.255901554 | 0.839278111 | 0.007190141 | 0.043948585 |
| WNT7B | 2.713202257 | 1.009525023 | 0.007196695 | 0.043963057 |
| DAB2IP | -2.370894353 | 0.882247514 | 0.007202474 | 0.043972781 |
| IPPK | -2.75564682 | 1.025644664 | 0.007215177 | 0.043987914 |
| RTN3 | 1.867370989 | 0.69506399 | 0.007217958 | 0.043987914 |
| DDX55 | -2.433902851 | 0.906006757 | 0.007222506 | 0.043987914 |
| FEM1C | 1.78924703 | 0.666065765 | 0.007224995 | 0.043987914 |
| FAM101B | -2.142096374 | 0.797429981 | 0.007225898 | 0.043987914 |
| S100PBP | -2.562914636 | 0.954628634 | 0.007258967 | 0.044163623 |
| GTPBP2 | 1.787326006 | 0.66583414 | 0.00726729 | 0.044188659 |
| ENC1 | -2.750992836 | 1.025417218 | 0.007300789 | 0.044305819 |
| ZNF589 | -2.759318878 | 1.028522338 | 0.007300882 | 0.044305819 |
| IMP4 | -1.925157742 | 0.717615421 | 0.007302717 | 0.044305819 |
| DVL1 | -2.5633831 | 0.955529909 | 0.007303435 | 0.044305819 |
| CEP85L | -2.748254246 | 1.024801183 | 0.007323952 | 0.044404631 |
| ORAI1 | -2.42801842 | 0.90556784 | 0.007335625 | 0.044449742 |
| GLTSCR2 | -2.357730287 | 0.879572728 | 0.007350338 | 0.044497971 |
| USP31 | -2.543490641 | 0.94890007 | 0.00735206 | 0.044497971 |
| INTS1 | -2.58102471 | 0.963011019 | 0.007358673 | 0.04451234 |
| ZNF761 | -2.757532032 | 1.029054901 | 0.007369381 | 0.044551452 |
| AJUBA | -2.741851091 | 1.024363195 | 0.007436458 | 0.044931096 |
| ARHGEF25 | -2.787876555 | 1.041670302 | 0.00744284 | 0.044943794 |
| FCGR1B | 2.693219699 | 1.006396929 | 0.007448422 | 0.044951657 |
| CKS1B | -2.740761362 | 1.024505965 | 0.007468403 | 0.045026419 |
| TRMT12 | -2.741616683 | 1.024842592 | 0.007469386 | 0.045026419 |
| DNAJC17 | -2.741565079 | 1.025116216 | 0.007486435 | 0.0451033 |
| ZNF419 | -2.739037457 | 1.024386311 | 0.007498989 | 0.045129718 |
| CGRRF1 | -2.744634364 | 1.026486848 | 0.007499415 | 0.045129718 |
| IL27RA | -2.757475715 | 1.031383737 | 0.007504881 | 0.045132286 |
| CFLAR | 1.556893556 | 0.582364419 | 0.007508646 | 0.045132286 |
| OTX1 | 2.81494935 | 1.053018904 | 0.007512736 | 0.045132286 |
| MMP24 | -2.740337763 | 1.026003533 | 0.007565149 | 0.045411233 |
| PRKAG2 | -2.24220698 | 0.839536757 | 0.007567818 | 0.045411233 |
| ARMCX4 | -2.749567547 | 1.029639762 | 0.007575709 | 0.045429305 |
| LPCAT4 | -2.75337925 | 1.031131688 | 0.007579482 | 0.045429305 |
| HIRIP3 | -2.754686671 | 1.03174629 | 0.007586787 | 0.045447149 |
| C8orf33 | -2.095173656 | 0.784952108 | 0.007603808 | 0.045523138 |
| TPM4 | 1.601535251 | 0.600200619 | 0.007622861 | 0.04559182 |
| GBA2 | -2.14214413 | 0.802816937 | 0.007623963 | 0.04559182 |
| KCNK5 | -2.752690741 | 1.03190296 | 0.007639815 | 0.045660611 |
| HS1BP3 | -2.757262932 | 1.033826695 | 0.007652129 | 0.045684215 |
| EFHC1 | -2.739287202 | 1.027092437 | 0.007652465 | 0.045684215 |
| PLCE1 | -2.768240793 | 1.038045983 | 0.007658167 | 0.045692279 |
| CSTB | 1.501114124 | 0.563051909 | 0.00767525 | 0.045752106 |
| BRD3 | -2.737511294 | 1.026972779 | 0.007684826 | 0.045752106 |
| RAB15 | -2.731230294 | 1.024732516 | 0.007691728 | 0.045752106 |
| KIAA0895L | -2.759298535 | 1.035264432 | 0.007691785 | 0.045752106 |
| TRPA1 | 2.614947686 | 0.981276588 | 0.007702436 | 0.045752106 |
| ZBTB24 | -2.730742249 | 1.024755805 | 0.007704016 | 0.045752106 |
| PLA2G15 | -2.730733757 | 1.024761619 | 0.007704552 | 0.045752106 |
| SYT11 | -2.634316896 | 0.988601947 | 0.007705951 | 0.045752106 |
| ZNF264 | -2.732076964 | 1.025313613 | 0.007707406 | 0.045752106 |
| ZNF841 | -2.752275765 | 1.033084622 | 0.007718683 | 0.045761163 |
| GUK1 | -2.039170101 | 0.765431358 | 0.007719928 | 0.045761163 |
| TMEM133 | -2.731975267 | 1.025520422 | 0.007722006 | 0.045761163 |
| STARD5 | 2.01826169 | 0.7577646 | 0.007734586 | 0.045809862 |
| ZNF805 | -2.557328252 | 0.960300086 | 0.007743564 | 0.045837186 |
| C21orf2 | -2.747749379 | 1.031974672 | 0.007753649 | 0.045871022 |
| UBC | -1.77099919 | 0.665267088 | 0.007765769 | 0.045916859 |
| FAM109B | -2.732038813 | 1.026543423 | 0.007781732 | 0.045934604 |
| GTF2B | 1.905001726 | 0.715803585 | 0.00778287 | 0.045934604 |
| MNF1 | -2.320205046 | 0.871837499 | 0.007784384 | 0.045934604 |
| SEPT6 | 1.620428794 | 0.608925451 | 0.007787926 | 0.045934604 |
| SPECC1 | -2.549265284 | 0.958006303 | 0.007790642 | 0.045934604 |
| SBNO1 | -2.343541901 | 0.880969685 | 0.007809786 | 0.045995857 |
| ZNF862 | -2.73076164 | 1.026571395 | 0.007812218 | 0.045995857 |
| MLLT10 | -2.410758089 | 0.906329403 | 0.007816065 | 0.045995857 |
| KLHDC4 | -2.471967104 | 0.929378482 | 0.007818551 | 0.045995857 |
| TIMP3 | -2.190974016 | 0.823879066 | 0.007829406 | 0.046033926 |
| BCDIN3D-AS1 | 2.800146499 | 1.053205828 | 0.007844539 | 0.04605777 |
| ZNF766 | -2.727102141 | 1.025743662 | 0.007845243 | 0.04605777 |
| PRR24 | -2.742611295 | 1.031600047 | 0.007846619 | 0.04605777 |
| LOC645638 | 2.157095578 | 0.811479934 | 0.007855362 | 0.046083329 |
| PHRF1 | -2.408686757 | 0.906547145 | 0.007884177 | 0.046226543 |
| RBFA | -2.729337685 | 1.027557129 | 0.007904032 | 0.046309573 |
| RPL10 | -1.556206872 | 0.585919347 | 0.007907158 | 0.046309573 |
| RSF1 | -2.613945167 | 0.984482175 | 0.007927379 | 0.046402122 |
| TNIK | -2.728759966 | 1.027880279 | 0.00793684 | 0.046406027 |
| PAGR1 | -2.615857391 | 0.985352392 | 0.007936885 | 0.046406027 |
| SLPI | 1.762810147 | 0.664194647 | 0.00795305 | 0.046474667 |
| RPH3A | 2.792204489 | 1.052389525 | 0.007973158 | 0.046566258 |
| GDE1 | -2.198735538 | 0.828770036 | 0.007977741 | 0.046567125 |
| NDUFS2 | -1.662861283 | 0.626828124 | 0.007982277 | 0.046567715 |
| METTL22 | -2.71938192 | 1.025263058 | 0.007992776 | 0.046580687 |
| CASP7 | -2.132282728 | 0.803952507 | 0.007995741 | 0.046580687 |
| ANXA6 | -2.151231881 | 0.811123754 | 0.007997808 | 0.046580687 |
| MGC27345 | -2.718195151 | 1.025364545 | 0.008026456 | 0.046721628 |
| GIMAP4 | 1.648285576 | 0.621854592 | 0.008034978 | 0.046745321 |
| IKBKG | -2.717619855 | 1.025434054 | 0.008044076 | 0.046772339 |
| ZNF687 | -2.716204341 | 1.025402672 | 0.008075068 | 0.046926557 |
| LOR | 2.781311423 | 1.050243738 | 0.008090888 | 0.046992488 |
| STON2 | -2.716531729 | 1.026416287 | 0.008130116 | 0.04719422 |
| POM121C | -2.306313274 | 0.871799783 | 0.008157943 | 0.04732959 |
| TNFRSF25 | -2.711345555 | 1.025646472 | 0.008204216 | 0.047571769 |
| YWHAZ | 1.70148686 | 0.643684637 | 0.008208897 | 0.047572643 |
| CD68 | 1.363145908 | 0.515744763 | 0.008215974 | 0.047587395 |
| ACAT2 | -2.71016726 | 1.025696683 | 0.008235245 | 0.0476504 |
| SLC39A3 | -2.719113992 | 1.029129625 | 0.008238176 | 0.0476504 |
| MIF4GD | -2.735695042 | 1.035485035 | 0.008243132 | 0.0476504 |
| SGSH | -2.239069172 | 0.847532369 | 0.008245003 | 0.0476504 |
| RPL36AL | -1.877949324 | 0.71108967 | 0.008267493 | 0.047754093 |
| LSM4 | -2.281597568 | 0.864057925 | 0.008276892 | 0.0477821 |
| KLHL33 | 2.78130401 | 1.053529768 | 0.008290941 | 0.047791983 |
| GIMAP7 | -2.407136524 | 0.911813806 | 0.008291996 | 0.047791983 |
| VAPB | -2.0697676 | 0.784025941 | 0.008292503 | 0.047791983 |
| EEF2 | -1.535822839 | 0.581862617 | 0.008302991 | 0.047791983 |
| UBE2G2 | -1.758891249 | 0.666384539 | 0.008303972 | 0.047791983 |
| TIMM10B | -2.151053163 | 0.814985882 | 0.008305911 | 0.047791983 |
| NDUFAF5 | -2.707122814 | 1.025813564 | 0.008315079 | 0.047818532 |
| FGFR1 | -2.362887277 | 0.895458376 | 0.008321328 | 0.047828281 |
| C7orf50 | -2.596241203 | 0.98457096 | 0.008366097 | 0.048044522 |
| TRMT11 | -2.710766672 | 1.028034064 | 0.008368101 | 0.048044522 |
| MRPL37 | -2.081562637 | 0.789801051 | 0.008400027 | 0.048201468 |
| TMEM136 | -2.714733612 | 1.0304623 | 0.008426596 | 0.048306124 |
| SH3TC1 | -2.608049231 | 0.98998007 | 0.008427466 | 0.048306124 |
| POLR2G | -2.111370361 | 0.801558739 | 0.008436545 | 0.048317742 |
| ZNF440 | -2.703458878 | 1.026418539 | 0.008441638 | 0.048317742 |
| LRRK2 | 2.293343046 | 0.870751404 | 0.00844472 | 0.048317742 |
| LRR1 | -2.702488832 | 1.026170904 | 0.008449341 | 0.048317742 |
| DHPS | -2.118574427 | 0.804489619 | 0.008452499 | 0.048317742 |
| ALDH3A1 | 2.651959711 | 1.007492218 | 0.008482432 | 0.048462468 |
| LDLRAD4 | -2.708129354 | 1.029165305 | 0.008503783 | 0.048553114 |
| ZNF83 | -2.129184673 | 0.809196261 | 0.008507545 | 0.048553114 |
| GNB5 | -2.714719274 | 1.032077509 | 0.008529841 | 0.048636728 |
| AKAP13 | -2.199866073 | 0.836472861 | 0.008540215 | 0.048636728 |
| MXRA7 | -2.395640534 | 0.910966928 | 0.008544071 | 0.048636728 |
| THRB | -2.700030939 | 1.026775471 | 0.008547996 | 0.048636728 |
| WBSCR22 | -2.048307312 | 0.778944406 | 0.008548697 | 0.048636728 |
| CYP51A1 | 1.675545336 | 0.637200338 | 0.008549986 | 0.048636728 |
| IGJ | -2.674575343 | 1.017233629 | 0.008557001 | 0.048650278 |
| PNPT1 | 1.69220171 | 0.643712096 | 0.00856823 | 0.048663556 |
| NOTCH2 | -2.487433345 | 0.946236234 | 0.008569538 | 0.048663556 |
| FGF1 | -2.709295022 | 1.030691459 | 0.008573239 | 0.048663556 |
| ITPRIP | -1.692376788 | 0.643883658 | 0.008579032 | 0.048666875 |
| CCDC88C | -2.703881611 | 1.028853927 | 0.008587539 | 0.048666875 |
| SON | -1.849134742 | 0.703674354 | 0.008593211 | 0.048666875 |
| CHL1 | -2.715598586 | 1.033446331 | 0.008596144 | 0.048666875 |
| GALNT15 | -2.716693874 | 1.033876417 | 0.008596996 | 0.048666875 |
| FAHD2A | -2.699706408 | 1.027634638 | 0.008611419 | 0.048680162 |
| FAM135A | -2.700375966 | 1.028231564 | 0.008633562 | 0.048680162 |
| SMG5 | -2.045162445 | 0.778743664 | 0.008633565 | 0.048680162 |
| PARP3 | -2.708884923 | 1.031528335 | 0.00863723 | 0.048680162 |
| USP30 | -2.721789113 | 1.036569621 | 0.008645427 | 0.048680162 |
| RGAG4 | -2.697976337 | 1.027623021 | 0.008653366 | 0.048680162 |
| LCE1C | 2.72094441 | 1.036860923 | 0.008684933 | 0.048680162 |
| HCAR1 | 2.72094441 | 1.036860923 | 0.008684933 | 0.048680162 |
| LINC00331 | 2.72094441 | 1.036860923 | 0.008684933 | 0.048680162 |
| LOC100505619 | 2.72094441 | 1.036860923 | 0.008684933 | 0.048680162 |
| CETN1 | 2.72094441 | 1.036860923 | 0.008684933 | 0.048680162 |
| LILRP2 | 2.72094441 | 1.036860923 | 0.008684933 | 0.048680162 |
| SCP2D1 | 2.72094441 | 1.036860923 | 0.008684933 | 0.048680162 |
| LINC00308 | 2.72094441 | 1.036860923 | 0.008684933 | 0.048680162 |
| SPATA16 | 2.72094441 | 1.036860923 | 0.008684933 | 0.048680162 |
| CPA5 | 2.72094441 | 1.036860923 | 0.008684933 | 0.048680162 |
| IFNA6 | 2.72094441 | 1.036860923 | 0.008684933 | 0.048680162 |
| RPAP2 | 1.808953784 | 0.689334934 | 0.008685238 | 0.048680162 |
| ZNF583 | 1.966159662 | 0.749298229 | 0.008690341 | 0.048680162 |
| GNB2L1 | -1.333675445 | 0.508273209 | 0.008692058 | 0.048680162 |
| TMEM50A | 1.523453308 | 0.580669965 | 0.008700256 | 0.048700101 |
| PDDC1 | -2.696021988 | 1.027832722 | 0.00871551 | 0.048711213 |
| ACSF2 | -2.697868871 | 1.028542113 | 0.008715855 | 0.048711213 |
| SGMS1 | -2.298986734 | 0.876475264 | 0.008716157 | 0.048711213 |
| COL4A1 | 1.456186991 | 0.555262881 | 0.008728261 | 0.048752912 |
| GPR97 | 2.234696339 | 0.852505776 | 0.008758823 | 0.048897605 |
| PSMD4 | -1.840159092 | 0.702092847 | 0.008768188 | 0.048907749 |
| ZC3H6 | -2.700672057 | 1.030439283 | 0.008769954 | 0.048907749 |
| TRMT44 | -2.690354783 | 1.026651908 | 0.008779755 | 0.048918591 |
| ELP2 | -2.287140909 | 0.872802502 | 0.008781216 | 0.048918591 |
| SLC6A4 | -2.547083963 | 0.972173063 | 0.008793228 | 0.048946142 |
| VPS39 | -2.143696992 | 0.818268425 | 0.008798218 | 0.048946142 |
| SAFB | -2.062877316 | 0.787441272 | 0.008800144 | 0.048946142 |
| GALT | -2.717464267 | 1.037582122 | 0.008817881 | 0.049001853 |
| MYC | -2.327240657 | 0.888608124 | 0.008819494 | 0.049001853 |
| ARHGAP32 | -2.688285297 | 1.026779154 | 0.008840201 | 0.049090934 |
| RND3 | -2.039511603 | 0.77913246 | 0.00885324 | 0.049137353 |
| FARSA | -2.030940125 | 0.77602971 | 0.008868278 | 0.049194815 |
| TBC1D2 | -2.15310487 | 0.823147302 | 0.008904524 | 0.049369802 |
| THBS3 | -2.634760324 | 1.007605794 | 0.008926086 | 0.049463235 |
| CYTIP | 2.017265538 | 0.771548085 | 0.008934017 | 0.049475909 |
| RAB11B | 1.830069277 | 0.699989392 | 0.008937796 | 0.049475909 |
| DGUOK | 1.549897831 | 0.592977132 | 0.008955284 | 0.049517547 |
| CXorf36 | -2.71979046 | 1.040621334 | 0.008958818 | 0.049517547 |
| HLA-F | -1.699958453 | 0.650441202 | 0.008960784 | 0.049517547 |
| APBB3 | -2.582201132 | 0.988055462 | 0.00896418 | 0.049517547 |
| VSIG10 | -2.701390638 | 1.033735363 | 0.008969032 | 0.049518298 |
| DYNLRB1 | 1.63071291 | 0.62413249 | 0.008981245 | 0.049554503 |
| USP9Y | -2.523675358 | 0.965954547 | 0.008985028 | 0.049554503 |
| EMB | 1.68567549 | 0.645321091 | 0.008997477 | 0.049596484 |
| CHD4 | -1.751507155 | 0.670568133 | 0.009002085 | 0.049596484 |
| BTN2A2 | -2.644676276 | 1.013030457 | 0.009036816 | 0.049761724 |
| PDK4 | -2.468058051 | 0.945603825 | 0.009053325 | 0.0498265 |
| RNF167 | 1.639409374 | 0.628338422 | 0.009077581 | 0.04993383 |
| DCPS | -2.682931407 | 1.02853499 | 0.009094056 | 0.049998262 |
| S100A16 | -1.817038643 | 0.696661394 | 0.009101732 | 0.050001293 |
| HDLBP | -1.51814885 | 0.582113926 | 0.009107491 | 0.050001293 |
| LPHN2 | -1.984895635 | 0.761097125 | 0.009108892 | 0.050001293 |
| SNTN | 2.422491858 | 0.929017397 | 0.009118342 | 0.050002186 |
| RNU6ATAC | 2.684724936 | 1.029586387 | 0.009118578 | 0.050002186 |
| STK11IP | -2.684682097 | 1.029740549 | 0.00913009 | 0.050039185 |
| KHSRP | 1.868043698 | 0.71678404 | 0.009156748 | 0.050145079 |
| C4orf27 | -2.683552299 | 1.029757764 | 0.009160554 | 0.050145079 |
| S100A2 | -2.737292846 | 1.050427568 | 0.009163737 | 0.050145079 |
| FAM3A | -2.513989183 | 0.964808781 | 0.009169035 | 0.050147936 |
| HOXA5 | -2.698483271 | 1.03632 | 0.009216715 | 0.050357019 |
| SUOX | -2.685808028 | 1.031454285 | 0.009216855 | 0.050357019 |
| GATSL3 | -2.698991048 | 1.036833032 | 0.009238215 | 0.050447475 |
| AAK1 | -2.159807349 | 0.830493116 | 0.009305209 | 0.050774077 |
| TMEM199 | -2.499472533 | 0.96122151 | 0.009313997 | 0.050774077 |
| MBTPS2 | 1.93689278 | 0.744884742 | 0.00931536 | 0.050774077 |
| PRKD1 | -2.671916848 | 1.027599119 | 0.00931817 | 0.050774077 |
| MXD4 | -2.655028872 | 1.021162403 | 0.0093222 | 0.050774077 |
| SFI1 | -2.671219647 | 1.027681621 | 0.009342288 | 0.050844181 |
| TANC2 | -2.50277714 | 0.962914729 | 0.009345003 | 0.050844181 |
| EEF1D | -1.772034059 | 0.681814118 | 0.009349611 | 0.050844181 |
| PODN | -2.722488748 | 1.047585869 | 0.009354438 | 0.050844181 |
| RHOG | 1.399570142 | 0.538625189 | 0.009365605 | 0.050878542 |
| LOC283174 | -2.671603787 | 1.028335746 | 0.009377181 | 0.050915086 |
| VGLL4 | -2.207656444 | 0.849818776 | 0.009382401 | 0.050917105 |
| PTPN1 | -2.257778305 | 0.869252358 | 0.009393806 | 0.050943954 |
| WDR44 | -2.676440666 | 1.030485872 | 0.009397052 | 0.050943954 |
| RAI1 | -2.673535583 | 1.02952268 | 0.009407778 | 0.050975789 |
| ABCC3 | 2.278901403 | 0.877668923 | 0.009416827 | 0.050985542 |
| PPAT | -2.676361 | 1.030815915 | 0.009421948 | 0.050985542 |
| WDYHV1 | -2.672867765 | 1.029502229 | 0.009424144 | 0.050985542 |
| SBF2 | -2.279626215 | 0.878139326 | 0.00943234 | 0.051003608 |
| NEXN | 2.081168969 | 0.801787433 | 0.009440896 | 0.051023595 |
| PCCA | -2.673639652 | 1.030216251 | 0.00945299 | 0.051062679 |
| FBXO44 | -2.671026466 | 1.029341492 | 0.009462159 | 0.05108593 |
| DPH2 | -2.179682168 | 0.840071222 | 0.009469012 | 0.051096655 |
| PDZD11 | 1.958108952 | 0.754737228 | 0.009474935 | 0.051102359 |
| PPAP2A | -2.634814688 | 1.015740604 | 0.009487095 | 0.051120881 |
| UBE2Q1 | -2.041906043 | 0.787181034 | 0.009488106 | 0.051120881 |
| CD164 | 2.058527306 | 0.793676111 | 0.009495988 | 0.051137114 |
| SLC26A4 | 2.416976963 | 0.932271181 | 0.009526219 | 0.051273616 |
| NAT10 | -2.102499885 | 0.811511108 | 0.009574047 | 0.051504645 |
| FGD5 | -2.242954398 | 0.866195687 | 0.009613455 | 0.051690163 |
| THSD7B | 2.71974218 | 1.05047905 | 0.009624128 | 0.051721069 |
| RGL1 | 1.597628265 | 0.617304291 | 0.009651466 | 0.051841456 |
| ZNF496 | -2.660678239 | 1.028258317 | 0.009665887 | 0.051892369 |
| NKTR | -1.796166877 | 0.694268463 | 0.009677741 | 0.051929462 |
| MTA2 | -1.800686881 | 0.696121105 | 0.009688763 | 0.051962054 |
| IGIP | -2.664840959 | 1.030765927 | 0.009729381 | 0.052153254 |
| LINS | -2.659602863 | 1.028847626 | 0.009737028 | 0.052167619 |
| QTRT1 | -2.425906486 | 0.938760192 | 0.009761651 | 0.052272871 |
| WIPI2 | -1.991466114 | 0.771131858 | 0.009808069 | 0.052409472 |
| ADCY3 | -2.597009113 | 1.005733975 | 0.009817187 | 0.052409472 |
| ODF3B | -2.545767287 | 0.985941298 | 0.009821029 | 0.052409472 |
| ANKRD27 | -2.273893029 | 0.880665762 | 0.009822505 | 0.052409472 |
| STEAP1 | 2.143301672 | 0.830095103 | 0.009823092 | 0.052409472 |
| MRFAP1L1 | -2.01280077 | 0.779569544 | 0.009824704 | 0.052409472 |
| PRRC2A | -1.783527833 | 0.690777453 | 0.009825412 | 0.052409472 |
| SYMPK | -2.106029961 | 0.815704215 | 0.009827088 | 0.052409472 |
| POP5 | -2.667984369 | 1.033455558 | 0.009833922 | 0.052419298 |
| WASF3 | -2.663329315 | 1.031847693 | 0.009847852 | 0.052451027 |
| IPO4 | -2.183084149 | 0.845810502 | 0.009849864 | 0.052451027 |
| COMMD10 | -2.65689268 | 1.029544039 | 0.009861454 | 0.052486129 |
| ZNF211 | -2.472333438 | 0.95888201 | 0.009927339 | 0.052809906 |
| PI4K2A | -2.207474528 | 0.8562364 | 0.009934139 | 0.052809906 |
| ADAMTS1 | -2.364410899 | 0.917148993 | 0.009937375 | 0.052809906 |
| PFKFB4 | -2.665870887 | 1.034372327 | 0.00995802 | 0.052879997 |
| SOCS1 | 2.056393013 | 0.797919719 | 0.009960635 | 0.052879997 |
| C2CD4B | 1.526434335 | 0.592373974 | 0.009971716 | 0.05291207 |
| GUF1 | 1.836494137 | 0.712883271 | 0.009990734 | 0.052986209 |
| MAPK9 | -2.307820494 | 0.895998755 | 0.010003853 | 0.053029006 |
| FAM193A | -2.315686045 | 0.89919831 | 0.010015941 | 0.053066296 |
| TMEM259 | -2.116962064 | 0.822156556 | 0.010027219 | 0.053099259 |
| ADAT2 | -2.648211518 | 1.028661112 | 0.010040667 | 0.053143673 |
| MICA | -2.180466902 | 0.847070503 | 0.010049343 | 0.053145967 |
| TCEA3 | -2.675772399 | 1.039513534 | 0.010051223 | 0.053145967 |
| FAM84B | -2.493972141 | 0.968981758 | 0.010058633 | 0.053158383 |
| PNLDC1 | 2.710837717 | 1.053464326 | 0.010074546 | 0.053209615 |
| ZFP36L2 | -1.964101735 | 0.763340242 | 0.010081079 | 0.053209615 |
| ARHGEF18 | -2.36750719 | 0.920152218 | 0.010083529 | 0.053209615 |
| PHC1 | -2.647189064 | 1.029281277 | 0.010114762 | 0.053347623 |
| ELMOD2 | -2.649777323 | 1.030430408 | 0.010125177 | 0.053356291 |
| SIRPA | 1.515672498 | 0.589485455 | 0.010135299 | 0.053356291 |
| IL4R | -1.879146658 | 0.730872835 | 0.010137625 | 0.053356291 |
| MAP2K3 | -1.752923696 | 0.681891467 | 0.010149952 | 0.053356291 |
| BCL6 | 1.672946146 | 0.650791285 | 0.010151257 | 0.053356291 |
| ADCK3 | -2.604650438 | 1.013249303 | 0.010152489 | 0.053356291 |
| GRWD1 | -2.066105214 | 0.80376295 | 0.010153995 | 0.053356291 |
| FAM216B | 2.389434205 | 0.929622124 | 0.010160213 | 0.053356291 |
| BCL11A | 2.2592565 | 0.878998203 | 0.010162135 | 0.053356291 |
| PITRM1 | -1.944731474 | 0.756797895 | 0.010179131 | 0.053366261 |
| MEIS2 | -2.498729441 | 0.972396133 | 0.010179777 | 0.053366261 |
| ARPC5L | -2.037269062 | 0.792848456 | 0.010182873 | 0.053366261 |
| EXOSC4 | -2.258750166 | 0.879060078 | 0.010184362 | 0.053366261 |
| NYNRIN | -2.664838033 | 1.03721612 | 0.010192726 | 0.05338345 |
| ARRDC1 | -2.443098096 | 0.95103098 | 0.010202356 | 0.053388368 |
| PAAF1 | -2.657468198 | 1.034499579 | 0.010203833 | 0.053388368 |
| LOC729737 | 2.469882318 | 0.961665189 | 0.010218714 | 0.053406619 |
| CYBRD1 | -2.361451019 | 0.919464098 | 0.010220143 | 0.053406619 |
| MARS | -2.239395219 | 0.871968006 | 0.010222579 | 0.053406619 |
| ZNF12 | -2.462428942 | 0.958897747 | 0.010229346 | 0.053415395 |
| IKBKAP | -2.249616455 | 0.876201148 | 0.01024449 | 0.053467889 |
| NR1H2 | -2.084267178 | 0.811976527 | 0.010261051 | 0.053505464 |
| FAM110B | -2.647202758 | 1.03135492 | 0.010266448 | 0.053505464 |
| LRRC37BP1 | -2.642448712 | 1.029509877 | 0.010266976 | 0.053505464 |
| KRT17 | -2.68656466 | 1.046877422 | 0.010280032 | 0.053521248 |
| MAPK7 | -2.642592031 | 1.029806583 | 0.010284763 | 0.053521248 |
| ETV1 | -2.66576062 | 1.038842539 | 0.010285295 | 0.053521248 |
| GALK2 | -2.64032765 | 1.029025139 | 0.010292232 | 0.05353082 |
| TSPAN9 | -2.49751345 | 0.973625431 | 0.010312566 | 0.05360228 |
| SNORA45 | 2.131436826 | 0.830956506 | 0.010316386 | 0.05360228 |
| FOXO4 | -2.555776458 | 0.996452463 | 0.010321285 | 0.05360228 |
| MIR1976 | 2.681190629 | 1.045439675 | 0.010327885 | 0.053610047 |
| ECHDC3 | -2.652641881 | 1.034404863 | 0.010335031 | 0.053620634 |
| PNPLA6 | -2.170829325 | 0.846640538 | 0.010345841 | 0.05365021 |
| CCDC107 | -2.638503019 | 1.029169873 | 0.010355716 | 0.053669627 |
| C20orf27 | -2.668957527 | 1.041104588 | 0.010359807 | 0.053669627 |
| NMT2 | -2.640919411 | 1.03025865 | 0.010366578 | 0.05367822 |
| C12orf43 | -2.644265843 | 1.031642538 | 0.010372396 | 0.053681878 |
| SAT1 | 2.025966523 | 0.790536471 | 0.010383954 | 0.053715221 |
| ENTHD2 | -2.639673419 | 1.030077705 | 0.010389274 | 0.053716281 |
| DPH5 | -2.637282342 | 1.029220346 | 0.010394915 | 0.053719 |
| VGLL3 | -2.463034143 | 0.96133445 | 0.010404162 | 0.053740338 |
| USP25 | 1.708954918 | 0.667095901 | 0.010413637 | 0.053743105 |
| ZNF45 | -2.636551487 | 1.029203499 | 0.010414934 | 0.053743105 |
| FAM65C | -2.647586376 | 1.033644185 | 0.01042483 | 0.05376775 |
| PLCB3 | -2.177013161 | 0.850032263 | 0.01043429 | 0.053775596 |
| PET100 | -2.13284375 | 0.832832598 | 0.010438598 | 0.053775596 |
| FTSJD1 | -2.641434259 | 1.031468733 | 0.010441714 | 0.053775596 |
| MACC1 | -2.660849301 | 1.039146566 | 0.010448852 | 0.053785977 |
| RBM4 | -2.070124892 | 0.808511054 | 0.010454684 | 0.053789631 |
| RFTN1 | -1.829364656 | 0.714772578 | 0.010486327 | 0.053926011 |
| STK36 | -2.634091056 | 1.029273133 | 0.010492062 | 0.053929096 |
| RDX | -2.068047306 | 0.808226758 | 0.010505035 | 0.053961001 |
| KPRP | 2.678483616 | 1.046994195 | 0.010519734 | 0.053961001 |
| C7orf71 | 2.678483616 | 1.046994195 | 0.010519734 | 0.053961001 |
| HELLS | 2.391428321 | 0.934865717 | 0.010526255 | 0.053961001 |
| LMOD1 | -2.69195381 | 1.052371551 | 0.010527972 | 0.053961001 |
| SHE | -2.675937979 | 1.04621699 | 0.010535861 | 0.053961001 |
| AXIN1 | -2.454868376 | 0.959861934 | 0.010542077 | 0.053961001 |
| LAMC3 | -2.45913697 | 0.961545703 | 0.010543265 | 0.053961001 |
| PRX | -2.66477278 | 1.041968063 | 0.010544517 | 0.053961001 |
| FBXL6 | -2.631802976 | 1.029202353 | 0.010554013 | 0.053983289 |
| ALKBH5 | -2.007560155 | 0.785338093 | 0.010579172 | 0.054085629 |
| TLL1 | -2.64041469 | 1.03331507 | 0.010610083 | 0.054179836 |
| NARF | -2.076442435 | 0.812634777 | 0.010612753 | 0.054179836 |
| LRP6 | -2.233313039 | 0.87403116 | 0.010613077 | 0.054179836 |
| GTF2H1 | -2.060012929 | 0.806428061 | 0.010634329 | 0.054261948 |
| SENP5 | -2.253637412 | 0.882333927 | 0.0106439 | 0.054284406 |
| MPG | -2.134085692 | 0.835652278 | 0.010655558 | 0.054317481 |
| KLF10 | -2.332162908 | 0.913583619 | 0.010687204 | 0.054435325 |
| UBN1 | -2.038467049 | 0.798561646 | 0.010689969 | 0.054435325 |
| C1QTNF1 | -2.149961864 | 0.842285081 | 0.010694227 | 0.054435325 |
| ANK3 | -2.626991187 | 1.029660956 | 0.010731676 | 0.054599481 |
| TAGAP | 2.201091674 | 0.86295261 | 0.010752167 | 0.054619001 |
| DNTTIP1 | -2.267073446 | 0.888862992 | 0.010755867 | 0.054619001 |
| ITLN2 | -2.625555392 | 1.029436617 | 0.010757533 | 0.054619001 |
| NDUFAB1 | -1.988860143 | 0.779801033 | 0.010757739 | 0.054619001 |
| COX4I1 | -1.681079005 | 0.659156506 | 0.010761519 | 0.054619001 |
| KIAA0226 | 1.577184325 | 0.618523668 | 0.010774841 | 0.054660197 |
| YKT6 | -2.005855504 | 0.786768766 | 0.010788202 | 0.054701549 |
| RBM26 | -2.455937832 | 0.963387215 | 0.01079475 | 0.054708334 |
| PEX1 | -2.627088609 | 1.030980548 | 0.010829724 | 0.05485911 |
| HPCAL1 | -1.794717256 | 0.70451984 | 0.010851862 | 0.054944744 |
| DHRS12 | -2.64448972 | 1.038411564 | 0.010875678 | 0.055027693 |
| MYADM | 1.669089027 | 0.655426213 | 0.010878725 | 0.055027693 |
| RUNX1T1 | -2.640348299 | 1.036970255 | 0.010889837 | 0.05505738 |
| ZNF331 | -1.984880717 | 0.779869971 | 0.010923295 | 0.055199961 |
| TSPYL4 | -2.453247086 | 0.964016704 | 0.01093347 | 0.055224804 |
| KLHDC7B | 2.635182609 | 1.035768666 | 0.010953442 | 0.055295859 |
| C11orf54 | -2.628616122 | 1.033247598 | 0.010958069 | 0.055295859 |
| GIT1 | -2.052837898 | 0.807462701 | 0.011011567 | 0.055539126 |
| CCDC71 | -2.450690419 | 0.964121558 | 0.011025504 | 0.055577289 |
| TRAPPC12 | -2.350231305 | 0.924737925 | 0.011037458 | 0.055577289 |
| PLXNA1 | -2.386628465 | 0.939083452 | 0.011039547 | 0.055577289 |
| TIAM1 | -2.636287312 | 1.037328239 | 0.011040303 | 0.055577289 |
| PNRC1 | 1.928638026 | 0.75905179 | 0.011058282 | 0.055641118 |
| SERPINE2 | 2.068329552 | 0.814227039 | 0.011077741 | 0.055655935 |
| ANKRD46 | -2.628420631 | 1.034746381 | 0.011080199 | 0.055655935 |
| DYNC2LI1 | -2.628109077 | 1.034642642 | 0.01108167 | 0.055655935 |
| MAPK11 | -2.492894721 | 0.981427398 | 0.011083015 | 0.055655935 |
| TMTC1 | -2.663587287 | 1.04873606 | 0.011091363 | 0.055655935 |
| DHX38 | -1.891778497 | 0.744901671 | 0.011096815 | 0.055655935 |
| PP7080 | -2.6242608 | 1.033388592 | 0.011102011 | 0.055655935 |
| RBM39 | -1.557799596 | 0.613446877 | 0.011103627 | 0.055655935 |
| LINC00667 | -2.363422498 | 0.930832668 | 0.011115673 | 0.055689735 |
| SIK2 | -2.119588501 | 0.834863635 | 0.011121941 | 0.055692733 |
| MICB | 1.978064838 | 0.779167944 | 0.011126879 | 0.055692733 |
| MTM1 | -2.614294861 | 1.030232494 | 0.011162263 | 0.055843223 |
| CCRL2 | 2.137350951 | 0.842446688 | 0.011178281 | 0.055896726 |
| HSPB7 | -2.641453849 | 1.041223506 | 0.011184689 | 0.055902152 |
| RPL4 | -1.285889273 | 0.506912841 | 0.011190062 | 0.055902396 |
| PIGO | -2.614829258 | 1.030882674 | 0.011196818 | 0.055909553 |
| GRHL1 | -2.611993821 | 1.030493872 | 0.011254335 | 0.056170043 |
| ZNF385D | -2.639256766 | 1.041337157 | 0.011261171 | 0.056177462 |
| SELRC1 | -2.612211045 | 1.030828233 | 0.011273992 | 0.056210123 |
| ZNF131 | -2.228150196 | 0.879369122 | 0.011283138 | 0.056210123 |
| CEP192 | -2.612335468 | 1.031065038 | 0.011288843 | 0.056210123 |
| NCDN | -2.61186177 | 1.030881689 | 0.01128913 | 0.056210123 |
| CDK11A | -2.625189602 | 1.036416738 | 0.011310776 | 0.056291214 |
| PDGFC | -2.612647291 | 1.031603003 | 0.011321709 | 0.056318931 |
| ADM5 | -2.648502467 | 1.045880143 | 0.011331076 | 0.056321956 |
| TUBB6 | -2.256599897 | 0.891189558 | 0.011337482 | 0.056321956 |
| ZNF181 | -2.60856846 | 1.030202669 | 0.011338407 | 0.056321956 |
| MAN1B1 | -2.481149785 | 0.980049159 | 0.01135245 | 0.056365046 |
| TMEM60 | 1.829268293 | 0.722706969 | 0.011369435 | 0.056422704 |
| CEP104 | -2.607192559 | 1.03022342 | 0.011383323 | 0.056461005 |
| LOC100288778 | -2.635134444 | 1.041322675 | 0.011387907 | 0.056461005 |
| GAS5 | -1.799903133 | 0.711530094 | 0.011418503 | 0.056577124 |
| ZNF136 | -2.606339815 | 1.030372286 | 0.011422103 | 0.056577124 |
| NRARP | -2.625235744 | 1.038045014 | 0.011438178 | 0.056605527 |
| PFAS | -2.6055783 | 1.030280322 | 0.011438823 | 0.056605527 |
| TFEB | -2.616423649 | 1.034633801 | 0.011444009 | 0.056605527 |
| SIRT5 | -2.629038651 | 1.039970825 | 0.011471674 | 0.056715653 |
| DHX35 | -2.606874301 | 1.031326392 | 0.01148154 | 0.056737717 |
| HECTD3 | 1.593944578 | 0.630711982 | 0.01149712 | 0.056787986 |
| MAPK8 | -2.630446506 | 1.04135966 | 0.011537831 | 0.056962277 |
| MTA3 | -2.605684935 | 1.031710679 | 0.011550204 | 0.056996565 |
| ZBTB9 | -2.607239106 | 1.032490737 | 0.011563453 | 0.057035145 |
| CCNJL | -2.602267866 | 1.030660267 | 0.011574599 | 0.057063317 |
| SMARCAL1 | -2.604251725 | 1.03153935 | 0.011582127 | 0.057073633 |
| WDR16 | 2.319615133 | 0.918967216 | 0.011597698 | 0.057123559 |
| ZNF783 | -2.453718968 | 0.97239096 | 0.011623032 | 0.057171033 |
| UBQLN4 | -2.094563038 | 0.830062414 | 0.011623267 | 0.057171033 |
| C6orf136 | -2.600139675 | 1.030488324 | 0.011628854 | 0.057171033 |
| XPA | -2.614070976 | 1.036013475 | 0.011629167 | 0.057171033 |
| IL1R2 | 2.498951043 | 0.990452903 | 0.011634558 | 0.057171033 |
| LEPR | -2.638555032 | 1.046157423 | 0.011664342 | 0.057282928 |
| FLT4 | -2.03535298 | 0.807095851 | 0.01167484 | 0.057282928 |
| MLX | -2.014042025 | 0.79868153 | 0.011678643 | 0.057282928 |
| NKD1 | -2.612256894 | 1.035913812 | 0.01167915 | 0.057282928 |
| WDR35 | -2.602897073 | 1.032321896 | 0.011688869 | 0.057303833 |
| STYX | -2.450101624 | 0.97180809 | 0.011696252 | 0.05731327 |
| GREM1 | -2.598140062 | 1.030632276 | 0.011704893 | 0.057328862 |
| CSF2RA | -2.607582515 | 1.034544014 | 0.011718363 | 0.057368077 |
| SNRNP200 | -1.809313532 | 0.71795917 | 0.011732863 | 0.057412302 |
| KLC4 | -2.604465358 | 1.033636665 | 0.011745133 | 0.057445569 |
| STK25 | -2.041074544 | 0.810121946 | 0.011753305 | 0.05745878 |
| FLNA | -1.853908058 | 0.736097061 | 0.011783424 | 0.057579216 |
| C1S | 1.621938433 | 0.644079441 | 0.011794718 | 0.057591549 |
| COQ6 | -2.599834992 | 1.032433718 | 0.011796917 | 0.057591549 |
| RNU12 | 2.634026076 | 1.046089521 | 0.011803208 | 0.057595486 |
| KPNB1 | 1.679139374 | 0.66699207 | 0.011819759 | 0.057649461 |
| ASB13 | -2.621522345 | 1.041403241 | 0.011825874 | 0.05765251 |
| ANAPC2 | -2.503964983 | 0.994828348 | 0.01183649 | 0.05766941 |
| ZNF697 | -2.505499932 | 0.995532436 | 0.011844497 | 0.05766941 |
| METTL21A | -2.596040117 | 1.031523649 | 0.011845816 | 0.05766941 |
| STON1 | -2.619152639 | 1.040797026 | 0.011853113 | 0.057678191 |
| ZMYND12 | 2.472474951 | 0.982706119 | 0.011869986 | 0.057733544 |
| PASK | -2.595102915 | 1.031550865 | 0.011878632 | 0.05774885 |
| NSMF | -2.142094451 | 0.851599705 | 0.01189051 | 0.057779847 |
| CAPZB | 1.491163111 | 0.592961492 | 0.01191093 | 0.0578523 |
| BCL7A | -2.599784305 | 1.033946128 | 0.011922525 | 0.057881846 |
| DHX30 | -1.787291956 | 0.710895199 | 0.011932215 | 0.057902121 |
| PDE5A | -2.619558054 | 1.042116141 | 0.011947503 | 0.057943893 |
| PARD3 | -2.082236431 | 0.828400492 | 0.011951859 | 0.057943893 |
| WAPAL | -2.108360361 | 0.838999421 | 0.011972761 | 0.058001204 |
| POMT1 | -2.495031128 | 0.9928938 | 0.011974727 | 0.058001204 |
| TTC31 | -2.307060537 | 0.91837966 | 0.012001545 | 0.058084084 |
| ST6GAL1 | -1.971008569 | 0.784618731 | 0.012002901 | 0.058084084 |
| ZNF212 | -2.593929869 | 1.032954717 | 0.012033009 | 0.058151169 |
| DYNC1H1 | -1.796993403 | 0.715634768 | 0.012037326 | 0.058151169 |
| CD34 | -2.628650662 | 1.046852707 | 0.012038855 | 0.058151169 |
| AKR1C2 | 1.971747149 | 0.785243281 | 0.012038915 | 0.058151169 |
| MIA3 | 1.694241655 | 0.674776213 | 0.012045108 | 0.058154333 |
| LAMB1 | -2.220379792 | 0.884405691 | 0.012052971 | 0.058165554 |
| RGCC | -2.028048674 | 0.808593153 | 0.012137543 | 0.058546781 |
| NQO1 | 1.648235883 | 0.657401181 | 0.012169202 | 0.058663716 |
| RIMS3 | -2.619704323 | 1.044918188 | 0.012172959 | 0.058663716 |
| PIK3C2B | -1.95544493 | 0.780317582 | 0.012211931 | 0.058824535 |
| SEMA4A | 1.802837929 | 0.719474323 | 0.012218471 | 0.058829052 |
| MYO6 | -2.082850804 | 0.83137625 | 0.012234595 | 0.058871535 |
| DAG1 | -2.352544402 | 0.939067648 | 0.012238507 | 0.058871535 |
| SMYD3 | -2.595797848 | 1.036653974 | 0.012279258 | 0.059040516 |
| PHPT1 | -2.226850196 | 0.889394095 | 0.012287322 | 0.059052251 |
| KDELR3 | 1.993983291 | 0.796460533 | 0.012295233 | 0.059063241 |
| COMMD3 | -2.581809609 | 1.031455244 | 0.012311953 | 0.059116514 |
| LPXN | 1.648460804 | 0.658775553 | 0.012338575 | 0.059217267 |
| SCML1 | -2.155708014 | 0.861691274 | 0.012359255 | 0.059236276 |
| CEP164 | -2.461636867 | 0.984017043 | 0.012362648 | 0.059236276 |
| DEF8 | -2.116560837 | 0.846087362 | 0.012363819 | 0.059236276 |
| PSMB10 | -1.734468787 | 0.69335765 | 0.0123651 | 0.059236276 |
| SNHG10 | -2.579757016 | 1.031382584 | 0.012375194 | 0.059256074 |
| ZNF629 | -2.378851048 | 0.951118632 | 0.012380519 | 0.059256074 |
| DPT | -2.532349798 | 1.012739089 | 0.012401962 | 0.059318855 |
| URM1 | -2.126653815 | 0.850521734 | 0.012404933 | 0.059318855 |
| PFDN6 | -2.100402147 | 0.840126074 | 0.012415702 | 0.059343328 |
| ERVK13-1 | -2.578101873 | 1.031427446 | 0.012435204 | 0.059409496 |
| PRMT2 | -2.10889989 | 0.84413364 | 0.01247902 | 0.059591717 |
| EFTUD1 | -2.508918886 | 1.004328606 | 0.0124859 | 0.059597473 |
| RPL14 | -1.586269078 | 0.635265287 | 0.012524248 | 0.05975335 |
| NSUN5P1 | -2.581226726 | 1.033910659 | 0.012540215 | 0.059780336 |
| TMEM222 | -2.02158121 | 0.80975444 | 0.012541289 | 0.059780336 |
| GCC1 | -2.245115866 | 0.899411695 | 0.012553007 | 0.059809043 |
| TRAP1 | -1.953849229 | 0.78288781 | 0.012571062 | 0.059867902 |
| FRYL | -2.030594079 | 0.813699195 | 0.012577638 | 0.059872067 |
| CARHSP1 | -1.84715094 | 0.740284092 | 0.012588899 | 0.059875139 |
| ETS1 | -1.973704122 | 0.791009947 | 0.012589687 | 0.059875139 |
| FAM134B | -2.579136031 | 1.033818913 | 0.01260403 | 0.059916216 |
| CACTIN | -2.577785653 | 1.03334657 | 0.012609943 | 0.0599172 |
| ZNF185 | -2.591161419 | 1.038795343 | 0.012617359 | 0.05992532 |
| TOLLIP | -2.081063528 | 0.834554676 | 0.012644728 | 0.06002816 |
| IER3 | -2.148113281 | 0.861741149 | 0.012675473 | 0.060143261 |
| AGPAT2 | -2.298945243 | 0.922300533 | 0.012680429 | 0.060143261 |
| CCNK | 1.737746295 | 0.697255163 | 0.012693045 | 0.060175919 |
| KCNE4 | -2.221147259 | 0.891648491 | 0.012736367 | 0.060350112 |
| ABHD3 | -2.575658788 | 1.034019086 | 0.012741282 | 0.060350112 |
| TERF2 | 1.508771346 | 0.605778903 | 0.01275167 | 0.060372084 |
| KLHL22 | -2.575614908 | 1.034719945 | 0.012803439 | 0.060589868 |
| THBD | -2.034319231 | 0.817659286 | 0.012847128 | 0.060751026 |
| MAGEF1 | -2.424038917 | 0.974321345 | 0.012849065 | 0.060751026 |
| POGZ | -1.94419059 | 0.781557938 | 0.012861428 | 0.060782111 |
| ATP2C2 | -2.584427739 | 1.039551373 | 0.012915199 | 0.060997683 |
| SMARCA4 | -1.940583151 | 0.780603465 | 0.01291866 | 0.060997683 |
| TRAPPC9 | -2.582635327 | 1.039159308 | 0.012943786 | 0.06108885 |
| CDC40 | -2.565102676 | 1.032244803 | 0.012956048 | 0.061119255 |
| MAPKAPK3 | -1.968694518 | 0.792483223 | 0.012983932 | 0.061223291 |
| C1orf192 | 2.236165095 | 0.900424344 | 0.013011406 | 0.061325303 |
| KIF1C | -2.039987344 | 0.821635652 | 0.013034074 | 0.06140458 |
| POLA1 | -2.562129225 | 1.032228092 | 0.013059788 | 0.06149813 |
| SRSF5 | -1.99164198 | 0.802713641 | 0.013096427 | 0.061643022 |
| CRTC2 | -2.006934335 | 0.808947748 | 0.013104392 | 0.061652878 |
| SNHG12 | -2.313852361 | 0.93272254 | 0.013110593 | 0.061654426 |
| LGALS17A | 2.599352394 | 1.048156232 | 0.013140877 | 0.061699197 |
| FAM126A | -2.082656518 | 0.839871793 | 0.013148136 | 0.061699197 |
| MAN2C1 | -2.192093001 | 0.884014326 | 0.013149185 | 0.061699197 |
| OLA1 | 1.62379851 | 0.654861198 | 0.013152721 | 0.061699197 |
| ZNF134 | -2.568428794 | 1.035852767 | 0.013155538 | 0.061699197 |
| ZUFSP | -2.568428794 | 1.035852767 | 0.013155538 | 0.061699197 |
| IL18BP | 1.765331555 | 0.71200634 | 0.013161242 | 0.061699197 |
| FIS1 | -2.059548277 | 0.830798334 | 0.013175166 | 0.061704271 |
| KAT2A | -2.347505622 | 0.946971223 | 0.013176536 | 0.061704271 |
| RAPGEFL1 | -2.564383317 | 1.03449711 | 0.013179952 | 0.061704271 |
| TRAF4 | -2.06181861 | 0.832143129 | 0.013222445 | 0.06187562 |
| ARRDC4 | -2.098660706 | 0.847174417 | 0.013240005 | 0.061901725 |
| SAP30BP | -1.724246394 | 0.696072236 | 0.013245166 | 0.061901725 |
| TENC1 | -2.539515808 | 1.025273203 | 0.013252301 | 0.061901725 |
| CERK | -2.011987895 | 0.812331761 | 0.013256407 | 0.061901725 |
| POLD1 | -2.558394748 | 1.032953508 | 0.013257497 | 0.061901725 |
| CDH5 | -1.474227881 | 0.595334381 | 0.013275113 | 0.061956429 |
| PLCL2 | -2.558980225 | 1.033651369 | 0.013298624 | 0.062033364 |
| IPO8 | -2.118495689 | 0.855770461 | 0.013303413 | 0.062033364 |
| LUC7L | -1.73548615 | 0.701155875 | 0.013316949 | 0.062068923 |
| CTNNBL1 | -1.797614134 | 0.726629263 | 0.013364401 | 0.062258869 |
| INO80E | -2.569912308 | 1.038864537 | 0.01336956 | 0.062258869 |
| CNTN2 | 2.60478768 | 1.053169832 | 0.013387785 | 0.062316106 |
| LRRC40 | -2.554418569 | 1.032976006 | 0.013403173 | 0.062360088 |
| ATOX1 | -1.77329533 | 0.717192192 | 0.013415202 | 0.062388411 |
| PCSK9 | 2.450431284 | 0.9911503 | 0.013424287 | 0.062403026 |
| ADIPOR2 | -2.174538431 | 0.879684637 | 0.013437746 | 0.062437951 |
| CAMSAP2 | -1.947969277 | 0.788267314 | 0.013465901 | 0.062523098 |
| NAGK | 1.625543457 | 0.65780883 | 0.013467979 | 0.062523098 |
| TMEM186 | -2.55144436 | 1.032596935 | 0.013477328 | 0.06253044 |
| KRT72 | 2.601029038 | 1.052711202 | 0.01348147 | 0.06253044 |
| SEC16A | -1.934449509 | 0.783034409 | 0.013494214 | 0.062561918 |
| PLXNA3 | -2.555098793 | 1.034801674 | 0.013542776 | 0.062738506 |
| SNX10 | 2.085391126 | 0.844667016 | 0.013553235 | 0.062738506 |
| SLC27A1 | -2.559280287 | 1.03663818 | 0.013555675 | 0.062738506 |
| ASAP3 | -2.571208586 | 1.041475605 | 0.013556201 | 0.062738506 |
| LOC284751 | 2.356845086 | 0.955198136 | 0.013610253 | 0.062960908 |
| AUP1 | -1.673641996 | 0.678783252 | 0.013676486 | 0.063239444 |
| RPTOR | -2.387367376 | 0.968593115 | 0.013709802 | 0.063360881 |
| FLJ39051 | 2.323418488 | 0.942698148 | 0.013714816 | 0.063360881 |
| ZNF248 | -2.545032235 | 1.032822371 | 0.013733751 | 0.063420458 |
| DBF4B | 2.31040588 | 0.937697395 | 0.013742902 | 0.063427479 |
| RNPS1 | -2.027358297 | 0.822858936 | 0.013747352 | 0.063427479 |
| MOSPD1 | -2.547352781 | 1.033990476 | 0.013754438 | 0.063432304 |
| ZFYVE20 | -2.25169723 | 0.914140949 | 0.013770928 | 0.063480472 |
| NFIL3 | 1.494126748 | 0.606669252 | 0.013784303 | 0.063501106 |
| PPP1R9A | -2.543586544 | 1.03288721 | 0.013793426 | 0.063501106 |
| L3MBTL3 | -2.54356125 | 1.032878243 | 0.013793546 | 0.063501106 |
| HDHD1 | -2.546927543 | 1.034384078 | 0.013806268 | 0.063531822 |
| SMCR7L | -2.016403952 | 0.819065261 | 0.013822804 | 0.063565056 |
| ID4 | -2.215187092 | 0.899837648 | 0.013825596 | 0.063565056 |
| MAP2K5 | -2.551521714 | 1.036813273 | 0.013857858 | 0.063671258 |
| ZNF384 | -1.837828 | 0.746826442 | 0.013860822 | 0.063671258 |
| TBC1D9B | -1.9453679 | 0.790613648 | 0.013871276 | 0.063691414 |
| TRIM29 | -2.585581595 | 1.051378352 | 0.013923523 | 0.06383935 |
| NCF2 | 1.814899952 | 0.738001665 | 0.013924373 | 0.06383935 |
| GLI3 | -2.5406833 | 1.033184212 | 0.013929332 | 0.06383935 |
| PDK2 | -2.566034606 | 1.043537889 | 0.013933394 | 0.06383935 |
| TBC1D30 | -2.540204608 | 1.033038898 | 0.013933891 | 0.06383935 |
| ZNF689 | -2.542785641 | 1.034421575 | 0.013964647 | 0.063952357 |
| HCG26 | -2.539001986 | 1.033311284 | 0.014004376 | 0.064106343 |
| CASKIN2 | -2.515246876 | 1.024015013 | 0.014039163 | 0.064237584 |
| UACA | -2.252276193 | 0.917235376 | 0.014068674 | 0.0643014 |
| AGFG2 | -2.557293562 | 1.041491737 | 0.014072236 | 0.0643014 |
| FLG | 2.582868485 | 1.051940918 | 0.014075293 | 0.0643014 |
| TAP1 | -1.840119883 | 0.749493217 | 0.014082497 | 0.0643014 |
| SCLT1 | 1.947268624 | 0.793151755 | 0.014084454 | 0.0643014 |
| MS4A15 | -2.56425438 | 1.04451791 | 0.014089851 | 0.0643014 |
| IARS2 | -2.004064001 | 0.816437181 | 0.014102351 | 0.06432787 |
| FARS2 | -2.545485851 | 1.037067232 | 0.014107903 | 0.06432787 |
| BAG6 | -1.557033177 | 0.634500538 | 0.014129627 | 0.064398962 |
| CYP24A1 | 2.51475508 | 1.024957948 | 0.014146557 | 0.064448156 |
| SF3A3 | -1.694929217 | 0.69099267 | 0.014171355 | 0.064520264 |
| DOLK | -2.538202882 | 1.034815969 | 0.014174674 | 0.064520264 |
| PEX2 | -2.121463816 | 0.865001429 | 0.014184559 | 0.064537283 |
| CFP | 2.096176703 | 0.854962872 | 0.014215347 | 0.064649353 |
| SLC18B1 | 1.798926509 | 0.733775714 | 0.01422216 | 0.064652339 |
| LEPREL2 | -2.542864239 | 1.037480373 | 0.014246001 | 0.064697693 |
| TWIST2 | 1.880981191 | 0.767467885 | 0.014250255 | 0.064697693 |
| PHTF2 | -2.537367183 | 1.035286881 | 0.01425062 | 0.064697693 |
| GPKOW | -2.1542047 | 0.87905069 | 0.014261695 | 0.064719992 |
| PLAC9 | -2.522717887 | 1.029689321 | 0.014286429 | 0.064766673 |
| B2M | 1.732220617 | 0.707036526 | 0.01428668 | 0.064766673 |
| GTPBP1 | 1.558899478 | 0.636317334 | 0.014290485 | 0.064766673 |
| ITFG3 | -2.353690483 | 0.960910076 | 0.0143079 | 0.064814644 |
| CDH11 | -2.225261735 | 0.908541674 | 0.014314703 | 0.064814644 |
| PTPN3 | -2.551870664 | 1.041967744 | 0.014321847 | 0.064814644 |
| ZNF839 | -2.54321733 | 1.038476165 | 0.014325758 | 0.064814644 |
| SETD1A | -2.377645806 | 0.970961133 | 0.014335097 | 0.064828963 |
| DCP1B | -2.530477207 | 1.033509087 | 0.014347932 | 0.064859078 |
| MERTK | -2.534759 | 1.035545752 | 0.014375063 | 0.064953761 |
| SLC16A5 | -2.530133321 | 1.033734135 | 0.014382447 | 0.064959174 |
| ANAPC7 | -1.985985278 | 0.811521512 | 0.014395624 | 0.064990734 |
| SP6 | -2.545653432 | 1.040299548 | 0.01440353 | 0.064998484 |
| FIP1L1 | -2.089316967 | 0.854104287 | 0.014436758 | 0.065120445 |
| AK1 | -1.990697068 | 0.8138623 | 0.014445598 | 0.065132343 |
| KRIT1 | -2.236631076 | 0.914570505 | 0.014463 | 0.065180662 |
| UNC93B1 | 1.802619284 | 0.73714379 | 0.014468729 | 0.065180662 |
| PTPRC | 1.997809578 | 0.817076637 | 0.014482403 | 0.065187952 |
| ITGA5 | -1.540503178 | 0.63004692 | 0.014482763 | 0.065187952 |
| SUPT4H1 | 1.604447164 | 0.656302347 | 0.014498197 | 0.065229465 |
| STEAP4 | 2.220269103 | 0.908347762 | 0.014513589 | 0.065270747 |
| ERBB2 | -2.346726195 | 0.960167307 | 0.014522186 | 0.065274544 |
| MRFAP1 | -1.423109861 | 0.582295689 | 0.014526865 | 0.065274544 |
| PTPN6 | 1.425916373 | 0.583628714 | 0.014558034 | 0.065386617 |
| SPIN1 | -1.913221583 | 0.783222318 | 0.014575559 | 0.065437342 |
| CBR1 | -2.427342772 | 0.993764282 | 0.014582941 | 0.065442504 |
| AIMP2 | -2.530438758 | 1.036072906 | 0.014592535 | 0.065457585 |
| LRPAP1 | -1.966530247 | 0.805290768 | 0.014605635 | 0.065488373 |
| TANGO6 | -2.524211237 | 1.03372866 | 0.014612181 | 0.065489354 |
| FCHO2 | -2.251786724 | 0.922221233 | 0.014618326 | 0.065489354 |
| UIMC1 | -2.039839928 | 0.83566974 | 0.014648106 | 0.065586401 |
| PPARGC1B | -2.556027407 | 1.047240093 | 0.014657721 | 0.065586401 |
| SLC12A7 | -2.06536155 | 0.846216028 | 0.014658726 | 0.065586401 |
| SSTR2 | -2.524427396 | 1.034407955 | 0.014668721 | 0.065603169 |
| TRAPPC5 | -2.05879862 | 0.843673754 | 0.014675957 | 0.06560653 |
| SCAP | -2.169037451 | 0.88890233 | 0.014681968 | 0.06560653 |
| FDX1L | -2.528885458 | 1.036485102 | 0.014692683 | 0.065626486 |
| TARDBP | -2.077549087 | 0.851714147 | 0.014717526 | 0.0657095 |
| SCNN1G | -2.545353008 | 1.043670178 | 0.014734161 | 0.065755812 |
| SETD6 | -2.521758404 | 1.034119038 | 0.014746026 | 0.065776317 |
| ZSCAN9 | -2.528317322 | 1.036863495 | 0.014751283 | 0.065776317 |
| VPS33B | -2.356541956 | 0.966839253 | 0.014794661 | 0.065941739 |
| GPR56 | -1.61119174 | 0.66113163 | 0.014808815 | 0.06597682 |
| HID1 | -2.411131154 | 0.989669778 | 0.014838428 | 0.066080716 |
| ZDHHC16 | -2.166527174 | 0.889594901 | 0.014874977 | 0.066190306 |
| VAX1 | 2.542386018 | 1.043972447 | 0.014879451 | 0.066190306 |
| GOLGA2 | -1.947783133 | 0.799832348 | 0.014881946 | 0.066190306 |
| B4GALT7 | -2.23208948 | 0.916676187 | 0.014892554 | 0.066209447 |
| NRDE2 | -2.520037231 | 1.035287635 | 0.014927133 | 0.066335093 |
| ZNF576 | -2.517466084 | 1.034534886 | 0.014956613 | 0.066437982 |
| NUDT16L1 | -2.526267926 | 1.038314388 | 0.014972349 | 0.06647976 |
| C21orf91 | 1.665869537 | 0.684803328 | 0.014989826 | 0.066511527 |
| TTC38 | -2.255592706 | 0.927247495 | 0.014992171 | 0.066511527 |
| HTRA3 | -2.549027658 | 1.048036801 | 0.015007712 | 0.066533148 |
| MROH2B | 2.520366844 | 1.03627346 | 0.015009716 | 0.066533148 |
| DBR1 | -2.51493862 | 1.034252535 | 0.015030286 | 0.066596216 |
| NCOA7 | 1.730705047 | 0.711802547 | 0.015038947 | 0.066606488 |
| ACSF3 | -2.516331944 | 1.03498632 | 0.015045967 | 0.066609487 |
| SESN1 | -2.312901884 | 0.951483876 | 0.015064004 | 0.066626306 |
| ALKBH6 | -2.520530242 | 1.036934988 | 0.015067586 | 0.066626306 |
| SMARCD2 | -2.149928305 | 0.884496053 | 0.015070451 | 0.066626306 |
| PCED1B | -2.522199029 | 1.037699109 | 0.015075146 | 0.066626306 |
| TNFRSF10A | -2.517969636 | 1.036292187 | 0.015107684 | 0.066724788 |
| SNORA58 | 2.538533955 | 1.044780909 | 0.015110137 | 0.066724788 |
| TBC1D22A | -2.104638785 | 0.866325032 | 0.015124378 | 0.066759602 |
| MIR17HG | -2.527184625 | 1.04038683 | 0.01513712 | 0.066785696 |
| ARHGEF10 | -1.926418688 | 0.793116975 | 0.015143821 | 0.066785696 |
| IFI27L1 | -2.522644649 | 1.038643221 | 0.015149369 | 0.066785696 |
| SOX4 | -1.949799077 | 0.803061794 | 0.015184166 | 0.066911004 |
| WBP5 | -1.93446774 | 0.796859961 | 0.015198544 | 0.066946271 |
| KLC1 | -1.618664585 | 0.666872611 | 0.015213891 | 0.066985775 |
| TRIB3 | -2.465791812 | 1.016115442 | 0.015237491 | 0.067061566 |
| DPM3 | -2.338227516 | 0.963811187 | 0.015265329 | 0.067155934 |
| PSMD13 | -1.500988501 | 0.61877239 | 0.015276696 | 0.0671778 |
| LRFN3 | 2.554143334 | 1.053092808 | 0.015292647 | 0.067219791 |
| NDUFB9 | -1.545459896 | 0.637387901 | 0.015322005 | 0.067320658 |
| EVI2A | 2.09124271 | 0.862587587 | 0.015334422 | 0.067347038 |
| FBXO22 | -2.50995265 | 1.035447169 | 0.015349433 | 0.067384783 |
| PPOX | -2.516222204 | 1.038231642 | 0.015368989 | 0.067427518 |
| ACVR1B | -1.911835599 | 0.788918421 | 0.015377558 | 0.067427518 |
| OSBPL2 | -1.961005214 | 0.809215133 | 0.015378431 | 0.067427518 |
| WDR25 | -2.506899345 | 1.034664562 | 0.015396731 | 0.067459439 |
| IMPDH2 | -1.777905903 | 0.733828644 | 0.01540222 | 0.067459439 |
| SCN4B | -2.532803295 | 1.045516313 | 0.015412502 | 0.067459439 |
| IL1B | 2.35417839 | 0.971797718 | 0.015414195 | 0.067459439 |
| BST1 | 2.067788854 | 0.853611973 | 0.015418408 | 0.067459439 |
| CD4 | 2.035108855 | 0.840201263 | 0.015428204 | 0.067459439 |
| KANSL3 | -1.820607268 | 0.751678586 | 0.015432992 | 0.067459439 |
| TRIOBP | -2.03420812 | 0.839902063 | 0.015437104 | 0.067459439 |
| IFRD2 | -1.920886969 | 0.793341085 | 0.015466707 | 0.067532884 |
| SHANK3 | -2.091091368 | 0.863637367 | 0.015466773 | 0.067532884 |
| WDR60 | -2.195023445 | 0.906632845 | 0.01547481 | 0.067534152 |
| LRIG3 | -2.504860765 | 1.034659459 | 0.015479926 | 0.067534152 |
| ARHGAP23 | 1.530716766 | 0.632319077 | 0.01548648 | 0.067534685 |
| ANKRA2 | -2.50517759 | 1.034946434 | 0.015495483 | 0.067545849 |
| EXOC3 | -1.960976074 | 0.810174699 | 0.015501904 | 0.067545849 |
| PCBD1 | 1.484226455 | 0.613284841 | 0.015515139 | 0.067575478 |
| UAP1L1 | -2.504742712 | 1.035189888 | 0.015537701 | 0.067619589 |
| PGAP3 | -2.532523765 | 1.046676075 | 0.015538146 | 0.067619589 |
| PVR | -2.086167463 | 0.862277337 | 0.01554744 | 0.067632006 |
| CCDC176 | 2.277685455 | 0.941918827 | 0.015600353 | 0.067834081 |
| DHRS4L2 | -2.504568835 | 1.035870566 | 0.015612959 | 0.067860796 |
| KLF12 | -2.50144328 | 1.034710792 | 0.015626292 | 0.067890644 |
| CARKD | -1.907680692 | 0.789364984 | 0.015660702 | 0.068001846 |
| TPR | -1.893478111 | 0.783519369 | 0.015664838 | 0.068001846 |
| SF3B1 | -1.941283815 | 0.803479741 | 0.015687945 | 0.068074011 |
| MON1A | -2.507018851 | 1.03771499 | 0.015696238 | 0.068081866 |
| C21orf33 | -2.040585935 | 0.844743286 | 0.01570808 | 0.068105098 |
| ABHD6 | -2.52738528 | 1.046467068 | 0.015728304 | 0.068164639 |
| SLC4A7 | -2.203664104 | 0.912528892 | 0.015739621 | 0.068173599 |
| SLC16A6 | -2.499928362 | 1.035267893 | 0.015745378 | 0.068173599 |
| COL6A6 | -2.51960433 | 1.043460767 | 0.015749848 | 0.068173599 |
| OCLN | -2.50931922 | 1.039343259 | 0.01576411 | 0.06820722 |
| ACTR1A | -1.530002088 | 0.633769145 | 0.015772767 | 0.068216566 |
| PSMD8 | -1.707719704 | 0.707452376 | 0.015782768 | 0.06823172 |
| C10orf10 | -1.899844863 | 0.787473249 | 0.015839915 | 0.068450594 |
| GLG1 | -1.929647975 | 0.799941676 | 0.015855022 | 0.068487696 |
| SMPD2 | -2.503785136 | 1.038509483 | 0.01591142 | 0.068703053 |
| SRR | 1.88953745 | 0.783830156 | 0.015924273 | 0.068706373 |
| DOHH | -2.508971998 | 1.040797915 | 0.015925275 | 0.068706373 |
| GSTM2 | -2.527293164 | 1.048589773 | 0.015944524 | 0.068730276 |
| NIF3L1 | -2.494474222 | 1.035015477 | 0.015948849 | 0.068730276 |
| AKT3 | -1.928402383 | 0.800151247 | 0.015950451 | 0.068730276 |
| TEX10 | -2.180628754 | 0.90503394 | 0.01597684 | 0.06881575 |
| DOK1 | -2.498530968 | 1.037138008 | 0.01599353 | 0.068838812 |
| DAXX | -1.749413132 | 0.726192065 | 0.015995305 | 0.068838812 |
| AKNA | -1.929846208 | 0.801175512 | 0.016006455 | 0.068841596 |
| KIF21B | -2.409863942 | 1.000540857 | 0.016015538 | 0.068841596 |
| C12orf10 | -2.065245429 | 0.857460862 | 0.016015619 | 0.068841596 |
| CCHCR1 | -2.502797941 | 1.039220872 | 0.016025217 | 0.068854668 |
| APLN | -2.502204914 | 1.039055497 | 0.016033447 | 0.068861853 |
| DRD2 | 2.534222922 | 1.05310183 | 0.016108994 | 0.069155206 |
| DNASE1 | 2.534043257 | 1.053085974 | 0.016114921 | 0.069155206 |
| NTMT1 | -2.204736189 | 0.916330725 | 0.016126123 | 0.069175005 |
| HCAR3 | 2.203270242 | 0.915819048 | 0.016137445 | 0.069195307 |
| SDHAP2 | -2.331752493 | 0.969359583 | 0.016152266 | 0.069230592 |
| APTX | -2.068916419 | 0.860284311 | 0.016175931 | 0.069303732 |
| EPHA2 | -2.274373363 | 0.945809223 | 0.016186406 | 0.06932033 |
| BTG2 | -2.003730301 | 0.833332028 | 0.016195487 | 0.069330944 |
| IRGQ | -2.087591787 | 0.868775479 | 0.016265062 | 0.06957425 |
| CHST10 | -2.502105551 | 1.041365121 | 0.016273782 | 0.06957425 |
| PI4KB | -1.698552063 | 0.706960718 | 0.016278464 | 0.06957425 |
| C8orf42 | -2.008694559 | 0.836097653 | 0.016285035 | 0.06957425 |
| ERI1 | 1.694920938 | 0.705495471 | 0.01628545 | 0.06957425 |
| DHX57 | -2.49182508 | 1.037330254 | 0.016298929 | 0.069603517 |
| C19orf21 | -2.486581003 | 1.035221513 | 0.016306616 | 0.069608037 |
| HES1 | -1.889192658 | 0.786630875 | 0.016322415 | 0.069626857 |
| HLA-L | -2.385649593 | 0.993416143 | 0.016329773 | 0.069626857 |
| VIM | -1.549780545 | 0.64535606 | 0.016330916 | 0.069626857 |
| TRPM8 | 2.511950931 | 1.046362202 | 0.016365917 | 0.069747766 |
| NT5DC3 | -2.485466536 | 1.035580373 | 0.016391887 | 0.069830104 |
| AARS2 | -2.491935876 | 1.038348517 | 0.01639941 | 0.069833823 |
| NLN | 1.932860092 | 0.805474976 | 0.01641064 | 0.069853314 |
| ATP5D | -2.035622102 | 0.848390528 | 0.016422289 | 0.069855247 |
| SDF2L1 | -1.996127397 | 0.831946541 | 0.016424398 | 0.069855247 |
| LRRC42 | -2.207206384 | 0.91998169 | 0.016431605 | 0.069857606 |
| ATP6V0D1 | -1.904877272 | 0.794167988 | 0.016458678 | 0.069924437 |
| ATG16L2 | -1.968365121 | 0.820651851 | 0.016460643 | 0.069924437 |
| DAK | -2.501801711 | 1.043506204 | 0.016507572 | 0.070095434 |
| RXRA | -2.082421069 | 0.868728886 | 0.016525877 | 0.070144801 |
| RIPK3 | -2.48149361 | 1.035415645 | 0.016547259 | 0.070207178 |
| ZBTB14 | -2.488121506 | 1.038318002 | 0.016561526 | 0.07023576 |
| PADI4 | 2.394236409 | 0.999199678 | 0.016568123 | 0.07023576 |
| CTPS2 | -2.492285207 | 1.040200301 | 0.0165766 | 0.07023576 |
| C1orf189 | 2.485979198 | 1.037672138 | 0.016587438 | 0.07023576 |
| ZNF252P-AS1 | 2.485979198 | 1.037672138 | 0.016587438 | 0.07023576 |
| PACSIN2 | -1.695215706 | 0.707679518 | 0.016599671 | 0.070259227 |
| PCTP | -2.35052629 | 0.981493069 | 0.016627276 | 0.070347714 |
| VASH1 | -2.238110412 | 0.934619171 | 0.016635042 | 0.070352227 |
| TSC22D3 | -2.290251766 | 0.956452379 | 0.016641784 | 0.070352405 |
| COL1A1 | 1.643909294 | 0.686696651 | 0.01666856 | 0.070414371 |
| DUSP8 | -2.491611727 | 1.040812753 | 0.016669853 | 0.070414371 |
| GPX1 | -1.704340031 | 0.712000774 | 0.016677867 | 0.070419894 |
| GSN | -2.315929745 | 0.967951838 | 0.016729083 | 0.070594424 |
| FAT4 | -2.486458526 | 1.039305935 | 0.016737579 | 0.070594424 |
| MED24 | -2.021038239 | 0.844867144 | 0.016750573 | 0.070594424 |
| MAMDC2 | -2.49568177 | 1.043311574 | 0.016753321 | 0.070594424 |
| ZBED2 | -2.49300769 | 1.042258477 | 0.01676011 | 0.070594424 |
| VPS25 | -2.03921262 | 0.852577869 | 0.016765072 | 0.070594424 |
| TMEM201 | -2.480939289 | 1.03733522 | 0.016772982 | 0.070594424 |
| C17orf103 | -2.480939289 | 1.03733522 | 0.016772982 | 0.070594424 |
| C2orf42 | -2.476423528 | 1.035682542 | 0.016797844 | 0.070670738 |
| RBM15B | -1.93732792 | 0.81044294 | 0.01682748 | 0.070767069 |
| ATG14 | -2.478118786 | 1.036924574 | 0.016854167 | 0.070823565 |
| PTPDC1 | -2.475826005 | 1.036020093 | 0.016859978 | 0.070823565 |
| USHBP1 | -2.500537494 | 1.046371876 | 0.016861148 | 0.070823565 |
| CMTM2 | 2.474433617 | 1.035849321 | 0.016903645 | 0.070952809 |
| STAT6 | -1.439154463 | 0.602470886 | 0.016905639 | 0.070952809 |
| MRPS18A | -1.931936281 | 0.808811517 | 0.016912188 | 0.070952809 |
| PLEKHA5 | -2.480463335 | 1.038594203 | 0.016927015 | 0.070986656 |
| ABCD4 | -2.129143512 | 0.891579148 | 0.016937627 | 0.071002805 |
| HLCS | -2.476789556 | 1.037235377 | 0.016946054 | 0.071009783 |
| LCK | -2.48894578 | 1.043585459 | 0.017079368 | 0.071539865 |
| PLEKHG4 | -2.477840703 | 1.039003242 | 0.017087257 | 0.071544371 |
| U2AF1L4 | -2.333277923 | 0.978569653 | 0.017108116 | 0.071577364 |
| TNRC6C | -2.336284236 | 0.979836265 | 0.017108769 | 0.071577364 |
| LAMTOR4 | -2.070324652 | 0.868375106 | 0.017119258 | 0.071592115 |
| NDRG3 | -2.393216154 | 1.003868803 | 0.01712593 | 0.071592115 |
| PYROXD1 | -2.470766688 | 1.036583057 | 0.017145695 | 0.071646216 |
| CYB561D2 | -2.47304661 | 1.037623435 | 0.017154671 | 0.071655211 |
| SFTPB | 1.51770575 | 0.637094791 | 0.017208188 | 0.071829964 |
| B3GNT5 | 1.39579409 | 0.585929863 | 0.017210188 | 0.071829964 |
| LTBP1 | -1.756595603 | 0.737616249 | 0.017244671 | 0.071918196 |
| GCNT2 | -2.473047295 | 1.038466499 | 0.017245026 | 0.071918196 |
| YRDC | -2.080908988 | 0.87386573 | 0.017253129 | 0.071923423 |
| SLC8A1 | 1.956082655 | 0.821739942 | 0.017293118 | 0.072039569 |
| A1BG | -2.477323824 | 1.040725428 | 0.01729471 | 0.072039569 |
| KIFC2 | 2.287088248 | 0.960879677 | 0.017303125 | 0.07204604 |
| LENG8 | -1.819959374 | 0.764688844 | 0.017312643 | 0.072057099 |
| ZNF468 | -2.469094918 | 1.037520886 | 0.017321914 | 0.072058341 |
| TIFA | 1.712996438 | 0.719836672 | 0.017326665 | 0.072058341 |
| LRRC23 | -2.473015691 | 1.039643816 | 0.017373132 | 0.072222983 |
| PROK2 | 2.416752462 | 1.016083011 | 0.017383282 | 0.072236584 |
| PPDPF | 2.049584141 | 0.861838335 | 0.017399565 | 0.072236913 |
| LOC284023 | 1.915725019 | 0.805566893 | 0.017401732 | 0.072236913 |
| ZNF587 | -2.466814538 | 1.037364267 | 0.017408541 | 0.072236913 |
| IVNS1ABP | 1.577257699 | 0.663311502 | 0.017413734 | 0.072236913 |
| FCER2 | 2.50507445 | 1.053540091 | 0.017417757 | 0.072236913 |
| ZNF232 | -2.463249549 | 1.036255087 | 0.017450837 | 0.072345534 |
| ACOT8 | -2.466685277 | 1.037880052 | 0.017470307 | 0.072397671 |
| ARF5 | -2.06702059 | 0.87000365 | 0.017507356 | 0.072522583 |
| VNN2 | 2.22902281 | 0.938375765 | 0.017529688 | 0.072586455 |
| PLEKHO2 | -2.117685985 | 0.891564227 | 0.017537172 | 0.072588821 |
| PLEKHH2 | -2.163954734 | 0.911174657 | 0.01755339 | 0.072627323 |
| MIR143HG | -2.494980288 | 1.050718057 | 0.017570459 | 0.072669316 |
| PROSAPIP1 | -2.460590174 | 1.036386846 | 0.017586997 | 0.072709078 |
| CTBS | 1.776825552 | 0.748523779 | 0.017607371 | 0.072764659 |
| LRWD1 | -2.45904449 | 1.036394883 | 0.017659045 | 0.072895348 |
| REV1 | -1.898561367 | 0.800196088 | 0.017662417 | 0.072895348 |
| POGLUT1 | 1.536866689 | 0.647771658 | 0.017666064 | 0.072895348 |
| EIF4H | -1.729162425 | 0.728826581 | 0.017666761 | 0.072895348 |
| MAL | -2.479622794 | 1.045369939 | 0.017691848 | 0.072970187 |
| BRAT1 | -2.463355884 | 1.038631517 | 0.017704915 | 0.072993573 |
| CAMKK1 | -2.47501005 | 1.043605029 | 0.01771142 | 0.072993573 |
| NTHL1 | -2.484408228 | 1.047786202 | 0.017735124 | 0.073020277 |
| P2RY8 | -2.459774358 | 1.037419327 | 0.017737573 | 0.073020277 |
| OSBPL7 | -2.466482667 | 1.040259442 | 0.017738761 | 0.073020277 |
| AFAP1L1 | -2.186160509 | 0.92228689 | 0.017770327 | 0.073121552 |
| CBLB | -1.863849027 | 0.786426886 | 0.017787026 | 0.0731616 |
| MUC20 | -2.467612877 | 1.041301631 | 0.017800656 | 0.073188992 |
| PTPRD | -2.459472644 | 1.038090484 | 0.017825279 | 0.07326155 |
| RAB23 | 1.732904798 | 0.73155403 | 0.017845926 | 0.073317712 |
| ZBTB39 | -2.460935378 | 1.039098958 | 0.017868304 | 0.073380941 |
| FLT1 | -1.695194989 | 0.715829903 | 0.017877124 | 0.07338846 |
| RPL19 | -1.238300252 | 0.523146988 | 0.017931883 | 0.073584489 |
| ARIH2 | -1.66482831 | 0.703416412 | 0.017943843 | 0.073587056 |
| TBCE | -2.453345872 | 1.036601834 | 0.017946524 | 0.073587056 |
| CLK3 | -1.79223348 | 0.757382225 | 0.017964321 | 0.073631278 |
| NCS1 | -1.977713554 | 0.835826229 | 0.017972806 | 0.073637312 |
| CASP9 | -2.353441009 | 0.99471024 | 0.017983564 | 0.073652656 |
| ZNF320 | -2.457787697 | 1.039102385 | 0.018015526 | 0.07375479 |
| CRABP2 | -2.3044681 | 0.974658137 | 0.018059982 | 0.073907979 |
| KDM3A | -2.089262636 | 0.883797558 | 0.018080734 | 0.073960243 |
| STARD13 | -1.884385167 | 0.797172703 | 0.01808684 | 0.073960243 |
| SOGA1 | -2.463295735 | 1.042639328 | 0.018149308 | 0.074167775 |
| FAM92A1 | -2.451541986 | 1.037720162 | 0.018155535 | 0.074167775 |
| MIB1 | -2.031676013 | 0.860018021 | 0.01815878 | 0.074167775 |
| LOC654433 | -1.992449678 | 0.843499244 | 0.018170571 | 0.074184234 |
| LRRC25 | 1.741641396 | 0.73736065 | 0.018176939 | 0.074184234 |
| DEPDC5 | -2.448110637 | 1.036739377 | 0.018208247 | 0.074247559 |
| LOC388692 | -2.448875147 | 1.037100452 | 0.01821242 | 0.074247559 |
| TTLL4 | -2.229587393 | 0.944242075 | 0.018213666 | 0.074247559 |
| ITIH4 | -2.472327337 | 1.047317697 | 0.018244028 | 0.074339887 |
| RNF139 | -1.939278675 | 0.821629675 | 0.018260991 | 0.074339887 |
| BNIP1 | -2.306524063 | 0.977228994 | 0.018261649 | 0.074339887 |
| ADCK2 | -2.459947717 | 1.042258345 | 0.018264633 | 0.074339887 |
| MRGPRF | -2.466941448 | 1.045406134 | 0.018285165 | 0.07439462 |
| HIAT1 | 1.388814897 | 0.588673654 | 0.01831304 | 0.074479175 |
| CCT4 | -1.821120846 | 0.771990551 | 0.018324567 | 0.074497203 |
| DNAAF2 | -2.448432613 | 1.038195904 | 0.018356219 | 0.074597004 |
| C15orf57 | 1.459177443 | 0.619267469 | 0.018458204 | 0.074963106 |
| SH3D19 | -2.181163849 | 0.925693632 | 0.018460584 | 0.074963106 |
| PDGFRA | 1.620039879 | 0.68767872 | 0.018482406 | 0.07502271 |
| ADCY7 | 1.6612639 | 0.705261659 | 0.018496381 | 0.075050423 |
| PAK2 | 1.27376583 | 0.54079785 | 0.018505486 | 0.075058367 |
| CDC7 | -2.445114318 | 1.038337909 | 0.018530938 | 0.075103602 |
| EFNA1 | -1.898824538 | 0.806351492 | 0.018530943 | 0.075103602 |
| ERMAP | -2.447965815 | 1.039804336 | 0.018559814 | 0.075191591 |
| NDUFS7 | -2.159635597 | 0.917720161 | 0.018609538 | 0.075324511 |
| LOC729970 | -2.440307626 | 1.037063144 | 0.0186179 | 0.075324511 |
| RASSF7 | -2.442771659 | 1.03813305 | 0.018620483 | 0.075324511 |
| RRP36 | -1.969136279 | 0.83685263 | 0.018621315 | 0.075324511 |
| ATP2A3 | -2.187432854 | 0.92996272 | 0.018664111 | 0.075468551 |
| AKT2 | -2.032938302 | 0.864432638 | 0.018684815 | 0.075514932 |
| NTRK2 | -2.474545925 | 1.052255985 | 0.018689964 | 0.075514932 |
| LURAP1L | -2.320448895 | 0.986899401 | 0.018710373 | 0.075568316 |
| CLUH | -1.885580175 | 0.802120084 | 0.018735844 | 0.075606166 |
| KCNS3 | -2.381389896 | 1.013095374 | 0.018742777 | 0.075606166 |
| NBPF10 | -2.454665133 | 1.044321604 | 0.018748825 | 0.075606166 |
| NANP | -2.441838297 | 1.038899433 | 0.018752806 | 0.075606166 |
| HEYL | -2.395160582 | 1.019065302 | 0.018755743 | 0.075606166 |
| ARMC7 | -2.436555472 | 1.037374962 | 0.018835524 | 0.075881872 |
| ASPSCR1 | -2.332977437 | 0.993301765 | 0.018838591 | 0.075881872 |
| NMT1 | -1.643272836 | 0.699719985 | 0.018850613 | 0.075901185 |
| IP6K2 | -1.679662031 | 0.715336428 | 0.018870831 | 0.075953468 |
| TMEM74B | -2.46665172 | 1.051247576 | 0.018955539 | 0.076245517 |
| CPNE8 | 1.393385567 | 0.593850492 | 0.018957913 | 0.076245517 |
| DNAH1 | -2.3028971 | 0.981537616 | 0.018965225 | 0.076245723 |
| COL5A3 | -2.460387221 | 1.048761521 | 0.018976462 | 0.076261703 |
| HPS3 | -2.432724371 | 1.037522408 | 0.019040151 | 0.076486587 |
| TMEM218 | -2.438636214 | 1.040103112 | 0.019046988 | 0.076486587 |
| TBC1D10B | 1.483487296 | 0.63282298 | 0.019066032 | 0.076533791 |
| TSC2 | -2.230796439 | 0.952191058 | 0.019139466 | 0.07679921 |
| PREPL | -2.154231387 | 0.919748653 | 0.019170657 | 0.076894985 |
| CMPK2 | 2.355859371 | 1.006081889 | 0.01920036 | 0.076949761 |
| WDR81 | -2.441690693 | 1.042794101 | 0.019207003 | 0.076949761 |
| COMMD7 | -1.990117794 | 0.849959871 | 0.019210255 | 0.076949761 |
| TMEM2 | -1.954399387 | 0.834766773 | 0.019219189 | 0.076949761 |
| SLC52A2 | -2.151380775 | 0.918968685 | 0.019227959 | 0.076949761 |
| MTX3 | -2.431010006 | 1.038421474 | 0.019228926 | 0.076949761 |
| ACAA2 | -1.914300423 | 0.81777253 | 0.019238787 | 0.076949761 |
| NDUFV1 | -2.182719125 | 0.932470746 | 0.019242936 | 0.076949761 |
| OTUD4 | -1.888300478 | 0.806941593 | 0.019280083 | 0.077068764 |
| TNFRSF10B | -2.01743968 | 0.862179638 | 0.019287374 | 0.077068764 |
| PIGS | -1.906203745 | 0.814772618 | 0.019306831 | 0.077117165 |
| RPS9 | -1.579166133 | 0.675120304 | 0.019330854 | 0.077183764 |
| SBF1 | -2.158022237 | 0.923020053 | 0.019387172 | 0.077358089 |
| PCCB | -2.428419452 | 1.03877447 | 0.019399 | 0.077358089 |
| HAT1 | -1.75594988 | 0.751143388 | 0.019402679 | 0.077358089 |
| SCN7A | -2.452535961 | 1.049133715 | 0.019404 | 0.077358089 |
| RNF170 | -2.431189242 | 1.040065084 | 0.019411348 | 0.077358089 |
| CMAHP | -1.972230702 | 0.843947706 | 0.019443811 | 0.077458064 |
| KCTD18 | -2.429095089 | 1.039949903 | 0.019502665 | 0.077663059 |
| ADAMTSL3 | -2.448748636 | 1.048586488 | 0.01952852 | 0.07773654 |
| RNF20 | -2.081430633 | 0.891407115 | 0.019543713 | 0.077755333 |
| CD6 | -2.437306811 | 1.043854096 | 0.019548051 | 0.077755333 |
| PSMB4 | -1.492433638 | 0.639271573 | 0.019565126 | 0.077782536 |
| SUSD3 | 2.251317674 | 0.964369415 | 0.019569704 | 0.077782536 |
| ARPC2 | 1.221664707 | 0.523506537 | 0.01961569 | 0.077929095 |
| C20orf85 | 2.077077903 | 0.890117385 | 0.019622567 | 0.077929095 |
| WFDC2 | 1.750090605 | 0.750027697 | 0.019628841 | 0.077929095 |
| COL5A1 | 1.667272682 | 0.714610157 | 0.019641743 | 0.077950847 |
| CABLES1 | -2.271217577 | 0.973940893 | 0.019701371 | 0.07814759 |
| ELF1 | 1.438641142 | 0.616940717 | 0.019706201 | 0.07814759 |
| CXCL6 | 2.376414201 | 1.019175837 | 0.019716381 | 0.078158442 |
| XYLT2 | -2.267098875 | 0.972404565 | 0.019730388 | 0.078184456 |
| ZRANB1 | -1.928884703 | 0.827408364 | 0.019740885 | 0.07819654 |
| CXCL11 | 2.396780355 | 1.028331522 | 0.019766717 | 0.078269343 |
| AAMP | -1.525353738 | 0.654520122 | 0.019780176 | 0.078293113 |
| NDUFS8 | -1.927933327 | 0.827401611 | 0.019800558 | 0.0783414 |
| INO80 | -1.887813543 | 0.810255729 | 0.019811521 | 0.0783414 |
| CCDC68 | -2.351458492 | 1.009293352 | 0.019816366 | 0.0783414 |
| CPSF1 | -2.107099621 | 0.904452555 | 0.019822217 | 0.0783414 |
| WDR83 | -2.421754723 | 1.039735274 | 0.019848304 | 0.078414988 |
| GNAS | 1.440099726 | 0.618513951 | 0.019895004 | 0.078536905 |
| CAPN10 | -2.421558571 | 1.040079475 | 0.019899163 | 0.078536905 |
| HGS | -1.778838707 | 0.764041039 | 0.0199016 | 0.078536905 |
| MARK3 | -1.612724566 | 0.692814832 | 0.019923477 | 0.07855034 |
| NUAK1 | -2.358133617 | 1.013064339 | 0.019926793 | 0.07855034 |
| PIGU | -2.260083739 | 0.970946811 | 0.019927446 | 0.07855034 |
| LYVE1 | -2.230367115 | 0.958317189 | 0.019945113 | 0.07859048 |
| BTNL9 | -2.438596518 | 1.048170316 | 0.019990455 | 0.078739597 |
| ZNF621 | -2.138612541 | 0.91933855 | 0.020005146 | 0.078767918 |
| AHDC1 | -2.294571156 | 0.986459187 | 0.020014923 | 0.078776876 |
| TMEM126B | 1.470208168 | 0.632523485 | 0.020106556 | 0.079107886 |
| PNN | -1.640513992 | 0.705921239 | 0.02012906 | 0.079166764 |
| ACTR3B | -2.41530094 | 1.039445389 | 0.020144587 | 0.07919817 |
| KCTD7 | -2.254393046 | 0.970272073 | 0.020154194 | 0.079206286 |
| TRIQK | -2.426545487 | 1.044874468 | 0.020215062 | 0.079415774 |
| RNPEP | -1.996164516 | 0.860048896 | 0.020287407 | 0.07967018 |
| NPLOC4 | -1.55081803 | 0.668370407 | 0.020324802 | 0.079787194 |
| VLDLR | -2.417903573 | 1.042563823 | 0.020384737 | 0.079992574 |
| ANO6 | -1.972711539 | 0.850710868 | 0.02040058 | 0.080024837 |
| CHERP | -1.874179981 | 0.808385474 | 0.020426309 | 0.080095846 |
| FGG | 2.174545684 | 0.93802434 | 0.020437431 | 0.080109543 |
| SFRP2 | -2.4420206 | 1.053519687 | 0.020451283 | 0.080119412 |
| GOLGA8B | -1.941439714 | 0.837588627 | 0.020455208 | 0.080119412 |
| RNF213 | 1.634478151 | 0.705211731 | 0.020464943 | 0.080127653 |
| IMMP2L | -2.408997977 | 1.039809202 | 0.020516305 | 0.080298816 |
| N4BP3 | -2.415908783 | 1.042932765 | 0.020533336 | 0.08033553 |
| PPP1R14A | -2.394109612 | 1.033678579 | 0.020552458 | 0.080380395 |
| RNF175 | 2.158145283 | 0.931897975 | 0.020565912 | 0.080403067 |
| CHMP5 | 1.746542984 | 0.754341152 | 0.020595294 | 0.080487971 |
| SH3RF3 | -1.704014649 | 0.736255753 | 0.02064398 | 0.080642846 |
| SRSF9 | -1.820510623 | 0.7866294 | 0.020650282 | 0.080642846 |
| DUS2L | -2.406789557 | 1.040154964 | 0.020674528 | 0.080707514 |
| CCDC14 | -2.063181832 | 0.891884141 | 0.020706998 | 0.080804231 |
| HBS1L | -1.911114025 | 0.826203897 | 0.020715692 | 0.080808128 |
| PKN3 | -2.357695183 | 1.01939457 | 0.020731511 | 0.080839805 |
| NPTX1 | -2.425574021 | 1.049168702 | 0.020783148 | 0.081011074 |
| CIDEB | -2.062526183 | 0.892203781 | 0.020793065 | 0.081019659 |
| NR5A2 | -2.412308622 | 1.044574804 | 0.020923116 | 0.081496159 |
| GPATCH4 | -1.810939473 | 0.784279834 | 0.02094094 | 0.081521986 |
| ABCB10 | -2.404923958 | 1.041557264 | 0.020945274 | 0.081521986 |
| TRPS1 | -2.403661907 | 1.041301613 | 0.020981101 | 0.081631175 |
| PMS1 | -2.400481663 | 1.04010532 | 0.021003491 | 0.08168802 |
| C2CD4A | 1.868338618 | 0.810006267 | 0.021078634 | 0.081926237 |
| GOSR2 | -1.937947508 | 0.840195918 | 0.021080344 | 0.081926237 |
| ZNF518A | -2.400207922 | 1.040736962 | 0.021096222 | 0.081957611 |
| PRR13 | 1.359528326 | 0.589658088 | 0.021131795 | 0.082065449 |
| YPEL1 | -2.397524267 | 1.039953171 | 0.021143308 | 0.082079808 |
| AP2A2 | -1.807554395 | 0.784181332 | 0.0211654 | 0.082135207 |
| METTL9 | 1.404935424 | 0.609632139 | 0.021191083 | 0.082204492 |
| SEC22C | -2.024467203 | 0.878631112 | 0.021216184 | 0.082271472 |
| SPAG7 | -1.661572113 | 0.721377655 | 0.02126018 | 0.082411647 |
| APOBR | 1.830267843 | 0.794712307 | 0.021275629 | 0.0824411 |
| MVB12B | -2.402065314 | 1.043056347 | 0.021283885 | 0.082442668 |
| CYTH2 | -1.81818294 | 0.789584266 | 0.021295211 | 0.082456126 |
| SOD2 | 1.940979716 | 0.843067245 | 0.021319196 | 0.082518568 |
| ILF3 | -2.015493541 | 0.875697612 | 0.021358519 | 0.082640312 |
| ITK | -2.405372599 | 1.045188082 | 0.021370288 | 0.082655395 |
| ZNF688 | -2.404897448 | 1.045391856 | 0.021421345 | 0.082814159 |
| NAP1L5 | -2.404400343 | 1.045222039 | 0.021427109 | 0.082814159 |
| MTDH | -1.812815802 | 0.788237002 | 0.021457515 | 0.082855911 |
| FBXO9 | -2.031697226 | 0.883435915 | 0.021461394 | 0.082855911 |
| RNASEH1 | -2.255746627 | 0.980909579 | 0.021468178 | 0.082855911 |
| PSMA7 | -1.369953477 | 0.59575324 | 0.021474764 | 0.082855911 |
| SFXN5 | -2.398576542 | 1.043092519 | 0.021477363 | 0.082855911 |
| MCCC1 | -2.115884232 | 0.920559611 | 0.021534709 | 0.083046631 |
| ZBTB18 | -2.255868226 | 0.981685549 | 0.021564408 | 0.083130634 |
| TMEM8A | -1.90760976 | 0.830203121 | 0.021575299 | 0.083142097 |
| DCAF8 | -1.842416922 | 0.802097439 | 0.021618833 | 0.083279299 |
| CCDC69 | -1.965158432 | 0.855662414 | 0.021638654 | 0.083325084 |
| GDPD3 | -2.388837916 | 1.04025446 | 0.021653154 | 0.083350356 |
| SMG8 | -2.391966768 | 1.041916419 | 0.021690885 | 0.083465001 |
| COX10-AS1 | 2.177359531 | 0.948665569 | 0.021722733 | 0.083541425 |
| GAL | -2.417707192 | 1.053415386 | 0.021726658 | 0.083541425 |
| CLDN11 | -2.399232215 | 1.04544176 | 0.021736228 | 0.083547634 |
| HSD17B8 | -2.392847361 | 1.042888636 | 0.02176513 | 0.083599537 |
| DCAF10 | 1.725876536 | 0.752201842 | 0.021765654 | 0.083599537 |
| LRP5L | -2.388389233 | 1.041026663 | 0.021775381 | 0.083606317 |
| HBB | 2.244580196 | 0.978456874 | 0.021790491 | 0.083633754 |
| PDE4C | -2.396140456 | 1.044599394 | 0.021799879 | 0.083639214 |
| TLR4 | 1.917619003 | 0.836125124 | 0.021821572 | 0.083691864 |
| SLIT3 | -2.165028191 | 0.944089716 | 0.021833954 | 0.083708782 |
| GRB7 | -2.404692863 | 1.049220256 | 0.021912242 | 0.083978266 |
| NAIF1 | -2.390249861 | 1.043181776 | 0.021945655 | 0.084075639 |
| CCPG1 | 1.969321228 | 0.860105999 | 0.022042995 | 0.084417758 |
| ESRRA | -1.969368231 | 0.860339297 | 0.022075869 | 0.084512833 |
| LGALS3BP | 1.549809599 | 0.677144659 | 0.022094332 | 0.084530915 |
| RBM28 | -1.945443469 | 0.850020557 | 0.022096692 | 0.084530915 |
| ZFP90 | -2.24224115 | 0.979785691 | 0.022108325 | 0.084544619 |
| CYP2S1 | 1.796631804 | 0.785313293 | 0.02214976 | 0.084672237 |
| TMEM100 | -2.332669504 | 1.020292438 | 0.022238152 | 0.084979197 |
| RABIF | 1.515458299 | 0.663007807 | 0.02226993 | 0.085063812 |
| SLC35C2 | -1.869781249 | 0.818063022 | 0.022276496 | 0.085063812 |
| CA4 | -2.405243787 | 1.052459313 | 0.022291985 | 0.085092016 |
| REXO4 | -1.919431194 | 0.839937347 | 0.022300659 | 0.085094193 |
| BAHD1 | -1.861116699 | 0.814560348 | 0.022323894 | 0.085151911 |
| PPFIA1 | -2.023153401 | 0.885725965 | 0.022361229 | 0.085223825 |
| CSNK2A2 | -2.2292648 | 0.975971948 | 0.022362809 | 0.085223825 |
| KIFC3 | -1.727928055 | 0.756510922 | 0.022367095 | 0.085223825 |
| MROH1 | -2.246444665 | 0.983621821 | 0.02238035 | 0.0852434 |
| MRPL48 | -2.377677522 | 1.041224491 | 0.022398594 | 0.085281955 |
| MED15 | -1.564171196 | 0.685090164 | 0.022420882 | 0.085335876 |
| DYM | -2.133627011 | 0.93487127 | 0.022473508 | 0.085500199 |
| SLC44A2 | -2.165101565 | 0.948721653 | 0.022481951 | 0.085500199 |
| KCNN3 | -2.3756023 | 1.041029261 | 0.022490842 | 0.085500199 |
| CBFA2T3 | -2.39464557 | 1.049419379 | 0.022496624 | 0.085500199 |
| ACN9 | 1.975694126 | 0.866210119 | 0.022557408 | 0.085700196 |
| TMEM205 | -1.62787745 | 0.713832498 | 0.022579508 | 0.085743718 |
| PER2 | -2.036277533 | 0.892955576 | 0.022585194 | 0.085743718 |
| SCRN2 | -2.351766719 | 1.031584718 | 0.022621861 | 0.085851883 |
| EIF6 | -1.55636679 | 0.68290782 | 0.022665336 | 0.085960168 |
| IL11RA | -2.389392445 | 1.048475144 | 0.022671738 | 0.085960168 |
| H3F3B | 1.507803692 | 0.661646096 | 0.022674951 | 0.085960168 |
| ARHGEF9 | -2.385214632 | 1.046750267 | 0.022685769 | 0.08597014 |
| MS4A4A | 1.772792726 | 0.778036217 | 0.022693959 | 0.085970152 |
| GPR68 | -2.153079168 | 0.945101049 | 0.022717818 | 0.086012388 |
| IQCK | 1.800136654 | 0.790211847 | 0.022724009 | 0.086012388 |
| FAM167A | -2.374051117 | 1.042188495 | 0.022729681 | 0.086012388 |
| POLR2J3 | -2.385502144 | 1.047400025 | 0.022753618 | 0.086071953 |
| SNTA1 | -2.380721403 | 1.045654835 | 0.02279963 | 0.08621495 |
| PRPF31 | -1.936701617 | 0.850762502 | 0.022820262 | 0.086242365 |
| LZTS1 | -2.394482718 | 1.051895635 | 0.022825065 | 0.086242365 |
| RNF149 | 1.955237518 | 0.858976049 | 0.022831518 | 0.086242365 |
| NR2C2AP | -2.253675345 | 0.990163735 | 0.022842221 | 0.086251766 |
| C8orf82 | 1.980769635 | 0.870463284 | 0.022873858 | 0.08634018 |
| OBSCN | -2.344622835 | 1.030506951 | 0.022893154 | 0.086381966 |
| CNRIP1 | -1.787829662 | 0.78602463 | 0.02293467 | 0.086507531 |
| C10orf118 | -2.369008979 | 1.041753839 | 0.022962481 | 0.086581331 |
| LOC253039 | -2.386518904 | 1.050028783 | 0.023037461 | 0.08683287 |
| GBGT1 | -2.373604879 | 1.044516441 | 0.023059721 | 0.086885585 |
| HNRNPUL1 | 1.407166917 | 0.619324549 | 0.023080509 | 0.086932721 |
| ADCYAP1R1 | -2.375355935 | 1.045879293 | 0.023137484 | 0.087116069 |
| TMEM45B | -2.390651404 | 1.052890753 | 0.02317364 | 0.087192143 |
| DMKN | 1.547068832 | 0.681363337 | 0.023174295 | 0.087192143 |
| PLA2G16 | -1.960526649 | 0.863742148 | 0.023219389 | 0.087330516 |
| EEF1G | -1.567957002 | 0.690875545 | 0.023236623 | 0.087348743 |
| SLC5A6 | -1.905325704 | 0.839553316 | 0.023240871 | 0.087348743 |
| CRTC3 | -1.74268753 | 0.768493202 | 0.023349443 | 0.087697684 |
| SOX13 | -2.194817519 | 0.967881041 | 0.023350417 | 0.087697684 |
| FAM110D | -2.386507809 | 1.052841937 | 0.023406759 | 0.087844854 |
| SERP1 | 1.688575338 | 0.744979431 | 0.023414265 | 0.087844854 |
| DDX31 | -2.169631576 | 0.957218736 | 0.023414698 | 0.087844854 |
| GOLIM4 | -2.217169297 | 0.978904686 | 0.023515803 | 0.0881681 |
| RFNG | -2.362732071 | 1.043186122 | 0.02351765 | 0.0881681 |
| FAM124A | -2.373804909 | 1.048182307 | 0.02353189 | 0.088176071 |
| CDC42BPA | -1.827715327 | 0.807076998 | 0.02353657 | 0.088176071 |
| SAMD14 | -2.367815056 | 1.045967665 | 0.023589157 | 0.08834156 |
| ILF3-AS1 | -2.373612895 | 1.048887822 | 0.023636879 | 0.088474329 |
| KIF17 | -2.100412046 | 0.928192188 | 0.02364146 | 0.088474329 |
| USE1 | -1.955132445 | 0.864114921 | 0.023661356 | 0.088517243 |
| GUCY1A3 | -2.337270923 | 1.033875449 | 0.023778518 | 0.088916266 |
| TFPI2 | -2.358985165 | 1.043528521 | 0.023784953 | 0.088916266 |
| PPIH | -2.145774872 | 0.949294866 | 0.023797171 | 0.088930282 |
| ZNF521 | -2.360554235 | 1.044380571 | 0.023806152 | 0.088932196 |
| GLCE | -2.359300561 | 1.043965282 | 0.023824876 | 0.088970492 |
| NEURL1B | -2.224415615 | 0.984629649 | 0.023874716 | 0.089124918 |
| C6orf47 | -1.873091696 | 0.829379731 | 0.023919192 | 0.089259218 |
| PRADC1 | -2.36376278 | 1.047514237 | 0.024036534 | 0.089665239 |
| ZW10 | -2.214413446 | 0.981412454 | 0.0240485 | 0.089678018 |
| PEAK1 | -1.876989616 | 0.831989073 | 0.024068955 | 0.089722434 |
| SYNGAP1 | 1.54237972 | 0.683786634 | 0.024092889 | 0.089779784 |
| WDR78 | 2.032987891 | 0.901706559 | 0.024158428 | 0.089992072 |
| SDE2 | -1.950728533 | 0.8653117 | 0.024173206 | 0.090015191 |
| USP37 | -2.069014192 | 0.918045269 | 0.02421398 | 0.090135059 |
| TMEM171 | 2.369182971 | 1.051364388 | 0.024231635 | 0.090168817 |
| ATRIP | -2.351282687 | 1.043500412 | 0.024242461 | 0.090177148 |
| FLJ10038 | -2.349424076 | 1.042787818 | 0.024257751 | 0.090202069 |
| PSMB5 | -1.592732465 | 0.707043312 | 0.024280213 | 0.090253634 |
| BICD2 | -1.819014275 | 0.807590196 | 0.024297153 | 0.090284643 |
| GPIHBP1 | -2.370327204 | 1.052484583 | 0.024314352 | 0.090309554 |
| C9orf142 | -2.081274305 | 0.92418141 | 0.024321057 | 0.090309554 |
| NPL | 1.500585749 | 0.666713705 | 0.024403289 | 0.090564075 |
| UCHL5 | -1.994369946 | 0.886125284 | 0.02440685 | 0.090564075 |
| CCDC113 | 2.051361658 | 0.911571596 | 0.024426283 | 0.090604166 |
| CDC42EP1 | -1.838638221 | 0.817599103 | 0.024523561 | 0.090932878 |
| PLA2G7 | 2.13879924 | 0.951232226 | 0.024547433 | 0.090989269 |
| MYL12A | 1.517938321 | 0.675273107 | 0.024583332 | 0.091086358 |
| TSPAN33 | -2.345387575 | 1.043429467 | 0.024590975 | 0.091086358 |
| IRF4 | -2.352228018 | 1.046747251 | 0.024628616 | 0.091149916 |
| PARVG | 1.49378122 | 0.664748343 | 0.024631213 | 0.091149916 |
| DPYSL3 | -2.2316544 | 0.993130145 | 0.024634174 | 0.091149916 |
| HSPA1A | 1.569220683 | 0.698513056 | 0.024670983 | 0.091253958 |
| TMEM54 | -2.24164977 | 0.997981878 | 0.024692299 | 0.091300645 |
| ZDHHC12 | -2.350659349 | 1.046705916 | 0.024718829 | 0.091330039 |
| S100A14 | -1.921213331 | 0.855546499 | 0.024729782 | 0.091330039 |
| ANKS1A | -2.076545063 | 0.924828913 | 0.024747028 | 0.091330039 |
| SLC39A1 | -1.504197564 | 0.669938625 | 0.024750377 | 0.091330039 |
| PABPN1 | -2.3417595 | 1.042984356 | 0.024752157 | 0.091330039 |
| FTO | 1.628256051 | 0.725202067 | 0.024752432 | 0.091330039 |
| ZNF821 | -2.343029913 | 1.043644493 | 0.024765179 | 0.091344974 |
| KBTBD11 | -2.343646124 | 1.043999588 | 0.024776309 | 0.09135394 |
| LSM1 | 1.404535087 | 0.625963535 | 0.024845467 | 0.091552976 |
| SNF8 | -1.811481328 | 0.807341168 | 0.024847727 | 0.091552976 |
| ABCG2 | -2.351099068 | 1.04794279 | 0.024862167 | 0.09157405 |
| LRP5 | -2.177675087 | 0.970907032 | 0.024901423 | 0.091686482 |
| SGIP1 | -1.864456168 | 0.83131301 | 0.024910696 | 0.091688476 |
| FGF2 | -1.990931357 | 0.887825472 | 0.024930331 | 0.091698344 |
| ITGB1BP1 | -1.751175451 | 0.780912741 | 0.024930842 | 0.091698344 |
| TARS2 | -2.342784532 | 1.04482987 | 0.024944307 | 0.091715745 |
| GTF2H3 | -2.338586369 | 1.043312962 | 0.024993683 | 0.091858906 |
| IFT172 | -2.346129527 | 1.046729104 | 0.025000738 | 0.091858906 |
| RCE1 | -1.998015798 | 0.891656626 | 0.025039641 | 0.091969665 |
| FBXL5 | 1.674714389 | 0.747453024 | 0.0250545 | 0.091979311 |
| SLC25A19 | -2.337246956 | 1.043190335 | 0.025059786 | 0.091979311 |
| RPE | -2.204305292 | 0.984016185 | 0.02508373 | 0.092035028 |
| BRWD1 | -1.819653642 | 0.812678106 | 0.025150523 | 0.092247868 |
| SERINC4 | -2.336444582 | 1.043600541 | 0.025166956 | 0.092275908 |
| APBB1 | -1.966711941 | 0.878561736 | 0.02518465 | 0.092308554 |
| SLC25A30 | -2.189357731 | 0.978175446 | 0.025207653 | 0.092333291 |
| HSF1 | -1.897720367 | 0.847883553 | 0.025208984 | 0.092333291 |
| MZB1 | -2.353695579 | 1.052239324 | 0.025296515 | 0.092587306 |
| SLC35E1 | -1.96677689 | 0.879279734 | 0.025299119 | 0.092587306 |
| NEDD9 | -1.319230804 | 0.589830808 | 0.025310807 | 0.092587306 |
| MAPK1 | -1.488969549 | 0.665734076 | 0.025313604 | 0.092587306 |
| PRKCE | -1.915975365 | 0.85702322 | 0.025376894 | 0.092750624 |
| TMCO4 | -2.332869442 | 1.043545317 | 0.025383041 | 0.092750624 |
| SPRYD4 | 2.032577339 | 0.909228483 | 0.025384753 | 0.092750624 |
| SGPP2 | 2.204092255 | 0.986104677 | 0.025407477 | 0.092778115 |
| KCNQ1 | -2.332087423 | 1.043419815 | 0.025414572 | 0.092778115 |
| C9orf171 | 2.350765599 | 1.051806967 | 0.025418783 | 0.092778115 |
| RPS6KC1 | 1.546209744 | 0.691909949 | 0.025437185 | 0.092813024 |
| ITM2C | -2.206610709 | 0.987648835 | 0.025469538 | 0.092898791 |
| DTHD1 | 2.144830919 | 0.960116008 | 0.02548775 | 0.092932939 |
| FAM3C | -1.799944285 | 0.80578997 | 0.025498633 | 0.09294035 |
| COQ4 | -2.179291788 | 0.977166004 | 0.025733071 | 0.093743557 |
| BLOC1S6 | -1.617102379 | 0.725170069 | 0.025749903 | 0.093743557 |
| ZC3H12A | 1.772158374 | 0.794719876 | 0.025753036 | 0.093743557 |
| TECR | -1.965315165 | 0.881350479 | 0.025754705 | 0.093743557 |
| FRMD6 | -2.228478938 | 0.999440898 | 0.025765666 | 0.093750958 |
| FBP1 | -2.210124588 | 0.992103834 | 0.025899526 | 0.094200522 |
| COPZ2 | -2.344937722 | 1.052708511 | 0.025912004 | 0.094200522 |
| TMEM134 | -2.158921397 | 0.969227457 | 0.025916133 | 0.094200522 |
| C20orf96 | 1.972588042 | 0.88576163 | 0.025947508 | 0.094281933 |
| LOC100129034 | -1.782070436 | 0.800357651 | 0.025974513 | 0.094347409 |
| CRYBG3 | -2.327262853 | 1.045347752 | 0.025993763 | 0.094384686 |
| GUCY1A2 | -2.338094274 | 1.050636775 | 0.026053938 | 0.094570483 |
| HSPB2 | -2.333863415 | 1.048898352 | 0.026077105 | 0.094621866 |
| MRPL49 | -1.811604914 | 0.814446916 | 0.026125737 | 0.094765585 |
| SRSF7 | -1.419070165 | 0.638227871 | 0.026185217 | 0.094948538 |
| SLCO2B1 | -2.105659429 | 0.947500965 | 0.02626104 | 0.095190604 |
| FGD4 | 1.811903623 | 0.815377123 | 0.026272064 | 0.095197704 |
| MRPL23 | -1.874389146 | 0.843671579 | 0.026303266 | 0.095277888 |
| MYD88 | 1.519897525 | 0.684234158 | 0.026329839 | 0.095341254 |
| ICOSLG | -2.201791857 | 0.992031806 | 0.026454285 | 0.095758857 |
| TRIB1 | -1.358880841 | 0.612359534 | 0.026480611 | 0.095821122 |
| MCOLN1 | -1.981941573 | 0.893701555 | 0.026576849 | 0.096136236 |
| SCGB1A1 | 2.044827232 | 0.92213924 | 0.026590167 | 0.096147897 |
| SWAP70 | -1.847211615 | 0.833067342 | 0.026598385 | 0.096147897 |
| RPL10A | -1.36779583 | 0.616991383 | 0.026631296 | 0.096233736 |
| PITPNM2 | -2.031781985 | 0.916637002 | 0.026653094 | 0.096279372 |
| C11orf75 | 1.532543453 | 0.691531441 | 0.026680632 | 0.096345707 |
| DCAF11 | -1.866654047 | 0.842360421 | 0.026692855 | 0.096356712 |
| CCNB1 | 1.807572036 | 0.815908997 | 0.026732013 | 0.096461722 |
| COQ2 | 2.02643323 | 0.914749424 | 0.026740317 | 0.096461722 |
| SLC25A28 | 1.390044 | 0.627523603 | 0.026751401 | 0.096468564 |
| JAG2 | -2.330285137 | 1.052109033 | 0.026768962 | 0.096498755 |
| RPH3AL | -2.318356961 | 1.046949946 | 0.026801866 | 0.096575263 |
| TMED9 | -1.603624982 | 0.72421511 | 0.026808579 | 0.096575263 |
| LZTS2 | -1.837640708 | 0.830241899 | 0.026871467 | 0.096750551 |
| SLC38A2 | -1.364011583 | 0.6163186 | 0.026886627 | 0.096750551 |
| ELK1 | -1.782715463 | 0.805543181 | 0.026893503 | 0.096750551 |
| DPM1 | -1.850739772 | 0.836302516 | 0.026897456 | 0.096750551 |
| TMEM163 | -2.313817407 | 1.045652172 | 0.026911551 | 0.096750551 |
| AMT | -2.323152785 | 1.049927707 | 0.026919798 | 0.096750551 |
| ARNTL | -2.073859125 | 0.93727359 | 0.026921732 | 0.096750551 |
| SNORA54 | 2.323916893 | 1.050436395 | 0.026943547 | 0.096768514 |
| COA5 | -1.996647712 | 0.902677264 | 0.02697236 | 0.096768514 |
| AHRR | -2.313938638 | 1.046144163 | 0.026975401 | 0.096768514 |
| HS6ST1 | -2.191163925 | 0.990724929 | 0.026988964 | 0.096768514 |
| MFSD4 | -2.317971353 | 1.048061958 | 0.0269892 | 0.096768514 |
| PTH1R | -2.3294743 | 1.053276448 | 0.026991157 | 0.096768514 |
| CDK13 | -1.866110201 | 0.843765888 | 0.026991237 | 0.096768514 |
| SLU7 | -1.737116052 | 0.785536312 | 0.027009819 | 0.09679914 |
| ZNF595 | 1.940429211 | 0.87760767 | 0.027032787 | 0.09679914 |
| MLKL | 1.559543158 | 0.705358625 | 0.027036304 | 0.09679914 |
| KLF9 | -1.724469106 | 0.78003632 | 0.027052815 | 0.09679914 |
| TAF11 | -1.773726518 | 0.802321444 | 0.027053639 | 0.09679914 |
| SNRNP70 | -1.970866772 | 0.891503631 | 0.027055088 | 0.09679914 |
| SYNM | -2.108109907 | 0.953990923 | 0.027120446 | 0.096929071 |
| PRKRIR | -1.762289951 | 0.7975075 | 0.027122713 | 0.096929071 |
| STK17B | 1.371567058 | 0.620706488 | 0.027126893 | 0.096929071 |
| TPPP | -2.319431578 | 1.0497071 | 0.027133017 | 0.096929071 |
| ECM1 | -1.825876197 | 0.826384008 | 0.027141502 | 0.096929071 |
| MMP10 | -2.327432216 | 1.053438927 | 0.027149189 | 0.096929071 |
| TP53TG1 | -2.316164947 | 1.048495957 | 0.027172161 | 0.096929071 |
| TRMT10A | 1.995829128 | 0.903492994 | 0.027173632 | 0.096929071 |
| UBE2Z | -1.708598902 | 0.773476295 | 0.027175561 | 0.096929071 |
| CDCA7 | 2.323374176 | 1.05183851 | 0.027183708 | 0.096929071 |
| ZBED4 | -2.100178018 | 0.950959556 | 0.027210621 | 0.096988627 |
| CSNK1A1 | -1.607079123 | 0.72772371 | 0.027218882 | 0.096988627 |
| TLR8 | 1.96985821 | 0.892070409 | 0.027231246 | 0.096999766 |
| MAGI1 | -1.84196518 | 0.834214415 | 0.027242623 | 0.097007386 |
| PPP1R12C | -1.982016712 | 0.897739568 | 0.027259205 | 0.09703353 |
| HMOX2 | -1.502440174 | 0.680626925 | 0.027283635 | 0.097087579 |
| GYPC | -1.482705585 | 0.671924794 | 0.027338236 | 0.097248922 |
| MLYCD | -2.303913441 | 1.044918614 | 0.027462967 | 0.097659539 |
| ACE2 | 2.246210747 | 1.018878978 | 0.027482855 | 0.097669543 |
| VPS51 | -2.060905602 | 0.934833971 | 0.027484382 | 0.097669543 |
| RIN3 | -2.004243878 | 0.909253399 | 0.027505061 | 0.097709961 |
| TRIM65 | -2.3043735 | 1.045518878 | 0.027520988 | 0.097733479 |
| CCK | -2.318776854 | 1.05211726 | 0.027530333 | 0.097733613 |
| MLL | -1.954600562 | 0.887000581 | 0.027552005 | 0.097759709 |
| PARN | -2.142712983 | 0.972393217 | 0.027556303 | 0.097759709 |
| APLP2 | 1.436481928 | 0.652152558 | 0.02761747 | 0.097943617 |
| FABP6 | 2.316793706 | 1.052022292 | 0.027649156 | 0.097995521 |
| ECM2 | -2.313273551 | 1.050434738 | 0.027650769 | 0.097995521 |
| RASEF | -2.306511967 | 1.047445468 | 0.02766281 | 0.098005119 |
| LILRB5 | 1.932861762 | 0.877867699 | 0.02768164 | 0.098038753 |
| GINM1 | -1.791177034 | 0.813800881 | 0.027735911 | 0.098197843 |
| PARD6B | -2.299817197 | 1.044962553 | 0.027745875 | 0.09820001 |
| SHC3 | -2.300920225 | 1.04571768 | 0.027783745 | 0.098271315 |
| SERTAD2 | -1.906057104 | 0.866266583 | 0.027784738 | 0.098271315 |
| ZER1 | -2.100285576 | 0.954868074 | 0.027838429 | 0.098428061 |
| RNF4 | -1.745404707 | 0.793858032 | 0.027903828 | 0.098626084 |
| TXNDC12 | 1.270067217 | 0.577703742 | 0.027915276 | 0.09863335 |
| KIAA0947 | -1.820008782 | 0.827979516 | 0.027939657 | 0.098686288 |
| NCF1C | 2.112129498 | 0.961038661 | 0.027966423 | 0.098740399 |
| ORC4 | -2.032837085 | 0.925117911 | 0.027993204 | 0.098740399 |
| HYAL2 | -2.210854824 | 1.006169012 | 0.027999052 | 0.098740399 |
| ENDOV | -2.298328177 | 1.045985315 | 0.028000076 | 0.098740399 |
| CYSLTR1 | 2.125055743 | 0.967139626 | 0.028001991 | 0.098740399 |
| INSIG2 | -2.010442888 | 0.915158978 | 0.028033043 | 0.098790142 |
| WDR43 | -1.689755262 | 0.76924525 | 0.028046132 | 0.098790142 |
| SSRP1 | -1.784384584 | 0.812386666 | 0.028058176 | 0.098790142 |
| APOO | 1.627416128 | 0.740954582 | 0.02806492 | 0.098790142 |
| ZNF600 | -2.301169431 | 1.04780257 | 0.028078626 | 0.098790142 |
| HLA-DPA1 | 1.539996947 | 0.701226065 | 0.028081278 | 0.098790142 |
| PRRC2B | -1.470111434 | 0.66948144 | 0.028099407 | 0.098790142 |
| PTP4A3 | -2.169542259 | 0.988000452 | 0.028099677 | 0.098790142 |
| SYNE2 | -1.723994659 | 0.785126568 | 0.028105008 | 0.098790142 |
| AGAP11 | -2.305468777 | 1.05000949 | 0.028115951 | 0.098790142 |
| SNAP23 | -1.739390846 | 0.792211579 | 0.028119582 | 0.098790142 |
| C11orf73 | 1.401946748 | 0.638638914 | 0.028148513 | 0.098858707 |
| PARP11 | 2.026324217 | 0.923187746 | 0.028169261 | 0.0988985 |
| IL21R | 1.82117398 | 0.829847266 | 0.028193068 | 0.098948998 |
| EPN1 | 1.45695652 | 0.664293931 | 0.028290038 | 0.099243306 |
| CD248 | -2.304343002 | 1.050695156 | 0.028295825 | 0.099243306 |
| PAQR9 | 2.275277327 | 1.037595029 | 0.028319086 | 0.099259706 |
| GPR107 | -1.590625633 | 0.725374689 | 0.028319406 | 0.099259706 |
| TMOD2 | -2.2977312 | 1.04836885 | 0.028399715 | 0.099498772 |
| SOCS4 | -1.843871183 | 0.84132587 | 0.028406563 | 0.099498772 |
| ARPC5 | 1.306567526 | 0.596327063 | 0.028449981 | 0.099617621 |
| IL17RC | -2.293457491 | 1.047170443 | 0.028513558 | 0.099806956 |
| PDXDC1 | -1.77975009 | 0.812878164 | 0.028564685 | 0.099952603 |
| S1PR4 | -2.289600405 | 1.045855324 | 0.028581339 | 0.099977562 |
| GTPBP4 | -1.922193115 | 0.878287647 | 0.028628181 | 0.100108069 |
| CYB5D2 | -2.297723508 | 1.050350372 | 0.028700346 | 0.100327008 |
| RMND5A | -1.829270414 | 0.836470782 | 0.028750483 | 0.100468826 |
| LSM3 | 1.666439825 | 0.762236699 | 0.028797335 | 0.100545971 |
| SETD3 | -1.741837212 | 0.796728249 | 0.028798231 | 0.100545971 |
| HNRNPH1 | -2.036649038 | 0.931594984 | 0.028801284 | 0.100545971 |
| SLC25A11 | -1.688095675 | 0.77233655 | 0.028837592 | 0.10061992 |
| GLYCTK | -2.285500284 | 1.04568715 | 0.02884163 | 0.10061992 |
| MPP7 | -2.285864163 | 1.046131052 | 0.028884094 | 0.100734596 |
| VEZT | -1.738528342 | 0.795717779 | 0.028899445 | 0.100754674 |
| WEE1 | -2.000851488 | 0.915973024 | 0.028932867 | 0.100837715 |
| DHX16 | -1.811366338 | 0.829332763 | 0.028953071 | 0.100874653 |
| SDHA | -1.537858054 | 0.704360067 | 0.029010724 | 0.101041994 |
| HDAC11 | -2.288713738 | 1.048435164 | 0.029037231 | 0.101100782 |
| CDC42 | 1.113375807 | 0.510127917 | 0.029069526 | 0.101179678 |
| ING1 | -1.916925588 | 0.878543562 | 0.029114277 | 0.101273048 |
| NUDC | -1.604236034 | 0.735241461 | 0.02911564 | 0.101273048 |
| GTF3C1 | -1.669797276 | 0.765479049 | 0.029155651 | 0.10137864 |
| TNNT2 | -2.199210535 | 1.008487492 | 0.029205476 | 0.101518275 |
| SNX14 | -1.827164302 | 0.838228078 | 0.029272756 | 0.101718467 |
| PPBP | -2.283918761 | 1.048096545 | 0.02932342 | 0.10186081 |
| NAPG | -1.80798232 | 0.830065433 | 0.029397086 | 0.102082937 |
| METRNL | -1.532445487 | 0.70375957 | 0.029442326 | 0.102206237 |
| FZD8 | -2.277843611 | 1.046511126 | 0.02950989 | 0.102406927 |
| APOL1 | 1.350757755 | 0.620864769 | 0.029584657 | 0.10263247 |
| MYBBP1A | -2.02128064 | 0.929315543 | 0.029628557 | 0.102750819 |
| PRPF4B | -1.773312721 | 0.815805476 | 0.029728008 | 0.103061675 |
| LTB4R | -2.1780538 | 1.0023204 | 0.029779448 | 0.103198705 |
| NELFCD | -1.623719442 | 0.747256173 | 0.029787189 | 0.103198705 |
| MYO1C | -1.489133833 | 0.68556348 | 0.029845767 | 0.103345162 |
| RYK | -1.708607754 | 0.786620523 | 0.029849145 | 0.103345162 |
| PYGL | 1.505523001 | 0.693322794 | 0.029896386 | 0.103474606 |
| CDADC1 | 1.685886974 | 0.776891526 | 0.03000369 | 0.103807513 |
| EIF2D | -2.050992331 | 0.945242898 | 0.030021656 | 0.103807513 |
| SMG1P1 | -2.27496766 | 1.048470279 | 0.030022228 | 0.103807513 |
| RRP9 | -1.719308273 | 0.792496335 | 0.030045943 | 0.103855315 |
| RTF1 | -1.727427513 | 0.796517199 | 0.030103497 | 0.104020013 |
| HS2ST1 | -2.01230854 | 0.928492874 | 0.030213141 | 0.104330872 |
| TMEM189 | -1.848862113 | 0.853078557 | 0.03021333 | 0.104330872 |
| CXCR2 | 2.175714529 | 1.004139616 | 0.0302543 | 0.104349687 |
| SERPINA3 | -1.82184159 | 0.840830822 | 0.030256516 | 0.104349687 |
| TMEM65 | -2.268036708 | 1.046799429 | 0.030262374 | 0.104349687 |
| YARS | -1.874998755 | 0.865445372 | 0.030271998 | 0.104349687 |
| PHACTR1 | 1.649083157 | 0.761183955 | 0.030275194 | 0.104349687 |
| TTC19 | -1.55415714 | 0.717404573 | 0.030283628 | 0.104349687 |
| EIF2AK1 | 1.314099023 | 0.606610152 | 0.030288339 | 0.104349687 |
| KRT4 | -2.266650049 | 1.046441888 | 0.030307031 | 0.104379839 |
| PDLIM3 | -1.854097525 | 0.856145777 | 0.030339245 | 0.104456527 |
| AIF1L | -2.242877671 | 1.035900828 | 0.030376422 | 0.104550249 |
| TNFRSF10D | -1.762208817 | 0.814235968 | 0.030445297 | 0.104745527 |
| RGL2 | -1.605351569 | 0.741794318 | 0.030453109 | 0.104745527 |
| MKNK2 | -1.835850316 | 0.848500792 | 0.030491984 | 0.104828668 |
| NPR3 | -2.276685064 | 1.052280916 | 0.030497246 | 0.104828668 |
| GPR3 | -2.267923216 | 1.048361009 | 0.030517831 | 0.10483666 |
| COX5B | -1.647460916 | 0.761556435 | 0.030519538 | 0.10483666 |
| STOML1 | -2.266124917 | 1.047905376 | 0.030577486 | 0.105001369 |
| COL4A3 | -2.263582055 | 1.047248519 | 0.030660098 | 0.105250635 |
| DCP1A | -1.90187956 | 0.880189859 | 0.030713838 | 0.105400657 |
| NOC4L | -1.922968799 | 0.89013458 | 0.030748491 | 0.105472632 |
| CROCC | -2.264426482 | 1.048234168 | 0.030754899 | 0.105472632 |
| AOC3 | -2.146907347 | 0.994236548 | 0.030822817 | 0.10567104 |
| NPR1 | -2.144837994 | 0.993347092 | 0.030834424 | 0.105673614 |
| CGN | -1.833810256 | 0.849346633 | 0.030843694 | 0.105673614 |
| POLA2 | -2.147385584 | 0.995006776 | 0.030915257 | 0.10588425 |
| FNDC4 | -2.263893112 | 1.049326769 | 0.030968907 | 0.106032626 |
| SPATA13 | 1.627207508 | 0.754263836 | 0.030978773 | 0.106032626 |
| SNHG11 | -2.262452327 | 1.049104867 | 0.031040355 | 0.106208789 |
| ARFIP2 | -1.713141896 | 0.794580123 | 0.031080997 | 0.106313209 |
| FIZ1 | 1.78569665 | 0.828429573 | 0.031121152 | 0.106415897 |
| PAPPA | 1.81932149 | 0.844190089 | 0.031153321 | 0.10649122 |
| PRPF4 | -1.73743189 | 0.806300385 | 0.031175952 | 0.106515785 |
| RIPK4 | -2.265084522 | 1.051201464 | 0.031180794 | 0.106515785 |
| IPO5 | -1.824504769 | 0.84715391 | 0.031264689 | 0.106744808 |
| C17orf80 | -2.106711161 | 0.978208095 | 0.031268168 | 0.106744808 |
| ARFGAP2 | -1.709860315 | 0.794126598 | 0.031308215 | 0.106846786 |
| ZNF577 | -2.253540439 | 1.046970092 | 0.031362706 | 0.106997978 |
| ENKUR | 1.952500201 | 0.907304724 | 0.031399053 | 0.107087188 |
| MDN1 | -1.893057551 | 0.879805122 | 0.031422704 | 0.107104979 |
| SAP30 | -2.139066978 | 0.994150379 | 0.031424668 | 0.107104979 |
| RUVBL2 | -1.606018869 | 0.746508854 | 0.031446784 | 0.107145579 |
| APEH | -1.664093668 | 0.773577993 | 0.031463203 | 0.107163761 |
| SAA2 | 2.227824087 | 1.035693135 | 0.03147253 | 0.107163761 |
| ACAP3 | -2.251650513 | 1.04736178 | 0.031568608 | 0.10739255 |
| DYSF | 1.317127247 | 0.612667339 | 0.0315691 | 0.10739255 |
| CRYL1 | -1.840063869 | 0.855928718 | 0.031572119 | 0.10739255 |
| FKBP1B | -2.253412853 | 1.048255602 | 0.03158063 | 0.10739255 |
| FBXL3 | -1.759415693 | 0.818793335 | 0.031650977 | 0.107596926 |
| C6orf48 | -1.478790263 | 0.688421303 | 0.031706682 | 0.107751413 |
| FOXRED1 | -2.150805331 | 1.001448823 | 0.031738096 | 0.107803131 |
| ARHGEF37 | -2.259495044 | 1.052102089 | 0.0317455 | 0.107803131 |
| ZNF266 | -1.788655305 | 0.832897294 | 0.031752698 | 0.107803131 |
| TLE3 | -1.833163135 | 0.853745067 | 0.031777202 | 0.107851453 |
| FAM222B | 1.386929171 | 0.645988152 | 0.031794193 | 0.107874254 |
| ELMOD3 | -1.993865848 | 0.928842498 | 0.031824064 | 0.107940729 |
| TMEM198B | -2.258327311 | 1.052105865 | 0.031834467 | 0.107941148 |
| FYN | -1.531831883 | 0.713707189 | 0.031848812 | 0.107954931 |
| FBXW2 | -1.716847135 | 0.800105567 | 0.031890873 | 0.108062619 |
| NEK3 | -2.103768693 | 0.980693128 | 0.031938017 | 0.108187458 |
| WDR46 | -1.495976143 | 0.697584282 | 0.031992082 | 0.108335652 |
| FMR1 | 1.486488903 | 0.693276359 | 0.032020824 | 0.108398024 |
| HSF4 | -2.246084589 | 1.04779599 | 0.032062723 | 0.108473769 |
| FAM189A2 | -2.013251293 | 0.939185716 | 0.032063858 | 0.108473769 |
| TINAGL1 | -1.998031152 | 0.932235876 | 0.032091599 | 0.108532652 |
| SLC35F1 | 2.255283768 | 1.052407008 | 0.032114964 | 0.108576702 |
| OGG1 | -2.244476563 | 1.047442743 | 0.032127915 | 0.108585527 |
| MCM3AP | -1.695148959 | 0.791156801 | 0.032143566 | 0.108603469 |
| MRPS26 | -1.720406398 | 0.803100908 | 0.032177028 | 0.108673235 |
| BRWD3 | 1.55409157 | 0.72549687 | 0.032184912 | 0.108673235 |
| LRRC20 | -2.243394027 | 1.047382164 | 0.032201056 | 0.108692795 |
| SNX20 | 1.895675617 | 0.885571381 | 0.032304348 | 0.109006412 |
| PPP6C | 1.246655742 | 0.582631334 | 0.032379092 | 0.109177274 |
| FAM180A | -2.250603466 | 1.05185597 | 0.032383075 | 0.109177274 |
| TOB2 | -1.692613014 | 0.791084262 | 0.032386173 | 0.109177274 |
| MED16 | -1.86241627 | 0.870925271 | 0.03248153 | 0.10946359 |
| MED12 | -1.87496953 | 0.876993281 | 0.03252064 | 0.109560232 |
| PPRC1 | -1.606597332 | 0.751542572 | 0.032538422 | 0.109568237 |
| HOXB3 | -2.241181265 | 1.048445191 | 0.032547298 | 0.109568237 |
| RP9P | -2.2399574 | 1.047934753 | 0.032557589 | 0.109568237 |
| MAPK12 | -2.159485848 | 1.010328862 | 0.032564753 | 0.109568237 |
| GABRB3 | 2.040644082 | 0.95494216 | 0.032603725 | 0.109664228 |
| LAMA4 | -1.880307705 | 0.880237173 | 0.032668201 | 0.109845912 |
| CLEC5A | 1.964943198 | 0.919952869 | 0.03268616 | 0.109871115 |
| FZD10 | -2.238307588 | 1.048082463 | 0.032710266 | 0.109916962 |
| C16orf45 | -2.094772252 | 0.980950384 | 0.032724124 | 0.109928352 |
| LHFP | -1.927018527 | 0.902548507 | 0.032754 | 0.109993111 |
| AHR | -1.732585657 | 0.811531077 | 0.032764351 | 0.109993111 |
| LGALS3 | -1.601529231 | 0.750857961 | 0.032930277 | 0.110514808 |
| MCAT | -2.121557736 | 0.994882729 | 0.032968215 | 0.110606781 |
| TMEM173 | 1.236980773 | 0.580127969 | 0.032985888 | 0.110630727 |
| ZNF800 | -1.995539046 | 0.935989122 | 0.033005947 | 0.110662661 |
| WASH7P | -2.234740393 | 1.048506177 | 0.033059789 | 0.110777226 |
| SART1 | -1.508422114 | 0.707734453 | 0.033061216 | 0.110777226 |
| SLCO4A1 | -1.48245187 | 0.695622401 | 0.033079606 | 0.110803489 |
| TMEM97 | 1.548355793 | 0.727044669 | 0.033199939 | 0.111171095 |
| COL18A1 | -1.853546927 | 0.87044214 | 0.033218575 | 0.111198042 |
| HMGN2 | -1.800637242 | 0.845971396 | 0.03329691 | 0.111424747 |
| PMEPA1 | -1.745417601 | 0.820083356 | 0.033308763 | 0.111428902 |
| ARSD | -2.028339085 | 0.953221893 | 0.033347271 | 0.111522194 |
| UHRF1BP1 | -2.090095559 | 0.982877934 | 0.033461173 | 0.111806284 |
| PAPSS2 | -1.7886078 | 0.841149483 | 0.0334712 | 0.111806284 |
| PION | -1.793501042 | 0.843497791 | 0.033481081 | 0.111806284 |
| RNF166 | -1.934926272 | 0.910022625 | 0.033483303 | 0.111806284 |
| PSMB8 | -1.328489349 | 0.624814541 | 0.033485455 | 0.111806284 |
| CENPO | -2.231266376 | 1.049494102 | 0.033499934 | 0.111819075 |
| ANAPC1 | -1.830387486 | 0.86131453 | 0.033577497 | 0.112042356 |
| DIS3L2 | -2.107979499 | 0.992255754 | 0.033634068 | 0.112195474 |
| EPHA7 | 2.235044353 | 1.052370721 | 0.033685328 | 0.11233078 |
| CISD3 | -1.851854891 | 0.872028662 | 0.033702183 | 0.112351308 |
| MPPE1 | -2.229623239 | 1.050012542 | 0.033718226 | 0.112369117 |
| CPD | 1.216608148 | 0.573030342 | 0.033744387 | 0.112415223 |
| FAM208A | -1.696559505 | 0.799137246 | 0.033754782 | 0.112415223 |
| RPL18A | -1.176975762 | 0.554424899 | 0.033764176 | 0.112415223 |
| COMTD1 | -2.228042761 | 1.049675397 | 0.033787244 | 0.112456371 |
| NOP2 | -1.567163486 | 0.73869409 | 0.03387702 | 0.11271945 |
| FPGS | -1.805414696 | 0.851055768 | 0.033889638 | 0.112725718 |
| PSMD11 | 1.320075361 | 0.622453814 | 0.033941976 | 0.112864056 |
| AHCTF1 | -1.706028827 | 0.805214167 | 0.034113563 | 0.113391414 |
| LOC646214 | 2.231752948 | 1.053396842 | 0.034122166 | 0.113391414 |
| PALMD | -2.231750831 | 1.053478428 | 0.034136216 | 0.113402215 |
| HNRPDL | -1.575096833 | 0.74355645 | 0.034147255 | 0.113403013 |
| ST3GAL1 | -1.770651539 | 0.836020048 | 0.034179011 | 0.113472587 |
| AZI2 | 1.246789604 | 0.588712654 | 0.034190002 | 0.113473203 |
| BCKDK | -1.707751374 | 0.80650037 | 0.034218819 | 0.113532958 |
| KRT16P2 | -2.230356481 | 1.053480471 | 0.034248706 | 0.113596229 |
| CD96 | -2.220964001 | 1.049481793 | 0.0343237 | 0.113757669 |
| MARK4 | -2.081820523 | 0.983743377 | 0.034325832 | 0.113757669 |
| PCF11 | -1.808816013 | 0.854757063 | 0.034329878 | 0.113757669 |
| CPXM2 | -2.224110514 | 1.051167528 | 0.034357753 | 0.113790125 |
| SIL1 | -1.739004605 | 0.821911338 | 0.034361345 | 0.113790125 |
| PLSCR4 | -2.125468976 | 1.004725837 | 0.034389773 | 0.113848362 |
| CCM2L | -2.226408801 | 1.053064522 | 0.034496585 | 0.114165974 |
| PGC | -1.729724329 | 0.818277057 | 0.034527102 | 0.114230971 |
| EPS8L2 | -1.830540921 | 0.866112422 | 0.034556779 | 0.114293146 |
| ECE1 | -1.908262344 | 0.903251203 | 0.034629912 | 0.114487773 |
| PPIB | -1.823791277 | 0.863303786 | 0.03463743 | 0.114487773 |
| U2AF2 | 1.123756936 | 0.532283914 | 0.034755264 | 0.114841104 |
| NUP133 | -1.792054577 | 0.848889774 | 0.034767425 | 0.11484515 |
| TDRD10 | -2.217874724 | 1.050849648 | 0.034810682 | 0.114925551 |
| RHOA | 1.182471538 | 0.560301233 | 0.034822102 | 0.114925551 |
| LYRM9 | -2.220625384 | 1.052233564 | 0.034824598 | 0.114925551 |
| MLF2 | 1.392087863 | 0.659926352 | 0.034904945 | 0.115154516 |
| MVP | -1.577082079 | 0.747919116 | 0.034976832 | 0.115355438 |
| WDR4 | -1.995001859 | 0.946177616 | 0.034989009 | 0.115359366 |
| PTPN9 | -1.720238953 | 0.815989928 | 0.03501698 | 0.115415349 |
| SYNGR1 | -2.214577636 | 1.050601675 | 0.035038457 | 0.115449902 |
| RREB1 | -1.751076171 | 0.830987493 | 0.035098232 | 0.115610582 |
| AGBL5 | -2.13312838 | 1.01251234 | 0.035137713 | 0.11569638 |
| KLF4 | -1.920752512 | 0.911748769 | 0.035146315 | 0.11569638 |
| TRMT1 | -1.656415135 | 0.7863569 | 0.035165984 | 0.115724238 |
| JOSD1 | -1.363812052 | 0.647486583 | 0.035176818 | 0.115724238 |
| SYNGR2 | -1.568367831 | 0.744734329 | 0.035209329 | 0.115794915 |
| LINC00094 | -2.211185263 | 1.050144511 | 0.035239031 | 0.115856315 |
| ABCB7 | -1.960070952 | 0.931113302 | 0.035284068 | 0.115958654 |
| CD9 | -1.873330181 | 0.889947626 | 0.035292244 | 0.115958654 |
| PHF12 | 1.450884585 | 0.689358032 | 0.035318349 | 0.115994556 |
| ARL14EP | -1.925493927 | 0.914893425 | 0.035325263 | 0.115994556 |
| WASH3P | -2.084603212 | 0.990596679 | 0.035344312 | 0.116020826 |
| KRT23 | 2.033679682 | 0.966459332 | 0.035355957 | 0.116022781 |
| NRGN | -2.02166104 | 0.96095861 | 0.035396231 | 0.116118658 |
| FUT8 | -2.062601716 | 0.980481243 | 0.035407889 | 0.116120627 |
| ARHGEF3 | -1.980414398 | 0.941488195 | 0.035422644 | 0.116132745 |
| SNCG | -2.198545036 | 1.045420919 | 0.035463692 | 0.116209048 |
| CPSF6 | -1.661427785 | 0.790071852 | 0.035476096 | 0.116209048 |
| MBOAT1 | -2.206003339 | 1.049070641 | 0.035481789 | 0.116209048 |
| WRNIP1 | -1.717285835 | 0.816697035 | 0.035490184 | 0.116209048 |
| RHBDD1 | 1.4758801 | 0.702088997 | 0.03554217 | 0.116342993 |
| DAPK2 | -1.874134986 | 0.891689692 | 0.035572608 | 0.116371994 |
| FNBP4 | -1.861420238 | 0.885642988 | 0.035573193 | 0.116371994 |
| C22orf46 | -2.208802813 | 1.051163517 | 0.035615231 | 0.116464148 |
| TRAF6 | -1.917928382 | 0.912778293 | 0.035623545 | 0.116464148 |
| GSDMD | -1.688497377 | 0.803693605 | 0.035647836 | 0.116507289 |
| AKAP10 | -1.845441334 | 0.878572943 | 0.035684994 | 0.116592446 |
| FGD5-AS1 | -1.687972875 | 0.80376022 | 0.035720479 | 0.116672084 |
| ADAMTS14 | -2.203872916 | 1.049787257 | 0.035785888 | 0.116849381 |
| TFB2M | -1.92519334 | 0.917158942 | 0.035809527 | 0.116890223 |
| DNAJB12 | -1.684441098 | 0.802584271 | 0.035837037 | 0.116943668 |
| KIAA0247 | -1.791552984 | 0.853938928 | 0.035906271 | 0.117078227 |
| PARP8 | 1.375021733 | 0.655415084 | 0.035910417 | 0.117078227 |
| CITED2 | -1.915284423 | 0.91298431 | 0.035920306 | 0.117078227 |
| DHRSX | -2.003663344 | 0.955126281 | 0.035922869 | 0.117078227 |
| MXRA5 | 2.031175646 | 0.968968583 | 0.036062284 | 0.117496135 |
| TP53BP2 | -1.960660544 | 0.935395148 | 0.036075334 | 0.117502196 |
| PLCB1 | -2.203793509 | 1.051563406 | 0.036106104 | 0.117556605 |
| POLM | -2.067541767 | 0.986610178 | 0.036117556 | 0.117556605 |
| ZBTB4 | -1.780316609 | 0.849617059 | 0.036132416 | 0.117556605 |
| BET1L | -1.642354517 | 0.783796214 | 0.036136817 | 0.117556605 |
| C9orf84 | 2.202765591 | 1.051656021 | 0.036209367 | 0.117756134 |
| IFT74 | -2.197988536 | 1.049561934 | 0.036242513 | 0.117827438 |
| MRPL12 | -1.670616246 | 0.79781217 | 0.036260252 | 0.117848626 |
| ARID5A | -1.52728041 | 0.729496851 | 0.036294918 | 0.117924795 |
| PITHD1 | -1.638076239 | 0.782783151 | 0.036382113 | 0.118171532 |
| ZBTB16 | -2.20286885 | 1.052957027 | 0.036431486 | 0.118295311 |
| CPLX1 | -2.201874454 | 1.052556399 | 0.036444766 | 0.11830185 |
| ESYT1 | -1.648477092 | 0.788147756 | 0.036475761 | 0.118365875 |
| ZMIZ2 | -1.695798112 | 0.811313244 | 0.036600815 | 0.11870422 |
| NAA10 | -1.480226417 | 0.708199662 | 0.036606497 | 0.11870422 |
| DRG2 | -1.749288815 | 0.836963563 | 0.036614047 | 0.11870422 |
| TTC7B | -1.825189012 | 0.873330797 | 0.036625242 | 0.11870422 |
| CTSW | 1.475828268 | 0.706404206 | 0.036688628 | 0.118851447 |
| ATXN1L | -1.542168694 | 0.73817637 | 0.036693305 | 0.118851447 |
| COG3 | -1.781717414 | 0.853033955 | 0.036736279 | 0.118953953 |
| FAM150B | -1.812512352 | 0.868292437 | 0.036847965 | 0.119278816 |
| SLC34A2 | 1.155033795 | 0.553359685 | 0.036860025 | 0.119281086 |
| API5 | 1.374841116 | 0.658992898 | 0.036953623 | 0.119547133 |
| PSMB9 | 1.335511008 | 0.640357814 | 0.037017597 | 0.11971721 |
| S1PR5 | 1.953598495 | 0.936887482 | 0.03705108 | 0.119774359 |
| FLI1 | -1.508337437 | 0.723380337 | 0.037058079 | 0.119774359 |
| MPZL1 | -1.952084069 | 0.936786941 | 0.03717764 | 0.120123816 |
| BCAP31 | -1.226310074 | 0.588718251 | 0.037249682 | 0.120319566 |
| LTA4H | -1.735089548 | 0.833076219 | 0.037274012 | 0.12036113 |
| FAM129A | 1.478781364 | 0.710083138 | 0.037292553 | 0.120383984 |
| MDFIC | -2.04344462 | 0.981356208 | 0.037318184 | 0.120396134 |
| BAZ2A | -1.770187695 | 0.850141166 | 0.037321654 | 0.120396134 |
| EFCC1 | -2.191121893 | 1.052397003 | 0.03733975 | 0.120396134 |
| LGALS1 | -1.098557207 | 0.527668898 | 0.037351028 | 0.120396134 |
| AP3D1 | -1.659916142 | 0.797347234 | 0.037360894 | 0.120396134 |
| EFHD2 | 1.359698141 | 0.653199233 | 0.037379215 | 0.120396134 |
| LOC643837 | -1.70608287 | 0.819637213 | 0.03738727 | 0.120396134 |
| C9orf135 | 2.01092855 | 0.966095231 | 0.037388039 | 0.120396134 |
| FAM46C | -1.848093022 | 0.888055698 | 0.037428844 | 0.120490586 |
| GOLGA4 | -1.677907272 | 0.807150973 | 0.037635529 | 0.121118813 |
| DDX51 | -1.907548174 | 0.918070176 | 0.037729594 | 0.121384335 |
| TMEM175 | -2.1860743 | 1.052513757 | 0.037801276 | 0.121577704 |
| CTDNEP1 | 1.367367711 | 0.65846063 | 0.037837319 | 0.121623823 |
| RAPGEF2 | -1.59043522 | 0.765885389 | 0.03783878 | 0.121623823 |
| WDR55 | -1.857718215 | 0.894700772 | 0.037860945 | 0.121657828 |
| RORA | -1.783561527 | 0.859111825 | 0.037889066 | 0.121710946 |
| LOC100130476 | 2.006633986 | 0.967109285 | 0.037997823 | 0.122022979 |
| MTMR1 | -2.046697566 | 0.986822414 | 0.038076687 | 0.122238854 |
| LRP4 | -2.182984398 | 1.052607529 | 0.038090219 | 0.122244924 |
| MLST8 | -2.093094825 | 1.009531028 | 0.038141223 | 0.122371214 |
| SPTB | -2.177278527 | 1.050665951 | 0.038238938 | 0.122614198 |
| VTI1A | 1.376542619 | 0.664268129 | 0.03824031 | 0.122614198 |
| HSPA12A | -1.858411792 | 0.896945522 | 0.03827161 | 0.122656711 |
| PPP1R2 | -1.664997352 | 0.80363842 | 0.038281859 | 0.122656711 |
| TSC22D1 | -1.409665445 | 0.680440393 | 0.038293828 | 0.122656711 |
| EMC3 | -1.526626989 | 0.736921942 | 0.038300291 | 0.122656711 |
| OR2AT4 | 2.16212452 | 1.043810085 | 0.03832355 | 0.12269378 |
| CD47 | 1.279050179 | 0.618090316 | 0.038512499 | 0.123261124 |
| VAV2 | -1.904886302 | 0.920620341 | 0.03853357 | 0.123285516 |
| SYBU | -2.177742547 | 1.052544544 | 0.038543601 | 0.123285516 |
| FBXO25 | -1.933697616 | 0.934967838 | 0.038621508 | 0.123497095 |
| NBPF9 | -2.172460548 | 1.050635008 | 0.038662641 | 0.123590988 |
| PNP | -1.425654464 | 0.689610062 | 0.038702674 | 0.123681309 |
| PSMA6 | 1.381394742 | 0.668322448 | 0.038738068 | 0.123756753 |
| CST6 | -1.979852399 | 0.9582404 | 0.038815895 | 0.123967674 |
| GFOD1 | -2.170233978 | 1.050525885 | 0.038842189 | 0.124013932 |
| SETD7 | -1.519958052 | 0.735928164 | 0.038888763 | 0.124112197 |
| TUSC2 | -1.73471504 | 0.839972666 | 0.038903688 | 0.124112197 |
| PLXDC1 | -2.174833606 | 1.053165503 | 0.038918792 | 0.124112197 |
| ZNF740 | -1.787733531 | 0.865718303 | 0.038920242 | 0.124112197 |
| PRMT7 | -2.071230205 | 1.003064647 | 0.038932273 | 0.124112871 |
| MUM1L1 | 2.144582874 | 1.039178499 | 0.039043412 | 0.124379738 |
| IFT57 | -1.383148677 | 0.670235028 | 0.039048342 | 0.124379738 |
| SPRY4 | -1.395842519 | 0.676431973 | 0.03906162 | 0.124379738 |
| DTX3L | 1.480190038 | 0.71731357 | 0.039063363 | 0.124379738 |
| C22orf34 | -2.169068593 | 1.05135629 | 0.039101734 | 0.124464174 |
| MED17 | -1.81565602 | 0.880305485 | 0.039157353 | 0.124603444 |
| CPE | -2.097439639 | 1.017155104 | 0.039201576 | 0.124683641 |
| C4orf33 | 1.832484049 | 0.888686066 | 0.039206303 | 0.124683641 |
| SLC19A2 | -1.810357501 | 0.878021344 | 0.039221022 | 0.124692689 |
| ANKH | -1.673655862 | 0.811796634 | 0.039239271 | 0.124697967 |
| MPP3 | -1.842246949 | 0.893603296 | 0.039246432 | 0.124697967 |
| PDIA5 | -1.71472299 | 0.83207731 | 0.03932464 | 0.124857027 |
| CDKN2A | 1.563591842 | 0.758740449 | 0.0393247 | 0.124857027 |
| LMO7 | -1.760560539 | 0.854352868 | 0.039332163 | 0.124857027 |
| MTG1 | -1.742132706 | 0.845523777 | 0.039358564 | 0.124903076 |
| VCP | -1.492763924 | 0.724674225 | 0.039407114 | 0.125019366 |
| RABAC1 | -1.581026693 | 0.767726595 | 0.039459608 | 0.125148097 |
| NDUFA13 | -1.579169895 | 0.76704281 | 0.039515631 | 0.125280318 |
| SEPT4 | -2.007816683 | 0.975314128 | 0.03952913 | 0.125280318 |
| MAN2A1 | -2.106690366 | 1.023388671 | 0.039537965 | 0.125280318 |
| LINC00641 | -1.80945947 | 0.879048984 | 0.039549019 | 0.125280318 |
| LIPA | 1.485943214 | 0.721931543 | 0.039562471 | 0.125281251 |
| P4HB | -1.391588594 | 0.676126884 | 0.039573175 | 0.125281251 |
| FHL1 | -2.040960037 | 0.991952043 | 0.039636343 | 0.12544341 |
| SH3BP5L | -1.950117426 | 0.948020079 | 0.039682155 | 0.12555056 |
| CHPF2 | -1.788265618 | 0.869464131 | 0.039710804 | 0.125603359 |
| ACD | -1.840993603 | 0.895156792 | 0.039723197 | 0.125604725 |
| PLA2G6 | -2.161332355 | 1.051149329 | 0.039766991 | 0.12570535 |
| VAMP5 | -1.878020034 | 0.913506756 | 0.039798331 | 0.125762557 |
| CERS4 | -1.846888602 | 0.898412336 | 0.039809041 | 0.125762557 |
| TOP1MT | -1.997403692 | 0.97235011 | 0.03995613 | 0.126162265 |
| AAR2 | -1.614908856 | 0.786162647 | 0.039959594 | 0.126162265 |
| DGKG | -2.161303935 | 1.052561771 | 0.040036231 | 0.126366233 |
| DBN1 | -1.766264912 | 0.860644457 | 0.040144546 | 0.126670035 |
| ADCY9 | -1.710908974 | 0.833880821 | 0.040194664 | 0.126790078 |
| MPND | -2.156202774 | 1.051244165 | 0.040257592 | 0.126950443 |
| SCARNA16 | 2.151896141 | 1.049286679 | 0.040284662 | 0.126997668 |
| SLC24A6 | -1.646389108 | 0.803037536 | 0.040344729 | 0.127123591 |
| MRPL41 | -1.682605256 | 0.820741702 | 0.040354353 | 0.127123591 |
| NAB1 | 1.22571392 | 0.597898744 | 0.040360923 | 0.127123591 |
| YBX1 | -1.184963297 | 0.578191136 | 0.040419911 | 0.12727121 |
| SFXN4 | -1.910966761 | 0.932806467 | 0.040499206 | 0.12746454 |
| NSUN5 | -1.833471899 | 0.895008244 | 0.040505836 | 0.12746454 |
| DIAPH1 | -1.713725864 | 0.836603889 | 0.040517725 | 0.12746454 |
| ETV6 | 1.173969491 | 0.573401923 | 0.040621154 | 0.127751645 |
| ABI3 | -2.040941788 | 0.997012927 | 0.040652538 | 0.127812066 |
| ZNF480 | -1.967913627 | 0.961818644 | 0.040753028 | 0.128089657 |
| MED27 | -1.763503712 | 0.862055967 | 0.040786373 | 0.128156105 |
| BRMS1 | -1.697039942 | 0.82964056 | 0.040804381 | 0.128174336 |
| ERC1 | -1.607259061 | 0.785884282 | 0.040839091 | 0.128198472 |
| PDGFB | -1.385767878 | 0.677594131 | 0.040842074 | 0.128198472 |
| NRIP3 | -2.15193131 | 1.052279529 | 0.040853015 | 0.128198472 |
| PAPD7 | -1.705428283 | 0.833975242 | 0.040860898 | 0.128198472 |
| FAM208B | -1.832940631 | 0.89673199 | 0.040951295 | 0.128426305 |
| PCED1A | -1.853987077 | 0.907058576 | 0.040957975 | 0.128426305 |
| EPOR | -2.0141309 | 0.985608492 | 0.04099896 | 0.12851644 |
| CASP1 | 1.763242348 | 0.862952354 | 0.041025982 | 0.128562769 |
| ACBD6 | -1.859927774 | 0.910421584 | 0.041059346 | 0.128593914 |
| FABP3 | -1.865306599 | 0.913059299 | 0.041060413 | 0.128593914 |
| PPP6R3 | -1.530201796 | 0.749074809 | 0.041073328 | 0.12859601 |
| UBE3C | -1.553920967 | 0.761119851 | 0.041188789 | 0.128919068 |
| SMURF1 | -1.604655461 | 0.786044388 | 0.041207996 | 0.128940752 |
| CORO1C | -1.894499975 | 0.928112794 | 0.041227083 | 0.128962049 |
| IQCA1 | 1.902942249 | 0.932335999 | 0.041246086 | 0.12898307 |
| NDUFS4 | -1.667833815 | 0.817207996 | 0.041261496 | 0.128992846 |
| TOX2 | -1.948366824 | 0.954735405 | 0.041276667 | 0.12900187 |
| ATP6V1H | -1.70269083 | 0.834588754 | 0.041334841 | 0.129145245 |
| TBKBP1 | -2.00636647 | 0.983538416 | 0.041355582 | 0.129171613 |
| CEP19 | 1.896282796 | 0.929955853 | 0.041438995 | 0.129390818 |
| PLCG2 | -1.835192953 | 0.900047261 | 0.041450406 | 0.129390818 |
| CADM4 | 1.613839676 | 0.791700483 | 0.041505225 | 0.129507206 |
| SZT2 | -1.71062718 | 0.839246086 | 0.041520906 | 0.129507206 |
| PYGB | -1.904202342 | 0.93423281 | 0.041524689 | 0.129507206 |
| SNHG8 | -1.265950918 | 0.621139129 | 0.041538765 | 0.129512639 |
| SLC7A5 | -1.366880334 | 0.670837724 | 0.041592729 | 0.129642401 |
| MAP3K14 | -1.655838354 | 0.812727623 | 0.041611564 | 0.129662621 |
| NT5C | -1.677262021 | 0.823416101 | 0.041654494 | 0.129736022 |
| METTL7A | -2.046904772 | 1.004910607 | 0.041659829 | 0.129736022 |
| URB2 | -1.872093041 | 0.919472931 | 0.041745315 | 0.129963699 |
| RPL7 | -1.280514314 | 0.62901298 | 0.041775283 | 0.13001845 |
| SAMD12 | -2.140809587 | 1.051743802 | 0.041802015 | 0.130041107 |
| ADH1B | -2.067024085 | 1.015520607 | 0.041807331 | 0.130041107 |
| PPFIA3 | -2.139788295 | 1.051471022 | 0.041846589 | 0.130124677 |
| TRAM2 | -1.721331472 | 0.846052778 | 0.041896793 | 0.130239267 |
| CPSF3 | -1.765330753 | 0.867727387 | 0.041908245 | 0.130239267 |
| CD27-AS1 | -2.140114691 | 1.05216952 | 0.041951415 | 0.130306009 |
| HIST1H2BK | 1.668014565 | 0.820110949 | 0.041962797 | 0.130306009 |
| PRPF40B | -2.021979147 | 0.994164456 | 0.041966948 | 0.130306009 |
| IRF5 | 1.792458739 | 0.881661921 | 0.042047933 | 0.130518873 |
| LCAT | -1.88829078 | 0.928955127 | 0.042082439 | 0.130568518 |
| FBXL17 | -2.136380084 | 1.051036541 | 0.042088795 | 0.130568518 |
| TULP3 | -1.748069138 | 0.860050985 | 0.04210124 | 0.130568552 |
| BRF1 | -2.013182605 | 0.990662201 | 0.042137613 | 0.130642774 |
| CPSF4 | -1.68955593 | 0.831479174 | 0.042154843 | 0.130657618 |
| ANO2 | -2.135628332 | 1.051433694 | 0.042238933 | 0.130879622 |
| CD5 | -2.136215354 | 1.052072784 | 0.04230752 | 0.131027043 |
| AKR1A1 | -1.505801818 | 0.741612226 | 0.042311466 | 0.131027043 |
| ADIRF | -1.748564735 | 0.861314377 | 0.042345146 | 0.131092683 |
| TOR4A | 1.697886696 | 0.836491022 | 0.042379642 | 0.131160809 |
| 1-Mar | 1.799720435 | 0.886785824 | 0.042408697 | 0.131212057 |
| RHOBTB2 | -1.949714813 | 0.960765228 | 0.042424148 | 0.131221201 |
| SLC10A3 | -1.829664744 | 0.901909004 | 0.042493156 | 0.131395946 |
| PPP1R11 | 1.317394885 | 0.64970199 | 0.042591794 | 0.131620036 |
| RAB2B | 1.673206415 | 0.825178962 | 0.042591985 | 0.131620036 |
| CLDND1 | 1.298402643 | 0.640403523 | 0.042613777 | 0.131620036 |
| PLEKHA7 | -2.131790763 | 1.051460737 | 0.042615763 | 0.131620036 |
| AEBP1 | -1.433240587 | 0.70705171 | 0.042655648 | 0.131704487 |
| WDR89 | 1.691152557 | 0.834453477 | 0.04269731 | 0.13179437 |
| FUNDC2 | -1.362444447 | 0.67239914 | 0.042739829 | 0.131886848 |
| PSAP | -1.342793209 | 0.662774029 | 0.042762771 | 0.131890158 |
| THAP9-AS1 | -1.71728562 | 0.84762875 | 0.042766021 | 0.131890158 |
| SH3BP5-AS1 | -1.89989564 | 0.937840318 | 0.042783237 | 0.131904514 |
| C16orf91 | -1.889776011 | 0.933147372 | 0.042850577 | 0.132073351 |
| TRAPPC1 | 1.490556206 | 0.736174714 | 0.042894932 | 0.132171268 |
| GIMAP1 | -1.906344766 | 0.941773731 | 0.042948881 | 0.13229868 |
| EVA1B | -1.821132461 | 0.899860041 | 0.042991225 | 0.13239028 |
| HK1 | -1.411100695 | 0.697330518 | 0.043013886 | 0.13242123 |
| GPX4 | -1.373672389 | 0.678883314 | 0.043028906 | 0.132428646 |
| RPL5 | -1.162610435 | 0.574727461 | 0.043084489 | 0.132560862 |
| CLDN4 | -2.073173033 | 1.025502744 | 0.04321601 | 0.132926573 |
| ZBTB8OS | 1.495696047 | 0.740006789 | 0.043259859 | 0.133022484 |
| RAP1GDS1 | -1.65799095 | 0.820411489 | 0.043287421 | 0.133068269 |
| KLF16 | -1.943432668 | 0.961951367 | 0.043352029 | 0.133227876 |
| MPEG1 | 1.669619839 | 0.826494468 | 0.043370712 | 0.133246297 |
| TBC1D10A | -1.628546229 | 0.806328131 | 0.043413832 | 0.133339764 |
| NADK | 1.291291326 | 0.639386971 | 0.043427266 | 0.133342023 |
| UPF3B | -1.792501132 | 0.887659808 | 0.043450234 | 0.133373549 |
| BCS1L | -1.762717952 | 0.87332944 | 0.043550833 | 0.13364328 |
| KAT8 | -1.494109836 | 0.740539398 | 0.043633282 | 0.133831428 |
| HNRNPM | -1.829148725 | 0.90661657 | 0.043637635 | 0.133831428 |
| BMP1 | -1.859433446 | 0.922240063 | 0.043777589 | 0.13422145 |
| ABL1 | -1.707742548 | 0.847525262 | 0.043907227 | 0.134557559 |
| UBB | -1.313024459 | 0.651663592 | 0.04391714 | 0.134557559 |
| EMP3 | 1.210893075 | 0.600999277 | 0.043925656 | 0.134557559 |
| ACTN4 | -1.582189387 | 0.785598713 | 0.044010392 | 0.134777813 |
| CTC1 | -1.638994213 | 0.814346867 | 0.044151593 | 0.135170811 |
| GPN1 | -1.643169829 | 0.81654439 | 0.044183478 | 0.135199805 |
| NFIX | 1.583374543 | 0.786842556 | 0.044186814 | 0.135199805 |
| CABIN1 | -1.749113385 | 0.869263888 | 0.044201212 | 0.135204463 |
| CCNG2 | -1.643432237 | 0.816924012 | 0.044248188 | 0.135308741 |
| SYNPO2 | -1.937209428 | 0.963062322 | 0.044271622 | 0.135340589 |
| ECI2 | -1.751717695 | 0.870899488 | 0.044284379 | 0.135340589 |
| TTC37 | -1.773973808 | 0.882212161 | 0.044344 | 0.13548337 |
| SMARCD3 | -1.871426019 | 0.930819962 | 0.044376878 | 0.135544385 |
| PRDM11 | -2.002421327 | 0.996384384 | 0.044464265 | 0.135760413 |
| SNCA | -2.115250624 | 1.052572568 | 0.044473462 | 0.135760413 |
| INO80C | -1.742449739 | 0.867940389 | 0.04468917 | 0.136379244 |
| CCND3 | -1.865422871 | 0.92999275 | 0.044872608 | 0.136899262 |
| PDIA6 | -1.707783735 | 0.851663891 | 0.044938245 | 0.137059688 |
| NOTCH1 | -1.720676164 | 0.858419687 | 0.045019781 | 0.137268501 |
| COX6B1 | 1.217055209 | 0.60739654 | 0.045099601 | 0.137471963 |
| CBL | -1.76688023 | 0.882062776 | 0.045164088 | 0.137628581 |
| C22orf39 | -1.734485446 | 0.866211578 | 0.04524379 | 0.13783146 |
| KCNQ3 | -2.108970715 | 1.053525686 | 0.045303897 | 0.137974544 |
| CCR7 | -2.104578821 | 1.051600908 | 0.045359053 | 0.13810247 |
| WDR18 | -1.783337576 | 0.891332471 | 0.045418838 | 0.138216325 |
| C14orf166B | 2.049848319 | 1.02455627 | 0.045422773 | 0.138216325 |
| MTMR10 | -1.639405078 | 0.819497806 | 0.045446337 | 0.138243309 |
| ADCY5 | -2.106727727 | 1.053157536 | 0.04545797 | 0.138243309 |
| CDK5RAP2 | -1.662221462 | 0.831012914 | 0.045474849 | 0.138254601 |
| ENO1 | -1.499588813 | 0.749959612 | 0.045547859 | 0.138398518 |
| CHCHD7 | 1.330678958 | 0.665540621 | 0.045565573 | 0.138398518 |
| MDK | 1.616996939 | 0.808760376 | 0.045570246 | 0.138398518 |
| SEPP1 | -1.898386271 | 0.949521081 | 0.045574905 | 0.138398518 |
| CLCF1 | -1.999175478 | 1.000175545 | 0.045627336 | 0.138517679 |
| SYPL1 | -1.545564653 | 0.773332486 | 0.045654122 | 0.138522039 |
| PLOD1 | -1.596152316 | 0.798648133 | 0.045655155 | 0.138522039 |
| LY75 | 1.77278779 | 0.887335229 | 0.045729857 | 0.138621714 |
| SEMA3A | 1.548926608 | 0.775289315 | 0.045730848 | 0.138621714 |
| ATG2A | -1.789777544 | 0.895868148 | 0.045736875 | 0.138621714 |
| BYSL | -1.680352502 | 0.841143751 | 0.045749242 | 0.138621714 |
| HARS | -1.347915 | 0.674748438 | 0.04575401 | 0.138621714 |
| USP2 | -2.101687518 | 1.052276138 | 0.045795039 | 0.138706002 |
| DDX6 | -1.614690376 | 0.808596284 | 0.045835448 | 0.138788363 |
| IST1 | -1.827209227 | 0.915324059 | 0.045907481 | 0.138966405 |
| EGFLAM | -2.102502435 | 1.053441197 | 0.045951098 | 0.139058351 |
| SIGLEC1 | 1.771269683 | 0.887562448 | 0.045971335 | 0.139079514 |
| IL3RA | -1.349543652 | 0.676458574 | 0.046041462 | 0.139201974 |
| CCT7 | -1.571563125 | 0.787763516 | 0.046046354 | 0.139201974 |
| H1F0 | -1.776898313 | 0.890711464 | 0.046051581 | 0.139201974 |
| IER5L | 1.636017443 | 0.820144161 | 0.046065513 | 0.139204015 |
| PDSS1 | 1.77768141 | 0.891311835 | 0.046102345 | 0.139237163 |
| SEC22B | 1.523361585 | 0.763823199 | 0.046109417 | 0.139237163 |
| TMEM119 | -2.100809306 | 1.053460829 | 0.046130438 | 0.139237163 |
| NUP107 | -1.693728825 | 0.849341422 | 0.046133783 | 0.139237163 |
| INO80D | -1.72032263 | 0.86271284 | 0.046142779 | 0.139237163 |
| TGM2 | -1.309206866 | 0.656763394 | 0.046215212 | 0.139398915 |
| RAB33B | 1.798888878 | 0.902444234 | 0.046222933 | 0.139398915 |
| ELMSAN1 | -1.555284071 | 0.780351856 | 0.046255438 | 0.139456893 |
| ABL2 | -1.443273542 | 0.724662932 | 0.046409683 | 0.139863792 |
| BAIAP2-AS1 | -2.095102214 | 1.051979357 | 0.046417038 | 0.139863792 |
| UBE2E3 | -1.70436177 | 0.855922375 | 0.046452595 | 0.139930782 |
| PEX16 | -1.751599947 | 0.87973927 | 0.046476006 | 0.139961153 |
| ADAMDEC1 | 2.062130539 | 1.035969856 | 0.046532427 | 0.140090889 |
| RUSC2 | -1.721635018 | 0.865380293 | 0.046651021 | 0.140390554 |
| CAP1 | 1.132448155 | 0.569245291 | 0.046658702 | 0.140390554 |
| DOCK4 | -1.603850537 | 0.806423353 | 0.046718385 | 0.140529865 |
| TMUB2 | -1.628597846 | 0.819013042 | 0.046757712 | 0.140607886 |
| MACROD2 | -2.093879166 | 1.053229358 | 0.046805474 | 0.140711217 |
| FOXO1 | -1.586030982 | 0.798137336 | 0.046904059 | 0.140967236 |
| RPP25 | -1.966356265 | 0.989616163 | 0.046923634 | 0.140973015 |
| 9-Mar | -1.977231501 | 0.995130948 | 0.046932831 | 0.140973015 |
| CLPTM1L | -1.595317019 | 0.803043893 | 0.046968119 | 0.141038668 |
| STRA13 | -1.627565057 | 0.819367342 | 0.046992479 | 0.141071475 |
| ABHD2 | -1.630135452 | 0.820937467 | 0.047066655 | 0.141217338 |
| PLEKHB2 | -1.652095487 | 0.83200149 | 0.047067963 | 0.141217338 |
| TSHZ1 | -1.93025164 | 0.972686035 | 0.047205143 | 0.141568328 |
| GEM | -2.035758822 | 1.025884282 | 0.047211912 | 0.141568328 |
| APOL4 | -2.089891751 | 1.053266275 | 0.047233463 | 0.14159252 |
| GPAA1 | -1.806408783 | 0.910501819 | 0.047259124 | 0.141616451 |
| SHKBP1 | 1.456412864 | 0.73412081 | 0.047268418 | 0.141616451 |
| HUS1 | -1.725724109 | 0.87015613 | 0.047341184 | 0.141794002 |
| RANBP1 | -1.382661664 | 0.697302539 | 0.047381732 | 0.141874983 |
| CC2D1A | -2.006361749 | 1.012232779 | 0.047466374 | 0.142087911 |
| TP53 | 1.556454555 | 0.785344673 | 0.047493293 | 0.142127975 |
| RNASEH2B | -1.761516994 | 0.889227183 | 0.047596578 | 0.142372832 |
| SNRNP27 | 1.264247233 | 0.638217859 | 0.04760223 | 0.142372832 |
| ARID2 | -1.897412454 | 0.957964682 | 0.047628229 | 0.142410033 |
| FAM65A | -1.193358328 | 0.602669752 | 0.047690065 | 0.142554334 |
| SPATA18 | 1.811906919 | 0.915107717 | 0.04770431 | 0.142556334 |
| KIAA1279 | -1.812578127 | 0.91579242 | 0.04778836 | 0.142766876 |
| GBA | -1.652491228 | 0.834962397 | 0.047802437 | 0.142768313 |
| RAE1 | -1.542378925 | 0.779423264 | 0.047830412 | 0.142789465 |
| IPW | -2.083781744 | 1.053044665 | 0.047836714 | 0.142789465 |
| SRSF8 | -1.73405938 | 0.877550614 | 0.048152278 | 0.143651446 |
| SETDB1 | -1.623080385 | 0.821389887 | 0.048152851 | 0.143651446 |
| GGNBP2 | -1.469007657 | 0.743467786 | 0.04816768 | 0.143654873 |
| C1orf162 | 1.77413676 | 0.898011171 | 0.048196831 | 0.143669578 |
| UBTF | -1.721345321 | 0.871302065 | 0.048199974 | 0.143669578 |
| AHCYL2 | -1.731914952 | 0.876780917 | 0.048232871 | 0.143726838 |
| MFSD5 | -1.665692835 | 0.84339306 | 0.048269286 | 0.143794546 |
| PGM5 | -2.010654188 | 1.018135161 | 0.048286292 | 0.14380441 |
| LMO3 | -1.741775581 | 0.882289191 | 0.048364126 | 0.143966263 |
| ARSB | 1.392404389 | 0.705329022 | 0.048368058 | 0.143966263 |
| DESI2 | -1.587395576 | 0.804246848 | 0.048408287 | 0.144045175 |
| ZMIZ1-AS1 | -2.077930968 | 1.052990504 | 0.048454386 | 0.144141502 |
| C11orf48 | -1.601601145 | 0.811676348 | 0.048472587 | 0.144154812 |
| NSUN2 | -1.639761162 | 0.831223093 | 0.048528742 | 0.14428095 |
| EIF3L | -1.653006357 | 0.838165929 | 0.048590114 | 0.144422526 |
| IQCE | -1.998059401 | 1.013487427 | 0.048670215 | 0.144619675 |
| CSPP1 | -1.775230779 | 0.90063586 | 0.048714034 | 0.144708932 |
| SEC23A | -1.632252385 | 0.828175934 | 0.048735255 | 0.144724497 |
| TUBG1 | -1.714865976 | 0.870137302 | 0.048746838 | 0.144724497 |
| LOC728392 | -2.021610041 | 1.025898827 | 0.048772569 | 0.144738504 |
| CARS2 | -1.621977142 | 0.823122538 | 0.048779122 | 0.144738504 |
| LBP | 2.075586292 | 1.053464484 | 0.048809955 | 0.144789078 |
| SEC61A1 | -1.419399679 | 0.720581426 | 0.048861553 | 0.144901206 |
| TTC17 | -1.55554128 | 0.789818566 | 0.04889663 | 0.144964289 |
| UQCRC2 | -1.251265372 | 0.635576118 | 0.048986346 | 0.14518928 |
| ATP6V1C1 | 1.522106498 | 0.773427613 | 0.049067895 | 0.145389946 |
| EIF4ENIF1 | -1.651184016 | 0.83946073 | 0.049188051 | 0.145704857 |
| TICAM1 | -1.681058114 | 0.854747271 | 0.04921421 | 0.145741234 |
| MIR22HG | -1.692236779 | 0.8605772 | 0.049252722 | 0.145801469 |
| GPD1 | -2.071377366 | 1.053540024 | 0.049285709 | 0.145801469 |
| LGI4 | -2.064745322 | 1.050170776 | 0.049286559 | 0.145801469 |
| EFNB2 | -1.790144588 | 0.9105175 | 0.049290088 | 0.145801469 |
| KCTD12 | -1.881312719 | 0.957166772 | 0.049356223 | 0.145940821 |
| ARHGAP15 | 1.648903712 | 0.838955019 | 0.049364993 | 0.145940821 |
| ABHD16A | 1.1537586 | 0.587082301 | 0.049386273 | 0.145962639 |
| MRPL32 | -1.558736476 | 0.793356659 | 0.049444788 | 0.146066616 |
| SCAND1 | -1.675554858 | 0.852831014 | 0.049449273 | 0.146066616 |
| C17orf97 | 1.898042465 | 0.966379732 | 0.049521372 | 0.146238449 |
| ATOH8 | -1.949631674 | 0.99357957 | 0.049735709 | 0.146830103 |
| ADD1 | -1.838296358 | 0.937058877 | 0.049788974 | 0.146946041 |
| UMPS | -1.688677856 | 0.860867872 | 0.049809148 | 0.146964278 |
| HAPLN3 | 1.100257353 | 0.56096918 | 0.049838115 | 0.146981859 |
| CCDC3 | -2.036475534 | 1.038325189 | 0.049843101 | 0.146981859 |
| TUG1 | -1.224294713 | 0.624324436 | 0.049880052 | 0.147049529 |
| ARHGEF17 | -1.898595014 | 0.968668752 | 0.049995276 | 0.147338267 |
| UPK2 | 4.235439273 | 1.005251628 | 2.52E-05 | NaN |
| LYPD1 | 4.142314293 | 1.005713178 | 3.81E-05 | NaN |
| ACOT6 | 4.270697509 | 1.05019449 | 4.77E-05 | NaN |
| MS4A3 | 4.051730598 | 1.002127 | 5.27E-05 | NaN |
| IFNL1 | 4.101680525 | 1.048990313 | 9.22E-05 | NaN |
| PAX3 | 4.101680525 | 1.048990313 | 9.22E-05 | NaN |
| HBD | 3.866101225 | 0.994658658 | 0.000101548 | NaN |
| LOC642236 | 3.873113328 | 1.001459018 | 0.00010997 | NaN |
| EFCAB10 | 3.818027496 | 0.991908825 | 0.000118518 | NaN |
| FBXO39 | 3.738795681 | 0.980889893 | 0.00013805 | NaN |
| C8A | 3.744944708 | 0.998283272 | 0.000175861 | NaN |
| CNTN5 | 3.822491133 | 1.033730104 | 0.000217506 | NaN |
| CLCA3P | 3.752795753 | 1.029067169 | 0.000265533 | NaN |
| CEACAM8 | 3.566812625 | 0.997445408 | 0.000348961 | NaN |
| MND1 | 3.595769447 | 1.005905559 | 0.000350685 | NaN |
| HMX1 | 4.586071315 | 1.345530075 | 0.00065351 | NaN |
| IL12B | 3.530412819 | 1.037802069 | 0.000669394 | NaN |
| TCF23 | 3.525242802 | 1.046389934 | 0.000754532 | NaN |
| BPIFB2 | 3.525242802 | 1.046389934 | 0.000754532 | NaN |
| LINC00309 | 3.528890019 | 1.050995358 | 0.000786039 | NaN |
| PPP2R2C | 3.443538138 | 1.027670414 | 0.000805728 | NaN |
| FBN2 | 3.408886685 | 1.019385247 | 0.000825615 | NaN |
| PTPRZ1 | 3.271372824 | 0.979140314 | 0.000834573 | NaN |
| C5orf58 | 3.481660266 | 1.052960823 | 0.000944548 | NaN |
| GABRA5 | 4.476468802 | 1.386112222 | 0.001240008 | NaN |
| EYA1 | 3.163193864 | 0.989721717 | 0.00139326 | NaN |
| HBG2 | 3.329076162 | 1.053535203 | 0.00157818 | NaN |
| EPHA1-AS1 | 3.109237199 | 0.99045058 | 0.001694012 | NaN |
| ACVR1C | 3.090018718 | 0.987084045 | 0.001745378 | NaN |
| HGD | 2.956085452 | 0.95955272 | 0.002065208 | NaN |
| TMEM252 | 3.127946725 | 1.017283907 | 0.002106423 | NaN |
| MYL1 | 4.300797021 | 1.404103249 | 0.002191151 | NaN |
| PRL | 3.208893871 | 1.049543309 | 0.002232518 | NaN |
| CYP4F11 | 2.996650829 | 0.980267866 | 0.002235856 | NaN |
| SSTR5 | 3.132441698 | 1.03235162 | 0.002411122 | NaN |
| SIGLEC6 | 2.955593655 | 0.974386019 | 0.002419044 | NaN |
| TM4SF20 | 3.189793692 | 1.053024818 | 0.002452247 | NaN |
| LOC285540 | 3.017959243 | 0.997337943 | 0.002478003 | NaN |
| MUC5B | 2.996716419 | 0.992560964 | 0.002534631 | NaN |
| TMIGD2 | 3.10433943 | 1.030370916 | 0.00258818 | NaN |
| CACNG6 | 2.989545805 | 1.024738204 | 0.003529909 | NaN |
| LINC00520 | 2.873666563 | 0.989878827 | 0.00369549 | NaN |
| MED18 | 2.826280498 | 0.97513878 | 0.003751476 | NaN |
| EBLN1 | 4.091037996 | 1.424725612 | 0.004085847 | NaN |
| KIAA0825 | 2.820467632 | 0.988647627 | 0.004332848 | NaN |
| LOC100131626 | 2.939459945 | 1.050250146 | 0.00512899 | NaN |
| C20orf26 | 2.71959095 | 0.971861135 | 0.005136715 | NaN |
| FAM86JP | 2.807975158 | 1.006899448 | 0.005291444 | NaN |
| DNAH7 | 2.629267119 | 0.954210821 | 0.005861392 | NaN |
| IGF2BP3 | 2.718431518 | 0.989226013 | 0.00599529 | NaN |
| FCRL1 | 2.868356643 | 1.046057197 | 0.006105429 | NaN |
| ANKS4B | 2.850442762 | 1.041156533 | 0.006185811 | NaN |
| SLC25A21 | 2.815872657 | 1.032961862 | 0.00641035 | NaN |
| CCDC138 | 2.622329746 | 0.969915127 | 0.00685785 | NaN |
| EMBP1 | 2.799099878 | 1.040074517 | 0.007118497 | NaN |
| MIR3945 | 2.805402881 | 1.047693637 | 0.007413093 | NaN |
| VSX1 | 2.731679863 | 1.032737554 | 0.008167011 | NaN |
| SMC1B | 2.724011 | 1.034144659 | 0.008436771 | NaN |
| LINC00659 | 2.662100313 | 1.015729533 | 0.008770437 | NaN |
| SNORA28 | 2.754116587 | 1.051335588 | 0.008802366 | NaN |
| SCGB2A1 | 2.695083611 | 1.031819214 | 0.009002144 | NaN |
| IGFBP1 | 2.69474994 | 1.048429173 | 0.010161819 | NaN |
| CCDC148 | 2.599497488 | 1.022020978 | 0.010975201 | NaN |
| C8orf34 | 2.486686394 | 0.979693873 | 0.011141538 | NaN |
| TSPAN19 | 2.659944987 | 1.050922862 | 0.011371958 | NaN |
| SCN2A | 2.659944987 | 1.050922862 | 0.011371958 | NaN |
| HIST1H2AE | 2.625981038 | 1.043551183 | 0.011856414 | NaN |
| ZBED3 | 2.634548805 | 1.050907 | 0.012178531 | NaN |
| FAM133B | 2.585216506 | 1.031401071 | 0.012192981 | NaN |
| TMEM52B | 2.540839004 | 1.015850033 | 0.012377501 | NaN |
| HTA | 2.604815172 | 1.048522413 | 0.012981651 | NaN |
| HIST2H2BC | 2.596279863 | 1.05245037 | 0.013629201 | NaN |
| CADPS | 2.553816308 | 1.036520864 | 0.01374594 | NaN |
| KIF9-AS1 | 2.475544307 | 1.011319436 | 0.014371698 | NaN |
| TOMM20L | 2.570758813 | 1.051670937 | 0.01450725 | NaN |
| MRVI1-AS1 | 2.497977027 | 1.040511029 | 0.016362792 | NaN |
| CLPS | 2.483781693 | 1.034723321 | 0.016375788 | NaN |
| RASSF10 | 2.39687379 | 0.99993285 | 0.016528356 | NaN |
| SIX1 | 2.289248098 | 0.956953674 | 0.016746592 | NaN |
| LOC79015 | 2.442398031 | 1.021906035 | 0.01684646 | NaN |
| RBM11 | 2.29230219 | 0.964628015 | 0.017484456 | NaN |
| PROB1 | 2.470963815 | 1.040264984 | 0.017533669 | NaN |
| MRE11A | -2.437412565 | 1.037163074 | 0.018769553 | NaN |
| FKTN | -2.425906448 | 1.03762563 | 0.019390362 | NaN |
| INTS7 | -2.4241204 | 1.037875751 | 0.019509194 | NaN |
| ZNF564 | -2.420828468 | 1.038234319 | 0.019717619 | NaN |
| ZNF514 | -2.417245562 | 1.038105999 | 0.019884765 | NaN |
| ZPLD1 | 2.429238379 | 1.043489196 | 0.019912331 | NaN |
| FAM189B | -2.412563203 | 1.038238072 | 0.020141093 | NaN |
| SPATA2L | -2.411405249 | 1.038371123 | 0.020216995 | NaN |
| TROAP | 2.390302515 | 1.02954166 | 0.020248276 | NaN |
| MNS1 | 2.207131308 | 0.951453347 | 0.020354553 | NaN |
| TAF4 | -2.403842963 | 1.038680398 | 0.020649957 | NaN |
| POMZP3 | -2.399103737 | 1.03870422 | 0.02090431 | NaN |
| ZNF449 | -2.397687602 | 1.038874394 | 0.021000984 | NaN |
| MAP1LC3B2 | 2.374849337 | 1.030315282 | 0.021168062 | NaN |
| CLEC6A | 2.373257147 | 1.029812216 | 0.021191604 | NaN |
| SPAG4 | -2.394015371 | 1.038924138 | 0.021204595 | NaN |
| ZNF182 | -2.392713608 | 1.039044213 | 0.021289958 | NaN |
| APAF1 | -2.390729372 | 1.038955586 | 0.021386595 | NaN |
| LOC645212 | -2.391042429 | 1.039462912 | 0.021433087 | NaN |
| ZNF528 | -2.390160866 | 1.039080208 | 0.021433155 | NaN |
| ZSCAN26 | -2.390869834 | 1.039392389 | 0.021433652 | NaN |
| PARP16 | -2.388904662 | 1.039620719 | 0.02156966 | NaN |
| FANCC | -2.385211788 | 1.039496722 | 0.021757018 | NaN |
| SIPA1L3 | -2.382635783 | 1.039262877 | 0.021869841 | NaN |
| SDR39U1 | -2.380813709 | 1.039324394 | 0.021978915 | NaN |
| IBA57 | -2.380073523 | 1.040556366 | 0.022177817 | NaN |
| LOC400657 | -2.37473728 | 1.039576043 | 0.022352001 | NaN |
| SDHAP1 | -2.372212611 | 1.039713396 | 0.022512823 | NaN |
| MIPEP | -2.370639072 | 1.040197885 | 0.02266546 | NaN |
| ENOX2 | -2.370107446 | 1.040127312 | 0.022686658 | NaN |
| PLEKHM1P | -2.368478303 | 1.039876624 | 0.022747249 | NaN |
| MS4A8B | 2.176986102 | 0.955868644 | 0.02275668 | NaN |
| ZNF549 | -2.369854967 | 1.041070327 | 0.022824235 | NaN |
| UST | -2.36840937 | 1.040458625 | 0.02282729 | NaN |
| METTL2A | -2.368252326 | 1.040589199 | 0.022853413 | NaN |
| HSD17B1 | -2.368645224 | 1.041287898 | 0.022922339 | NaN |
| PPP1R3E | -2.365457081 | 1.039963686 | 0.022932495 | NaN |
| FHDC1 | -2.36413095 | 1.040231944 | 0.023044508 | NaN |
| C16orf88 | -2.363017294 | 1.040234732 | 0.023109514 | NaN |
| C10orf128 | -2.363606225 | 1.040696347 | 0.023136229 | NaN |
| THG1L | -2.362900283 | 1.040419495 | 0.023140717 | NaN |
| PTPLA | -2.360580487 | 1.040086361 | 0.023231835 | NaN |
| RPL32P3 | -2.362689203 | 1.041582425 | 0.023306964 | NaN |
| HLTF | -2.359952285 | 1.041032754 | 0.023394273 | NaN |
| ZFP28 | -2.356861193 | 1.040238115 | 0.023470129 | NaN |
| CYP2R1 | -2.356337625 | 1.040610282 | 0.023550723 | NaN |
| MATK | -2.355230987 | 1.040467026 | 0.023596963 | NaN |
| THAP1 | -2.354490424 | 1.040767243 | 0.023681088 | NaN |
| SLC27A4 | -2.352009721 | 1.040398283 | 0.023778953 | NaN |
| SNORA49 | 2.345333633 | 1.037465402 | 0.023781687 | NaN |
| AMDHD2 | -2.354588404 | 1.041805077 | 0.023814762 | NaN |
| USP46 | -2.352305801 | 1.041029665 | 0.023846376 | NaN |
| GGTLC1 | -2.353120672 | 1.041634538 | 0.023879309 | NaN |
| XRCC3 | -2.350018375 | 1.040990626 | 0.023977927 | NaN |
| CHCHD6 | -2.350301704 | 1.042307378 | 0.024139438 | NaN |
| TDRD1 | 2.257188537 | 1.002362981 | 0.024330652 | NaN |
| FAM179B | -2.342500763 | 1.040733078 | 0.024397066 | NaN |
| ADAP2 | -2.342693602 | 1.040836038 | 0.024399434 | NaN |
| UBXN8 | -2.344120666 | 1.041547635 | 0.024410058 | NaN |
| SMIM11 | -2.344045115 | 1.042247255 | 0.024510602 | NaN |
| DCAF4 | -2.338507747 | 1.041139654 | 0.024697359 | NaN |
| PLEKHF1 | -2.332351169 | 1.041434007 | 0.025119675 | NaN |
| KIF16B | -2.332325229 | 1.042653022 | 0.025291956 | NaN |
| TMEM120B | -2.329041369 | 1.041212006 | 0.02529575 | NaN |
| SLIT2 | -2.328993665 | 1.041312488 | 0.025312862 | NaN |
| NAPSB | -2.328744099 | 1.041894621 | 0.025410442 | NaN |
| DSN1 | -2.328835562 | 1.042404516 | 0.025476517 | NaN |
| NUP37 | -2.328883103 | 1.042558138 | 0.025495179 | NaN |
| CCDC66 | -2.326946747 | 1.042077399 | 0.025549709 | NaN |
| GMNN | -2.324252737 | 1.041465912 | 0.025633955 | NaN |
| SPTBN5 | -2.321740494 | 1.042075998 | 0.025880824 | NaN |
| AR | -2.321801516 | 1.042136817 | 0.02588559 | NaN |
| CDH17 | 2.347013161 | 1.053521617 | 0.025895267 | NaN |
| G2E3 | -2.318728623 | 1.042507553 | 0.026136067 | NaN |
| LOC100505716 | 2.233259884 | 1.004693892 | 0.026227523 | NaN |
| TTC9 | -2.314985114 | 1.041751704 | 0.026269502 | NaN |
| PSTPIP1 | -2.313749754 | 1.041750778 | 0.026349578 | NaN |
| CCDC135 | 2.251184026 | 1.014878086 | 0.026542444 | NaN |
| CDK20 | -2.311390481 | 1.042031062 | 0.026544 | NaN |
| MGAT3 | -2.313959926 | 1.043243011 | 0.026551765 | NaN |
| BMX | -2.311127735 | 1.04199157 | 0.026555458 | NaN |
| LINC00847 | -2.310429914 | 1.04187011 | 0.026583509 | NaN |
| SIMC1 | -2.310262913 | 1.042005778 | 0.026614167 | NaN |
| LOC338799 | -2.312480291 | 1.043813658 | 0.026731602 | NaN |
| C2orf44 | -2.307943401 | 1.042922041 | 0.026900493 | NaN |
| OXLD1 | -2.309343576 | 1.043742071 | 0.026927888 | NaN |
| NUDT19 | -2.305303407 | 1.04217204 | 0.026965415 | NaN |
| IDI2 | -2.305615673 | 1.042931439 | 0.027056144 | NaN |
| WDSUB1 | -2.302278256 | 1.042622385 | 0.027233041 | NaN |
| MTHFSD | -2.303679928 | 1.043966836 | 0.027337817 | NaN |
| DALRD3 | -2.301897373 | 1.043647274 | 0.027410076 | NaN |
| RPL23AP82 | -2.298172848 | 1.042462474 | 0.027484866 | NaN |
| POPDC2 | -2.298108508 | 1.04248503 | 0.027492552 | NaN |
| SND1-IT1 | 2.285306619 | 1.037489236 | 0.027613924 | NaN |
| BACH2 | -2.294309998 | 1.042428972 | 0.027741197 | NaN |
| LOC90784 | -2.291467772 | 1.042863993 | 0.02800021 | NaN |
| OVGP1 | -2.290531249 | 1.04286956 | 0.028065208 | NaN |
| FOXL1 | -2.292191084 | 1.043705842 | 0.028077336 | NaN |
| PPIL3 | -2.289684859 | 1.042591231 | 0.028081337 | NaN |
| ZFY | -2.289684859 | 1.042591231 | 0.028081337 | NaN |
| XPNPEP3 | -2.290434685 | 1.043494672 | 0.028166081 | NaN |
| LLGL1 | -2.290720258 | 1.044319471 | 0.028270996 | NaN |
| TTI2 | -2.288088462 | 1.043254948 | 0.028291474 | NaN |
| HSD17B7 | -2.287714393 | 1.043252836 | 0.028316985 | NaN |
| SPATA5L1 | -2.284677185 | 1.04340477 | 0.028550609 | NaN |
| CASP8AP2 | -2.279137224 | 1.043085087 | 0.028889106 | NaN |
| RNF219 | -2.278478541 | 1.043000285 | 0.028922401 | NaN |
| RSBN1L-AS1 | -2.277736423 | 1.042992899 | 0.028973527 | NaN |
| ARHGEF28 | -2.279169151 | 1.043753404 | 0.028989595 | NaN |
| ZAP70 | -2.278886602 | 1.044265031 | 0.029088315 | NaN |
| SEPSECS | -2.277336403 | 1.043719924 | 0.029113809 | NaN |
| LOC100499484 | 2.235643278 | 1.024935843 | 0.029164788 | NaN |
| C2orf54 | -2.273793885 | 1.043311759 | 0.029301949 | NaN |
| MDH1B | 2.189930382 | 1.005477115 | 0.029405946 | NaN |
| HCG11 | -2.272778676 | 1.043760187 | 0.029443934 | NaN |
| C7orf25 | -2.275150121 | 1.044956574 | 0.029460605 | NaN |
| ZSCAN22 | -2.271099224 | 1.043423042 | 0.029511525 | NaN |
| BAHCC1 | -2.270677397 | 1.043235027 | 0.029512427 | NaN |
| PDE1B | -2.273327026 | 1.0449876 | 0.029595784 | NaN |
| ELMO3 | -2.268698367 | 1.043297713 | 0.029664191 | NaN |
| TFCP2L1 | -2.269472739 | 1.043846108 | 0.029694251 | NaN |
| KAL1 | -2.268480333 | 1.043594424 | 0.029726295 | NaN |
| CD300C | -2.272530873 | 1.045496511 | 0.029732337 | NaN |
| CABLES2 | -2.266975574 | 1.043393989 | 0.029803376 | NaN |
| AP4S1 | -2.268729678 | 1.044518186 | 0.02985305 | NaN |
| ZNF324 | -2.266039272 | 1.043439236 | 0.029878133 | NaN |
| METTL8 | -2.263926565 | 1.043513612 | 0.030043016 | NaN |
| ZC3H10 | -2.264096796 | 1.0437837 | 0.030073233 | NaN |
| TMEM231 | -2.262856151 | 1.043565794 | 0.030129134 | NaN |
| SCUBE1 | -2.264019898 | 1.045148732 | 0.030294539 | NaN |
| WDR53 | -2.260522568 | 1.043836002 | 0.030342402 | NaN |
| C3orf62 | -2.25971846 | 1.04360826 | 0.030365196 | NaN |
| TTC39A | -2.259968907 | 1.044022505 | 0.03041262 | NaN |
| ARMCX6 | -2.260190707 | 1.044169906 | 0.03041976 | NaN |
| BEND3 | -2.259005535 | 1.04380371 | 0.030448596 | NaN |
| TRIM32 | -2.257287147 | 1.043705746 | 0.030559488 | NaN |
| ADAP1 | -2.257683665 | 1.043914584 | 0.030563554 | NaN |
| RAD52 | -2.257156791 | 1.043909351 | 0.030601583 | NaN |
| GMEB1 | -2.258368973 | 1.04548557 | 0.030763775 | NaN |
| ARSI | -2.254994531 | 1.044206716 | 0.030809161 | NaN |
| CYB561D1 | -2.254654335 | 1.044160256 | 0.030826967 | NaN |
| WIF1 | -2.257277247 | 1.046230718 | 0.030964163 | NaN |
| GNG7 | -2.256594653 | 1.046019859 | 0.030981105 | NaN |
| UBOX5 | -2.251258765 | 1.043878193 | 0.031034525 | NaN |
| C1orf159 | -2.249699252 | 1.044342091 | 0.031226178 | NaN |
| PLEKHG5 | -2.249699252 | 1.044342091 | 0.031226178 | NaN |
| NOMO2 | -2.248332665 | 1.044140169 | 0.031296189 | NaN |
| ADSSL1 | -2.248314574 | 1.044814748 | 0.031406911 | NaN |
| C17orf51 | -2.248004603 | 1.044757109 | 0.031420935 | NaN |
| LRIG2 | -2.246433994 | 1.044036185 | 0.031422399 | NaN |
| ZNF691 | -2.247450781 | 1.044578223 | 0.031433681 | NaN |
| FAM86A | -2.246900067 | 1.044470317 | 0.031457734 | NaN |
| SPOCD1 | -2.244430136 | 1.044224278 | 0.031604642 | NaN |
| FAM21C | -2.244007827 | 1.044293873 | 0.031648044 | NaN |
| ARL15 | -2.247877822 | 1.046758999 | 0.031756318 | NaN |
| PRAME | 2.054827215 | 0.957290983 | 0.031832932 | NaN |
| C6orf203 | -2.242130594 | 1.044805809 | 0.03187471 | NaN |
| DOPEY1 | -2.239960771 | 1.044474415 | 0.031986303 | NaN |
| SNORA53 | 2.212931614 | 1.032748919 | 0.032132488 | NaN |
| ACAD10 | -2.239716351 | 1.045344865 | 0.032148272 | NaN |
| SPICE1 | -2.23680534 | 1.044751909 | 0.032274694 | NaN |
| FAM117B | -2.236994897 | 1.044960545 | 0.032294543 | NaN |
| SUZ12P1 | -2.234473887 | 1.045019694 | 0.032499522 | NaN |
| C20orf118 | 2.134628359 | 0.998381243 | 0.032509488 | NaN |
| ANKRD37 | -2.238498694 | 1.047004509 | 0.032516506 | NaN |
| CCT6P1 | -2.232837898 | 1.044473297 | 0.032535865 | NaN |
| LOC152225 | 2.13970729 | 1.001834826 | 0.032696658 | NaN |
| C1orf200 | 2.146835257 | 1.00564877 | 0.032779279 | NaN |
| HBG1 | 2.146835257 | 1.00564877 | 0.032779279 | NaN |
| LOC100652770 | 2.146835257 | 1.00564877 | 0.032779279 | NaN |
| LMO1 | 1.993981468 | 0.934073935 | 0.032784312 | NaN |
| LOC503519 | 1.993981468 | 0.934073935 | 0.032784312 | NaN |
| CNGB1 | 2.187354271 | 1.024984785 | 0.032839847 | NaN |
| LOC100506123 | -2.229436489 | 1.04482676 | 0.03286032 | NaN |
| CEP290 | -2.230831257 | 1.045524794 | 0.032867738 | NaN |
| ACPL2 | -2.230600641 | 1.04672835 | 0.033087369 | NaN |
| OGFOD2 | -2.228296296 | 1.045759205 | 0.033106207 | NaN |
| AREG | -2.222605653 | 1.044845117 | 0.033402585 | NaN |
| BNC2 | -2.224678469 | 1.045924802 | 0.033420368 | NaN |
| LOC100292680 | 2.239530329 | 1.052909884 | 0.033420793 | NaN |
| ZSCAN31 | -2.225538792 | 1.046618509 | 0.033469237 | NaN |
| SNORD10 | -2.222282557 | 1.046118204 | 0.033643975 | NaN |
| LOC100507634 | 2.019111664 | 0.951104181 | 0.033761122 | NaN |
| MDFI | -2.223587234 | 1.047487132 | 0.033772092 | NaN |
| TCF7 | -2.220413241 | 1.046559723 | 0.033868762 | NaN |
| LOC100996455 | 2.21959613 | 1.047100247 | 0.0340267 | NaN |
| DDX28 | -2.21555246 | 1.045278234 | 0.03404135 | NaN |
| ABCC10 | -2.219540705 | 1.04794485 | 0.034175592 | NaN |
| NBEAL1 | -2.217452141 | 1.047410495 | 0.034253032 | NaN |
| DHRS13 | -2.210497909 | 1.045376237 | 0.034468516 | NaN |
| INPP4B | -2.209340304 | 1.046359593 | 0.034733295 | NaN |
| C14orf28 | -2.206359666 | 1.045576623 | 0.03484246 | NaN |
| RBM12B | -2.209025901 | 1.047290482 | 0.034920664 | NaN |
| HNRNPA1L2 | -2.205641473 | 1.045822706 | 0.034944467 | NaN |
| PCGF6 | -2.202895033 | 1.04564565 | 0.035140827 | NaN |
| MORC2-AS1 | -2.205713321 | 1.047402914 | 0.035214075 | NaN |
| FAM78A | -2.204400611 | 1.046849815 | 0.035226355 | NaN |
| TRAPPC2 | -2.200285298 | 1.045977367 | 0.035416083 | NaN |
| ZDHHC8 | -2.198821018 | 1.045832586 | 0.035513013 | NaN |
| RDH13 | -2.196910171 | 1.045950071 | 0.035693951 | NaN |
| ZNF526 | -2.197090316 | 1.046058516 | 0.035697954 | NaN |
| OTUD6B | -2.195180466 | 1.04565658 | 0.035787632 | NaN |
| ERCC6 | -2.193926601 | 1.045707783 | 0.03590247 | NaN |
| LOC100505761 | -2.194336743 | 1.046335712 | 0.035979131 | NaN |
| TESK2 | -2.190937746 | 1.045788129 | 0.036170005 | NaN |
| CMC4 | -2.194008241 | 1.047515547 | 0.036216574 | NaN |
| APBA2 | -2.194160435 | 1.047750478 | 0.03624545 | NaN |
| C1orf131 | -2.188741048 | 1.046525949 | 0.036489083 | NaN |
| ARMC9 | -2.188616804 | 1.046846133 | 0.036557056 | NaN |
| COMMD8 | -2.189764226 | 1.047413961 | 0.036560458 | NaN |
| PKDCC | -2.186861951 | 1.046134404 | 0.036579945 | NaN |
| TCEAL7 | -2.188390164 | 1.047077102 | 0.036617884 | NaN |
| SNORA57 | -2.188875383 | 1.047650171 | 0.036679019 | NaN |
| NDOR1 | -2.186870521 | 1.046752684 | 0.036690169 | NaN |
| BTD | -2.190366672 | 1.048491136 | 0.036701826 | NaN |
| ZNF568 | -2.184029437 | 1.046528774 | 0.036894709 | NaN |
| SRRM3 | 2.161821837 | 1.036326196 | 0.036974656 | NaN |
| RFC5 | -2.184755786 | 1.047441061 | 0.036996465 | NaN |
| D2HGDH | -2.183606478 | 1.048449676 | 0.037278548 | NaN |
| PHKA1 | -2.178342748 | 1.046250317 | 0.037338141 | NaN |
| SERAC1 | -2.178389774 | 1.046315843 | 0.037345945 | NaN |
| FER | -2.178970379 | 1.046637566 | 0.037353732 | NaN |
| LOC100268168 | 2.085014555 | 1.001779017 | 0.03740537 | NaN |
| PIP5K1B | -2.181906453 | 1.048534117 | 0.037442029 | NaN |
| NCAPD3 | -2.176126075 | 1.04622543 | 0.037527534 | NaN |
| HSD17B6 | -2.174894681 | 1.046269093 | 0.037643604 | NaN |
| TYSND1 | -2.177003376 | 1.047421175 | 0.037668737 | NaN |
| PCDH10 | -2.177009653 | 1.047831692 | 0.037743179 | NaN |
| TMEM237 | -2.177939099 | 1.048336083 | 0.037753598 | NaN |
| GRIP1 | 2.094505314 | 1.008501273 | 0.037815457 | NaN |
| PAX8 | -2.17196649 | 1.047054032 | 0.038045935 | NaN |
| SLC35E2 | -2.170784514 | 1.046571748 | 0.038062036 | NaN |
| CLECL1 | 2.04169561 | 0.984503561 | 0.038094858 | NaN |
| HEATR6 | -2.169678605 | 1.047066199 | 0.038251421 | NaN |
| MSH2 | -2.166814084 | 1.047159856 | 0.038524554 | NaN |
| SEC14L4 | 2.175490478 | 1.051655241 | 0.038580383 | NaN |
| PARG | -2.164604653 | 1.047139474 | 0.038719096 | NaN |
| HES7 | 2.128066428 | 1.029641344 | 0.038752665 | NaN |
| EFS | -2.169008652 | 1.04950666 | 0.038763039 | NaN |
| FGGY | -2.165652629 | 1.048059865 | 0.038795972 | NaN |
| TIGD6 | -2.164126922 | 1.047738287 | 0.038873596 | NaN |
| ABHD8 | -2.161569508 | 1.047268246 | 0.039017006 | NaN |
| RALGPS1 | -2.165954976 | 1.049858209 | 0.039103806 | NaN |
| LRRC14 | -2.160766249 | 1.047580862 | 0.039148291 | NaN |
| RTTN | -2.162352566 | 1.048719809 | 0.039217512 | NaN |
| FAM184B | -2.157979606 | 1.046757184 | 0.0392472 | NaN |
| CDK8 | -2.159112785 | 1.047640657 | 0.039309835 | NaN |
| RAD9A | -2.153303161 | 1.046922116 | 0.039706042 | NaN |
| MYO9A | -2.15301796 | 1.047372565 | 0.039817504 | NaN |
| FAAH | -2.15219934 | 1.047090194 | 0.03983945 | NaN |
| KCNC4 | -2.155842795 | 1.050393312 | 0.040129366 | NaN |
| ZNF347 | -2.147721025 | 1.047249984 | 0.040284497 | NaN |
| TMC4 | -2.148304493 | 1.047583943 | 0.040293929 | NaN |
| BTNL3 | 2.111199588 | 1.0296389 | 0.040322763 | NaN |
| CDAN1 | -2.147019111 | 1.047152365 | 0.040331196 | NaN |
| GRAMD2 | -2.151233013 | 1.049753641 | 0.040435314 | NaN |
| GNA11 | -2.148134397 | 1.048636308 | 0.040510759 | NaN |
| PDE10A | -2.151168393 | 1.050124826 | 0.04051218 | NaN |
| PRR5 | -2.151103932 | 1.050557612 | 0.040600874 | NaN |
| SPSB2 | -2.143837219 | 1.047327081 | 0.040661958 | NaN |
| LRRC37B | -2.143383953 | 1.047190493 | 0.040678246 | NaN |
| TEFM | -2.143878139 | 1.047570692 | 0.040704883 | NaN |
| RABEP2 | -2.145790624 | 1.048994211 | 0.040798739 | NaN |
| TCTN1 | -2.148254524 | 1.050275411 | 0.040813451 | NaN |
| HPR | 2.149146458 | 1.050759033 | 0.040822571 | NaN |
| WDFY4 | -2.144483081 | 1.048863016 | 0.040896402 | NaN |
| FAM184A | -2.136726835 | 1.047388909 | 0.041345242 | NaN |
| PUS10 | -2.134734476 | 1.047441407 | 0.041545265 | NaN |
| ZNF880 | -2.134481985 | 1.047441414 | 0.041569378 | NaN |
| KLHL8 | -2.134056562 | 1.047645587 | 0.041649803 | NaN |
| ZNF16 | -2.130973779 | 1.048022196 | 0.042019309 | NaN |
| GTF2E1 | -2.128372234 | 1.047607693 | 0.042189095 | NaN |
| ZNF236 | -2.127994712 | 1.047917933 | 0.042286621 | NaN |
| CLDN15 | -2.132987894 | 1.050417624 | 0.042294634 | NaN |
| WDR27 | -2.126627674 | 1.047636673 | 0.042363795 | NaN |
| SLC9A7 | -2.131352634 | 1.050740581 | 0.042516486 | NaN |
| IQCG | -2.125105456 | 1.047709476 | 0.042526103 | NaN |
| VMO1 | -2.124959326 | 1.04841663 | 0.042680089 | NaN |
| KLF8 | -2.123804301 | 1.04797613 | 0.042705693 | NaN |
| EBF4 | -2.125160672 | 1.049375121 | 0.04285014 | NaN |
| TMEM233 | -2.12657387 | 1.050782318 | 0.042990674 | NaN |
| ZNF876P | 1.941768773 | 0.960612394 | 0.043239788 | NaN |
| ELP4 | -2.12096092 | 1.049279211 | 0.043243509 | NaN |
| CHEK2 | -2.118103185 | 1.047980975 | 0.043266567 | NaN |
| CCDC151 | 1.960649393 | 0.970418849 | 0.043340295 | NaN |
| NEK4 | -2.115716608 | 1.048089429 | 0.043524515 | NaN |
| MARK1 | -2.1162988 | 1.048463369 | 0.043541646 | NaN |
| SULT1A2 | -2.118458209 | 1.049887939 | 0.043612658 | NaN |
| ADAMTS7 | -2.119665453 | 1.050738644 | 0.043663185 | NaN |
| DBF4 | -2.113593008 | 1.048627871 | 0.043843948 | NaN |
| ETAA1 | -2.110766349 | 1.048461366 | 0.044093199 | NaN |
| PRODH | -2.114341624 | 1.050483312 | 0.044142806 | NaN |
| HAUS8 | -2.110312887 | 1.049029023 | 0.044253461 | NaN |
| DHRS11 | -2.112635993 | 1.050500355 | 0.044317436 | NaN |
| KLHL28 | -2.107005098 | 1.048164659 | 0.04441159 | NaN |
| ZDBF2 | -2.105763969 | 1.048375951 | 0.044579973 | NaN |
| TAMM41 | -2.105085723 | 1.048237111 | 0.044620426 | NaN |
| ZNF525 | -2.107138088 | 1.049316035 | 0.044632002 | NaN |
| MCM2 | -2.1049937 | 1.04841325 | 0.044665606 | NaN |
| GDF11 | -2.104444734 | 1.049378945 | 0.044918246 | NaN |
| FRAT2 | -2.106155832 | 1.050402351 | 0.04495296 | NaN |
| GZMK | -2.106341886 | 1.051268939 | 0.045110943 | NaN |
| ZNF773 | -2.099204939 | 1.048449427 | 0.045263277 | NaN |
| EEF1E1 | -2.098857716 | 1.048442881 | 0.045297549 | NaN |
| SNORA25 | -2.101165205 | 1.049834143 | 0.045346516 | NaN |
| ANKRD6 | -2.100051256 | 1.049532004 | 0.045398786 | NaN |
| TMEM234 | -2.098454963 | 1.048953539 | 0.045443893 | NaN |
| LRMP | -2.099543068 | 1.049814866 | 0.045509179 | NaN |
| UNC5C | -2.101397447 | 1.050775329 | 0.045516011 | NaN |
| NOXRED1 | 2.105617711 | 1.053132938 | 0.045566764 | NaN |
| LOC100128770 | 2.035248776 | 1.019247493 | 0.045845274 | NaN |
| NBPF1 | -2.094018163 | 1.048723581 | 0.045854487 | NaN |
| FANCE | -2.092690981 | 1.048592862 | 0.045965112 | NaN |
| ZNF780B | -2.093043002 | 1.048892552 | 0.045990668 | NaN |
| GLMN | -2.09219842 | 1.048657783 | 0.046029762 | NaN |
| CETN3 | -2.093519854 | 1.049502619 | 0.046067605 | NaN |
| TMEM180 | -2.092595046 | 1.049091474 | 0.046078493 | NaN |
| SARS2 | -2.095629789 | 1.051253844 | 0.04621138 | NaN |
| CCSAP | -2.088195899 | 1.048652564 | 0.046446444 | NaN |
| FAM72A | 2.089835754 | 1.049749693 | 0.046503501 | NaN |
| USP32P2 | -2.091422814 | 1.05061148 | 0.046516963 | NaN |
| TBC1D19 | -2.090188113 | 1.050156237 | 0.046551383 | NaN |
| HMBOX1 | -2.090188113 | 1.050156237 | 0.046551383 | NaN |
| PPP1R16A | -2.086013727 | 1.048710921 | 0.046687764 | NaN |
| TUB | -2.086909308 | 1.049195475 | 0.046694943 | NaN |
| INVS | -2.086402376 | 1.049463456 | 0.046804416 | NaN |
| SWT1 | -2.084657028 | 1.048781727 | 0.046845555 | NaN |
| ZBTB6 | -2.085435489 | 1.049207699 | 0.046852754 | NaN |
| PIGM | -2.083797006 | 1.048844097 | 0.046949474 | NaN |
| KIF21A | -2.085362439 | 1.049961062 | 0.047018545 | NaN |
| ZNF605 | -2.083109088 | 1.048850959 | 0.047023682 | NaN |
| PITPNM3 | -2.087697203 | 1.051830722 | 0.047164232 | NaN |
| ADAMTS9-AS2 | -2.081626454 | 1.048818936 | 0.04717409 | NaN |
| MRPS31P5 | -2.081977294 | 1.049758359 | 0.047334829 | NaN |
| CHST7 | -2.079725373 | 1.04899476 | 0.047413368 | NaN |
| FAM183A | 1.919081625 | 0.968432873 | 0.047519974 | NaN |
| NPFF | -2.077368942 | 1.049484782 | 0.047768977 | NaN |
| EPS8L1 | -2.077368942 | 1.049484782 | 0.047768977 | NaN |
| LYRM7 | -2.079590808 | 1.050691332 | 0.047786796 | NaN |
| AMN | 2.063936691 | 1.042947556 | 0.047822108 | NaN |
| EPM2A | -2.079212677 | 1.051193036 | 0.047933767 | NaN |
| SLC35F3 | 2.082655689 | 1.053401441 | 0.048032935 | NaN |
| MST1R | -2.079627009 | 1.052041582 | 0.048069486 | NaN |
| FAM45B | 2.066752526 | 1.045953294 | 0.04816032 | NaN |
| HSD17B13 | 2.066752526 | 1.045953294 | 0.04816032 | NaN |
| RUNX2 | -2.07144475 | 1.049072612 | 0.048319409 | NaN |
| ANG | -2.070740558 | 1.049689986 | 0.048527892 | NaN |
| TAF5 | -2.070258892 | 1.049536819 | 0.048547393 | NaN |
| YEATS4 | -2.069366978 | 1.049949515 | 0.048732975 | NaN |
| ZMAT5 | -2.068540265 | 1.04966777 | 0.048762563 | NaN |
| ZBTB41 | -2.067517024 | 1.049228599 | 0.048779778 | NaN |
| SCARNA1 | 1.970946043 | 1.000446518 | 0.048830764 | NaN |
| B3GALT4 | -2.067538265 | 1.050166605 | 0.048979316 | NaN |
| SGSM1 | -2.064523001 | 1.049595875 | 0.049186735 | NaN |
| PREX2 | -2.066668721 | 1.050738987 | 0.04919801 | NaN |
| PKI55 | -2.063718432 | 1.04946023 | 0.049245843 | NaN |
| PLCD4 | -2.063305857 | 1.04928836 | 0.049254049 | NaN |
| C16orf87 | -2.066431331 | 1.050970307 | 0.049274028 | NaN |
| ZNF674-AS1 | -2.062261567 | 1.04988851 | 0.049499113 | NaN |
| CLPB | -2.062644661 | 1.050276418 | 0.049540939 | NaN |
| SPRR1B | 2.022260927 | 1.029819793 | 0.049564465 | NaN |
| KIAA1704 | -2.060530071 | 1.049481294 | 0.049602093 | NaN |
| ZNF707 | -2.064653495 | 1.0518515 | 0.049660647 | NaN |
| FAM149A | -2.062445787 | 1.050787066 | 0.04967374 | NaN |
| ZNF764 | -2.058395797 | 1.049403141 | 0.049821665 | NaN |
| ZMAT1 | -2.063028569 | 1.051863711 | 0.04984312 | NaN |
| CARD11 | -2.058674182 | 1.049841884 | 0.049886329 | NaN |
