# Supplementary material for: Identification of SLC15A3 as a shared prognostic biomarker for AML and COVID-19 via integrative bioinformatics, machine learning and Mendelian randomization
Source: Front Oncol. 2026 Oct 14;16:1911550. doi: 10.3389/fonc.2026.1911550 (PMC13713349; doi:10.3389/fonc.2026.1911550)
Supplement: Supplementary file 3 [file Table3.doc]

**Supplementary Table S3 The differentially expressed mRNAs of GTEX.TCGA**

| id | logFC | AveExpr | t | P.Value | adj.P.Val | B |
| --- | --- | --- | --- | --- | --- | --- |
| RN7SL1 | -2.917575 | 0.67006887 | -353.0496614 | 2.13E-277 | 5.06E-274 | 622.2490842 |
| AC026464.4 | -2.129411572 | 0.491650848 | -300.6979801 | 1.00E-263 | 1.19E-260 | 592.2058898 |
| AC012321.1 | -2.136877331 | 0.490827058 | -256.2562172 | 4.14E-250 | 3.28E-247 | 561.870998 |
| AC068831.4 | -2.334362377 | 0.536168012 | -238.3549339 | 6.00E-244 | 3.56E-241 | 548.0362007 |
| OVCA2 | -2.098657936 | 0.482052197 | -233.8450021 | 2.53E-242 | 1.20E-239 | 544.3783741 |
| MT-TP | 3.230547001 | 2.824174899 | 232.0041971 | 1.19E-241 | 4.71E-239 | 542.8641057 |
| TOMM6 | -2.424777325 | 0.556926546 | -224.2222842 | 9.46E-239 | 3.21E-236 | 536.3209356 |
| AL138478.1 | -1.999292162 | 0.459238626 | -206.7491994 | 7.44E-232 | 2.21E-229 | 520.7258352 |
| AC234031.1 | -1.876071673 | 0.461569006 | -194.6690453 | 9.67E-227 | 2.55E-224 | 509.1257764 |
| AL355816.2 | -1.811551512 | 0.416134905 | -194.0098428 | 1.88E-226 | 4.46E-224 | 508.4716266 |
| XBP1 | -2.414038241 | 0.55446094 | -192.3628972 | 9.93E-226 | 2.15E-223 | 506.8272836 |
| ARPIN-AP3S2 | -1.943718629 | 0.463831172 | -191.8444229 | 1.68E-225 | 3.33E-223 | 506.3066383 |
| GIMAP1-GIMAP5 | -1.991703606 | 0.459352579 | -185.3535022 | 1.41E-222 | 2.57E-220 | 499.6637417 |
| TREX1 | -2.18115216 | 0.500992197 | -181.5709669 | 7.90E-221 | 1.34E-218 | 495.6820155 |
| AC011558.1 | -2.65660864 | 0.610153124 | -176.0607224 | 3.25E-218 | 5.15E-216 | 489.7275189 |
| INO80B-WBP1 | -1.843439533 | 0.47323339 | -172.9452861 | 1.06E-216 | 1.58E-214 | 486.2763501 |
| BOLA2 | -1.74259517 | 0.400303092 | -171.9127361 | 3.42E-216 | 4.78E-214 | 485.1185669 |
| AC015674.1 | -2.062857302 | 0.473832663 | -169.0963999 | 8.59E-215 | 1.13E-212 | 481.9243849 |
| AC015688.6 | -1.791763945 | 0.411591842 | -167.136557 | 8.36E-214 | 1.05E-211 | 479.6695916 |
| AC087292.1 | -1.819234016 | 0.417898746 | -162.9700887 | 1.15E-211 | 1.37E-209 | 474.7857313 |
| ISY1-RAB43 | -1.903222528 | 0.446573114 | -162.450721 | 2.15E-211 | 2.43E-209 | 474.1680977 |
| AL583722.2 | -2.041936799 | 0.469029487 | -157.2010321 | 1.30E-208 | 1.41E-206 | 467.8107187 |
| AC124068.1 | -2.213869175 | 0.508503757 | -156.0803195 | 5.25E-208 | 5.43E-206 | 466.4257986 |
| AC006027.1 | -1.773242086 | 0.407339374 | -155.0855632 | 1.83E-207 | 1.81E-205 | 465.1881052 |
| RPL12P16 | -1.897503368 | 0.43586875 | -152.2112998 | 7.00E-206 | 6.66E-204 | 461.5664493 |
| SLX1A | -2.274818605 | 0.622541965 | -148.9605918 | 4.70E-204 | 4.29E-202 | 457.3866182 |
| RPL32P29 | -2.001118474 | 0.459657932 | -148.5418769 | 8.13E-204 | 7.16E-202 | 456.8415734 |
| CMC4 | -1.59348308 | 0.387407602 | -147.4053644 | 3.63E-203 | 3.08E-201 | 455.3543534 |
| AL445363.3 | -2.039297525 | 0.50324343 | -145.6597728 | 3.69E-202 | 3.03E-200 | 453.0475459 |
| AC007068.1 | -1.928256182 | 0.442929345 | -143.8447092 | 4.24E-201 | 3.36E-199 | 450.6193499 |
| AC018557.1 | -1.672370058 | 0.38417998 | -138.7060924 | 5.04E-198 | 3.86E-196 | 443.5748551 |
| RPL41P5 | 2.560802003 | 2.308197886 | 136.90143 | 6.44E-197 | 4.78E-195 | 441.0388097 |
| SMIM11B | -1.620611129 | 0.390804663 | -136.0872537 | 2.05E-196 | 1.48E-194 | 439.8837282 |
| PTPRCAP | -2.585071452 | 0.593728769 | -135.8263289 | 2.98E-196 | 2.09E-194 | 439.5120921 |
| AC005523.2 | -1.739847391 | 0.399672225 | -134.6933707 | 1.52E-195 | 1.03E-193 | 437.8901179 |
| AL732372.3 | -2.426506091 | 0.649123891 | -134.338536 | 2.54E-195 | 1.68E-193 | 437.3793296 |
| SULT1A4 | -2.0754781 | 0.507030239 | -131.5400734 | 1.52E-193 | 9.76E-192 | 433.3031887 |
| AC024267.6 | -2.104183956 | 0.522302056 | -131.0218245 | 3.27E-193 | 2.02E-191 | 432.5388643 |
| SLX1B-SULT1A4 | -1.762899461 | 0.433420238 | -131.0136503 | 3.31E-193 | 2.02E-191 | 432.5267847 |
| AC020978.5 | -1.777728443 | 0.409477159 | -129.7295681 | 2.25E-192 | 1.33E-190 | 430.6198479 |
| ICAM4 | -1.728926324 | 0.397164837 | -129.4687897 | 3.32E-192 | 1.92E-190 | 430.230286 |
| AC093484.3 | -2.453307873 | 0.563476927 | -128.801086 | 9.07E-192 | 5.13E-190 | 429.2292779 |
| RPPH1 | -1.958240095 | 0.449813407 | -125.6316554 | 1.14E-189 | 6.32E-188 | 424.4063777 |
| AC130304.1 | -2.06571687 | 0.474489197 | -125.3995955 | 1.64E-189 | 8.85E-188 | 424.0485274 |
| RNA5-8SN2 | -2.935379596 | 0.721359051 | -123.4583262 | 3.38E-188 | 1.79E-186 | 421.0290189 |
| AC009630.3 | -1.933054333 | 0.444030961 | -119.9315254 | 9.35E-186 | 4.83E-184 | 415.4211446 |
| ST20-MTHFS | -1.752337412 | 0.429373065 | -119.884171 | 1.01E-185 | 5.10E-184 | 415.3447442 |
| AC034102.4 | -1.657961473 | 0.380871886 | -119.8688319 | 1.04E-185 | 5.12E-184 | 415.3199901 |
| AC011511.4 | -1.89618902 | 0.468838563 | -119.3231759 | 2.51E-185 | 1.22E-183 | 414.437374 |
| SNORD62B | 2.417198308 | 2.197564427 | 118.1565762 | 1.68E-184 | 8.00E-183 | 412.5369254 |
| FAM156A | -1.635984545 | 0.486185008 | -116.4700484 | 2.73E-183 | 1.27E-181 | 409.7565256 |
| AC099548.2 | -1.867200999 | 0.530777249 | -115.9988767 | 5.99E-183 | 2.74E-181 | 408.9726644 |
| AC006449.7 | -2.084181449 | 0.478728513 | -115.7465727 | 9.13E-183 | 4.09E-181 | 408.5516312 |
| NPIPA9 | -2.185986562 | 0.582852478 | -114.9876305 | 3.26E-182 | 1.44E-180 | 407.279676 |
| SLX1A-SULT1A3 | -1.858097603 | 0.572582419 | -114.7994134 | 4.48E-182 | 1.94E-180 | 406.9629539 |
| AL160408.2 | -2.211866241 | 0.508043899 | -113.3785451 | 5.00E-181 | 2.12E-179 | 404.5553792 |
| RPS10-NUDT3 | -1.79847905 | 0.477305511 | -111.3647534 | 1.60E-179 | 6.68E-178 | 401.0918578 |
| AC018638.8 | 2.428149247 | 2.20600112 | 110.6490449 | 5.58E-179 | 2.29E-177 | 399.8460875 |
| TBC1D3B | -1.587456392 | 0.432642134 | -109.4789714 | 4.36E-178 | 1.76E-176 | 397.7923281 |
| AC010653.2 | -1.56530302 | 0.397584032 | -109.3277628 | 5.70E-178 | 2.26E-176 | 397.5253529 |
| TNFSF12-TNFSF13 | -1.676225778 | 0.430815839 | -108.4144057 | 2.88E-177 | 1.12E-175 | 395.9049912 |
| RNA5-8SN3 | -2.92730297 | 0.72758135 | -108.3547727 | 3.21E-177 | 1.23E-175 | 395.7987338 |
| SLX1B | -1.991440541 | 0.564479273 | -105.1099496 | 1.14E-174 | 4.31E-173 | 389.9289778 |
| RASA4B | -1.733647875 | 0.479347225 | -103.9133432 | 1.04E-173 | 3.87E-172 | 387.7195988 |
| SULT1A3 | -1.952480436 | 0.624691672 | -101.9905827 | 3.83E-172 | 1.40E-170 | 384.1169918 |
| AC017116.1 | -1.817502513 | 0.417501207 | -99.53082216 | 4.24E-170 | 1.53E-168 | 379.4106347 |
| POLR2F | -2.01345313 | 0.622073509 | -97.77282631 | 1.31E-168 | 4.65E-167 | 375.9772597 |
| AC008738.7 | -1.715993755 | 0.394195625 | -97.18864126 | 4.16E-168 | 1.45E-166 | 374.8230818 |
| AC138969.1 | -2.093635074 | 0.66153678 | -97.14420689 | 4.55E-168 | 1.57E-166 | 374.7350177 |
| AC008738.3 | -1.94005538 | 0.445638345 | -96.71713039 | 1.06E-167 | 3.60E-166 | 373.8866034 |
| DEFA1 | -3.181785845 | 0.734728245 | -96.23494208 | 2.78E-167 | 9.30E-166 | 372.9243359 |
| DEFA1B | -3.224063751 | 0.742506336 | -95.56719467 | 1.06E-166 | 3.50E-165 | 371.5840298 |
| DUSP13 | -1.683205896 | 0.430971408 | -95.42924587 | 1.40E-166 | 4.56E-165 | 371.3060097 |
| SNORD14A | 2.285108511 | 2.095801369 | 94.26552677 | 1.48E-165 | 4.76E-164 | 368.9450869 |
| SNORD63B | 2.40354432 | 2.187045283 | 93.83603897 | 3.57E-165 | 1.13E-163 | 368.0666355 |
| MIR590 | 2.309779307 | 2.114807951 | 93.25552673 | 1.18E-164 | 3.68E-163 | 366.8731 |
| BLOC1S5-TXNDC5 | -1.587800222 | 0.418862717 | -92.25309705 | 9.39E-164 | 2.90E-162 | 364.7951318 |
| DNAJC25-GNG10 | -1.98017053 | 0.457452583 | -90.77817472 | 2.07E-162 | 6.32E-161 | 361.6978369 |
| AC129492.8 | -1.663620062 | 0.382171052 | -89.6064224 | 2.51E-161 | 7.55E-160 | 359.2024964 |
| RNA5-8SN1 | -2.893704004 | 0.753466267 | -89.29868928 | 4.86E-161 | 1.44E-159 | 358.5419574 |
| PTRH1 | -1.60056887 | 0.481592807 | -88.85429284 | 1.26E-160 | 3.71E-159 | 357.5842094 |
| RF00002 | -1.90928373 | 0.499222301 | -88.77813011 | 1.49E-160 | 4.32E-159 | 357.4196049 |
| RNU6-125P | 2.111581246 | 1.962114547 | 88.53936716 | 2.50E-160 | 7.15E-159 | 356.9027061 |
| LIMD1-AS1 | -1.618330363 | 0.538031954 | -87.93500078 | 9.29E-160 | 2.63E-158 | 355.5883146 |
| HIST2H4A | -1.825334106 | 0.570062768 | -87.50115853 | 2.40E-159 | 6.70E-158 | 354.6394355 |
| AL513534.1 | 2.198189353 | 2.02883814 | 86.17282582 | 4.49E-158 | 1.24E-156 | 351.7059118 |
| RNU6-1016P | 2.247222092 | 2.066613362 | 83.73868153 | 1.08E-155 | 2.95E-154 | 346.2163753 |
| AC100835.2 | -1.979223239 | 0.454630965 | -83.58295203 | 1.54E-155 | 4.16E-154 | 345.8600117 |
| AC010132.3 | -1.646739378 | 0.424551187 | -83.31275724 | 2.86E-155 | 7.64E-154 | 345.2402088 |
| CXCR5 | -1.638731291 | 0.49167668 | -83.10675043 | 4.59E-155 | 1.21E-153 | 344.7663606 |
| MIR647 | 2.270899725 | 2.084854804 | 82.6365339 | 1.36E-154 | 3.55E-153 | 343.6805881 |
| ALDH1A2 | -1.521164766 | 0.461843125 | -82.52804664 | 1.75E-154 | 4.51E-153 | 343.4292472 |
| RNY4P10 | 2.087065563 | 1.943227465 | 82.45682462 | 2.06E-154 | 5.26E-153 | 343.2640706 |
| AP001267.1 | -1.651522044 | 0.587557658 | -81.96007932 | 6.53E-154 | 1.65E-152 | 342.1082451 |
| NECAB2 | -1.743406405 | 0.443409288 | -81.14725689 | 4.38E-153 | 1.10E-151 | 340.2025453 |
| CICP27 | -1.628584945 | 0.433161649 | -80.82050903 | 9.46E-153 | 2.34E-151 | 339.4313615 |
| NUDT4P2 | -1.649186278 | 0.415990541 | -80.70841205 | 1.23E-152 | 3.02E-151 | 339.1661112 |
| SNORD12B | 2.384964477 | 2.17273122 | 79.73978953 | 1.23E-151 | 2.99E-150 | 336.8594514 |
| AC011472.3 | -2.116909937 | 0.486242707 | -79.45437841 | 2.44E-151 | 5.86E-150 | 336.1747179 |
| NUDT4B | -1.649423371 | 0.415807882 | -79.44219509 | 2.51E-151 | 5.97E-150 | 336.1454369 |
| AC125611.3 | -1.760810477 | 0.503100688 | -79.09758883 | 5.76E-151 | 1.35E-149 | 335.3154598 |
| TOP3B | -1.628666492 | 0.762178045 | -78.98994366 | 7.46E-151 | 1.74E-149 | 335.0554978 |
| AC009084.3 | -1.663197156 | 0.382073956 | -78.6087722 | 1.88E-150 | 4.33E-149 | 334.1322731 |
| RNU6-595P | 2.185330267 | 2.018931395 | 78.00639088 | 8.12E-150 | 1.86E-148 | 332.6646067 |
| AD000671.1 | -1.702295327 | 0.425995626 | -77.78434967 | 1.40E-149 | 3.16E-148 | 332.1209173 |
| RNU6-1053P | 1.989491764 | 1.868055814 | 77.57708681 | 2.32E-149 | 5.20E-148 | 331.6120917 |
| H2BFS | -1.760652792 | 0.487109886 | -77.42373966 | 3.38E-149 | 7.51E-148 | 331.2348024 |
| SLC22A1 | -1.599245795 | 0.469974684 | -77.00545294 | 9.49E-149 | 2.09E-147 | 330.2020747 |
| AC020913.3 | 1.972309909 | 1.854818772 | 76.78828379 | 1.62E-148 | 3.54E-147 | 329.6638101 |
| BOLA2B | -1.842830899 | 0.634570766 | -76.17136241 | 7.53E-148 | 1.63E-146 | 328.1268871 |
| AL355310.2 | -1.805219102 | 0.414681036 | -76.06148499 | 9.90E-148 | 2.12E-146 | 327.8519241 |
| AC012441.1 | -1.777972339 | 0.503877516 | -75.91838837 | 1.42E-147 | 3.01E-146 | 327.4932714 |
| AL627309.5 | -2.110973837 | 0.728192077 | -75.87286154 | 1.59E-147 | 3.33E-146 | 327.3790312 |
| NPIPA7 | -1.677472573 | 0.405046699 | -75.87017728 | 1.60E-147 | 3.33E-146 | 327.3722936 |
| MIR4312 | 2.183544945 | 2.017555968 | 75.67532306 | 2.61E-147 | 5.38E-146 | 326.8826018 |
| AL669918.1 | -1.610546653 | 0.401382655 | -75.08085025 | 1.17E-146 | 2.39E-145 | 325.3812703 |
| AC012615.3 | -1.835167239 | 0.554925385 | -75.02001457 | 1.36E-146 | 2.76E-145 | 325.227002 |
| GP1BB | -2.037633912 | 0.468041579 | -74.79604556 | 2.40E-146 | 4.83E-145 | 324.658044 |
| IGLC1 | -2.598793261 | 0.596879185 | -74.5306829 | 4.71E-146 | 9.41E-145 | 323.9818607 |
| ATP2C2 | -1.517673019 | 0.478401914 | -74.25884661 | 9.43E-146 | 1.87E-144 | 323.2868381 |
| AC087393.2 | -1.713637248 | 0.39365459 | -74.17591649 | 1.17E-145 | 2.29E-144 | 323.0743304 |
| AL669831.3 | -1.62933796 | 0.406952586 | -74.15675556 | 1.22E-145 | 2.38E-144 | 323.025199 |
| GTF2IP1 | -1.895714883 | 0.800236072 | -73.78514323 | 3.18E-145 | 6.13E-144 | 322.0699741 |
| AL662899.2 | -1.739887899 | 0.551386719 | -73.54177884 | 5.94E-145 | 1.14E-143 | 321.4419662 |
| HIST2H2AA3 | -2.777572334 | 0.80832864 | -73.50630245 | 6.51E-145 | 1.24E-143 | 321.3502562 |
| AL353813.1 | -1.827295329 | 0.419749557 | -72.84380707 | 3.62E-144 | 6.84E-143 | 319.6299951 |
| KIAA1324 | -1.679417781 | 0.575225699 | -72.82520103 | 3.80E-144 | 7.12E-143 | 319.5814713 |
| ADORA2A | -1.854431161 | 0.828825763 | -71.70962055 | 7.09E-143 | 1.32E-141 | 316.6507284 |
| KREMEN1 | -1.920446356 | 0.441136273 | -71.66602121 | 7.95E-143 | 1.46E-141 | 316.5353276 |
| HIST2H4B | -1.706617038 | 0.439332466 | -71.07911035 | 3.77E-142 | 6.89E-141 | 314.9754677 |
| SPOCD1 | -1.552854811 | 0.39610786 | -70.65100719 | 1.18E-141 | 2.14E-140 | 313.8301027 |
| MMP23B | -1.746370969 | 0.453395905 | -70.30153106 | 3.02E-141 | 5.43E-140 | 312.8903084 |
| CBS | -1.669657886 | 0.442238478 | -69.04039981 | 9.22E-140 | 1.65E-138 | 309.4625055 |
| RNU6-850P | 2.127143299 | 1.97410368 | 68.96505337 | 1.13E-139 | 2.01E-138 | 309.2558793 |
| AC008763.3 | -2.227605967 | 0.574557392 | -68.89595731 | 1.37E-139 | 2.41E-138 | 309.06621 |
| ATP6V1G2-DDX39B | -1.640336651 | 0.397517751 | -68.5364408 | 3.68E-139 | 6.42E-138 | 308.0764857 |
| AD000671.2 | -1.492642538 | 0.540600127 | -68.51912005 | 3.86E-139 | 6.69E-138 | 308.0286818 |
| HNRNPUL2-BSCL2 | -1.629063583 | 0.798854781 | -68.36968341 | 5.82E-139 | 1.00E-137 | 307.6157836 |
| RNASE4 | -1.606602443 | 0.461618756 | -68.3564256 | 6.04E-139 | 1.03E-137 | 307.5791116 |
| EIF3CL | -1.733252176 | 0.702458186 | -68.24914628 | 8.12E-139 | 1.38E-137 | 307.2821276 |
| PGA3 | -1.815784914 | 0.433109183 | -68.17687043 | 9.92E-139 | 1.67E-137 | 307.0818014 |
| C4B | -1.782834193 | 0.538793922 | -67.95020712 | 1.86E-138 | 3.11E-137 | 306.4522869 |
| RNU6-611P | 1.969812612 | 1.852894834 | 67.00556066 | 2.60E-137 | 4.32E-136 | 303.8076781 |
| CBWD3 | -1.558154901 | 0.630842298 | -66.38625013 | 1.49E-136 | 2.46E-135 | 302.0551752 |
| AL132780.3 | -1.826764309 | 0.41962764 | -65.96859941 | 4.89E-136 | 8.02E-135 | 300.8648176 |
| MIR570 | 2.147473197 | 1.989765999 | 65.36152693 | 2.78E-135 | 4.53E-134 | 299.1221922 |
| AC073172.1 | -1.717853414 | 0.414400999 | -65.2930026 | 3.39E-135 | 5.48E-134 | 298.9245593 |
| MIR3198-1 | 2.068346634 | 1.928806249 | 65.08945619 | 6.09E-135 | 9.78E-134 | 298.3363834 |
| EEF1G | -2.500332614 | 1.092368067 | -64.98870632 | 8.15E-135 | 1.30E-133 | 298.0446291 |
| HIST2H2AA4 | -2.622697473 | 0.790267604 | -64.83566599 | 1.27E-134 | 2.01E-133 | 297.6006576 |
| MIR5094 | 1.720433152 | 1.660770863 | 64.63796454 | 2.25E-134 | 3.54E-133 | 297.0257025 |
| RNU6-118P | 2.125489701 | 1.972829734 | 64.0626738 | 1.21E-133 | 1.88E-132 | 295.3434523 |
| LINC01270 | -1.815019247 | 0.531777131 | -63.61898658 | 4.44E-133 | 6.85E-132 | 294.0365886 |
| C4A | -1.720652558 | 0.590034605 | -62.59937195 | 9.16E-132 | 1.40E-130 | 291.0016086 |
| COMMD3-BMI1 | -1.653350397 | 0.441680201 | -62.58623107 | 9.52E-132 | 1.45E-130 | 290.962201 |
| AC125494.1 | -1.499764896 | 0.733787116 | -62.23621197 | 2.72E-131 | 4.12E-130 | 289.9097672 |
| KPNA2P3 | -1.811505953 | 0.416124446 | -62.04425317 | 4.85E-131 | 7.29E-130 | 289.3303047 |
| MT-TY | 2.504696436 | 2.264973699 | 61.97331689 | 6.00E-131 | 8.97E-130 | 289.1157593 |
| RNU6-415P | 2.095850061 | 1.949995114 | 61.80467224 | 9.99E-131 | 1.48E-129 | 288.6048017 |
| LINC02193 | -1.668759237 | 0.555185762 | -61.65984144 | 1.55E-130 | 2.29E-129 | 288.1649858 |
| AC234781.5 | -1.507453117 | 0.421187564 | -61.59856918 | 1.87E-130 | 2.74E-129 | 287.9786352 |
| FFAR3 | -1.710925363 | 0.438643175 | -61.55311769 | 2.14E-130 | 3.12E-129 | 287.8402927 |
| AC015813.2 | -1.503377341 | 0.449872375 | -61.54734276 | 2.18E-130 | 3.16E-129 | 287.8227087 |
| CRYAB | -1.638839982 | 0.478763175 | -61.40368342 | 3.37E-130 | 4.86E-129 | 287.3848012 |
| SNORA55 | 1.942446496 | 1.831811755 | 61.19986386 | 6.28E-130 | 8.99E-129 | 286.7619182 |
| RASA4 | -1.674298122 | 0.692029655 | -61.16002295 | 7.09E-130 | 1.01E-128 | 286.6399432 |
| AC146944.4 | -1.540065069 | 0.492572725 | -61.02472988 | 1.07E-129 | 1.51E-128 | 286.2251999 |
| BORCS8-MEF2B | -1.586771608 | 0.840194518 | -60.90686029 | 1.54E-129 | 2.15E-128 | 285.8631909 |
| BTNL8 | -1.860792492 | 0.552729782 | -60.86748683 | 1.74E-129 | 2.41E-128 | 285.7421235 |
| LINC00937 | -1.894466574 | 0.695093113 | -60.85464776 | 1.81E-129 | 2.49E-128 | 285.70263 |
| MIR4292 | 1.941205284 | 1.830855515 | 60.82774041 | 1.96E-129 | 2.69E-128 | 285.6198375 |
| NBPF10 | -1.37893791 | 0.723566657 | -60.64055525 | 3.49E-129 | 4.76E-128 | 285.0429622 |
| MIR186 | 2.010476713 | 1.884222789 | 60.19468433 | 1.38E-128 | 1.87E-127 | 283.6623624 |
| NT5DC4 | -1.50271249 | 0.505067569 | -59.91632467 | 3.28E-128 | 4.42E-127 | 282.7957678 |
| RNU2-2P | -1.878932779 | 0.431605094 | -59.766617 | 5.22E-128 | 7.01E-127 | 282.3281956 |
| SPSB3 | -1.930907879 | 1.001971226 | -59.63217633 | 7.94E-128 | 1.06E-126 | 281.9074074 |
| TEN1 | -1.62940288 | 0.742655794 | -59.36143932 | 1.85E-127 | 2.46E-126 | 281.0574295 |
| LUZP6 | -2.127742193 | 0.488729705 | -59.11558636 | 4.01E-127 | 5.30E-126 | 280.282554 |
| SNORA66 | 1.933785001 | 1.825138869 | 59.08520937 | 4.42E-127 | 5.80E-126 | 280.1866116 |
| AC007032.1 | -1.714322493 | 0.55013879 | -58.98020423 | 6.15E-127 | 8.03E-126 | 279.8546235 |
| MIR4513 | 1.875229543 | 1.780027266 | 58.86963159 | 8.72E-127 | 1.13E-125 | 279.5044597 |
| NHEJ1 | -1.316956112 | 0.763303611 | -58.81329792 | 1.04E-126 | 1.34E-125 | 279.3258343 |
| AC016596.2 | 2.049707596 | 1.914446582 | 58.3266569 | 4.88E-126 | 6.24E-125 | 277.7763604 |
| RGPD1 | -1.560795274 | 0.447122622 | -58.27662209 | 5.73E-126 | 7.28E-125 | 277.6163935 |
| SNORD6 | 2.017419565 | 1.88957162 | 58.02704551 | 1.27E-125 | 1.60E-124 | 276.8166316 |
| SNORD13E | 2.005120954 | 1.880096669 | 58.02685124 | 1.27E-125 | 1.60E-124 | 276.8160079 |
| LGALS9C | -1.685929999 | 0.52662778 | -57.82239274 | 2.45E-125 | 3.06E-124 | 276.1585312 |
| DBNDD2 | -1.639614879 | 0.701150028 | -57.81785047 | 2.49E-125 | 3.09E-124 | 276.1439011 |
| STARD5 | -1.406114952 | 0.789100794 | -57.81345314 | 2.52E-125 | 3.12E-124 | 276.1297368 |
| CATIP-AS1 | -1.613450597 | 0.562191481 | -57.76848055 | 2.91E-125 | 3.59E-124 | 275.9848202 |
| AL732372.2 | -2.021433577 | 0.948217388 | -57.75520511 | 3.04E-125 | 3.72E-124 | 275.9420231 |
| SNORA11F | 1.66365509 | 1.61702858 | 57.68292143 | 3.84E-125 | 4.68E-124 | 275.7088423 |
| CLEC1A | -1.472903483 | 0.491876384 | -57.66363027 | 4.08E-125 | 4.95E-124 | 275.6465667 |
| AL158207.2 | 1.892497739 | 1.816706526 | 57.47292943 | 7.55E-125 | 9.11E-124 | 275.0299457 |
| RNA5SP82 | 2.115378546 | 1.965040018 | 57.34727718 | 1.13E-124 | 1.36E-123 | 274.6226587 |
| AL354740.1 | -1.597865965 | 0.534244521 | -57.29072236 | 1.36E-124 | 1.62E-123 | 274.4390834 |
| KIR2DL3 | -1.734475927 | 0.510281677 | -57.02180735 | 3.26E-124 | 3.87E-123 | 273.5639807 |
| SNORD100 | 1.992819679 | 1.870619667 | 56.72211766 | 8.65E-124 | 1.02E-122 | 272.5843967 |
| AC108471.1 | 1.947829943 | 1.90489078 | 56.66699847 | 1.04E-123 | 1.22E-122 | 272.4037304 |
| SNORD117 | 1.977506803 | 1.858822502 | 56.10330698 | 6.59E-123 | 7.71E-122 | 270.5470915 |
| AC159540.2 | -1.586063032 | 0.440040811 | -56.00549834 | 9.10E-123 | 1.06E-121 | 270.2232559 |
| SNORA70G | 2.089631738 | 1.980654527 | 55.99144706 | 9.53E-123 | 1.10E-121 | 270.1766923 |
| MT-TL1 | 2.519425663 | 2.276321216 | 55.98516414 | 9.73E-123 | 1.12E-121 | 270.1558684 |
| AC241585.2 | -1.620667447 | 0.616890931 | -55.54583186 | 4.18E-122 | 4.80E-121 | 268.6946079 |
| CNTNAP3 | -1.719423682 | 0.462968414 | -55.47500254 | 5.29E-122 | 6.04E-121 | 268.4580674 |
| AC022966.1 | -2.068987152 | 0.475240027 | -55.36459271 | 7.64E-122 | 8.69E-121 | 268.088812 |
| MIR621 | 1.974964714 | 1.856864055 | 55.33855768 | 8.34E-122 | 9.43E-121 | 268.0016456 |
| PIK3R2 | -1.385367558 | 0.711581987 | -55.21784532 | 1.25E-121 | 1.41E-120 | 267.5970219 |
| MUSTN1 | -1.531882721 | 0.670856528 | -55.03853427 | 2.27E-121 | 2.55E-120 | 266.9945352 |
| TNNC1 | -1.483028363 | 0.461609461 | -55.01243717 | 2.48E-121 | 2.77E-120 | 266.9067045 |
| CTRL | -1.436315219 | 0.670834662 | -54.99374716 | 2.64E-121 | 2.94E-120 | 266.8437801 |
| KCNJ15 | -2.29446314 | 0.68449152 | -54.32132639 | 2.56E-120 | 2.81E-119 | 264.5672991 |
| MYL2 | -1.622007225 | 0.39022033 | -54.24244833 | 3.34E-120 | 3.66E-119 | 264.2986365 |
| FAM157A | -1.677723525 | 0.552546369 | -54.23611262 | 3.42E-120 | 3.72E-119 | 264.2770419 |
| SERF1B | -1.422570285 | 0.59102383 | -54.19990337 | 3.86E-120 | 4.19E-119 | 264.1535839 |
| RNU6-942P | 1.866790156 | 1.773525493 | 53.97102004 | 8.42E-120 | 9.10E-119 | 263.3715115 |
| AC012615.2 | -1.626695909 | 0.373693568 | -53.90782592 | 1.05E-119 | 1.12E-118 | 263.155071 |
| MIR5587 | 2.111435247 | 1.962002069 | 53.88964589 | 1.11E-119 | 1.19E-118 | 263.0927631 |
| AC233968.1 | -1.562983766 | 0.522161922 | -53.68986191 | 2.20E-119 | 2.34E-118 | 262.4068329 |
| SNORA28 | 1.900413808 | 1.799429429 | 53.52859771 | 3.83E-119 | 4.05E-118 | 261.8515236 |
| LAT | -1.823034036 | 0.928480252 | -53.39568843 | 6.05E-119 | 6.37E-118 | 261.3927529 |
| SCARNA2 | -1.684756627 | 0.387023835 | -53.20382654 | 1.17E-118 | 1.23E-117 | 260.728729 |
| LINC02289 | -1.504454738 | 0.40474733 | -52.64022498 | 8.30E-118 | 8.61E-117 | 258.7659801 |
| NOTCH2NLA | -1.571866488 | 0.789786339 | -52.54741434 | 1.15E-117 | 1.19E-116 | 258.4410122 |
| RF02119 | 2.258988963 | 2.075678655 | 52.46443549 | 1.53E-117 | 1.58E-116 | 258.1500464 |
| RPSAP58 | -1.881228379 | 0.797596936 | -52.42739011 | 1.75E-117 | 1.79E-116 | 258.0200173 |
| AP000763.2 | 2.05679524 | 1.976246088 | 52.27471726 | 2.98E-117 | 3.04E-116 | 257.4832925 |
| AC023509.1 | -1.456982897 | 0.509022533 | -52.2287847 | 3.50E-117 | 3.56E-116 | 257.321549 |
| AL449212.1 | 1.79826628 | 1.809297349 | 52.06388974 | 6.26E-117 | 6.30E-116 | 256.7398812 |
| PGA4 | -1.684742635 | 0.39000881 | -51.90194806 | 1.11E-116 | 1.11E-115 | 256.1670736 |
| ARPC4-TTLL3 | -1.567020958 | 1.009314659 | -51.90123472 | 1.11E-116 | 1.11E-115 | 256.164547 |
| AC025423.2 | -1.651817235 | 0.379461219 | -51.82579472 | 1.45E-116 | 1.44E-115 | 255.8971741 |
| SRXN1 | -1.75143649 | 0.779410989 | -51.81790999 | 1.49E-116 | 1.47E-115 | 255.8692098 |
| AC011462.2 | -1.656646641 | 0.755643309 | -51.76475648 | 1.80E-116 | 1.77E-115 | 255.6805968 |
| RNF216-IT1 | 1.646837367 | 1.647434067 | 51.73126167 | 2.02E-116 | 1.99E-115 | 255.5616557 |
| AC139495.2 | -1.83021426 | 0.424827748 | -51.56869201 | 3.60E-116 | 3.52E-115 | 254.9834172 |
| C10orf105 | -1.542436552 | 0.542203458 | -51.32942359 | 8.44E-116 | 8.22E-115 | 254.1294971 |
| AC004057.1 | -2.3838278 | 0.878574604 | -51.09642597 | 1.94E-115 | 1.88E-114 | 253.2946459 |
| MIR5194 | 2.071956954 | 1.931587669 | 50.66040078 | 9.28E-115 | 8.97E-114 | 251.7234706 |
| GUSBP11 | -1.609002648 | 1.035371705 | -50.65623875 | 9.42E-115 | 9.07E-114 | 251.7084172 |
| RNU6-1262P | 1.763781333 | 1.694166655 | 50.55696967 | 1.35E-114 | 1.29E-113 | 251.3490588 |
| AL807752.6 | -1.695158248 | 0.392571851 | -50.51374992 | 1.58E-114 | 1.50E-113 | 251.1924118 |
| AC087276.1 | 1.642429711 | 1.626950065 | 50.47624128 | 1.80E-114 | 1.72E-113 | 251.056371 |
| PLA2G4B | -1.458549492 | 0.783765957 | -50.38328491 | 2.53E-114 | 2.39E-113 | 250.7188508 |
| ALG13-AS1 | 1.807042324 | 1.751742311 | 50.31702859 | 3.21E-114 | 3.03E-113 | 250.4779507 |
| FAM157B | -1.9024217 | 0.644095721 | -50.29494084 | 3.48E-114 | 3.27E-113 | 250.3975817 |
| AC138035.1 | -1.841592304 | 1.02580122 | -50.24442387 | 4.18E-114 | 3.91E-113 | 250.2136557 |
| TLR9 | -1.374709488 | 0.616418401 | -49.9089739 | 1.42E-113 | 1.32E-112 | 248.9882848 |
| SNORD63 | 1.908995214 | 1.806040615 | 49.80534124 | 2.07E-113 | 1.91E-112 | 248.6082968 |
| MB | -1.563145023 | 0.406074691 | -49.71993907 | 2.83E-113 | 2.61E-112 | 248.2946454 |
| AL121845.2 | -1.896482709 | 0.440755834 | -49.55940343 | 5.10E-113 | 4.68E-112 | 247.7038069 |
| AC240565.1 | -1.48608727 | 0.476313669 | -49.34023646 | 1.14E-112 | 1.04E-111 | 246.8945354 |
| RAB43 | -1.716448111 | 0.869921938 | -49.33578809 | 1.16E-112 | 1.06E-111 | 246.8780781 |
| RNU6-199P | 1.933161578 | 1.824658578 | 49.26133071 | 1.53E-112 | 1.39E-111 | 246.6024258 |
| DAPK2 | -1.82634594 | 0.779974649 | -49.21302023 | 1.83E-112 | 1.65E-111 | 246.4233831 |
| HSPE1P27 | 1.81753878 | 1.781440896 | 49.20046594 | 1.91E-112 | 1.72E-111 | 246.3768314 |
| SELENOM | -1.556969702 | 0.643109826 | -49.16708595 | 2.17E-112 | 1.94E-111 | 246.2530079 |
| AC007283.1 | -1.80281349 | 0.414128727 | -48.9691641 | 4.51E-112 | 4.00E-111 | 245.5173399 |
| RNU6-672P | 1.806158835 | 1.726814629 | 48.8101532 | 8.14E-112 | 7.19E-111 | 244.9244672 |
| MIR3176 | 1.935914163 | 1.826779193 | 48.72773404 | 1.11E-111 | 9.74E-111 | 244.6165207 |
| MT-TF | 2.386720894 | 2.174084378 | 48.65775639 | 1.44E-111 | 1.25E-110 | 244.3547124 |
| CHIT1 | -1.80719556 | 0.567373443 | -48.64024094 | 1.53E-111 | 1.33E-110 | 244.2891316 |
| TMEM191A | -1.392764752 | 0.694723413 | -48.60221122 | 1.77E-111 | 1.53E-110 | 244.1466729 |
| RNY4P25 | 1.83022793 | 1.745357656 | 48.44592434 | 3.17E-111 | 2.74E-110 | 243.5602299 |
| AC131392.2 | -1.487390511 | 0.498434999 | -48.3104565 | 5.27E-111 | 4.54E-110 | 243.050608 |
| MYB | 1.428880464 | 2.574543604 | 48.25248382 | 6.55E-111 | 5.62E-110 | 242.8321483 |
| SNORD11 | 1.914301378 | 1.810128527 | 48.20256136 | 7.90E-111 | 6.73E-110 | 242.6438463 |
| KLC3 | -1.539087253 | 0.510757131 | -48.19617147 | 8.09E-111 | 6.87E-110 | 242.6197324 |
| RNU1-3 | -1.929546921 | 0.467904932 | -48.14538636 | 9.80E-111 | 8.29E-110 | 242.4279855 |
| CA4 | -2.110704106 | 0.669327941 | -48.12165911 | 1.07E-110 | 9.03E-110 | 242.3383411 |
| AC090971.3 | 1.720592602 | 1.878450585 | 48.00769569 | 1.65E-110 | 1.38E-109 | 241.9072517 |
| FOXP1-AS1 | 1.586287894 | 1.55742426 | 47.92626822 | 2.24E-110 | 1.87E-109 | 241.5987059 |
| NRG1 | -1.588916488 | 0.447353878 | -47.78833742 | 3.77E-110 | 3.13E-109 | 241.0750456 |
| RNU7-40P | 1.818409378 | 1.736252547 | 47.78776967 | 3.78E-110 | 3.13E-109 | 241.0728875 |
| SNORA1B | 2.12397355 | 2.040729498 | 47.69106245 | 5.46E-110 | 4.50E-109 | 240.7049695 |
| AC079329.1 | -1.675037285 | 0.384792353 | -47.59407155 | 7.89E-110 | 6.48E-109 | 240.3353397 |
| RNU6-879P | 1.907000408 | 1.804503799 | 47.4955201 | 1.15E-109 | 9.40E-109 | 239.9591122 |
| AP003108.2 | -1.429988202 | 1.031227035 | -47.22020485 | 3.28E-109 | 2.67E-108 | 238.9045831 |
| AL049830.4 | 1.693535071 | 1.717743921 | 47.18947737 | 3.69E-109 | 2.99E-108 | 238.7865684 |
| ABCC13 | -1.487890554 | 0.44080264 | -46.93473194 | 9.81E-109 | 7.93E-108 | 237.805678 |
| AP006222.1 | -1.461507452 | 0.560096192 | -46.84869072 | 1.37E-108 | 1.10E-107 | 237.4733698 |
| PRSS23 | -1.458906087 | 0.522902346 | -46.84514467 | 1.39E-108 | 1.11E-107 | 237.4596633 |
| TUBA8 | -1.462210583 | 0.674539475 | -46.71554906 | 2.28E-108 | 1.83E-107 | 236.9581412 |
| SNORD62A | 1.867488944 | 1.774063846 | 46.55226354 | 4.29E-108 | 3.42E-107 | 236.3245821 |
| RNU6-151P | 1.941051916 | 1.83073736 | 46.44173983 | 6.59E-108 | 5.22E-107 | 235.8946875 |
| RTEL1 | -1.189901465 | 0.842817667 | -46.22584726 | 1.53E-107 | 1.20E-106 | 235.0524787 |
| RNU4ATAC11P | 1.885020287 | 1.787570135 | 46.15504287 | 2.01E-107 | 1.58E-106 | 234.7755525 |
| PLGLB1 | -1.363233047 | 0.62994142 | -45.85459475 | 6.51E-107 | 5.10E-106 | 233.5965068 |
| RNU7-181P | 2.035184534 | 1.903257896 | 45.71640259 | 1.12E-106 | 8.74E-106 | 233.0520425 |
| ITIH4 | -1.425498612 | 0.896665496 | -45.71594792 | 1.12E-106 | 8.74E-106 | 233.0502489 |
| AC103810.3 | 2.10903153 | 1.960150225 | 45.68456121 | 1.27E-106 | 9.86E-106 | 232.9263967 |
| RNY4P6 | 1.877048334 | 1.781428477 | 45.59862142 | 1.78E-106 | 1.38E-105 | 232.586916 |
| AC025262.3 | -1.729239392 | 0.397236715 | -45.58893465 | 1.85E-106 | 1.43E-105 | 232.5486179 |
| AL513314.2 | -1.416708771 | 0.773719088 | -45.52507831 | 2.38E-106 | 1.83E-105 | 232.2959833 |
| RGPD2 | -1.55498846 | 0.522668986 | -45.50316411 | 2.59E-106 | 1.99E-105 | 232.2092165 |
| SNX15 | -1.353119692 | 0.891674295 | -45.39508703 | 3.98E-106 | 3.03E-105 | 231.7807905 |
| RNU4-89P | 1.733093146 | 1.704577057 | 45.38231255 | 4.18E-106 | 3.17E-105 | 231.7300956 |
| RNU6-780P | 1.873873475 | 1.77898254 | 45.24338276 | 7.25E-106 | 5.48E-105 | 231.1779961 |
| SEPT14P19 | -1.549242955 | 0.377525595 | -45.22229068 | 7.88E-106 | 5.94E-105 | 231.0940548 |
| NPIPB12 | -1.568677132 | 1.084482979 | -45.22033393 | 7.94E-106 | 5.97E-105 | 231.0862658 |
| SNORD12C | 1.875599389 | 1.780312199 | 45.20272888 | 8.52E-106 | 6.38E-105 | 231.0161747 |
| AC005944.1 | -1.574278183 | 0.361658886 | -45.16657378 | 9.83E-106 | 7.32E-105 | 230.8721594 |
| TMSB4XP6 | -2.275584531 | 0.522673099 | -45.09712833 | 1.30E-105 | 9.62E-105 | 230.5952724 |
| RNU7-3P | 1.724635507 | 1.664008391 | 45.05018342 | 1.56E-105 | 1.16E-104 | 230.407898 |
| STIMATE | -1.344053677 | 0.974169484 | -44.94705083 | 2.35E-105 | 1.74E-104 | 229.9956916 |
| AC060780.3 | 2.023005404 | 1.893874995 | 44.86512683 | 3.26E-105 | 2.39E-104 | 229.6676966 |
| MIR573 | 1.84880241 | 1.759667587 | 44.8288178 | 3.77E-105 | 2.76E-104 | 229.5221701 |
| MIR3609 | 2.225410485 | 2.049809522 | 44.72940118 | 5.62E-105 | 4.09E-104 | 229.1232111 |
| RMRP | -1.555602121 | 0.514776866 | -44.69149056 | 6.54E-105 | 4.75E-104 | 228.9708834 |
| KIR2DL1 | -1.686418416 | 0.546548731 | -44.68273311 | 6.77E-105 | 4.91E-104 | 228.9356802 |
| KLRK1 | -1.876158712 | 0.778787726 | -44.59327019 | 9.69E-105 | 6.98E-104 | 228.5757317 |
| AC092368.3 | -1.463508323 | 0.740631246 | -44.40764505 | 2.04E-104 | 1.47E-103 | 227.826983 |
| CCNJL | -1.999080588 | 0.669741469 | -44.33395037 | 2.75E-104 | 1.96E-103 | 227.5290111 |
| MIR616 | 1.959025927 | 1.844584684 | 44.23253441 | 4.14E-104 | 2.95E-103 | 227.118288 |
| MIR421 | 2.066275198 | 1.927210398 | 44.21780851 | 4.40E-104 | 3.12E-103 | 227.0585856 |
| MTHFS | -1.475346031 | 1.236734518 | -44.130887 | 6.25E-104 | 4.42E-103 | 226.7058526 |
| ANKRD10-IT1 | 1.006889924 | 2.384721217 | 44.04825441 | 8.73E-104 | 6.16E-103 | 226.3699965 |
| MIR4755 | 1.671214433 | 1.622852359 | 43.92732165 | 1.43E-103 | 1.00E-102 | 225.8775411 |
| MT-TC | 2.172846354 | 2.009313687 | 43.84091578 | 2.03E-103 | 1.42E-102 | 225.5250052 |
| SNORA75 | 2.015542016 | 1.959393292 | 43.83546672 | 2.07E-103 | 1.45E-102 | 225.502754 |
| MIR1285-1 | 1.778102438 | 1.705199751 | 43.70185049 | 3.57E-103 | 2.49E-102 | 224.9564261 |
| DNLZ | -1.435019167 | 0.751632774 | -43.68531683 | 3.82E-103 | 2.65E-102 | 224.8887288 |
| SNORD59A | 1.887866753 | 1.789763076 | 43.68104112 | 3.89E-103 | 2.69E-102 | 224.8712185 |
| OSGIN1 | -1.357374895 | 0.631925725 | -43.61083801 | 5.18E-103 | 3.56E-102 | 224.5835153 |
| TOGARAM2 | -1.497598349 | 0.638823847 | -43.5457926 | 6.76E-103 | 4.63E-102 | 224.3166123 |
| AL139220.2 | -1.599045307 | 0.601359777 | -43.44601635 | 1.02E-102 | 6.94E-102 | 223.9065656 |
| SNORD101 | 1.939866617 | 1.829824195 | 43.40706962 | 1.19E-102 | 8.12E-102 | 223.7462999 |
| TMEM40 | -1.636446402 | 0.517520296 | -43.37743715 | 1.35E-102 | 9.12E-102 | 223.6242839 |
| LIMS2 | -1.424889957 | 0.566072052 | -43.15221917 | 3.40E-102 | 2.28E-101 | 222.6946974 |
| ST6GALNAC3 | -1.581008402 | 0.528144077 | -43.11840495 | 3.91E-102 | 2.61E-101 | 222.5547896 |
| GET4 | -1.389988088 | 1.074161299 | -43.03143371 | 5.60E-102 | 3.71E-101 | 222.1945334 |
| AC091390.3 | 1.690373694 | 1.753362843 | 42.97052468 | 7.20E-102 | 4.75E-101 | 221.9418819 |
| TMEM45B | -1.292533955 | 0.70831135 | -42.87361724 | 1.08E-101 | 7.08E-101 | 221.5393104 |
| RNU6-856P | 1.557477317 | 1.535228357 | 42.80256065 | 1.44E-101 | 9.48E-101 | 221.2436603 |
| DNAH17 | -1.398614824 | 1.006355485 | -42.71317905 | 2.09E-101 | 1.37E-100 | 220.8712004 |
| PLIN5 | -1.86308674 | 0.753524021 | -42.68101995 | 2.39E-101 | 1.56E-100 | 220.737037 |
| RNU6-510P | 1.829076017 | 1.744470212 | 42.60609782 | 3.27E-101 | 2.13E-100 | 220.4241554 |
| AC240565.2 | -1.835696498 | 0.939525225 | -42.57135888 | 3.78E-101 | 2.45E-100 | 220.2789318 |
| KIR3DL1 | -1.653556686 | 0.563848375 | -42.55141834 | 4.10E-101 | 2.66E-100 | 220.1955287 |
| AC068580.4 | -1.489136068 | 0.489153442 | -42.43010521 | 6.81E-101 | 4.37E-100 | 219.6874459 |
| RNU6-91P | 1.614148412 | 1.578888231 | 42.42427517 | 6.98E-101 | 4.47E-100 | 219.6629991 |
| AL133453.1 | -1.63078579 | 0.374632571 | -42.42206983 | 7.04E-101 | 4.50E-100 | 219.6537509 |
| RGS9 | -1.4403795 | 0.544243675 | -42.31870854 | 1.08E-100 | 6.89E-100 | 219.2198642 |
| AP003419.1 | -1.857364555 | 0.429710282 | -42.29067228 | 1.22E-100 | 7.73E-100 | 219.1020276 |
| PLSCR3 | -1.611592243 | 0.989758431 | -42.24234727 | 1.49E-100 | 9.42E-100 | 218.8987697 |
| AL831737.1 | 1.683984662 | 1.657676863 | 42.14112474 | 2.28E-100 | 1.43E-99 | 218.4724156 |
| TNFRSF13B | -1.383371973 | 0.595394388 | -42.09907103 | 2.73E-100 | 1.70E-99 | 218.2950417 |
| SNORA2B | 1.720040086 | 1.660468041 | 42.03086417 | 3.63E-100 | 2.27E-99 | 218.0070567 |
| RNU6-531P | 2.142952807 | 1.986283454 | 41.98283938 | 4.45E-100 | 2.77E-99 | 217.8040602 |
| RNU4ATAC18P | 1.724467569 | 1.699880895 | 41.86881648 | 7.19E-100 | 4.46E-99 | 217.3213496 |
| SNORD83A | 1.849709296 | 1.760366259 | 41.71219458 | 1.40E-99 | 8.59E-99 | 216.6565808 |
| RNU6-1095P | 1.640657981 | 1.59931142 | 41.69670689 | 1.49E-99 | 9.15E-99 | 216.5907363 |
| AL356320.1 | 1.71337376 | 1.683004188 | 41.56151708 | 2.64E-99 | 1.61E-98 | 216.0151581 |
| AC011498.4 | -1.493121912 | 0.564110978 | -41.49071376 | 3.57E-99 | 2.17E-98 | 215.713113 |
| RNU5B-2P | 1.681617864 | 1.630867248 | 41.47772042 | 3.78E-99 | 2.28E-98 | 215.6576391 |
| CASTOR1 | -1.299626209 | 0.801480372 | -41.38240698 | 5.67E-99 | 3.42E-98 | 215.2502836 |
| NDUFA3P2 | 1.549466855 | 1.55152587 | 41.31722425 | 7.48E-99 | 4.50E-98 | 214.971272 |
| KIR3DL2 | -1.568127663 | 0.525882359 | -41.2913105 | 8.36E-99 | 5.01E-98 | 214.8602522 |
| SNRPGP18 | 1.84518434 | 1.833005816 | 41.14107681 | 1.59E-98 | 9.49E-98 | 214.2155271 |
| MIA2 | -1.356705072 | 1.015472592 | -41.12059728 | 1.73E-98 | 1.03E-97 | 214.1274949 |
| RNU1-4 | -1.887617271 | 0.500207876 | -41.08581269 | 2.01E-98 | 1.20E-97 | 213.977892 |
| ZDHHC19 | -2.024984892 | 0.617304149 | -41.05187754 | 2.33E-98 | 1.38E-97 | 213.8318454 |
| AC136475.5 | -1.630758721 | 0.689951727 | -41.04665987 | 2.38E-98 | 1.41E-97 | 213.8093817 |
| AL451062.2 | 1.708044959 | 1.721878426 | 41.00810315 | 2.81E-98 | 1.65E-97 | 213.6433124 |
| AC023389.2 | 1.692587702 | 1.768006196 | 40.91482333 | 4.20E-98 | 2.46E-97 | 213.2410308 |
| FSD1 | -1.479016544 | 0.538176409 | -40.85560135 | 5.41E-98 | 3.16E-97 | 212.9852511 |
| MIR3174 | 1.981911968 | 1.862216277 | 40.84039281 | 5.78E-98 | 3.37E-97 | 212.9195181 |
| AC116348.4 | -1.583317674 | 0.496224339 | -40.82256785 | 6.24E-98 | 3.63E-97 | 212.842452 |
| ARL6IP4 | -1.791422621 | 1.189656753 | -40.71023911 | 1.01E-97 | 5.86E-97 | 212.3561865 |
| SNORA33 | 1.929063057 | 1.910527827 | 40.67153803 | 1.20E-97 | 6.90E-97 | 212.1884061 |
| MIR374B | 1.991929594 | 1.869933938 | 40.65950617 | 1.26E-97 | 7.24E-97 | 212.1362188 |
| EPHB1 | -1.513967518 | 0.559911495 | -40.5760377 | 1.81E-97 | 1.04E-96 | 211.7738446 |
| MIR378J | 1.767418218 | 1.740721162 | 40.54911133 | 2.03E-97 | 1.16E-96 | 211.6568197 |
| AL513008.1 | 2.04163282 | 1.999969647 | 40.54133347 | 2.10E-97 | 1.20E-96 | 211.6230049 |
| AL031600.3 | -1.624713081 | 0.373238327 | -40.44426926 | 3.20E-97 | 1.82E-96 | 211.2005808 |
| AL132989.1 | 1.589374844 | 1.900495531 | 40.43918221 | 3.28E-97 | 1.86E-96 | 211.1784199 |
| CDK6 | 1.375088369 | 2.557013318 | 40.37994431 | 4.24E-97 | 2.40E-96 | 210.9201982 |
| MIR3682 | 1.873627048 | 1.778792691 | 40.29505027 | 6.13E-97 | 3.45E-96 | 210.54962 |
| C17orf49 | -1.769715583 | 1.07395433 | -40.20797949 | 8.96E-97 | 5.02E-96 | 210.1689022 |
| AL121845.3 | -1.301779481 | 0.758814323 | -40.14093185 | 1.20E-96 | 6.68E-96 | 209.8752945 |
| CBWD6 | -1.281797705 | 0.758311452 | -40.1020016 | 1.42E-96 | 7.90E-96 | 209.7046388 |
| SNORD69 | 1.833683151 | 1.748019586 | 40.09235681 | 1.48E-96 | 8.22E-96 | 209.6623396 |
| CBWD5 | -1.338914012 | 0.926770518 | -39.72404327 | 7.47E-96 | 4.09E-95 | 208.0410337 |
| REM2 | -1.497280109 | 0.836502139 | -39.55276229 | 1.59E-95 | 8.66E-95 | 207.283059 |
| PSMC1P5 | -1.82018147 | 0.515116662 | -39.43175356 | 2.71E-95 | 1.47E-94 | 206.7460137 |
| RN7SL558P | 1.569542155 | 1.748430755 | 39.35232739 | 3.86E-95 | 2.08E-94 | 206.3928179 |
| DARS-AS1 | -1.310026468 | 0.781985085 | -39.208826 | 7.30E-95 | 3.92E-94 | 205.7532845 |
| SNORD19 | 1.825976589 | 1.742082388 | 39.18109459 | 8.25E-95 | 4.43E-94 | 205.6294866 |
| MTMR3 | -1.230802177 | 1.53687959 | -39.16311357 | 8.94E-95 | 4.79E-94 | 205.5491798 |
| AL138721.1 | 1.562993596 | 1.567174816 | 39.07309884 | 1.34E-94 | 7.11E-94 | 205.1467269 |
| RNU4ATAC12P | 1.786043641 | 1.711317719 | 39.02198465 | 1.68E-94 | 8.92E-94 | 204.9178776 |
| AL139120.1 | 1.664093623 | 1.61736643 | 38.93849544 | 2.44E-94 | 1.29E-93 | 204.5435797 |
| AL356320.2 | 1.72364233 | 1.733329822 | 38.93431965 | 2.48E-94 | 1.31E-93 | 204.5248426 |
| EIF3C | -1.563199709 | 1.28213715 | -38.89197157 | 3.00E-94 | 1.58E-93 | 204.3347354 |
| MIR199A1 | 1.722494463 | 1.662358913 | 38.88640263 | 3.07E-94 | 1.62E-93 | 204.3097237 |
| SNORA16B | 1.72664993 | 1.769678537 | 38.70285508 | 7.00E-94 | 3.66E-93 | 203.4838091 |
| KRT13 | -1.633517356 | 0.491656156 | -38.67894418 | 7.80E-94 | 4.07E-93 | 203.3759945 |
| AC007533.1 | 1.445679263 | 1.546008306 | 38.49306134 | 1.80E-93 | 9.36E-93 | 202.5360943 |
| RNA5SP78 | 1.69191611 | 1.638801101 | 38.47326698 | 1.97E-93 | 1.02E-92 | 202.4464714 |
| CXCR1 | -2.447030299 | 1.011189553 | -38.45092328 | 2.18E-93 | 1.13E-92 | 202.3452632 |
| CHI3L2 | -1.333207664 | 0.795861636 | -38.43841718 | 2.30E-93 | 1.19E-92 | 202.2885959 |
| RPS11P5 | 1.00703172 | 2.198573059 | 38.43646437 | 2.32E-93 | 1.20E-92 | 202.2797461 |
| DIP2A-IT1 | 1.527851592 | 1.664635991 | 38.4334791 | 2.36E-93 | 1.21E-92 | 202.2662167 |
| RNA5SP307 | 1.950547194 | 1.838052599 | 38.42843541 | 2.41E-93 | 1.24E-92 | 202.2433566 |
| RNU1-27P | -1.836248099 | 0.539783106 | -38.38896722 | 2.88E-93 | 1.48E-92 | 202.0643908 |
| AK1 | -1.70282979 | 0.946452051 | -38.38117875 | 2.98E-93 | 1.52E-92 | 202.0290579 |
| RPS12P27 | 1.602139223 | 1.86488032 | 38.31369974 | 4.05E-93 | 2.06E-92 | 201.7227046 |
| SNORA71C | 1.94122528 | 1.974246925 | 38.30629131 | 4.19E-93 | 2.13E-92 | 201.6890453 |
| EEF1A1P3 | 1.500885531 | 1.870981336 | 38.23562857 | 5.77E-93 | 2.92E-92 | 201.3677475 |
| RNA5SP317 | 1.711292036 | 1.653728472 | 38.21501404 | 6.33E-93 | 3.20E-92 | 201.2739293 |
| AC018607.1 | 1.762631605 | 2.160435415 | 38.20012913 | 6.77E-93 | 3.42E-92 | 201.206163 |
| RNU6-1165P | 1.641294938 | 1.599802136 | 38.17553523 | 7.57E-93 | 3.80E-92 | 201.0941505 |
| U2AF1L5 | -1.894520926 | 0.85254212 | -38.15078289 | 8.48E-93 | 4.25E-92 | 200.9813607 |
| CYP1B1-AS1 | -1.469622171 | 0.477241306 | -38.07175309 | 1.21E-92 | 6.06E-92 | 200.6208687 |
| CD177 | -2.484998997 | 0.803211868 | -37.91594349 | 2.47E-92 | 1.23E-91 | 199.9084738 |
| CD177P1 | -1.601874139 | 0.397665651 | -37.89029131 | 2.78E-92 | 1.38E-91 | 199.7909729 |
| MIR25 | 1.811728026 | 1.731105179 | 37.8161212 | 3.89E-92 | 1.93E-91 | 199.450893 |
| AC027644.1 | 1.683338364 | 1.653053241 | 37.75541963 | 5.14E-92 | 2.54E-91 | 199.1721907 |
| SLC51A | -1.282172842 | 1.038426948 | -37.73003503 | 5.78E-92 | 2.85E-91 | 199.0555403 |
| MIR5690 | 2.025431784 | 1.895744299 | 37.66819066 | 7.67E-92 | 3.77E-91 | 198.7710958 |
| TRAPPC5 | -1.559623864 | 1.176686253 | -37.65905755 | 8.00E-92 | 3.93E-91 | 198.7290594 |
| LSMEM1 | -1.35196886 | 0.932208798 | -37.63437627 | 8.95E-92 | 4.39E-91 | 198.6154216 |
| ORM1 | -2.117536897 | 0.75935894 | -37.59698569 | 1.06E-91 | 5.20E-91 | 198.4431598 |
| PI3 | -2.322489827 | 0.710488653 | -37.53954145 | 1.38E-91 | 6.75E-91 | 198.1782563 |
| TRIM34 | -1.198373888 | 0.880214683 | -37.51983963 | 1.52E-91 | 7.38E-91 | 198.0873308 |
| BOLA2-SMG1P6 | -1.108197703 | 0.988060714 | -37.34175941 | 3.44E-91 | 1.67E-90 | 197.2638353 |
| AP000936.3 | 1.759982591 | 1.952110042 | 37.26215418 | 4.97E-91 | 2.40E-90 | 196.8947584 |
| RAB43P1 | -1.493825258 | 0.942322394 | -37.19829367 | 6.68E-91 | 3.21E-90 | 196.5982497 |
| SNORA77 | 1.7846589 | 1.751069514 | 37.18423297 | 7.13E-91 | 3.42E-90 | 196.5329134 |
| KCNMA1 | -1.530064028 | 0.416649185 | -37.16336457 | 7.86E-91 | 3.74E-90 | 196.4359093 |
| PCSK1N | -1.571942858 | 0.506405985 | -37.14673612 | 8.49E-91 | 4.03E-90 | 196.3585848 |
| AC093162.2 | 1.276798824 | 2.279684845 | 36.96186578 | 2.00E-90 | 9.49E-90 | 195.4971556 |
| GAPDHP61 | 1.501919092 | 1.794708606 | 36.93979365 | 2.22E-90 | 1.05E-89 | 195.3940916 |
| AL031846.1 | -1.420778591 | 0.600770575 | -36.92873452 | 2.34E-90 | 1.10E-89 | 195.3424345 |
| RNU6-387P | 1.678763287 | 1.628668058 | 36.92603173 | 2.37E-90 | 1.11E-89 | 195.329808 |
| RPH3A | -1.668764552 | 0.50403475 | -36.91203705 | 2.52E-90 | 1.18E-89 | 195.2644187 |
| ISCA1P1 | -1.499708262 | 0.51128436 | -36.83254528 | 3.66E-90 | 1.70E-89 | 194.8926458 |
| AC005086.1 | 1.517749925 | 1.729869519 | 36.72900258 | 5.93E-90 | 2.75E-89 | 194.4074885 |
| RNU6-375P | 1.458048427 | 1.458627528 | 36.60416962 | 1.06E-89 | 4.93E-89 | 193.8212138 |
| RNU1-28P | -1.820947164 | 0.551571071 | -36.5977976 | 1.10E-89 | 5.06E-89 | 193.7912478 |
| RNU6-638P | 1.796263338 | 1.719191057 | 36.5927261 | 1.12E-89 | 5.18E-89 | 193.767395 |
| MIR548AA1 | 1.850180934 | 1.760729613 | 36.51802077 | 1.59E-89 | 7.33E-89 | 193.415748 |
| HAUS7 | -1.256361123 | 1.046382185 | -36.48164252 | 1.89E-89 | 8.68E-89 | 193.2443172 |
| AL008729.2 | -1.535285346 | 0.757770333 | -36.47496689 | 1.95E-89 | 8.94E-89 | 193.2128448 |
| RPL26P27 | 1.269885335 | 1.439190411 | 36.42094968 | 2.51E-89 | 1.15E-88 | 192.9580218 |
| FTH1P10 | -1.569277981 | 0.773320023 | -36.26833176 | 5.16E-89 | 2.35E-88 | 192.2365353 |
| ALB | -1.893574104 | 0.496277501 | -36.2094091 | 6.81E-89 | 3.09E-88 | 191.9573816 |
| LINC00570 | -1.705471346 | 0.559894562 | -36.08340154 | 1.24E-88 | 5.58E-88 | 191.3592739 |
| RPL36AP15 | 1.580599918 | 1.645726458 | 36.05572301 | 1.41E-88 | 6.35E-88 | 191.2276881 |
| PSMC1P1 | -1.319872793 | 0.838445581 | -36.02857817 | 1.60E-88 | 7.21E-88 | 191.0985671 |
| AC018755.2 | -1.812288191 | 0.471871401 | -36.02830255 | 1.61E-88 | 7.21E-88 | 191.0972557 |
| PF4V1 | -1.486148939 | 0.467016801 | -35.99501222 | 1.88E-88 | 8.43E-88 | 190.9388031 |
| SNORD99 | 1.824892913 | 1.741247515 | 35.98181128 | 2.00E-88 | 8.94E-88 | 190.8759404 |
| AC024075.1 | 1.02571864 | 1.973444637 | 35.95080321 | 2.32E-88 | 1.03E-87 | 190.7282135 |
| SNORA70B | 1.439486392 | 1.444327185 | 35.94422281 | 2.39E-88 | 1.06E-87 | 190.6968514 |
| SAA1 | -1.650353174 | 0.480895936 | -35.92667731 | 2.60E-88 | 1.15E-87 | 190.6132092 |
| TMEM110-MUSTN1 | -1.183228052 | 0.929022917 | -35.81620199 | 4.40E-88 | 1.93E-87 | 190.0858632 |
| SNORA71A | 1.830886364 | 1.85022585 | 35.79706459 | 4.82E-88 | 2.11E-87 | 189.9943906 |
| NBPF14 | -1.262242752 | 1.073855163 | -35.75893783 | 5.78E-88 | 2.52E-87 | 189.8120458 |
| RUNDC3A | -1.980635917 | 0.782600702 | -35.74706808 | 6.11E-88 | 2.66E-87 | 189.7552485 |
| CIDEB | -1.677568722 | 0.979307205 | -35.71502249 | 7.12E-88 | 3.09E-87 | 189.6018394 |
| AC005062.1 | -1.410574572 | 0.547557845 | -35.69048121 | 8.01E-88 | 3.47E-87 | 189.4842868 |
| RNU6-10P | 1.976082408 | 1.857725136 | 35.67850116 | 8.48E-88 | 3.66E-87 | 189.4268808 |
| TBC1D3L | -1.741306389 | 0.641217956 | -35.62360009 | 1.10E-87 | 4.75E-87 | 189.163625 |
| LGALS9B | -1.428988241 | 0.487928162 | -35.5377546 | 1.66E-87 | 7.14E-87 | 188.7513913 |
| AC253536.3 | 1.651835792 | 1.804304767 | 35.48430759 | 2.15E-87 | 9.21E-87 | 188.494368 |
| TMCC3 | -1.604279233 | 0.795065083 | -35.47418441 | 2.25E-87 | 9.65E-87 | 188.4456544 |
| ANKRD37 | -1.319336495 | 0.863361882 | -35.38238693 | 3.50E-87 | 1.49E-86 | 188.0034527 |
| CHMP1B-AS1 | -1.586096365 | 0.364372244 | -35.34787316 | 4.13E-87 | 1.76E-86 | 187.8369782 |
| PABPC1P1 | 1.4633759 | 1.698260377 | 35.32364953 | 4.64E-87 | 1.97E-86 | 187.7200665 |
| MIR320E | 1.742697809 | 1.719898953 | 35.30932808 | 4.98E-87 | 2.11E-86 | 187.6509187 |
| MIR3679 | 1.628923031 | 1.590270719 | 35.30804817 | 5.01E-87 | 2.12E-86 | 187.644738 |
| GIMAP5 | -1.636404948 | 0.934207994 | -35.30431341 | 5.10E-87 | 2.15E-86 | 187.6267017 |
| SNORD124 | 1.48889892 | 1.482395 | 35.23171437 | 7.23E-87 | 3.04E-86 | 187.2758231 |
| MIR221 | 2.014450629 | 1.929497743 | 35.21985618 | 7.66E-87 | 3.21E-86 | 187.2184613 |
| FLT3LG | -1.471405526 | 1.055788377 | -35.08384635 | 1.48E-86 | 6.19E-86 | 186.5595318 |
| AC104692.1 | 1.636260692 | 1.595923712 | 35.05555161 | 1.69E-86 | 7.08E-86 | 186.4222187 |
| RNY1P16 | 1.798051744 | 1.803125291 | 35.03750304 | 1.85E-86 | 7.71E-86 | 186.3345878 |
| RNY1P13 | 1.757505023 | 1.689331335 | 34.97123887 | 2.55E-86 | 1.06E-85 | 186.0125755 |
| CCR3 | -1.425119421 | 0.571089971 | -34.96804345 | 2.59E-86 | 1.08E-85 | 185.9970361 |
| NOMO3 | -1.303307136 | 0.937857125 | -34.95041886 | 2.82E-86 | 1.17E-85 | 185.9113089 |
| LINC01001 | -1.852950124 | 1.276263183 | -34.94778468 | 2.85E-86 | 1.18E-85 | 185.8984934 |
| PTENP1 | -1.238392846 | 0.82139277 | -34.93056404 | 3.10E-86 | 1.28E-85 | 185.8146962 |
| PHBP19 | 1.35796709 | 1.7538684 | 34.9289124 | 3.13E-86 | 1.29E-85 | 185.8066576 |
| AL645939.1 | -1.338475167 | 0.669766379 | -34.89693766 | 3.65E-86 | 1.51E-85 | 185.650981 |
| NPIPA1 | -1.411851134 | 1.034822396 | -34.82323451 | 5.22E-86 | 2.15E-85 | 185.2917465 |
| RABGEF1 | -1.274219391 | 1.182495307 | -34.77248005 | 6.68E-86 | 2.74E-85 | 185.0440461 |
| AC068533.3 | 1.578432401 | 1.551372325 | 34.76051237 | 7.08E-86 | 2.90E-85 | 184.9856013 |
| MIRLET7D | 1.739589827 | 1.675529322 | 34.69579637 | 9.71E-86 | 3.97E-85 | 184.6693063 |
| MT-TT | 2.230784044 | 2.053949356 | 34.6389504 | 1.28E-85 | 5.22E-85 | 184.3911245 |
| RNU6-1280P | 1.790368164 | 1.714649367 | 34.54346456 | 2.04E-85 | 8.31E-85 | 183.9231143 |
| RNU6-90P | 1.501721137 | 1.492273341 | 34.51600686 | 2.34E-85 | 9.47E-85 | 183.7883622 |
| ST6GALNAC2 | -1.540180936 | 0.848880383 | -34.31643472 | 6.21E-85 | 2.51E-84 | 182.8066197 |
| RNU1-87P | 1.464845856 | 1.463864323 | 34.26205852 | 8.12E-85 | 3.27E-84 | 182.538422 |
| GTF2H2C | -1.146970371 | 0.930279874 | -34.25828621 | 8.27E-85 | 3.32E-84 | 182.5198047 |
| IGKV1-33 | -1.925992069 | 0.700144603 | -34.19883248 | 1.11E-84 | 4.43E-84 | 182.2261921 |
| AC011939.2 | 1.722375998 | 1.730831486 | 34.18694457 | 1.17E-84 | 4.69E-84 | 182.1674398 |
| SNORD104 | 1.987348582 | 1.866404688 | 34.18507642 | 1.19E-84 | 4.73E-84 | 182.1582058 |
| MIR6739 | 1.425038764 | 1.433196615 | 34.15412048 | 1.38E-84 | 5.50E-84 | 182.0051415 |
| KIR2DL4 | -1.554074322 | 0.576962272 | -34.13719741 | 1.50E-84 | 5.96E-84 | 181.9214222 |
| GCHFR | -1.326605076 | 0.959154669 | -34.11586155 | 1.67E-84 | 6.60E-84 | 181.8158304 |
| MIR553 | 1.674947361 | 1.625728238 | 34.08027966 | 1.99E-84 | 7.86E-84 | 181.6396298 |
| RPL11P3 | 1.063184393 | 2.284255341 | 34.01078582 | 2.80E-84 | 1.10E-83 | 181.2951204 |
| RNU6-476P | 1.684678066 | 1.633224853 | 33.89732769 | 4.91E-84 | 1.93E-83 | 180.7315856 |
| HIST1H4J | -1.469317475 | 0.673147243 | -33.89553049 | 4.96E-84 | 1.94E-83 | 180.7226483 |
| CKLF-CMTM1 | -1.290582704 | 0.767537172 | -33.83608956 | 6.65E-84 | 2.60E-83 | 180.426866 |
| AC023389.1 | 1.521432844 | 1.687810813 | 33.82223825 | 7.13E-84 | 2.78E-83 | 180.357888 |
| HCAR3 | -1.994465616 | 0.953806644 | -33.80317227 | 7.84E-84 | 3.05E-83 | 180.2629089 |
| SNORD9 | 1.38095405 | 1.399233391 | 33.77322128 | 9.09E-84 | 3.53E-83 | 180.1136283 |
| IKBKG | -1.288272807 | 1.305508676 | -33.75748206 | 9.83E-84 | 3.81E-83 | 180.035144 |
| AL022238.1 | 1.465485357 | 1.622916945 | 33.74998368 | 1.02E-83 | 3.95E-83 | 179.9977439 |
| RPS29P14 | 1.548052331 | 1.616914519 | 33.72319976 | 1.17E-83 | 4.50E-83 | 179.8641042 |
| AC135048.1 | -1.290315865 | 0.802447613 | -33.71657917 | 1.20E-83 | 4.65E-83 | 179.8310589 |
| AC020978.3 | 1.459792028 | 1.801054392 | 33.70572889 | 1.27E-83 | 4.90E-83 | 179.776892 |
| HBZ | -1.532217105 | 0.441513849 | -33.61073459 | 2.04E-83 | 7.81E-83 | 179.3021342 |
| PLCH2 | -1.416329823 | 0.691106677 | -33.61059015 | 2.04E-83 | 7.81E-83 | 179.3014117 |
| AP000753.3 | 1.670859208 | 1.653924631 | 33.57558647 | 2.43E-83 | 9.29E-83 | 179.126233 |
| MTCO1P40 | 1.868442741 | 2.042543713 | 33.44604049 | 4.65E-83 | 1.77E-82 | 178.4767897 |
| CFAP45 | -1.372052863 | 0.68877187 | -33.44221505 | 4.74E-83 | 1.80E-82 | 178.457585 |
| AP001628.1 | 1.554036578 | 1.575247767 | 33.39597305 | 5.97E-83 | 2.27E-82 | 178.2253167 |
| RN7SL182P | 1.592789995 | 1.60634769 | 33.37850045 | 6.51E-83 | 2.47E-82 | 178.137495 |
| AC022018.1 | 1.47509866 | 1.515970662 | 33.36708873 | 6.90E-83 | 2.61E-82 | 178.0801195 |
| FOSB | 1.50098026 | 2.709879409 | 33.353174 | 7.40E-83 | 2.79E-82 | 178.0101408 |
| AL031281.1 | 1.596448951 | 1.862433315 | 33.32310008 | 8.60E-83 | 3.24E-82 | 177.8588257 |
| AC013437.1 | 1.711372259 | 1.696418669 | 33.28495278 | 1.04E-82 | 3.92E-82 | 177.6667523 |
| COX6CP14 | 1.507524052 | 1.525854811 | 33.270872 | 1.12E-82 | 4.20E-82 | 177.5958161 |
| SNORD71 | 1.7596671 | 1.690997016 | 33.25648253 | 1.20E-82 | 4.51E-82 | 177.5233029 |
| TFF3 | -1.533080369 | 0.547225876 | -33.15246364 | 2.03E-82 | 7.58E-82 | 176.9984654 |
| CDK3 | -1.172490318 | 0.896848444 | -33.14955682 | 2.06E-82 | 7.68E-82 | 176.9837823 |
| RPL10P12 | 1.622964121 | 1.96849118 | 33.13620591 | 2.20E-82 | 8.20E-82 | 176.9163316 |
| KIR2DS4 | -1.589783168 | 0.6155572 | -33.10544185 | 2.57E-82 | 9.56E-82 | 176.760835 |
| RN7SL246P | 1.412527184 | 1.483568133 | 33.09779509 | 2.67E-82 | 9.92E-82 | 176.722169 |
| SNHG22 | -1.372347194 | 0.920074165 | -32.89964351 | 7.26E-82 | 2.69E-81 | 175.7180345 |
| AL359076.1 | 1.081358769 | 2.300952334 | 32.70125642 | 1.99E-81 | 7.32E-81 | 174.7084947 |
| C3 | -1.384618797 | 0.597209627 | -32.59326019 | 3.44E-81 | 1.26E-80 | 174.1571502 |
| ANG | -1.465252164 | 0.72634442 | -32.59240367 | 3.45E-81 | 1.27E-80 | 174.1527724 |
| AC022973.2 | 1.614028522 | 1.803270862 | 32.5659824 | 3.95E-81 | 1.45E-80 | 174.0176919 |
| MRPS24 | -1.405584378 | 1.14511516 | -32.54113684 | 4.49E-81 | 1.64E-80 | 173.8905984 |
| HSD11B1-AS1 | -1.409151931 | 0.665369641 | -32.50529187 | 5.38E-81 | 1.97E-80 | 173.7071216 |
| AL049869.3 | 1.415657058 | 1.561178087 | 32.49972052 | 5.54E-81 | 2.02E-80 | 173.6785915 |
| RNU6-645P | 1.72189893 | 1.66190011 | 32.49284114 | 5.74E-81 | 2.09E-80 | 173.6433585 |
| CEACAM3 | -2.091374514 | 0.869570949 | -32.45699387 | 6.89E-81 | 2.50E-80 | 173.4596824 |
| ARL4AP4 | 1.563003837 | 1.741875783 | 32.42773385 | 8.00E-81 | 2.89E-80 | 173.3096552 |
| AC009120.1 | 1.314990249 | 2.027225661 | 32.32727392 | 1.34E-80 | 4.81E-80 | 172.7938513 |
| ZBP1 | -1.549894144 | 0.956662387 | -32.31247034 | 1.44E-80 | 5.18E-80 | 172.7177506 |
| CCDC107 | -1.415780502 | 1.037178136 | -32.29082298 | 1.61E-80 | 5.78E-80 | 172.6064253 |
| MIR645 | 1.562546506 | 1.539133701 | 32.28278633 | 1.68E-80 | 6.01E-80 | 172.5650824 |
| RNU6-878P | 2.105261303 | 1.957245612 | 32.25014478 | 1.99E-80 | 7.10E-80 | 172.3970924 |
| AC007192.2 | -1.758299755 | 0.403908737 | -32.2337484 | 2.16E-80 | 7.70E-80 | 172.3126644 |
| CD68 | -2.047960367 | 1.224817026 | -32.22466889 | 2.26E-80 | 8.05E-80 | 172.2658997 |
| AC090617.7 | 1.598305331 | 1.566682592 | 32.19428489 | 2.65E-80 | 9.40E-80 | 172.1093393 |
| RN7SKP163 | 1.458155091 | 1.619109205 | 32.08098621 | 4.74E-80 | 1.68E-79 | 171.5246525 |
| AC127024.4 | 1.575190842 | 2.021240972 | 32.08056549 | 4.75E-80 | 1.68E-79 | 171.5224787 |
| SPCS2P4 | -1.215423839 | 0.967892343 | -32.04934276 | 5.58E-80 | 1.97E-79 | 171.3611028 |
| DGAT2 | -1.94406364 | 1.018773251 | -31.95580728 | 9.04E-80 | 3.18E-79 | 170.8770209 |
| FP565260.1 | -1.218627106 | 0.811753105 | -31.91094203 | 1.14E-79 | 4.00E-79 | 170.6444848 |
| MTCO1P53 | 1.605695044 | 1.572375687 | 31.86922005 | 1.41E-79 | 4.96E-79 | 170.4280415 |
| STX1A | -1.428724565 | 0.734134764 | -31.7634695 | 2.44E-79 | 8.55E-79 | 169.8785737 |
| RNU6-288P | 1.448933755 | 1.451605511 | 31.75420305 | 2.56E-79 | 8.96E-79 | 169.8303675 |
| RAB11FIP4 | -1.359034983 | 0.968473425 | -31.72141555 | 3.04E-79 | 1.06E-78 | 169.6597229 |
| GPR146 | -1.525580823 | 0.915957922 | -31.71590178 | 3.13E-79 | 1.09E-78 | 169.6310145 |
| HMGN2P28 | 1.468153596 | 1.511118659 | 31.70611346 | 3.29E-79 | 1.14E-78 | 169.5800415 |
| AC011933.1 | 1.538402062 | 1.764354934 | 31.69798427 | 3.43E-79 | 1.19E-78 | 169.5377005 |
| FP236383.3 | -2.734382516 | 1.377545457 | -31.67876971 | 3.79E-79 | 1.32E-78 | 169.437592 |
| RNU6-1141P | 1.696465321 | 1.64230585 | 31.60844555 | 5.47E-79 | 1.89E-78 | 169.0708519 |
| AC090114.3 | 1.020590964 | 2.165462042 | 31.53372883 | 8.06E-79 | 2.78E-78 | 168.6806031 |
| WLS | -1.816453313 | 0.681552716 | -31.51826426 | 8.74E-79 | 3.01E-78 | 168.5997535 |
| HIGD1AP16 | 1.42941573 | 1.510246567 | 31.50228496 | 9.50E-79 | 3.27E-78 | 168.5161848 |
| ZNF410 | -1.170474432 | 1.215282004 | -31.44301141 | 1.29E-78 | 4.44E-78 | 168.2059473 |
| AC005154.4 | 1.459762952 | 1.560160134 | 31.3791142 | 1.81E-78 | 6.19E-78 | 167.8710708 |
| H2AFZP1 | 1.401213886 | 1.494908183 | 31.34779108 | 2.13E-78 | 7.29E-78 | 167.7067442 |
| RN7SL648P | 1.758556537 | 1.778608545 | 31.34028099 | 2.21E-78 | 7.57E-78 | 167.6673286 |
| RNASEK | -1.194732983 | 2.0891785 | -31.28438314 | 2.97E-78 | 1.01E-77 | 167.373759 |
| AC004975.1 | 1.431256209 | 1.461431885 | 31.26893806 | 3.22E-78 | 1.09E-77 | 167.2925814 |
| AL035661.1 | -1.768995268 | 0.650691232 | -31.25361787 | 3.48E-78 | 1.18E-77 | 167.2120338 |
| PPIAP41 | 1.676119185 | 1.890980647 | 31.2387083 | 3.77E-78 | 1.28E-77 | 167.1336199 |
| RNA5SP474 | 1.437056229 | 1.442454968 | 31.23351889 | 3.87E-78 | 1.31E-77 | 167.1063214 |
| ARSG | -1.268376138 | 0.910238818 | -31.22913793 | 3.96E-78 | 1.34E-77 | 167.0832733 |
| AC002558.3 | 1.46054552 | 1.537605809 | 31.21298767 | 4.31E-78 | 1.46E-77 | 166.9982887 |
| AC093422.1 | 1.347642707 | 1.373570061 | 31.19252756 | 4.80E-78 | 1.62E-77 | 166.8905833 |
| AL353662.3 | 1.670695179 | 1.622452322 | 31.1799157 | 5.13E-78 | 1.73E-77 | 166.8241689 |
| SNORD46 | 1.627176929 | 1.635748523 | 31.17474158 | 5.27E-78 | 1.77E-77 | 166.7969166 |
| MTCO1P2 | 1.643008286 | 1.601122114 | 31.15855203 | 5.74E-78 | 1.93E-77 | 166.7116265 |
| RNU6ATAC24P | 1.606811256 | 1.64682708 | 31.1103265 | 7.39E-78 | 2.48E-77 | 166.4573891 |
| AC008649.2 | 1.493632378 | 1.532507959 | 31.07061823 | 9.11E-78 | 3.05E-77 | 166.247857 |
| ANKRD44-IT1 | 1.464166001 | 1.822373185 | 31.06390936 | 9.43E-78 | 3.15E-77 | 166.2124381 |
| IGKV1D-39 | -2.138817199 | 0.775231949 | -30.99277484 | 1.37E-77 | 4.58E-77 | 165.8365786 |
| RNU6-711P | 1.577318857 | 1.550514441 | 30.96193194 | 1.61E-77 | 5.37E-77 | 165.673434 |
| RNA5SP219 | 1.624944306 | 1.628708028 | 30.94890911 | 1.73E-77 | 5.75E-77 | 165.604517 |
| F8A3 | -1.44695771 | 0.444927708 | -30.9457554 | 1.76E-77 | 5.83E-77 | 165.5878246 |
| AC136475.9 | -1.643779565 | 0.789580018 | -30.92600741 | 1.95E-77 | 6.47E-77 | 165.4832745 |
| RPL23AP64 | 1.310888486 | 2.016011425 | 30.84577553 | 2.98E-77 | 9.86E-77 | 165.0580558 |
| PSMD9 | -1.121792584 | 1.380313723 | -30.79187029 | 3.96E-77 | 1.31E-76 | 164.7719556 |
| MT-TM | 2.00269079 | 1.878224451 | 30.71753989 | 5.88E-77 | 1.94E-76 | 164.376909 |
| MIR1273H | 1.618964891 | 1.582598886 | 30.68457635 | 7.00E-77 | 2.30E-76 | 164.2015155 |
| RNU7-75P | 1.682022008 | 1.631178604 | 30.65665469 | 8.12E-77 | 2.67E-76 | 164.0528524 |
| LIN37 | -1.175524319 | 1.143886936 | -30.57162134 | 1.28E-76 | 4.18E-76 | 163.5995631 |
| MME | -1.869040137 | 0.705651763 | -30.56773118 | 1.30E-76 | 4.27E-76 | 163.578806 |
| NLRP6 | -1.513247541 | 0.835783333 | -30.56375709 | 1.33E-76 | 4.35E-76 | 163.5575993 |
| PMS2P6 | -1.35171415 | 0.627316967 | -30.5323762 | 1.57E-76 | 5.13E-76 | 163.3900801 |
| TREML3P | -1.56817535 | 0.80050677 | -30.50830362 | 1.79E-76 | 5.83E-76 | 163.2614983 |
| AC079354.1 | 1.469635672 | 1.467554437 | 30.49377856 | 1.93E-76 | 6.29E-76 | 163.1838818 |
| MTND4P12 | 1.87127589 | 2.352602149 | 30.48856079 | 1.98E-76 | 6.45E-76 | 163.1559941 |
| FP671120.4 | -2.824853233 | 1.307846078 | -30.48840642 | 1.99E-76 | 6.45E-76 | 163.155169 |
| RN7SL285P | 1.404895581 | 1.46354938 | 30.45280735 | 2.40E-76 | 7.79E-76 | 162.9648171 |
| AC090543.2 | 1.445978788 | 1.638909841 | 30.45063221 | 2.43E-76 | 7.87E-76 | 162.9531817 |
| ILK | -1.328805611 | 1.512668594 | -30.44823605 | 2.46E-76 | 7.96E-76 | 162.9403634 |
| MIR4477B | 2.039094065 | 1.906269832 | 30.44244697 | 2.54E-76 | 8.20E-76 | 162.9093918 |
| AL357552.2 | 1.462563535 | 1.462106005 | 30.37737066 | 3.59E-76 | 1.16E-75 | 162.56097 |
| RNU6-101P | 1.633094717 | 1.59348462 | 30.32441818 | 4.76E-76 | 1.53E-75 | 162.2771018 |
| ATP5PDP4 | 1.511052899 | 2.123133928 | 30.31191662 | 5.09E-76 | 1.64E-75 | 162.2100363 |
| SLC37A3 | -1.45113242 | 0.92658421 | -30.30149323 | 5.38E-76 | 1.73E-75 | 162.1541056 |
| RNU6-890P | 1.594222056 | 1.563536804 | 30.29508527 | 5.57E-76 | 1.78E-75 | 162.1197151 |
| RPS15AP10 | 1.366861212 | 1.840372668 | 30.29112392 | 5.69E-76 | 1.82E-75 | 162.0984528 |
| SNORD70 | 1.632305331 | 1.59287647 | 30.27536029 | 6.19E-76 | 1.98E-75 | 162.0138245 |
| POLR2J3 | -1.393463647 | 1.349701209 | -30.26645944 | 6.49E-76 | 2.07E-75 | 161.966027 |
| EEF1A1P25 | 1.469812664 | 1.716781065 | 30.24889675 | 7.13E-76 | 2.27E-75 | 161.8716888 |
| AC127024.3 | 1.73402514 | 1.724915211 | 30.23500439 | 7.69E-76 | 2.44E-75 | 161.7970407 |
| RNA5SP283 | 2.000737763 | 1.915350578 | 30.19861392 | 9.34E-76 | 2.96E-75 | 161.6013977 |
| AL157871.5 | 1.257419049 | 1.460842044 | 30.17609934 | 1.05E-75 | 3.34E-75 | 161.4802782 |
| AP001363.1 | 1.84246494 | 1.775781464 | 30.13583038 | 1.31E-75 | 4.14E-75 | 161.2635016 |
| AC015849.3 | 1.083396762 | 2.028879322 | 30.1310108 | 1.34E-75 | 4.24E-75 | 161.2375442 |
| SNORD3A | -1.733701444 | 0.825278746 | -30.13039872 | 1.35E-75 | 4.25E-75 | 161.2342474 |
| SAA2 | -1.52526821 | 0.370526665 | -30.09243045 | 1.65E-75 | 5.20E-75 | 161.0296609 |
| AC020913.1 | 1.290861223 | 1.554502251 | 30.0745366 | 1.82E-75 | 5.72E-75 | 160.9331848 |
| AC009120.3 | 1.199667889 | 1.929858595 | 30.0557696 | 2.01E-75 | 6.32E-75 | 160.8319613 |
| ASGR1 | -1.4137714 | 0.876671474 | -30.04984577 | 2.08E-75 | 6.52E-75 | 160.8000015 |
| CDC42-IT1 | 1.334388817 | 1.735650986 | 30.04174114 | 2.17E-75 | 6.80E-75 | 160.7562695 |
| PPIAP42 | 1.426284484 | 1.54080572 | 29.98050759 | 3.01E-75 | 9.44E-75 | 160.4256121 |
| TRBC1 | -1.8867901 | 1.118890492 | -29.97740008 | 3.07E-75 | 9.58E-75 | 160.4088202 |
| AL162578.1 | 1.509791065 | 1.558745781 | 29.97544618 | 3.10E-75 | 9.67E-75 | 160.3982614 |
| AC080162.1 | 1.488717005 | 1.912697729 | 29.97408912 | 3.12E-75 | 9.73E-75 | 160.3909276 |
| RNU6-1048P | 1.49682788 | 1.488503536 | 29.92102103 | 4.15E-75 | 1.29E-74 | 160.1039728 |
| AC008982.2 | 1.233558221 | 2.016263998 | 29.88365276 | 5.08E-75 | 1.58E-74 | 159.9017157 |
| CREM | -1.175161641 | 1.232240976 | -29.86422862 | 5.64E-75 | 1.75E-74 | 159.7965179 |
| RC3H1-IT1 | 1.430245518 | 1.534357872 | 29.82637149 | 6.92E-75 | 2.14E-74 | 159.5913643 |
| AC119396.2 | 1.704228989 | 2.118587486 | 29.80299502 | 7.86E-75 | 2.43E-74 | 159.4646006 |
| AC021016.3 | -1.43268695 | 0.862757037 | -29.74894363 | 1.05E-74 | 3.25E-74 | 159.1712525 |
| ZMYND15 | -1.402300706 | 0.606398601 | -29.72497242 | 1.20E-74 | 3.69E-74 | 159.0410469 |
| HSPE1P13 | 1.435305442 | 1.481288904 | 29.7105931 | 1.30E-74 | 3.99E-74 | 158.96291 |
| AL591623.2 | 1.643880216 | 1.601793856 | 29.70753127 | 1.32E-74 | 4.05E-74 | 158.946269 |
| AC007216.2 | 1.329591555 | 1.527601731 | 29.68893854 | 1.46E-74 | 4.47E-74 | 158.8451941 |
| PDE4DIP | -1.20694514 | 0.900841513 | -29.66351295 | 1.67E-74 | 5.12E-74 | 158.706909 |
| AL138963.1 | 1.450009302 | 1.689279508 | 29.57087244 | 2.76E-74 | 8.46E-74 | 158.2024159 |
| AC137932.1 | 1.396950196 | 2.027507708 | 29.51938372 | 3.66E-74 | 1.12E-73 | 157.92159 |
| RNU6-88P | 1.631790068 | 1.592479507 | 29.50305292 | 4.00E-74 | 1.22E-73 | 157.832455 |
| MIR644A | 1.521706603 | 1.507670307 | 29.48027825 | 4.52E-74 | 1.38E-73 | 157.7080967 |
| CLDN5 | -1.370676947 | 0.669505656 | -29.47310486 | 4.70E-74 | 1.43E-73 | 157.6689147 |
| AC245060.2 | 1.658220715 | 2.184925681 | 29.46894723 | 4.81E-74 | 1.46E-73 | 157.6462024 |
| SNORA65 | 1.39990818 | 1.486737357 | 29.45415044 | 5.21E-74 | 1.58E-73 | 157.5653541 |
| MIR576 | 1.665040248 | 1.618095717 | 29.39946236 | 7.02E-74 | 2.13E-73 | 157.266321 |
| LY6G6F | -1.513094182 | 0.567251057 | -29.34437886 | 9.49E-74 | 2.86E-73 | 156.964771 |
| PLEK2 | -1.439432246 | 0.662003227 | -29.32997489 | 1.03E-73 | 3.09E-73 | 156.8858589 |
| AL161787.1 | 1.57208005 | 1.787588193 | 29.32676485 | 1.04E-73 | 3.14E-73 | 156.8682694 |
| MRPL53P1 | 1.395018295 | 1.899137791 | 29.29212442 | 1.26E-73 | 3.79E-73 | 156.6783791 |
| AC024940.4 | 1.575819328 | 1.587984704 | 29.26792554 | 1.44E-73 | 4.32E-73 | 156.5456431 |
| PVRIG | -1.362186916 | 1.052216773 | -29.22419506 | 1.83E-73 | 5.47E-73 | 156.3055972 |
| STEAP4 | -1.608888574 | 0.670788157 | -29.21180494 | 1.96E-73 | 5.85E-73 | 156.2375444 |
| AL139421.1 | 1.368959105 | 1.389992388 | 29.19855853 | 2.11E-73 | 6.28E-73 | 156.1647683 |
| RNU6-190P | 1.510609683 | 1.499121149 | 29.19322349 | 2.17E-73 | 6.46E-73 | 156.1354516 |
| LINC00106 | 1.21588872 | 2.14032915 | 29.18930075 | 2.22E-73 | 6.59E-73 | 156.1138935 |
| AC105052.3 | -1.483241988 | 0.558153418 | -29.17788152 | 2.36E-73 | 7.00E-73 | 156.0511269 |
| MTND4LP30 | 1.658432326 | 1.655883236 | 29.16004335 | 2.60E-73 | 7.71E-73 | 155.9530474 |
| KCNJ2 | -1.46653667 | 0.566634993 | -29.14363174 | 2.85E-73 | 8.43E-73 | 155.8627784 |
| KRT23 | -1.916114166 | 0.669507294 | -29.08504496 | 3.92E-73 | 1.16E-72 | 155.5402735 |
| AC022001.1 | 1.564501932 | 1.609130804 | 29.076649 | 4.11E-73 | 1.21E-72 | 155.4940228 |
| RN7SL382P | 1.435901248 | 1.687549149 | 29.03489762 | 5.17E-73 | 1.52E-72 | 155.2639035 |
| C18orf32 | -1.19929047 | 1.201765703 | -28.99820223 | 6.32E-73 | 1.86E-72 | 155.0614808 |
| AC098934.3 | 1.729960212 | 1.992288352 | 28.99177709 | 6.55E-73 | 1.93E-72 | 155.0260214 |
| RNU6-789P | 1.640818847 | 1.599435352 | 28.9877304 | 6.70E-73 | 1.97E-72 | 155.0036859 |
| MIR3671 | 1.803739776 | 1.724950966 | 28.98457139 | 6.82E-73 | 2.00E-72 | 154.9862484 |
| MIR548O | 1.579072335 | 1.551865335 | 28.97871516 | 7.04E-73 | 2.06E-72 | 154.9539196 |
| AL139396.1 | 1.675820525 | 1.688345821 | 28.97533411 | 7.17E-73 | 2.10E-72 | 154.9352529 |
| AC139451.1 | 1.458103341 | 1.586427348 | 28.91751056 | 9.86E-73 | 2.88E-72 | 154.6158009 |
| AC011465.1 | 1.350906254 | 1.433957495 | 28.8326051 | 1.57E-72 | 4.59E-72 | 154.1460137 |
| RNVU1-4 | 1.732444623 | 1.817135156 | 28.77972163 | 2.11E-72 | 6.14E-72 | 153.8529734 |
| AC090543.1 | 1.51197941 | 1.667475957 | 28.77902143 | 2.12E-72 | 6.16E-72 | 153.8490912 |
| RNA5SP282 | 1.662871616 | 1.616424985 | 28.73590265 | 2.69E-72 | 7.79E-72 | 153.6099098 |
| BPTFP1 | -1.424701132 | 0.536259059 | -28.73073829 | 2.76E-72 | 8.01E-72 | 153.5812481 |
| AC091231.1 | 1.353672125 | 1.378215174 | 28.71605093 | 3.00E-72 | 8.68E-72 | 153.4997172 |
| GADD45G | -1.661971177 | 0.775826475 | -28.71327567 | 3.04E-72 | 8.80E-72 | 153.4843086 |
| AC130895.1 | 1.46505352 | 1.521003747 | 28.71076998 | 3.09E-72 | 8.91E-72 | 153.4703959 |
| AL136131.3 | -1.879859696 | 0.431817907 | -28.67508898 | 3.76E-72 | 1.08E-71 | 153.272198 |
| GTF2IP23 | 1.512500497 | 1.646092281 | 28.66384322 | 4.00E-72 | 1.15E-71 | 153.2096995 |
| COX6CP1 | 1.484209809 | 1.660707732 | 28.6224024 | 5.04E-72 | 1.45E-71 | 152.9792613 |
| RNU7-45P | 1.653612246 | 1.609291491 | 28.59210294 | 5.96E-72 | 1.71E-71 | 152.8106468 |
| RPS27AP7 | 1.466818653 | 1.689300001 | 28.56471192 | 6.94E-72 | 1.99E-71 | 152.6581232 |
| AC146944.2 | -1.653964504 | 0.945594326 | -28.5070546 | 9.56E-72 | 2.73E-71 | 152.3367725 |
| AC004967.1 | 1.249756471 | 1.93959468 | 28.43319098 | 1.44E-71 | 4.10E-71 | 151.9245156 |
| AC092127.2 | 1.48636001 | 1.613551727 | 28.42090401 | 1.54E-71 | 4.39E-71 | 151.8558747 |
| UPK3BL1 | -1.837912242 | 0.873843163 | -28.39217429 | 1.81E-71 | 5.13E-71 | 151.695306 |
| ATP5MC2P3 | 1.405487345 | 1.601093143 | 28.34708305 | 2.33E-71 | 6.59E-71 | 151.4430943 |
| IGKV2-28 | -1.999595783 | 0.76894528 | -28.30757365 | 2.90E-71 | 8.19E-71 | 151.2219033 |
| EXT1 | -1.436592858 | 0.822061704 | -28.29671783 | 3.08E-71 | 8.69E-71 | 151.1610948 |
| RNU4ATAC | 1.67056881 | 1.658231509 | 28.27762023 | 3.43E-71 | 9.66E-71 | 151.0540859 |
| AC022167.3 | -1.202300959 | 0.836638541 | -28.26048124 | 3.78E-71 | 1.06E-70 | 150.9580143 |
| SNORA71B | 1.534583201 | 1.902480418 | 28.23170107 | 4.44E-71 | 1.24E-70 | 150.7966094 |
| AC093423.2 | 1.501284425 | 1.507073772 | 28.22590744 | 4.58E-71 | 1.28E-70 | 150.7641056 |
| PPIAP72 | 1.581092879 | 1.810689412 | 28.22556937 | 4.59E-71 | 1.28E-70 | 150.7622088 |
| AC027644.2 | 1.547982388 | 1.718434092 | 28.2250422 | 4.60E-71 | 1.29E-70 | 150.759251 |
| TNFRSF14-AS1 | -1.273384641 | 0.920620753 | -28.18784549 | 5.67E-71 | 1.58E-70 | 150.5504671 |
| AL445248.1 | 1.265284423 | 1.41292241 | 28.18524328 | 5.75E-71 | 1.60E-70 | 150.5358547 |
| U2AF1 | -1.696810372 | 1.067476165 | -28.12818155 | 7.92E-71 | 2.21E-70 | 150.2152275 |
| APOC3 | -1.584393117 | 0.40524774 | -28.09353938 | 9.61E-71 | 2.67E-70 | 150.0203834 |
| ASPRV1 | -1.225335312 | 0.889551649 | -28.05742904 | 1.18E-70 | 3.27E-70 | 149.8171275 |
| FCF1P7 | 1.304807039 | 1.578528061 | 28.03890673 | 1.31E-70 | 3.62E-70 | 149.7128091 |
| RNY4P7 | 1.505102833 | 1.494878627 | 28.02140122 | 1.44E-70 | 3.99E-70 | 149.6141794 |
| RNA5SP465 | 1.57181592 | 1.546274933 | 28.00686069 | 1.56E-70 | 4.33E-70 | 149.5322267 |
| RPL18P10 | 1.390297792 | 1.902650515 | 27.99949512 | 1.63E-70 | 4.50E-70 | 149.4907035 |
| CCZ1 | -1.107420909 | 1.18771777 | -27.99079506 | 1.71E-70 | 4.71E-70 | 149.4416488 |
| MIR4420 | 1.904795981 | 1.802805491 | 27.97219929 | 1.90E-70 | 5.23E-70 | 149.3367669 |
| MIR4748 | 1.496832529 | 1.488507118 | 27.96452835 | 1.98E-70 | 5.45E-70 | 149.29349 |
| SARNP | -1.17960063 | 1.167542682 | -27.96421755 | 1.99E-70 | 5.45E-70 | 149.2917364 |
| MIR135A1 | 1.396768229 | 1.411416763 | 27.96203887 | 2.01E-70 | 5.51E-70 | 149.2794436 |
| HM13-IT1 | 1.185743875 | 2.01561555 | 27.95222963 | 2.13E-70 | 5.82E-70 | 149.2240897 |
| RNU6-198P | 1.570424836 | 1.545203231 | 27.90614821 | 2.76E-70 | 7.53E-70 | 148.9638949 |
| SH2D3A | -1.247588168 | 0.848578315 | -27.89719099 | 2.90E-70 | 7.91E-70 | 148.9132889 |
| RNU5D-1 | 1.924988187 | 1.818361732 | 27.87942707 | 3.20E-70 | 8.73E-70 | 148.8128986 |
| UBE2V2P4 | 1.356665766 | 1.404811435 | 27.82610118 | 4.33E-70 | 1.18E-69 | 148.5113051 |
| RNU4-9P | 1.653469232 | 1.609181312 | 27.80689845 | 4.82E-70 | 1.31E-69 | 148.4026166 |
| FP236383.2 | -2.80721309 | 1.321436188 | -27.62172497 | 1.37E-69 | 3.72E-69 | 147.3522274 |
| AC002553.2 | 1.247214849 | 1.997333327 | 27.62117212 | 1.38E-69 | 3.73E-69 | 147.3490851 |
| AC134407.1 | 1.191147768 | 1.784361638 | 27.59574906 | 1.59E-69 | 4.30E-69 | 147.2045468 |
| RHOF | -1.329201937 | 1.114646203 | -27.59091773 | 1.64E-69 | 4.42E-69 | 147.1770703 |
| MIR7854 | 1.597354137 | 1.565949785 | 27.58257951 | 1.71E-69 | 4.62E-69 | 147.1296428 |
| ANKRD55 | -1.305353478 | 0.72043271 | -27.58174587 | 1.72E-69 | 4.64E-69 | 147.1249006 |
| RNU6-60P | 1.612741432 | 1.577804282 | 27.5775332 | 1.76E-69 | 4.75E-69 | 147.1009355 |
| MIR5584 | 2.204569028 | 2.033753094 | 27.57741491 | 1.77E-69 | 4.75E-69 | 147.1002625 |
| RNA5SP195 | 1.616760336 | 1.580900479 | 27.56633364 | 1.88E-69 | 5.05E-69 | 147.0372125 |
| MIR548C | 1.603872565 | 1.570971635 | 27.52570959 | 2.37E-69 | 6.34E-69 | 146.8059429 |
| STARD10 | -1.432923453 | 1.120934506 | -27.48397668 | 3.00E-69 | 8.02E-69 | 146.5681513 |
| ADM | -1.996940314 | 1.066073271 | -27.42740746 | 4.14E-69 | 1.10E-68 | 146.2454842 |
| AL157935.2 | -1.44656259 | 0.912441223 | -27.40008832 | 4.84E-69 | 1.29E-68 | 146.0895178 |
| SNORD19C | 1.507252903 | 1.496535058 | 27.28937142 | 9.09E-69 | 2.41E-68 | 145.4564959 |
| FCGR3B | -2.503356859 | 1.090824598 | -27.2704524 | 1.01E-68 | 2.68E-68 | 145.3481768 |
| CMTM2 | -1.913753442 | 1.163918811 | -27.26826352 | 1.03E-68 | 2.72E-68 | 145.3356417 |
| MTCO1P12 | 1.105686328 | 2.831689594 | 27.23517925 | 1.24E-68 | 3.28E-68 | 145.1461067 |
| AL031668.1 | 1.423494343 | 1.43200678 | 27.15185168 | 1.99E-68 | 5.27E-68 | 144.668141 |
| FTH1P11 | -1.371887686 | 0.907959312 | -27.14724661 | 2.05E-68 | 5.40E-68 | 144.6417016 |
| GATD3B | -1.263985382 | 1.109183044 | -27.13407862 | 2.21E-68 | 5.82E-68 | 144.5660849 |
| AP001189.1 | -1.51034413 | 0.593224661 | -27.10380592 | 2.63E-68 | 6.92E-68 | 144.3921644 |
| H19 | -1.575221149 | 0.553801204 | -27.09705566 | 2.73E-68 | 7.18E-68 | 144.353368 |
| NSUN7 | -1.50825731 | 0.601762078 | -27.08415328 | 2.94E-68 | 7.72E-68 | 144.2791974 |
| OGFOD2 | -1.045559077 | 1.081765788 | -27.06835016 | 3.22E-68 | 8.44E-68 | 144.1883236 |
| MYOM2 | -1.5206115 | 0.856420472 | -27.03358225 | 3.93E-68 | 1.03E-67 | 143.9882876 |
| REPS2 | -1.261736849 | 1.23921224 | -26.98324197 | 5.24E-68 | 1.37E-67 | 143.6983933 |
| AC104984.6 | 1.438087312 | 1.443249322 | 26.86503424 | 1.03E-67 | 2.70E-67 | 143.0164461 |
| SOX4 | 1.287566411 | 2.349746836 | 26.85143958 | 1.12E-67 | 2.91E-67 | 142.9379075 |
| HSPA6 | -2.078015617 | 1.195546005 | -26.80272719 | 1.48E-67 | 3.85E-67 | 142.6563009 |
| RNU6-606P | 1.419576437 | 1.428988393 | 26.75912123 | 1.90E-67 | 4.94E-67 | 142.4039665 |
| E2F3-IT1 | 1.404999052 | 1.473028065 | 26.750339 | 2.00E-67 | 5.19E-67 | 142.3531181 |
| AC113191.1 | 1.441221904 | 1.995104205 | 26.74361476 | 2.08E-67 | 5.39E-67 | 142.3141788 |
| RNU6-5P | 1.3353093 | 1.424901953 | 26.73927506 | 2.14E-67 | 5.52E-67 | 142.2890452 |
| AL691449.1 | 1.451639037 | 1.63073651 | 26.71460941 | 2.46E-67 | 6.35E-67 | 142.1461484 |
| GNGT2 | -1.384542069 | 0.874008171 | -26.66268244 | 3.32E-67 | 8.55E-67 | 141.8450718 |
| AC083798.2 | -1.001162489 | 1.27547924 | -26.66133372 | 3.35E-67 | 8.61E-67 | 141.8372474 |
| MIR23A | 1.983173035 | 1.863187813 | 26.60797195 | 4.56E-67 | 1.17E-66 | 141.5274959 |
| MIR1296 | 1.565517084 | 1.541422259 | 26.57900212 | 5.40E-67 | 1.38E-66 | 141.359186 |
| NEDD4L | -1.491506377 | 0.74885301 | -26.56475481 | 5.86E-67 | 1.50E-66 | 141.2763735 |
| SNRPGP14 | 1.344465286 | 1.444359213 | 26.48939504 | 9.07E-67 | 2.32E-66 | 140.837927 |
| ABCB6 | -1.278905621 | 0.882785247 | -26.48249787 | 9.44E-67 | 2.41E-66 | 140.7977638 |
| RNU6-652P | 1.374372536 | 1.394162939 | 26.44667285 | 1.16E-66 | 2.96E-66 | 140.5890552 |
| FAM20A | -1.396152505 | 0.829387137 | -26.41785202 | 1.37E-66 | 3.49E-66 | 140.421036 |
| RNU6-795P | 1.41225719 | 1.423349585 | 26.41634448 | 1.39E-66 | 3.52E-66 | 140.4122446 |
| RNU6-877P | 1.456443047 | 1.457390731 | 26.38430854 | 1.67E-66 | 4.23E-66 | 140.2253553 |
| CPXM1 | 1.480951584 | 2.388877803 | 26.32715871 | 2.33E-66 | 5.90E-66 | 139.8916421 |
| AC139887.1 | 1.286824993 | 1.579010237 | 26.31544321 | 2.49E-66 | 6.31E-66 | 139.8231822 |
| AL035413.1 | 1.29940658 | 1.582706257 | 26.22783983 | 4.16E-66 | 1.05E-65 | 139.3107289 |
| MIR5692A1 | 1.771064715 | 1.699777832 | 26.22376354 | 4.26E-66 | 1.07E-65 | 139.2868606 |
| MARK2P8 | 1.289499296 | 1.910924312 | 26.15984103 | 6.19E-66 | 1.56E-65 | 138.9122995 |
| RNU6-100P | 1.421136054 | 1.430189935 | 26.11936532 | 7.84E-66 | 1.97E-65 | 138.6748649 |
| AKR1C1 | -1.385311592 | 0.614332641 | -26.10803354 | 8.37E-66 | 2.10E-65 | 138.6083551 |
| AC011120.1 | 1.59299898 | 1.633677253 | 26.07573243 | 1.01E-65 | 2.53E-65 | 138.4186819 |
| COL9A3 | -1.345532939 | 0.721056533 | -26.03228991 | 1.30E-65 | 3.26E-65 | 138.1633813 |
| MTFP1 | -1.34466059 | 0.993058165 | -26.02522228 | 1.36E-65 | 3.40E-65 | 138.1218245 |
| RPL35AP32 | 1.415683271 | 1.657409686 | 25.9609305 | 1.98E-65 | 4.95E-65 | 137.7435106 |
| HP | -1.894785796 | 1.054390199 | -25.92334779 | 2.47E-65 | 6.17E-65 | 137.5221231 |
| AL157402.2 | 1.670856368 | 1.758294449 | 25.86111077 | 3.57E-65 | 8.88E-65 | 137.1551181 |
| MIR548L | 1.423531745 | 1.432035595 | 25.83464734 | 4.17E-65 | 1.04E-64 | 136.9989198 |
| RNU6-1223P | 1.399674786 | 1.413655999 | 25.82717629 | 4.35E-65 | 1.08E-64 | 136.9548067 |
| IGHJ3 | 2.099161855 | 1.952546547 | 25.82403025 | 4.43E-65 | 1.10E-64 | 136.9362287 |
| MIR210HG | -1.42793291 | 0.689065777 | -25.82230047 | 4.48E-65 | 1.11E-64 | 136.9260135 |
| AL627311.1 | 1.385853456 | 1.403007933 | 25.79250522 | 5.34E-65 | 1.32E-64 | 136.7499989 |
| RNU4-40P | 1.441266305 | 1.445698445 | 25.74980341 | 6.87E-65 | 1.70E-64 | 136.4975462 |
| AC087749.2 | 1.266858821 | 1.374398368 | 25.72365419 | 8.01E-65 | 1.97E-64 | 136.3428398 |
| FKBP1AP1 | 1.302072981 | 1.42215193 | 25.67910779 | 1.04E-64 | 2.57E-64 | 136.0790939 |
| SNORD94 | 1.570983272 | 1.875564854 | 25.63422726 | 1.36E-64 | 3.34E-64 | 135.813119 |
| MIR3685 | 1.522219165 | 1.508065189 | 25.62249529 | 1.46E-64 | 3.57E-64 | 135.7435505 |
| AL590762.1 | 1.174805074 | 1.711039581 | 25.58884671 | 1.78E-64 | 4.36E-64 | 135.5439249 |
| SAMD3 | -1.383069812 | 0.87991345 | -25.57415102 | 1.94E-64 | 4.75E-64 | 135.4566961 |
| A1BG | -1.21615365 | 0.98582634 | -25.54574743 | 2.29E-64 | 5.60E-64 | 135.2880249 |
| MIR3186 | 2.19654053 | 2.027567873 | 25.53831972 | 2.40E-64 | 5.85E-64 | 135.2438997 |
| CR391992.1 | 1.183794218 | 1.288360266 | 25.53602174 | 2.43E-64 | 5.92E-64 | 135.2302469 |
| PDZD4 | -1.440741181 | 0.850207224 | -25.53118423 | 2.50E-64 | 6.09E-64 | 135.201504 |
| CCZ1B | -1.092924671 | 1.235035712 | -25.52508005 | 2.59E-64 | 6.31E-64 | 135.1652308 |
| RNU7-84P | 1.600114092 | 1.568076077 | 25.5071257 | 2.88E-64 | 7.01E-64 | 135.0585127 |
| ALPL | -2.186265815 | 1.106357843 | -25.50540042 | 2.91E-64 | 7.07E-64 | 135.0482558 |
| ID3 | -1.415789157 | 0.716284489 | -25.48340581 | 3.32E-64 | 8.05E-64 | 134.9174633 |
| AC144530.1 | 1.158637879 | 1.574695336 | 25.47918122 | 3.40E-64 | 8.25E-64 | 134.8923345 |
| MGP | -1.44321209 | 0.68085341 | -25.40765689 | 5.20E-64 | 1.26E-63 | 134.4665537 |
| MTND4P24 | 1.69501573 | 1.641189073 | 25.40740095 | 5.21E-64 | 1.26E-63 | 134.4650289 |
| RNA5SP464 | 1.455622029 | 1.456758212 | 25.39150644 | 5.72E-64 | 1.38E-63 | 134.3703223 |
| AC002059.2 | 1.498775714 | 1.490004163 | 25.39099999 | 5.74E-64 | 1.38E-63 | 134.3673041 |
| MIR5581 | 1.564764389 | 1.540842377 | 25.3768417 | 6.24E-64 | 1.50E-63 | 134.282915 |
| ZBTB12BP | 1.08273876 | 1.569020377 | 25.36485017 | 6.71E-64 | 1.61E-63 | 134.2114211 |
| RNU4-39P | 1.369531232 | 1.390433159 | 25.34840374 | 7.40E-64 | 1.78E-63 | 134.1133376 |
| RNU6-268P | 1.342760805 | 1.369809003 | 25.32933733 | 8.28E-64 | 1.99E-63 | 133.9995867 |
| MTCYBP18 | 1.526860179 | 1.652222621 | 25.31400218 | 9.07E-64 | 2.18E-63 | 133.9080635 |
| RNU4-62P | 1.667283195 | 1.905719265 | 25.28224535 | 1.10E-63 | 2.62E-63 | 133.7184389 |
| RNU6-1306P | 1.35137272 | 1.376443693 | 25.28132758 | 1.10E-63 | 2.63E-63 | 133.7129569 |
| RNU6-1005P | 1.55485792 | 1.533210352 | 25.27966323 | 1.11E-63 | 2.66E-63 | 133.7030152 |
| AC245033.3 | -1.396100524 | 0.439653618 | -25.27707658 | 1.13E-63 | 2.70E-63 | 133.6875635 |
| RF02126 | 1.466020385 | 1.46476919 | 25.26642977 | 1.20E-63 | 2.87E-63 | 133.6239547 |
| AL603825.1 | 1.573674291 | 1.547706638 | 25.22175708 | 1.57E-63 | 3.74E-63 | 133.3569052 |
| MIR4677 | 1.478226746 | 1.47417307 | 25.21541941 | 1.63E-63 | 3.88E-63 | 133.3189989 |
| HIGD1AP17 | 1.29095217 | 1.391241588 | 25.1958248 | 1.83E-63 | 4.36E-63 | 133.2017695 |
| MIR3177 | 1.586260702 | 1.557403312 | 25.17453008 | 2.08E-63 | 4.94E-63 | 133.0743143 |
| RPP21 | -1.046289657 | 1.264115991 | -25.16768012 | 2.17E-63 | 5.14E-63 | 133.0333031 |
| SHISA4 | -1.403137026 | 0.722072687 | -25.09198449 | 3.41E-63 | 8.06E-63 | 132.5797168 |
| AL513365.2 | 1.165197477 | 1.703633162 | 25.07694192 | 3.73E-63 | 8.82E-63 | 132.4894926 |
| RNU6-834P | 1.482180896 | 1.477219379 | 25.07228525 | 3.84E-63 | 9.06E-63 | 132.4615565 |
| AC020656.1 | -1.702421594 | 0.391079567 | -25.06462918 | 4.02E-63 | 9.47E-63 | 132.4156205 |
| EIF1AX-AS1 | 1.392611211 | 1.492219637 | 25.06341938 | 4.05E-63 | 9.53E-63 | 132.4083611 |
| CYCSP10 | 1.297145961 | 1.511116874 | 25.05921346 | 4.15E-63 | 9.76E-63 | 132.3831221 |
| AC092683.1 | -1.229725183 | 0.834130478 | -25.01980875 | 5.25E-63 | 1.23E-62 | 132.1465532 |
| TIMM9P2 | 1.255096982 | 1.323875063 | 25.01100268 | 5.54E-63 | 1.30E-62 | 132.0936588 |
| AC005695.2 | 1.240697179 | 1.291178353 | 25.01014999 | 5.57E-63 | 1.31E-62 | 132.0885364 |
| RNU6-1011P | 1.429233919 | 1.436428597 | 25.00376018 | 5.78E-63 | 1.36E-62 | 132.0501487 |
| PBX1 | -1.522005635 | 0.528104796 | -24.99671583 | 6.03E-63 | 1.41E-62 | 132.0078227 |
| CHKB-CPT1B | -1.131372289 | 0.992953385 | -24.96327341 | 7.37E-63 | 1.72E-62 | 131.8067991 |
| AL591866.1 | 1.192581188 | 1.379235652 | 24.92848603 | 9.08E-63 | 2.12E-62 | 131.5975418 |
| RNU6-407P | 1.279314071 | 1.320929122 | 24.90207925 | 1.06E-62 | 2.48E-62 | 131.4385952 |
| SNORD13D | 1.395071059 | 1.41010925 | 24.87392085 | 1.26E-62 | 2.94E-62 | 131.2690088 |
| AC109583.2 | 1.281345666 | 1.35816882 | 24.84303552 | 1.52E-62 | 3.53E-62 | 131.0828847 |
| AC009303.4 | -1.230392801 | 0.845916506 | -24.84280501 | 1.52E-62 | 3.53E-62 | 131.0814951 |
| RNU6-94P | 1.434041578 | 1.440132456 | 24.83998362 | 1.55E-62 | 3.59E-62 | 131.0644866 |
| AC098591.2 | 1.266725272 | 1.718676814 | 24.82414861 | 1.70E-62 | 3.94E-62 | 130.9690076 |
| AC090519.1 | 1.043092352 | 1.739061594 | 24.81713048 | 1.77E-62 | 4.11E-62 | 130.9266809 |
| AC096720.1 | 1.413221871 | 1.501385899 | 24.81359944 | 1.81E-62 | 4.19E-62 | 130.9053827 |
| PDZK1IP1 | -1.906042103 | 0.937642495 | -24.79183007 | 2.06E-62 | 4.78E-62 | 130.7740416 |
| AC130456.3 | -1.870015639 | 0.819243859 | -24.78006583 | 2.22E-62 | 5.12E-62 | 130.7030397 |
| MT-TV | 1.686048659 | 1.634280769 | 24.77687038 | 2.26E-62 | 5.21E-62 | 130.6837509 |
| AC005261.5 | 1.18267849 | 2.000281042 | 24.76159788 | 2.48E-62 | 5.71E-62 | 130.5915431 |
| RNA5SP394 | 1.842020452 | 1.754442711 | 24.74554504 | 2.73E-62 | 6.27E-62 | 130.4945924 |
| AP001893.1 | 1.252071163 | 1.357399279 | 24.72492154 | 3.09E-62 | 7.09E-62 | 130.3699899 |
| CAPN3 | -1.112345979 | 1.179104066 | -24.72110653 | 3.16E-62 | 7.25E-62 | 130.3469346 |
| RNU4-82P | 1.252242854 | 1.36703692 | 24.66206308 | 4.51E-62 | 1.03E-61 | 129.9898835 |
| AC018644.1 | 1.137346707 | 1.949723227 | 24.65739136 | 4.64E-62 | 1.06E-61 | 129.9616137 |
| MIR548AR | 1.535960717 | 1.518651793 | 24.65409645 | 4.73E-62 | 1.08E-61 | 129.9416737 |
| CRISP3 | -1.663348106 | 0.613268429 | -24.65144071 | 4.81E-62 | 1.10E-61 | 129.9256008 |
| HNRNPA1P14 | 1.223352562 | 1.478700985 | 24.63636161 | 5.27E-62 | 1.20E-61 | 129.8343232 |
| GRAP | -1.151559262 | 0.967685425 | -24.63567603 | 5.29E-62 | 1.20E-61 | 129.8301726 |
| MEI1 | -1.118279014 | 1.270417407 | -24.63413642 | 5.34E-62 | 1.21E-61 | 129.8208512 |
| KRR1P1 | 1.092563838 | 1.720241183 | 24.61363153 | 6.04E-62 | 1.37E-61 | 129.6966783 |
| AL139246.5 | -1.215678984 | 0.861748788 | -24.59555419 | 6.74E-62 | 1.53E-61 | 129.5871623 |
| RNU6-1284P | 1.496836739 | 1.48851036 | 24.54876085 | 8.94E-62 | 2.02E-61 | 129.3034886 |
| AC093732.1 | 1.335324908 | 1.53836784 | 24.54844688 | 8.96E-62 | 2.03E-61 | 129.3015842 |
| AC135279.1 | 1.24451414 | 1.475445085 | 24.5320149 | 9.89E-62 | 2.23E-61 | 129.2019032 |
| CASP5 | -1.490318388 | 0.688364792 | -24.52289635 | 1.05E-61 | 2.36E-61 | 129.1465729 |
| RNU6-430P | 1.388815936 | 1.405290252 | 24.51862369 | 1.07E-61 | 2.42E-61 | 129.1206433 |
| FLT3 | 1.289209886 | 2.629946234 | 24.51489523 | 1.10E-61 | 2.47E-61 | 129.0980144 |
| MIR320B2 | 1.209500176 | 1.299525524 | 24.47999375 | 1.36E-61 | 3.05E-61 | 128.8861044 |
| DHRS13 | -1.263594187 | 1.253284426 | -24.45768569 | 1.55E-61 | 3.48E-61 | 128.7505772 |
| BICRA-AS1 | 1.167951591 | 1.331902423 | 24.44972204 | 1.63E-61 | 3.65E-61 | 128.7021809 |
| RNU6-1297P | 1.428331182 | 1.435733121 | 24.42345515 | 1.91E-61 | 4.28E-61 | 128.5424963 |
| RPL26P6 | 1.220572355 | 1.653454096 | 24.41638563 | 1.99E-61 | 4.46E-61 | 128.4995037 |
| SPACA6 | -1.296416853 | 0.884937408 | -24.40605693 | 2.12E-61 | 4.74E-61 | 128.4366794 |
| RN7SL481P | 1.420985271 | 1.885958771 | 24.38821369 | 2.36E-61 | 5.28E-61 | 128.3281162 |
| PMM1 | -1.21871869 | 1.069152631 | -24.38515323 | 2.41E-61 | 5.37E-61 | 128.3094916 |
| RNA5SP383 | 1.44437621 | 1.70151609 | 24.38355172 | 2.43E-61 | 5.42E-61 | 128.2997449 |
| AP002381.1 | 1.140702012 | 1.367053023 | 24.33866001 | 3.19E-61 | 7.11E-61 | 128.0264083 |
| AC079316.2 | 1.372307502 | 1.706980843 | 24.32994598 | 3.37E-61 | 7.49E-61 | 127.9733209 |
| SCARNA9 | 1.037254927 | 2.510534342 | 24.2758436 | 4.68E-61 | 1.04E-60 | 127.6435063 |
| AP001458.1 | 1.374067242 | 1.393927738 | 24.25721509 | 5.24E-61 | 1.16E-60 | 127.5298595 |
| EP300-AS1 | 1.150125169 | 1.806026661 | 24.25496062 | 5.31E-61 | 1.18E-60 | 127.5161027 |
| AL158801.1 | 1.345016383 | 1.671958278 | 24.24436011 | 5.67E-61 | 1.26E-60 | 127.4514097 |
| AC024896.1 | 1.016702444 | 2.19474333 | 24.23490915 | 6.00E-61 | 1.33E-60 | 127.3937204 |
| MIR623 | 1.490193256 | 1.483392167 | 24.22843213 | 6.24E-61 | 1.38E-60 | 127.3541777 |
| AL121753.1 | 1.190514227 | 1.527949775 | 24.20257892 | 7.31E-61 | 1.61E-60 | 127.1962893 |
| MFSD13B | 1.258542068 | 1.572961195 | 24.16417411 | 9.23E-61 | 2.04E-60 | 126.9615918 |
| SCARNA20 | 1.47070078 | 1.617451835 | 24.16138064 | 9.39E-61 | 2.07E-60 | 126.9445133 |
| CR936218.1 | 1.24955535 | 1.961983948 | 24.15354168 | 9.85E-61 | 2.17E-60 | 126.8965827 |
| ZBTB40-IT1 | 1.209134984 | 1.444762533 | 24.1491108 | 1.01E-60 | 2.22E-60 | 126.8694871 |
| MIR4706 | 1.329921486 | 1.359917488 | 24.12828665 | 1.15E-60 | 2.52E-60 | 126.7421106 |
| RPL9P30 | 1.305898299 | 1.39286717 | 24.04768782 | 1.88E-60 | 4.12E-60 | 126.2485927 |
| RNU6-1043P | 1.390741017 | 1.406773351 | 24.04606382 | 1.90E-60 | 4.15E-60 | 126.2386403 |
| AL078581.3 | 1.129708247 | 1.365833232 | 24.00420385 | 2.45E-60 | 5.36E-60 | 125.9819951 |
| PRKCZ | -1.347344074 | 0.899260848 | -23.99623528 | 2.57E-60 | 5.62E-60 | 125.9331145 |
| SH2D1B | -1.303562317 | 0.81780289 | -23.98476249 | 2.76E-60 | 6.03E-60 | 125.8627244 |
| NDUFA7 | -1.028023847 | 1.399177246 | -23.94325528 | 3.56E-60 | 7.75E-60 | 125.6079231 |
| AC011939.1 | 1.295321629 | 1.650271454 | 23.93657492 | 3.71E-60 | 8.07E-60 | 125.566894 |
| MRVI1 | -1.288136725 | 0.885578717 | -23.93623288 | 3.72E-60 | 8.08E-60 | 125.5647931 |
| AC092490.1 | -2.124813076 | 0.960086552 | -23.92658849 | 3.94E-60 | 8.56E-60 | 125.5055493 |
| AC008267.3 | 1.183653348 | 1.345571989 | 23.89156883 | 4.89E-60 | 1.06E-59 | 125.2903315 |
| AC087752.2 | 1.440876506 | 1.467658625 | 23.87256538 | 5.49E-60 | 1.19E-59 | 125.1734789 |
| PSMD10P1 | 1.09544057 | 1.823973949 | 23.8401163 | 6.70E-60 | 1.45E-59 | 124.9738439 |
| CCL4L2 | -1.572518865 | 0.82545516 | -23.81122402 | 8.00E-60 | 1.73E-59 | 124.7959798 |
| RNU6-1099P | 1.440188002 | 1.444867711 | 23.79870125 | 8.64E-60 | 1.87E-59 | 124.7188557 |
| RPS29P5 | 1.248730035 | 1.378328094 | 23.79775886 | 8.69E-60 | 1.88E-59 | 124.713051 |
| DUTP2 | 1.331903047 | 1.448840644 | 23.7812802 | 9.62E-60 | 2.07E-59 | 124.6115318 |
| CAMTA1 | -1.011119191 | 1.268327548 | -23.77392052 | 1.01E-59 | 2.17E-59 | 124.5661804 |
| MIR640 | 1.531950357 | 1.515562179 | 23.76487636 | 1.06E-59 | 2.29E-59 | 124.5104396 |
| AL353807.4 | 1.245310019 | 1.513258941 | 23.75288128 | 1.14E-59 | 2.46E-59 | 124.4364959 |
| AC009970.1 | 1.568614068 | 1.581825421 | 23.74510267 | 1.20E-59 | 2.58E-59 | 124.3885351 |
| STAG3L5P-PVRIG2P-PILRB | -1.189007733 | 1.416298075 | -23.70177591 | 1.57E-59 | 3.36E-59 | 124.1212545 |
| RNA5SP194 | 1.446230698 | 1.449523054 | 23.62739104 | 2.48E-59 | 5.30E-59 | 123.6618283 |
| MIR5692C2 | 1.589514228 | 1.559909855 | 23.60820273 | 2.79E-59 | 5.96E-59 | 123.5432021 |
| SAP25 | -1.264250061 | 1.352237654 | -23.59727443 | 2.98E-59 | 6.37E-59 | 123.4756203 |
| MIR3680-1 | 1.443251775 | 1.447228067 | 23.58779245 | 3.16E-59 | 6.75E-59 | 123.4169707 |
| IFI30 | -2.194256353 | 1.387813128 | -23.55501486 | 3.87E-59 | 8.25E-59 | 123.2141418 |
| RNU6ATAC16P | 1.309051471 | 1.343839057 | 23.54805018 | 4.04E-59 | 8.61E-59 | 123.1710268 |
| MIR6512 | 1.538847141 | 1.520875517 | 23.54431857 | 4.14E-59 | 8.80E-59 | 123.1479236 |
| AC120349.1 | 1.26207746 | 1.329069536 | 23.52834817 | 4.57E-59 | 9.70E-59 | 123.049028 |
| SNORD11B | 1.421017706 | 1.430098759 | 23.52535755 | 4.65E-59 | 9.87E-59 | 123.0305052 |
| AC069499.1 | 1.329388048 | 1.593283289 | 23.51769058 | 4.88E-59 | 1.03E-58 | 122.9830137 |
| AC106800.4 | 1.195609319 | 1.256442297 | 23.50370747 | 5.32E-59 | 1.13E-58 | 122.8963793 |
| AC073569.1 | 1.191715271 | 1.383949101 | 23.47639318 | 6.30E-59 | 1.33E-58 | 122.7270788 |
| MIR6819 | 1.439706133 | 1.444496475 | 23.46583175 | 6.72E-59 | 1.42E-58 | 122.6615914 |
| DES | -1.511959278 | 0.632476017 | -23.46039115 | 6.95E-59 | 1.47E-58 | 122.6278509 |
| IFI27 | -1.686094512 | 0.825227328 | -23.45439425 | 7.21E-59 | 1.52E-58 | 122.5906561 |
| AC099850.3 | 1.266229605 | 2.041854397 | 23.431396 | 8.32E-59 | 1.75E-58 | 122.4479715 |
| RN7SL587P | 1.430010101 | 1.895201924 | 23.43033709 | 8.37E-59 | 1.76E-58 | 122.4414002 |
| AC017104.3 | 1.533288739 | 1.586226036 | 23.42780056 | 8.50E-59 | 1.79E-58 | 122.4256588 |
| NPIPB5 | -1.197639998 | 1.394896594 | -23.37558446 | 1.17E-58 | 2.47E-58 | 122.1014324 |
| AC016590.2 | 1.312145638 | 1.484007456 | 23.36907173 | 1.22E-58 | 2.56E-58 | 122.0609689 |
| MTCO2P12 | 1.103558211 | 2.166649902 | 23.35176723 | 1.36E-58 | 2.85E-58 | 121.9534302 |
| AL512604.1 | 1.348795124 | 1.417391589 | 23.29700778 | 1.91E-58 | 4.00E-58 | 121.612881 |
| OLFM4 | -1.800118766 | 0.691673302 | -23.28821603 | 2.02E-58 | 4.21E-58 | 121.5581701 |
| AC011603.2 | -1.245920511 | 0.703018934 | -23.27876039 | 2.14E-58 | 4.46E-58 | 121.499317 |
| TRGJP1 | 2.047293666 | 1.912586871 | 23.26280795 | 2.36E-58 | 4.92E-58 | 121.4000016 |
| TRGJP2 | 2.125812613 | 1.973078508 | 23.23668978 | 2.78E-58 | 5.79E-58 | 121.2373285 |
| AC027018.1 | 1.302452476 | 1.394759192 | 23.21638204 | 3.15E-58 | 6.56E-58 | 121.1107858 |
| AC104763.3 | 1.246465184 | 1.594476849 | 23.21273216 | 3.23E-58 | 6.70E-58 | 121.088037 |
| MIR326 | 1.389667 | 1.405945919 | 23.20378023 | 3.41E-58 | 7.08E-58 | 121.0322347 |
| NSUN5P2 | -1.170431713 | 1.003945546 | -23.19468859 | 3.61E-58 | 7.48E-58 | 120.9755513 |
| GTDC1 | -1.000977966 | 1.222441289 | -23.15473695 | 4.63E-58 | 9.59E-58 | 120.7263434 |
| RNVU1-3 | 1.611491901 | 1.756364298 | 23.09736958 | 6.61E-58 | 1.37E-57 | 120.3681516 |
| AC087284.1 | 1.276217913 | 1.603834686 | 23.04840053 | 8.98E-58 | 1.85E-57 | 120.062072 |
| CMTM5 | -1.59657007 | 0.685484831 | -23.02401934 | 1.05E-57 | 2.16E-57 | 119.9095664 |
| MIR7848 | 1.596253153 | 1.565101578 | 22.9878026 | 1.31E-57 | 2.69E-57 | 119.6828917 |
| POLR2KP1 | 1.362150829 | 1.412278538 | 22.93124144 | 1.87E-57 | 3.83E-57 | 119.3285571 |
| RNU6-522P | 1.65699662 | 1.61189884 | 22.92758107 | 1.91E-57 | 3.92E-57 | 119.3056125 |
| LINC01127 | -1.581570643 | 0.755035718 | -22.90364888 | 2.22E-57 | 4.54E-57 | 119.1555551 |
| RPS18P12 | 1.383620953 | 1.739878782 | 22.89170287 | 2.39E-57 | 4.89E-57 | 119.0806257 |
| RNU6-828P | 1.268308996 | 1.312450722 | 22.81982023 | 3.75E-57 | 7.66E-57 | 118.6293774 |
| KLRD1 | -1.405948003 | 1.009974441 | -22.80964875 | 3.99E-57 | 8.16E-57 | 118.5654733 |
| EGR1 | 1.34199776 | 2.659535916 | 22.79990926 | 4.24E-57 | 8.65E-57 | 118.5042711 |
| RNU2-68P | 1.3334556 | 1.362640198 | 22.79456079 | 4.39E-57 | 8.93E-57 | 118.4706567 |
| AL353662.1 | 1.381210533 | 1.729083685 | 22.77590968 | 4.93E-57 | 1.00E-56 | 118.3534092 |
| SNORD121A | 1.328424332 | 1.358764067 | 22.77391033 | 4.99E-57 | 1.01E-56 | 118.340838 |
| FO393419.2 | 1.212177443 | 1.327004465 | 22.77060683 | 5.10E-57 | 1.03E-56 | 118.3200657 |
| ASLP1 | 1.126981955 | 1.686321965 | 22.76946275 | 5.14E-57 | 1.04E-56 | 118.3128715 |
| SYNE1 | -1.084735558 | 1.296823091 | -22.74891737 | 5.84E-57 | 1.18E-56 | 118.1836493 |
| ITCH-IT1 | 1.230917714 | 1.329975684 | 22.7271793 | 6.70E-57 | 1.35E-56 | 118.0468684 |
| NAMPTP1 | -1.580043313 | 1.007797092 | -22.69023182 | 8.44E-57 | 1.70E-56 | 117.8142515 |
| MIR938 | 1.448562736 | 1.451319675 | 22.68467504 | 8.74E-57 | 1.76E-56 | 117.779252 |
| MIR3680-2 | 1.309483637 | 1.344172001 | 22.64368036 | 1.13E-56 | 2.27E-56 | 117.5209275 |
| MIR1284 | 1.461715255 | 1.461452483 | 22.62430355 | 1.28E-56 | 2.56E-56 | 117.3987535 |
| RNU6-32P | 1.303790889 | 1.339786262 | 22.62409348 | 1.28E-56 | 2.57E-56 | 117.3974287 |
| AL138785.1 | 1.271615054 | 1.421110708 | 22.61729475 | 1.34E-56 | 2.68E-56 | 117.3545504 |
| AC087749.1 | 1.186120942 | 1.373101295 | 22.59887035 | 1.50E-56 | 3.00E-56 | 117.2383221 |
| AC127024.2 | 1.397844628 | 1.51085236 | 22.51426922 | 2.55E-56 | 5.11E-56 | 116.7040847 |
| AC008280.1 | 1.288839177 | 1.347491573 | 22.50749279 | 2.67E-56 | 5.33E-56 | 116.6612546 |
| FAM157C | -1.691885288 | 1.093006147 | -22.50138521 | 2.77E-56 | 5.53E-56 | 116.6226471 |
| ARF1P2 | 1.23271083 | 1.358190204 | 22.49903568 | 2.81E-56 | 5.61E-56 | 116.6077939 |
| AL513327.1 | -1.133676785 | 1.427907487 | -22.47640551 | 3.24E-56 | 6.46E-56 | 116.4646961 |
| AC078962.1 | 1.343830174 | 1.390151153 | 22.46940649 | 3.39E-56 | 6.75E-56 | 116.4204263 |
| ARHGAP24 | -1.331292771 | 0.870009308 | -22.46225294 | 3.55E-56 | 7.05E-56 | 116.3751727 |
| OLAH | -1.757325021 | 0.574302538 | -22.45801057 | 3.64E-56 | 7.24E-56 | 116.3483323 |
| KRT1 | -1.589565934 | 0.725545842 | -22.45073632 | 3.81E-56 | 7.56E-56 | 116.3023049 |
| MIR4766 | 1.342138482 | 1.369329561 | 22.44681929 | 3.91E-56 | 7.75E-56 | 116.2775174 |
| AC245884.3 | 1.328173654 | 1.666296559 | 22.43077705 | 4.33E-56 | 8.56E-56 | 116.17598 |
| NCR3 | -1.264995818 | 1.13786901 | -22.41662956 | 4.73E-56 | 9.36E-56 | 116.0864087 |
| NDUFA11 | -1.001031506 | 1.733338874 | -22.41360016 | 4.82E-56 | 9.53E-56 | 116.0672256 |
| PINK1 | -1.097187551 | 1.583835419 | -22.38945112 | 5.62E-56 | 1.11E-55 | 115.9142657 |
| AC093423.1 | 1.528177616 | 1.512655628 | 22.37487197 | 6.16E-56 | 1.21E-55 | 115.8218865 |
| SNORA68B | 1.464010428 | 1.497199825 | 22.33534365 | 7.91E-56 | 1.55E-55 | 115.5712875 |
| AC083862.2 | -1.082568862 | 1.120213123 | -22.32689343 | 8.34E-56 | 1.64E-55 | 115.5176903 |
| AC133550.1 | -1.353548028 | 0.490623435 | -22.32160082 | 8.62E-56 | 1.69E-55 | 115.4841163 |
| RNU1-1 | -1.557742937 | 0.754345756 | -22.31720884 | 8.87E-56 | 1.74E-55 | 115.456253 |
| MIR579 | 1.296058955 | 1.333829517 | 22.27657916 | 1.15E-55 | 2.24E-55 | 115.1983793 |
| AC132216.1 | 1.426245936 | 1.729934573 | 22.26453618 | 1.24E-55 | 2.42E-55 | 115.1219042 |
| MIR3155A | 1.343405762 | 1.370305884 | 22.25686135 | 1.30E-55 | 2.54E-55 | 115.0731583 |
| LCN2 | -2.13881925 | 1.0932419 | -22.18811333 | 2.01E-55 | 3.91E-55 | 114.6361883 |
| GTF2IP4 | -1.029872372 | 1.425900228 | -22.16316722 | 2.35E-55 | 4.57E-55 | 114.477484 |
| PPP4R1L | -1.157480542 | 1.097037707 | -22.13562845 | 2.80E-55 | 5.44E-55 | 114.3021965 |
| FFAR2 | -1.846846149 | 1.106167432 | -22.10708047 | 3.36E-55 | 6.51E-55 | 114.1203867 |
| AC019080.3 | 1.340771356 | 1.406185568 | 22.09936103 | 3.53E-55 | 6.83E-55 | 114.0712077 |
| MYB-AS1 | 1.47046892 | 1.574662864 | 22.08025619 | 3.98E-55 | 7.70E-55 | 113.949463 |
| AC005912.1 | 1.089279962 | 2.469663412 | 22.07606218 | 4.09E-55 | 7.90E-55 | 113.9227309 |
| FOLR3 | -1.959629514 | 0.959325113 | -22.06213533 | 4.47E-55 | 8.63E-55 | 113.8339473 |
| AC009961.2 | 1.262666939 | 1.308104035 | 22.06119987 | 4.50E-55 | 8.67E-55 | 113.8279829 |
| AC090617.9 | 1.260367068 | 1.306332195 | 22.05165034 | 4.78E-55 | 9.21E-55 | 113.7670898 |
| SLC16A6 | -1.308437009 | 0.806106156 | -22.03044656 | 5.47E-55 | 1.05E-54 | 113.6318427 |
| BEST1 | -1.302923206 | 1.20221921 | -22.0102155 | 6.22E-55 | 1.20E-54 | 113.5027486 |
| AL139099.3 | 1.251649338 | 1.343436214 | 22.00975146 | 6.24E-55 | 1.20E-54 | 113.499787 |
| MIR4744 | 1.445540067 | 1.448990986 | 21.99074062 | 7.04E-55 | 1.35E-54 | 113.3784322 |
| SNORA11B | 1.361461992 | 1.384216551 | 21.95411256 | 8.89E-55 | 1.71E-54 | 113.1444938 |
| AC079140.2 | 1.344293516 | 1.442373025 | 21.9491005 | 9.18E-55 | 1.76E-54 | 113.1124696 |
| PCOLCE2 | -1.462814474 | 0.559158773 | -21.94511148 | 9.42E-55 | 1.80E-54 | 113.0869799 |
| LIME1 | -1.237450873 | 1.412161983 | -21.93882411 | 9.80E-55 | 1.88E-54 | 113.0467998 |
| RPS27AP10 | 1.420180256 | 1.479270316 | 21.87434754 | 1.48E-54 | 2.82E-54 | 112.6344766 |
| DAAM2 | -1.574291819 | 0.577675206 | -21.8566812 | 1.66E-54 | 3.16E-54 | 112.5214128 |
| MIR601 | 1.297327034 | 1.334806456 | 21.82993817 | 1.96E-54 | 3.74E-54 | 112.3501859 |
| AC090739.1 | 1.011398156 | 1.680897511 | 21.82816965 | 1.99E-54 | 3.78E-54 | 112.3388596 |
| AC245100.8 | -1.120508116 | 1.008084728 | -21.81336674 | 2.18E-54 | 4.15E-54 | 112.2440406 |
| RBP7 | -1.548265326 | 0.897793858 | -21.81265551 | 2.19E-54 | 4.17E-54 | 112.2394842 |
| AC004000.1 | 1.198292786 | 1.435976995 | 21.80111696 | 2.36E-54 | 4.48E-54 | 112.1655553 |
| AL133477.1 | 1.243254732 | 1.47064148 | 21.78510099 | 2.62E-54 | 4.96E-54 | 112.0629121 |
| RNA5SP201 | 1.233750167 | 1.285826318 | 21.77193368 | 2.84E-54 | 5.39E-54 | 111.9785019 |
| AC068587.2 | 1.59197799 | 1.561807957 | 21.75691068 | 3.13E-54 | 5.93E-54 | 111.8821698 |
| SIAH2-AS1 | 1.149983367 | 1.521976759 | 21.7531026 | 3.21E-54 | 6.08E-54 | 111.8577469 |
| SCN1B | -1.244084945 | 0.830082887 | -21.74718451 | 3.33E-54 | 6.30E-54 | 111.819788 |
| C5AR2 | -1.544593361 | 1.030480112 | -21.73511758 | 3.60E-54 | 6.80E-54 | 111.7423769 |
| AC008937.2 | 1.325845502 | 1.465183578 | 21.71955915 | 3.98E-54 | 7.51E-54 | 111.6425412 |
| TTN-AS1 | -1.160405521 | 1.093526454 | -21.71541373 | 4.08E-54 | 7.70E-54 | 111.6159357 |
| CDH23 | -1.207773447 | 0.937596025 | -21.69963815 | 4.52E-54 | 8.51E-54 | 111.5146685 |
| NANOGP5 | 1.153009276 | 1.347917626 | 21.6922726 | 4.74E-54 | 8.92E-54 | 111.4673769 |
| AC010536.2 | 1.497654493 | 1.489140365 | 21.68914583 | 4.83E-54 | 9.09E-54 | 111.447299 |
| AC018638.3 | 1.445520527 | 1.448975932 | 21.68197988 | 5.06E-54 | 9.51E-54 | 111.4012799 |
| AC104695.3 | -1.318749595 | 0.827442324 | -21.67841439 | 5.18E-54 | 9.71E-54 | 111.3783803 |
| HMGB1P14 | 1.122273661 | 1.60829644 | 21.65840487 | 5.88E-54 | 1.10E-53 | 111.2498391 |
| RNA5SP39 | 1.505289441 | 1.541581483 | 21.65553587 | 5.99E-54 | 1.12E-53 | 111.2314047 |
| APBB1 | -1.314423305 | 0.93020427 | -21.62871323 | 7.12E-54 | 1.33E-53 | 111.0590103 |
| RPL22P3 | 1.143243745 | 1.396988249 | 21.62475508 | 7.30E-54 | 1.36E-53 | 111.0335631 |
| RIMKLBP2 | 1.175259709 | 1.755040995 | 21.62471304 | 7.30E-54 | 1.36E-53 | 111.0332928 |
| AC104564.1 | 1.310190127 | 1.553740519 | 21.60765883 | 8.15E-54 | 1.52E-53 | 110.9236282 |
| WASH7P | -1.039656225 | 1.247382527 | -21.59483024 | 8.84E-54 | 1.65E-53 | 110.8411126 |
| SLC26A11 | -1.221002826 | 0.99152105 | -21.58523604 | 9.41E-54 | 1.75E-53 | 110.7793881 |
| RNU6-315P | 1.294403502 | 1.332554142 | 21.5634461 | 1.08E-53 | 2.01E-53 | 110.6391605 |
| RNA5SP33 | 1.550137818 | 1.529573947 | 21.54949635 | 1.18E-53 | 2.20E-53 | 110.5493577 |
| PECR | -1.382458351 | 0.845998571 | -21.52083956 | 1.42E-53 | 2.64E-53 | 110.3648028 |
| TKTL1 | -1.508623555 | 0.654993356 | -21.48996237 | 1.73E-53 | 3.22E-53 | 110.1658368 |
| ATP6V0E1P1 | 1.427376029 | 1.55272005 | 21.48794871 | 1.76E-53 | 3.26E-53 | 110.1528572 |
| ASH1L-IT1 | 1.16201478 | 1.264548705 | 21.46210483 | 2.07E-53 | 3.84E-53 | 109.9862299 |
| HBQ1 | -1.610566964 | 1.218416181 | -21.46162889 | 2.08E-53 | 3.85E-53 | 109.9831606 |
| MTATP8P2 | 1.632560358 | 1.779055514 | 21.45628065 | 2.15E-53 | 3.98E-53 | 109.9486676 |
| ALG1L6P | 1.285047923 | 1.325346528 | 21.4467369 | 2.29E-53 | 4.23E-53 | 109.8871075 |
| MTLN | -1.147901481 | 1.050206417 | -21.4327767 | 2.51E-53 | 4.62E-53 | 109.7970402 |
| UBE2V1P1 | 1.308228754 | 1.58065267 | 21.42806485 | 2.58E-53 | 4.76E-53 | 109.7666353 |
| SNORA54 | 1.266828583 | 1.420823524 | 21.39183919 | 3.26E-53 | 5.99E-53 | 109.532787 |
| RNU6-141P | 1.471063069 | 1.468654115 | 21.37917018 | 3.54E-53 | 6.49E-53 | 109.4509671 |
| RPL21P123 | 1.222496141 | 1.825583853 | 21.33975945 | 4.56E-53 | 8.36E-53 | 109.1963182 |
| AC244669.1 | -1.19369637 | 1.074410556 | -21.3224669 | 5.10E-53 | 9.34E-53 | 109.0845249 |
| AC092755.1 | 1.240708425 | 1.291187017 | 21.3213606 | 5.14E-53 | 9.40E-53 | 109.0773717 |
| INKA2 | -1.132239074 | 1.200399588 | -21.28731067 | 6.40E-53 | 1.17E-52 | 108.8571352 |
| PCOLCE | -1.157863025 | 0.992180368 | -21.26953632 | 7.18E-53 | 1.31E-52 | 108.7421145 |
| SIGLEC5 | -1.511209236 | 1.266556498 | -21.26763041 | 7.27E-53 | 1.32E-52 | 108.7297788 |
| RNU6-1188P | 1.471764394 | 1.469194422 | 21.26323059 | 7.47E-53 | 1.36E-52 | 108.7012999 |
| RNU5E-4P | 1.841732634 | 1.79249257 | 21.26102019 | 7.58E-53 | 1.38E-52 | 108.6869918 |
| AC136475.1 | -1.201345083 | 0.874147489 | -21.25497042 | 7.88E-53 | 1.43E-52 | 108.6478279 |
| AL354726.1 | 1.281550537 | 1.437135973 | 21.25439029 | 7.91E-53 | 1.44E-52 | 108.6440721 |
| AC068888.2 | 1.122519455 | 1.311998595 | 21.23697822 | 8.86E-53 | 1.61E-52 | 108.5313273 |
| AC079316.1 | 1.374868964 | 1.536803281 | 21.23229228 | 9.13E-53 | 1.66E-52 | 108.5009792 |
| MIR5695 | 1.449070834 | 1.451711118 | 21.1791204 | 1.29E-52 | 2.33E-52 | 108.1564315 |
| AC013244.2 | 1.212779389 | 1.269670259 | 21.17858184 | 1.29E-52 | 2.33E-52 | 108.1529399 |
| AC090617.8 | 1.229144379 | 1.282277981 | 21.15667022 | 1.49E-52 | 2.69E-52 | 108.0108552 |
| ANXA3 | -1.8206554 | 1.083828226 | -21.15480521 | 1.51E-52 | 2.72E-52 | 107.9987589 |
| S1PR5 | -1.456306424 | 0.987622837 | -21.14127755 | 1.64E-52 | 2.96E-52 | 107.9110078 |
| FKBP1B | -1.21591705 | 0.845045015 | -21.13340386 | 1.73E-52 | 3.12E-52 | 107.8599228 |
| FAAP20 | -1.05832901 | 1.4260465 | -21.12536329 | 1.82E-52 | 3.28E-52 | 107.8077473 |
| RNU6-377P | 1.463925946 | 1.463155617 | 21.11064609 | 2.00E-52 | 3.61E-52 | 107.7122271 |
| AL353748.2 | 1.234326609 | 1.339151629 | 21.10308543 | 2.11E-52 | 3.78E-52 | 107.6631455 |
| OPLAH | -1.441619773 | 0.874865309 | -21.09143975 | 2.27E-52 | 4.07E-52 | 107.5875317 |
| AC131182.1 | 1.272160451 | 1.41670191 | 21.05551551 | 2.87E-52 | 5.14E-52 | 107.3541788 |
| TMIGD3 | -1.298951096 | 0.824955212 | -21.02570677 | 3.48E-52 | 6.22E-52 | 107.1604336 |
| SNORD15B | 1.406208349 | 1.905554782 | 21.00869951 | 3.88E-52 | 6.94E-52 | 107.0498455 |
| RF00422 | 1.22038986 | 1.304438639 | 21.00220168 | 4.05E-52 | 7.24E-52 | 107.007585 |
| TNFRSF10C | -1.711370864 | 1.38381035 | -20.94465228 | 5.88E-52 | 1.05E-51 | 106.6330763 |
| STX16-NPEPL1 | -1.0184901 | 1.181668332 | -20.92165987 | 6.83E-52 | 1.22E-51 | 106.4833409 |
| AC131934.1 | 1.091656312 | 1.46376111 | 20.87785605 | 9.08E-52 | 1.62E-51 | 106.1979003 |
| AL158832.1 | 1.37448544 | 1.425528609 | 20.87228473 | 9.41E-52 | 1.68E-51 | 106.1615794 |
| MIR3164 | 1.358363573 | 1.381829503 | 20.85114329 | 1.08E-51 | 1.92E-51 | 106.0237193 |
| SNORA30 | 1.217488317 | 1.273298056 | 20.8481717 | 1.10E-51 | 1.96E-51 | 106.0043378 |
| CR1L | -1.334197501 | 0.684113318 | -20.84338388 | 1.14E-51 | 2.02E-51 | 105.9731082 |
| COX5BP6 | 1.271721411 | 1.418836336 | 20.82509517 | 1.28E-51 | 2.27E-51 | 105.853791 |
| CLIC3 | -1.425844784 | 0.986662885 | -20.81877323 | 1.33E-51 | 2.36E-51 | 105.8125369 |
| MIR1537 | 1.600117422 | 1.568078642 | 20.80382097 | 1.47E-51 | 2.60E-51 | 105.7149465 |
| AC032044.2 | 1.243497237 | 1.728439142 | 20.80225757 | 1.48E-51 | 2.63E-51 | 105.7047411 |
| AL157827.1 | 1.289890905 | 1.329077601 | 20.78807703 | 1.63E-51 | 2.88E-51 | 105.6121606 |
| AC005072.1 | 1.109075267 | 1.483705496 | 20.78613028 | 1.65E-51 | 2.92E-51 | 105.599449 |
| VNN3 | -1.772019399 | 0.987548145 | -20.73453609 | 2.31E-51 | 4.08E-51 | 105.2623943 |
| RNU6-196P | 1.345558393 | 1.371964288 | 20.66552379 | 3.62E-51 | 6.39E-51 | 104.8110613 |
| NCR1 | -1.244103764 | 0.855579139 | -20.63388414 | 4.45E-51 | 7.84E-51 | 104.6039548 |
| HCAR2 | -1.643676567 | 1.20176619 | -20.61178134 | 5.14E-51 | 9.05E-51 | 104.4592049 |
| DMPK | -1.22391799 | 0.944085355 | -20.61062137 | 5.18E-51 | 9.11E-51 | 104.4516068 |
| RNU1-134P | 1.226426695 | 1.431230881 | 20.60277172 | 5.46E-51 | 9.58E-51 | 104.4001851 |
| IL1R2 | -2.311499375 | 1.078631429 | -20.58566326 | 6.10E-51 | 1.07E-50 | 104.2880856 |
| MIR320C1 | 1.323984402 | 1.355343509 | 20.58037538 | 6.32E-51 | 1.11E-50 | 104.253431 |
| AC245060.5 | 1.271533446 | 1.775252251 | 20.57878993 | 6.38E-51 | 1.12E-50 | 104.24304 |
| RNU6-322P | 1.434610909 | 1.440571074 | 20.57734177 | 6.44E-51 | 1.13E-50 | 104.2335485 |
| AL136320.1 | 1.405440239 | 1.909001894 | 20.5769192 | 6.46E-51 | 1.13E-50 | 104.2307789 |
| RNA5SP159 | 1.254694646 | 1.301962115 | 20.56130557 | 7.15E-51 | 1.25E-50 | 104.1284282 |
| MIR1972-1 | 1.319464086 | 1.351861021 | 20.55434611 | 7.49E-51 | 1.31E-50 | 104.0827983 |
| RN7SL128P | 1.227024056 | 1.387402106 | 20.53366712 | 8.57E-51 | 1.50E-50 | 103.9471826 |
| MIR378H | 1.503826055 | 1.493894987 | 20.5084974 | 1.01E-50 | 1.76E-50 | 103.7820489 |
| AC234772.2 | 1.441148909 | 1.980852281 | 20.50432263 | 1.04E-50 | 1.81E-50 | 103.7546518 |
| MIR3128 | 1.392266459 | 1.407948564 | 20.47577325 | 1.25E-50 | 2.18E-50 | 103.5672418 |
| SNORA22 | 1.275713663 | 1.35174508 | 20.46664368 | 1.33E-50 | 2.31E-50 | 103.5072915 |
| MIR6769A | 1.191130489 | 1.25299177 | 20.43337113 | 1.65E-50 | 2.87E-50 | 103.2887219 |
| AC005730.3 | 1.067853191 | 1.857569819 | 20.42620687 | 1.73E-50 | 3.01E-50 | 103.2416425 |
| MIR8055 | 1.217495495 | 1.273303586 | 20.41194638 | 1.90E-50 | 3.30E-50 | 103.1479133 |
| FCGR2B | -1.422504415 | 0.977948654 | -20.39085734 | 2.19E-50 | 3.79E-50 | 103.0092593 |
| AL049637.1 | 1.186555414 | 1.249467095 | 20.36717603 | 2.55E-50 | 4.42E-50 | 102.8535005 |
| FCGR1B | -1.474148524 | 1.039377729 | -20.36693454 | 2.56E-50 | 4.43E-50 | 102.8519119 |
| TNFRSF12A | -1.195325183 | 0.86283743 | -20.34892933 | 2.88E-50 | 4.98E-50 | 102.7334426 |
| TPM1 | -1.234266609 | 1.073779449 | -20.34806306 | 2.89E-50 | 5.00E-50 | 102.7277419 |
| SNORD53B | 1.357173182 | 1.380912416 | 20.29876149 | 4.00E-50 | 6.90E-50 | 102.4031552 |
| RNVU1-18 | -1.573702425 | 0.742050436 | -20.2735452 | 4.72E-50 | 8.14E-50 | 102.2370306 |
| AC012368.1 | -1.371729598 | 1.270842052 | -20.26670157 | 4.94E-50 | 8.51E-50 | 102.1919322 |
| ZC3HAV1L | 1.168543691 | 1.755514008 | 20.25011124 | 5.51E-50 | 9.48E-50 | 102.0825825 |
| AC108693.1 | 1.055389365 | 1.630087306 | 20.24362337 | 5.75E-50 | 9.88E-50 | 102.0398111 |
| RNU6-135P | 1.482688881 | 1.477610735 | 20.22818093 | 6.36E-50 | 1.09E-49 | 101.9379875 |
| IL18RAP | -1.655680506 | 1.279705974 | -20.13844857 | 1.15E-49 | 1.97E-49 | 101.3457742 |
| AC093484.4 | 1.301924582 | 1.64322935 | 20.13521778 | 1.17E-49 | 2.01E-49 | 101.3244345 |
| RNA5SP494 | 1.287022301 | 1.326867605 | 20.13506106 | 1.17E-49 | 2.01E-49 | 101.3233994 |
| MIR32 | 1.183308528 | 1.246965668 | 20.11106506 | 1.38E-49 | 2.36E-49 | 101.1648662 |
| RGL4 | -1.039117264 | 1.693625402 | -20.04263284 | 2.16E-49 | 3.69E-49 | 100.7123988 |
| MIR548K | 1.236236629 | 1.287741908 | 20.03763236 | 2.23E-49 | 3.81E-49 | 100.6793153 |
| MIR548AA2 | 1.34392862 | 1.370708697 | 20.00841708 | 2.71E-49 | 4.62E-49 | 100.4859683 |
| RNU6-1316P | 1.238121663 | 1.289194154 | 19.99829979 | 2.90E-49 | 4.94E-49 | 100.4189894 |
| AL353804.2 | 1.216677177 | 1.363190152 | 19.97716654 | 3.33E-49 | 5.66E-49 | 100.279045 |
| MANSC1 | -1.432922013 | 1.042755758 | -19.96601487 | 3.58E-49 | 6.09E-49 | 100.2051783 |
| BBC3 | -1.122144902 | 1.217892686 | -19.94131118 | 4.22E-49 | 7.16E-49 | 100.0414955 |
| AC063950.1 | 1.052519056 | 1.780937283 | 19.92764644 | 4.62E-49 | 7.83E-49 | 99.95092549 |
| AL357552.1 | 1.174369149 | 1.274349044 | 19.92171053 | 4.80E-49 | 8.14E-49 | 99.9115757 |
| SH3BP5 | -1.393703371 | 1.132997154 | -19.92077003 | 4.83E-49 | 8.19E-49 | 99.90534066 |
| RNU7-170P | 1.324010656 | 1.355363736 | 19.8991633 | 5.58E-49 | 9.43E-49 | 99.76207156 |
| RN7SL328P | 1.149369332 | 1.440320996 | 19.89593533 | 5.70E-49 | 9.63E-49 | 99.74066316 |
| AC141557.1 | 1.364207489 | 1.583690995 | 19.87268307 | 6.65E-49 | 1.12E-48 | 99.58641589 |
| AC103591.3 | 1.433107329 | 1.863482587 | 19.86576916 | 6.96E-49 | 1.17E-48 | 99.54053981 |
| SNORD72 | 1.211311627 | 1.268539484 | 19.8298319 | 8.83E-49 | 1.49E-48 | 99.30199771 |
| RNU6-761P | 1.429560905 | 1.481514298 | 19.82154352 | 9.32E-49 | 1.57E-48 | 99.24696112 |
| RPS15AP29 | 1.128177347 | 1.445791981 | 19.79333999 | 1.12E-48 | 1.89E-48 | 99.05962624 |
| AP003086.3 | 1.378514767 | 1.607503561 | 19.78528125 | 1.19E-48 | 1.99E-48 | 99.00608183 |
| TPTEP1 | -1.632448282 | 0.761965635 | -19.76745755 | 1.33E-48 | 2.24E-48 | 98.88763057 |
| GPR84 | -1.362896284 | 0.971617386 | -19.74315754 | 1.57E-48 | 2.63E-48 | 98.72608272 |
| MIR27A | 1.638081679 | 1.597326616 | 19.73078087 | 1.70E-48 | 2.85E-48 | 98.64377664 |
| RNU6-312P | 1.325447154 | 1.356470425 | 19.7208272 | 1.82E-48 | 3.04E-48 | 98.57757147 |
| RN7SKP80 | 1.167741765 | 1.774230973 | 19.71933943 | 1.84E-48 | 3.07E-48 | 98.56767484 |
| RNU6-613P | 1.298260375 | 1.335525509 | 19.67505216 | 2.46E-48 | 4.12E-48 | 98.27296478 |
| ACKR1 | -1.351010104 | 0.665814189 | -19.67342977 | 2.49E-48 | 4.16E-48 | 98.26216447 |
| SNORA38 | 1.233625735 | 1.320114869 | 19.6659929 | 2.62E-48 | 4.37E-48 | 98.21265312 |
| UQCRBP1 | 1.196764088 | 1.335186196 | 19.64553812 | 3.00E-48 | 5.00E-48 | 98.07644281 |
| AC092431.2 | 1.150544135 | 1.349540258 | 19.61118985 | 3.77E-48 | 6.28E-48 | 97.84761063 |
| MIR106B | 1.388174904 | 1.404796396 | 19.597195 | 4.13E-48 | 6.88E-48 | 97.75433807 |
| RN7SL431P | 1.192351279 | 1.895792654 | 19.58832466 | 4.39E-48 | 7.29E-48 | 97.69520807 |
| MIR624 | 1.376327199 | 1.395668827 | 19.58497441 | 4.48E-48 | 7.45E-48 | 97.67287293 |
| TPK1 | -1.091692187 | 1.089592787 | -19.57180086 | 4.89E-48 | 8.12E-48 | 97.58503672 |
| MIR1255A | 1.30598952 | 1.341480106 | 19.54599598 | 5.81E-48 | 9.62E-48 | 97.41292452 |
| AC005086.2 | 1.153610435 | 1.224086014 | 19.54164446 | 5.98E-48 | 9.89E-48 | 97.38389373 |
| RNY3P12 | 1.216724765 | 1.272709809 | 19.54078656 | 6.02E-48 | 9.94E-48 | 97.37817009 |
| LINC01506 | -1.40215175 | 0.925917978 | -19.5386086 | 6.10E-48 | 1.01E-47 | 97.36363903 |
| SNORA14B | 1.193603486 | 1.288824042 | 19.523053 | 6.77E-48 | 1.12E-47 | 97.25983914 |
| NFE4 | -1.278338048 | 0.764009687 | -19.51822594 | 6.99E-48 | 1.15E-47 | 97.22762361 |
| AL360181.1 | 1.269874673 | 1.313656932 | 19.50493301 | 7.64E-48 | 1.26E-47 | 97.13889419 |
| ABCC3 | -1.287768034 | 0.763016845 | -19.49955933 | 7.92E-48 | 1.30E-47 | 97.10301972 |
| PRSS57 | 1.276811352 | 2.676815698 | 19.49380518 | 8.23E-48 | 1.35E-47 | 97.06460182 |
| AC106037.2 | 1.112932799 | 1.651707733 | 19.47978131 | 9.03E-48 | 1.48E-47 | 96.97095542 |
| AL136310.1 | 1.147991778 | 1.388615689 | 19.45239718 | 1.08E-47 | 1.78E-47 | 96.7880322 |
| PTCRA | -1.356049636 | 0.751325901 | -19.44211372 | 1.16E-47 | 1.90E-47 | 96.71931869 |
| SCARNA7 | 1.520040038 | 2.042080525 | 19.4392871 | 1.18E-47 | 1.94E-47 | 96.70042931 |
| IL32 | -1.522003336 | 1.537276795 | -19.43793243 | 1.19E-47 | 1.96E-47 | 96.69137622 |
| MTND5P11 | 1.162378712 | 1.462370961 | 19.42531076 | 1.30E-47 | 2.12E-47 | 96.60701746 |
| SNORD36C | 1.327973127 | 1.358416455 | 19.41794403 | 1.36E-47 | 2.23E-47 | 96.55777283 |
| OASL | -1.349399162 | 0.991798249 | -19.40205263 | 1.52E-47 | 2.48E-47 | 96.45152306 |
| AL445524.1 | -1.259653323 | 1.268870047 | -19.35801159 | 2.04E-47 | 3.32E-47 | 96.1569221 |
| AC020908.3 | 1.070956099 | 1.578221041 | 19.35754207 | 2.04E-47 | 3.33E-47 | 96.15378021 |
| MMP9 | -2.022822868 | 1.235933539 | -19.35633063 | 2.06E-47 | 3.35E-47 | 96.14567359 |
| AC093809.1 | 1.50566696 | 1.495313235 | 19.35115678 | 2.13E-47 | 3.47E-47 | 96.11104979 |
| AATK | -1.30639043 | 1.205776216 | -19.3397119 | 2.30E-47 | 3.74E-47 | 96.03444949 |
| MTATP8P1 | 1.59800999 | 1.643230262 | 19.32257056 | 2.58E-47 | 4.19E-47 | 95.91969655 |
| AC005096.1 | 1.113082392 | 1.535032332 | 19.26381694 | 3.82E-47 | 6.19E-47 | 95.52612976 |
| RN7SL334P | 1.497059307 | 1.707925566 | 19.26373352 | 3.82E-47 | 6.19E-47 | 95.52557074 |
| AC090498.1 | -1.157190503 | 2.061695286 | -19.26286948 | 3.84E-47 | 6.22E-47 | 95.51978012 |
| SUMO2P8 | 1.190840808 | 1.296246979 | 19.24272024 | 4.40E-47 | 7.11E-47 | 95.3847212 |
| RNA5SP493 | 1.443775341 | 1.447631426 | 19.23714197 | 4.57E-47 | 7.37E-47 | 95.3473228 |
| AC092651.2 | 1.379948911 | 1.455610648 | 19.22089501 | 5.09E-47 | 8.21E-47 | 95.23837926 |
| VN1R83P | 1.143238812 | 1.62716509 | 19.21447867 | 5.31E-47 | 8.56E-47 | 95.19534692 |
| RNU7-70P | 1.378844215 | 1.397607957 | 19.1893414 | 6.29E-47 | 1.01E-46 | 95.02671707 |
| RNU7-18P | 1.484742525 | 1.47919288 | 19.18519505 | 6.46E-47 | 1.04E-46 | 94.99889539 |
| RNU6-107P | 1.230410981 | 1.283253782 | 19.180627 | 6.66E-47 | 1.07E-46 | 94.968242 |
| TNFSF14 | -1.265900049 | 1.112359514 | -19.16019412 | 7.64E-47 | 1.23E-46 | 94.83110237 |
| TDGF1P6 | 1.121432934 | 1.329070926 | 19.14353762 | 8.54E-47 | 1.37E-46 | 94.71927596 |
| PPP1R3B | -1.008475438 | 1.35063249 | -19.08079011 | 1.30E-46 | 2.08E-46 | 94.29774542 |
| AC114402.2 | 1.131555345 | 1.721728714 | 19.07624246 | 1.34E-46 | 2.14E-46 | 94.26717863 |
| TET2-AS1 | 1.286019192 | 1.391068177 | 19.07133166 | 1.39E-46 | 2.21E-46 | 94.23416855 |
| ZNF888 | 1.125363443 | 1.4590216 | 19.04374194 | 1.67E-46 | 2.66E-46 | 94.04866503 |
| MMP25 | -1.569325931 | 1.651508283 | -19.03692438 | 1.75E-46 | 2.78E-46 | 94.00281383 |
| ELMO1-AS1 | 1.269214394 | 1.377667696 | 19.02443211 | 1.90E-46 | 3.03E-46 | 93.91878499 |
| AC135507.2 | 1.322720713 | 1.51551269 | 19.01813731 | 1.98E-46 | 3.15E-46 | 93.876437 |
| AL353804.1 | 1.188835882 | 1.644294675 | 18.98907691 | 2.41E-46 | 3.83E-46 | 93.68088074 |
| AC022973.3 | 1.144247001 | 1.649828917 | 18.97522115 | 2.64E-46 | 4.20E-46 | 93.58761005 |
| AL122020.1 | 1.092792513 | 1.66301088 | 18.96543954 | 2.82E-46 | 4.48E-46 | 93.5217526 |
| BMX | -1.52715406 | 0.755676012 | -18.95170548 | 3.10E-46 | 4.91E-46 | 93.42926731 |
| HSF4 | -1.051804361 | 1.105377939 | -18.94643636 | 3.21E-46 | 5.09E-46 | 93.39377983 |
| MAPRE3 | -1.089644689 | 1.007214332 | -18.94093791 | 3.33E-46 | 5.27E-46 | 93.35674474 |
| AP001318.3 | 1.450237421 | 1.452609866 | 18.90699319 | 4.18E-46 | 6.60E-46 | 93.12803849 |
| RNU6-875P | 1.288018062 | 1.327634747 | 18.88600847 | 4.81E-46 | 7.60E-46 | 92.98659193 |
| RNU7-113P | 1.265110542 | 1.309986607 | 18.86614518 | 5.50E-46 | 8.67E-46 | 92.85266232 |
| TNFAIP6 | -1.426143111 | 0.964982301 | -18.86482713 | 5.55E-46 | 8.74E-46 | 92.84377384 |
| SNORD17 | 1.064894014 | 1.887574053 | 18.81945149 | 7.54E-46 | 1.19E-45 | 92.53766662 |
| GNG10 | -1.014630319 | 1.461248873 | -18.79504264 | 8.88E-46 | 1.40E-45 | 92.37291518 |
| ISG20 | -1.366648616 | 1.555339768 | -18.7730909 | 1.03E-45 | 1.62E-45 | 92.22469627 |
| AC130454.1 | 1.248437662 | 1.405995932 | 18.7542603 | 1.17E-45 | 1.84E-45 | 92.09751208 |
| WASH6P | -1.242606883 | 1.373958831 | -18.75407579 | 1.17E-45 | 1.84E-45 | 92.09626568 |
| RNU6-1322P | 1.301204109 | 1.337793386 | 18.71061808 | 1.57E-45 | 2.46E-45 | 91.80260814 |
| AC007298.2 | -1.147893773 | 0.93674993 | -18.68092563 | 1.92E-45 | 3.00E-45 | 91.60185643 |
| MIR548AQ | 1.70532807 | 1.649133784 | 18.66834386 | 2.09E-45 | 3.26E-45 | 91.51676371 |
| SNORD37 | 1.23413111 | 1.2861198 | 18.65946127 | 2.22E-45 | 3.46E-45 | 91.45667954 |
| MTCO3P12 | 1.067734249 | 2.172999841 | 18.64774752 | 2.40E-45 | 3.74E-45 | 91.37743247 |
| AL024508.1 | 1.094051826 | 1.436419697 | 18.63607883 | 2.60E-45 | 4.05E-45 | 91.29847647 |
| RNU6-1237P | 1.21130271 | 1.268532614 | 18.60326754 | 3.24E-45 | 5.05E-45 | 91.07638588 |
| RNU6-130P | 1.195384732 | 1.256269274 | 18.58765396 | 3.60E-45 | 5.61E-45 | 90.97066387 |
| AL162730.1 | 1.164489267 | 1.314876536 | 18.42086787 | 1.11E-44 | 1.73E-44 | 89.83981269 |
| MIR4263 | 1.191862226 | 1.253555506 | 18.4126736 | 1.18E-44 | 1.83E-44 | 89.78418255 |
| AC022022.1 | 1.275970561 | 1.344005215 | 18.3789424 | 1.48E-44 | 2.29E-44 | 89.55511527 |
| COL18A1 | -1.201437709 | 1.308424412 | -18.36533092 | 1.62E-44 | 2.51E-44 | 89.46264866 |
| RNU6-1034P | 1.181162937 | 1.245312687 | 18.36347457 | 1.64E-44 | 2.54E-44 | 89.45003656 |
| AC092801.1 | 1.302720689 | 1.338961771 | 18.34500398 | 1.86E-44 | 2.88E-44 | 89.32452838 |
| RNU6-1010P | 1.357487397 | 1.38115449 | 18.34003202 | 1.93E-44 | 2.98E-44 | 89.29073804 |
| MIR4505 | 1.277979088 | 1.31990064 | 18.29474764 | 2.62E-44 | 4.04E-44 | 88.98286708 |
| GLT1D1 | -1.730707736 | 1.045112699 | -18.28525439 | 2.79E-44 | 4.30E-44 | 88.91830094 |
| RNU6-742P | 1.336911328 | 1.365302518 | 18.2807254 | 2.88E-44 | 4.43E-44 | 88.88749496 |
| XCL2 | -1.245699624 | 0.859615364 | -18.24378286 | 3.70E-44 | 5.69E-44 | 88.63613985 |
| MIR3153 | 1.307891305 | 1.342945256 | 18.23205893 | 4.01E-44 | 6.15E-44 | 88.5563434 |
| FTH1P7 | -1.093782917 | 1.192622916 | -18.22817004 | 4.11E-44 | 6.31E-44 | 88.52987158 |
| ESPN | -1.381744605 | 0.68141484 | -18.22070044 | 4.33E-44 | 6.64E-44 | 88.47902164 |
| RF00134 | 1.204395184 | 1.263210999 | 18.21867536 | 4.39E-44 | 6.72E-44 | 88.46523477 |
| MIR3690 | 1.50426302 | 1.494231628 | 18.20009804 | 4.98E-44 | 7.62E-44 | 88.33874126 |
| SLC31A2 | -1.209654421 | 1.298751754 | -18.17576444 | 5.88E-44 | 8.99E-44 | 88.1730032 |
| HNRNPRP1 | 1.060188239 | 1.360862468 | 18.1601415 | 6.53E-44 | 9.98E-44 | 88.06656438 |
| RNU6-1019P | 1.323235139 | 1.354766271 | 18.14627999 | 7.18E-44 | 1.10E-43 | 87.97210665 |
| MIR4518 | 1.382109057 | 1.400123218 | 18.12900244 | 8.08E-44 | 1.23E-43 | 87.85434525 |
| CAMP | -1.708452107 | 1.010426402 | -18.12209933 | 8.46E-44 | 1.29E-43 | 87.80728664 |
| AC090970.1 | 1.187469657 | 1.250171435 | 18.10535138 | 9.49E-44 | 1.44E-43 | 87.69309699 |
| CXCR2 | -1.63043747 | 1.46156657 | -18.08553516 | 1.09E-43 | 1.65E-43 | 87.55795322 |
| AC141557.2 | 1.308648905 | 1.391526102 | 18.08259952 | 1.11E-43 | 1.68E-43 | 87.53792942 |
| VN1R81P | 1.333406834 | 1.546663068 | 18.0816226 | 1.11E-43 | 1.69E-43 | 87.53126576 |
| BNIP3P27 | 1.186661745 | 1.391093682 | 18.0808418 | 1.12E-43 | 1.70E-43 | 87.52593975 |
| LINC01145 | -1.079253261 | 1.208567762 | -18.0504699 | 1.38E-43 | 2.09E-43 | 87.31872266 |
| AC010240.2 | 1.057592196 | 1.56071674 | 18.01405488 | 1.77E-43 | 2.67E-43 | 87.07016152 |
| AP001052.1 | 1.191978433 | 1.253645033 | 17.99220108 | 2.05E-43 | 3.09E-43 | 86.92093281 |
| TREML1 | -1.45519772 | 1.038063726 | -17.96387134 | 2.49E-43 | 3.75E-43 | 86.72741703 |
| DEFA3 | -2.305467699 | 1.203208198 | -17.9508219 | 2.72E-43 | 4.10E-43 | 86.63825345 |
| MIR620 | 1.391991373 | 1.407736635 | 17.93405217 | 3.05E-43 | 4.59E-43 | 86.52364701 |
| IGHD3-9 | 1.993808105 | 1.871381158 | 17.90808824 | 3.64E-43 | 5.47E-43 | 86.3461551 |
| SCML4 | -1.126935584 | 0.981642047 | -17.87702759 | 4.50E-43 | 6.76E-43 | 86.1337403 |
| KIT | 1.419064164 | 2.227505328 | 17.82160571 | 6.57E-43 | 9.85E-43 | 85.75450736 |
| MT-TI | 1.588092834 | 1.558814801 | 17.79863467 | 7.69E-43 | 1.15E-42 | 85.59724273 |
| HLA-S | 1.24253493 | 1.292594171 | 17.79407253 | 7.93E-43 | 1.19E-42 | 85.56600372 |
| NADK2-AS1 | 1.089623877 | 1.394480522 | 17.7649759 | 9.67E-43 | 1.45E-42 | 85.3667217 |
| RNU6-258P | 1.433692767 | 1.439863729 | 17.76329442 | 9.79E-43 | 1.46E-42 | 85.35520297 |
| ROPN1L | -1.35700771 | 1.026137181 | -17.741241 | 1.14E-42 | 1.70E-42 | 85.20410569 |
| CD8B | -1.229876764 | 1.09265164 | -17.72624362 | 1.26E-42 | 1.88E-42 | 85.10132749 |
| RNU6-310P | 1.253806698 | 1.301278033 | 17.72469334 | 1.27E-42 | 1.90E-42 | 85.09070218 |
| CCL4 | -1.388750504 | 1.353997063 | -17.70704685 | 1.44E-42 | 2.14E-42 | 84.96974127 |
| AL355297.2 | 1.225321198 | 1.314994267 | 17.67799802 | 1.75E-42 | 2.61E-42 | 84.77056082 |
| RPS2P52 | 1.577115154 | 1.643851922 | 17.67093219 | 1.84E-42 | 2.74E-42 | 84.72210089 |
| RPL41P1 | 2.25323657 | 2.071246965 | 17.66525118 | 1.91E-42 | 2.84E-42 | 84.68313533 |
| SNORA19 | 1.156572189 | 1.226367773 | 17.61589113 | 2.68E-42 | 3.98E-42 | 84.34445951 |
| PHACTR2-AS1 | 1.0515773 | 1.368914656 | 17.56634389 | 3.77E-42 | 5.58E-42 | 84.0042847 |
| MIAT | -1.128629502 | 1.144256659 | -17.55709969 | 4.02E-42 | 5.94E-42 | 83.9407934 |
| MIR378E | 1.33938904 | 1.367211368 | 17.55575349 | 4.05E-42 | 5.99E-42 | 83.93154676 |
| ASS1P1 | 1.041369319 | 2.025959165 | 17.54700864 | 4.30E-42 | 6.36E-42 | 83.87147732 |
| LUCAT1 | -1.260799764 | 0.829554794 | -17.49075876 | 6.33E-42 | 9.34E-42 | 83.48493225 |
| AC018738.1 | 1.266012376 | 1.806596461 | 17.46557925 | 7.53E-42 | 1.11E-41 | 83.31181247 |
| RNU6-597P | 1.174870445 | 1.2404649 | 17.45200107 | 8.26E-42 | 1.22E-41 | 83.21843431 |
| MTND4LP5 | 1.31127905 | 1.345555203 | 17.44459571 | 8.69E-42 | 1.28E-41 | 83.16750047 |
| MTCO2P2 | 1.176365861 | 1.533836878 | 17.41010066 | 1.10E-41 | 1.62E-41 | 82.93018319 |
| RNA5SP260 | 1.159624625 | 1.228719395 | 17.38144733 | 1.34E-41 | 1.97E-41 | 82.73297912 |
| KIFC3 | -1.234036784 | 0.991126532 | -17.36047745 | 1.55E-41 | 2.27E-41 | 82.58861196 |
| CPNE5 | -1.065274093 | 1.130804105 | -17.31282287 | 2.15E-41 | 3.14E-41 | 82.26039758 |
| SYNE2 | -1.210012688 | 1.097596927 | -17.29551211 | 2.42E-41 | 3.54E-41 | 82.14112536 |
| AL627309.2 | -1.251030012 | 0.533977199 | -17.29361411 | 2.45E-41 | 3.58E-41 | 82.12804653 |
| CRIP1 | -1.453375756 | 1.499987105 | -17.27721096 | 2.75E-41 | 4.01E-41 | 82.01500236 |
| IGHD3-16 | 2.144932111 | 1.987808326 | 17.27558117 | 2.78E-41 | 4.05E-41 | 82.0037693 |
| HBA1 | -2.28187832 | 2.174116341 | -17.27446857 | 2.80E-41 | 4.08E-41 | 81.99610073 |
| IGHJ3P | 1.671161776 | 1.622811792 | 17.26346211 | 3.02E-41 | 4.39E-41 | 81.92023359 |
| AC020612.3 | 1.043248287 | 1.439772386 | 17.24490795 | 3.43E-41 | 4.99E-41 | 81.79231798 |
| PHOSPHO1 | -1.481422496 | 1.446446804 | -17.21946893 | 4.09E-41 | 5.94E-41 | 81.61689114 |
| AC078817.1 | 1.179715419 | 1.352858572 | 17.21673499 | 4.17E-41 | 6.05E-41 | 81.5980348 |
| AC007278.1 | -1.335981903 | 0.948356764 | -17.20176595 | 4.62E-41 | 6.69E-41 | 81.49478077 |
| CYSTM1 | -1.325132155 | 1.678834603 | -17.18627036 | 5.14E-41 | 7.44E-41 | 81.38787555 |
| SMIM1 | -1.323403474 | 1.122495285 | -17.16581218 | 5.92E-41 | 8.57E-41 | 81.24670332 |
| AL442663.2 | 1.193613358 | 1.339566226 | 17.12742576 | 7.71E-41 | 1.11E-40 | 80.98172608 |
| RPS15AP6 | 1.10948553 | 1.37232794 | 17.08324252 | 1.05E-40 | 1.51E-40 | 80.67658847 |
| AL139415.2 | 1.262633607 | 1.308078356 | 17.08091335 | 1.06E-40 | 1.53E-40 | 80.6604985 |
| AC005920.2 | 1.205751173 | 1.264255665 | 17.07195367 | 1.13E-40 | 1.63E-40 | 80.59860077 |
| MIR3136 | 1.228360584 | 1.281674139 | 17.06141117 | 1.22E-40 | 1.75E-40 | 80.52576013 |
| GZMM | -1.289724981 | 1.348771095 | -16.9987602 | 1.87E-40 | 2.70E-40 | 80.09270976 |
| RNA5SP151 | 1.263031238 | 1.347925112 | 16.96685726 | 2.33E-40 | 3.35E-40 | 79.87207595 |
| SNORA71D | 1.19466331 | 1.366519035 | 16.96340487 | 2.39E-40 | 3.43E-40 | 79.84819527 |
| SNORD51 | 1.172826575 | 1.238890285 | 16.96229726 | 2.41E-40 | 3.46E-40 | 79.84053356 |
| AC022022.2 | 1.152518919 | 1.280966072 | 16.95152106 | 2.60E-40 | 3.72E-40 | 79.76598615 |
| MIR613 | 1.445452371 | 1.448923424 | 16.95046515 | 2.61E-40 | 3.75E-40 | 79.7586811 |
| SLC22A18 | -1.094197678 | 1.383715257 | -16.94373174 | 2.74E-40 | 3.92E-40 | 79.71209583 |
| RNU11 | 1.361948413 | 1.384591293 | 16.88497335 | 4.11E-40 | 5.87E-40 | 79.30542716 |
| RNU1-2 | -1.263380308 | 0.981125128 | -16.88493152 | 4.11E-40 | 5.87E-40 | 79.30513756 |
| AC009032.1 | 1.044254591 | 1.546971407 | 16.87845989 | 4.30E-40 | 6.13E-40 | 79.26033121 |
| RNU6-553P | 1.219623635 | 1.274943123 | 16.86638422 | 4.67E-40 | 6.66E-40 | 79.17671674 |
| NAIP | -1.415657705 | 1.351555686 | -16.86193965 | 4.82E-40 | 6.86E-40 | 79.14593887 |
| AL627309.6 | -1.808014175 | 1.485465447 | -16.81405304 | 6.71E-40 | 9.55E-40 | 78.8142386 |
| RPL37P2 | 1.091108266 | 1.779158713 | 16.81397493 | 6.72E-40 | 9.55E-40 | 78.81369737 |
| IGFLR1 | -1.061888459 | 1.568244985 | -16.76075656 | 9.71E-40 | 1.38E-39 | 78.4448652 |
| MIR222 | 1.41185981 | 1.423043441 | 16.75959753 | 9.79E-40 | 1.39E-39 | 78.43683017 |
| IGHD3-3 | 2.11829712 | 1.967288512 | 16.73683033 | 1.15E-39 | 1.62E-39 | 78.2789757 |
| SLC6A8 | -1.276970571 | 1.067345652 | -16.6935838 | 1.55E-39 | 2.19E-39 | 77.97902587 |
| RNU6-1112P | 1.192671638 | 1.254179084 | 16.68582274 | 1.63E-39 | 2.31E-39 | 77.92518227 |
| RNU5F-1 | 1.507682463 | 1.536941046 | 16.67867039 | 1.71E-39 | 2.42E-39 | 77.8755579 |
| MT-TR | 1.369558623 | 1.390454261 | 16.66858696 | 1.84E-39 | 2.60E-39 | 77.80559095 |
| MTRNR2L12 | 1.263902316 | 1.818476378 | 16.66573648 | 1.87E-39 | 2.65E-39 | 77.78581071 |
| MTCO2P11 | 1.136582796 | 1.526072857 | 16.65190631 | 2.06E-39 | 2.91E-39 | 77.68983119 |
| HMGA1P4 | 1.149545589 | 1.612929411 | 16.64593789 | 2.15E-39 | 3.03E-39 | 77.64840689 |
| PLEKHF1 | -1.003284625 | 1.118728803 | -16.6444856 | 2.17E-39 | 3.06E-39 | 77.63832682 |
| MIR6797 | 1.191623234 | 1.253371385 | 16.6280863 | 2.43E-39 | 3.43E-39 | 77.52449162 |
| MIR3978 | 1.211814169 | 1.268926646 | 16.61352896 | 2.69E-39 | 3.79E-39 | 77.4234264 |
| CYP4F3 | -1.527831008 | 0.988965335 | -16.59759243 | 3.01E-39 | 4.23E-39 | 77.31276894 |
| RPS20P15 | 1.279079462 | 1.384544463 | 16.57741423 | 3.46E-39 | 4.85E-39 | 77.17263336 |
| RNU7-140P | 1.224464379 | 1.278672471 | 16.57084184 | 3.62E-39 | 5.08E-39 | 77.12698261 |
| B3GALNT1P1 | 1.087818507 | 1.295429302 | 16.54008591 | 4.48E-39 | 6.27E-39 | 76.91331726 |
| MIR635 | 1.227255522 | 1.28082279 | 16.53435546 | 4.66E-39 | 6.53E-39 | 76.87349983 |
| RNA5SP123 | 1.173986277 | 1.239783729 | 16.52339329 | 5.03E-39 | 7.04E-39 | 76.79732409 |
| MIR766 | 1.29548531 | 1.333387576 | 16.51953614 | 5.17E-39 | 7.22E-39 | 76.77051892 |
| AC010768.2 | 1.098781117 | 1.645347112 | 16.50518079 | 5.71E-39 | 7.98E-39 | 76.67074785 |
| MXRA7 | -1.195871455 | 1.047140438 | -16.43256985 | 9.45E-39 | 1.32E-38 | 76.16588046 |
| MIR93 | 1.281145076 | 1.322339743 | 16.42843686 | 9.73E-39 | 1.36E-38 | 76.13713293 |
| AC087481.2 | 1.244318043 | 1.313703195 | 16.41065915 | 1.10E-38 | 1.53E-38 | 76.01346479 |
| SNORD1B | 1.18160389 | 1.2456524 | 16.3333187 | 1.88E-38 | 2.62E-38 | 75.4752143 |
| MT1X | -1.248860818 | 1.23088425 | -16.32636363 | 1.98E-38 | 2.75E-38 | 75.4267914 |
| AC002400.1 | 1.023302542 | 1.478508017 | 16.31595919 | 2.13E-38 | 2.95E-38 | 75.35434732 |
| AC087741.1 | -1.166406519 | 1.028518843 | -16.3135004 | 2.16E-38 | 3.00E-38 | 75.33722623 |
| MTND6P4 | 1.336239585 | 1.569850515 | 16.30286352 | 2.33E-38 | 3.23E-38 | 75.26315483 |
| RNA5SP311 | 1.450004272 | 1.452430246 | 16.29896775 | 2.39E-38 | 3.31E-38 | 75.23602427 |
| RPL21P16 | -1.113006419 | 1.912844104 | -16.28757628 | 2.59E-38 | 3.58E-38 | 75.15668726 |
| AC109597.1 | 1.134448017 | 1.288639778 | 16.28024717 | 2.73E-38 | 3.76E-38 | 75.10563865 |
| MIR4762 | 1.237187281 | 1.288474299 | 16.2746892 | 2.83E-38 | 3.91E-38 | 75.066924 |
| AL355480.2 | 1.165863733 | 1.233526055 | 16.22787234 | 3.92E-38 | 5.41E-38 | 74.74073886 |
| PGLYRP1 | -1.559980252 | 1.48655227 | -16.22283385 | 4.07E-38 | 5.60E-38 | 74.70562618 |
| ALDOC | -1.072621006 | 1.305220801 | -16.21650543 | 4.25E-38 | 5.84E-38 | 74.66152191 |
| MIR4435-2HG | -1.031661057 | 1.560009156 | -16.18099712 | 5.44E-38 | 7.48E-38 | 74.41400989 |
| RNU6-142P | 1.167167623 | 1.234530582 | 16.0606227 | 1.26E-37 | 1.72E-37 | 73.57436413 |
| AC136628.3 | 1.100949044 | 1.843482532 | 16.0403013 | 1.45E-37 | 1.98E-37 | 73.43253143 |
| SNORD14E | 1.342853793 | 1.369880642 | 16.00640882 | 1.84E-37 | 2.51E-37 | 73.19592624 |
| SNORA12 | 1.350917145 | 1.537142214 | 16.00380084 | 1.87E-37 | 2.56E-37 | 73.17771704 |
| AC062037.2 | 1.282680469 | 1.323522622 | 15.99031432 | 2.05E-37 | 2.80E-37 | 73.08354639 |
| RNU5A-1 | 1.6166185 | 1.661196919 | 15.98362844 | 2.15E-37 | 2.94E-37 | 73.0368579 |
| CEACAM1 | -1.269610365 | 1.271022443 | -15.9367998 | 2.98E-37 | 4.07E-37 | 72.70977534 |
| AC008440.1 | 1.118766357 | 1.384813526 | 15.88237154 | 4.36E-37 | 5.94E-37 | 72.32945776 |
| AC068580.3 | -1.081984923 | 1.17083639 | -15.74607649 | 1.13E-36 | 1.54E-36 | 71.37639259 |
| STX8P1 | 1.286766258 | 1.326670348 | 15.72697318 | 1.29E-36 | 1.75E-36 | 71.24273176 |
| ANKRD9 | -1.075558685 | 1.067551686 | -15.70590155 | 1.50E-36 | 2.03E-36 | 71.09527749 |
| MGAM | -1.444118513 | 1.11516546 | -15.6633626 | 2.02E-36 | 2.73E-36 | 70.79753197 |
| RPL34P20 | 1.272442567 | 1.374978907 | 15.64383008 | 2.31E-36 | 3.13E-36 | 70.66078665 |
| EFEMP2 | -1.000927795 | 1.151943541 | -15.63739422 | 2.42E-36 | 3.27E-36 | 70.61572573 |
| MIR4659A | 1.284931232 | 1.325256628 | 15.62356386 | 2.66E-36 | 3.60E-36 | 70.51888501 |
| AC099677.1 | 1.485646849 | 1.592677211 | 15.62255972 | 2.68E-36 | 3.62E-36 | 70.51185365 |
| MIR4261 | 1.41686414 | 1.426898817 | 15.58475969 | 3.50E-36 | 4.72E-36 | 70.24712752 |
| FTH1P2 | -1.010342174 | 1.30122921 | -15.54643145 | 4.57E-36 | 6.16E-36 | 69.97863284 |
| MIR646 | 1.300560347 | 1.337297426 | 15.54171956 | 4.72E-36 | 6.36E-36 | 69.94562061 |
| RNU6-1024P | 1.217244964 | 1.273110575 | 15.53045306 | 5.11E-36 | 6.87E-36 | 69.86668168 |
| AC011445.2 | -1.197598837 | 1.092192949 | -15.52637535 | 5.26E-36 | 7.06E-36 | 69.83810968 |
| RNU6-437P | 1.172515488 | 1.238650622 | 15.49308481 | 6.64E-36 | 8.91E-36 | 69.60481861 |
| HIST2H2BD | -1.039829742 | 1.138283141 | -15.49042015 | 6.77E-36 | 9.08E-36 | 69.58614321 |
| AC020915.2 | 1.089598317 | 1.565045601 | 15.36706193 | 1.61E-35 | 2.15E-35 | 68.72123887 |
| AL021068.2 | 1.24011825 | 1.290732341 | 15.35893875 | 1.70E-35 | 2.27E-35 | 68.66426175 |
| LINC01503 | -1.156187736 | 0.970655406 | -15.35885581 | 1.70E-35 | 2.27E-35 | 68.66367994 |
| RNU6-920P | 1.178389646 | 1.243176121 | 15.34476924 | 1.88E-35 | 2.50E-35 | 68.56486817 |
| MIPEPP3 | 1.240806333 | 1.442193174 | 15.34365063 | 1.89E-35 | 2.52E-35 | 68.55702124 |
| AC015883.1 | 1.312353549 | 1.651895017 | 15.3192989 | 2.24E-35 | 2.99E-35 | 68.38618289 |
| TRBV29-1 | -1.180360719 | 1.09135694 | -15.30867854 | 2.42E-35 | 3.22E-35 | 68.31166873 |
| RNU6-82P | 1.191225662 | 1.253065092 | 15.30640458 | 2.46E-35 | 3.27E-35 | 68.29571373 |
| RNY1P12 | 1.156419825 | 1.226250392 | 15.26365664 | 3.32E-35 | 4.41E-35 | 67.99573773 |
| MYL9 | -1.191028118 | 1.247536399 | -15.2460494 | 3.75E-35 | 4.98E-35 | 67.87216117 |
| TBX21 | -1.144964585 | 1.129567024 | -15.23663875 | 4.01E-35 | 5.32E-35 | 67.80610753 |
| NAMPT | -1.071719096 | 2.2511443 | -15.21021342 | 4.82E-35 | 6.40E-35 | 67.62060926 |
| RNU6-681P | 1.186036508 | 1.249067326 | 15.19238445 | 5.47E-35 | 7.24E-35 | 67.49544005 |
| IGHJ2 | 1.624398314 | 1.586784839 | 15.17078753 | 6.36E-35 | 8.42E-35 | 67.34380195 |
| MTATP6P11 | 1.049888837 | 1.495524739 | 15.1252228 | 8.76E-35 | 1.16E-34 | 67.02382348 |
| TUBB2A | -1.234457737 | 1.013933981 | -15.07191633 | 1.27E-34 | 1.68E-34 | 66.64938568 |
| MAP1LC3A | -1.137393161 | 1.254679016 | -15.05596896 | 1.42E-34 | 1.88E-34 | 66.5373486 |
| MIR7111 | 1.20033479 | 1.260082839 | 15.03767429 | 1.62E-34 | 2.14E-34 | 66.40881022 |
| APBA2 | -1.148051792 | 0.985207929 | -15.00277278 | 2.07E-34 | 2.73E-34 | 66.16356189 |
| MT-TG | 1.327255926 | 1.357863918 | 14.98592149 | 2.33E-34 | 3.07E-34 | 66.04513616 |
| RNU6-194P | 1.394289829 | 1.409507384 | 14.98568079 | 2.33E-34 | 3.07E-34 | 66.04344455 |
| CREB5 | -1.285716945 | 1.24314377 | -14.9732857 | 2.55E-34 | 3.35E-34 | 65.95632985 |
| MIR6514 | 1.167145436 | 1.23451349 | 14.94959425 | 3.01E-34 | 3.95E-34 | 65.78980926 |
| AC114801.3 | 1.357489431 | 1.381156057 | 14.94468579 | 3.11E-34 | 4.09E-34 | 65.75530692 |
| THBD | -1.131943515 | 1.172169563 | -14.94160186 | 3.18E-34 | 4.17E-34 | 65.73362913 |
| QPCT | -1.552439329 | 1.266975936 | -14.91997719 | 3.70E-34 | 4.85E-34 | 65.58161537 |
| LINC01150 | 1.329940284 | 1.94689511 | 14.91573925 | 3.81E-34 | 4.99E-34 | 65.55182249 |
| FPR2 | -1.605676653 | 1.308988491 | -14.90354555 | 4.15E-34 | 5.44E-34 | 65.4660975 |
| KCNQ5-IT1 | 1.35832139 | 1.381797005 | 14.89846706 | 4.31E-34 | 5.63E-34 | 65.43039309 |
| F5 | -1.32013978 | 1.130732639 | -14.89175223 | 4.51E-34 | 5.90E-34 | 65.38318327 |
| HSPA1B | -1.214828488 | 1.832025502 | -14.86762095 | 5.35E-34 | 6.99E-34 | 65.21351312 |
| HIST2H2BF | -1.180461497 | 1.286962153 | -14.8480421 | 6.14E-34 | 8.02E-34 | 65.07583985 |
| AC015912.3 | -1.084037045 | 1.223511121 | -14.8450819 | 6.27E-34 | 8.18E-34 | 65.05502358 |
| PFKFB2 | -1.149829037 | 1.162019456 | -14.83446658 | 6.75E-34 | 8.81E-34 | 64.98037422 |
| FO393411.1 | 1.02410207 | 1.507887166 | 14.78964192 | 9.25E-34 | 1.20E-33 | 64.66512389 |
| MIR340 | 1.203830881 | 1.262776256 | 14.78241036 | 9.73E-34 | 1.27E-33 | 64.61425967 |
| IGHD2-2 | 1.806408544 | 1.727007006 | 14.77557096 | 1.02E-33 | 1.33E-33 | 64.5661526 |
| ARG1 | -1.115251877 | 1.664724805 | -14.75736292 | 1.16E-33 | 1.51E-33 | 64.43807482 |
| SH2D2A | -1.149402588 | 1.321865941 | -14.74286131 | 1.29E-33 | 1.67E-33 | 64.33606274 |
| LINC00869 | -1.005748516 | 1.335593313 | -14.63073395 | 2.83E-33 | 3.67E-33 | 63.54713497 |
| S100P | -1.620799254 | 1.581189618 | -14.61878674 | 3.08E-33 | 3.99E-33 | 63.46305837 |
| CD247 | -1.196597173 | 1.51619936 | -14.59674294 | 3.59E-33 | 4.65E-33 | 63.30792111 |
| MIR4639 | 1.161227328 | 1.229954131 | 14.57671627 | 4.14E-33 | 5.35E-33 | 63.16697148 |
| IL18R1 | -1.219277478 | 1.195067282 | -14.56798246 | 4.40E-33 | 5.69E-33 | 63.10549968 |
| MYL4 | -1.529753641 | 1.197208792 | -14.56732949 | 4.42E-33 | 5.71E-33 | 63.10090378 |
| UPP1 | -1.240961594 | 1.778178635 | -14.55986548 | 4.66E-33 | 6.01E-33 | 63.04836805 |
| MT1G | -1.367115909 | 0.694553036 | -14.52877122 | 5.80E-33 | 7.48E-33 | 62.82949921 |
| SLC15A3 | -1.520476061 | 1.398617318 | -14.5111702 | 6.56E-33 | 8.46E-33 | 62.70560014 |
| AC020951.1 | 1.174089086 | 1.681821702 | 14.49092404 | 7.57E-33 | 9.74E-33 | 62.56307469 |
| IGHD4-11 | 1.998641779 | 1.87510506 | 14.4560324 | 9.67E-33 | 1.24E-32 | 62.31743502 |
| IL1R1 | -1.257642404 | 1.03416785 | -14.45580612 | 9.69E-33 | 1.25E-32 | 62.31584197 |
| IGHD5-12 | 2.00218217 | 1.877832606 | 14.38798619 | 1.56E-32 | 2.01E-32 | 61.83833345 |
| IGHD1-1 | 1.937694639 | 1.828150886 | 14.36741402 | 1.80E-32 | 2.32E-32 | 61.69347615 |
| CAMK1 | -1.126477514 | 1.19358517 | -14.35932229 | 1.91E-32 | 2.45E-32 | 61.63649752 |
| HERC2P10 | 1.124592689 | 1.497688923 | 14.3430611 | 2.14E-32 | 2.75E-32 | 61.52199049 |
| HIST3H2A | -1.356645814 | 0.833408207 | -14.33094998 | 2.33E-32 | 2.99E-32 | 61.43670515 |
| BLK | -1.066142441 | 1.120164602 | -14.32367559 | 2.46E-32 | 3.14E-32 | 61.3854789 |
| RNU6-514P | 1.167540871 | 1.234818136 | 14.3091969 | 2.72E-32 | 3.48E-32 | 61.28351824 |
| CXCL1 | -1.235699854 | 0.964179043 | -14.27530996 | 3.45E-32 | 4.41E-32 | 61.04487413 |
| RNU6-1176P | 1.168586245 | 1.2356235 | 14.26290652 | 3.77E-32 | 4.81E-32 | 60.95752198 |
| AC005776.1 | 1.08307962 | 1.440380592 | 14.184881 | 6.53E-32 | 8.29E-32 | 60.40799347 |
| LILRA6 | -1.60814989 | 1.466155412 | -14.17445133 | 7.02E-32 | 8.92E-32 | 60.33453493 |
| NUAK2 | -1.013902765 | 1.292425104 | -14.15806166 | 7.88E-32 | 1.00E-31 | 60.2190975 |
| PLEKHG3 | -1.18117319 | 1.261251316 | -14.11737326 | 1.05E-31 | 1.33E-31 | 59.93251119 |
| RUBCNL | -1.17663611 | 1.20199968 | -14.04192345 | 1.79E-31 | 2.26E-31 | 59.40107114 |
| HBG2 | -1.811666007 | 1.58605864 | -14.01839495 | 2.11E-31 | 2.66E-31 | 59.23534348 |
| MYO7B | -1.199236942 | 0.996564193 | -13.97725226 | 2.82E-31 | 3.56E-31 | 58.94554683 |
| MIR2355 | 1.141418467 | 1.214693223 | 13.97708506 | 2.82E-31 | 3.56E-31 | 58.94436917 |
| AC026124.1 | 1.123292461 | 1.289435001 | 13.95382771 | 3.32E-31 | 4.19E-31 | 58.78055234 |
| DTX3 | -1.116030775 | 0.960647698 | -13.9430845 | 3.58E-31 | 4.51E-31 | 58.70488122 |
| RN7SL141P | 1.156945712 | 1.293878391 | 13.92258738 | 4.14E-31 | 5.21E-31 | 58.56050818 |
| KATNBL1P1 | 1.187017804 | 1.299035526 | 13.91872297 | 4.26E-31 | 5.35E-31 | 58.53328904 |
| CHI3L1 | -1.559294398 | 1.123822456 | -13.88805268 | 5.28E-31 | 6.63E-31 | 58.31726368 |
| IFIT1B | -1.299912709 | 0.867110619 | -13.88119264 | 5.54E-31 | 6.95E-31 | 58.2689457 |
| CLEC4E | -1.657261549 | 1.268062547 | -13.87219663 | 5.91E-31 | 7.40E-31 | 58.20558364 |
| ADGRE3 | -1.333287707 | 1.042432353 | -13.85526191 | 6.65E-31 | 8.33E-31 | 58.08630761 |
| LY9 | -1.000111262 | 1.331522734 | -13.81033925 | 9.13E-31 | 1.14E-30 | 57.76991325 |
| KCNE1 | -1.247941161 | 1.015482186 | -13.80739038 | 9.32E-31 | 1.16E-30 | 57.74914458 |
| GP9 | -1.249428024 | 1.086025794 | -13.79350664 | 1.03E-30 | 1.28E-30 | 57.65136341 |
| TMEM14EP | 1.053139382 | 1.751991209 | 13.76311371 | 1.27E-30 | 1.59E-30 | 57.43731601 |
| RNU6-610P | 1.135808003 | 1.210370875 | 13.71543952 | 1.78E-30 | 2.22E-30 | 57.1015801 |
| IGHD6-13 | 1.886891958 | 1.789012085 | 13.67581516 | 2.36E-30 | 2.93E-30 | 56.82255258 |
| EPB41L3 | -1.305172169 | 0.804628881 | -13.63836271 | 3.07E-30 | 3.81E-30 | 56.55883746 |
| TPST1 | -1.144680934 | 1.101925882 | -13.59424687 | 4.18E-30 | 5.19E-30 | 56.24822881 |
| MIR3161 | 1.338458372 | 1.366494374 | 13.59401337 | 4.19E-30 | 5.20E-30 | 56.24658488 |
| IGHD6-6 | 1.978526874 | 1.859608373 | 13.57924281 | 4.65E-30 | 5.77E-30 | 56.14259577 |
| RNA5SP450 | 1.134850377 | 1.209633112 | 13.56325319 | 5.20E-30 | 6.45E-30 | 56.03002822 |
| SUGT1P1 | 1.158122038 | 1.284067706 | 13.54876444 | 5.76E-30 | 7.14E-30 | 55.92803062 |
| RPS4XP22 | 1.408259402 | 1.533362021 | 13.51690606 | 7.21E-30 | 8.93E-30 | 55.70376773 |
| TSPAN5 | -1.041544356 | 1.190469584 | -13.49396751 | 8.48E-30 | 1.05E-29 | 55.54230668 |
| ALDH2 | -1.230209675 | 1.422565346 | -13.49132321 | 8.64E-30 | 1.07E-29 | 55.5236945 |
| AQP9 | -1.702464226 | 1.500615595 | -13.4791677 | 9.41E-30 | 1.16E-29 | 55.43813848 |
| GZMB | -1.314842722 | 1.517797878 | -13.46153909 | 1.07E-29 | 1.31E-29 | 55.31406566 |
| AC009244.1 | -1.020890177 | 1.157210888 | -13.45861497 | 1.09E-29 | 1.34E-29 | 55.2934859 |
| GYPB | -1.334875203 | 1.075013367 | -13.35400459 | 2.27E-29 | 2.80E-29 | 54.55737186 |
| AC016292.1 | 1.287107478 | 1.349484119 | 13.2988944 | 3.35E-29 | 4.11E-29 | 54.16968603 |
| IGHD4-4 | 1.982006208 | 1.86228888 | 13.29235326 | 3.51E-29 | 4.30E-29 | 54.12367628 |
| MIR3134 | 1.285424528 | 1.325636668 | 13.27995773 | 3.83E-29 | 4.69E-29 | 54.0364906 |
| ECHDC3 | -1.064552335 | 1.099669584 | -13.27050052 | 4.09E-29 | 5.02E-29 | 53.96997493 |
| RNA5SP187 | 1.148160185 | 1.259599169 | 13.24605902 | 4.86E-29 | 5.95E-29 | 53.79808201 |
| TFEB | -1.028472002 | 1.644266894 | -13.24176619 | 5.00E-29 | 6.13E-29 | 53.76789309 |
| RNA5SP498 | 1.404831092 | 1.654068919 | 13.23485537 | 5.25E-29 | 6.43E-29 | 53.71929455 |
| IGHD6-19 | 1.902407869 | 1.80096567 | 13.22222959 | 5.74E-29 | 7.03E-29 | 53.63051085 |
| AC007431.2 | 1.232029129 | 1.284500416 | 13.20537338 | 6.47E-29 | 7.91E-29 | 53.51198674 |
| CXCL16 | -1.299209579 | 1.487630432 | -13.18591633 | 7.41E-29 | 9.06E-29 | 53.37518608 |
| AC025040.1 | 1.125095123 | 1.655619424 | 13.18524562 | 7.45E-29 | 9.10E-29 | 53.3704706 |
| MT1F | -1.099883189 | 1.041871228 | -13.11018861 | 1.26E-28 | 1.54E-28 | 52.84287183 |
| RNA5SP168 | 1.134635535 | 1.209467597 | 13.08842133 | 1.47E-28 | 1.79E-28 | 52.68989979 |
| LILRB3 | -1.307099093 | 1.905954676 | -13.04222801 | 2.04E-28 | 2.48E-28 | 52.36532931 |
| AC133552.4 | 1.120899402 | 1.587469568 | 13.04059227 | 2.06E-28 | 2.51E-28 | 52.3538375 |
| C3orf86 | -1.060764924 | 1.583073117 | -13.03980883 | 2.07E-28 | 2.52E-28 | 52.34833351 |
| BAG3 | -1.184913534 | 1.053410742 | -13.00909633 | 2.57E-28 | 3.12E-28 | 52.13258508 |
| RNVU1-19 | 1.66114512 | 1.725688471 | 13.00575037 | 2.63E-28 | 3.20E-28 | 52.10908275 |
| CLDN9 | -1.071918442 | 1.072005769 | -12.96685178 | 3.46E-28 | 4.20E-28 | 51.83588885 |
| APOC1 | -1.227183788 | 0.855700899 | -12.92077819 | 4.78E-28 | 5.80E-28 | 51.51238575 |
| MIR4298 | 1.273199973 | 1.31621877 | 12.88114623 | 6.32E-28 | 7.66E-28 | 51.23418697 |
| H2AFJ | -1.057468119 | 1.590309632 | -12.85650745 | 7.51E-28 | 9.10E-28 | 51.06126992 |
| RNU6-1208P | 1.146969363 | 1.218969679 | 12.84407856 | 8.20E-28 | 9.92E-28 | 50.97405365 |
| LINC02207 | -1.123865502 | 1.108448476 | -12.79840137 | 1.13E-27 | 1.36E-27 | 50.65359064 |
| AL139020.1 | -1.110974366 | 1.069202749 | -12.77648541 | 1.32E-27 | 1.59E-27 | 50.49986875 |
| DMTN | -1.273237459 | 1.685745985 | -12.76565514 | 1.42E-27 | 1.72E-27 | 50.42391249 |
| VEGFA | -1.189538756 | 1.295499151 | -12.74423307 | 1.65E-27 | 1.99E-27 | 50.27369024 |
| CRISPLD2 | -1.213310274 | 1.160887763 | -12.7308076 | 1.82E-27 | 2.19E-27 | 50.17955637 |
| MIR181B2 | 1.237639067 | 1.288822358 | 12.70220459 | 2.22E-27 | 2.67E-27 | 49.97903564 |
| CRIP2 | -1.149060774 | 0.960946033 | -12.66470762 | 2.89E-27 | 3.47E-27 | 49.7162313 |
| MAL | -1.126208188 | 1.151671606 | -12.63757294 | 3.49E-27 | 4.19E-27 | 49.52610192 |
| SECTM1 | -1.455413781 | 1.521412168 | -12.57922076 | 5.26E-27 | 6.29E-27 | 49.11737913 |
| CCL3 | -1.207636471 | 1.366004453 | -12.57784577 | 5.31E-27 | 6.35E-27 | 49.1077506 |
| IGHD4-17 | 1.857561973 | 1.766416025 | 12.57420319 | 5.45E-27 | 6.51E-27 | 49.08224344 |
| IGHV1-3 | -1.173851574 | 0.844848292 | -12.57080085 | 5.58E-27 | 6.66E-27 | 49.05841925 |
| G0S2 | -1.53492619 | 1.321180963 | -12.53835186 | 7.01E-27 | 8.35E-27 | 48.8312376 |
| MPO | 1.124679969 | 3.011148675 | 12.5310112 | 7.38E-27 | 8.78E-27 | 48.77985321 |
| ADGRG3 | -1.213231939 | 1.930696366 | -12.51321837 | 8.36E-27 | 9.94E-27 | 48.6553179 |
| IGKJ5 | 1.385201943 | 1.402506002 | 12.49507505 | 9.49E-27 | 1.13E-26 | 48.52835004 |
| NCF1 | -1.421634934 | 1.800738757 | -12.48955431 | 9.86E-27 | 1.17E-26 | 48.48971981 |
| AC074135.1 | 1.135408704 | 1.287506319 | 12.47306977 | 1.11E-26 | 1.31E-26 | 48.37438437 |
| TNNT1 | -1.272439958 | 0.902546704 | -12.46703887 | 1.15E-26 | 1.37E-26 | 48.33219313 |
| HBG1 | -1.472690246 | 0.92110303 | -12.42755862 | 1.52E-26 | 1.81E-26 | 48.0560544 |
| IGHD3-10 | 1.729927034 | 1.668085027 | 12.42669857 | 1.53E-26 | 1.82E-26 | 48.05004009 |
| RNU6-1004P | 1.131859242 | 1.207328717 | 12.38215825 | 2.09E-26 | 2.48E-26 | 47.73863838 |
| HBM | -1.551803882 | 1.555097016 | -12.34430975 | 2.73E-26 | 3.22E-26 | 47.47413062 |
| MIR3619 | 1.22006741 | 1.27528501 | 12.32758018 | 3.07E-26 | 3.62E-26 | 47.357247 |
| APOBEC3A | -1.453087321 | 1.622578129 | -12.32591769 | 3.10E-26 | 3.66E-26 | 47.34563287 |
| SLC11A1 | -1.507761118 | 1.908487734 | -12.31866788 | 3.26E-26 | 3.85E-26 | 47.29498814 |
| AC005392.2 | -1.408757231 | 1.136838905 | -12.31233787 | 3.41E-26 | 4.02E-26 | 47.25077194 |
| DEFA4 | -1.551469934 | 1.182060805 | -12.29099939 | 3.96E-26 | 4.66E-26 | 47.1017407 |
| VNN2 | -1.354558884 | 1.930296093 | -12.25120386 | 5.23E-26 | 6.15E-26 | 46.82389328 |
| SLC43A2 | -1.054015168 | 1.707499995 | -12.21525201 | 6.72E-26 | 7.90E-26 | 46.57298564 |
| SIPA1L2 | -1.135129686 | 1.151320179 | -12.21154408 | 6.90E-26 | 8.10E-26 | 46.54711376 |
| KYNU | -1.054573665 | 1.136717821 | -12.08930019 | 1.62E-25 | 1.89E-25 | 45.69478118 |
| AC141424.1 | 1.154535296 | 1.759370776 | 12.00914152 | 2.83E-25 | 3.31E-25 | 45.13656401 |
| TRBVB | 1.681421869 | 1.630716251 | 11.99268004 | 3.18E-25 | 3.71E-25 | 45.02199764 |
| IGHD1-7 | 1.732283385 | 1.669900379 | 11.99113442 | 3.21E-25 | 3.75E-25 | 45.0112419 |
| IGHD5-5 | 1.811672603 | 1.73106248 | 11.98562083 | 3.34E-25 | 3.89E-25 | 44.97287539 |
| IGHD2-15 | 1.646623801 | 1.603907536 | 11.96761453 | 3.79E-25 | 4.41E-25 | 44.84759695 |
| NPL | -1.08944864 | 1.539909048 | -11.95784066 | 4.05E-25 | 4.72E-25 | 44.77960785 |
| MIR6503 | 1.455265161 | 1.456483278 | 11.95697015 | 4.08E-25 | 4.75E-25 | 44.77355284 |
| IGHD3-22 | 1.551603108 | 1.530702819 | 11.94598051 | 4.40E-25 | 5.12E-25 | 44.69711788 |
| MIR4480 | 1.141392696 | 1.214673369 | 11.92497894 | 5.10E-25 | 5.92E-25 | 44.55107923 |
| RNASE1 | -1.260308041 | 1.241288025 | -11.86254882 | 7.87E-25 | 9.13E-25 | 44.11720398 |
| MIR606 | 1.206625052 | 1.264928908 | 11.84819856 | 8.70E-25 | 1.01E-24 | 44.01752577 |
| MIR320C2 | 1.168636785 | 1.235662437 | 11.78724644 | 1.33E-24 | 1.54E-24 | 43.59437285 |
| SCML2P2 | 1.064842041 | 1.425594563 | 11.76973806 | 1.50E-24 | 1.74E-24 | 43.47289173 |
| TGFBI | -1.368296835 | 1.281838502 | -11.71230263 | 2.24E-24 | 2.59E-24 | 43.07459978 |
| HBA2 | -1.352185057 | 2.941193582 | -11.68956195 | 2.62E-24 | 3.03E-24 | 42.91699744 |
| FPR1 | -1.540235048 | 1.927034656 | -11.68332779 | 2.74E-24 | 3.16E-24 | 42.87380171 |
| MARCO | -1.337149487 | 1.031674859 | -11.67335306 | 2.93E-24 | 3.39E-24 | 42.8046967 |
| IER3 | -1.167748763 | 1.453958131 | -11.60067052 | 4.86E-24 | 5.60E-24 | 42.30147737 |
| NCF1B | -1.270524858 | 1.581114786 | -11.57427882 | 5.83E-24 | 6.72E-24 | 42.11889849 |
| RNA5SP51 | 1.128195575 | 1.204506199 | 11.56965864 | 6.02E-24 | 6.94E-24 | 42.08694395 |
| FGFBP2 | -1.074171149 | 1.4591013 | -11.56574262 | 6.19E-24 | 7.12E-24 | 42.05986153 |
| EPB42 | -1.197442061 | 1.317957682 | -11.55341447 | 6.74E-24 | 7.76E-24 | 41.97461373 |
| LTF | -1.643726995 | 1.338817836 | -11.49238326 | 1.03E-23 | 1.18E-23 | 41.55284756 |
| BAHCC1 | 1.017040791 | 2.143991331 | 11.46491306 | 1.24E-23 | 1.43E-23 | 41.36315265 |
| SELENBP1 | -1.345403061 | 1.362360319 | -11.46454063 | 1.25E-23 | 1.43E-23 | 41.36058145 |
| IGHD1-20 | 1.731619144 | 1.669388642 | 11.45653792 | 1.32E-23 | 1.51E-23 | 41.30533617 |
| SMIM25 | -1.436586855 | 1.569422248 | -11.40712948 | 1.86E-23 | 2.12E-23 | 40.96442493 |
| IGKV1-8 | -1.057276489 | 1.147925786 | -11.38743822 | 2.13E-23 | 2.43E-23 | 40.82864116 |
| BPI | -1.358023122 | 1.244636797 | -11.38619363 | 2.14E-23 | 2.45E-23 | 40.82006057 |
| TCN1 | -1.264060308 | 1.121911909 | -11.36021283 | 2.57E-23 | 2.93E-23 | 40.64098416 |
| FCGR1A | -1.074312574 | 1.618451924 | -11.35767215 | 2.61E-23 | 2.98E-23 | 40.62347667 |
| PADI2 | -1.201696542 | 1.513281365 | -11.35437302 | 2.67E-23 | 3.05E-23 | 40.60074404 |
| MIR3939 | 1.130787774 | 1.20650325 | 11.33346136 | 3.09E-23 | 3.52E-23 | 40.45668437 |
| RFX8 | 1.259390249 | 1.811694791 | 11.1052621 | 1.49E-22 | 1.69E-22 | 38.88836392 |
| CYP27A1 | -1.246756529 | 1.071421814 | -11.09865679 | 1.56E-22 | 1.77E-22 | 38.84307426 |
| IGHD5-18 | 1.722792033 | 1.662588164 | 11.09330767 | 1.62E-22 | 1.83E-22 | 38.80640234 |
| CEACAM8 | -1.238072511 | 0.99331135 | -11.04162722 | 2.30E-22 | 2.61E-22 | 38.45230725 |
| COL6A2 | -1.040324558 | 1.15057828 | -10.94494431 | 4.47E-22 | 5.05E-22 | 37.79092062 |
| GNLY | -1.113667062 | 1.94881807 | -10.93565788 | 4.77E-22 | 5.38E-22 | 37.72746774 |
| TNFRSF18 | -1.128924439 | 1.000155957 | -10.91486774 | 5.50E-22 | 6.20E-22 | 37.58545927 |
| TNNI2 | -1.117675392 | 1.23395349 | -10.83938489 | 9.22E-22 | 1.04E-21 | 37.07042981 |
| IGHD1-14 | 1.656417442 | 1.611452637 | 10.83894878 | 9.24E-22 | 1.04E-21 | 37.06745677 |
| CD24 | -1.175240079 | 1.219860955 | -10.59376136 | 4.92E-21 | 5.50E-21 | 35.40089532 |
| AC010970.1 | 1.315857054 | 1.349082134 | 10.58877426 | 5.09E-21 | 5.69E-21 | 35.36710311 |
| SIGLEC7 | -1.112701976 | 1.267911229 | -10.587121 | 5.15E-21 | 5.75E-21 | 35.35590165 |
| MMP8 | -1.421095171 | 1.185592239 | -10.55710489 | 6.32E-21 | 7.05E-21 | 35.1526156 |
| AC002454.1 | 1.172287573 | 1.716461059 | 10.54763451 | 6.74E-21 | 7.51E-21 | 35.08850983 |
| LRG1 | -1.056089797 | 1.658725324 | -10.53601889 | 7.29E-21 | 8.12E-21 | 35.00990454 |
| RHEX | 1.00670367 | 2.179417329 | 10.47813406 | 1.08E-20 | 1.20E-20 | 34.61854719 |
| MIR6774 | 1.28410275 | 1.324618359 | 10.40404126 | 1.78E-20 | 1.98E-20 | 34.11850248 |
| IL1RN | -1.187785267 | 1.596315755 | -10.39913309 | 1.84E-20 | 2.04E-20 | 34.08541379 |
| TMEM176A | -1.194203774 | 1.142895998 | -10.36889894 | 2.26E-20 | 2.50E-20 | 33.88168897 |
| GZMH | -1.019249477 | 1.551701483 | -10.3371051 | 2.80E-20 | 3.10E-20 | 33.66764208 |
| ASGR2 | -1.138217484 | 1.248895112 | -10.27337336 | 4.31E-20 | 4.77E-20 | 33.23916622 |
| CLEC4D | -1.220680077 | 1.067449929 | -10.23880719 | 5.44E-20 | 6.01E-20 | 33.00710713 |
| LIN7A | -1.325768333 | 1.073424699 | -10.20271747 | 6.94E-20 | 7.66E-20 | 32.76507392 |
| AC099489.1 | -1.114469171 | 1.444244156 | -10.13810018 | 1.07E-19 | 1.18E-19 | 32.33238186 |
| TREM1 | -1.051863471 | 1.966911496 | -10.13187213 | 1.12E-19 | 1.23E-19 | 32.29072267 |
| LILRA5 | -1.38515526 | 1.779828701 | -10.11189207 | 1.28E-19 | 1.40E-19 | 32.15713116 |
| AC246787.2 | 1.351744846 | 1.596663842 | 10.08507169 | 1.53E-19 | 1.68E-19 | 31.97793455 |
| HPGD | -1.161490133 | 1.051964709 | -10.02373312 | 2.31E-19 | 2.53E-19 | 31.56868094 |
| CLEC11A | 1.025648642 | 2.584187986 | 10.02094638 | 2.35E-19 | 2.58E-19 | 31.55010675 |
| MIR5190 | 1.112708218 | 1.192574612 | 10.00643883 | 2.59E-19 | 2.84E-19 | 31.45343814 |
| AC132807.2 | 1.18641065 | 1.249355568 | 10.00113801 | 2.69E-19 | 2.94E-19 | 31.41812829 |
| AC055748.1 | 1.2585736 | 1.611546777 | 9.986153787 | 2.97E-19 | 3.25E-19 | 31.31834847 |
| MARC1 | -1.120862309 | 1.370956728 | -9.879537351 | 6.05E-19 | 6.60E-19 | 30.60981672 |
| LRRK2 | -1.041904734 | 1.270421402 | -9.687054595 | 2.18E-18 | 2.37E-18 | 29.33721202 |
| AC125603.2 | 1.545428322 | 1.641842101 | 9.664071794 | 2.53E-18 | 2.75E-18 | 29.18584531 |
| CHST15 | -1.078717464 | 1.58794476 | -9.637831589 | 3.01E-18 | 3.27E-18 | 29.01318156 |
| CES1 | -1.332635851 | 1.041610373 | -9.561064427 | 5.00E-18 | 5.41E-18 | 28.50901541 |
| HSPA7 | -1.105745617 | 1.288867939 | -9.548867322 | 5.42E-18 | 5.86E-18 | 28.42904617 |
| IGHD2-8 | 1.470998151 | 1.468604102 | 9.532185382 | 6.05E-18 | 6.54E-18 | 28.31973306 |
| NCF1C | -1.055361663 | 1.785197038 | -9.520137678 | 6.55E-18 | 7.08E-18 | 28.24083079 |
| C1QB | -1.209089927 | 1.351713234 | -9.478248312 | 8.63E-18 | 9.32E-18 | 27.96677819 |
| AC016735.1 | 1.584418275 | 1.555983891 | 9.383866658 | 1.60E-17 | 1.73E-17 | 27.35096657 |
| SLPI | -1.327060601 | 1.450695343 | -9.374245036 | 1.71E-17 | 1.84E-17 | 27.28831959 |
| RPS12P23 | 1.062289915 | 1.466830543 | 9.32994297 | 2.28E-17 | 2.46E-17 | 27.00018401 |
| CR1 | -1.023090052 | 1.359574447 | -9.306932559 | 2.65E-17 | 2.85E-17 | 26.85073467 |
| TLR5 | -1.010048216 | 1.221704284 | -9.28695699 | 3.02E-17 | 3.25E-17 | 26.72111238 |
| AC244502.3 | 1.19618545 | 1.387179658 | 9.181263608 | 6.01E-17 | 6.45E-17 | 26.03708777 |
| IGHD2-21 | 1.333050161 | 1.362327844 | 9.11297366 | 9.37E-17 | 1.00E-16 | 25.59679337 |
| AC125603.1 | 1.503780974 | 1.544035638 | 9.045204965 | 1.45E-16 | 1.55E-16 | 25.16118138 |
| MEFV | -1.121188667 | 1.530691986 | -8.982336686 | 2.18E-16 | 2.33E-16 | 24.75826893 |
| CYTL1 | 1.017752695 | 2.152237329 | 8.972463287 | 2.32E-16 | 2.48E-16 | 24.6950983 |
| MTRNR2L1 | 1.071734446 | 1.208204213 | 8.844051717 | 5.30E-16 | 5.64E-16 | 23.8761933 |
| FCAR | -1.039329643 | 1.804879935 | -8.639082044 | 1.96E-15 | 2.07E-15 | 22.5797131 |
| IGHD6-25 | 1.351128894 | 1.376255847 | 8.632211289 | 2.04E-15 | 2.16E-15 | 22.53648802 |
| PTGDS | -1.133289506 | 1.383628109 | -8.498253058 | 4.77E-15 | 5.03E-15 | 21.69686316 |
| IGLL1 | 1.154492801 | 2.05350977 | 8.49460219 | 4.88E-15 | 5.15E-15 | 21.67406461 |
| SERPINA1 | -1.033502284 | 2.429575574 | -8.454750008 | 6.27E-15 | 6.61E-15 | 21.4254958 |
| SPINK2 | 1.228929339 | 1.987322568 | 8.420761429 | 7.76E-15 | 8.18E-15 | 21.21393103 |
| CPA3 | 1.005710494 | 2.216397509 | 8.386319181 | 9.62E-15 | 1.01E-14 | 20.9999511 |
| C1QC | -1.046623011 | 1.295416277 | -8.143622746 | 4.35E-14 | 4.58E-14 | 19.50412818 |
| MIR4740 | 1.10827701 | 1.189160774 | 8.137281034 | 4.53E-14 | 4.76E-14 | 19.46533015 |
| IPPKP1 | 1.05127478 | 1.176411374 | 8.123111318 | 4.94E-14 | 5.20E-14 | 19.37869531 |
| MIR4802 | 1.05063311 | 1.144751443 | 8.103534153 | 5.58E-14 | 5.86E-14 | 19.25912249 |
| AC245128.3 | -1.002386296 | 1.844414655 | -8.096917145 | 5.81E-14 | 6.10E-14 | 19.21873987 |
| PLBD1 | -1.093241149 | 1.856706617 | -7.935064757 | 1.57E-13 | 1.64E-13 | 18.23617793 |
| PF4 | -1.062290697 | 1.66052117 | -7.883959485 | 2.14E-13 | 2.24E-13 | 17.92804426 |
| ALAS2 | -1.051706375 | 2.1285216 | -7.858184113 | 2.50E-13 | 2.61E-13 | 17.77302657 |
| RNU6ATAC6P | 1.282949636 | 1.32372999 | 7.596021546 | 1.21E-12 | 1.26E-12 | 16.21167754 |
| LILRB4 | -1.04190053 | 1.450570596 | -7.546643029 | 1.63E-12 | 1.69E-12 | 15.92080631 |
| IGHD5-24 | 1.229316003 | 1.282410202 | 7.497583622 | 2.18E-12 | 2.26E-12 | 15.6328507 |
| IGHD4-23 | 1.177559115 | 1.242536273 | 7.443702051 | 3.00E-12 | 3.11E-12 | 15.31779473 |
| S100A12 | -1.119884354 | 2.353346835 | -7.44219715 | 3.02E-12 | 3.13E-12 | 15.3090135 |
| CD163 | -1.068869147 | 1.611313588 | -7.367883073 | 4.68E-12 | 4.85E-12 | 14.8766289 |
| IGHD1-26 | 1.154274839 | 1.224597876 | 7.102957431 | 2.19E-11 | 2.25E-11 | 13.35555054 |
| IGHJ2P | 1.002000215 | 1.107284263 | 6.642301629 | 2.96E-10 | 3.02E-10 | 10.79082527 |
